# Diastereo- and enantioselective [3 + 3] cycloaddition of spirocyclopropyl oxindoles using both aldonitrones and ketonitrones

**DOI:** 10.1038/s41467-017-01451-1

**Published:** 2017-11-20

**Authors:** Peng-Wei Xu, Jia-Kuan Liu, Lan Shen, Zhong-Yan Cao, Xiao-Li Zhao, Jun Yan, Jian Zhou

**Affiliations:** 1 Shanghai Key Laboratory of Green Chemistry and Chemical Processes, School of Chemistry and Molecular Engineering, East China Normal University, Shanghai, 200062 China; 2 State Key Laboratory of Pharmaceutical Biotechnology and MOE Key Laboratory of Model Animals for Disease Study, Model Animal Research Center, Nanjing University, Nanjing, Jiangsu 210061 China; 3 Shanghai Engineering Research Center of Molecular Therapeutics and New Drug Development, School of Chemistry and Chemical Engineering, East China Normal University, Shanghai, 200062 China; 4 State Key Laboratory of Elemento-Organic Chemistry, Nankai University, Tianjin, 300071 China

## Abstract

Optically active spirocyclic compounds play an important role in drug discovery, and new synthetic strategies for the efficient generation of spiro stereocenters are in much demand. Here we report a catalytic enantioselective cycloaddition using spirocyclic donor–acceptor cyclopropanes as a promising approach for the generation of spiro stereocenters. A diastereo- and enantioselective [3 + 3] cycloaddition of spirocyclopropyl oxindoles with both aldonitrones and ketonitrones is developed. The key to reaction development is the activation of spirocyclopropyl oxindoles by a suitable electron-withdrawing *N*-protecting group. This activation approach offers the promise of a general solution to enable spirocyclopropyl oxindoles as synthons for catalytic enantioselective synthesis of spirocyclic oxindoles featuring a C3 spiro stereocenter, a prominent structural motif in drugs and pharmaceutically active compounds. This protocol also constitutes the catalytic enantioselective reaction using unactivated achiral ketonitrones to construct tetrasubstituted carbon stereocenters.

## Abstract (web summary)

Chiral spirocyclic compounds are important structural motifs for drug discovery. Here, the authors report a synthetic route to spirocycles based on enantioselective cycloaddition of activated spirocyclopropyl oxindoles, which act as donor-acceptor cyclopropanes.

## Introduction

To enhance the reward in modern probe- and drug-discovery programs, there is a vast demand for synthetic libraries of optically active compounds recapitulating the structural features of privileged scaffolds that widely present in natural products, drugs, and pharmaceutically active compounds^1^. In this context, spirocyclic compounds have found ever-increasing utilization in drug discovery, because the conformational constraints imposed by a spiro-ring fusion often bring about improved biological activities of biomolecules^2, 3^. Therefore, efficient and enantioselective approaches capable of flexible construction of spiro stereocenters would facilitate the buildup of synthetic libraries of optically active spirocyclic compounds, which will help in the search for new lead candidates. However, the catalytic enantioselective synthesis of spirocyclic sterocenters is still a long-standing challenge in synthetic chemistry, especially when a spiro all-carbon quaternary stereocenter^4–6^ is involved. Despite the invention of some elegant protocols^7^, the exploitation of new synthetic routes for the catalytic enantioselective construction of spiro stereocenters is still highly desirable.

**Fig. 1:**
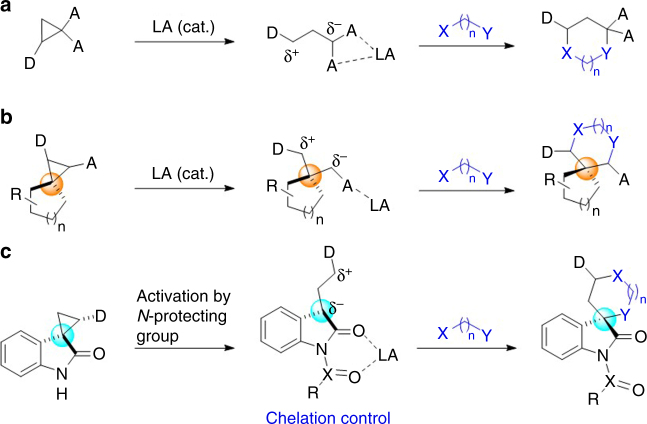
Enantioselective cycloaddition reactions using D−A cyclopropanes. **a** Well-established cyclic compounds synthesis. **b** Undeveloped spiro stereocenter generation compounds synthesis. **c** Activation of spirocyclopropyl oxindoles for cycloaddition reactions. Donor (D): electron-releasing group; Acceptor (A): electron-withdrawing group (CO_2_R or COR)

The catalytic enantioselective cycloaddition reactions of doubly activated donor–acceptor (D−A) cyclopropanes^8–13^ with different dienes, 1,3-dipoles or dipolarphiles has been established as a powerful approach for the efficient and diverse synthesis of cyclic compounds^14–31^. However, the application of this strategy to construct spirocycles is largely undeveloped, because known successful protocols all rely on the use of 2-substituted cyclopropane-1,1-dicarboxylates and analogous cyclopropyl diketones (Fig. 1a)^14–31^. This is possibly because the geminal ester or ketone groups of such cyclopropanes can effectively stabilize the negative charge in the 1,3-zwitterionic intermediates and facilitate the formation of a well-organized catalyst–substrate complex via bidentate chelation, which is often important for enantiocontrol^20–23^. The use of spirocyclic D−A cyclopropanes for cycloaddition would open new avenues to construct chiral spirocyclic scaffolds (Fig. 1b); however, although an elegant chiral cyclopropane-based version had been reported^32^, no successful catalytic enantioselective example is currently known^7–13^.

For example, spirocyclopropyl oxindoles represent a class of easily available spirocylic D–A cyclopropanes, but the use of such monoactivated D–A cyclopropanes for enantioselective catalysis is largely unexplored^33^. This is surprising, because as early as in 1999, Carreira et al.^34–37^ have successfully utilized unprotected or *N*-benzyl spirocyclopropyl oxindoles to build up the spiro[pyrrolidine-3,3′-oxindole] ring systems via MgI_2_-catalyzed annulation with imines. Since the absolute configuration and the substituent of the C3 spiro stereocenters of spirocyclic oxindoles greatly influenced the biological activities^38, 39^, it is of current interest to exploit new catalytic enantioselective methods for the synthesis of spirocyclic oxindoles that are prominent structural motifs in natural products and drugs^38–53^. While enantioselective cycloaddition using spirocyclopropyl oxindoles as D–A cyclopropanes constitutes a new entry for diverse synthesis of optically active spirocyclic oxindoles, it is difficult to make use of such monoactivated D–A cyclopropanes, for two reasons. First, with only one amide acceptor group, the activity of spirocyclopropyl oxindoles is not high. Second, a high level of transition state organization is difficult to realize simply by monodentate coordination of the amide group to a chiral Lewis acid. To tackle these two challenges, coupled with our interest in oxindole chemistry^54, 55^, we consider activating spirocyclopropyl oxindoles, easily prepared from olefin cyclopropanation using diazooxindoles^55^, by installing an electron-withdrawing *N*-protecting group. It may not only improve the stabilization of the negative charge developed at the C3 position of an oxindole via charge separation upon Lewis acid activation, but it may enable the binding of oxindole to chiral catalyst in a bidentate fashion, which is helpful for enantiocontrol. Herein, we demonstrate the power of this approach by a highly diastereo- and enantioselective [3 + 3] cycloaddition of spirocyclopropyl oxindoles and nitrones.

## Results

### Optimization of the reaction conditions

A variety of activated spirocyclopropyl oxindoles **1c**–**e** and **2a**, with different electron-withdrawing *N*-protecting groups, were readily prepared in one step from the corresponding unprotected precursor. With these spirocyclic D–A cyclopropanes at hand, we first evaluated their performance in the [3 + 3] cycloaddition reaction using nitrones, because the thus-obtained quaternary spirocyclic oxindoles, merging the structural feature of tetrahydro-1,2-oxazine, are interesting targets for medicinal research. It is worth mentioning that since the pioneering work of Young and Kerr^56^, the [3 + 3] cycloaddition reaction of D–A cyclopropanes and nitrones^56–60^ has been established as an elegant approach to access tetrahydro-1,2-oxazines, which hold potential as therapeutic agents and as synthons^61–63^. Later, Sibi^18^ and Tang^19^ independently achieved highly enantioselective versions, which subsequently prompted further studies into catalytic asymmetric synthesis based on D–A cyclopropanes^8–31^. Nevertheless, the use of aliphatic nitrones or ketonitrones for such catalytic enantioselective cycloaddition reactions were undeveloped.

The *N*-protecting group of spirocyclopropyl oxindoles in deed plays an important role in securing high reactivity, as shown in Table 1. All reactions were catalyzed by 10 mol% Ni(OTf)_2_, using 1,2-dichloroethane (DCE) as solvent at 50 °C. For the screening of Lewis acids, see Supplementary Table 1. Not unexpectedly, the reaction of unprotected oxindole **1a** with nitrone **3a** resulted in a mess, affording the desired adduct **4a** in ca. 30% NMR yield (entry 1, Table 1), and no reaction took place in the case of *N*-benzyl oxindole **1b** (entry 2, Table 1). In contrast, spirocyclopropyl oxindoles **1c** and **1d**, activated by a *N*-acetyl or benzoyl group, exhibited much higher reactivity, giving adducts **4c** and **4d** in high yields (entries 3–4, Table 1). With *N*-*p*-tolylsulfonyl group, oxindole **1e** also worked well with **3a** to give adduct **4e** in high yield and dr value (entry 5, Table 1). Interestingly, *N*-diethoxyphosphoryl oxindole **2a** showed high activity, and the corresponding reaction could complete within 6 h to give adduct **5a** in 95% yield and 20:1 dr (entry 6, Table 1). NMR analysis of these adducts **4** and **5** suggested that the *N*-protecting groups had little influence on the diastereoselectivity; X-ray analysis revealed that the major diastereomer of both *N*-diethoxyphosphoryl and *N*-Ts protected adducts have the same relative configuration. The relative configuration of the minor diastereomer of **4e** was also determined by X-ray analysis, which differed from the major diastereomer at the quaternary center of oxindole (see Supplementary Table 2). These results strongly support our working hypothesis, namely, that it is possible to activate spirocyclopropyl oxindoles by installing electron-withdrawing *N*-protecting groups (Fig. 1c).

**Table 1:** Evaluation of *N*-withdrawing protection group^a^


Entry	**1** or **2**	Solvent	Adduct	Time (h)	dr^b^	Yield (%)^c^
1	**1a**	DCE	**4a**	48	—	30
2	**1b**	DCE	**4b**	48	—	Nr
3	**1c**	DCE	**4c**	64	5:1	82
4	**1d**	DCE	**4d**	13	6:1	95
5	**1e**	DCE	**4e**	48	11:1	96
6	**2a**	DCE	**5a**	6	>20:1	95
7^d^	**2a**	DCE	**5a**	12	>20:1	87
8^e^	**2a**	DCE	**5a**	3	>20:1	90
9^f^	**2a**	DCE	**5a**	6	>20:1	95(84)

^a^0.1 mmol scale in 1.0 ml of solvent

^b^Determined by ^1^H NMR analysis

^c^NMR yield using mesitylene as the internal standard

^d^At 40 °C

^e^At 60 °C

^f^With 30 mg 3 Å MS as additive

### The elaboration of the activated spirocyclopropyl oxindoles

**Fig. 2:**
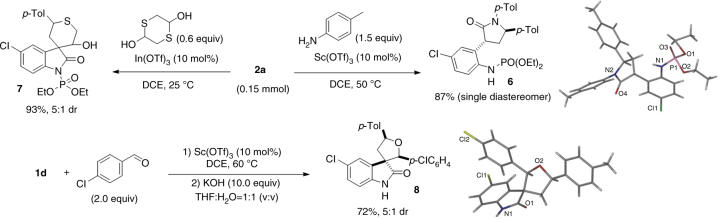
Other typical reactions of the activated spirocyclopropyl oxindoles. Ring opening reaction of **2a** using *p*-toluidine. Cyclization reaction of **2a** with 1,4-dithiane-2,5-diol. Diploar cycloaddition of **1d** with 4-chlorobenzaldehyde, followed by deprotection using KOH. Isolated yield

Notably, these activated spirocyclopropyl oxindoles could serve as viable synthons for other typical reactions of D–A cyclopropanes as well (Fig. 2). For example, *N*-diethoxyphosphoryl oxindole **2a** readily underwent ring-opening/cyclization reaction to give 3,5-disubstituted pyrrolidinone **6** in 87% yield, or reaction with 1,4-dithiane-2,5-diol to afford **7** in 93% yield and 5:1 dr. On the other hand, *N*-benzoyl oxindole **1d** was superior in the [3 + 2] cycloaddition with aldehyde. Because deprotection of the adduct occurred in the reaction course, KOH was added to facilitate the removal of protecting group after cycloaddition finished, furnishing unprotected spirocyclic oxindoles **8** in 72% yield with 5:1 dr. The relative configuration of product **6** and **8** was assigned by X-ray analysis. These results implied it possible to adjust *N*-electron-withdrawing group to develop new reactions. It should be noted that *N*-unprotected or *N*-benzyl analogs **1a** and **1b** all failed to participate in the three different kinds of reactions, confirming the importance of the activation effect of electron-withdrawing *N*-protecting group.

### Substrate scope of the reaction

The potential of these activated spirocyclopropyl oxindoles in enantioselective catalysis was further demonstrated by a highly enantioselective [3 + 3] cycloaddition using nitrones. The screening of chiral ligands (see Supplementary Table 3) revealed that bisoxazoline **L**_**1**_^64^/Ni(OTf)_2_ complex allowed the synthesis of **5a** in 41% yield and 92% ee (entry 1, Table 2). Gratifyingly, when the reaction solvent was changed from DCE to THF, both adduct **5a** and cyclopropane **2a** were obtained with excellent ee values (entries 1–3, Table 2). By adjusting the amount of nitrone **3a** to 0.55 equiv, adduct **5a** was obtained in 49% yield and 97% ee, with **2a** recovered in 46% yield and 96% ee (entry 4, Table 2). Therefore, the substrate scope was examined using THF as the solvent, in the presence of 10 mol% catalyst **L**_**1**_/Ni(OTf)_2_.

**Table 2:** Optimization for enantioselective synthesis


Entry	X	Solvent	**5a** Y/ee (%)^a, b^	**2a** R/ee (%)^b, c^	*s* ^d^ selectivity
1	0.50	DCE	41/92	40/78	7
2	0.50	Toluene	43/96	41/84	10
3	0.50	THF	44/96	48/90	33
4	0.55	THF	49/97	46/96	39

^a^Y/ee: isolated yield and ee value of 5a

^b^Ee value determined by chiral HPLC analysis

^c^R/ee: the recovery and ee value of 2a

^d^s = ln[(1 − C)(1 − ee)]/ln[(1 − C)(1 + ee)]; C refers to the conversion of (±)−2a (1-(yield of recovered 2a))

It emerged that both aromatic and aliphatic substituted nitrones were viable substrates for this kinetic resolution, with >20:1 dr values achieved in most cases (Table 3). Both electron-deficient and electron-rich α-aryl nitrones **3a**–**g** worked well to afford the desired adducts **5a**–**g** with excellent ee values (entries 1–7, Table 3). Nitrones **3h**–**j** with a 2-naphthyl, furyl, or 2-thienyl group all afforded the corresponding adducts **5h**–**j** in excellent yields and ee values (entries 8–10, Table 3). In these cases, spirocyclopropyl oxindole **2a** was recovered with an excellent ee value and good yield. Interestingly, aliphatic nitrones **3k**, **l** also gave the desired 1,2-oxazine adducts **5k**, **l** in good yields and with >90% ee; cyclopropane **2a**, however, was recovered with lower ee values (entries 11–12, Table 3). To our knowledge, aliphatic nitrones have not been used for such catalytic enantioselective cycloaddition previously^18, 19, 56–60^.

**Table 3:** Scope of different nitrones


Entry	Nitrone **3**	X	**5**: Y/ee(%)^a, b^	**2a**: R/ee(%)^b, c^	*s* ^d^ selectivity
1	**3a**: R1 = Ph	0.55	**5a**: 42/96	45/97	36
2	**3b**: R^1^ = *p*-Tolyl	0.56	**5b**: 45/98	48/97	76
3	**3c**: R^1^ = *p*-MeOC_6_H_4_	0.55	**5c**: 37/96	42/99	31
4	**3d**: R^1^ = *p*-ClC_6_H_4_	0.55	**5d**: 41/97	41/97	21
5	**3e**: R^1^ = *p*-BrC_6_H_4_	0.55	**5e**: 40/97	50/92	79
6	**3f**: R^1^ = *p*-FC_6_H_4_	0.57	**5f**: 45/95	47/99	80
7	**3g**: R^1^ = *m*-MeOC_6_H_4_	0.55	**5g**: 46/95	49/90	42
8	**3h**: R^1^ = 2-naphthyl	0.56	**5h**: 42/97	48/98	91
9	**3i**: R^1^ = 2-furyl	0.56	**5i**: 48/98	44/99	41
10	**3j**: R^1^ = 2-thienyl	0.56	**5j**: 42/97	48/90	33
11	**3k**: R^1^ = *i*-Pr	0.57	**5k**: 42/93	40/77	7
12^e^	**3l**: R^1^ = Cy	0.56	**5l**: 36/90	45/79	11

^a^Y/ee: isolated yield and ee value of **5**

^b^Ee determined by chiral HPLC analysis

^c^R/ee: the recovery and ee value of **2a**

^d^ *s* = ln[(1 − *C*)(1 − ee)]/ln[(1 − *C*)(1 + ee)]; *C* refers to the conversion of (±)−**2a** (1-(yield of recovered **2a**))

^e^13:1 dr

On the other hand, the substituents on oxindole framework of cyclopropane **2** were found to have an influence on the reaction outcome (Table 4). With electron-withdrawing groups on the C5 or C6 positions, oxindoles **2c**–**e** gave the desired adducts **5n**–**p** in 38–44% yield and 95–98% ee, together with the recovery of cyclopropanes **2c**–**e** in good yields and with >90% ee (entries 2–4, Table 4). In contrast, electron-donating groups retarded the reaction. The reactions of **2f**, **g** were run at 90 °C and gave adducts **5q**, **r** with excellent ee values and obviously in lower yields. Cyclopropanes **2f**, **g** were recovered with lower ee values (entries 5 and 6, Table 4). Oxindoles **2h**–**k** with different donor groups (R^3^) of the cyclopropane were also tried. As expected, with the R^3^ group varying from the *p*-tolyl to phenyl and *p*-chlorophenyl groups, the reaction temperature increased from 50 to 60 and 70 °C (entry 1, Table 3 vs. entries 7–8, Table 4). The adducts **5s**–**v** were all prepared satisfactorily. Cyclopropanes **2h**–**k** were recovered in high yields and excellent ee values (entries 7–10, Table 4).

**Table 4:** Scope of spirocyclopropyl oxindoles


Entry	Cyclopropane **2** (R^2^, R^3^)	X	Temp. (°C)	**5**: Y/ee(%)^a, b^	**2**: R/ee(%)^b, c^	*s* ^d^ selectivity
1	**2b**: H, *p*-Tolyl	0.57	50	**5m**: 31/95	39/90	11
2	**2c**: 5-F, *p*-Tolyl	0.56	50	**5n**: 40/98	50/92	79
3	**2d**: 5-Br, *p*-Tolyl	0.56	50	**5o**: 44/96	49/99	211
4	**2e**: 6-Br, *p*-Tolyl	0.56	50	**5p**: 38/95	34/99	15
5	**2f**: 5-Me, *p*-Tolyl	0.57	90	**5q**: 34/92	47/70	9
6	**2g**: 5-OMe, *p*-Tolyl	0.57	90	**5r**: 35/90	52/70	15
7	**2h**: 5-Cl, Ph	0.57	60	**5s**: 48/95	48/99	116
8	**2i**: 5-Cl, 4-ClC_6_H_4_	0.57	70	**5t**: 46/97	48/94	50
9	**2j**: 5-Cl, 2-naphthyl	0.57	60	**5u**: 47/93	44/99	41
10	**2k**: 5-Cl, (*E*)-PhCH=CH	0.54	40	**5v**: 48/90	40/98	21

^a^Y/Ee: isolated yield and ee value of **5**

^b^Determined by HPLC analysis

^c^R/ee: the recovery and ee value of **2**

^d^ *s* = ln[(1 − *C*)(1 − ee)]/ln[(1 − *C*)(1 + ee)]; *C* refers to the conversion of (±)−**2** (1-(yield of recovered **2**))

**Fig. 3:**
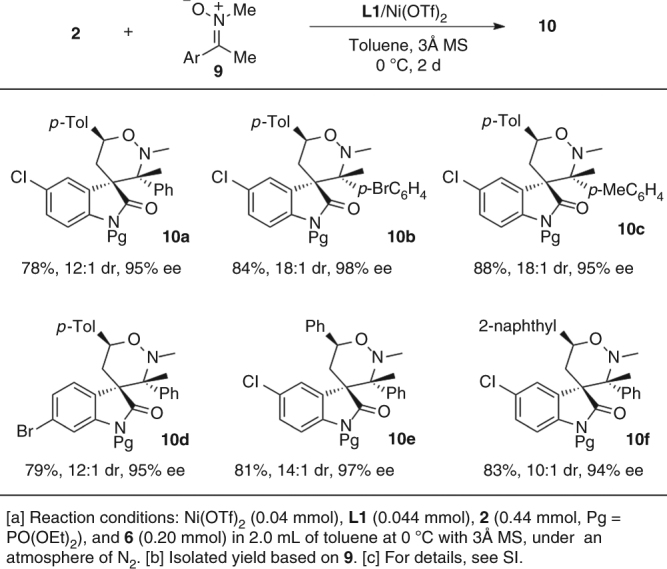
Cycloaddition of spirocyclopropyl oxindoles using ketonitrones. Construction of adjacent quaternary and tetrasubstituted carbon stereocenters

Notably, acetophenone-derived ketonitrones **9** are also viable substrates, enabling highly stereoselective synthesis of spirocylic oxindoles **10a**–**f** with adjacent quaternary and tetrasubstituted carbon stereocenters (Fig. 3). It is worth mentioning that enantioselective catalytic reactions based on such unactivated achiral ketonitrones to create tetrasubstituted carbon stereocenters is unprecedented^65^, although two protocols using activated ketonitrones have been reported^66, 67^.

### Proposed working model

**Fig. 4:**
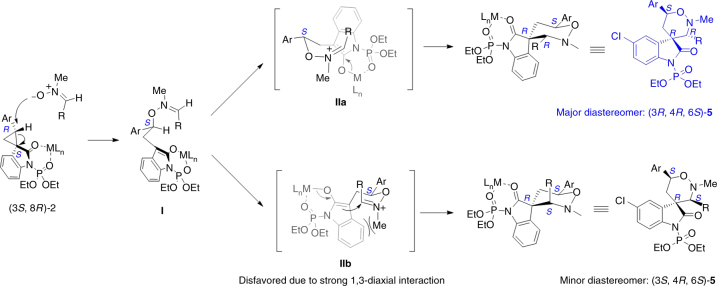
Working model for the observed 3,6-trans selectivity of adducts **5**. A stepwise annulation mechanism. The favored boat-like transition state **IIa** is possibly stabilized by the cation–π interaction. The chiral-like transition state **IIb** is presumably destabilized by the strong 1,3-diaxial repulsion

The relative configuration of racemic compound **4e** and **5o** was assigned by X-ray analysis. By converting to the corresponding *N*-*p*-tolylsulfonyl analogs, the relative and absolute configuration of compound **5a** and **10a** were also determined by X-ray analysis, as shown in Table 3 and Fig. 3 (for details, see Supplementary Figs. 1 and 2). Those of others were tentatively assigned by NMR analysis. Interestingly, no matter using *N*-*p*-tolylsulfonyl or *N*-diethoxyphosphoryl spirocyclic oxindoles, the cycloaddition reaction with nitrones afforded the major diastereomers of corresponding adducts in which the substituents at C3 and C6 of the tetrahydro-1,2-oxazine bore a *trans* relationship, different from the 3,6-*cis* selectivity of the known [3 + 3] cycloaddition of 2-substituted cyclopropane-1,1-dicarboxylates and nitrones^19, 56^. The observed diastereoselectivity could be rationalized by the following working model involving a stepwise annulation mechanism (Fig. 4). According to previous studies in the Lewis acid catalyzed [3 + n] annulations of D–A cyclopropanes^21, 58^, we proposed that the binding of the *N*-diethoxyphosphoryl oxindole **2** to the Lewis acid facilitated the *O*-attack of nitrones at the donor-substituted site of cyclopropane, leading to the reversion at that position, which was consistent with our experiments that under the catalysis of 10 mol% Ni(OTf)_2_, chiral cyclopropane (3*S*, 8*R*)-**2b** with 90% ee afforded tetrahydro-1,2-oxazine (3*R*, 4*R*, 6*S*)-**5m** as the major product with 90% ee. The stepwise mechanism was also supported by the fact that the ^1^H NMR analysis of the reaction mixture of nitrone **3a** with *N*-*p*-tolylsulfonyl oxindole **1e** could obviously detect the presence and the gradual disappearance of the intermediate correlating to the nucleophilic *O*-attack of nitrones to the donor-substituted site of cyclopropane (for details, see Supplementary Figs. 15 and 16). The resulting intermediate **I** further underwent an intramolecular Mannich cyclization to afford the desired product with 3,6-*trans* tetrahydro-1,2-oxazine, via a favored boat-like transition state **IIa**, which is possibly stabilized by the cation–π interaction between the iminium species and enolate^68^. The chiral-like transition state **IIb**, leading to the formation of 3,6-*cis* tetrahydro-1,2-oxazine, is presumably destabilized by the strong 1,3-diaxial repulsion between the *N*-methyl group of nitrone and the aromatic framework of oxindole.

### Synthetic application

**Fig. 5:**
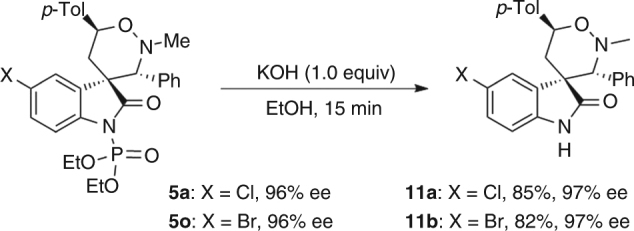
Deprotection of product **5a** and **5o**. Reaction conditions: **5** (0.1 mmol), KOH (1.0 mmol) in 2.0 mL of EtOH at room temperature. Isolated yield

Impressively, our protocol provides a facile access to optically active oxindole-based spirocyclic tetrahydro-1,2-oxazines, and spirocyclopropyl oxindoles as well. It is noteworthy that optically active spirocyclopropyl oxindoles have wide application^33^. On the other hand, the *N*-diphenoxyphosphoryl group of adducts **5** could be readily removed, as evidenced by the conversion of **5a** and **5o** to the corresponding spirocyclic 1,2-oxazine **11a** and **11b** (Fig. 5).

## Discussion

In summary, we have demonstrated that spirocyclic oxindoles could be effectively activated by electron-withdrawing *N*-protecting group to serve as D−A cyclopropanes for complexity-generating synthesis. A highly enantioselective [3 + 3] cycloaddition and kinetic resolution of *N*-diethoxyphosphoryl spirocyclopropyl oxindoles is developed, providing a facile access to optically active oxindole-based spirocyclic tetrahydro-1,2-oxazines and spirocyclopropyl oxindoles of wide application^33^. This work also implies that catalytic enantioselective cycloaddition using spirocyclic D–A cyclopropanes is a promising approach for the flexible synthesis of chiral spirocyclic scaffolds. In addition, our work also suggests new synthetic opportunities of ketonitrones in creating tetrasubstituted carbon stereocenters. The evaluation of the biological activities of these spirocyclopropyl oxindoles is now in progress in our laboratories. The application of this strategy to explore other enantioselective cycloaddition additions is now under way in our laboratory.

## Methods

### General methods

See Supplementary Methods for further details.

### General procedure for catalytic enantioselective [3 + 3] cycloaddition of **2** and aldonitrone **3**

To a Schlenk tube was sequentially added Ni(OTf)_2_ (10.7 mg, 0.030 mmol, 10 mol%) and **L**_**1**_ (11.8 mg, 0.033 mmol, 11 mol%), followed by the addition of anhydrous THF (3.0 ml). After the resulting solution was stirred at 50 °C for 2 h, oxindole **2** (0.30 mmol), nitrone **3**, and MS 3 Å (90 mg) were added successively. The reaction was kept stirring at the temperature indicated in Tables 3 and 4 till the full consumption of **3** by TLC analysis. Then THF was removed under reduced pressure. The residue was dissolved in CH_2_Cl_2_, rapidly passed through a glass funnel with a thin layer (5 mm) of silica gel (100 mesh), washed with CH_2_Cl_2_, and concentrated under reduced pressure. To determine the dr value of product, the residue was first dissolved in CDCl_3_, and took some samples to determine diastereoselectivity by ^1^H NMR analysis. Then the sample for analysis and rest crude product were recombined for column chromatography purification to afford product **5** and recovered spirocyclopropyl oxindole **2**, using DCM/EtOAc (40/1, v/v) as the eluent.

### General procedure for catalytic enantioselective [3 + 3] cycloaddition of **2** and ketonitrone **9**

To a Schlenk tube was sequentially added Ni(OTf)_2_ (14.3 mg, 0.040 mmol) and **L**_**1**_ (15.8 mg, 0.044 mmol), followed by the addition of anhydrous toluene (2.0 ml). After the resulting solution was stirred at room temperature for 2 h and cooled to 0 °C, nitrone **9** (0.2 mmol), oxindole **2** (0.44 mmol), and MS 3 Å (60 mg) were added successively. The reaction was kept stirring at 0 °C for 2 days. Then toluene was removed under reduced pressure. The residue was dissolved in CH_2_Cl_2_, rapidly passed through a glass funnel with a thin layer (5 mm) of silica gel (100 mesh), washed with CH_2_Cl_2_, and concentrated under reduced pressure. To determine the dr value of product, the residue was first dissolved in CDCl_3_, and took some samples to determine diastereoselectivity by ^1^H NMR analysis. Then the sample for analysis and rest crude product were recombined for column chromatography purification to afford product **10**, using DCM/EtOAc (30/1, v/v) as the eluent.

### Data availability

The X-ray crystallographic coordinates for structures reported in this study have been deposited at the Cambridge Crystallographic Data Centre (CCDC), under deposition numbers CCDC-1502102 (racemic major-**4e**), CCDC-1551103 (minor**-4e**), CCDC-1502103 (major-**5o**) CCDC-1502104 (**4e**), CCDC-1502105 (**12a**), CCDC-1523864 (**6**), and CCDC-1523855 (**8**). These data can be obtained free of charge from The Cambridge Crystallographic Data Centre via www.ccdc.cam.ac.uk/data_request/cif. The authors declare that all other data supporting the findings of this study are available within the article and Supplementary Information files, and also are available from the corresponding author on reasonable request.

## Electronic supplementary material

Supplementary Information

## References

[1] Sharma I, Tan DS (2013). Drug discovery: diversifying complexity. Nat. Chem..

[2] Zheng Y, Tice CM, Singh SB (2014). The use of spirocyclic scaffolds in drug discovery. Bioorg. Med. Chem. Lett..

[3] Carreira EM, Fessard TC (2014). Four-membered ring-containing spirocycles: synthetic strategies and opportunities. Chem. Rev..

[4] Quasdorf KW, Overman LE (2014). Catalytic enantioselective synthesis of quaternary carbon stereocentres. Nature.

[5] Liu Y, Han S-J, Liu W-B, Stoltz BM (2015). Catalytic enantioselective construction of quaternary stereocenters: assembly of key building blocks for the synthesis of biologically active molecules. Acc. Chem. Res..

[6] Zeng X-P, Cao Z-Y, Wang Y-H, Zhou F, Zhou J (2016). Catalytic enantioselective desymmetrization reactions to all-carbon quaternary stereocenters. Chem. Rev..

[7] Rios R (2012). Enantioselective methodologies for the synthesis of spiro compounds. Chem. Soc. Rev..

[8] Grover HK, Emmett MR, Kerr MA (2015). Carbocycles from donor–acceptor cyclopropanes. Org. Biomol. Chem..

[9] Schneider TF, Kaschel J, Werz DB (2014). A new golden age for donor–acceptor cyclopropanes. Angew. Chem. Int. Ed..

[10] Campbell MJ, Johnson JS, Parsons AT, Pohlhaus PD, Sanders SD (2010). Complexity-building annulations of strained cycloalkanes and C=O π Bonds. J. Org. Chem..

[11] Carson CA, Kerr MA (2009). Heterocycles from cyclopropanes: applications in natural product synthesis. Chem. Soc. Rev..

[12] de Simone, F. & Waser, J. Cyclization and cycloaddition reactions of cyclopropyl carbonyls and imines. *Synthesis* 3353–3374 (2009).

[13] Reissig H-U, Zimmer R (2003). Donor-acceptor-substituted cyclopropane derivatives and their application in organic synthesis. Chem. Rev..

[14] Moran J, Smith AG, Carris RM, Johnson JS, Krische MJ (2011). Polarity inversion of donor-acceptor cyclopropanes: disubstituted δ-lactones via enantioselective iridium catalysis. J. Am. Chem. Soc..

[15] Zhou Y-Y, Wang L-J, Li J, Sun X-L, Tang Y (2012). Side-arm-promoted highly enantioselective ring-opening reactions and kinetic resolution of donor−acceptor cyclopropanes with amines. J. Am. Chem. Soc..

[16] Wales SM, Walker MM, Johnson JS (2013). Asymmetric synthesis of indole homo-Michael adducts via dynamic kinetic Friedel-Crafts alkylation with cyclopropanes. Org. Lett..

[17] Xia Y, Liu X, Zheng H, Lin L, Feng X (2015). Asymmetric synthesis of 2,3-dihydropyrroles by ring-opening/cyclization of cyclopropyl ketones using primary amines. Angew. Chem. Int. Ed..

[18] Sibi MP, Ma Z, Jasperse CP (2005). Enantioselective addition of nitrones to activated cyclopropanes. J. Am. Chem. Soc..

[19] Kang Y-B, Sun X-L, Tang Y (2007). Highly enantioselective and diastereoselective cycloaddition of cyclopropanes with nitrones and its application in the kinetic resolution of 2-substituted cyclopropane-1,1-dicarboxylates. Angew. Chem. Int. Ed..

[20] Parsons AT, Johnson JS (2009). Catalytic enantioselective synthesis of tetrahydrofurans: a dynamic kinetic asymmetric [3+2] cycloaddition of racemic cyclopropanes and aldehydes. J. Am. Chem. Soc..

[21] Parsons AT, Smith AG, Neel AJ, Johnson JS (2010). Dynamic kinetic asymmetric synthesis of substituted pyrrolidines from racemic cyclopropanes and aldimines: reaction development and mechanistic insights. J. Am. Chem. Soc..

[22] Nanteuil D, Serrano FE, Perrotta D, Waser J (2014). Dynamic kinetic asymmetric [3+2] annulation reactions of aminocyclopropanes. J. Am. Chem. Soc..

[23] Xiong H, Xu H, Liao S, Xie Z, Tang Y (2013). Copper-catalyzed highly enantioselective cyclopentannulation of indoles with donor−acceptor cyclopropanes. J. Am. Chem. Soc..

[24] Xu H, Qu J-P, Liao S, Xiong H, Tang Y (2013). Highly enantioselective [3+2] annulation of cyclic enol silyl ethers with donor–acceptor cyclopropanes: accessing *3α*-hydroxy [n.3.0]carbobicycles. Angew. Chem. Int. Ed..

[25] Trost BM, Morris PJ (2011). Palladium-catalyzed diastereo- and enantioselective synthesis of substituted cyclopentanes through a dynamic kinetic asymmetric formal [3+2]-cycloaddition of vinyl cyclopropanes and alkylidene azlactones. Angew. Chem. Int. Ed..

[26] Trost BM, Morris PJ, Sprague SJ (2012). Palladium-catalyzed diastereo- and enantioselective formal [3+2]-cycloadditions of substituted vinylcyclopropanes. J. Am. Chem. Soc..

[27] Hashimoto T, Kawamata Y, Maruoka K (2014). An organic thiyl radical catalyst for enantioselective cyclization. Nat. Chem..

[28] Halskov KS, Næsborg L, Tur F, Jørgensen KA (2016). Asymmetric [3+2] cycloaddition of vinylcyclopropanes and *α,β*-unsaturated aldehydes by synergistic palladium and organocatalysis. Org. Lett..

[29] Meazza M, Rios R (2016). Synergistic catalysis: enantioselective ring expansion of vinyl cyclopropanes combining four catalytic cycles for the synthesis of highly substituted spirocyclopentanes bearing up to four stereocenters. Chem. Eur. J..

[30] Xu H, Hu J-L, Wang L, Liao S, Tang Y (2015). Asymmetric annulation of donor−acceptor cyclopropanes with dienes. J. Am. Chem. Soc..

[31] Halskov KS (2015). Organocatalytic enamine-activation of cyclopropanes for highly stereoselective formation of cyclobutanes. J. Am. Chem. Soc..

[32] Carson CA, Kerr MA (2009). Total synthesis of FR901483. Org. Lett..

[33] Cao Z-Y, Zhou J (2015). Catalytic asymmetric synthesis of polysubstituted spirocyclopropyl oxindoles: organocatalysis versus transition metal catalysis. Org. Chem. Front..

[34] Alper PB, Meyers C, Lerchner A, Siegel DR, Carreira EM (1999). Facile, novel methodology for the synthesis of spiro[pyrrolidin-3,3′-oxindoles]: catalyzed ring expansion reactions of cyclopropanes by aldimines. Angew. Chem. Int. Ed..

[35] Lerchner A, Carreira EM (2002). First total synthesis of (±)-strychnofoline via a highly selective ring-expansion reaction. J. Am. Chem. Soc..

[36] Marti C, Carreira EM (2005). Total synthesis of (−)-spirotryprostatin B: synthesis and related studies. J. Am. Chem. Soc..

[37] Marti, C. & Carreira, E. M. Construction of spiro[pyrrolidine-3,3′-oxindoles]-recent applications to the synthesis of oxindole alkaloids. *Eur. J. Org. Chem*. 2209–2219 (2003).

[38] Shen K, Liu X, Lin L, Feng X (2012). Recent progress in enantioselective synthesis of C3-functionalized oxindoles: rare earth metals take action. Chem. Sci..

[39] Hong L, Wang R (2013). Recent advances in asymmetric organocatalytic construction of 3,3′-spirocyclic oxindoles. Adv. Synth. Catal..

[40] Trost BM, Cramer N, Silverman SM (2007). Enantioselective construction of spirocyclic oxindolic cyclopentanes by Palladium-catalyzed trimethylenemethane-[3+2]-cycloaddition. J. Am. Chem. Soc..

[41] Bencivenni G (2009). Targeting structural and stereochemical complexity by organocascade catalysis: construction of spirocyclic oxindoles having multiple stereocenters. Angew. Chem. Int. Ed..

[42] Chen X-H, Wei Q, Luo S-W, Xiao H, Gong L-Z (2009). Organocatalytic synthesis of spiro[pyrrolidin-3,3′-oxindoles] with high enantiopurity and structural diversity. J. Am. Chem. Soc..

[43] Antonchick AP (2010). Highly enantioselective synthesis and cellular evaluation of spirooxindoles inspired by natural products. Nat. Chem..

[44] Tan B, Candeias NR, Barbas CF (2011). Construction of bispirooxindoles containing three quaternary stereocentres in a cascade using a single multifunctional organocatalyst. Nat. Chem..

[45] Jia Z-J (2011). Trienamines in asymmetric organocatalysis: diels−Alder and tandem reactions. J. Am. Chem. Soc..

[46] Lan Y-B (2011). Chiral counteranion synergistic organocatalysis under high temperature: efficient construction of optically pure spiro[cyclohexanone-oxindole] backbone. Org. Lett..

[47] Shen L-T, Jia W-Q, Ye S (2013). Catalytic [4+2] cyclization of *α,β*-unsaturated acyl chlorides with 3-alkylenyloxindoles: highly diastereo- and enantioselective synthesis of spirocarbocyclic oxindoles. Angew. Chem. Int. Ed..

[48] Manoni F, Connon SJ (2014). Catalytic asymmetric tamura cycloadditions. Angew. Chem. Int. Ed..

[49] Xiang B, Belyk KM, Reamer RA, Yasuda N (2014). Discovery and application of doubly quaternized cinchona-alkaloid-based phase-transfer catalysts. Angew. Chem. Int. Ed..

[50] Shi F, Zhu R-Y, Dai W, Wang C-S, Tu S-J (2014). Catalytic asymmetric formal [3+3] cycloaddition of an azomethine ylide with 3-indolylmethanol: enantioselective construction of a six-membered piperidine framework. Chem. Eur. J..

[51] Stiller JP (2014). Organocatalytic [4+2] addition reactions via tetraenamine intermediate. Chem. Sci..

[52] Sun Q-S (2015). Squaramide-catalyzed synthesis of enantioenriched spirocyclic oxindoles via ketimine intermediates with multiple active sites. Angew. Chem. Int. Ed..

[53] Zhan G (2016). Catalyst-controlled switch in chemo- and diastereoselectivities: annulations of Morita–Baylis–Hillman carbonates from isatins. Angew. Chem. Int. Ed..

[54] Yin X-P (2014). Asymmetric triple relay catalysis: enantioselective synthesis of spirocyclic indolines through a one-pot process featuring an asymmetric 6π electrocyclization. Angew. Chem. Int. Ed..

[55] Cao Z-Y (2013). Highly stereoselective olefin cyclopropanation of diazooxindoles catalyzed by a C2‑symmetric spiroketal bisphosphine/Au(I) complex. J. Am. Chem. Soc..

[56] Young IS, Kerr MA (2003). A homo [3+2] dipolar cycloaddition: the reaction of nitrones with cyclopropanes. Angew. Chem. Int. Ed..

[57] Jackson SK, Karadeolian A, Driega AB, Kerr MA (2008). Stereodivergent methodology for the synthesis of complex pyrrolidines. J. Am. Chem. Soc..

[58] Karadeolian A, Kerr MA (2007). Examination of homo-[3+2]-dipolar cycloaddition: mechanistic insight into regio- and diastereoselectivity. J. Org. Chem..

[59] Hardman AM, So SS, Mattson AE (2013). Urea-catalyzed construction of oxazinanes. Org. Biomol. Chem..

[60] Chidley T, Vemula N, Carson CA, Kerr MA, Pagenkopf BL (2016). Cascade reaction of donor–acceptor cyclopropanes: mechanistic studies on cycloadditions with nitrosoarenes and cis-diazenes. Org. Lett..

[61] Yu Q-S (2002). Anticholinesterase activity of compounds related to geneserinetautomers. *N*-oxides and 1,2-oxazines. J. Med. Chem..

[62] Uchida I, Takase S, Kayakiri H, Kiyoto S, Hashimoto M (1987). Structure of FR 900482, a novel antitumor antibiotic from a *streptomyces*. J. Am. Chem. Soc..

[63] Pulz R, Al-Harrasi A, Reissig H-U (2002). New polyhydroxylated pyrrolidines derived from enantiopure 3,6-dihydro-2H-1,2-oxazines. Org. Lett..

[64] Davies IW, Senanayake CH, Larsen RD, Verhoeven TR, Reider PJ (1996). Application of a Ritter-type reaction to the synthesis of chiral indane-derived C_2_-symmetric bis(oxazolines). Tetrahedron Lett..

[65] Shi W-M, Ma X-P, Su G-F, Mo D-L (2016). New developments of ketonnitrones in organic synthesis. Org. Chem. Front..

[66] Liu R-R (2015). Dual catalysis for the redox annulation of nitroalkynes with indoles: enantioselective construction of indolin-3-ones bearing quaternary stereocenters. Angew. Chem. Int. Ed..

[67] Selim KB (2014). Enantioselective ruthenium-catalyzed 1,3-dipolar cycloadditions between C‑carboalkoxy ketonitrones and methacrolein: solvent effect on reaction selectivity and its rational. J. Org. Chem..

[68] McCurdy A, Jimenez L, Stauffer DA, Dougberty DA (1992). Biomimetic catalysis of S_N_2 reactions through cation-π Interactions. the role of polarizability in catalysis. J. Am. Chem. Soc..

