# Supplementary material for: Diastereo- and enantioselective [3 + 3] cycloaddition of spirocyclopropyl oxindoles using both aldonitrones and ketonitrones
Source: Nat Commun. 2017 Nov 20;8:1619. doi: 10.1038/s41467-017-01451-1 (PMC5696380; doi:10.1038/s41467-017-01451-1)
Supplement: Supplementary file 1 — Supplementary Information [file 41467_2017_1451_MOESM1_ESM.pdf]

Supplementary Methods

General information

Reactions were monitored by thin layer chromatography using UV light to visualize the course of reaction. Purification of reaction products was carried out by flash chromatography on silica gel. Chemical yields refered to pure isolated substances. The $[\alpha]_D$ were recorded using PolAAr 3005 High Accuracy Polarimeter. Infrared (IR) spectra were obtained using a Bruker tensor 27 infrared spectrometer. ^{1}H NMR, ^{13}C NMR, ^{31}P NMR and ^{19}F NMR spectra were obtained using Bruker DPX-400 or DPX-300 MHz Spectrometer. Chemical shifts were reported in ppm from tetramethylsilane with the solvent resonance or sample of 85% H_3PO_4 as the internal standard. The following abbreviations were used to designate chemical shift multiplicities: s = singlet, d = doublet, t = triplet, q = quartet, h = heptet, m = multiplet, br = broad.

All reactions were run in an atmosphere of N_2 except noted. Anhydrous CH_2ClCH_2Cl was prepared by first distillation over P_2O_5 and then from CaH_2. Toluene and THF were prepared by distillation over sodium-benzophenone ketyl prior to use. Activated molecular sieves powder 3Å (MS 3 Å) was dried at 150 °C in vacuum before use. $Ni(OTf)_2$ was purchased from Strem Chemicals and used as it received. All the nitrones[1,2] and racemic *N*-unprotected spirocyclopropyl oxindoles[3] were synthesized according to the literature procedures.

.

General procedure for synthesis of *N*-protected spiropropyl oxindoles 2

All the *N*-diethoxyphosphoryl spirocyclopropyl oxindoles **2a-k** are prepared according to the described general procedure. To a stirred solution of unprotected spirocyclopropyl oxindoles **I** (5.0 mmol) in 1 mL of THF was added NaH (240 mg, 60% in mineral oil, 1.2 equivs) in one portion. After the mixture was stirred at room temperature for 30 minutes, diethyl phosphorochloridate (1.2 equivs) was added dropwise. The resulting mixture was kept stirring for another 15 minutes, and then saturated NH_4Cl (10 mL) was added. After extraction with ethyl acetate (3 × 15 mL), the combined organic layer was dried by Na_2SO_4, and concentrated in vacuum. The residue was purified by silica gel column chromatography to afford *N*-diethoxyphosphoryl spirocyclopropyl oxindoles **2a-k** in 60-80% yield. The full characterization of compounds **2a-k**, see corresponding enantioenriched samples.

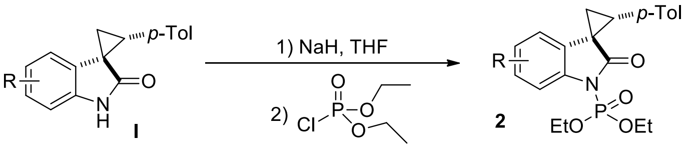

Other *N*-protected spirocyclopropyl oxindoles **1a-e** are prepared from unprotected oxindoles **I** and the corresponding acid chlorides, sulfonyl chlorides, or diphenylphosphinic chloride. Compound **1e** was first used for the screening of different Lewis acids.

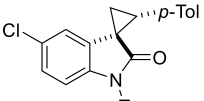

1e

1e was synthesized in 90% yield as a white solid (m.p. 197-198 °C); ^{1}H NMR (400 MHz, $CDCl_3$): 7.99 (d, J = 8.4 Hz, 2H), 7.88 (d, J = 8.8 Hz, 1H), 7.35 (d, J = 8.4 Hz, 2H), 7.15 (dd, J_1 = 8.8 Hz, J_2 = 2.4 Hz, 1H), 7.10 (d, J = 8.0 Hz, 2H), 6.95 (d, J = 8.0 Hz, 2H), 5.88 (d, J = 2.0 Hz, 1H), 3.31 (t, J = 8.8 Hz, 1H), 2.44 (s, 3H), 2.33 (s, 3H), 2.21 (dd, J_1 = 9.2 Hz, J_2 = 4.8 Hz, 1H), 1.95 (dd, J_1 = 8.4 Hz, J_2 = 4.8 Hz, 1H); ^{13}C NMR (100 MHz, $CDCl_3$): 174.19, 145.79, 138.00, 137.50, 135.25, 130.02, 129.88, 129.66, 129.51, 129.37, 128.48, 127.95, 127.11, 121.12, 114.35, 39.13, 33.54, 24.70, 21.80, 21.20; IR (ATR): 3034, 2921, 1745, 1440, 1371, 1281, 1238, 1190, 1174, 1137, 1089, 1062 cm^{-1}; MS (EI): 437, 439 (M^+, 33, 10), 282 (100), 145 (87), 91 (87), 105 (49), 130 (46), 43 (40), 77 (38); HRMS (EI): Exact mass calcd for $C_{24}H_{20}NO_3S^{35}Cl$ $[M]^+$: 437.0852, Found: 437.0854.

Catalytic enantioselective [3+3] cycloaddition of 2 and aldonitrone 3

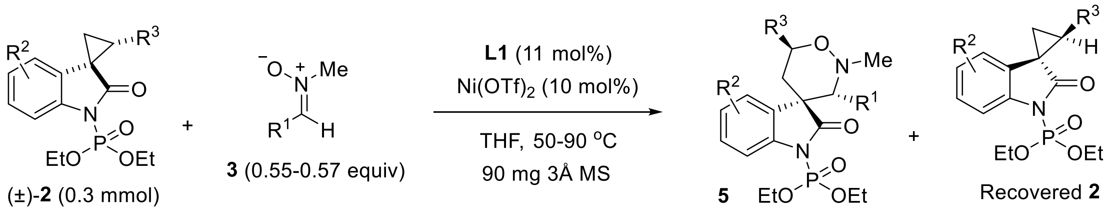

General procedure: To a Schlenk tube was sequentially added $Ni(OTf)_2$ (10.7 mg, 0.030 mmol, 10 mol %) and **L_1** (11.8 mg, 0.033 mmol, 11 mol %), followed by the addition of anhydrous THF (3.0 mL). After the resulting solution was stirred at 50 °C for 2 h, oxindole **2** (0.30 mmol), nitrone **3** and MS 3Å (90 mg) were added successively. The reaction was kept stirring at the temperature indicated in Table 3-4 till the full consumption of **3** by TLC analysis. Then THF was removed under reduced pressure. The residue was dissolved in CH_2Cl_2, rapidly passed through a glass funnel with a thin layer (5 mm) of silica gel (100 mesh), washed with CH_2Cl_2, and concentrated under reduced pressure. To determine the dr value of product, the residue was first dissolved in $CDCl_3$, and took some samples to determine diastereoselectivity by ^{1}H NMR analysis. Then the sample for analysis and rest crude product were recombined for column chromatography purification to afford product **5** and recovered spirocyclopropyl oxindole **2**, using DCM/EtOAc (40/1, v/v) as the eluent.

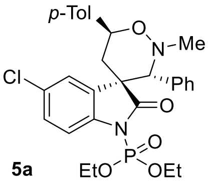

5a

5a was obtained in 42% yield as a white solid (m.p. 134-137 °C); NMR analysis showed the dr value was above 20:1. HPLC analysis (Chiralcel IC, ^{i}PrOH/hexane = 10/90, 1.0 mL/min, 230 nm; t_r (major) = 9.57 min, t_r (minor) = 12.49 min) gave the isomeric composition of the product: 96% ee, $[\alpha]^{25}_D$ = -55.2 (c = 1.0, $CHCl_3$); ^{1}H NMR (400 MHz, $CDCl_3$): 8.36 (s, 1H), 7.58 (d, J = 8.8 Hz, 1H), 7.35 (d, J = 8.0 Hz, 2H), 7.26 (dd, J_1 = 8.8 Hz, J_2 = 2.4 Hz, 1H), 7.20-7.09 (m, 5H), 6.96-6.94 (m, 1H), 6.67-6.66 (m, 1H), 5.52 (dd, J_1 = 12.0 Hz, J_2 = 2.4 Hz, 1H), 4.18 (s, 1H), 4.14-4.08 (m, 1H), 3.97-3.91 (m, 1H), 3.76-3.70 (m, 1H), 3.51-3.45 (m, 1H), 2.55-2.49 (m, 1H), 2.50 (s, 3H), 2.35 (s, 3H), 1.89 (dd, J_1 = 13.2 Hz, J_2 = 2.4 Hz, 1H), 1.24 (td, J_1 = 7.2 Hz, J_2 = 0.8 Hz, 3H), 1.15 (td, J_1 = 7.2 Hz, J_2 = 0.8 Hz, 3H); ^{13}C NMR (100 MHz, $CDCl_3$): 178.28, 139.17 (d, J = 7.1 Hz), 138.19, 136.59, 135.57, 132.67 (d, J = 9.4 Hz), 130.58, 129.25, 128.96, 128.56, 128.06, 127.92, 127.75, 126.97, 126.56, 115.16, 75.59, 75.56, 64.65 (d, J = 5.7 Hz), 64.02 (d, J = 5.5 Hz), 54.41 (d, J = 5.7 Hz), 44.68, 41.30, 21.20, 15.96 (d, J = 7.0 Hz) ,

15.89 (d, J = 7.8 Hz); ^{31}P NMR (122 MHz, CDCl$_3$): -6.82; IR (ATR): 2981, 1745, 1466, 1310, 1248, 1166, 1030 cm^{-1}; MS (EI): 554, 556 (M$^+$, 4, 2), 419 (100), 118 (58), 136 (54), 43 (52), 119 (41), 421 (32), 91 (29); HRMS (EI): Exact mass calcd for $C_{29}H_{32}N_2O_5P^{35}Cl$ [M]$^+$: 554.1737, Found: 554.1735.

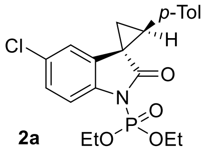

2a

2a was obtained in 45% recovery as a white solid (m.p. 81-84 °C); HPLC analysis (Chiralcel IC, ^{i}PrOH/hexane = 10/90, 1.0 mL/min, 230 nm; t_r (major) = 16.63 min, t_r (minor) = 20.58 min) gave the isomeric composition of the recovered: 97% ee, $[\alpha]^{25}_D$ = -75.9 (c = 1.0, CHCl$_3$); ^{1}H NMR (400 MHz, CDCl$_3$): 7.76 (d, J = 8.8 Hz, 1H), 7.13-7.08 (m, 3H), 7.03 (d, J = 8.0 Hz, 2H), 5.91 (s, 1H), 4.40-4.19 (m, 4H), 3.35 (t, J = 8.8 Hz, 1H), 2.34 (s, 3H), 2.26 (dd, J_1 = 9.2 Hz, J_2 = 4.4 Hz, 1H), 2.01 (dd, J_1 = 8.4 Hz, J_2 = 4.8 Hz, 1H), 1.42-1.36 (m, 6H); ^{13}C NMR (100 MHz, CDCl$_3$): 177.56, 139.45 (d, J = 6.9 Hz), 137.78, 130.45, 129.64, 129.46 (d, J = 9.0 Hz), 129.31, 128.55, 126.81, 120.74, 114.90, 64.76 (d, J= 3.7 Hz), 64.70 (d, J= 3.9 Hz), 38.39, 33.94 (d, J = 8.0 Hz), 24.49, 21.17, 16.11 (d, J= 1.8 Hz), 16.04 (d, J= 2.2 Hz); ^{31}P NMR (122 MHz, CDCl$_3$): -5.88; IR (ATR): 2984, 2925, 1736, 1467, 1287, 1249, 1198, 1149, 1019 cm^{-1}; MS (EI): 419, 421 (M$^+$, 100, 35), 363 (48), 391 (33), 265 (32), 420 (24), 283 (22), 230 (20), 248 (16); HRMS (EI): Exact mass calcd for $C_{21}H_{23}NO_4P^{35}Cl$ [M]$^+$: 419.1053, Found: 419.1058.

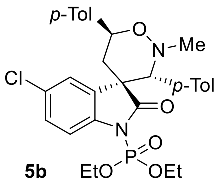

5b

5b was obtained in 45% yield as a white solid (m.p. 62-65 °C); ^{1}H NMR analysis revealed that the dr was above 20:1. HPLC analysis (Chiralcel IC, ^{i}PrOH/hexane = 10/90, 1.0 mL/min, 230 nm; t_r (major) = 12.69 min, t_r (minor) = 16.97 min) gave the isomeric composition of the product: 98% ee, $[\alpha]^{25}_D$ = -102.8 (c = 1.0, CHCl$_3$); ^{1}H NMR (400 MHz, CDCl$_3$): 8.35 (s, 1H), 7.58 (d, J = 8.4 Hz, 1H), 7.34 (d, J = 8.0 Hz, 2H), 7.28-7.25 (m, 1H), 7.18 (d, J = 8.0 Hz, 2H), 7.09 (d, J = 8.8 Hz, 1H), 6.95 (d, J = 7.6 Hz, 1H), 6.75 (d, J = 8.0 Hz, 1H), 6.51 (d, J = 8.0 Hz, 1H), 5.51 (dd, J_1 = 12.0 Hz, J_2 = 2.4 Hz, 1H), 4.14 (s, 1H), 4.13-4.07 (m, 1H), 3.95-3.89 (m, 1H), 3.73-3.67 (m, 1H), 3.45-3.39 (m, 1H), 2.54-2.51 (m, 1H), 2.48 (s, 3H), 2.34 (s, 3H), 2.17 (s, 3H), 1.87 (dd, J_1 = 13.2 Hz, J_2 = 2.4 Hz, 1H), 1.23 (td, J_1 = 7.2 Hz, J_2 = 0.8 Hz, 3H), 1.13 (td, J_1 = 7.2 Hz, J_2 = 0.8 Hz, 3H); ^{13}C NMR (100 MHz, CDCl$_3$): 178.32, 139.14 (d, J = 6.9 Hz), 138.21, 137.77, 136.55, 132.76 (d, J = 9.5 Hz), 132.46, 130.60, 129.26, 128.91, 128.71, 128.51, 128.48, 127.46, 126.97, 126.59, 115.11, 75.58, 75.30, 64.63 (d, J = 5.6 Hz), 63.93 (d, J = 5.5 Hz), 54.39 (d, J = 5.7 Hz), 44.64, 41.26, 21.23, 21.01, 15.97 (d, J = 7.0

Hz) , 15.83 (d, J = 7.9 Hz); ^{31}P NMR (162 MHz, $CDCl_3$): -6.66; IR (ATR): 2980, 2916, 2860, 1742, 1513, 1463, 1281, 1164, 1020 cm^{-1}; MS (EI): 568, 570 (M^+, 6, 3), 419 (100), 150 (73), 421 (39), 420 (25), 132 (18), 391 (15), 91 (15); HRMS (EI): Exact mass calcd for $C_{30}H_{34}N_2O_5P^{35}Cl$ $[M]^+$: 568.1894, Found: 568.1889. In this case, **2a** was recovered in 48% yield and 97% ee.

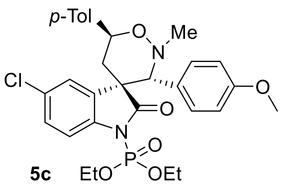

5c

5c was obtained in 37% yield as a white solid (m.p. 133-135 °C); ^{1}H NMR analysis revealed that the dr was above 20:1. HPLC analysis (Chiralcel IC, ^{i}PrOH/hexane = 10/90, 1.0 mL/min, 230 nm; t_r (major) = 12.59 min, t_r (minor) = 16.93 min) gave the isomeric composition of the product: 96% ee, $[\alpha]^{25}_D$ = -96.7 (c = 1.0, $CHCl_3$); ^{1}H NMR (400 MHz, $CDCl_3$): 8.35 (s, 1H), 7.59 (d, J = 8.8 Hz, 1H), 7.34 (d, J = 8.0 Hz, 2H), 7.27 (dd, J_1 = 8.8, J_2 = 2.0, 1H), 7.18 (d, J = 8.0 Hz, 2H), 7.13 (d, J = 8.4 Hz, 1H), 6.66 (d, J = 9.2 Hz, 1H), 6.54 (d, J = 8.4 Hz, 1H), 6.48 (d, J = 8.8 Hz, 1H), 5.51 (dd, J_1 = 12.0 Hz, J_2 = 2.0 Hz, 1H), 4.14-4.08 (m, 1H), 4.12 (s, 1H), 3.96-3.90 (m, 1H), 3.78-3.71 (m, 1H), 3.66 (s, 3H), 3.54-3.48 (m, 1H), 2.54-2.47 (m, 1H), 2.50 (s, 3H), 2.34 (s, 3H), 1.88 (dd, J_1 = 13.2 Hz, J_2 = 2.4 Hz, 1H), 1.23 (td, J_1 = 7.2 Hz, J_2 = 0.8 Hz, 3H), 1.15 (td, J_1 = 7.2 Hz, J_2 = 0.8 Hz, 3H); ^{13}C NMR (100 MHz, $CDCl_3$): 178.35, 159.25, 139.21 (d, J = 7.0 Hz), 138.13, 136.62, 132.83 (d, J = 9.4 Hz), 131.85, 129.22, 128.92, 128.64, 128.53, 127.49, 126.92, 126.54, 115.16, 113.46, 113.01, 75.57, 74.99, 64.62 (d, J = 5.7 Hz), 64.01 (d, J = 5.5 Hz), 55.02, 54.47 (d, J = 5.7 Hz), 44.56, 41.30, 21.17, 15.92 (d, J = 6.9 Hz), 15.82 (d, J = 7.7 Hz); ^{31}P NMR (162 MHz, $CDCl_3$): -6.92; IR (ATR): 3560, 1737, 1609, 1515, 1465, 1425, 1183, 1167, 1031 cm^{-1}; MS (EI): 584 (M^+, 1), 43 (100), 135 (73), 136 (47), 77 (22), 61 (17), 45 (15), 70 (12); HRMS (EI): Exact mass calcd for $C_{30}H_{34}N_2O_6P^{35}Cl$ $[M]^+$: 584.1843, Found: 584.1838. In this case, **2a** was recovered in 42% yield and 99% ee.

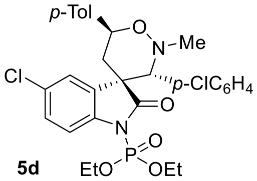

5d

5d was obtained in 41% yield as a white solid (m.p. 125-127 °C); ^{1}H NMR analysis revealed that the dr was above 20:1. HPLC analysis (Chiralcel IC, ^{i}PrOH/hexane = 10/90, 1.0 mL/min, 230 nm; t_r (major) = 9.39 min, t_r (minor) = 11.73 min) gave the isomeric composition of the product: 97% ee, $[\alpha]^{25}_D$ = -101.0 (c = 1.0, $CHCl_3$); ^{1}H NMR (400 MHz, $CDCl_3$): 8.32 (s, 1H), 7.58 (d, J = 8.4 Hz, 1H), 7.33 (d, J = 8.0 Hz, 2H), 7.28 (dd, J_1 = 8.4 Hz, J_2 = 2.0 Hz, 1H), 7.20-7.15 (m, 4H), 6.94 (d, J = 8.8 Hz, 1H), 6.61 (d, J = 8.4 Hz, 1H), 5.50 (dd, J_1 = 12.4 Hz, J_2 = 2.4 Hz, 1H), 4.16 (s, 1H), 4.15-4.09 (m, 1H), 3.97-3.91 (m, 1H),

3.84-3.78 (m, 1H), 3.59-3.53 (m, 1H), 2.54-2.47 (m, 1H), 2.49 (s, 3H), 2.34 (s, 3H), 1.89 (dd, J_1 = 13.2 Hz, J_2 = 2.4 Hz, 1H), 1.25 (td, J_1 = 7.2 Hz, J_2 = 0.8 Hz, 3H), 1.18 (td, J_1 = 7.2 Hz, J_2 = 0.8 Hz, 3H); ^{13}C NMR (100 MHz, $CDCl_3$): 178.07, 139.12 (d, J = 6.9 Hz), 138.30, 136.40, 134.09, 134.07, 132.26 (d, J = 9.3 Hz), 131.91, 129.28, 129.10, 128.99, 128.80, 128.14, 126.90, 126.56, 115.29, 75.59, 74.89, 64.74 (d, J = 5.7 Hz), 64.18 (d, J = 5.8 Hz), 54.40 (d, J = 5.8 Hz), 44.66, 41.09, 21.21, 15.95 (d, J = 6.9 Hz), 15.85 (d, J = 7.4 Hz); ^{31}P NMR (162 MHz, $CDCl_3$): -6.70; IR (ATR): 2919, 1744, 1468, 1288, 1166, 1028 cm^{-1}; MS (EI): 588 (M^+, 1), 419 (100), 421 (35), 170 (30), 152 (30), 420 (23), 153 (16), 391 (14); HRMS (EI): Exact mass calcd for $C_{29}H_{31}N_2O_5P^{35}Cl_2$ $[M]^+$: 588.1348, Found: 588.1349. In this case, **2a** was recovered in 41% yield and 97% ee.

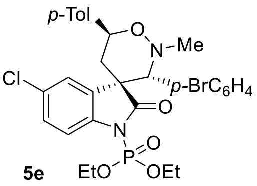

5e was obtained in 40% yield as a white solid (m.p. 78-81 °C); ^{1}H NMR analysis revealed that the dr was above 20:1. HPLC analysis (Chiralcel IC, ^{i}PrOH/hexane = 10/90, 1.0 mL/min, 230 nm; t_r (major) = 10.31 min, t_r (minor) = 13.40 min) gave the isomeric composition of the product: 97% ee, $[\alpha]^{25}_D$ = -100.8 (c = 1.0, $CHCl_3$); ^{1}H NMR (400 MHz, $CDCl_3$): 8.32 (s, 1H), 7.58 (d, J = 8.8 Hz, 1H), 7.34-7.27 (m, 4H), 7.19 (d, J = 8.0 Hz, 2H), 7.11-7.08 (m, 2H), 6.55 (d, J = 7.6 Hz, 1H), 5.49 (dd, J_1 = 12.4 Hz, J_2 = 2.4 Hz, 1H), 4.15 (s, 1H), 4.14-4.08 (m, 1H), 3.96-3.89 (m, 1H), 3.83-3.77 (m, 1H), 3.56-3.50 (m, 1H), 2.54-2.47 (m, 1H), 2.49 (s, 3H), 2.34 (s, 3H), 1.88 (dd, J_1 = 12.8 Hz, J_2 = 2.4 Hz, 1H), 1.25 (td, J_1 = 7.2 Hz, J_2 = 0.8 Hz, 3H), 1.18 (td, J_1 = 7.2 Hz, J_2 = 0.8 Hz, 3H); ^{13}C NMR (100 MHz, $CDCl_3$): 178.03, 139.07 (d, J = 7.0 Hz), 138.32, 136.31, 134.53, 132.22, 132.11, 131.09 (d, J = 5.3 Hz), 129.29, 129.08, 128.82, 126.88, 126.56, 122.26, 115.27, 75.57, 74.90, 64.73 (d, J = 5.5 Hz), 64.15 (d, J = 5.5 Hz), 54.30 (d, J = 5.7 Hz), 44.68, 41.01, 21.22, 15.98 (d, J = 7.2 Hz), 15.89 (d, J = 7.8 Hz); ^{31}P NMR (122 MHz, $CDCl_3$): -6.71; IR (ATR): 2980, 2917, 1741, 1466, 1285, 1164, 1021 cm^{-1}; MS (EI): 632, 634 (M^+, 1, 1), 419 (100), 421 (35), 420 (23), 214 (16), 216 (14), 391 (12), 363 (11); HRMS (EI): Exact mass calcd for $C_{29}H_{31}N_2O_5P^{35}Cl^{79}Br$ $[M]^+$: 632.0842, Found: 632.0837. In this case, **2a** was recovered in 50% yield and 92% ee.

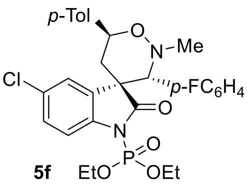

5f was obtained in 45% yield as a white solid (m.p. 62-64 °C); ^{1}H NMR analysis revealed that the dr was above 20:1. HPLC analysis (Chiralcel IC, ^{i}PrOH/hexane = 10/90, 1.0 mL/min, 230 nm; t_r (major) = 11.10 min, t_r (minor)

= 14.33 min) gave the isomeric composition of the product: 95% ee, $[\alpha]^{25}_{D}$ = -59.3 (c = 1.0, $CHCl_3$); ^{1}H NMR (400 MHz, $CDCl_3$): 8.32 (s, 1H), 7.58 (d, J = 8.4 Hz, 1H), 7.34 (d, J = 8.4 Hz, 2H), 7.28 (dd, J_1 = 8.8 Hz, J_2 = 2.4 Hz, 1H), 7.20-7.18 (m, 3H), 6.87-6.82 (m, 1H), 6.68-6.65 (m, 2H), 5.50 (dd, J_1 = 12.0 Hz, J_2 = 2.4 Hz, 1H), 4.17 (s, 1H), 4.15-4.09 (m, 1H), 3.98-3.92 (m, 1H), 3.86-3.80 (m, 1H), 3.61-3.55 (m, 1H), 2.54-2.51 (m, 1H), 2.48 (s, 3H), 2.34 (s, 3H), 1.89 (dd, J_1 = 13.2 Hz, J_2 = 2.4 Hz, 1H), 1.24 (td, J_1 = 7.2 Hz, J_2 = 0.8 Hz, 3H), 1.18 (td, J_1 = 7.2 Hz, J_2 = 0.8 Hz, 3H); ^{13}C NMR (100 MHz, $CDCl_3$): 178.20, 162.29 (d, J = 246.5 Hz), 139.10 (d, J = 7.0 Hz), 138.30, 136.40, 132.34 (d, J = 9.5 Hz), 132.19 (d, J = 7.9 Hz), 131.34 (d, J = 3.3 Hz), 129.38, 129.29, 129.05, 128.74, 126.88, 126.58, 115.26, 115.01, 114.76, 114.54, 75.59, 74.70, 64.70 (d, J = 5.6 Hz), 64.16 (d, J = 5.6 Hz), 54.42 (d, J = 5.6 Hz), 44.64, 41.11, 21.23, 15.96 (d, J = 6.8 Hz), 15.87 (d, J = 7.6 Hz); ^{31}P NMR (122 MHz, $CDCl_3$): -6.65; ^{19}F NMR (282 MHz, $CDCl_3$): -113.25; IR (ATR): 2980, 2918, 1742, 1604, 1509, 1466, 1281, 1224, 1163, 1021 cm^{-1}; MS (EI): 572, (M^+, 3), 419 (100), 421 (36), 154 (25), 420 (24), 391 (12), 363 (10), 136 (10); HRMS (EI): Exact mass calcd for $C_{29}H_{31}N_2O_5P^{35}ClF$ $[M]^+$: 572.1643, Found: 572.1639. In this case, **2a** was recovered in 47% yield and 99% ee.

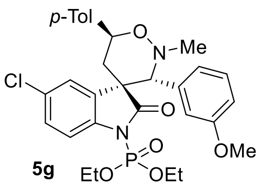

5g

5g was obtained in 46% yield as a white solid (m.p. 146-148 °C); ^{1}H NMR analysis revealed that the dr was above 20:1. HPLC analysis (Chiralcel IE, ^{i}PrOH/hexane = 15/85, 1.0 mL/min, 230 nm; t_r (major) = 12.04 min, t_r (minor) = 16.15 min) gave the isomeric composition of the product: 95% ee, $[\alpha]^{25}_{D}$ = -78.2 (c = 1.0, $CHCl_3$); ^{1}H NMR (400 MHz, $CDCl_3$): 8.38-8.32 (m, 1H), 7.64-7.58 (m, 1H), 7.34 (d, J = 7.6 Hz, 2H), 7.29-7.26 (m, 1H), 7.19 (d, J = 8.0 Hz, 2H), 7.05-7.60 (m, 3H), 6.25-6.20 (m, 1H), 5.51 (dd, J_1 = 12.0 Hz, J_2 = 2.4 Hz, 1H), 4.16-4.09 (m, 2H), 4.00-3.94 (m, 1H), 3.78-3.70 (m, 2H), 3.53-3.41 (m, 3H), 2.55-2.49 (m, 1H), 2.52 (s, 3H), 2.35 (s, 3H), 1.92-1.86 (m, 1H), 1.25 (td, J_1 = 7.2 Hz, J_2 = 0.8 Hz, 3H), 1.17 (td, J_1 = 7.2 Hz, J_2 = 0.8 Hz, 3H); ^{13}C NMR (100 MHz, $CDCl_3$): 178.13, 158.99, 139.42, 139.35, 139.16, 138.13, 136.83, 136.54, 133.01, 132.91, 129.21, 128.89, 128.70, 128.45, 126.89, 126.50, 122.70, 120.07, 116.40, 116.20, 115.39, 115.10, 112.73, 111.21, 75.69, 75.56, 75.14, 64.70, 64.64, 64.09, 64.03, 54.91, 54.31, 54.25, 44.60, 41.29, 41.14, 21.14, 15.96, 15.89, 15.80; ^{31}P NMR (162 MHz, $CDCl_3$): -7.04; IR (ATR): 2915, 1750, 1597, 1260, 1161, 1026 cm^{-1}; MS (EI): 584, 586 (M^+, 6, 3), 419 (100), 148 (54), 166 (52), 421 (39), 149 (33), 91 (25), 44 (24); HRMS (EI): Exact mass calcd for $C_{30}H_{34}N_2O_6P^{35}Cl$ $[M]^+$: 584.1843, Found: 584.1845. In this case, **2a** was

recovered in 49% yield and 90% ee.

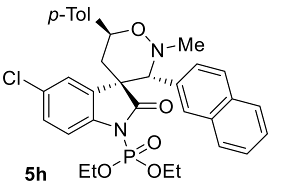

5h was obtained in 42% yield as a white solid (m.p. 136-138 ºC); ^{1}H NMR analysis revealed that the dr was above 20:1. HPLC analysis (Chiralcel IC, ^{i}PrOH/hexane = 10/90, 1.0 mL/min, 230 nm; t_r (major) = 13.11 min, t_r (minor) = 16.09 min) gave the isomeric composition of the product: 97% ee, $[\alpha]^{25}_{D}$ = -193.7 (c = 1.0, $CHCl_3$); ^{1}H NMR (400 MHz, $CDCl_3$): 8.49-8.43 (m, 1H), 7.77-7.32 (m, 9H), 7.28-7.25 (m, 1H), 7.21 (d, *J* = 8.0 Hz, 2H), 7.09-6.78 (m, 1H), 5.59-5.56 (m, 1H), 4.37-4.35 (m, 1H), 3.99-3.93 (m, 1H), 3.78-3.70 (m, 1H), 3.50-3.00 (m, 2H), 2.53 (s, 3H), 2.62-2.55 (m, 1H), 2.36 (s, 3H), 1.98-1.91 (m, 1H), 1.13-1.07 (m, 3H), 0.96-0.67 (m, 3H); ^{13}C NMR (100 MHz, $CDCl_3$): 178.20, 139.08, 139.01, 138.25, 136.55, 133.14, 132.83, 132.69, 129.58, 129.29, 129.02, 128.64, 128.17, 127.78, 127.51, 127.40, 127.10, 126.60, 126.43, 126.24, 124.79, 115.12, 75.82, 75.71, 75.54, 64.55, 64.50, 63.81, 63.76, 54.68, 54.62, 44.91, 44.73, 41.19, 21.23, 15.84, 15.77, 15.40, 15.33; ^{31}P NMR (122 MHz, $CDCl_3$): -6.87; IR (ATR): 2980, 2915, 1742, 1465, 1285, 1164, 1020 cm^{-1}; MS (EI): 604, 606 (M^+, 7, 2), 419 (100), 186 (92), 168 (42), 421 (36), 420 (23), 185 (15), 169 (14); HRMS (EI): Exact mass calcd for $C_{33}H_{34}N_2O_5P^{35}Cl$ $[M]^+$: 604.1894, Found: 604.1898. In this case, **2a** was recovered in 48% yield and 98% ee.

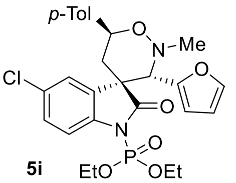

5i was obtained in 48% yield as a white solid (m.p. 146-148 ºC); ^{1}H NMR analysis revealed that the dr was above 20:1. HPLC analysis (Chiralcel IC, ^{i}PrOH/hexane = 10/90, 1.0 mL/min, 230 nm; t_r (major) = 9.23 min, t_r (minor) = 15.06 min) gave the isomeric composition of the product: 98% ee, $[\alpha]^{25}_{D}$ = -53.5 (c = 1.0, $CHCl_3$); ^{1}H NMR (400 MHz, $CDCl_3$): 8.28 (s, 1H), 7.80 (d, *J* = 8.8 Hz, 1H), 7.36-7.30 (m, 3H), 7.18-7.16 (m, 3H), 6.08 (dd, J_1 = 3.2 Hz, J_2 = 1.6 Hz, 1H), 5.47 (dd, J_1 = 3.2 Hz, J_2 = 0.8 Hz, 1H), 5.43 (dd, J_1 = 12.0 Hz, J_2 = 2.4 Hz, 1H), 4.30 (s, 1H), 4.22-4.07 (m, 2H), 3.88-3.81 (m, 1H), 3.63-3.57 (m, 1H), 2.48-2.41 (m, 1H), 2.45 (s, 3H), 2.34 (s, 3H), 1.88 (dd, J_1 = 13.2 Hz, J_2 = 2.4 Hz, 1H), 1.28 (td, J_1 = 7.2 Hz, J_2 = 0.8 Hz, 3H), 1.15 (td, J_1 = 7.2 Hz, J_2 = 1.2 Hz, 3H); ^{13}C NMR (100 MHz, $CDCl_3$): 178.11, 148.96, 142.32, 139.38 (d, *J* = 7.0 Hz), 138.23, 136.33, 133.19 (d, *J* = 9.4 Hz), 129.24, 129.09, 128.73, 126.87, 126.53, 115.28, 110.34, 109.44, 75.82, 68.56, 64.92 (d, *J* = 5.8 Hz), 64.16 (d, *J* = 5.6 Hz), 52.56 (d, *J* = 5.8 Hz), 44.23, 41.14, 21.19, 16.01 (d, *J* = 6.7 Hz), 15.80 (d, *J* =

7.5 Hz); ^{31}P NMR (122 MHz, $CDCl_3$): -6.70; IR (ATR): 2972, 2918, 2862, 1742, 1470, 1271, 1164, 1111, 1017 cm^{-1}; MS (EI): 544, 546 (M^+, 7, 3), 419 (100), 421 (33), 126 (32), 420 (22), 391 (16), 363 (13), 125 (10); HRMS (EI): Exact mass calcd for $C_{27}H_{30}N_2O_6P^{35}Cl$ $[M]^+$: 544.1530, Found: 544.1535. In this case, **2a** was recovered in 44% yield and 99% ee.

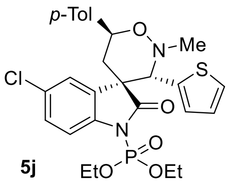

5j

5j was obtained in 42% yield as a white solid (m.p. 68-69 °C); ^{1}H NMR analysis revealed that the dr was above 20:1. HPLC analysis (Chiralcel IC, ^{i}PrOH/hexane = 10/90, 1.0 mL/min, 230 nm; t_r (major) = 10.48 min, t_r (minor) = 15.09 min) gave the isomeric composition of the product: 97% ee, $[\alpha]^{25}_D$ = -53.4 (c = 1.0, $CHCl_3$); ^{1}H NMR (400 MHz, $CDCl_3$): 8.34 (s, 1H), 7.74 (d, J = 8.8 Hz, 1H), 7.36 (dd, J_1 = 8.8 Hz, J_2 = 2.4 Hz, 1H), 7.33 (d, J = 8.0 Hz , 2H), 7.18 (d, J = 7.6 Hz , 2H), 7.06 (d, J = 5.2 Hz, 1H), 6.84 (s, br, 1H), 6.77-6.75 (m, 1H), 5.48 (dd, J_1 = 12.0 Hz, J_2 = 2.4 Hz, 1H), 4.48 (s, 1H), 4.20-4.13 (m, 1H), 4.05-3.99 (m, 1H), 3.70-3.64 (m, 1H), 3.35-3.29 (m, 1H), 2.53-2.46 (m, 1H), 2.44 (s, 3H), 2.34 (s, 3H), 1.92 (dd, J_1 = 13.2 Hz, J_2 = 2.4 Hz, 1H), 1.26 (td, J_1 = 7.2 Hz, J_2 = 0.8 Hz, 3H), 1.09 (td, J_1 = 7.2 Hz, J_2 = 0.8 Hz, 3H); ^{13}C NMR (100 MHz, $CDCl_3$): 178.06, 139.94 (d, J = 7.0 Hz), 138.29, 136.46, 136.31, 132.59 (d, J = 9.4 Hz), 129.28, 129.22, 129.10, 128.75, 127.34, 127.19, 126.56, 125.51, 115.36, 75.66, 71.41, 64.84 (d, J = 5.8 Hz), 64.81 (d, J = 5.5 Hz), 53.89 (d, J = 5.8 Hz), 44.40, 41.06, 21.23, 16.02 (d, J = 6.9 Hz), 15.85 (d, J = 7.8 Hz); ^{31}P NMR (122 MHz, $CDCl_3$): -6.70; IR (ATR): 2981, 2914, 1743, 1464, 1286, 1164, 1020 cm^{-1}; MS (EI): 560, 562 (M^+, 4, 2), 419 (100), 421 (35), 142 (35), 420 (24), 391 (15), 363 (14), 141 (8); HRMS (EI): Exact mass calcd for $C_{27}H_{30}N_2O_5PS^{35}Cl$ $[M]^+$: 560.1302, Found: 560.1299. In this case, **2a** was recovered in 48% yield and 90% ee.

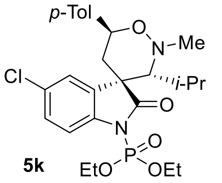

5k

5k was obtained in 42% yield as a colorless oil; ^{1}H NMR analysis revealed that the dr was above 20:1. HPLC analysis (Chiralcel IC, ^{i}PrOH/hexane = 10/90, 1.0 mL/min, 230 nm; t_r (major) = 6.43 min, t_r (minor) = 7.84 min) gave the isomeric composition of the product: 93% ee, $[\alpha]^{25}_D$ = -56.3 (c = 1.0, $CHCl_3$); ^{1}H NMR (400 MHz, $CDCl_3$): 8.14 (s, 1H), 7.87 (d, J = 8.4 Hz, 1H), 7.31 (dd, J_1 = 8.8 Hz, J_2 = 2.4 Hz, 1H), 7.24 (d, J = 8.0 Hz, 2H), 7.14 (d, J = 8.0 Hz, 2H), 5.24 (dd, J_1 = 12.0 Hz, J_2 = 2.4 Hz, 1H), 4.37-4.10 (m, 4H), 3.16 (d, J = 3.2 Hz, 1H), 2.76 (s, 3H), 2.34-2.28 (m, 1H), 2.32 (s, 3H), 1.82-1.74 (m, 1H), 1.60 (dd, J_1 = 13.2

Hz, J_2 = 2.4 Hz, 1H), 1.36 (td, J_1 = 7.2 Hz, J_2 = 0.8 Hz, 3H), 1.32 (td, J_1 = 7.2 Hz, J_2 = 0.8 Hz, 3H), 0.70 (dd, J_1 = 13.2 Hz, J_2 = 6.8 Hz, 6H); ^{13}C NMR (100 MHz, $CDCl_3$): 179.80, 138.67 (d, J = 7.2 Hz), 138.03, 136.65, 134.06 (d, J = 9.6 Hz), 129.18, 128.97, 128.35, 127.49, 126.51, 115.52, 75.15, 73.22, 65.08 (d, J = 6.1 Hz), 64.69 (d, J = 5.9 Hz), 52.35 (d, J = 5.5 Hz), 45.09, 44.22, 29.93, 21.35, 21.16, 19.28, 16.10 (d, J = 5.4 Hz), 16.04 (d, J = 5.1 Hz); ^{31}P NMR (122 MHz, $CDCl_3$): -6.51; IR (ATR): 2984, 1736, 1467, 1287, 1199, 1149, 1018 cm^{-1}; MS (EI): 520 (M^+, 1), 419 (100), 363 (38), 421 (36), 391 (31), 283 (30), 265 (26), 102 (24); HRMS (EI): Exact mass calcd for $C_{26}H_{34}N_2O_5P^{35}Cl$ $[M]^+$: 520.1894, Found: 520.1899. In this case, **2a** was recovered in 40% yield and 77% ee.

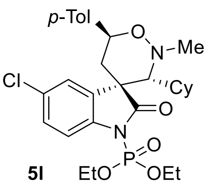

5l

5l was obtained in 36% yield as a white solid (m.p. 60-61 ºC); ^{1}H NMR analysis revealed that the dr was 13:1. HPLC analysis (Chiralcel IC, ^{i}PrOH/hexane = 15/85, 1.0 mL/min, 230 nm; t_r (major) = 5.02 min, t_r (minor) = 6.21 min) gave the isomeric composition of the product: 90% ee, $[\alpha]^{25}_D$ = -0.6 (c = 1.0, $CHCl_3$); ^{1}H NMR (400 MHz, $CDCl_3$): 8.13 (s, 1H), 7.88 (d, J = 8.4 Hz, 1H), 7.32 (dd, J_1 = 8.8 Hz, J_2 = 2.4 Hz, 1H), 7.24 (d, J = 8.0 Hz, 2H), 7.13 (d, J = 8.0 Hz, 2H), 5.23 (dd, J_1 = 12.0 Hz, J_2 = 2.0 Hz, 1H), 4.36-4.11 (m, 4H), 3.16 (d, J = 3.2 Hz, 1H), 2.77 (s, 3H), 2.33-2.26 (m, 1H), 2.31 (s, 3H), 1.55-1.51 (m, 7H), 1.38 (td, J_1 = 6.8 Hz, J_2 = 0.8 Hz, 3H), 1.31 (td, J_1 = 7.2 Hz, J_2 = 0.8 Hz, 3H), 1.01-0.81 (m, 4H), 0.47-0.43 (m, 1H); ^{13}C NMR (100 MHz, $CDCl_3$): 179.65, 138.42 (d, J = 7.2 Hz), 138.01, 136.57, 134.40 (d, J = 9.7 Hz), 129.15, 128.88, 128.31, 127.38, 126.50, 115.41, 75.10, 73.44, 64.95 (d, J = 6.0 Hz), 64.63 (d, J = 6.0 Hz), 51.86 (d, J = 5.6 Hz), 44.89, 44.55, 41.86, 31.06, 30.57, 27.41, 27.36, 26.39, 21.16, 16.13 (d, J = 7.1 Hz), 16.04 (d, J = 6.7 Hz); ^{31}P NMR (122 MHz, $CDCl_3$): -6.02; IR (ATR): 2923, 1748, 1466, 1025 cm^{-1}; MS (EI): 560, 562 (M^+, 5, 2), 419 (100), 142 (44), 421 (32), 420 (23), 44 (22), 42 (18), 91 (17); HRMS (EI): Exact mass calcd for $C_{29}H_{38}N_2O_5P^{35}Cl$ $[M]^+$: 560.2207, Found: 560.2210. In this case, **2a** was recovered in 45% yield and 79% ee.

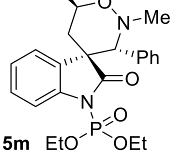

5m

5m was obtained in 31% yield as a white solid (m.p. 155-160 ºC); ^{1}H NMR analysis revealed that the dr was above 20:1. HPLC analysis (Chiralcel IC, ^{i}PrOH/hexane = 10/90, 1.0 mL/min, 230 nm; t_r (major) = 14.11 min, t_r (minor) = 16.94 min) gave the isomeric composition of the product: 95% ee, $[\alpha]^{25}_D$ = -56.7 (c = 1.0, $CHCl_3$); ^{1}H NMR (400 MHz, $CDCl_3$): 8.36 (d, J = 7.2 Hz, 1H), 7.61 (dd, J_1 = 7.6 Hz, J_2 = 1.6 Hz, 1H), 7.34-7.27

(m, 3H), 7.25-7.04 (m, 6H), 6.87 (s, br, 1H), 6.64 (s, br, 1H), 5.59 (dd, J_1 = 12.0 Hz, J_2 = 2.4 Hz, 1H), 4.18 (s, 1H), 4.12-4.06 (m, 1H), 3.93-3.87 (m, 1H), 3.75-3.69 (m, 1H), 3.51-3.45 (m, 1H), 2.56-2.50 (m, 1H), 2.49 (s, 3H), 2.34 (s, 3H), 1.90 (dd, J_1 = 12.8 Hz, J_2 = 2.4 Hz, 1H), 1.23 (td, J_1 = 7.6 Hz, J_2 = 0.8 Hz, 3H), 1.15 (td, J_1 = 7.2 Hz, J_2 = 1.2 Hz, 3H); ^{13}C NMR (100 MHz, $CDCl_3$): 178.82, 140.57 (d, J = 6.7 Hz), 138.06, 136.89, 135.88, 130.91 (d, J = 9.2 Hz), 130.65, 129.22, 129.18, 128.58, 127.89, 127.78, 127.69, 126.98, 126.56, 123.42, 113.99, 75.83, 75,76, 64.40 (d, J = 5.6 Hz), 63.84 (d, J = 5.5 Hz), 54.20 (d, J = 5.9 Hz), 44.76, 41.54, 21.19, 15.97 (d, J = 6.8 Hz), 15.89 (d, J = 7.6 Hz); ^{31}P NMR (122 MHz, $CDCl_3$): -6.60; IR (ATR): 2989, 2944, 2916, 1736, 1478, 1280, 1167, 1036 cm^{-1}; MS (EI): 520 (M$^+$, 3), 385 (100), 118 (30), 136 (26), 386 (23), 119 (19), 249 (18), 91 (16); HRMS (EI): Exact mass calcd for $C_{29}H_{33}N_2O_5P$ [M]$^+$: 520.2127, Found: 520.2130.

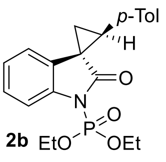

2b

2b was obtained in 39% recovery as a white solid (m.p. 115-116 ºC); HPLC analysis (Chiralcel IC, ^{i}PrOH/hexane = 20/80, 1.0 mL/min, 230 nm; t_r (major) = 12.26 min, t_r (minor) = 15.94 min) gave the isomeric composition of the recovered: 90% ee, $[\alpha]^{25}_D$ = -145.0 (c = 1.0, $CHCl_3$); ^{1}H NMR (400 MHz, $CDCl_3$): 7.82 (d, J = 8.0 Hz, 1H), 7.16-7.12 (m, 1H), 7.10 (d, J = 8.0 Hz, 2H), 7.04 (d, J = 8.0 Hz, 2H), 6.76 (td, J_1 = 7.6 Hz, J_2 = 1.2 Hz, 1H), 5.96 (d, J = 7.6 Hz, 1H), 4.37-4.24 (m, 4H), 3.32 (t, J = 8.4 Hz, 1H), 2.32 (s, 3H), 2.24 (dd, J_1 = 9.2 Hz, J_2 = 4.4 Hz, 1H), 2.00 (dd, J_1 = 8.4 Hz, J_2 = 4.4 Hz, 1H), 1.43-1.37 (m, 6H); ^{13}C NMR (100 MHz, $CDCl_3$): 178.18, 140.93 (d, J = 6.5 Hz), 137.38, 131.08, 129.79, 129.17, 127.54 (d, J = 8.8 Hz), 126.89, 123.02, 120.58, 113.82, 64.58 (d, J= 5.4 Hz), 64.53 (d, J= 5.4 Hz), 37.83, 33.88 (d, J = 8.2 Hz), 24.25, 21.22, 16.16 (d, J = 2.0 Hz), 16.09 (d, J = 2.2 Hz); ^{31}P NMR (122 MHz, $CDCl_3$): -5.45; IR (ATR): 2982, 2924, 1733, 1609, 1463, 1304, 1147, 1019 cm^{-1}; MS (GC-MS): 385 (M$^+$, 100), 231 (91), 329 (61), 357 (48), 249 (39), 204 (13), 220 (10), 281 (3); HRMS (EI): Exact mass calcd for $C_{21}H_{24}NO_4P$ [M]$^+$: 385.1443, Found: 385.1448.

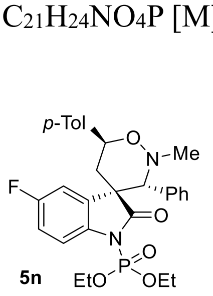

5n

5n was obtained in 40% yield as a white solid (m.p. 150-153 ºC); ^{1}H NMR analysis revealed that the dr was above 20:1. HPLC analysis (Chiralcel IC, ^{i}PrOH/hexane = 15/85, 1.0 mL/min, 230 nm; t_r (major) = 11.83 min, t_r (minor) = 15.53 min) gave the isomeric composition of the product: 98% ee, $[\alpha]^{25}_D$= -68.0 (c = 1.0, $CHCl_3$); ^{1}H NMR (400 MHz, $CDCl_3$): 8.12 (m, 1H), 7.60 (dd, J_1 = 9.2 Hz, J_2 = 4.8 Hz, 1H), 7.34 (d, J = 8.0

Hz, 2H), 7.23-7.07 (m, 5H), 7.02-6.93 (m, 2H), 6.70-6.68 (m, 1H), 5.52 (dd, J_1 = 12.0 Hz, J_2 = 2.4 Hz, 1H), 4.19 (s, 1H), 4.14-4.08 (m, 1H), 3.97-3.91 (m, 1H), 3.76-3.70 (m, 1H), 3.51-3.45 (m, 1H), 2.56-2.50 (m, 1H), 2.50 (s, 3H), 2.34 (s, 3H), 1.89 (dd, J_1 = 12.8 Hz, J_2 = 2.0 Hz, 1H), 1.24 (td, J_1 = 7.6 Hz, J_2 = 1.2 Hz, 3H), 1.15 (td, J_1 = 6.8 Hz, J_2 = 0.8 Hz, 3H); ^{13}C NMR (100 MHz, $CDCl_3$): 178.54, 159.20 (d, J = 240.2 Hz), 138.16, 136.61, 136.50 (dd, J_1 = 7.0 Hz, J_2 = 2.3 Hz), 135.62, 132.57 (d, J = 9.1 Hz), 132.48 (d, J = 8.9 Hz), 130.60, 129.24, 128.02, 127.87, 127.68, 126.54, 115.07 (d, J = 8.9 Hz), 114.91 (d, J = 6.1 Hz), 114.54 (d, J = 25.0 Hz), 75.62, 75.54, 64.56 (d, J = 5.7 Hz), 63.93 (d, J = 5.6 Hz), 54.53 (dd, J_1 = 5.9 Hz, J_2 = 1.8 Hz), 44.64, 41.38, 21.18, 15.95 (d, J = 7.0 Hz), 15.87 (d, J = 7.8 Hz); ^{31}P NMR (122 MHz, $CDCl_3$): -6.48; ^{19}F NMR (282 MHz, $CDCl_3$): -118.67; IR (ATR): 2980, 2919, 1738, 1475, 1294, 1259, 1164, 1098, 1024 cm^{-1}; MS (EI): 538 (M^+, 3), 403 (100), 136 (37), 118 (24), 404 (23), 375 (15), 347 (14), 249 (14); HRMS (EI): Exact mass calcd for $C_{29}H_{32}N_2O_5PF$ $[M]^+$: 538.2033, Found: 538.2029.

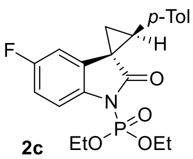

2c

2c was obtained in 50% recovery as a white solid (m.p. 109-110 °C); HPLC analysis (Chiralcel IC, ^{i}PrOH/hexane = 15/85, 1.0 mL/min, 230 nm; t_r (major) = 14.73 min, t_r (minor) = 18.71 min) gave the isomeric composition of the recovered: 92% ee, $[\alpha]^{25}_D$ = -150.2 (c = 1.0, $CHCl_3$); ^{1}H NMR (400 MHz, $CDCl_3$): 7.77 (dd, J_1 = 8.8 Hz, J_2 = 4.4 Hz, 1H), 7.11 (d, J = 8.0 Hz, 2H), 7.03 (d, J = 8.0 Hz, 2H), 6.82 (td, J_1 = 9.2 Hz, J_2 = 2.8 Hz, 1H), 5.68 (ddd, J_1 = 8.4 Hz, J_2 = 2.8 Hz, J_3 = 1.2 Hz, 1H), 4.36-4.24 (m, 4H), 3.35 (t, J = 8.4 Hz, 1H), 2.33 (s, 3H), 2.26 (dd, J_1 = 9.2 Hz, J_2 = 4.4 Hz, 1H), 2.00 (dd, J_1 = 8.0 Hz, J_2 = 4.4 Hz, 1H), 1.43-1.36 (m, 6H); ^{13}C NMR (100 MHz, $CDCl_3$): 177.90, 159.01 (d, J = 239.5 Hz), 137.75, 136.74 (dd, J_1 = 6.9 Hz, J_2 = 2.2 Hz), 130.50, 129.64, 129.50 (t, J = 9.2 Hz), 129.36, 114.73 (d, J = 8.1 Hz), 113.27 (d, J = 23.1 Hz), 108.16 (d, J = 25.6 Hz), 64.70 (d, J = 4.5 Hz), 64.64 (d, J = 4.4 Hz), 38.24, 34.18 (dd, J_1 = 8.1 Hz, J_2 = 2.2 Hz), 24.56, 21.21, 16.14 (d, J = 2.0 Hz), 16.07 (d, J = 2.3 Hz); ^{31}P NMR (122 MHz, $CDCl_3$): -5.54; ^{19}F NMR (282 MHz, $CDCl_3$): -119.45; IR (ATR): 2984, 2928, 1735, 1603, 1473, 1373, 1285, 1136, 1019 cm^{-1}; MS (GC-MS): 403 (M^+, 100), 249 (94), 347 (75), 375 (53), 267 (52), 222 (19), 235 (13), 207 (12); HRMS (EI): Exact mass calcd for $C_{21}H_{23}NO_4PF$ $[M]^+$: 403.1349, Found: 403.1353.

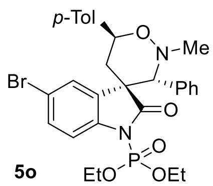

5o was obtained in 44% yield as a white solid (m.p. 129-132 °C); ^{1}H NMR analysis revealed that the dr was above 20:1. PLC analysis (Chiralcel IC, ^{i}PrOH/hexane = 10/90, 1.0 mL/min, 230 nm; t_r (major) = 9.61 min, t_r (minor) = 12.71 min) gave the isomeric composition of the product: 96% ee, $[\alpha]^{25}_{D}$ = -92.1 (c = 1.0, $CHCl_3$); ^{1}H NMR (400 MHz, $CDCl_3$): 8.49 (s, 1H), 7.53 (d, J = 8.8 Hz, 1H), 7.41(dd, J_1 = 8.8 Hz, J_2 = 2.4 Hz, 1H), 7.35 (d, J = 8.0 Hz, 2H), 7.20-7.07 (m, 5H), 6.97-6.95 (m, 1H), 6.67-6.65 (m, 1H), 5.51 (dd, J_1 = 12.0 Hz, J_2 = 2.4 Hz, 1H), 4.17 (s, 1H), 4.12-4.07 (m, 1H), 3.97-3.92 (m, 1H), 3.76-3.70 (m, 1H), 3.51-3.47 (m, 1H), 2.55-2.49 (m, 1H), 2.50 (s, 3H), 2.35 (s, 3H), 1.89 (dd, J_1 = 13.2 Hz, J_2 = 2.8 Hz, 1H), 1.23 (td, J_1 = 6.8 Hz, J_2 = 1.2 Hz, 3H), 1.15 (td, J_1 = 7.6 Hz, J_2 = 0.8 Hz, 3H); ^{13}C NMR (100 MHz, $CDCl_3$): 178.16, 139.69 (d, J = 7.0 Hz), 138.20, 136.58, 135.56, 133.09 (d, J = 9.4 Hz), 131.51, 130.58, 129.74, 129.26, 128.07, 127.93, 127.76, 126.57, 116.53, 115.62, 75.58, 64.67 (d, J = 5.7 Hz), 64.05 (d, J = 5.6 Hz), 54.38 (d, J = 5.8 Hz), 44.70, 41.29, 21.20, 15.96 (d, J = 6.9 Hz), 15.89 (d, J = 7.6 Hz); ^{31}P NMR (162 MHz, $CDCl_3$): -6.71; IR (ATR): 2975, 1740, 1466, 1291, 1189, 1166, 1032 cm^{-1}; MS (EI): 598, 600 (M^+, 3, 3), 463 (100), 465 (96), 136 (90), 118 (76), 119 (48), 91 (40), 77 (26); HRMS (EI): Exact mass calcd for $C_{29}H_{32}N_2O_5P^{79}Br$ $[M]^+$: 598.1232, Found: 598.1240.

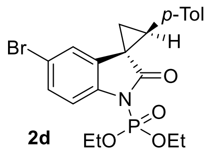

2d was obtained in 49% recovery as a white solid (m.p. 64-66 °C); HPLC analysis (Chiralcel IC, ^{i}PrOH/hexane = 10/90, 1.0 mL/min, 230 nm; t_r (major) = 15.43 min, t_r (minor) = 18.55 min) gave the isomeric composition of the recovered: 99% ee, $[\alpha]^{25}_{D}$ = -39.3 (c = 1.0, $CHCl_3$); ^{1}H NMR (400 MHz, $CDCl_3$): 7.71 (d, J = 8.4 Hz, 1H), 7.24 (dd, J_1 = 8.4 Hz, J_2 = 2.0 Hz, 1H), 7.13 (d, J = 8.0 Hz, 2H), 7.03 (d, J = 8.0 Hz, 2H), 6.03 (s, 1H), 4.35-4.23 (m, 4H), 3.35 (t, J = 8.8 Hz, 1H), 2.34 (s, 3H), 2.26 (dd, J_1 = 9.2 Hz, J_2 = 4.4 Hz, 1H), 2.02 (dd, J_1 = 8.4 Hz, J_2 = 4.8 Hz, 1H), 1.42-1.36 (m, 6H); ^{13}C NMR (100 MHz, $CDCl_3$): 177.46, 139.95 (d, J = 6.9 Hz), 137.82, 130.44, 129.82 (d, J = 9.0 Hz), 129.71, 129.67, 129.31, 123.60, 116.09, 115.36, 64.79 (d, J = 4.2 Hz), 64.74 (d, J = 4.2 Hz), 38.45, 33.86 (d, J = 8.0 Hz), 24.55, 21.20, 16.14 (d, J = 1.9 Hz), 16.07 (d, J = 2.1 Hz); ^{31}P NMR (122 MHz, $CDCl_3$): -5.78; IR (ATR): 2982, 1736, 1463, 1019 cm^{-1}; MS (EI): 463, 465 (M^+, 32, 32), 84 (100), 86 (70), 465 (32), 463 (32), 44 (24), 47 (22), 49 (19); HRMS (EI): Exact mass calcd for $C_{21}H_{23}NO_4P^{79}Br$ $[M]^+$: 463.0548, Found: 463.0552.

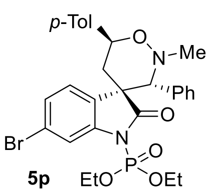

5p

5p was obtained in 38% yield as a white solid (m.p. 180-186 °C); ^{1}H NMR analysis revealed that the dr was above 20:1. HPLC analysis (Chiralcel IC, ^{i}PrOH/hexane = 10/90, 0.5 mL/min, 230 nm; t_r (major) = 19.48 min, t_r (minor) = 21.92 min) gave the isomeric composition of the product: 95% ee, $[\alpha]^{25}_{D}$ = -67.5 (c = 1.0, $CHCl_3$); ^{1}H NMR (400 MHz, $CDCl_3$): 8.23 (dd, J_1 = 8.0 Hz, J_2 = 1.6 Hz, 1H), 7.86 (d, J = 2.0 Hz, 1H), 7.40 (dd, J_1 = 8.4 Hz, J_2 = 2.0 Hz, 1H), 7.32 (d, J = 8.0 Hz, 2H), 7.21-7.08 (m, 5H), 6.94 (s, br, 1H), 6.66 (s, br, 1H), 5.51 (dd, J_1 = 12.0 Hz, J_2 = 2.0 Hz, 1H), 4.17 (s, 1H), 4.13-4.09 (m, 1H), 3.99-3.94 (m, 1H), 3.78-3.72 (m, 1H), 3.48-3.44 (m, 1H), 2.54-2.47 (m, 1H), 2.49 (s, 3H), 2.34 (s, 3H), 1.86 (dd, J_1 = 13.2 Hz, J_2 = 2.4 Hz, 1H), 1.25 (td, J_1 = 7.2 Hz, J_2 = 0.8 Hz, 3H), 1.16 (td, J_1 = 7.2 Hz, J_2 = 0.8 Hz, 3H); ^{13}C NMR (100 MHz, $CDCl_3$): 178.46, 141.79 (d, J = 6.7 Hz), 138.20, 136.65, 135.64, 130.59, 129.94 (d, J = 9.1 Hz), 129.28, 128.14, 128.10, 127.95, 127.74, 126.52, 122.52, 117.38, 75.68, 75.54, 64.70 (d, J = 5.7 Hz), 64.07 (d, J = 5.6 Hz), 54.08 (d, J = 5.6 Hz), 44.70, 41.57, 21.20, 15.98 (d, J = 7.2 Hz), 15.90 (d, J = 8.1 Hz); ^{31}P NMR (122 MHz, $CDCl_3$): -6.68; IR (ATR): 2989, 1743, 1598, 1465, 1412, 1288, 1164, 1033 cm^{-1}; MS (EI): 598, 600 (M^+, 2, 2), 465 (100), 463 (98), 136 (61), 118 (61), 119 (39), 91 (33), 464 (25); HRMS (EI): Exact mass calcd for $C_{29}H_{32}N_2O_5P^{79}Br$ $[M]^+$: 598.1232, Found: 598.1237.

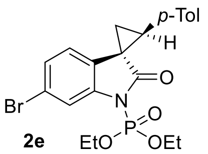

2e

2e was obtained in 34% recovery as a white solid (m.p. 110-111 °C); HPLC analysis (Chiralcel IC, ^{i}PrOH/hexane = 15/85, 1.0 mL/min, 230 nm; t_r (major) = 15.52 min, t_r (minor) = 24.20 min) gave the isomeric composition of the recovered: 99% ee, $[\alpha]^{25}_{D}$ = -148.8 (c = 1.0, $CHCl_3$); ^{1}H NMR (400 MHz, $CDCl_3$): 8.04 (d, J = 2.0 Hz, 1H), 7.10 (d, J = 7.6 Hz, 2H), 7.02 (d, J = 7.6 Hz, 2H), 6.89 (dd, J_1 = 8.0 Hz, J_2 = 1.6 Hz, 1H), 5.78 (dd, J_1 = 8.4 Hz, J_2 = 1.6 Hz, 1H), 4.37-4.24 (m, 4H), 3.33 (t, J = 8.8 Hz, 1H), 2.33 (s, 3H), 2.26 (dd, J_1 = 9.2 Hz, J_2 = 4.4 Hz, 1H), 2.00 (dd, J_1 = 8.4 Hz, J_2 = 4.8 Hz, 1H), 1.44-1.37 (m, 6H); ^{13}C NMR (100 MHz, $CDCl_3$): 177.74, 142.00 (d, J = 6.5 Hz), 137.66, 130.73, 129.73, 129.31, 126.59 (d, J = 8.7 Hz), 126.08, 121.64, 120.57, 117.20, 64.83 (d, J = 3.6 Hz), 64.77 (d, J = 3.6 Hz), 38.14, 33.72 (d, J = 7.9 Hz), 24.28, 21.23, 16.16 (d, J = 2.0 Hz), 16.10 (d, J = 2.3 Hz); ^{31}P NMR (122 MHz, $CDCl_3$): -5.78; IR (ATR): 2921, 2853, 1736, 1607, 1469, 1415, 1278, 1240, 1145, 1030 cm^{-1}; MS (EI): 463, 465 (M^+, 100, 100), 230 (53), 409 (41), 311 (41), 407 (40), 81 (36), 204 (34), 327 (28); HRMS (EI): Exact mass calcd for $C_{21}H_{23}NO_4P^{79}Br$ $[M]^+$: 463.0548, Found: 463.0545.

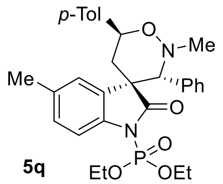

5q was obtained in 34% yield as a white solid (m.p. 125-130 °C); ^{1}H NMR analysis revealed that the dr was above 20:1. HPLC analysis (Chiralcel IC, ^{i}PrOH/hexane = 20/80, 1.0 mL/min, 230 nm; t_r (major) = 8.35 min, t_r (minor) = 9.68 min) gave the isomeric composition of the product: 92% ee, $[\alpha]^{25}_{D}$ = -64.6 (c = 1.0, $CHCl_3$); ^{1}H NMR (400 MHz, $CDCl_3$): 8.16 (s, 1H), 7.47 (d, J = 8.0 Hz, 1H), 7.35 (d, J = 8.0 Hz, 2H), 7.22-7.05 (m, 6H), 6.88 (s, br, 1H), 6.66 (s, br, 1H), 5.60 (dd, J_1 = 12.0 Hz, J_2 = 2.0 Hz, 1H), 4.17 (s, 1H), 4.08-4.04 (m, 1H), 3.90-3.84 (m, 1H), 3.74-3.68 (m, 1H), 3.50-3.44 (m, 1H), 2.55-2.52 (m, 1H), 2.49 (s, 6H), 2.34 (s, 3H), 1.90 (dd, J_1 = 13.2 Hz, J_2 = 2.4 Hz, 1H), 1.22 (td, J_1 = 6.8 Hz, J_2 = 0.8 Hz, 3H), 1.14 (td, J_1 = 7.2 Hz, J_2 = 0.8 Hz, 3H); ^{13}C NMR (100 MHz, $CDCl_3$): 178.93, 138.13 (d, J = 6.6 Hz), 138.04, 136.94, 135.95, 132.93, 130.88 (d, J = 9.2 Hz), 130.73, 129.21, 129.17, 129.03, 127.86, 127.75, 127.48, 126.57, 113.67, 75.90, 75.71, 64.31 (d, J = 5.7 Hz), 63.77 (d, J = 5.5 Hz), 54.24 (d, J = 6.0 Hz), 44.84, 41.51, 21.39, 21.20, 15.96 (d, J = 6.8 Hz), 15.89 (d, J = 7.2 Hz); ^{31}P NMR (162 MHz, $CDCl_3$): -6.32; IR (ATR): 2919, 1732, 1516, 1481, 1309, 1180, 1030 cm^{-1}; MS (EI): 534 (M^+, 4), 399 (100), 118 (34), 263 (33), 400 (23), 136 (23), 119 (23), 91 (16); HRMS (EI): Exact mass calcd for $C_{30}H_{35}N_2O_5P$ $[M]^+$: 534.2284, Found: 534.2288.

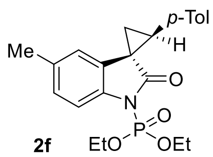

2f was obtained in 47% recovery as a white solid (m.p. 84-85 °C); HPLC analysis (Chiralcel IC, ^{i}PrOH/hexane = 20/80, 1.0 mL/min, 230 nm; t_r (major) = 12.17 min, t_r (minor) = 15.59 min) gave the isomeric composition of the recovered: 70% ee, $[\alpha]^{25}_{D}$ = -35.3 (c = 1.0, $CHCl_3$); ^{1}H NMR (400 MHz, $CDCl_3$): 7.67 (d, J = 8.4 Hz, 1H), 7.10 (d, J = 8.0 Hz, 2H), 7.04 (d J = 7.6 Hz, 2H), 6.95-6.92 (m, 1H), 5.76 (s, 1H), 4.35-4.22 (m, 4H), 3.30 (t, J = 8.4 Hz, 1H), 2.33 (s, 3H), 2.22 (dd, J_1 = 9.2 Hz, J_2 = 4.4 Hz, 1H), 2.03 (s, 3H), 1.96 (dd, J_1 = 8.4 Hz, J_2 = 4.8 Hz, 1H), 1.42-1.36 (m, 6H); ^{13}C NMR (100 MHz, $CDCl_3$): 178.28, 138.54 (d, J = 6.5 Hz), 137.25, 132.43, 131.18, 129.79, 129.04, 127.50 (d, J = 8.8 Hz), 127.33, 121.34, 113.46, 64.47 (d, J = 4.3 Hz), 64.42 (d, J = 4.3 Hz), 37.76, 33.89 (d, J = 8.2 Hz), 24.08, 21.13, 20.91, 16.11 (d, J = 1.8 Hz), 16.04 (d, J = 2.1 Hz); ^{31}P NMR (122 MHz, $CDCl_3$): -5.44; IR (ATR): 2975, 1733, 1477, 1149, 1021 cm^{-1}; MS (EI): 399 (M^+, 100), 245 (82), 343 (45), 371 (41), 263 (37), 246 (30), 262 (25), 400 (24); HRMS (EI): Exact mass calcd for $C_{22}H_{26}NO_4P$ $[M]^+$: 399.1599, Found: 399.1593.

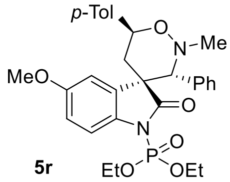

5r was obtained in 35% yield as a white solid (m.p. 60-65 ºC); ^{1}H NMR analysis revealed that the dr was above 20:1. HPLC analysis (Chiralcel IC, ^{i}PrOH/hexane = 20/80, 1.0 mL/min, 230 nm; t_r (major) = 9.28 min, t_r (minor) = 10.82 min) gave the isomeric composition of the product: 90% ee, $[\alpha]^{25}_{D}$ = -81.8 (c = 1.0, $CHCl_3$); ^{1}H NMR (400 MHz, $CDCl_3$): 7.97 (s, 1H), 7.52 (d, J = 8.8 Hz, 1H), 7.33 (d, J = 7.6 Hz, 2H), 7.22-7.06 (m, 5H), 6.91 (s, br, 1H), 6.81 (dd, J_1 = 8.8 Hz, J_2 = 2.8 Hz, 1H), 6.73 (s, br, 1H), 5.56 (dd, J_1 = 12.0 Hz, J_2 = 2.4 Hz, 1H), 4.18 (s, 1H), 4.11-4.04 (m, 1H), 3.92 (s, 3H), 3.92-3.88 (m, 1H), 3.73-3.67 (m, 1H), 3.49-3.42 (m, 1H), 2.54-2.51 (m, 1H), 2.48 (s, 3H), 2.34 (s, 3H), 1.89 (dd, J_1 = 13.2 Hz, J_2 = 2.4 Hz, 1H), 1.22 (td, J_1 = 7.2 Hz, J_2 = 0.8 Hz, 3H), 1.14 (td, J_1 = 7.2 Hz, J_2 = 1.2 Hz, 3H); ^{13}C NMR (100 MHz, $CDCl_3$): 178.80, 156.01, 138.08, 136.89, 135.94, 134.02 (d, J = 6.7 Hz), 132.24 (d, J = 9.4 Hz), 130.74, 129.24, 127.90, 127.80, 126.57, 114.47, 114.23, 112.65, 75.72, 75.70, 64.36 (d, J = 5.6 Hz), 63.78 (d, J = 5.5 Hz), 55.96, 54.54 (d, J = 5.9 Hz), 44.73, 41.60, 21.21, 15.98 (d, J = 7.0 Hz), 15.91 (d, J = 7.7 Hz); ^{31}P NMR (162 MHz, $CDCl_3$): -6.25; IR (ATR): 2916, 1738, 1516, 1479, 1295, 1260, 1171, 1023 cm^{-1}; MS (EI): 550 (M^+, 2), 415 (100), 43 (53), 57 (48), 118 (34), 77 (28), 279 (25), 119 (25); HRMS (EI): Exact mass calcd for $C_{30}H_{35}N_2O_6P$ $[M]^+$: 550.2233, Found: 550.2238.

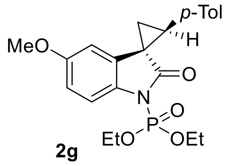

2g was obtained in 52% recovery as a white solid (m.p. 69-70 ºC); HPLC analysis (Chiralcel IC, ^{i}PrOH/hexane = 20/80, 1.0 mL/min, 230 nm; t_r (major) = 17.73 min, t_r (minor) = 22.75 min) gave the isomeric composition of the recovered: 70% ee, $[\alpha]^{25}_{D}$ = -61.2 (c = 1.0, $CHCl_3$); ^{1}H NMR (400 MHz, $CDCl_3$): 7.69 (d, J = 8.8 Hz, 1H), 7.11 (d, J = 8.0 Hz, 2H), 7.05 (d, J = 7.6 Hz, 2H), 6.66 (dd, J_1 = 8.8 Hz, J_2 = 2.4 Hz, 1H), 5.50 (s, 1H), 4.35-4.23 (m, 4H), 3.42 (s, 3H), 3.33 (t, J = 8.4 Hz, 1H), 2.32 (s, 3H), 2.23 (dd, J_1 = 13.2 Hz, J_2 = 4.8 Hz, 1H), 1.98 (dd, J_1 = 8.0 Hz, J_2 = 4.8 Hz, 1H), 1.42-1.36 (m, 6H); ^{13}C NMR (100 MHz, $CDCl_3$): 178.20, 155.68, 137.40, 134.29 (d, J = 6.8 Hz), 131.09, 129.84, 129.18, 128.80 (d, J = 9.1 Hz), 114.42, 112.32, 106.65, 64.50 (d, J = 5.2 Hz), 64.45 (d, J = 5.2 Hz), 55.28, 37.77, 34.14 (d, J = 8.4 Hz), 24.22, 21.14, 16.13 (d, J = 2.1 Hz), 16.06 (d, J = 2.3 Hz); ^{31}P NMR (122 MHz, $CDCl_3$): -5.34; IR (ATR): 2984, 2926, 1732, 1478, 1293, 1146, 1020 cm^{-1}; MS (GC-MS): 415 (M^+, 100), 279 (52), 246 (44), 207 (42), 359 (38), 387 (37), 261 (37), 264 (16); HRMS (EI): Exact mass calcd for $C_{22}H_{26}NO_5P$ $[M]^+$: 415.1549, Found: 415.1552.

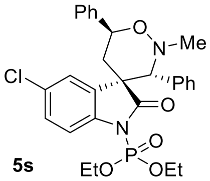

5s

5s was obtained in 48% yield as a white solid (m.p. 99-101 °C); ^{1}H NMR analysis revealed that the dr was above 20:1. HPLC analysis (Chiralcel IC, ^{i}PrOH/hexane = 10/90, 1.0 mL/min, 230 nm; t_r (major) = 8.73 min, t_r (minor) = 11.25 min) gave the isomeric composition of the product: 95% ee, $[\alpha]^{25}_{D}$ = -91.4 (c = 1.0, $CHCl_3$); ^{1}H NMR (400 MHz, $CDCl_3$): 8.36 (s, 1H), 7.58 (d, J = 8.8 Hz , 1H), 7.46-7.44 (m, 2H), 7.40-7.30 (m, 3H), 7.28-7.25 (m, 1H), 7.22-7.07 (m, 3H), 6.95 (s, br, 1H), 6.68 (s, br, 1H), 5.56 (dd, J_1 = 12.0 Hz, J_2 = 2.4 Hz, 1H), 4.19 (s, 1H), 4.12-4.08 (m, 1H), 3.98-3.93 (m, 1H), 3.74-3.70 (m, 1H), 3.51-3.47 (m, 1H), 2.55-2.49 (m, 1H), 2.52 (s, 3H), 1.92 (dd, J_1 = 13.2 Hz, J_2 = 2.4 Hz, 1H), 1.24 (td, J_1 = 6.8 Hz, J_2 = 0.8 Hz, 3H), 1.15 (td, J_1 = 7.2 Hz, J_2 = 1.2 Hz, 3H); ^{13}C NMR (100 MHz, $CDCl_3$): 178.17, 139.56, 139.17 (d, J = 7.0 Hz), 135.48, 132.60 (d, J = 9.5 Hz), 130.56, 128.94, 128.55, 128.30, 128.06, 127.90, 127.70, 126.91, 126.48, 115.16, 75.66, 75.54, 64.63 (d, J = 5.7 Hz), 63.98 (d, J = 5.7 Hz), 54.34 (d, J = 5.8 Hz), 44.64, 41.35, 15.93 (d, J = 7.1 Hz), 15.86 (d, J = 7.8 Hz); ^{31}P NMR (162 MHz, $CDCl_3$): -6.66; IR (ATR): 2980, 2916, 1741, 1465, 1290, 1165, 1021 cm^{-1}; MS (EI): 540, 542 (M^+, 6, 3), 405 (100), 136 (44), 407 (34), 406 (22), 118 (21), 349 (20), 377 (17); HRMS (EI): Exact mass calcd for $C_{28}H_{30}N_2O_5P^{35}Cl$ $[M]^+$: 540.1581, Found: 540.1580.

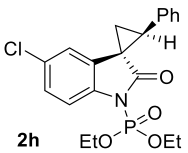

2h

2h was obtained in 48% recovery as a white solid (m.p. 97-98 °C); HPLC analysis (Chiralcel AD-H, ^{i}PrOH/hexane = 10/90, 1.0 mL/min, 230 nm; t_r (major) = 10.13 min, t_r (minor) = 12.20 min) gave the isomeric composition of the recovered: 99% ee, $[\alpha]^{25}_{D}$ = -49.7 (c = 1.0, $CHCl_3$); ^{1}H NMR (400 MHz, $CDCl_3$): 7.76 (d, J = 8.4 Hz, 1H), 7.36-7.30 (m, 3H), 7.16-7.14 (m, 2H), 7.10 (dd, J_1 = 8.4 Hz, J_2 = 2.0 Hz, 1H), 5.84 (s, 1H), 4.36-4.24 (m, 4H), 3.40 (t, J = 8.4 Hz, 1H), 2.29 (dd, J_1 = 9.2 Hz, J_2 = 4.4 Hz, 1H), 2.04 (dd, J_1 = 8.4 Hz, J_2 = 4.8 Hz, 1H), 1.43-1.36 (m, 6H); ^{13}C NMR (100 MHz, $CDCl_3$): 177.56, 139.46 (d, J = 6.8 Hz), 133.61, 129.87, 129.33 (d, J = 9.0 Hz), 128.68, 128.60, 128.08, 126.91, 120.74, 114.96, 64.81 (d, J = 3.4 Hz), 64.76 (d, J = 3.4 Hz), 38.41, 33.84 (d, J = 8.0 Hz), 24.47, 16.15 (d, J = 2.1 Hz), 16.09 (d, J = 2.4 Hz); ^{31}P NMR (122 MHz, $CDCl_3$): -5.76; IR (ATR): 2984, 1737, 1468, 1287, 1249, 1202, 1149, 1018 cm^{-1}; MS (GC-MS): 405, 407 (M^+, 100, 34), 251 (82), 349 (80), 377 (50), 269 (35), 216 (30), 406 (26), 204 (23); HRMS (EI): Exact mass calcd for $C_{20}H_{21}NO_4P^{35}Cl$ $[M]^+$: 405.0897, Found: 405.0896.

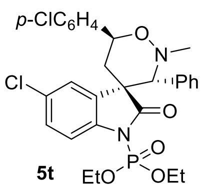

5t was obtained in 46% yield as a white solid (m.p. 117-120 °C); ^{1}H NMR analysis revealed that the dr was above 20:1. HPLC analysis (Chiralcel IC, $^{i}PrOH$/hexane = 10/90, 1.0 mL/min, 230 nm; t_r (major) = 8.78 min, t_r (minor) = 11.67 min) gave the isomeric composition of the product: 97% ee, $[\alpha]^{25}_{D}$ = -67.7 (c = 1.0, $CHCl_3$); ^{1}H NMR (400 MHz, $CDCl_3$): 8.32 (s, 1H), 7.57 (d, J = 8.8 Hz, 1H), 7.40-7.33 (m, 4H), 7.27 (dd, J_1 = 8.8 Hz, J_2 = 2.0 Hz, 1H), 7.21-7.07 (m, 3H), 6.94 (t, J = 8.0 Hz, 1H), 6.64 (d, J = 8.0 Hz, 1H), 5.52 (dd, J_1 = 12.4 Hz, J_2 = 2.4 Hz, 1H), 4.17 (s, 1H), 4.14-4.08 (m, 1H), 3.96-3.90 (m, 1H), 3.75-3.69 (m, 1H), 3.48-3.42 (m, 1H), 2.50 (s, 3H), 2.45 (dd, J_1 = 13.2 Hz, J_2 = 12.0 Hz, 1H), 1.90 (dd, J_1 = 13.2 Hz, J_2 = 2.4 Hz, 1H), 1.24 (td, J_1 = 7.2 Hz, J_2 = 0.8 Hz, 3H), 1.14 (td, J_1 = 6.8 Hz, J_2 = 0.8 Hz, 3H); ^{13}C NMR (100 MHz, $CDCl_3$): 178.08, 139.14 (d, J = 7.1 Hz), 138.05, 135.28, 134.08, 132.39 (d, J = 9.4 Hz), 130.56, 128.99, 128.74, 128.68, 128.16, 127.97, 127.90, 127.67, 126.86, 115.21, 75.49, 75.02, 64.70 (d, J = 5.7 Hz), 64.03 (d, J = 5.6 Hz), 54.23 (d, J = 5.8 Hz), 44.68, 41.26, 15.98 (d, J = 7.3 Hz), 15.90 (d, J = 8.4 Hz); ^{31}P NMR (122 MHz, $CDCl_3$): -6.74; IR (ATR): 2980, 1742, 1468, 1279, 1164, 1029 cm^{-1}; MS (EI): 574, 576 (M^+, 7, 3), 439 (100), 136 (80), 44 (68), 441 (63), 91 (36), 118 (31), 43 (29); HRMS (EI): Exact mass calcd for $C_{28}H_{29}N_2O_5P^{35}Cl_2$ $[M]^+$: 574.1191, Found: 574.1187.

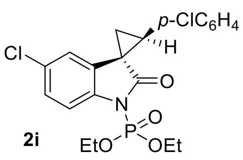

2i was obtained in 48% recovery as a white solid (m.p. 84-87 °C); HPLC analysis (Chiralcel AD-H, $^{i}PrOH$/hexane = 10/90, 1.0 mL/min, 230 nm; t_r (major) = 11.01 min, t_r (minor) = 12.55 min) gave the isomeric composition of the recovered: 94% ee, $[\alpha]^{25}_{D}$ = -75.8 (c = 1.0, $CHCl_3$); ^{1}H NMR (400 MHz, $CDCl_3$): 7.77 (d, J = 8.8 Hz, 1H), 7.30 (d, J = 8.4 Hz, 2H), 7.12 (dd, J_1 = 8.4 Hz, J_2 = 2.0 Hz, 1H), 7.09 (d, J = 8.0 Hz, 2H), 5.88 (s, 1H), 4.36-4.23 (m, 4H), 3.32 (t, J = 8.4 Hz, 1H), 2.28 (dd, J_1 = 9.2 Hz, J_2 = 4.8 Hz, 1H), 1.97 (dd, J_1 = 8.4 Hz, J_2 = 4.8 Hz, 1H), 1.42-1.36 (m, 6H); ^{13}C NMR (100 MHz, $CDCl_3$): 177.28, 139.52 (d, J = 6.9 Hz), 133.92, 132.19, 131.19, 128.96, 128.89, 128.87, 128.74, 127.17, 120.68, 115.12, 64.86 (d, J = 5.8 Hz), 64.79 (d, J = 5.8 Hz), 37.46, 33.80 (d, J = 8.1 Hz), 24.38, 16.14 (d, J = 3.1 Hz), 16.08 (d, J = 3.6 Hz); ^{31}P NMR (122 MHz, $CDCl_3$): -5.84; IR (ATR): 2983, 1737, 1468, 1287, 1196, 1150, 1015 cm^{-1}; MS (GC-MS): 439, 441, 443 (M^+, 92, 61, 11), 383 (100), 411 (51), 303 (40), 268 (36), 250 (34), 287 (32), 238 (14); HRMS (EI): Exact mass calcd for $C_{20}H_{20}NO_4P^{35}Cl_2$ $[M]^+$: 439.0507, Found: 439.0513.

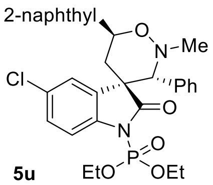

5u

5u was obtained in 47% yield as a white solid (m.p. 124-127 °C); ^{1}H NMR analysis revealed that the dr was above 20:1. HPLC analysis (Chiralcel IC, ^{i}PrOH/hexane = 10/90, 1.0 mL/min, 230 nm; t_r (major) = 11.69 min, t_r (minor) = 14.71 min) gave the isomeric composition of the product: 93% ee, $[\alpha]^{25}_D$ = -45.7 (c = 1.0, $CHCl_3$); ^{1}H NMR (400 MHz, $CDCl_3$): 8.42 (s, 1H), 7.90 (s, 1H), 7.88-7.82 (m, 3H), 7.61-7.57 (m, 2H), 7.51-7.46 (m, 2H), 7.29 (dd, J_1 = 8.4 Hz, J_2 = 2.0 Hz, 1H), 7.23-7.09 (m, 3H), 6.97 (s, br, 1H), 6.71 (s, br, 1H), 5.73 (dd, J_1 = 12.0 Hz, J_2 = 2.4 Hz, 1H), 4.25 (s, 1H), 4.12-4.08 (m, 1H), 3.98-3.94 (m, 1H), 3.76-3.71 (m, 1H), 3.52-3.46 (m, 1H), 2.62 (dd, J_1 = 12.8 Hz, J_2 = 12.0 Hz, 1H), 2.56 (s, 3H), 2.00 (dd, J_1 = 13.2 Hz, J_2 = 2.8 Hz, 1H), 1.24 (td, J_1 = 6.8 Hz, J_2 = 0.8 Hz, 3H), 1.16 (td, J_1 = 6.8 Hz, J_2 = 1.2 Hz, 3H); ^{13}C NMR (100 MHz, $CDCl_3$): 178.20, 139.19 (d, J = 7.0 Hz), 136.93, 135.47, 133.22, 133.20, 132.62 (d, J = 9.4 Hz), 130.57, 128.99, 128.60, 128.31, 128.08, 128.03, 127.92, 127.72, 127.63, 126.95, 126.21, 126.15, 125.50, 124.36, 115.19, 75.78, 75.59, 64.64 (d, J = 5.7 Hz), 64.01 (d, J = 5.6 Hz), 54.40 (d, J = 5.8 Hz), 44.69, 41.37, 15.92 (d, J = 6.5 Hz), 15.85 (d, J = 7.3 Hz); ^{31}P NMR (122 MHz, $CDCl_3$): -6.79; IR (ATR): 2980, 2914, 1741, 1465, 1164, 1020 cm^{-1}; MS (EI): 590, 592 (M^+, 4, 1), 455 (100), 136 (71), 457 (36), 456 (27), 118 (21), 301 (21), 127 (12); HRMS (EI): Exact mass calcd for $C_{32}H_{32}N_2O_5P^{35}Cl$ $[M]^+$: 590.1737, Found: 554.1740.

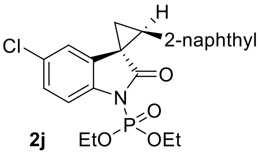

2j

2j was obtained in 44% recovery as a white solid (m.p. 111-112 °C); HPLC analysis (Chiralcel OD-H, ^{i}PrOH/hexane = 10/90, 1.0 mL/min, 230 nm; t_r (minor) = 12.19 min, t_r (major) = 14.07 min) gave the isomeric composition of the recovered: 99% ee, $[\alpha]^{25}_D$ = -149.8 (c = 1.0, $CHCl_3$); ^{1}H NMR (400 MHz, $CDCl_3$): 7.84-7.81 (m, 2H), 7.78-7.74 (m, 3H), 7.52-7.49 (m, 2H), 7.15 (dd, J_1 = 8.8 Hz, J_2 = 2.0 Hz, 1H), 7.07 (dd, J_1 = 8.8 Hz, J_2 = 2.4 Hz, 1H), 5.90 (s, 1H), 4.40-4.25 (m, 4H), 3.53 (t, J = 8.8 Hz, 1H), 2.37 (dd, J_1 = 9.2 Hz, J_2 = 4.8 Hz, 1H), 2.18 (dd, J_1 = 8.4 Hz, J_2 = 4.8 Hz, 1H), 1.46-1.38 (m, 6H); ^{13}C NMR (100 MHz, $CDCl_3$): 177.52, 139.48 (d, J = 6.9 Hz), 133.13, 132.82, 131.18, 129.25 (d, J = 9.1 Hz), 128.64, 128.52, 128.44, 127.80, 127.73, 127.61, 126.98, 126.49, 126.35, 120.64, 115.00, 64.86 (d, J = 5.8 Hz), 64.78 (d, J = 5.9 Hz), 38.72, 34.02 (d, J = 8.1 Hz), 24.62, 16.17 (d, J = 3.8 Hz), 16.10 (d, J = 4.3 Hz); ^{31}P NMR (122 MHz, $CDCl_3$): -5.75; IR (ATR): 2983, 1736, 1466, 1286, 1146, 1017 cm^{-1}; MS (EI): 455, 457 (M^+, 94, 38), 301 (100), 254 (37), 302 (36), 303 (34), 266 (26), 44 (26), 427 (13); HRMS (EI): Exact mass calcd for $C_{24}H_{23}NO_4P^{35}Cl$ $[M]^+$: 455.1053, Found: 453.1049.

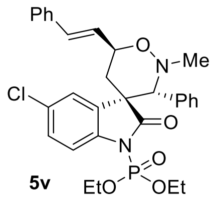

5v

5v was obtained in 48% yield as a white solid (m.p. 62-63 ºC); ^{1}H NMR analysis revealed that the dr was above 20:1. HPLC analysis (Chiralcel IC, ^{i}PrOH/hexane = 10/90, 1.0 mL/min, 230 nm; t_r (major) = 10.77 min, t_r (minor) = 12.97 min) gave the isomeric composition of the product: 90% ee, $[\alpha]^{25}_D$ = -87.6 (c = 1.0, $CHCl_3$); ^{1}H NMR (400 MHz, $CDCl_3$): 8.28 (s, 1H), 7.57 (d, J = 8.8 Hz, 1H), 7.41-7.30 (m, 2H), 7.34-7.30 (m, 2H), 7.27-7.24 (m, 2H), 7.18-7.07 (m, 3H), 6.94 (s, br, 1H), 6.72 (d, J = 16.0 Hz, 1H), 6.64 (s, br, 1H), 6.20 (dd, J_1 = 12.0 Hz, J_2 = 6.4 Hz, 1H), 5.23-5.18 (m, 1H), 4.18-4.08 (m, 1H), 4.09 (s, 1H), 3.98-3.92 (m, 1H), 3.78-3.68 (m, 1H), 3.50-3.42 (m, 1H), 2.49 (s, 3H), 2.37 (dd, J_1 = 13.2 Hz, J_2 = 12.0 Hz, 1H), 1.83 (dd, J_1 = 13.2 Hz, J_2 = 2.4 Hz, 1H), 1.26 (td, J_1 = 6.8 Hz, J_2 = 0.8 Hz, 3H), 1.15 (td, J_1 = 7.2 Hz, J_2 = 1.2 Hz, 3H); ^{13}C NMR (100 MHz, $CDCl_3$): 178.22, 139.14 (d, J = 7.0 Hz), 136.25, 135.49, 133.10, 132.57 (d, J = 9.4 Hz), 130.60, 128.95, 128.60, 128.57, 128.10, 127.93, 127.70, 126.93, 126.82, 126.64, 115.15, 75.49, 74.27, 64.68 (d, J = 5.7 Hz), 64.02 (d, J = 5.5 Hz), 54.10 (d, J = 5.8 Hz), 44.64, 39.95, 15.98 (d, J = 6.8 Hz), 15.89 (d, J = 7.7 Hz); ^{31}P NMR (122 MHz, $CDCl_3$): -6.65; IR (ATR): 2988, 1736, 1470, 1310, 1166, 1034 cm^{-1}; MS (EI): 566 (M^+, 2), 136 (100), 431 (98), 118 (73), 44 (44), 91 (36), 433 (35), 77 (34); HRMS (EI): Exact mass calcd for $C_{30}H_{32}N_2O_5P^{35}Cl$ $[M]^+$: 566.1737, Found: 566.1730.

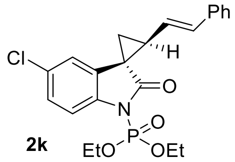

2k

2k was obtained in 40% recovery as a yellow oil; HPLC analysis (Chiralcel AD-H, ^{i}PrOH/hexane = 15/85, 1.0 mL/min, 230 nm; t_r (minor) = 8.65 min, t_r (major) = 11.93 min) gave the isomeric composition of the recovered: 98% ee, $[\alpha]^{25}_D$ = -130.2 (c = 1.0, $CHCl_3$); ^{1}H NMR (400 MHz, $CDCl_3$): 7.82 (d, J = 8.4 Hz, 1H), 7.35-7.24 (m, 5H), 7.21 (dd, J_1 = 8.8 Hz, J_2 = 2.4 Hz, 1H), 6.92 (s, 1H), 6.71 (d, J = 15.6 Hz, 1H), 6.12 (dd, J_1 = 15.6 Hz, J_2 = 7.6 Hz, 1H), 4.36-4.20 (m, 4H), 2.87-2.80 (m, 1H), 2.24 (dd, J_1 = 9.2 Hz, J_2 = 4.8 Hz, 1H), 1.83 (dd, J_1 = 8.0 Hz, J_2 = 4.8 Hz, 1H), 1.40-1.36 (m, 6H); ^{13}C NMR (100 MHz, $CDCl_3$): 177.15, 139.82 (d, J = 6.9 Hz), 136.26, 135.45, 129.45 (d, J = 9.0 Hz), 128.98, 128.75, 128.08, 127.16, 126.28, 123.61, 120.92, 115.44, 64.82 (d, J = 5.5 Hz), 64.77 (d, J = 5.5 Hz), 38.07, 34.69 (d, J = 8.0 Hz), 25.24, 16.13, 16.07; ^{31}P NMR (122 MHz, $CDCl_3$): -5.84; IR (ATR): 2985, 1737, 1471, 1289, 1166, 1027 cm^{-1}; MS (GC-MS): 431, 433 (M^+, 88, 32), 277 (100), 242 (23), 316 (21), 260 (20), 115 (19), 403 (12), 204 (12); HRMS (EI): Exact mass calcd for $C_{22}H_{23}NO_4P^{35}Cl$ $[M]^+$: 431.1053, Found: 431.1056.

Catalytic enantioselective [3+3] cycloaddition of 2 and ketonitrone 9

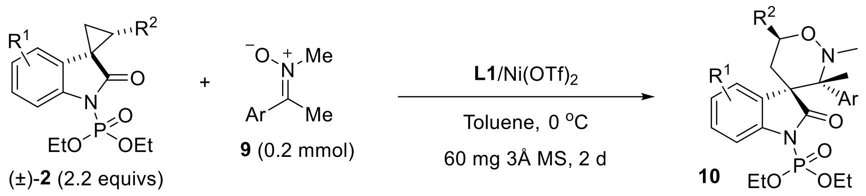

General procedure: To a Schlenk tube was sequentially added $Ni(OTf)_2$ (14.3 mg, 0.040 mmol) and $\mathbf{L_1}$ (15.8 mg, 0.044 mmol), followed by the addition of anhydrous toluene (2.0 mL). After the resulting solution was stirred at room temperature for 2 h and cooled to 0 °C, nitrone **9** (0.2 mmol), oxindole **2** (0.44 mmol) and MS 3Å (60 mg) were added successively. The reaction was kept stirring at 0 °C for 2 days. Then toluene was removed under reduced pressure. The residue was dissolved in CH_2Cl_2, rapidly passed through a glass funnel with a thin layer (5 mm) of silica gel (100 mesh), washed with CH_2Cl_2, and concentrated under reduced pressure. To determine the dr value of product, the residue was first dissolved in $CDCl_3$, and took some samples to determine diastereoselectivity by ^{1}H NMR analysis. Then the sample for analysis and rest crude product were recombined for column chromatography purification to afford product **10**, using DCM/EtOAc (30/1, v/v) as the eluent.

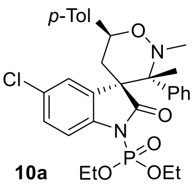

10a

Product **10a** was obtained in 78% yield as a white solid (m.p. 113-115 °C); NMR analysis showed the dr value was 12:1. HPLC analysis (Chiralcel IF, ^{i}PrOH/hexane = 10/90, 0.8 mL/min, 230 nm; t_r (minor) = 11.85 min, t_r (major) = 14.62 min) gave the isomeric composition of the product: 95% ee, $[\alpha]^{25}_{D}$ = -60.3 (c = 1.0, $CHCl_3$); ^{1}H NMR (400 MHz, $CDCl_3$): 8.53 (s, 1H), 7.41 (d, J = 8.8 Hz, 1H), 7.36 (d, J = 8.0 Hz, 2H), 7.19 (d, J = 8.0 Hz, 2H), 7.12 (dd, J_1 = 8.8 Hz, J_2 = 2.4 Hz, 1H), 7.10 (s, br, 2H), 6.98-6.97 (m, 3H), 5.53 (dd, J_1 = 12.4 Hz, J_2 = 2.8 Hz, 1H), 4.16-4.05 (m, 2H), 3.99-3.93 (m, 2H), 2.67 (dd, J_1 = 13.6 Hz, J_2 = 12.4 Hz, 1H), 2.62 (s, 3H), 2.35 (s, 3H), 2.12 (s, 3H), 1.66 (dd, J_1 = 13.6 Hz, J_2 = 2.8 Hz, 1H), 1.35 (td, J_1 = 6.8 Hz, J_2 = 0.8 Hz, 3H), 1.24 (td, J_1 = 7.2 Hz, J_2 = 1.2 Hz, 3H); ^{13}C NMR (100 MHz, $CDCl_3$): 178.28, 140.74, 138.33 (d, J = 6.8 Hz), 138.19, 136.94, 133.93 (d, J = 9.7 Hz), 129.30, 128.57, 128.24, 128.02, 127.82, 126.90, 126.82, 126.57, 114.46, 74.80, 67.82, 64.54, 64.48, 56.72 (d, J = 5.5 Hz), 38.77 (d, J = 1.6 Hz), 38.06, 21.21, 16.10 (d, J = 7.2 Hz), 15.98 (d, J = 6.7 Hz), 10.06; ^{31}P NMR (162 MHz, $CDCl_3$): -6.85; IR (KBr): 2982, 2922, 1739, 1601, 1493, 1287, 1191, 1160, 1116, 1028 cm^{-1}; MS (ESI): 569.1 $[M+H]^+$; HRMS (DART POS): Exact mass calcd for

$C_{30}H_{35}N_2O_5P^{35}Cl$ $[M+H]^+$: 569.1967, Found: 569.1964.

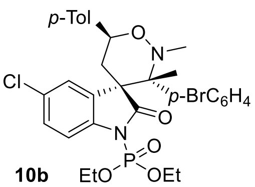

10b

Product **10b** was obtained in 84% yield as a white solid (m.p. 185-188 °C); NMR analysis showed the dr value was 18:1. HPLC analysis (Chiralcel IF, ^{i}PrOH/hexane = 10/90, 1.0 mL/min, 230 nm; t_r (minor) = 10.89 min, t_r (major) = 12.15 min) gave the isomeric composition of the product: 98% ee, $[\alpha]^{25}_D$ = -54.5 (c = 1.0, $CHCl_3$); ^{1}H NMR (400 MHz, $CDCl_3$): 8.50 (s, 1H), 7.40 (d, *J* = 8.8 Hz, 1H), 7.35 (d, *J* = 8.0 Hz, 2H), 7.20-7.16 (m, 3H), 7.12 (d, *J* = 8.0 Hz, 2H), 6.98 (d, *J* = 8.0 Hz, 2H), 5.52 (dd, J_1 = 12.4 Hz, J_2 = 2.8 Hz, 1H), 4.17-4.05 (m, 2H), 4.01-3.92 (m, 2H), 2.65 (dd, J_1 = 13.6 Hz, J_2 = 12.4 Hz, 1H), 2.57 (s, 3H), 2.35 (s, 3H), 2.10 (s, 3H), 1.66 (dd, J_1 = 13.6 Hz, J_2 = 2.8 Hz, 1H), 1.34 (td, J_1 = 7.6 Hz, J_2 = 1.2 Hz, 3H), 1.26 (td, J_1 = 7.6 Hz, J_2 = 1.2 Hz, 3H); ^{13}C NMR (100 MHz, $CDCl_3$): 177.91, 139.80, 138.26, 138.24 (d, *J* = 6.9 Hz), 136.68, 133.52 (d, *J* = 9.5 Hz), 130.00, 129.55, 129.30, 128.46, 128.36, 128.34, 126.53, 121.01, 114.57, 74.76, 67.56, 64.62 (d, *J* = 5.6 Hz), 64.54 (d, *J* = 5.7 Hz), 56.55 (d, *J* = 5.6 Hz), 38.68 (d, *J* = 2.0 Hz), 37.74, 21.20, 16.09 (d, *J* = 7.1 Hz), 15.99 (d, *J* = 6.9 Hz), 9.94; ^{31}P NMR (162 MHz, $CDCl_3$): -6.97; IR (KBr): 2986, 2905, 1736, 1468, 1315, 1190, 1059, 1027 cm^{-1}; MS (ESI): 647.0 $[M+H]^+$; HRMS (DART POS): Exact mass calcd for $C_{30}H_{34}N_2O_5P^{35}Cl^{79}Br$ $[M+H]^+$: 647.1072, Found: 647.1067.

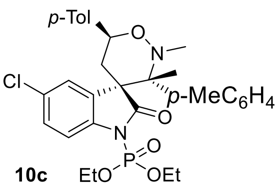

10c

Product **10c** was obtained in 88% yield as a white solid (m.p. 65-67 °C); NMR analysis showed the dr value was 18:1. HPLC analysis (Chiralcel IC + IF, ^{i}PrOH/hexane = 10/90, 1.0 mL/min, 230 nm; t_r (minor) = 18.78 min, t_r (major) = 20.77 min) gave the isomeric composition of the product: 95% ee, $[\alpha]^{25}_D$ = -15.5 (c = 1.0, $CHCl_3$); ^{1}H NMR (400 MHz, $CDCl_3$): 8.54 (s, 1H), 7.42 (d, *J* = 8.8 Hz, 1H), 7.36 (d, *J* = 8.0 Hz, 2H), 7.19 (d, *J* = 8.0 Hz, 2H), 7.14 (dd, J_1 = 8.8 Hz, J_2 = 2.4 Hz, 1H), 6.97 (d, *J* = 7.6 Hz, 2H), 6.78 (d, *J* = 7.6 Hz, 2H), 5.53 (dd, J_1 = 12.4 Hz, J_2 = 2.8 Hz, 1H), 4.15-4.03 (m, 2H), 3.99-3.92 (m, 2H), 2.66 (dd, J_1 = 13.6 Hz, J_2 = 12.4 Hz, 1H), 2.59 (s, 3H), 2.35 (s, 3H), 2.14 (s, 3H), 2.10 (s, 3H), 1.65 (dd, J_1 = 13.6 Hz, J_2 = 2.8 Hz, 1H), 1.34 (td, J_1 = 6.8 Hz, J_2 = 1.2 Hz, 3H), 1.24 (td, J_1 = 7.2 Hz, J_2 = 1.2 Hz, 3H); ^{13}C NMR (100 MHz, $CDCl_3$): 178.29, 138.32 (d, *J* = 6.7 Hz), 138.13, 137.71, 136.96, 136.31, 134.06 (d, *J* = 9.8 Hz), 129.26, 128.59, 128.17, 127.96, 127.70, 127.57, 126.55, 114.42, 74.76, 67.61, 64.48 (d, *J* = 4.0 Hz), 64.42 (d, *J* = 3.8 Hz), 56.76 (d, *J* = 5.6 Hz),

38.67, 38.13, 20.19, 20.81, 16.05 (d, J = 7.5 Hz), 15.96 (d, J = 6.8 Hz), 10.13; ^{31}P NMR (162 MHz, $CDCl_3$): -6.88; IR (KBr): 2989, 2924, 1740, 1467, 1315, 1298, 1192, 1160, 1025 cm^{-1}; MS (ESI): 583.1 [M+H]$^+$; HRMS (DART POS): Exact mass calcd for $C_{31}H_{37}N_2O_5P^{35}Cl$ [M+H]$^+$: 583.2123, Found: 583.2120.

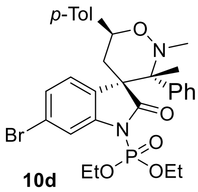

10d

Product **10d** was obtained in 79% yield as a white solid (m.p. 69-72 °C); NMR analysis showed the dr value was 12:1. HPLC analysis (Chiralcel IC + IF, ^{i}PrOH/hexane = 10/90, 1.0 mL/min, 230 nm; t_r (minor) = 17.96 min, t_r (major) = 20.57 min) gave the isomeric composition of the product: 95% ee, $[\alpha]^{25}_D$ = +2.6 (c = 1.0, $CHCl_3$); ^{1}H NMR (400 MHz, $CDCl_3$): 8.41 (dd, J_1 = 8.4 Hz, J_2 = 1.6 Hz, 1H), 7.70 (d, J = 2.0 Hz, 1H), 7.33 (d, J = 8.0 Hz, 2H), 7.29 (dd, J_1 = 8.0 Hz, J_2 = 1.6 Hz, 1H), 7.19 (d, J = 7.6 Hz, 2H), 7.09 (d, J = 6.0 Hz, 2H), 7.02-6.97 (m, 3H), 5.53 (dd, J_1 = 12.4 Hz, J_2 = 2.8 Hz, 1H), 4.18-4.04 (m, 2H), 4.00-3.92 (m, 2H), 2.65 (dd, J_1 = 13.6 Hz, J_2 = 12.4 Hz, 1H), 2.59 (s, 3H), 2.35 (s, 3H), 2.13 (s, 3H), 1.63 (dd, J_1 = 13.6 Hz, J_2 = 2.8 Hz, 1H), 1.36 (td, J_1 = 7.2 Hz, J_2 = 1.2 Hz, 3H), 1.25 (td, J_1 = 6.8 Hz, J_2 = 1.2 Hz, 3H); ^{13}C NMR (100 MHz, $CDCl_3$): 178.40, 140.92 (d, J = 6.4 Hz), 140.78, 138.16, 136.99, 131.23 (d, J = 9.4 Hz), 129.80, 129.30, 127.78, 126.90, 126.51, 125.72, 122.03, 116.67, 74.87, 67.76, 64.56 (d, J = 3.5 Hz), 64.50 (d, J = 3.3 Hz), 56.39 (d, J = 5.4 Hz), 38.72 (d, J = 2.0 Hz), 38.27, 21.21, 16.09 (d, J = 7.3 Hz), 15.98 (d, J = 6.9 Hz), 10.09; ^{31}P NMR (162 MHz, $CDCl_3$): -6.90; IR (KBr): 2982, 2922, 1740, 1595, 1467, 1321, 1189, 1056, 1027 cm^{-1}; MS (ESI): 613.0 [M+H]$^+$; HRMS (DART POS): Exact mass calcd for $C_{30}H_{35}N_2O_5P^{79}Br$ [M+H]$^+$: 613.1461, Found: 613.1459.

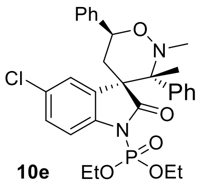

10e

Product **10e** was obtained in 81% yield as a white solid (m.p. 54-56 °C); NMR analysis showed the dr value was 14:1. HPLC analysis (Chiralcel IC + IF, ^{i}PrOH/hexane = 10/90, 1.0 mL/min, 230 nm; t_r (minor) = 17.49 min, t_r (major) = 19.31 min) gave the isomeric composition of the product: 97% ee, $[\alpha]^{25}_D$ = +19.9 (c = 1.0, $CHCl_3$); ^{1}H NMR (400 MHz, $CDCl_3$): 8.53 (s, 1H), 7.48-7.30 (m, 6H), 7.13 (dd, J_1 = 8.8 Hz, J_2 = 2.4 Hz, 1H), 7.09 (s, br, 2H), 6.99-6.97 (m, 3H), 5.57 (dd, J_1 = 12.0 Hz, J_2 = 2.8 Hz, 1H), 4.16-4.05 (m, 2H), 4.01-3.94 (m, 2H), 2.66 (dd, J_1 = 13.6 Hz, J_2 = 12.4 Hz, 1H), 2.63 (s, 3H), 2.13 (s, 3H), 1.70 (dd, J_1 = 13.6 Hz, J_2 = 2.8 Hz, 1H), 1.35 (td, J_1 = 7.2 Hz, J_2 = 1.2 Hz, 3H), 1.25 (td, J_1 =

7.2 Hz, J_2 = 1.2 Hz, 3H); ^{13}C NMR (100 MHz, $CDCl_3$): 178.20, 140.66, 139.95, 138.33 (d, J = 6.9 Hz), 133.88 (d, J = 9.6 Hz), 128.62, 128.53, 128.32, 128.25, 128.05, 127.77, 126.91, 126.83, 126.50, 114.47, 74.90, 67.83, 64.54 (d, J = 1.8 Hz), 64.48 (d, J = 1.5 Hz), 56.67 (d, J = 5.5 Hz), 38.76 (d, J = 1.9 Hz), 38.13, 16.09 (d, J = 7.3 Hz), 15.97 (d, J = 6.8 Hz), 10.06; ^{31}P NMR (162 MHz, $CDCl_3$): -6.85; IR (KBr): 2983, 1739, 1466, 1314, 1295, 1118, 1056, 1028 cm^{-1}; MS (ESI): 555.1 $[M+H]^+$; HRMS (DART POS): Exact mass calcd for $C_{29}H_{33}N_2O_5P^{35}Cl$ $[M+H]^+$: 555.1810, Found: 555.1807.

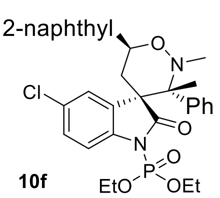

10f

Product **10f** was obtained in 83% yield as a white solid (m.p. 72-75 ºC); NMR analysis showed the dr value was 10:1. HPLC analysis (Chiralcel IF, ^{i}PrOH/hexane = 10/90, 1.0 mL/min, 230 nm; t_r (minor) = 15.07 min, t_r (major) = 19.92 min) gave the isomeric composition of the product: 94% ee, $[\alpha]^{25}_D$ = +18.0 (c = 1.0, $CHCl_3$); ^{1}H NMR (400 MHz, $CDCl_3$): 8.60 (s, 1H), 7.91-7.83 (m, 4H), 7.61 (dd, J_1 = 8.8 Hz, J_2 = 2.0 Hz, 1H), 7.50-7.48 (m, 2H), 7.44 (d, J = 8.4 Hz, 1H), 7.16 (dd, J_1 = 8.8 Hz, J_2 = 2.4 Hz, 1H), 7.13 (s, br, 2H), 7.01-7.00 (m, 3H), 5.75 (dd, J_1 = 12.4 Hz, J_2 = 2.8 Hz, 1H), 4.17-4.06 (m, 2H), 4.02-3.94 (m, 2H), 2.77 (dd, J_1 = 13.6 Hz, J_2 = 12.4 Hz, 1H), 2.68 (s, 3H), 2.19 (s, 3H), 1.78 (dd, J_1 = 13.6 Hz, J_2 = 2.8 Hz, 1H), 1.36 (td, J_1 = 7.2 Hz, J_2 = 1.2 Hz, 3H), 1.24 (td, J_1 = 7.2 Hz, J_2 = 1.2 Hz, 3H); ^{13}C NMR (100 MHz, $CDCl_3$): 178.24, 140.66, 138.36 (d, J = 6.9 Hz), 137.28, 133.89 (d, J = 9.7 Hz), 133.30, 133.29, 128.57, 128.42, 128.30, 128.09, 128.06, 127.77, 127.70, 126.94, 126.86, 126.27, 126.19, 125.56, 124.36, 114.52, 75.04, 67.90, 64.56, 64.50, 56.72 (d, J = 5.6 Hz), 38.82 (d, J = 2.2 Hz), 38.09, 16.10 (d, J = 7.3 Hz), 15.97 (d, J = 6.8 Hz), 10.16; ^{31}P NMR (162 MHz, $CDCl_3$): -6.85; IR (KBr): 2982, 1739, 1467, 1297, 1278, 1198, 1159, 1028 cm^{-1}; MS (ESI): 605.1 $[M+H]^+$; HRMS (DART POS): Exact mass calcd for $C_{33}H_{35}N_2O_5P^{35}Cl$ $[M+H]^+$: 605.1967, Found: 605.1965.

The elaboration of spiropropyl oxindoles 2a to compounds 6, 7 and 8

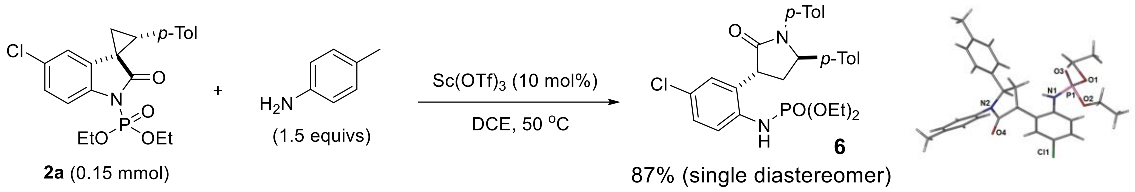

To a Schlenk tube was sequentially added Sc(OTf)3 (7.4 mg, 0.015 mmol, 10 mol %), oxindole **2a** (0.15 mmol), *p*-toluidine (0.225 mmol) and anhydrous DCE (1.5 mL). The resulting solution was stirred at 50 °C for 3 h, till the full consumption of **2a** by TLC analysis, and then purified by silica gel column chromatography to afford product **6** in 87% yield as a white solid (m.p. 156-158 °C), using Petroleum ether/EtOAc (1/1, v/v) as the eluent; NMR analysis showed that **6** was obtained as single diastereomer. ^{1}H NMR (400 MHz, $CDCl_3$): 7.41-7.36 (m, 2H), 7.27-7.24 (m, 2H), 7.21-7.13 (m, 5H), 7.03 (d, J = 8.8 Hz, 2H), 5.34 (dd, J_1 = 7.2 Hz, J_2 = 4.2 Hz, 1H), 4.32 (dd, J_1 = 8.8 Hz, J_2 = 6.8 Hz, 1H), 4.19-4.04 (m, 4H), 2.98 (m, 1H), 2.46-2.39 (m, 1H), 2.31 (s, 3H), 2.22 (s, 3H), 1.34 (t, J = 6.8 Hz, 3H), 1.28 (t, J = 6.8 Hz, 3H); ^{13}C NMR (100 MHz, $CDCl_3$): 174.54, 138.11 (d, J = 1.4 Hz), 137.88, 136.97, 135.34, 134.92, 130.41 (d, J = 10.2 Hz), 129.75, 129.31, 128.36, 128.15, 126.19, 126.08, 123.35 (d, J = 2.0 Hz), 122.41, 62.99 (d, J = 2.9 Hz), 62.94 (d, J = 2.9 Hz), 62.47, 43.21, 34.78, 21.07, 20.85, 16.22 (d, J = 6.9 Hz), 16.11 (d, J = 7.1 Hz); ^{31}P NMR (162 MHz, $CDCl_3$): 2.39; IR (ATR): 2925, 1690, 1513, 1495, 1384, 1242, 1021 cm^{-1}; MS (ESI): 527.08 [M+H]$^+$; HRMS (EI): Exact mass calcd for $C_{28}H_{32}N_2O_4P^{35}Cl$ [M]$^+$: 526.1788, Found: 526.1794.

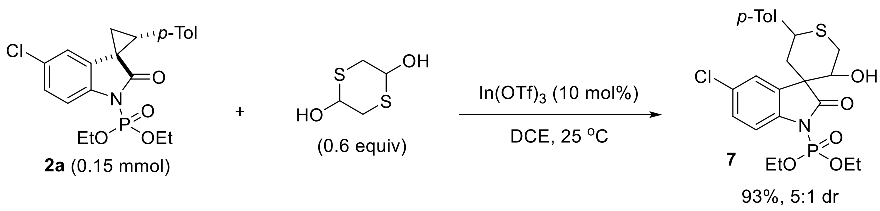

To a Schlenk tube was sequentially added In(OTf)3 (8.4 mg, 0.015 mmol, 10 mol %), oxindole **2a** (0.15 mmol), 1,4-dithiane-2,5-diol (0.09 mmol) and anhydrous DCE (1.5 mL). After the resulting solution was stirred at 25 °C for 40 min, DCE was removed under reduced pressure. The residue was dissolved in CH_2Cl_2, rapidly passed through a glass funnel with a thin layer (5 mm) of silica gel (100 mesh), eluted with CH_2Cl_2, and concentrated under reduced pressure. NMR analysis showed the dr value was 5:1, and silica gel column chromatography gave product **7** in 93% yield as a white solid (m.p. 58-60 °C), using Petroleum ether/EtOAc (1/1, v/v) as the eluent; ^{1}H NMR (400 MHz, $CDCl_3$):

7.90 (d, J = 8.8 Hz, 1H), 7.69 (s, 1H), 7.38 (dd, J_1 = 8.8 Hz, J_2 = 2.4 Hz, 1H), 7.27 (d, J = 8.4 Hz, 2H), 7.12 (d, J = 8.0 Hz, 2H), 4.52-4.48 (m, 1H), 4.36 (dd, J_1 = 13.2 Hz, J_2 = 2.8 Hz, 1H), 4.26-4.16 (m, 4H), 3.37 (dd, J_1 = 13.6 Hz, J_2 = 11.6 Hz, 1H), 2.72 (dd, J_1 = 19.6 Hz, J_2 = 4.8 Hz, 1H), 2.71 (t, J = 13.2 Hz, 1H), 2.31 (s, 3H), 2.14 (d, J = 6.8 Hz, 1H), 2.06 (dd, J_1 = 13.6 Hz, J_2 = 2.8 Hz, 1H), 1.35-1.30 (m, 6H); ^{13}C NMR (100 MHz, $CDCl_3$): 179.65, 140.46 (d, J = 7.2 Hz), 137.96, 136.22, 130.50 (d, J = 9.3 Hz), 129.47, 129.14, 129.10, 127.46, 125.62, 115.88, 72.55, 64.95 (d, J = 5.9 Hz), 64.75 (d, J = 5.7 Hz), 55.57 (d, J = 5.5 Hz), 43.19, 39.59, 31.42, 21.10, 16.04 (d, J = 7.0 Hz), 15.96 (d, J = 7.8 Hz); ^{31}P NMR (162 MHz, $CDCl_3$): -6.99; IR (ATR): 2950, 2917, 1747, 1468, 1375, 1286, 1024 cm^{-1}; HRMS (ESI): Exact mass calcd for $C_{23}H_{28}NO_5PS^{35}Cl$ [M+H]$^+$: 496.1109, Found: 496.1107.

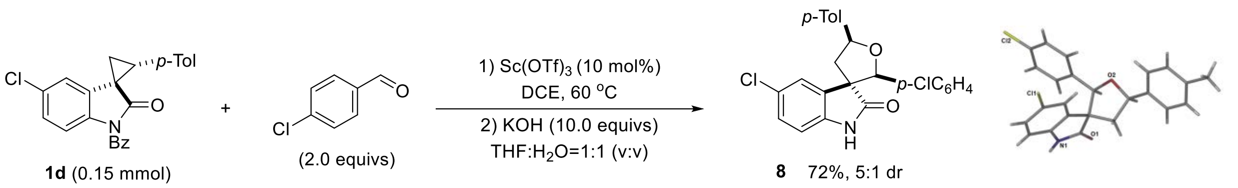

To a Schlenk tube was sequentially added $Sc(OTf)_3$ (7.4 mg, 0.015 mmol, 10 mol %), oxindole **1d** (0.15 mmol), 4-chlorobenzaldehyde (0.30 mmol) and anhydrous DCE (1.5 mL). After the resulting solution was stirred at 60 ºC for 25 min, DCE was removed under reduced pressure. Then THF (1 ml), water (1 ml) and KOH (56.0 mg, 1.0 equiv) was added. The reaction was stirred at room temperature for 30 min, then stopped and extracted with ethyl acetate (3 × 5 mL). The organic layer was dried over anhydrous Na_2SO_4, concentrated in vacuum. NMR analysis showed the dr value was 5:1, and silica gel column chromatography gave product **8** in 72% yield (using Petroleum ether/EtOAc (10/1, v/v) as the eluent) as a white solid (m.p. 239-241 ºC); ^{1}H NMR (400 MHz, $CDCl_3$): 8.18 (s, 1H), 7.46 (d, J = 8.0 Hz, 2H), 7.28 (d, J = 7.6 Hz, 2H), 7.16-7.11 (m, 4H), 6.99 (dd, J_1 = 8.4 Hz, J_2 = 2.0 Hz, 1H), 6.93 (d, J = 2.4 Hz, 1H), 6.62 (d, J = 8.0 Hz, 1H), 5.44 (t, J = 8.0 Hz, 1H), 5.36 (s, 1H), 3.34 (dd, J_1 = 13.2 Hz, J_2 = 8.0 Hz, 1H), 2.41 (s, 3H), 2.33 (dd, J_1 = 13.2 Hz, J_2 = 8.8 Hz, 1H); ^{13}C NMR (100 MHz, $CDCl_3$): 179.69, 138.06, 137.81, 137.32, 134.98, 133.60, 133.48, 129.44, 128.13, 127.92, 127.81, 126.74, 125.74, 125.39, 110.36, 87.42, 80.01, 60.28, 46.44, 21.25; IR (ATR): 3026, 1710, 1617, 1474, 1317, 1173, 1015 cm^{-1}; MS (ESI): 424.1 [M+H]$^+$; HRMS (DART POS): Exact mass calcd for $C_{24}H_{20}NO_2Cl_2$ [M+H]$^+$: 424.0866, Found: 424.0865.

Deprotection of product 5a and 5o

To a 10 mL flask were added KOH (56.0 mg, 10 equivs) and **5** (0.1 mmol), followed by EtOH (2.0 mL). The mixture was stirred vigorously for 15 min. After the reaction was finished, the ethanol was removed under reduced pressure, then purified by silica gel column chromatography to afford **11**, using DCM/EtOAc (20/1, v/v) as the eluent.

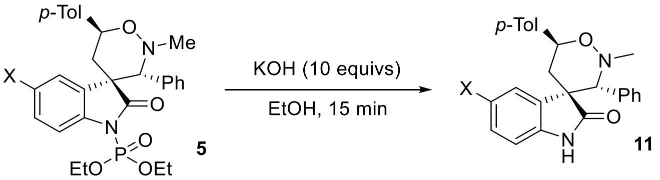

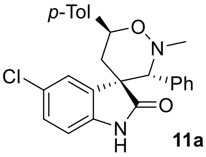

11a

Product **11a** was obtained from **5a** (96% ee) in 85% yield as a white solid (m.p. 256-258 °C); with 97% ee by HPLC analysis (Chiralcel IE, ^{i}PrOH/hexane = 10/90, 1.0 mL/min, 230 nm; t_r (major) = 6.44 min, t_r (minor) = 7.93 min), $[\alpha]^{25}_{D}$ = -112.8 (c = 1.0, $CHCl_3$); ^{1}H NMR (400 MHz, $CDCl_3$): 8.38 (s, br, 1H), 8.25 (d, J = 2.0 Hz, 1H), 7.34 (d, J = 8.4 Hz, 2H), 7.20-7.17 (m, 4H), 7.06 (s, br, 2H), 6.92 (s, 1H), 6.71 (s, 1H), 6.60 (d, J = 8.4 Hz, 1H), 5.50 (dd, J_1 = 12.0 Hz, J_2 = 2.0 Hz, 1H), 4.12 (s, 1H), 2.54 (s, 3H), 2.53-2.46 (m, 1H), 2.35 (s, 3H), 1.85 (dd, J_1 = 13.6 Hz, J_2 = 2.4 Hz, 1H); ^{13}C NMR (100 MHz, $CDCl_3$): 178.64, 138.83, 138.10, 136.78, 135.62, 133.19, 130.08, 129.23, 128.10, 127.82, 127.44, 127.36, 126.64, 110.49, 75.95, 75.04, 54.54, 44.78, 39.96, 21.21; IR (ATR): 3296, 2949, 2918, 2848, 1719, 1618, 1469, 1309, 1166, 1069, 1025 cm^{-1}; MS (EI): 418, 420 (M^+, 4, 1), 136 (100), 283 (45), 118 (28), 119 (17), 285 (16), 91 (15), 77 (11); HRMS (EI): Exact mass calcd for $C_{25}H_{23}N_2O_2{}^{35}Cl$ $[M]^+$: 418.1448, Found: 418.1452.

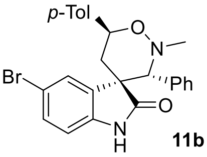

11b

Product **11b** was obtained from **5o** (96% ee) in 82% yield as a white solid (m.p. 252-256 °C); with 97% ee by HPLC analysis (Chiralcel IE, ^{i}PrOH/hexane = 10/90, 1.0 mL/min, 230 nm; t_r (major) = 6.21 min, t_r (minor) = 7.77 min), $[\alpha]^{25}_{D}$ = -172.2 (c = 1.0, $CHCl_3$); ^{1}H NMR (400 MHz, $CDCl_3$): 8.39 (d, J = 2.0 Hz, 1H), 8.02 (s, 1H), 7.36-7.32 (m, 3H), 7.19-7.13 (m, 3H), 7.07 (s, br, 2H), 6.93 (s, br, 1H), 6.70 (s, br, 1H), 6.54 (d, J = 8.0 Hz, 1H), 5.49 (dd, J_1 = 12.0 Hz, J_2 = 2.4 Hz, 1H), 4.12 (s, 1H), 2.53 (s, 3H), 2.50-2.47 (m, 1H), 2.35 (s, 3H), 1.86 (dd, J_1 = 13.6 Hz, J_2 = 2.4 Hz, 1H); ^{13}C NMR (100 MHz, $CDCl_3$): 178.55, 139.28, 138.11, 136.72, 135.57, 133.57, 131.01, 130.11, 129.23, 127.83, 127.48, 126.64, 114.71, 111.02, 75.92, 75.02, 54.48, 44.79, 39.90, 21.22; IR (ATR): 3213, 3026, 2916, 2856, 1708, 1614, 1468, 1310, 1076 cm^{-1}; MS (EI): 462, 464 (M^+, 2, 2), 136 (100), 329 (17), 327 (17), 118 (15), 91 (11), 137 (10), 43 (10); HRMS (EI): Exact mass calcd for $C_{25}H_{23}N_2O_2{}^{79}Br$ $[M]^+$: 462.0943, Found: 492.0941.

Determine the relative and absolute configuration of 5a, 10a and recovered 2b

Determine the relative and absolute configuration of adduct 5a

To a stirred solution of **11a** (62.7 mg, 0.15 mmol) in 1 mL of THF, NaH (12 mg, 60% in mineral oil, 2.0 equiv) was added in one portion and stirred at room temperature for 10 minutes. Then tosyl chloride (57 mg, 0.30 mmol) was added and stirred for another 15 minutes. The reaction mixture was quenched with saturated NH_4Cl and extracted with ethyl acetate (3 × 5 mL). The organic layer was dried by Na_2SO_4, concentrated in vacuum, then purified by silica gel column chromatography to afford enantiomer enriched **4e** in 93% yield as a white solid (m.p. 210-215 ^{o}C), using Petroleum ether/EtOAc (5/1, v/v) as the eluent. HPLC analysis (Chiralcel IE, ^{i}PrOH/hexane = 20/80, 1.0 mL/min, 230 nm; t_r (major) = 14.01 min, t_r (minor) = 16.47 min) gave the isomeric composition of the thus obtained enantoenriched compound **4e**: 97% ee. After recrystallization, its enantiomeric excess could be enriched to >99%. $[\alpha]^{25}_{D}$ = -67.4 (c = 1.0, $CHCl_3$); ^{1}H NMR (400 MHz, $CDCl_3$): 8.32 (d, J = 2.4 Hz, 1H), 7.68-7.65 (m, 3H), 7.31-7.29 (m, 3H), 7.22 (d, J = 8.4 Hz, 2H), 7.17 (d, J = 7.6 Hz, 2H), 6.99-6.95 (m, 1H), 6.83-6.74 (m, 3H), 6.54 (s, br, 1H), 5.43 (dd, J_1 = 12.0 Hz, J_2 = 2.4 Hz, 1H), 4.06 (s, 1H), 2.48-2.42 (m, 1H), 2.46 (s, 3H), 2.44 (s, 3H), 2.34 (s, 3H), 1.83 (dd, J_1 = 13.2 Hz, J_2 = 2.4 Hz, 1H); ^{13}C NMR (100 MHz, $CDCl_3$): 174.88, 145.47, 138.27, 137.12, 136.26, 134.76, 134.74, 131.66, 129.72, 129.26, 128.75, 128.08, 127.76, 127.68, 127.47, 127.41, 127.28, 126.54, 114.28, 75.46, 75.06, 53.86, 44.55, 40.56, 21.76, 21.20; IR (ATR): 2973, 2920, 1753, 1458, 1378, 1235, 1176, 1089 cm^{-1}; MS (EI): 572, 574 (M^{+}, 4, 2), 136 (100), 91 (71), 118 (66), 282 (61), 43 (55), 119 (45), 83 (41); HRMS (EI): Exact mass calcd for $C_{32}H_{29}N_2O_4S^{35}Cl$ [M]$^{+}$: 572.1537, Found: 572.1544.

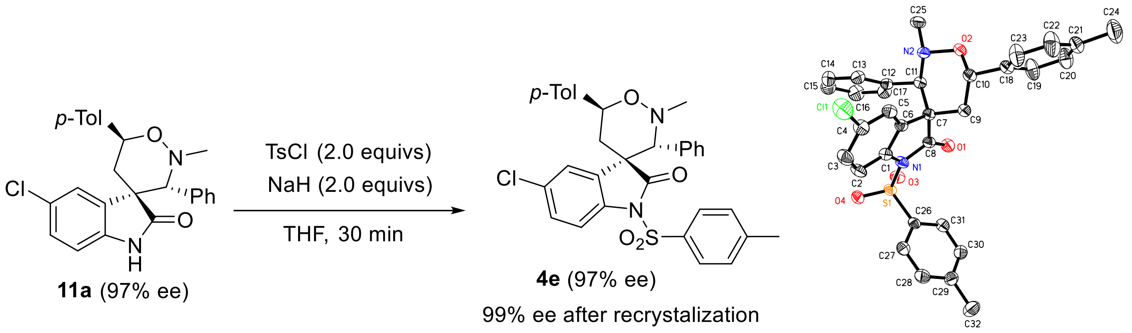

Supplementary Figure 1. Determine the relative and absolute configuration of adduct **5a**

Determine the relative and absolute configuration of adduct 10a

Compound **12a** was obtained from **10a** by the following two steps. The removal of the *N*-protecting group of **10a** (95% ee) was conducted according to the procedure described in section 7 (Page 31) to give unprotected **II**, which was protected by *N*-p-tolylsulfonyl group by the same procedure for the synthesis of enantoenriched compound **4e** described in Page 32. Compound **12a** was obtained in 73% yield by two steps as a white solid (m.p. 172-173 ^{o}C). HPLC analysis (Chiralcel IE, ^{i}PrOH/hexane = 10/90, 1.0 mL/min, 230 nm; t_r (minor) = 14.20 min, t_r (major) = 15.37 min) gave the isomeric composition of the thus obtained **12a**: 96% ee. After recrystallization, its enantiomeric excess could be enriched to >99%. $[\alpha]^{25}_{D}$ = +147.8 (c = 1.0, $CHCl_3$); ^{1}H NMR (400 MHz, $CDCl_3$): 8.47 (d, J = 2.4 Hz, 1H), 7.82 (d, J = 8.4 Hz, 2H), 7.49 (d, J = 8.8 Hz, 1H), 7.31-7.28 (m, 4H), 7.18-7.15 (m, 3H), 6.98-6.85 (m, 5H), 5.44 (dd, J_1 = 12.4 Hz, J_2 = 2.8 Hz, 1H), 2.61 (s, 3H), 2.54 (dd, J_1 = 13.6 Hz, J_2 = 12.4 Hz, 1H), 2.43 (s, 3H), 2.34 (s, 3H), 2.01 (s, 3H), 1.51 (dd, J_1 = 13.6 Hz, J_2 = 2.8 Hz, 1H); ^{13}C NMR (100 MHz, $CDCl_3$): 174.88, 145.66, 139.92, 138.23, 136.72, 136.37, 135.05, 133.11, 129.70, 129.27, 129.10, 128.58, 128.23, 128.02, 127.40, 126.99, 126.78, 126.50, 113.61, 74.80, 67.82, 56.19, 38.88, 37.26, 21.75, 21.20, 10.04; IR (KBr): 2921, 2851, 1749, 1460, 1378, 1178, 1090 cm^{-1}; MS (ESI): 587.0 [M+H]$^{+}$; HRMS (DART POS): Exact mass calcd for $C_{33}H_{32}N_2O_4S^{35}Cl$ [M+H]$^{+}$: 587.1766, Found: 587.1766.

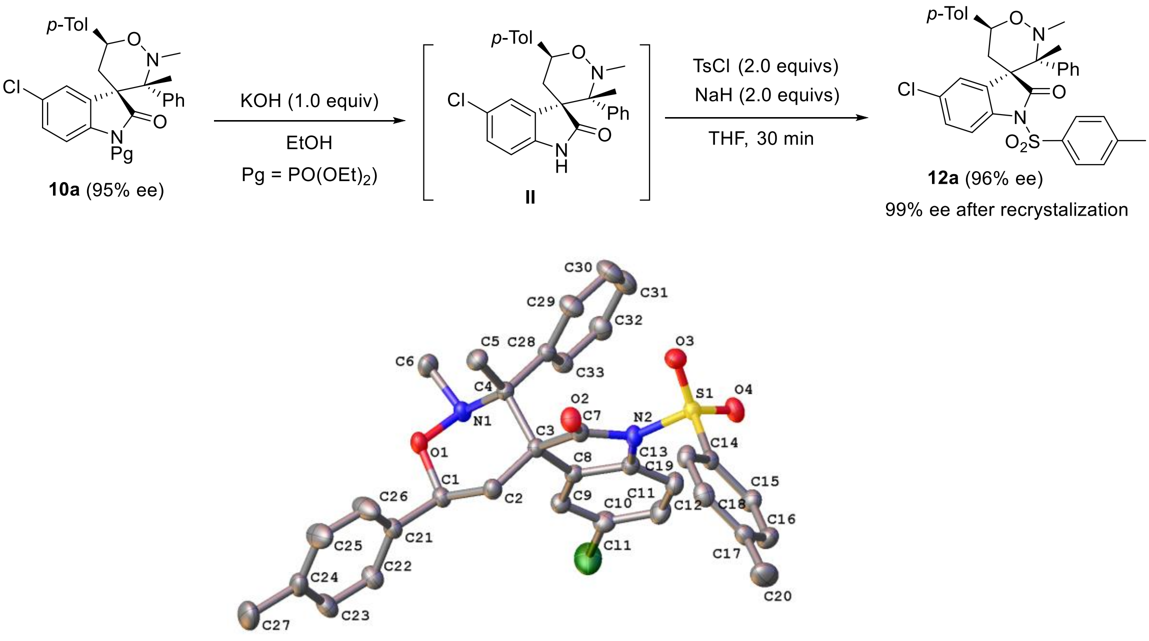

Supplementary Figure 2. Determine the relative and absolute configuration of adduct **10a**

Determine the relative and absolute configuration of recovered 2b

The absolute configuration of recovered spirocyclopropyl oxindole **2b** was determined to be (1*R*, 2*S*) by comparing the optical rotation value of **2b**'.

Unprotected spirocyclopropyl oxindole (1*S*, 2*R*)-**Ib** was prepared according to literature report using spiroketal bisphosphine (SKP) derived chiral digold complex (*S*,*S*,*S*)-**L**($AuCl)_2$ activated by $AgBF_4$.[3] The conversion of (1*S*, 2*R*)-**Ib** to *N*-diethoxyphosphoryl spirocyclopropyl oxindoles **2b'** was using the procedure described in supplementary methods. HPLC analysis (Chiralcel IC, ^{i}PrOH/hexane = 20/80, 1.0 mL/min, 230 nm; t_r (minor) = 12.63 min, t_r (major) = 16.43 min) gave the isomeric composition of the thus obtained enantoenriched compound **2b'**: 90% ee. $[\alpha]^{25}_D$ = +148.1 (c = 1.0, $CHCl_3$);

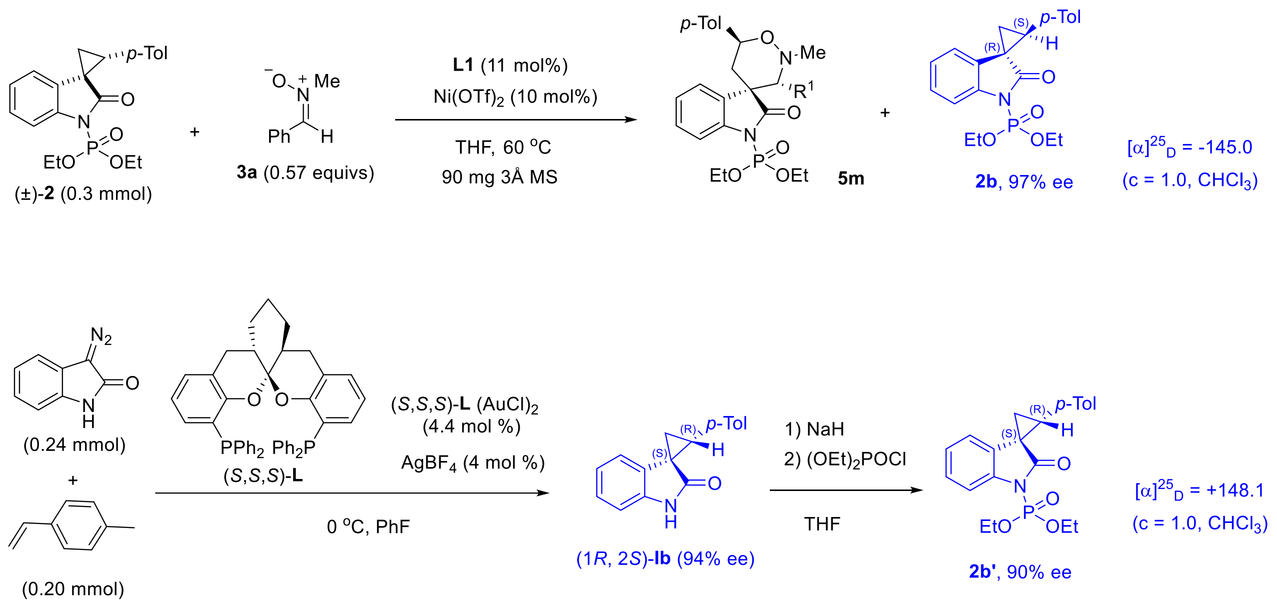

Supplementary Figure 3. Determine the relative and absolute configuration of recovered **2b**

By comparing HPLC retention time and the optical rotation value of compound **2b'** with those of compound **2b** obtained via kinetic resolution, the configuration of the stereogenic center of the major diasteromer of product **2b**, at C3 position of oxindole was assigned to be *R* and that at the remaining stereocenter was *S*.

.

Supplementary Discussion

Condition optimization

We first examined the performance of different metal triflates in the [3+3] cycloaddition of **1e** and nitrone **3a**. All reactions were conducted in CH_2ClCH_2Cl (DCE) at 50 °C, in the presence of 10 mol% Lewis acid catalyst. Of all the triflates we screened, $Zn(OTf)_2$, $Ba(OTf)_2$, $Cu(OTf)_2$, $In(OTf)_2$ and $Fe(OTf)_3$ failed to mediate this reaction, even the reaction was run for 24 h. $Ni(OTf)_2$ catalyzed the reaction to finish within 48 h, giving product **4e** in 94% NMR yield and 11:1 dr (entry 1, Supplementary Table 1). $Mg(OTf)_2$ and $La(OTf)_2$ seemed to be more active, as the reaction could finish in 24 h, giving **4e** in comparable yield and diastereoselectivity (entries 2-3). Several other triflates such as $Sc(OTf)_3$ and $Yb(OTf)_3$ also catalyzed the reaction well, albeit in moderate diastereoselectivity (entries 4-10).

The use of *N*-diethoxyphosphoryl spirocyclopropyl oxindoles **2a** resulted in obviously improved reactivity and diastereoselectivity (entries 11-15). Of the several triflates we examined, $Ni(OTf)_2$ turned out to be the best catalyst in terms of reactivity and diastereoselectivity (entry 11).

Supplementary Table 1. Evaluation of different metal triflates.

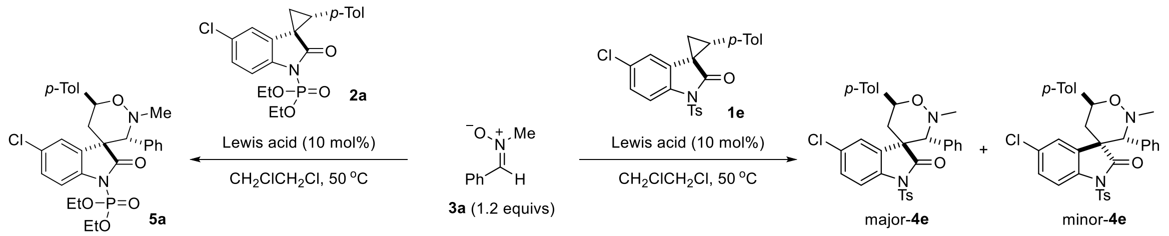

Entry[a]	Cyclopropane	Lewis acid	Adduct	Time (h)	Dr[b]	Yield (%)[c]
1	**1e**	$Ni(OTf)_2$	**4e**	48	11 : 1	94
2	**1e**	$Mg(OTf)_2$	**4e**	24	10 : 1	94
3	**1e**	$La(OTf)_2$	**4e**	24	10 : 1	> 95
4	**1e**	$Sc(OTf)_3$	**4e**	24	4 : 1	94
5	**1e**	$Yb(OTf)_3$	**4e**	24	3.5 : 1	> 95
6	**1e**	$Er(OTf)_3$	**4e**	24	3 : 1	> 95
7	**1e**	$Y(OTf)_3$	**4e**	24	3 : 1	> 95
8	**1e**	$Tm(OTf)_3$	**4e**	24	3 : 1	> 97
9	**1e**	$Sm(OTf)_3$	**4e**	24	3 : 1	> 95
10	**1e**	$Bi(OTf)_3$	**4e**	24	3 : 1	94
11[d]	**2a**	$Ni(OTf)_2$	**5a**	6	>20:1	>95
12[d]	**2a**	$Mg(OTf)_2$	**5a**	18	>20:1	>95
13[d]	**2a**	$La(OTf)_2$	**5a**	18	13:1	>95
14[d]	**2a**	$Er(OTf)_3$	**5a**	5	17:1	>95
15[d]	**2a**	$Yb(OTf)_3$	**5a**	5	15:1	>95

[a] 0.10 mmol scale in 1.0 mL of solvent. [b] Determined by ^{1}H NMR analysis of crude mixture. [c] NMR yield using mesitylene as the internal standard. [d] Contained about 5-10% of the corresponding deprotected pruducts.

The relative configuration of the major diastereomer of product major-**4e**, minor-**4e** and **5o** was assigned by X-ray analysis of the corresponding single crystals. For the full characterization of compound major-**4e** and **5o**, see the corresponding enantioenriched compound **4e** and **5o** in Supplementary Methods.

Irrespective of the *N*-protecting group, both major-**4e** and **5o** had the same relative configuration: the amide group of oxindole was *trans* to the C3 phenyl group, but bore a *cis* relationship with the C6 tolyl group. The relative configuration of all the other products **4a-d**, bearing different nitrogen substituents, was tentatively assigned to be the same by the ^{1}H NMR analysis.

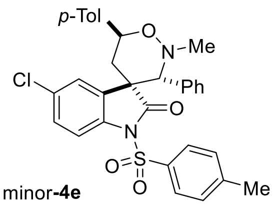

minor-**4e**

Minor-**4e** was obtained as a white solid (m.p. 209-210 ºC); ^{1}H NMR (400 MHz, $CDCl_3$): 7.84 (d, *J* = 8.0 Hz, 2H), 7.56 (d, *J* = 8.8 Hz, 1H), 7.37-7.30 (m, 5H), 7.21-7.16 (m, 3H), 7.06-6.98 (m, 2H), 6.87 (s, 1H), 6.68 (s, 1H), 6.48 (s, 1H), 5.80 (dd, J_1 = 11.2 Hz, J_2 = 2.4 Hz, 1H), 3.72 (s, 1H), 2.48 (s, 3H) , 2.45 (s, 3H) , 2.34 (s, 3H), 2.21-2.14 (m, 1H), 2.09-2.04 (m, 1H); ^{13}C NMR (100 MHz, $CDCl_3$): 174.81, 145.57, 138.05, 137.10, 136.54, 134.98, 134.36, 131.90, 130.09, 129.70, 129.24, 128.95, 128.51, 127.98, 126.57, 123.45, 114.48, 74.85, 52.05, 40.08, 21.79, 21.20; IR (ATR): 2922, 1753, 1466, 1388, 1307, 1177, 1072, 1048 cm^{-1}; HRMS (ESI): Exact mass calcd for $C_{32}H_{30}N_2O_4SCl$ $[M+H]^+$: 573.1615, Found: 573.1619.

Supplementary Table 2. Relative configuration identification of major-**4e**, minor-**4e** and **5o**

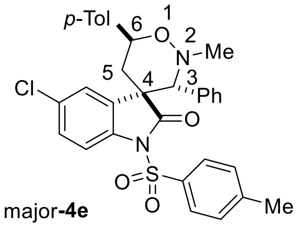 major-**4e**	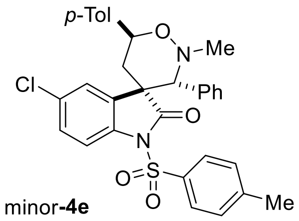 minor-**4e**	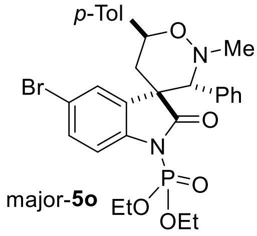 major-**5o**
X-ray analysis of major-**4e** 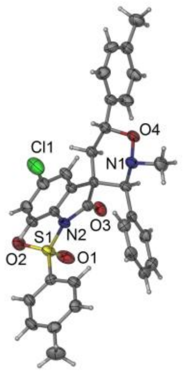	X-ray analysis of minor-**4e** 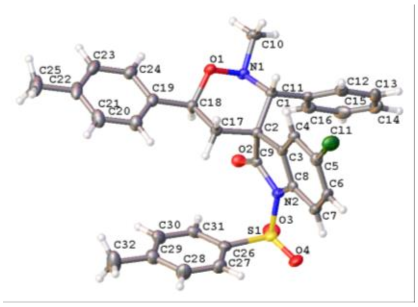	X-ray analysis of major-**5o** 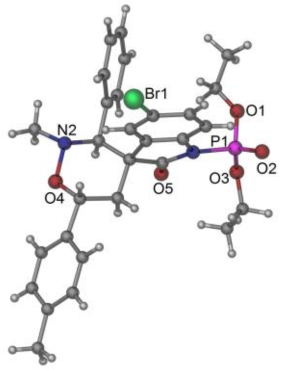

To develop a catalytic enantioselective version, we chose the [3+3] dipolar cycloaddition of **2a** and nitrone **3a** as the model reaction for the screening of different chiral ligands. All the reactions were carried out in DCE at 50 °C, in the presence of chiral catalyst prepared from 11 mol% of chiral ligand and 10 mol% $Ni(OTf)_2$. Of all the ligands we screened, axially chiral BINAP **L₂** and BINOL **L₃**, phosphoramide derived ligand **L₄** and **L₅**, and PYBOX **L₆** all gave poor results (entries 2-6). Bisoxazolines **L₁** and **L₇₋₁₀** afforded better results in terms of enantioselectivity (entries 7-10), and **L₁** was identified as the ligand of choice (entry 1).

Supplementary Table 3. The screening of different chiral ligands

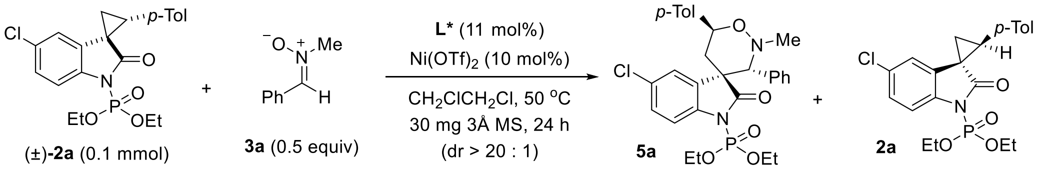

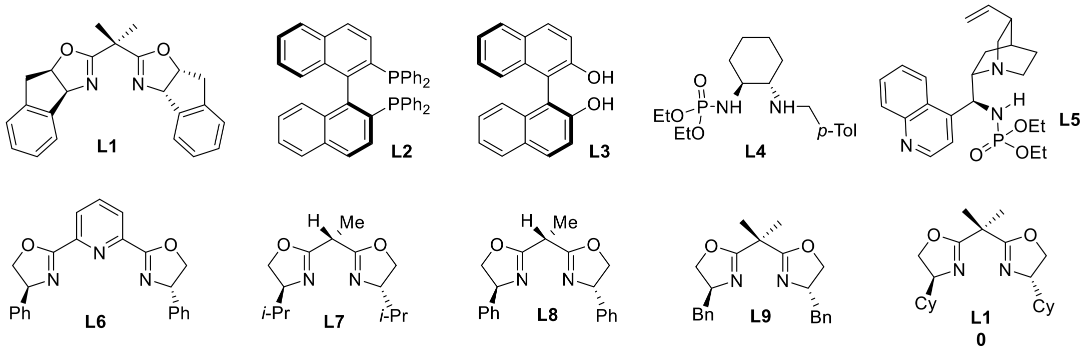

Entry	L*	Yield of **5a** (%)[a]	Ee of **5a** (%)[b]	Recovery of **2a** (%)[a]	Ee of **2a** (%)[b]
1	$\mathbf{L_1}$	41	92	40	78
2	$\mathbf{L_2}$	40	-8	39	-5
3[c]	$\mathbf{L_3}$	36	0	43	0
4	$\mathbf{L_4}$	38	2	37	10
5	$\mathbf{L_5}$	42	-3	38	2
6	$\mathbf{L_6}$	45	-16	41	-5
7	$\mathbf{L_7}$	41	43	40	35
8	$\mathbf{L_8}$	36	10	49	15
9	$\mathbf{L_9}$	34	41	50	42
10	$\mathbf{L_{10}}$	40	13	52	15

[a] Isolated yield. [b] Determined by HPLC analysis. [c] 10 : 1 dr.

Variable temperature ^{1}H NMR spectrum of 5g and 5h

The ^{1}H NMR spectra of compounds **5g** and **5h** collected at room temperature suggested the existence of two isomeric products. It was also found that the two isomers of **5g** and **5h** could not be separated by conventional techniques (TLC, HPLC). First, to obviate the possibility that this phenomenon resulted from the conformational change of *N*-diethoxyphosphoryl, we compared the ^{1}H NMR spectra of **5g** with the corresponding unprotected product **5g-II** (Supplementary Figure 4). The two isomeric products of products **5g** had a ratio of nearly 1.6:1 judged by the peaks at δ 8.32-8.38 ppm, and those of and **5g-II** had a similar ratio by analyzing the peaks of 8.23-8.28 ppm. This suggested that the occurrence of the two isomers was not caused by *N*-diethoxyphosphoryl group.

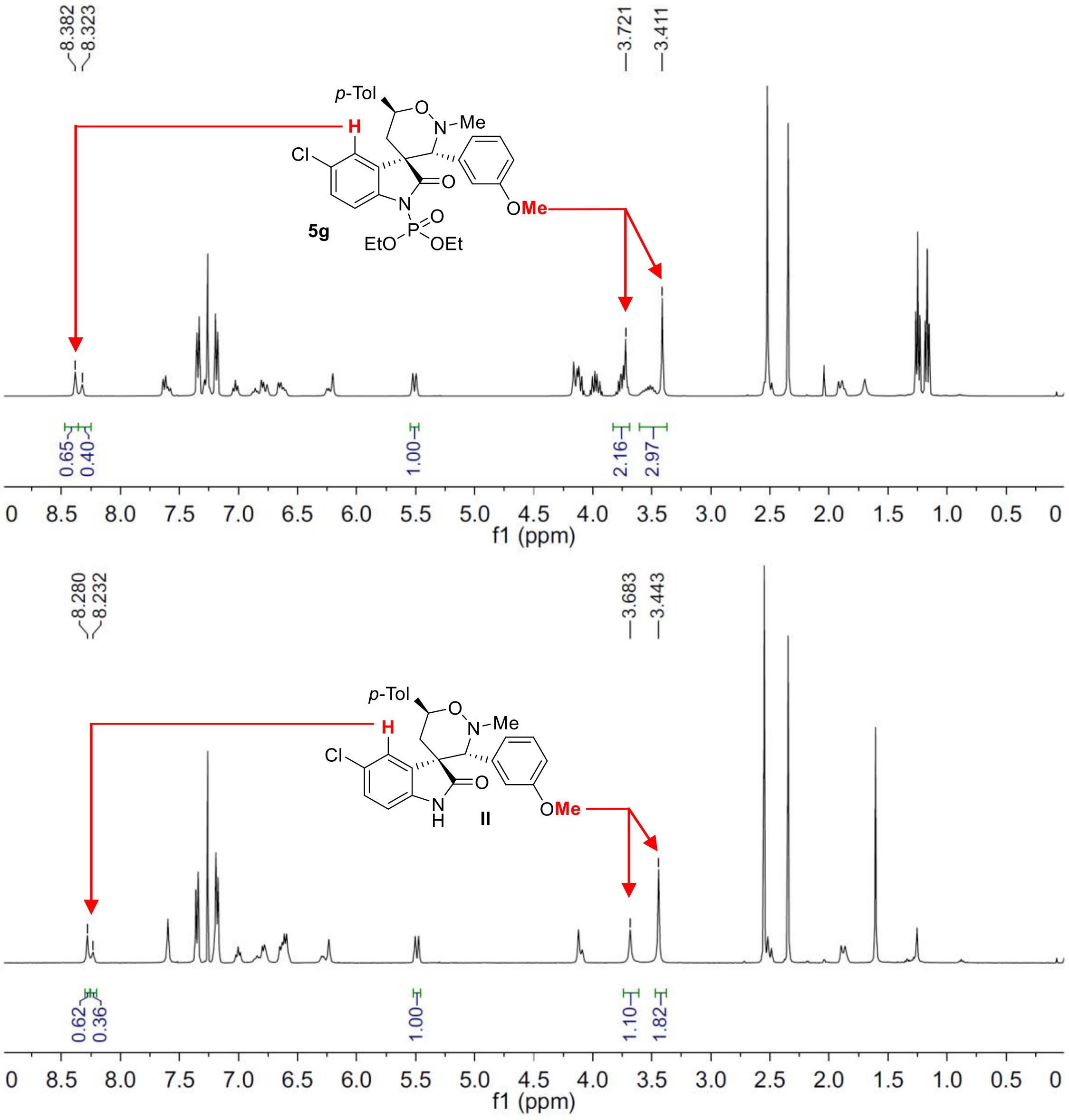

Supplementary Figure 4. The ^{1}H NMR spectra of compound **5g** and its unprotected analogue **5g-II**.

Based on the above results, we suspected the possibility of interconversion of these two isomers, so we tried variable temperature (VT) ^{1}H NMR experiments analysis.

Measurement of **5g** dissolved in DMSO-d_6 on VT ^{1}H NMR at higher temperature was shown in Supplementary Figure 5. The two set of separated signals (δ = 8.35 and 8.30) at 298 K got close at 313 K, merged into a broad singlet peak at 323 K. Further increasing the temperature, the broad peaks (δ = 8.32 at 323 K, δ = 8.31 at 353 K) became a sharp peak at 8.31 at 373 K. Meanwhile, another two separated signals (δ = 7.12 and δ = 6.91, at 298 K) converged into one broad peak at 353 K. Notably, at 373K, all signals changed into regular peaks. Therefore, it is believed that the presence of two sets of signals stems from the rotation of the *m*-methoxyphenyl group.

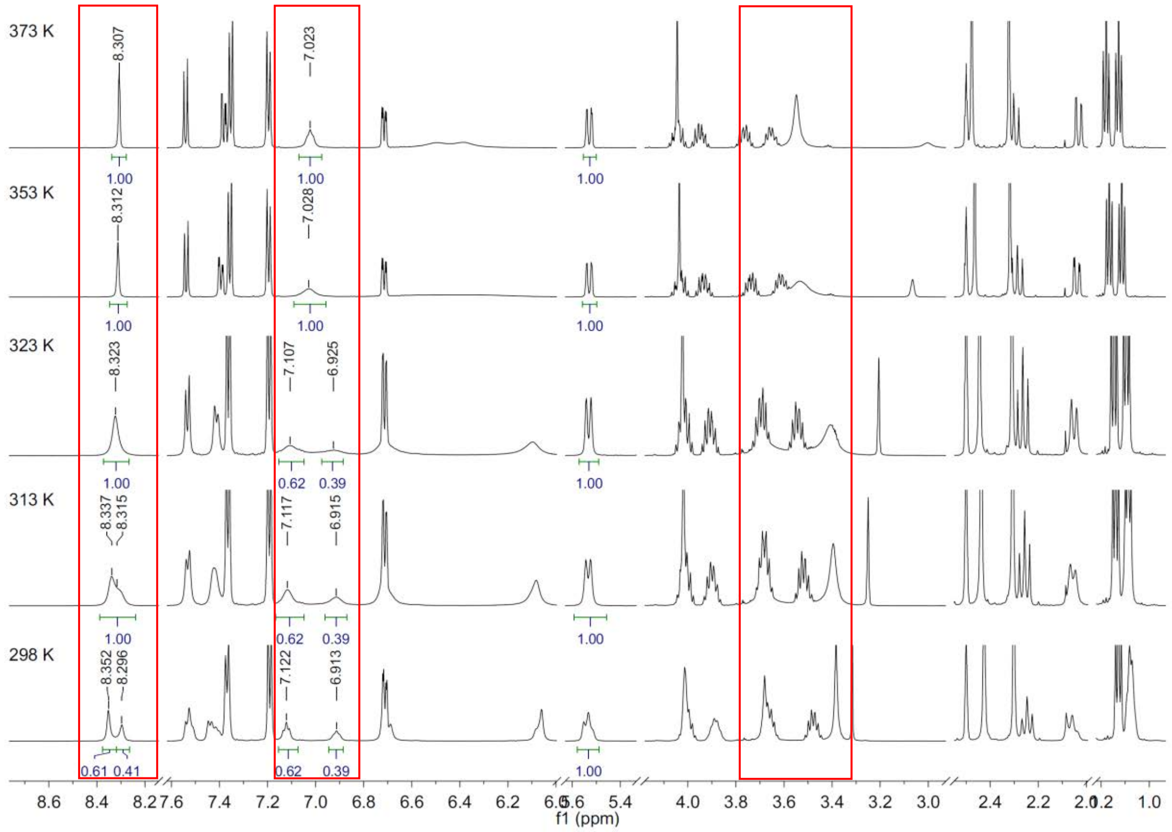

Supplementary Figure 5. Variable temperature ^{1}H NMR experiment analysis of compound **5g**.

The ^{1}H NMR spectra of compound **5h** was showed in Supplementary Figure 6. The two isomeric products was estimated in a ratio of nearly 3:1. VT ^{1}H NMR of compound **5h** showed that the two set of separated signals (δ= 8.44 and 8.41) at 298 K converged into one broad peak at 313K, which became sharper with temperature increasing and a sharp peak at 8.40 at 373 K. The two signals at δ = 0.88 and 0.62 became closer as the temperature went up and finally changed into a broad peak at 373 K (Supplementary Figure 7). It is believed that the presence of two sets of signals stems from the rotation of the 2-naphthyl group.

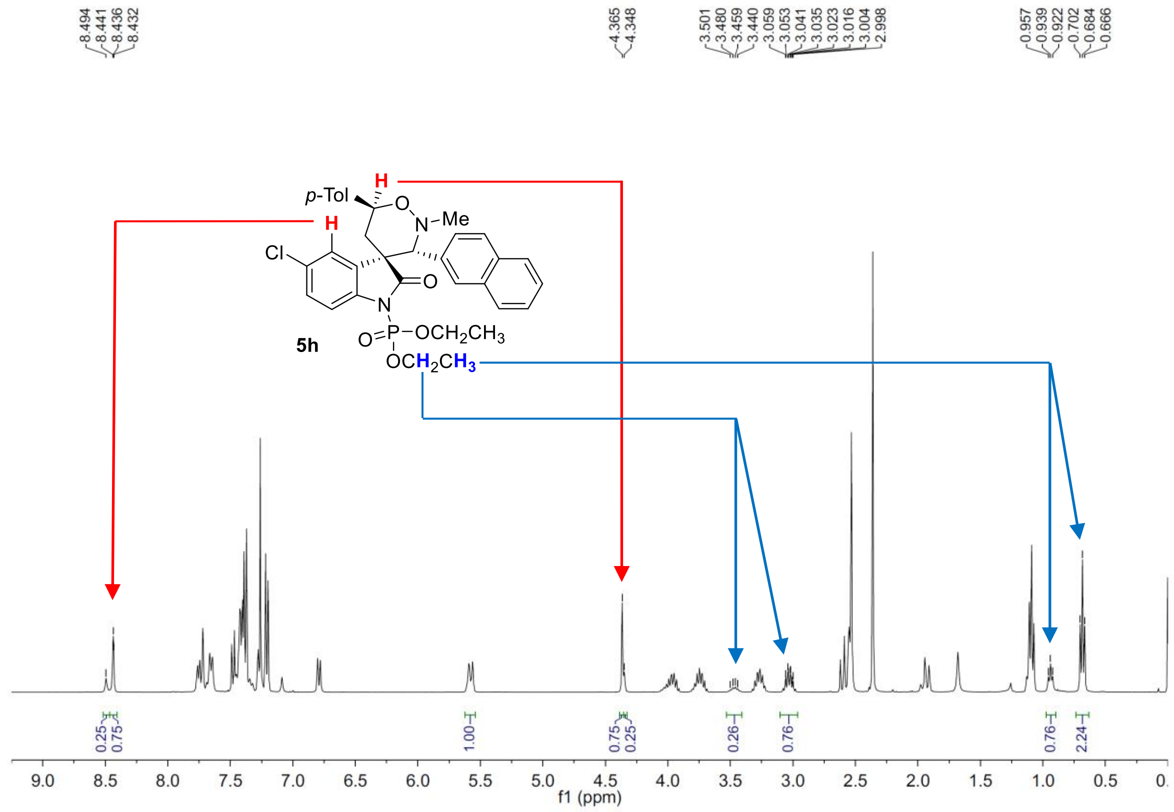

Supplementary Figure 6. The ^{1}H NMR spectra of compound **5h**.

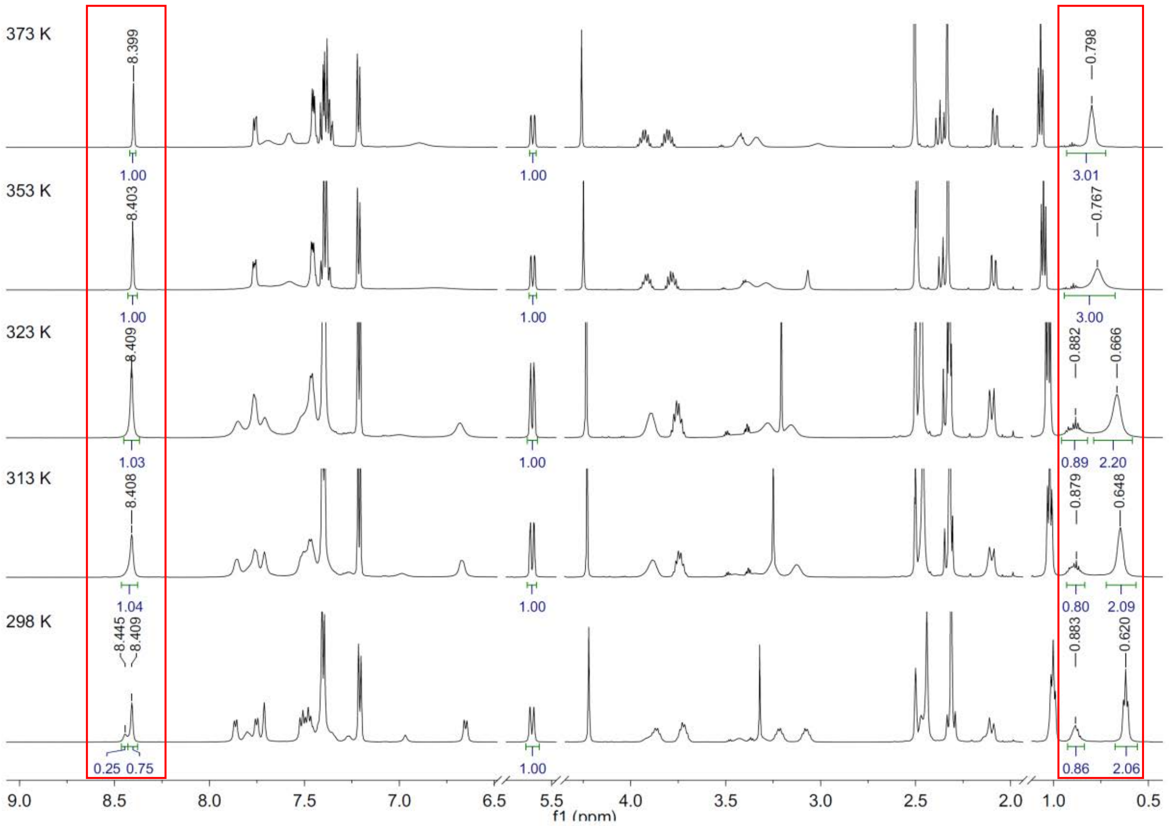

Supplementary Figure 7. Variable temperature ^{1}H NMR experiment analysis of compound **5h**.

Supplementary Tables

Tables for Single-Crystal X-ray Crystallography of major-4e

Supplementary Table 4. Crystal data and structure refinement for major-**4e**.

Identification code	major-**4e**	
Empirical formula	$C_{32}H_{29}ClN_2O_4S$	
Formula weight	573.08	
Temperature	296(2) K	
Wavelength	0.71073 Å	
Crystal system, space group	Monoclinic, C2/c	
Unit cell dimensions	*a* = 33.9594(19) Å	alpha = 90 deg.
	b = 8.3689(5) Å	beta = 96.408(2) deg.
	c = 19.1807(11) Å	gamma = 90 deg.
Volume	5417.2(5) $Å^3$	
Z, Calculated density	8, 1.405 Mg/m^3	
Absorption coefficient	0.261 mm^{-1}	
F(000)	2400	
Crystal size	0.12 x 0.06 x 0.04 mm	
Theta range for data collection	1.21 to 25.01 deg.	
Limiting indices	-40<=h<=40, -9<=k<=9, -22<=l<=22	
Reflections collected / unique	30722 / 4771 [R(int) = 0.1645]	
Completeness to theta = 25.01	99.9 %	
Absorption correction	Semi-empirical from equivalents	
Max. and min. transmission	0.9896 and 0.9694	
Refinement method	Full-matrix least-squares on F^2	
Data / restraints / parameters	4771 / 0 / 361	
Goodness-of-fit on F^2	1.022	
Final R indices [I>2sigma(I)]	R_1 = 0.0595, wR_2 = 0.1126	
R indices (all data)	R_1 = 0.1466, wR_2 = 0.1517	
Largest diff. peak and hole	0.292 and -0.288 e. $Å^{-3}$	

Supplementary Table 5. Atomic coordinates (x10^4) and equivalent isotropic displacement parameters (Å^{2}x10^3) for major-**4e**. U(eq) is defined as one third of the trace of the orthogonalized U^{ij} tensor.

	x	y	z	U(eq)
S(1)	624(1)	8456(2)	3498(1)	38(1)
Cl(1)	2610(1)	11083(2)	4246(1)	66(1)
N(1)	1577(1)	11151(4)	6216(2)	36(1)
N(2)	959(1)	8910(4)	4179(2)	32(1)
O(1)	262(1)	8130(4)	3778(2)	52(1)
O(2)	798(1)	7264(4)	3099(2)	54(1)
O(3)	616(1)	8324(4)	5141(2)	44(1)
O(4)	1643(1)	9845(4)	6728(1)	40(1)
C(1)	583(1)	10223(6)	3001(2)	32(1)
C(2)	495(1)	10083(6)	2283(2)	37(1)
C(3)	433(1)	11438(7)	1884(2)	44(1)
C(4)	470(1)	12950(6)	2181(2)	41(1)
C(5)	563(1)	13060(6)	2904(2)	40(1)
C(6)	616(1)	11705(6)	3321(2)	38(1)
C(7)	414(2)	14433(6)	1734(3)	64(2)
C(8)	1008(1)	12102(5)	5379(2)	33(1)
C(9)	1213(1)	13205(6)	5013(2)	39(1)
C(10)	1009(2)	14453(6)	4654(2)	42(1)
C(11)	603(2)	14575(6)	4644(2)	46(1)
C(12)	398(2)	13470(6)	4991(2)	47(1)
C(13)	600(1)	12262(6)	5361(2)	40(1)
C(14)	1203(1)	10722(5)	5791(2)	31(1)
C(15)	1298(1)	9275(5)	5327(2)	27(1)
C(16)	1562(1)	9666(5)	4760(2)	31(1)
C(17)	1950(1)	10182(6)	4810(2)	37(1)
C(18)	2121(1)	10428(6)	4194(2)	39(1)
C(19)	1914(1)	10185(6)	3550(2)	40(1)
C(20)	1525(1)	9676(6)	3492(2)	38(1)
C(21)	1356(1)	9416(5)	4104(2)	30(1)
C(22)	909(1)	8768(5)	4904(2)	31(1)
C(23)	1535(2)	12528(6)	6677(2)	51(1)

C(24)	1448(1)	7901(5)	5816(2)	34(1)
C(25)	1763(1)	8418(6)	6394(2)	36(1)
C(26)	1839(1)	7237(6)	6990(2)	36(1)
C(27)	1533(1)	6689(7)	7337(2)	49(1)
C(28)	1599(1)	5741(6)	7933(2)	45(1)
C(29)	1978(1)	5329(6)	8203(2)	42(1)
C(30)	2287(1)	5855(6)	7850(2)	43(1)
C(31)	2219(1)	6797(6)	7253(2)	47(1)
C(32)	2049(2)	4370(6)	8867(2)	53(2)

Supplementary Table 6. Bond lengths [Å] and angles [deg] for major-**4e**.

S(1)-O(1)	1.422(3)
S(1)-O(2)	1.425(3)
S(1)-N(2)	1.677(3)
S(1)-C(1)	1.756(5)
Cl(1)-C(18)	1.740(4)
N(1)-C(23)	1.469(5)
N(1)-O(4)	1.470(4)
N(1)-C(14)	1.474(5)
N(2)-C(22)	1.426(5)
N(2)-C(21)	1.435(5)
O(3)-C(22)	1.197(5)
O(4)-C(25)	1.435(5)
C(1)-C(2)	1.381(6)
C(1)-C(6)	1.382(6)
C(2)-C(3)	1.372(6)
C(2)-H(2A)	0.9300
C(3)-C(4)	1.387(7)
C(3)-H(3A)	0.9300
C(4)-C(5)	1.392(6)
C(4)-C(7)	1.509(6)
C(5)-C(6)	1.387(6)
C(5)-H(5A)	0.9300
C(6)-H(6A)	0.9300
C(7)-H(7A)	0.9600

C(7)-H(7B)	0.9600
C(7)-H(7C)	0.9600
C(8)-C(13)	1.389(6)
C(8)-C(9)	1.391(6)
C(8)-C(14)	1.510(6)
C(9)-C(10)	1.393(6)
C(9)-H(9A)	0.9300
C(10)-C(11)	1.382(6)
C(10)-H(10A)	0.9300
C(11)-C(12)	1.372(6)
C(11)-H(11A)	0.9300
C(12)-C(13)	1.374(6)
C(12)-H(12A)	0.9300
C(13)-H(13A)	0.9300
C(14)-C(15)	1.557(6)
C(14)-H(14A)	0.9800
C(15)-C(16)	1.519(5)
C(15)-C(22)	1.530(6)
C(15)-C(24)	1.535(6)
C(16)-C(17)	1.380(6)
C(16)-C(21)	1.386(5)
C(17)-C(18)	1.391(6)
C(17)-H(17A)	0.9300
C(18)-C(19)	1.366(6)
C(19)-C(20)	1.379(6)
C(19)-H(19A)	0.9300
C(20)-C(21)	1.381(5)
C(20)-H(20A)	0.9300
C(23)-H(23A)	0.9600
C(23)-H(23B)	0.9600
C(23)-H(23C)	0.9600
C(24)-C(25)	1.517(6)
C(24)-H(24A)	0.9700
C(24)-H(24B)	0.9700
C(25)-C(26)	1.511(6)
C(25)-H(25A)	0.9800
C(26)-C(27)	1.375(6)
C(26)-C(31)	1.382(6)

C(27)-C(28)	1.388(6)
C(27)-H(27A)	0.9300
C(28)-C(29)	1.375(6)
C(28)-H(28A)	0.9300
C(29)-C(30)	1.382(6)
C(29)-C(32)	1.502(6)
C(30)-C(31)	1.388(6)
C(30)-H(30A)	0.9300
C(31)-H(31A)	0.9300
C(32)-H(32A)	0.9600
C(32)-H(32B)	0.9600
C(32)-H(32C)	0.9600
O(1)-S(1)-O(2)	119.9(2)
O(1)-S(1)-N(2)	106.80(18)
O(2)-S(1)-N(2)	106.85(19)
O(1)-S(1)-C(1)	110.3(2)
O(2)-S(1)-C(1)	108.1(2)
N(2)-S(1)-C(1)	103.7(2)
C(23)-N(1)-O(4)	101.5(3)
C(23)-N(1)-C(14)	112.9(3)
O(4)-N(1)-C(14)	104.3(3)
C(22)-N(2)-C(21)	109.8(3)
C(22)-N(2)-S(1)	126.6(3)
C(21)-N(2)-S(1)	123.5(3)
C(25)-O(4)-N(1)	110.5(3)
C(2)-C(1)-C(6)	121.0(4)
C(2)-C(1)-S(1)	117.7(4)
C(6)-C(1)-S(1)	121.2(3)
C(3)-C(2)-C(1)	119.3(5)
C(3)-C(2)-H(2A)	120.3
C(1)-C(2)-H(2A)	120.3
C(2)-C(3)-C(4)	121.6(4)
C(2)-C(3)-H(3A)	119.2
C(4)-C(3)-H(3A)	119.2
C(3)-C(4)-C(5)	118.0(4)
C(3)-C(4)-C(7)	121.2(4)
C(5)-C(4)-C(7)	120.8(5)
C(6)-C(5)-C(4)	121.4(5)

C(6)-C(5)-H(5A)	119.3
C(4)-C(5)-H(5A)	119.3
C(1)-C(6)-C(5)	118.6(4)
C(1)-C(6)-H(6A)	120.7
C(5)-C(6)-H(6A)	120.7
C(4)-C(7)-H(7A)	109.5
C(4)-C(7)-H(7B)	109.5
H(7A)-C(7)-H(7B)	109.5
C(4)-C(7)-H(7C)	109.5
H(7A)-C(7)-H(7C)	109.5
H(7B)-C(7)-H(7C)	109.5
C(13)-C(8)-C(9)	118.5(4)
C(13)-C(8)-C(14)	117.7(4)
C(9)-C(8)-C(14)	123.8(4)
C(8)-C(9)-C(10)	119.9(4)
C(8)-C(9)-H(9A)	120.0
C(10)-C(9)-H(9A)	120.0
C(11)-C(10)-C(9)	120.2(5)
C(11)-C(10)-H(10A)	119.9
C(9)-C(10)-H(10A)	119.9
C(12)-C(11)-C(10)	120.1(5)
C(12)-C(11)-H(11A)	120.0
C(10)-C(11)-H(11A)	120.0
C(11)-C(12)-C(13)	119.8(5)
C(11)-C(12)-H(12A)	120.1
C(13)-C(12)-H(12A)	120.1
C(12)-C(13)-C(8)	121.5(5)
C(12)-C(13)-H(13A)	119.3
C(8)-C(13)-H(13A)	119.3
N(1)-C(14)-C(8)	113.8(4)
N(1)-C(14)-C(15)	106.7(3)
C(8)-C(14)-C(15)	113.7(3)
N(1)-C(14)-H(14A)	107.5
C(8)-C(14)-H(14A)	107.5
C(15)-C(14)-H(14A)	107.5
C(16)-C(15)-C(22)	103.0(3)
C(16)-C(15)-C(24)	114.6(3)
C(22)-C(15)-C(24)	108.9(3)

C(16)-C(15)-C(14)	114.6(4)
C(22)-C(15)-C(14)	107.3(3)
C(24)-C(15)-C(14)	108.0(3)
C(17)-C(16)-C(21)	119.4(4)
C(17)-C(16)-C(15)	130.8(4)
C(21)-C(16)-C(15)	109.9(4)
C(16)-C(17)-C(18)	118.3(4)
C(16)-C(17)-H(17A)	120.8
C(18)-C(17)-H(17A)	120.8
C(19)-C(18)-C(17)	121.7(4)
C(19)-C(18)-Cl(1)	119.3(3)
C(17)-C(18)-Cl(1)	119.0(3)
C(18)-C(19)-C(20)	120.6(4)
C(18)-C(19)-H(19A)	119.7
C(20)-C(19)-H(19A)	119.7
C(19)-C(20)-C(21)	117.8(4)
C(19)-C(20)-H(20A)	121.1
C(21)-C(20)-H(20A)	121.1
C(20)-C(21)-C(16)	122.2(4)
C(20)-C(21)-N(2)	128.1(4)
C(16)-C(21)-N(2)	109.7(3)
O(3)-C(22)-N(2)	126.2(4)
O(3)-C(22)-C(15)	126.2(4)
N(2)-C(22)-C(15)	107.6(3)
N(1)-C(23)-H(23A)	109.5
N(1)-C(23)-H(23B)	109.5
H(23A)-C(23)-H(23B)	109.5
N(1)-C(23)-H(23C)	109.5
H(23A)-C(23)-H(23C)	109.5
H(23B)-C(23)-H(23C)	109.5
C(25)-C(24)-C(15)	113.1(4)
C(25)-C(24)-H(24A)	109.0
C(15)-C(24)-H(24A)	109.0
C(25)-C(24)-H(24B)	109.0
C(15)-C(24)-H(24B)	109.0
H(24A)-C(24)-H(24B)	107.8
O(4)-C(25)-C(26)	103.9(3)
O(4)-C(25)-C(24)	110.6(3)

C(26)-C(25)-C(24)	114.4(4)
O(4)-C(25)-H(25A)	109.3
C(26)-C(25)-H(25A)	109.3
C(24)-C(25)-H(25A)	109.3
C(27)-C(26)-C(31)	117.3(4)
C(27)-C(26)-C(25)	120.7(4)
C(31)-C(26)-C(25)	121.6(4)
C(26)-C(27)-C(28)	121.9(4)
C(26)-C(27)-H(27A)	119.1
C(28)-C(27)-H(27A)	119.1
C(29)-C(28)-C(27)	120.8(4)
C(29)-C(28)-H(28A)	119.6
C(27)-C(28)-H(28A)	119.6
C(28)-C(29)-C(30)	117.7(4)
C(28)-C(29)-C(32)	120.6(4)
C(30)-C(29)-C(32)	121.8(4)
C(29)-C(30)-C(31)	121.3(4)
C(29)-C(30)-H(30A)	119.3
C(31)-C(30)-H(30A)	119.3
C(26)-C(31)-C(30)	121.0(4)
C(26)-C(31)-H(31A)	119.5
C(30)-C(31)-H(31A)	119.5
C(29)-C(32)-H(32A)	109.5
C(29)-C(32)-H(32B)	109.5
H(32A)-C(32)-H(32B)	109.5
C(29)-C(32)-H(32C)	109.5
H(32A)-C(32)-H(32C)	109.5
H(32B)-C(32)-H(32C)	109.5

Symmetry transformations used to generate equivalent atoms:

Supplementary Table 7. Anisotropic displacement parameters ($Å^2$ x 10^3) for major-**4e**. The anisotropic displacement factor exponent takes the form: -2 pi^2[h^2 a^{*2} U11 + ... + 2 h k a* b* U12]

	U11	U22	U33	U23	U13	U12
S(1)	44(1)	35(1)	32(1)	1(1)	-6(1)	-4(1)
Cl(1)	39(1)	110(1)	52(1)	2(1)	10(1)	-18(1)
N(1)	45(2)	31(3)	29(2)	3(2)	-1(2)	-2(2)

N(2)	30(2)	39(2)	26(2)	3(2)	-2(2)	-1(2)
O(1)	41(2)	69(3)	45(2)	15(2)	-6(2)	-20(2)
O(2)	81(3)	36(2)	43(2)	-11(2)	-10(2)	8(2)
O(3)	43(2)	56(2)	36(2)	-4(2)	7(2)	-13(2)
O(4)	51(2)	41(2)	27(2)	2(2)	-2(2)	4(2)
C(1)	30(2)	31(3)	34(3)	2(2)	2(2)	2(2)
C(2)	43(3)	40(3)	29(3)	-1(2)	4(2)	0(2)
C(3)	48(3)	59(4)	23(2)	3(3)	1(2)	2(3)
C(4)	32(3)	47(4)	44(3)	8(3)	5(2)	3(2)
C(5)	43(3)	33(3)	41(3)	-4(2)	0(2)	0(2)
C(6)	41(3)	42(3)	29(3)	2(3)	-2(2)	0(2)
C(7)	70(4)	58(4)	63(4)	28(3)	1(3)	1(3)
C(8)	37(3)	34(3)	27(2)	-3(2)	2(2)	4(2)
C(9)	38(3)	37(3)	41(3)	1(3)	6(2)	-5(2)
C(10)	64(3)	22(3)	41(3)	1(2)	7(3)	-2(2)
C(11)	64(4)	33(3)	39(3)	0(2)	0(3)	16(3)
C(12)	46(3)	46(3)	49(3)	-1(3)	8(2)	10(3)
C(13)	47(3)	36(3)	38(3)	2(2)	12(2)	2(2)
C(14)	32(2)	35(3)	25(2)	2(2)	1(2)	-1(2)
C(15)	33(2)	27(3)	23(2)	-2(2)	2(2)	1(2)
C(16)	34(3)	32(3)	25(2)	2(2)	0(2)	2(2)
C(17)	34(3)	49(3)	27(2)	1(2)	1(2)	-5(2)
C(18)	30(2)	51(4)	35(3)	-1(2)	3(2)	0(2)
C(19)	36(3)	55(4)	30(3)	4(2)	10(2)	3(2)
C(20)	46(3)	48(3)	22(2)	4(2)	4(2)	6(2)
C(21)	35(2)	29(3)	25(2)	-1(2)	0(2)	3(2)
C(22)	39(3)	25(3)	29(2)	1(2)	4(2)	2(2)
C(23)	67(4)	40(3)	44(3)	-7(3)	-2(3)	-1(3)
C(24)	40(3)	35(3)	27(2)	0(2)	3(2)	1(2)
C(25)	37(3)	42(3)	28(2)	-1(2)	1(2)	3(2)
C(26)	38(3)	40(3)	29(3)	1(2)	-2(2)	1(2)
C(27)	36(3)	70(4)	40(3)	9(3)	1(2)	2(3)
C(28)	41(3)	63(4)	32(3)	8(3)	5(2)	-8(3)
C(29)	47(3)	43(3)	35(3)	1(2)	2(2)	4(2)
C(30)	34(3)	46(3)	48(3)	11(3)	4(2)	8(2)
C(31)	39(3)	51(4)	51(3)	8(3)	7(2)	4(3)
C(32)	57(3)	58(4)	44(3)	13(3)	2(3)	2(3)

Supplementary Table 8. Hydrogen coordinates (x10^4) and isotropic displacement parameters (Å^{2}x10^3) for major-**4e**.

	x	y	z	U(eq)
H(2A)	478	9081	2073	45
H(3A)	364	11341	1403	52
H(5A)	591	14063	3113	48
H(6A)	673	11792	3805	45
H(7A)	351	14134	1251	96
H(7B)	654	15050	1784	96
H(7C)	201	15060	1881	96
H(9A)	1485	13109	5009	47
H(10A)	1147	15207	4420	51
H(11A)	467	15408	4401	55
H(12A)	124	13539	4975	56
H(13A)	460	11534	5605	48
H(14A)	1019	10348	6114	37
H(17A)	2093	10361	5246	44
H(19A)	2036	10365	3147	48
H(20A)	1382	9514	3055	46
H(23A)	1787	12764	6937	76
H(23B)	1345	12282	6997	76
H(23C)	1446	13438	6399	76
H(24A)	1226	7450	6026	41
H(24B)	1555	7068	5540	41
H(25A)	2011	8622	6193	43
H(27A)	1274	6962	7169	59
H(28A)	1385	5380	8151	54
H(30A)	2545	5573	8017	51
H(31A)	2433	7136	7027	56
H(32A)	1799	4108	9029	80
H(32B)	2205	4985	9219	80
H(32C)	2187	3404	8777	80

Supplementary Table 9. Torsion angles [deg] for major-**4e**.

O(1)-S(1)-N(2)-C(22)	8.5(4)
O(2)-S(1)-N(2)-C(22)	-120.9(4)
C(1)-S(1)-N(2)-C(22)	125.0(4)
O(1)-S(1)-N(2)-C(21)	-175.7(3)
O(2)-S(1)-N(2)-C(21)	54.9(4)

C(1)-S(1)-N(2)-C(21)	-59.2(4)
C(23)-N(1)-O(4)-C(25)	-169.6(3)
C(14)-N(1)-O(4)-C(25)	72.9(4)
O(1)-S(1)-C(1)-C(2)	-97.0(4)
O(2)-S(1)-C(1)-C(2)	35.8(4)
N(2)-S(1)-C(1)-C(2)	149.0(3)
O(1)-S(1)-C(1)-C(6)	79.7(4)
O(2)-S(1)-C(1)-C(6)	-147.5(4)
N(2)-S(1)-C(1)-C(6)	-34.3(4)
C(6)-C(1)-C(2)-C(3)	-1.3(6)
S(1)-C(1)-C(2)-C(3)	175.4(3)
C(1)-C(2)-C(3)-C(4)	2.3(7)
C(2)-C(3)-C(4)-C(5)	-1.5(7)
C(2)-C(3)-C(4)-C(7)	178.0(4)
C(3)-C(4)-C(5)-C(6)	-0.4(7)
C(7)-C(4)-C(5)-C(6)	-179.8(4)
C(2)-C(1)-C(6)-C(5)	-0.5(6)
S(1)-C(1)-C(6)-C(5)	-177.1(3)
C(4)-C(5)-C(6)-C(1)	1.3(6)
C(13)-C(8)-C(9)-C(10)	1.5(6)
C(14)-C(8)-C(9)-C(10)	-179.3(4)
C(8)-C(9)-C(10)-C(11)	-1.7(7)
C(9)-C(10)-C(11)-C(12)	0.3(7)
C(10)-C(11)-C(12)-C(13)	1.4(7)
C(11)-C(12)-C(13)-C(8)	-1.7(7)
C(9)-C(8)-C(13)-C(12)	0.3(7)
C(14)-C(8)-C(13)-C(12)	-179.1(4)
C(23)-N(1)-C(14)-C(8)	52.9(5)
O(4)-N(1)-C(14)-C(8)	162.2(3)
C(23)-N(1)-C(14)-C(15)	179.1(3)
O(4)-N(1)-C(14)-C(15)	-71.6(4)
C(13)-C(8)-C(14)-N(1)	-137.5(4)
C(9)-C(8)-C(14)-N(1)	43.2(6)
C(13)-C(8)-C(14)-C(15)	100.1(5)
C(9)-C(8)-C(14)-C(15)	-79.2(5)
N(1)-C(14)-C(15)-C(16)	-68.7(4)
C(8)-C(14)-C(15)-C(16)	57.5(5)
N(1)-C(14)-C(15)-C(22)	177.6(3)
C(8)-C(14)-C(15)-C(22)	-56.2(4)
N(1)-C(14)-C(15)-C(24)	60.4(4)
C(8)-C(14)-C(15)-C(24)	-173.4(4)
C(22)-C(15)-C(16)-C(17)	-179.5(5)
C(24)-C(15)-C(16)-C(17)	-61.4(6)
C(14)-C(15)-C(16)-C(17)	64.2(6)

C(22)-C(15)-C(16)-C(21)	-0.2(5)
C(24)-C(15)-C(16)-C(21)	117.9(4)
C(14)-C(15)-C(16)-C(21)	-116.5(4)
C(21)-C(16)-C(17)-C(18)	-0.2(7)
C(15)-C(16)-C(17)-C(18)	179.1(4)
C(16)-C(17)-C(18)-C(19)	0.6(7)
C(16)-C(17)-C(18)-Cl(1)	179.8(4)
C(17)-C(18)-C(19)-C(20)	-0.3(8)
Cl(1)-C(18)-C(19)-C(20)	-179.6(4)
C(18)-C(19)-C(20)-C(21)	-0.3(7)
C(19)-C(20)-C(21)-C(16)	0.8(7)
C(19)-C(20)-C(21)-N(2)	179.1(4)
C(17)-C(16)-C(21)-C(20)	-0.5(7)
C(15)-C(16)-C(21)-C(20)	-179.9(4)
C(17)-C(16)-C(21)-N(2)	-179.1(4)
C(15)-C(16)-C(21)-N(2)	1.5(5)
C(22)-N(2)-C(21)-C(20)	179.2(4)
S(1)-N(2)-C(21)-C(20)	2.8(6)
C(22)-N(2)-C(21)-C(16)	-2.3(5)
S(1)-N(2)-C(21)-C(16)	-178.7(3)
C(21)-N(2)-C(22)-O(3)	-177.6(4)
S(1)-N(2)-C(22)-O(3)	-1.3(7)
C(21)-N(2)-C(22)-C(15)	2.1(5)
S(1)-N(2)-C(22)-C(15)	178.4(3)
C(16)-C(15)-C(22)-O(3)	178.5(4)
C(24)-C(15)-C(22)-O(3)	56.5(6)
C(14)-C(15)-C(22)-O(3)	-60.1(6)
C(16)-C(15)-C(22)-N(2)	-1.1(4)
C(24)-C(15)-C(22)-N(2)	-123.2(4)
C(14)-C(15)-C(22)-N(2)	120.2(4)
C(16)-C(15)-C(24)-C(25)	81.3(4)
C(22)-C(15)-C(24)-C(25)	-164.0(3)
C(14)-C(15)-C(24)-C(25)	-47.8(5)
N(1)-O(4)-C(25)-C(26)	177.1(3)
N(1)-O(4)-C(25)-C(24)	-59.7(4)
C(15)-C(24)-C(25)-O(4)	47.7(5)
C(15)-C(24)-C(25)-C(26)	164.5(4)
O(4)-C(25)-C(26)-C(27)	66.8(5)
C(24)-C(25)-C(26)-C(27)	-53.9(6)
O(4)-C(25)-C(26)-C(31)	-106.3(5)
C(24)-C(25)-C(26)-C(31)	133.0(5)
C(31)-C(26)-C(27)-C(28)	0.4(8)
C(25)-C(26)-C(27)-C(28)	-172.9(4)
C(26)-C(27)-C(28)-C(29)	0.8(8)

C(27)-C(28)-C(29)-C(30)	-1.7(7)
C(27)-C(28)-C(29)-C(32)	177.0(5)
C(28)-C(29)-C(30)-C(31)	1.4(7)
C(32)-C(29)-C(30)-C(31)	-177.3(5)
C(27)-C(26)-C(31)-C(30)	-0.8(7)
C(25)-C(26)-C(31)-C(30)	172.5(4)
C(29)-C(30)-C(31)-C(26)	-0.1(8)

Symmetry transformations used to generate equivalent atoms:

Supplementary Table 10. Hydrogen bonds for major-**4e** [Å and deg.].

D-H...A	d(D-H)	d(H...A)	d(D...A)	<(DHA)

Tables for Single-Crystal X-ray Crystallography of minor-4e

Supplementary Table 11. Crystal data and structure refinement for minor-**4e**.

Identification code	minor-**4e**	
Empirical formula	$C_{32}H_{29}ClN_2O_4S$	
Formula weight	573.08	
Temperature	160.01 K	
Wavelength	0.71073 Å	
Crystal system	Triclinic	
Space group	P-1	
Unit cell dimensions	a = 8.9003(3) Å	α = 108.2960(10)°.
	b = 13.1778(4) Å	β = 102.6280(10)°.
	c = 13.2084(4) Å	γ = 99.4440(10)°.
Volume	1389.26(8) Å^3	
Z	2	
Density (calculated)	1.370 Mg/m^3	
Absorption coefficient	0.254 mm^{-1}	
F(000)	600	
Crystal size	0.3 x 0.25 x 0.2 mm^3	
Theta range for data collection	2.525 to 27.490°.	
Index ranges	-11<=h<=11, -17<=k<=17, -17<=l<=17	
Reflections collected	42723	

Independent reflections	6354 [$R_{(int)}$ = 0.0427]
Completeness to theta = 25.242°	99.6 %
Absorption correction	Semi-empirical from equivalents
Max. and min. transmission	0.7456 and 0.7073
Refinement method	Full-matrix least-squares on F^2
Data / restraints / parameters	6354 / 0 / 364
Goodness-of-fit on F^2	1.092
Final R indices [I>2sigma(I)]	R_1 = 0.0466, wR_2 = 0.0972
R indices (all data)	R_1 = 0.0570, wR_2 = 0.1025
Extinction coefficient	n/a
Largest diff. peak and hole	0.403 and -0.388 e.Å^{-3}

Supplementary Table 12. Atomic coordinates (x 10^4) and equivalent isotropic displacement parameters (Å^{2}x 10^3) for minor-**4e**. U(eq) is defined as one third of the trace of the orthogonalized U^{ij} tensor.

	x	y	z	U(eq)
Cl(1)	1529(1)	2376(1)	658(1)	33(1)
S(1)	5280(1)	7909(1)	1416(1)	24(1)
O(1)	6421(1)	6575(1)	5729(1)	21(1)
O(2)	6966(2)	8067(1)	3721(1)	25(1)
O(3)	6826(2)	8632(1)	1804(1)	34(1)
O(4)	4508(2)	7360(1)	254(1)	32(1)
N(1)	7629(2)	6500(1)	5131(1)	19(1)
N(2)	5422(2)	6897(1)	1945(1)	20(1)
C(1)	6802(2)	5736(1)	3978(1)	17(1)
C(2)	5487(2)	6202(1)	3420(1)	18(1)
C(3)	4448(2)	5366(1)	2311(1)	17(1)
C(4)	3591(2)	4301(1)	2073(1)	20(1)
C(5)	2675(2)	3704(2)	983(2)	22(1)
C(6)	2604(2)	4139(2)	154(2)	25(1)
C(7)	3466(2)	5205(2)	392(1)	24(1)
C(8)	4379(2)	5810(1)	1482(1)	19(1)

C(9)	6126(2)	7178(1)	3104(1)	20(1)
C(10)	8723(2)	6012(2)	5716(2)	26(1)
C(11)	7976(2)	5538(2)	3311(1)	21(1)
C(12)	7832(2)	4479(2)	2581(2)	28(1)
C(13)	8848(3)	4299(2)	1918(2)	38(1)
C(14)	10007(3)	5168(2)	1976(2)	42(1)
C(15)	10181(2)	6217(2)	2716(2)	37(1)
C(16)	9173(2)	6410(2)	3384(2)	27(1)
C(17)	4463(2)	6610(1)	4197(1)	20(1)
C(18)	5503(2)	7272(1)	5382(1)	20(1)
C(19)	4609(2)	7663(1)	6221(2)	23(1)
C(20)	3022(2)	7686(2)	5913(2)	29(1)
C(21)	2290(3)	8132(2)	6717(2)	34(1)
C(22)	3097(3)	8545(2)	7843(2)	34(1)
C(23)	4677(3)	8510(2)	8146(2)	34(1)
C(24)	5426(3)	8092(2)	7351(2)	29(1)
C(25)	2280(3)	9011(2)	8711(2)	48(1)
C(26)	3982(2)	8555(2)	2061(2)	22(1)
C(27)	2362(2)	8210(2)	1520(2)	30(1)
C(28)	1329(2)	8664(2)	2060(2)	34(1)
C(29)	1882(2)	9433(2)	3148(2)	31(1)
C(30)	3513(2)	9765(2)	3662(2)	28(1)
C(31)	4573(2)	9347(2)	3129(2)	26(1)
C(32)	741(3)	9890(2)	3741(2)	51(1)

Supplementary Table 13. Bond lengths [Å] and angles [°] for minor-**4e**.

Cl(1)-C(5)	1.7429(18)
S(1)-O(3)	1.4204(15)
S(1)-O(4)	1.4246(14)
S(1)-N(2)	1.6988(15)
S(1)-C(26)	1.7550(19)
O(1)-N(1)	1.4655(18)
O(1)-C(18)	1.438(2)
O(2)-C(9)	1.201(2)
N(1)-C(1)	1.476(2)
N(1)-C(10)	1.465(2)
N(2)-C(8)	1.433(2)

N(2)-C(9)	1.421(2)
C(1)-H(1)	1.0000
C(1)-C(2)	1.560(2)
C(1)-C(11)	1.512(2)
C(2)-C(3)	1.508(2)
C(2)-C(9)	1.533(2)
C(2)-C(17)	1.548(2)
C(3)-C(4)	1.384(2)
C(3)-C(8)	1.390(2)
C(4)-H(4)	0.9500
C(4)-C(5)	1.387(2)
C(5)-C(6)	1.381(3)
C(6)-H(6)	0.9500
C(6)-C(7)	1.388(3)
C(7)-H(7)	0.9500
C(7)-C(8)	1.387(2)
C(10)-H(10A)	0.9800
C(10)-H(10B)	0.9800
C(10)-H(10C)	0.9800
C(11)-C(12)	1.391(3)
C(11)-C(16)	1.396(3)
C(12)-H(12)	0.9500
C(12)-C(13)	1.389(3)
C(13)-H(13)	0.9500
C(13)-C(14)	1.379(4)
C(14)-H(14)	0.9500
C(14)-C(15)	1.380(3)
C(15)-H(15)	0.9500
C(15)-C(16)	1.389(3)
C(16)-H(16)	0.9500
C(17)-H(17A)	0.9900
C(17)-H(17B)	0.9900
C(17)-C(18)	1.520(2)
C(18)-H(18)	1.0000
C(18)-C(19)	1.512(2)
C(19)-C(20)	1.390(3)
C(19)-C(24)	1.392(3)
C(20)-H(20)	0.9500

C(20)-C(21)	1.391(3)
C(21)-H(21)	0.9500
C(21)-C(22)	1.386(3)
C(22)-C(23)	1.388(3)
C(22)-C(25)	1.513(3)
C(23)-H(23)	0.9500
C(23)-C(24)	1.383(3)
C(24)-H(24)	0.9500
C(25)-H(25A)	0.9800
C(25)-H(25B)	0.9800
C(25)-H(25C)	0.9800
C(26)-C(27)	1.388(3)
C(26)-C(31)	1.388(3)
C(27)-H(27)	0.9500
C(27)-C(28)	1.383(3)
C(28)-H(28)	0.9500
C(28)-C(29)	1.395(3)
C(29)-C(30)	1.390(3)
C(29)-C(32)	1.504(3)
C(30)-H(30)	0.9500
C(30)-C(31)	1.381(3)
C(31)-H(31)	0.9500
C(32)-H(32A)	0.9800
C(32)-H(32B)	0.9800
C(32)-H(32C)	0.9800
O(3)-S(1)-O(4)	120.79(9)
O(3)-S(1)-N(2)	106.22(8)
O(3)-S(1)-C(26)	110.49(9)
O(4)-S(1)-N(2)	106.02(8)
O(4)-S(1)-C(26)	109.29(9)
N(2)-S(1)-C(26)	102.27(8)
C(18)-O(1)-N(1)	106.63(12)
O(1)-N(1)-C(1)	106.34(12)
C(10)-N(1)-O(1)	102.95(12)
C(10)-N(1)-C(1)	111.05(14)
C(8)-N(2)-S(1)	125.35(12)
C(9)-N(2)-S(1)	120.01(12)
C(9)-N(2)-C(8)	109.81(13)

N(1)-C(1)-H(1)	108.4
N(1)-C(1)-C(2)	110.32(13)
N(1)-C(1)-C(11)	110.44(13)
C(2)-C(1)-H(1)	108.4
C(11)-C(1)-H(1)	108.4
C(11)-C(1)-C(2)	110.72(13)
C(3)-C(2)-C(1)	111.72(13)
C(3)-C(2)-C(9)	102.24(13)
C(3)-C(2)-C(17)	110.50(13)
C(9)-C(2)-C(1)	114.31(13)
C(9)-C(2)-C(17)	106.34(13)
C(17)-C(2)-C(1)	111.27(13)
C(4)-C(3)-C(2)	128.51(15)
C(4)-C(3)-C(8)	120.72(16)
C(8)-C(3)-C(2)	110.75(15)
C(3)-C(4)-H(4)	121.3
C(3)-C(4)-C(5)	117.43(16)
C(5)-C(4)-H(4)	121.3
C(4)-C(5)-Cl(1)	118.84(14)
C(6)-C(5)-Cl(1)	118.96(14)
C(6)-C(5)-C(4)	122.17(17)
C(5)-C(6)-H(6)	119.8
C(5)-C(6)-C(7)	120.39(16)
C(7)-C(6)-H(6)	119.8
C(6)-C(7)-H(7)	121.1
C(8)-C(7)-C(6)	117.78(16)
C(8)-C(7)-H(7)	121.1
C(3)-C(8)-N(2)	108.96(14)
C(7)-C(8)-N(2)	129.46(16)
C(7)-C(8)-C(3)	121.52(16)
O(2)-C(9)-N(2)	124.32(16)
O(2)-C(9)-C(2)	127.34(16)
N(2)-C(9)-C(2)	108.07(14)
N(1)-C(10)-H(10A)	109.5
N(1)-C(10)-H(10B)	109.5
N(1)-C(10)-H(10C)	109.5
H(10A)-C(10)-H(10B)	109.5
H(10A)-C(10)-H(10C)	109.5

H(10B)-C(10)-H(10C)	109.5
C(12)-C(11)-C(1)	119.77(16)
C(12)-C(11)-C(16)	119.20(17)
C(16)-C(11)-C(1)	120.99(16)
C(11)-C(12)-H(12)	119.9
C(13)-C(12)-C(11)	120.2(2)
C(13)-C(12)-H(12)	119.9
C(12)-C(13)-H(13)	119.8
C(14)-C(13)-C(12)	120.4(2)
C(14)-C(13)-H(13)	119.8
C(13)-C(14)-H(14)	120.1
C(13)-C(14)-C(15)	119.81(19)
C(15)-C(14)-H(14)	120.1
C(14)-C(15)-H(15)	119.8
C(14)-C(15)-C(16)	120.5(2)
C(16)-C(15)-H(15)	119.8
C(11)-C(16)-H(16)	120.0
C(15)-C(16)-C(11)	119.92(19)
C(15)-C(16)-H(16)	120.0
C(2)-C(17)-H(17A)	109.5
C(2)-C(17)-H(17B)	109.5
H(17A)-C(17)-H(17B)	108.0
C(18)-C(17)-C(2)	110.90(14)
C(18)-C(17)-H(17A)	109.5
C(18)-C(17)-H(17B)	109.5
O(1)-C(18)-C(17)	107.24(13)
O(1)-C(18)-H(18)	108.8
O(1)-C(18)-C(19)	108.12(14)
C(17)-C(18)-H(18)	108.8
C(19)-C(18)-C(17)	115.06(15)
C(19)-C(18)-H(18)	108.8
C(20)-C(19)-C(18)	122.82(16)
C(20)-C(19)-C(24)	117.87(17)
C(24)-C(19)-C(18)	119.15(17)
C(19)-C(20)-H(20)	119.7
C(19)-C(20)-C(21)	120.54(19)
C(21)-C(20)-H(20)	119.7
C(20)-C(21)-H(21)	119.2

C(22)-C(21)-C(20)	121.5(2)
C(22)-C(21)-H(21)	119.2
C(21)-C(22)-C(23)	117.69(18)
C(21)-C(22)-C(25)	121.1(2)
C(23)-C(22)-C(25)	121.2(2)
C(22)-C(23)-H(23)	119.4
C(24)-C(23)-C(22)	121.15(19)
C(24)-C(23)-H(23)	119.4
C(19)-C(24)-H(24)	119.4
C(23)-C(24)-C(19)	121.2(2)
C(23)-C(24)-H(24)	119.4
C(22)-C(25)-H(25A)	109.5
C(22)-C(25)-H(25B)	109.5
C(22)-C(25)-H(25C)	109.5
H(25A)-C(25)-H(25B)	109.5
H(25A)-C(25)-H(25C)	109.5
H(25B)-C(25)-H(25C)	109.5
C(27)-C(26)-S(1)	119.16(14)
C(27)-C(26)-C(31)	120.93(17)
C(31)-C(26)-S(1)	119.77(14)
C(26)-C(27)-H(27)	120.4
C(28)-C(27)-C(26)	119.17(18)
C(28)-C(27)-H(27)	120.4
C(27)-C(28)-H(28)	119.4
C(27)-C(28)-C(29)	121.17(18)
C(29)-C(28)-H(28)	119.4
C(28)-C(29)-C(32)	120.68(19)
C(30)-C(29)-C(28)	118.14(19)
C(30)-C(29)-C(32)	121.18(19)
C(29)-C(30)-H(30)	119.1
C(31)-C(30)-C(29)	121.77(18)
C(31)-C(30)-H(30)	119.1
C(26)-C(31)-H(31)	120.6
C(30)-C(31)-C(26)	118.76(18)
C(30)-C(31)-H(31)	120.6
C(29)-C(32)-H(32A)	109.5
C(29)-C(32)-H(32B)	109.5
C(29)-C(32)-H(32C)	109.5

H(32A)-C(32)-H(32B)	109.5
H(32A)-C(32)-H(32C)	109.5
H(32B)-C(32)-H(32C)	109.5

Symmetry transformations used to generate equivalent atoms:

Supplementary Table 14. Anisotropic displacement parameters ($Å^2$x 10^3) for minor-**4e**. The anisotropic displacement factor exponent takes the form: $-2p^2[h^2 a^{*2}U^{11} + ... + 2 h k a^* b^* U^{12}]$

	U^{11}	U^{22}	U^{33}	U^{23}	U^{13}	U^{12}
Cl(1)	31(1)	28(1)	27(1)	5(1)	-4(1)	-4(1)
S(1)	29(1)	28(1)	24(1)	16(1)	11(1)	10(1)
O(1)	21(1)	28(1)	17(1)	10(1)	6(1)	8(1)
O(2)	24(1)	22(1)	25(1)	9(1)	3(1)	3(1)
O(3)	32(1)	38(1)	44(1)	25(1)	17(1)	7(1)
O(4)	46(1)	37(1)	22(1)	18(1)	14(1)	17(1)
N(1)	16(1)	26(1)	16(1)	8(1)	3(1)	5(1)
N(2)	22(1)	24(1)	19(1)	12(1)	7(1)	8(1)
C(1)	16(1)	18(1)	17(1)	8(1)	2(1)	4(1)
C(2)	16(1)	21(1)	16(1)	7(1)	4(1)	5(1)
C(3)	13(1)	23(1)	16(1)	7(1)	3(1)	7(1)
C(4)	18(1)	25(1)	18(1)	9(1)	3(1)	7(1)
C(5)	17(1)	23(1)	22(1)	5(1)	4(1)	4(1)
C(6)	23(1)	32(1)	13(1)	3(1)	1(1)	8(1)
C(7)	25(1)	33(1)	16(1)	11(1)	6(1)	12(1)
C(8)	17(1)	24(1)	19(1)	9(1)	7(1)	9(1)
C(9)	18(1)	24(1)	20(1)	10(1)	7(1)	10(1)
C(10)	21(1)	34(1)	24(1)	16(1)	1(1)	8(1)
C(11)	18(1)	28(1)	17(1)	10(1)	2(1)	10(1)
C(12)	27(1)	32(1)	22(1)	8(1)	2(1)	14(1)
C(13)	40(1)	54(1)	21(1)	8(1)	5(1)	32(1)
C(14)	35(1)	81(2)	30(1)	29(1)	18(1)	38(1)
C(15)	21(1)	64(2)	42(1)	35(1)	13(1)	18(1)
C(16)	20(1)	35(1)	30(1)	17(1)	6(1)	11(1)
C(17)	17(1)	24(1)	18(1)	8(1)	4(1)	7(1)
C(18)	21(1)	20(1)	17(1)	6(1)	4(1)	6(1)

C(19)	28(1)	19(1)	22(1)	7(1)	10(1)	5(1)
C(20)	28(1)	30(1)	27(1)	6(1)	10(1)	7(1)
C(21)	33(1)	30(1)	44(1)	10(1)	21(1)	9(1)
C(22)	54(1)	20(1)	37(1)	11(1)	30(1)	10(1)
C(23)	53(1)	29(1)	22(1)	9(1)	15(1)	10(1)
C(24)	37(1)	27(1)	22(1)	8(1)	8(1)	8(1)
C(25)	74(2)	35(1)	50(1)	15(1)	45(1)	16(1)
C(26)	27(1)	22(1)	22(1)	12(1)	6(1)	9(1)
C(27)	31(1)	31(1)	21(1)	5(1)	2(1)	6(1)
C(28)	24(1)	40(1)	28(1)	5(1)	0(1)	6(1)
C(29)	30(1)	30(1)	31(1)	8(1)	7(1)	10(1)
C(30)	32(1)	20(1)	26(1)	3(1)	1(1)	7(1)
C(31)	25(1)	21(1)	29(1)	9(1)	0(1)	6(1)
C(32)	37(1)	56(2)	46(1)	-4(1)	12(1)	15(1)

Supplementary Table 15. Hydrogen coordinates (x 10^4) and isotropic displacement parameters ($Å^2$x 10^3) for minor-**4e**.

	x	y	z	U(eq)
H(1)	6281	5014	4002	21
H(4)	3630	3991	2635	24
H(6)	1961	3705	-584	30
H(7)	3431	5512	-172	28
H(10A)	9076	6455	6514	39
H(10B)	9647	5997	5427	39
H(10C)	8177	5257	5597	39
H(12)	7036	3877	2537	33
H(13)	8744	3575	1421	46
H(14)	10683	5045	1508	50
H(15)	10997	6811	2769	44
H(16)	9298	7134	3890	32
H(17A)	3843	7076	3919	24
H(17B)	3703	5967	4191	24
H(18)	6246	7929	5381	23

H(20)	2433	7394	5148	35
H(21)	1211	8155	6489	41
H(23)	5254	8777	8913	41
H(24)	6517	8098	7581	35
H(25A)	2737	8880	9390	72
H(25B)	1140	8649	8424	72
H(25C)	2434	9807	8881	72
H(27)	1967	7669	789	36
H(28)	223	8449	1684	40
H(30)	3908	10293	4400	34
H(31)	5685	9598	3486	32
H(32A)	940	9801	4464	77
H(32B)	-353	9492	3291	77
H(32C)	893	10676	3856	77

Supplementary Table 16. Torsion angles [°] for minor-**4e**.

Tables for Single-Crystal X-ray Crystallography of racemic major-5o

Supplementary Table 17. Crystal data and structure refinement for major-**5o**.

Identification code	major-**5o**	
Empirical formula	$C_{29}H_{32}BrN_2O_5P$	
Formula weight	599.45	
Temperature	296(2) K	
Wavelength	0.71073 Å	
Crystal system, space group	Triclinic, P-1	
Unit cell dimensions	*a* = 9.6736(7) Å	alpha = 108.990(2) deg.
	b = 12.6142(9) Å	beta = 99.303(2) deg.
	c = 12.7109(9) Å	gamma = 96.865(2) deg.
Volume	1422.50(18) $Å^3$	
Z, Calculated density	2, 1.400 Mg/m^3	
Absorption coefficient	1.542 mm^{-1}	
F(000)	620	
Crystal size	0.36 x 0.29 x 0.16 mm	
Theta range for data collection	1.73 to 25.01 deg.	

Limiting indices	-11<=h<=11, -14<=k<=14, -12<=l<=15
Reflections collected / unique	16675 / 4985 [R(int) = 0.0267]
Completeness to theta = 25.01	99.5 %
Absorption correction	Semi-empirical from equivalents
Max. and min. transmission	0.7905 and 0.6068
Refinement method	Full-matrix least-squares on F^2
Data / restraints / parameters	4985 / 10 / 343
Goodness-of-fit on F^2	1.059
Final R indices [I>2sigma(I)]	R_1 = 0.0521, wR_2 = 0.1544
R indices (all data)	R_1 = 0.0675, wR_2 = 0.1701
Largest diff. peak and hole	0.701 and -0.663 e.Å^{-3}

Supplementary Table 18. Atomic coordinates (x10^4) and equivalent isotropic displacement parameters (A^{2}x10^3) for major-**5o**. U(eq) is defined as one third of the trace of the orthogonalized Uij tensor.

	x	y	z	U(eq)
Br(1)	4153(1)	3171(1)	4355(1)	66(1)
P(1)	-2029(2)	-1370(1)	2224(1)	75(1)
O(1)	-2119(12)	-1551(5)	987(5)	208(5)
O(2)	-949(5)	-1932(3)	2595(7)	169(3)
O(3)	-3563(4)	-1727(3)	2272(3)	81(1)
O(4)	-2182(3)	4290(2)	4781(2)	43(1)
O(5)	-3830(3)	569(3)	2859(3)	67(1)
N(2)	-1724(3)	4014(3)	3687(2)	41(1)
N(1)	-1598(3)	63(3)	2912(3)	50(1)
C(1)	-942(4)	3952(4)	7519(3)	52(1)
C(2)	-1280(5)	4265(4)	8574(4)	62(1)
C(3)	-2403(5)	4819(4)	8811(3)	59(1)
C(4)	-3164(5)	5066(4)	7929(4)	64(1)
C(5)	-2847(4)	4758(4)	6861(3)	55(1)
C(6)	-1741(4)	4184(3)	6631(3)	43(1)
C(7)	-1408(4)	3764(3)	5467(3)	41(1)
C(8)	-1867(4)	2476(3)	4913(3)	43(1)
C(9)	-1676(3)	2045(3)	3663(3)	38(1)
C(10)	-2335(3)	2796(3)	3041(3)	41(1)

C(11)	-2171(4)	2473(3)	1820(3)	47(1)
C(12)	-910(5)	2798(4)	1520(4)	56(1)
C(13)	-813(6)	2528(5)	408(4)	72(1)
C(14)	-1965(7)	1901(5)	-445(5)	85(2)
C(15)	-3209(7)	1559(5)	-192(4)	89(2)
C(16)	-3338(5)	1854(4)	961(4)	67(1)
C(17)	-174(4)	691(3)	3226(3)	45(1)
C(18)	1074(5)	254(4)	3194(4)	60(1)
C(19)	2345(4)	1022(4)	3524(4)	60(1)
C(20)	2359(4)	2176(3)	3898(3)	46(1)
C(21)	1112(4)	2615(3)	3950(3)	42(1)
C(22)	-170(4)	1852(3)	3610(3)	39(1)
C(23)	-2540(4)	821(3)	3095(3)	47(1)
C(24)	-3909(11)	-1896(8)	3243(7)	136(3)
C(25)	-5352(9)	-1711(7)	3349(8)	132(3)
C(26)	-2320(18)	-988(10)	358(8)	230(8)
C(27)	-2473(11)	-1492(9)	-882(7)	161(4)
C(28)	-2457(4)	4730(3)	3170(3)	52(1)
C(29)	-2764(7)	5140(6)	9967(4)	91(2)

Supplementary Table 19. Bond lengths [Å] and angles [deg] for major-**5o**.

Br(1)-C(20)	1.898(4)
P(1)-O(2)	1.433(5)
P(1)-O(1)	1.499(7)
P(1)-O(3)	1.515(4)
P(1)-N(1)	1.697(3)
O(1)-C(26)	1.239(12)
O(3)-C(24)	1.404(8)
O(4)-C(7)	1.430(4)
O(4)-N(2)	1.474(4)
O(5)-C(23)	1.212(5)
N(2)-C(28)	1.462(5)
N(2)-C(10)	1.480(5)
N(1)-C(23)	1.390(5)
N(1)-C(17)	1.429(5)
C(1)-C(2)	1.375(6)
C(1)-C(6)	1.394(5)
C(1)-H(1A)	0.9300
C(2)-C(3)	1.382(6)

C(2)-H(2A)	0.9300
C(3)-C(4)	1.382(6)
C(3)-C(29)	1.502(6)
C(4)-C(5)	1.381(6)
C(4)-H(4A)	0.9300
C(5)-C(6)	1.379(5)
C(5)-H(5A)	0.9300
C(6)-C(7)	1.503(5)
C(7)-C(8)	1.522(5)
C(7)-H(7A)	0.9800
C(8)-C(9)	1.553(5)
C(8)-H(8A)	0.9700
C(8)-H(8B)	0.9700
C(9)-C(22)	1.513(5)
C(9)-C(23)	1.540(5)
C(9)-C(10)	1.549(5)
C(10)-C(11)	1.511(5)
C(10)-H(10A)	0.9800
C(11)-C(16)	1.383(6)
C(11)-C(12)	1.391(6)
C(12)-C(13)	1.364(6)
C(12)-H(12A)	0.9300
C(13)-C(14)	1.372(8)
C(13)-H(13A)	0.9300
C(14)-C(15)	1.352(9)
C(14)-H(14A)	0.9300
C(15)-C(16)	1.420(7)
C(15)-H(15A)	0.9300
C(16)-H(16A)	0.9300
C(17)-C(22)	1.385(5)
C(17)-C(18)	1.386(5)
C(18)-C(19)	1.382(6)
C(18)-H(18A)	0.9300
C(19)-C(20)	1.373(6)
C(19)-H(19A)	0.9300
C(20)-C(21)	1.389(5)
C(21)-C(22)	1.386(5)
C(21)-H(21A)	0.9300
C(24)-C(25)	1.463(11)
C(24)-H(24A)	0.9700
C(24)-H(24B)	0.9700
C(25)-H(25A)	0.9600
C(25)-H(25B)	0.9600

C(25)-H(25C)	0.9600
C(26)-C(27)	1.470(13)
C(26)-H(26A)	0.9700
C(26)-H(26B)	0.9700
C(27)-H(27A)	0.9600
C(27)-H(27B)	0.9600
C(27)-H(27C)	0.9600
C(28)-H(28A)	0.9600
C(28)-H(28B)	0.9600
C(28)-H(28C)	0.9600
C(29)-H(29A)	0.9600
C(29)-H(29B)	0.9600
C(29)-H(29C)	0.9600
O(2)-P(1)-O(1)	109.2(6)
O(2)-P(1)-O(3)	120.0(3)
O(1)-P(1)-O(3)	103.0(4)
O(2)-P(1)-N(1)	111.2(2)
O(1)-P(1)-N(1)	105.7(3)
O(3)-P(1)-N(1)	106.68(19)
C(26)-O(1)-P(1)	137.0(8)
C(24)-O(3)-P(1)	121.0(5)
C(7)-O(4)-N(2)	108.8(2)
C(28)-N(2)-O(4)	101.5(3)
C(28)-N(2)-C(10)	110.0(3)
O(4)-N(2)-C(10)	106.2(2)
C(23)-N(1)-C(17)	109.2(3)
C(23)-N(1)-P(1)	126.6(3)
C(17)-N(1)-P(1)	123.4(3)
C(2)-C(1)-C(6)	120.5(4)
C(2)-C(1)-H(1A)	119.8
C(6)-C(1)-H(1A)	119.8
C(1)-C(2)-C(3)	122.5(4)
C(1)-C(2)-H(2A)	118.8
C(3)-C(2)-H(2A)	118.8
C(4)-C(3)-C(2)	116.3(4)
C(4)-C(3)-C(29)	122.2(4)
C(2)-C(3)-C(29)	121.5(4)
C(5)-C(4)-C(3)	122.2(4)
C(5)-C(4)-H(4A)	118.9
C(3)-C(4)-H(4A)	118.9
C(6)-C(5)-C(4)	120.8(4)
C(6)-C(5)-H(5A)	119.6
C(4)-C(5)-H(5A)	119.6

C(5)-C(6)-C(1)	117.7(4)
C(5)-C(6)-C(7)	123.0(3)
C(1)-C(6)-C(7)	119.3(3)
O(4)-C(7)-C(6)	107.9(3)
O(4)-C(7)-C(8)	108.7(3)
C(6)-C(7)-C(8)	111.0(3)
O(4)-C(7)-H(7A)	109.7
C(6)-C(7)-H(7A)	109.7
C(8)-C(7)-H(7A)	109.7
C(7)-C(8)-C(9)	111.6(3)
C(7)-C(8)-H(8A)	109.3
C(9)-C(8)-H(8A)	109.3
C(7)-C(8)-H(8B)	109.3
C(9)-C(8)-H(8B)	109.3
H(8A)-C(8)-H(8B)	108.0
C(22)-C(9)-C(23)	101.9(3)
C(22)-C(9)-C(10)	119.4(3)
C(23)-C(9)-C(10)	108.0(3)
C(22)-C(9)-C(8)	111.1(3)
C(23)-C(9)-C(8)	106.8(3)
C(10)-C(9)-C(8)	108.7(3)
N(2)-C(10)-C(11)	110.8(3)
N(2)-C(10)-C(9)	110.0(3)
C(11)-C(10)-C(9)	113.3(3)
N(2)-C(10)-H(10A)	107.5
C(11)-C(10)-H(10A)	107.5
C(9)-C(10)-H(10A)	107.5
C(16)-C(11)-C(12)	118.5(4)
C(16)-C(11)-C(10)	118.6(4)
C(12)-C(11)-C(10)	122.9(3)
C(13)-C(12)-C(11)	121.4(4)
C(13)-C(12)-H(12A)	119.3
C(11)-C(12)-H(12A)	119.3
C(12)-C(13)-C(14)	120.1(5)
C(12)-C(13)-H(13A)	119.9
C(14)-C(13)-H(13A)	119.9
C(15)-C(14)-C(13)	120.4(5)
C(15)-C(14)-H(14A)	119.8
C(13)-C(14)-H(14A)	119.8
C(14)-C(15)-C(16)	120.2(5)
C(14)-C(15)-H(15A)	119.9
C(16)-C(15)-H(15A)	119.9
C(11)-C(16)-C(15)	119.3(5)

C(11)-C(16)-H(16A)	120.3
C(15)-C(16)-H(16A)	120.3
C(22)-C(17)-C(18)	122.2(4)
C(22)-C(17)-N(1)	110.5(3)
C(18)-C(17)-N(1)	127.3(4)
C(19)-C(18)-C(17)	117.7(4)
C(19)-C(18)-H(18A)	121.2
C(17)-C(18)-H(18A)	121.2
C(20)-C(19)-C(18)	120.6(4)
C(20)-C(19)-H(19A)	119.7
C(18)-C(19)-H(19A)	119.7
C(19)-C(20)-C(21)	121.8(4)
C(19)-C(20)-Br(1)	117.9(3)
C(21)-C(20)-Br(1)	120.3(3)
C(22)-C(21)-C(20)	118.0(3)
C(22)-C(21)-H(21A)	121.0
C(20)-C(21)-H(21A)	121.0
C(17)-C(22)-C(21)	119.7(3)
C(17)-C(22)-C(9)	109.0(3)
C(21)-C(22)-C(9)	131.1(3)
O(5)-C(23)-N(1)	126.0(4)
O(5)-C(23)-C(9)	125.3(3)
N(1)-C(23)-C(9)	108.7(3)
O(3)-C(24)-C(25)	111.7(7)
O(3)-C(24)-H(24A)	109.3
C(25)-C(24)-H(24A)	109.3
O(3)-C(24)-H(24B)	109.3
C(25)-C(24)-H(24B)	109.3
H(24A)-C(24)-H(24B)	108.0
C(24)-C(25)-H(25A)	109.5
C(24)-C(25)-H(25B)	109.5
H(25A)-C(25)-H(25B)	109.5
C(24)-C(25)-H(25C)	109.5
H(25A)-C(25)-H(25C)	109.5
H(25B)-C(25)-H(25C)	109.5
O(1)-C(26)-C(27)	122.1(9)
O(1)-C(26)-H(26A)	106.8
C(27)-C(26)-H(26A)	106.8
O(1)-C(26)-H(26B)	106.8
C(27)-C(26)-H(26B)	106.8
H(26A)-C(26)-H(26B)	106.7
C(26)-C(27)-H(27A)	109.5
C(26)-C(27)-H(27B)	109.5

H(27A)-C(27)-H(27B)	109.5
C(26)-C(27)-H(27C)	109.5
H(27A)-C(27)-H(27C)	109.5
H(27B)-C(27)-H(27C)	109.5
N(2)-C(28)-H(28A)	109.5
N(2)-C(28)-H(28B)	109.5
H(28A)-C(28)-H(28B)	109.5
N(2)-C(28)-H(28C)	109.5
H(28A)-C(28)-H(28C)	109.5
H(28B)-C(28)-H(28C)	109.5
C(3)-C(29)-H(29A)	109.5
C(3)-C(29)-H(29B)	109.5
H(29A)-C(29)-H(29B)	109.5
C(3)-C(29)-H(29C)	109.5
H(29A)-C(29)-H(29C)	109.5
H(29B)-C(29)-H(29C)	109.5

Symmetry transformations used to generate equivalent atoms:

Supplementary Table 20. Anisotropic displacement parameters ($Å^2x10^3$) for major-**5o**. The anisotropic displacement factor exponent takes the form: -2 pi^2[h^2 a^{*2} U11 + ... + 2 h k a* b* U12]

	U11	U22	U33	U23	U13	U12
Br(1)	36(1)	69(1)	96(1)	31(1)	14(1)	13(1)
P(1)	81(1)	38(1)	98(1)	6(1)	39(1)	5(1)
O(1)	387(13)	92(4)	101(2)	-14(3)	101(6)	-51(6)
O(2)	87(3)	40(2)	349(9)	30(3)	29(3)	28(2)
O(3)	81(2)	60(2)	102(3)	26(2)	32(2)	-1(2)
O(4)	48(1)	44(1)	41(1)	16(1)	12(1)	16(1)
O(5)	43(2)	49(2)	102(2)	18(2)	19(2)	2(1)
N(2)	42(2)	43(2)	41(2)	16(1)	13(1)	10(1)
N(1)	50(2)	38(2)	60(2)	11(2)	18(2)	9(1)
C(1)	50(2)	61(2)	50(2)	24(2)	9(2)	15(2)
C(2)	67(3)	71(3)	50(2)	28(2)	9(2)	10(2)
C(3)	63(3)	65(3)	45(2)	12(2)	16(2)	7(2)
C(4)	59(3)	76(3)	58(3)	15(2)	21(2)	28(2)
C(5)	53(2)	67(3)	48(2)	20(2)	10(2)	24(2)
C(6)	42(2)	41(2)	44(2)	13(2)	10(2)	6(2)
C(7)	39(2)	43(2)	44(2)	18(2)	10(1)	11(2)
C(8)	43(2)	42(2)	54(2)	23(2)	21(2)	15(2)

C(9)	34(2)	35(2)	47(2)	13(2)	13(1)	8(1)
C(10)	30(2)	45(2)	48(2)	15(2)	10(1)	7(1)
C(11)	46(2)	50(2)	46(2)	14(2)	10(2)	15(2)
C(12)	51(2)	74(3)	51(2)	25(2)	17(2)	22(2)
C(13)	77(3)	97(4)	64(3)	42(3)	31(3)	38(3)
C(14)	115(5)	96(4)	57(3)	27(3)	36(3)	41(4)
C(15)	102(4)	95(4)	48(3)	12(3)	-10(3)	10(3)
C(16)	60(3)	78(3)	53(3)	15(2)	3(2)	7(2)
C(17)	46(2)	41(2)	49(2)	13(2)	14(2)	13(2)
C(18)	59(2)	45(2)	74(3)	13(2)	17(2)	22(2)
C(19)	45(2)	63(3)	76(3)	21(2)	17(2)	26(2)
C(20)	37(2)	52(2)	53(2)	19(2)	12(2)	14(2)
C(21)	38(2)	44(2)	45(2)	16(2)	13(2)	14(2)
C(22)	40(2)	42(2)	40(2)	15(2)	16(1)	14(2)
C(23)	48(2)	38(2)	57(2)	15(2)	21(2)	8(2)
C(24)	177(9)	153(7)	140(7)	102(6)	73(6)	69(7)
C(25)	134(7)	142(7)	177(8)	98(6)	92(6)	41(5)
C(26)	375(19)	174(10)	96(4)	-7(6)	-53(8)	179(12)
C(27)	166(9)	200(10)	112(4)	30(6)	27(6)	96(8)
C(28)	61(2)	51(2)	55(2)	26(2)	11(2)	22(2)
C(29)	95(4)	125(5)	51(3)	24(3)	25(3)	16(4)

Supplementary Table 21. Hydrogen coordinates (x10^4) and isotropic displacement parameters (Å^{2}x10^3) for major-**5o**.

	x	y	z	U(eq)
H(1A)	-174	3583	7397	63
H(2A)	-732	4097	9151	74
H(4A)	-3916	5453	8059	77
H(5A)	-3385	4939	6290	66
H(7A)	-383	3977	5524	49
H(8A)	-2860	2265	4926	51
H(8B)	-1306	2111	5347	51
H(10A)	-3357	2689	3031	49
H(12A)	-115	3208	2090	67
H(13A)	35	2769	227	87

H(14A)	-1889	1710	-1202	103
H(15A)	-3982	1130	-775	107
H(16A)	-4198	1632	1136	81
H(18A)	1056	-528	2959	72
H(19A)	3199	756	3493	73
H(21A)	1136	3398	4205	50
H(24A)	-3232	-1375	3915	163
H(24B)	-3833	-2668	3201	163
H(25A)	-5559	-1846	4013	198
H(25B)	-6024	-2226	2685	198
H(25C)	-5421	-939	3418	198
H(26A)	-1535	-344	630	276
H(26B)	-3174	-678	487	276
H(27A)	-2344	-895	-1190	241
H(27B)	-3407	-1949	-1218	241
H(27C)	-1769	-1961	-1048	241
H(28A)	-2239	4622	2436	79
H(28B)	-2145	5516	3649	79
H(28C)	-3467	4521	3085	79
H(29A)	-2117	4894	10457	137
H(29B)	-3722	4777	9898	137
H(29C)	-2684	5952	10285	137

Supplementary Table 22 Torsion angles [deg] for major-**5o**.

O(2)-P(1)-O(1)-C(26)	-142.4(19)
O(3)-P(1)-O(1)-C(26)	89.0(19)
N(1)-P(1)-O(1)-C(26)	-23(2)
O(2)-P(1)-O(3)-C(24)	46.0(6)
O(1)-P(1)-O(3)-C(24)	167.5(6)
N(1)-P(1)-O(3)-C(24)	-81.5(5)
C(7)-O(4)-N(2)-C(28)	-172.6(3)
C(7)-O(4)-N(2)-C(10)	72.4(3)
O(2)-P(1)-N(1)-C(23)	-155.0(5)
O(1)-P(1)-N(1)-C(23)	86.6(6)
O(3)-P(1)-N(1)-C(23)	-22.5(4)
O(2)-P(1)-N(1)-C(17)	35.9(5)
O(1)-P(1)-N(1)-C(17)	-82.4(6)

O(3)-P(1)-N(1)-C(17)	168.4(3)
C(6)-C(1)-C(2)-C(3)	-0.3(7)
C(1)-C(2)-C(3)-C(4)	-1.2(7)
C(1)-C(2)-C(3)-C(29)	179.2(5)
C(2)-C(3)-C(4)-C(5)	1.3(7)
C(29)-C(3)-C(4)-C(5)	-179.1(5)
C(3)-C(4)-C(5)-C(6)	0.0(7)
C(4)-C(5)-C(6)-C(1)	-1.5(6)
C(4)-C(5)-C(6)-C(7)	176.1(4)
C(2)-C(1)-C(6)-C(5)	1.6(6)
C(2)-C(1)-C(6)-C(7)	-176.0(4)
N(2)-O(4)-C(7)-C(6)	172.4(3)
N(2)-O(4)-C(7)-C(8)	-67.1(3)
C(5)-C(6)-C(7)-O(4)	13.2(5)
C(1)-C(6)-C(7)-O(4)	-169.3(3)
C(5)-C(6)-C(7)-C(8)	-105.8(4)
C(1)-C(6)-C(7)-C(8)	71.7(4)
O(4)-C(7)-C(8)-C(9)	55.5(4)
C(6)-C(7)-C(8)-C(9)	174.1(3)
C(7)-C(8)-C(9)-C(22)	85.1(4)
C(7)-C(8)-C(9)-C(23)	-164.6(3)
C(7)-C(8)-C(9)-C(10)	-48.3(4)
C(28)-N(2)-C(10)-C(11)	60.2(4)
O(4)-N(2)-C(10)-C(11)	169.2(3)
C(28)-N(2)-C(10)-C(9)	-173.8(3)
O(4)-N(2)-C(10)-C(9)	-64.7(3)
C(22)-C(9)-C(10)-N(2)	-75.2(4)
C(23)-C(9)-C(10)-N(2)	169.2(3)
C(8)-C(9)-C(10)-N(2)	53.7(3)
C(22)-C(9)-C(10)-C(11)	49.5(4)
C(23)-C(9)-C(10)-C(11)	-66.1(4)
C(8)-C(9)-C(10)-C(11)	178.3(3)
N(2)-C(10)-C(11)-C(16)	-133.0(4)
C(9)-C(10)-C(11)-C(16)	102.8(4)
N(2)-C(10)-C(11)-C(12)	45.3(5)
C(9)-C(10)-C(11)-C(12)	-78.9(5)
C(16)-C(11)-C(12)-C(13)	0.7(7)
C(10)-C(11)-C(12)-C(13)	-177.6(4)

C(11)-C(12)-C(13)-C(14)	-1.5(8)
C(12)-C(13)-C(14)-C(15)	0.9(9)
C(13)-C(14)-C(15)-C(16)	0.5(9)
C(12)-C(11)-C(16)-C(15)	0.7(7)
C(10)-C(11)-C(16)-C(15)	179.0(4)
C(14)-C(15)-C(16)-C(11)	-1.3(9)
C(23)-N(1)-C(17)-C(22)	-0.1(4)
P(1)-N(1)-C(17)-C(22)	170.6(3)
C(23)-N(1)-C(17)-C(18)	178.6(4)
P(1)-N(1)-C(17)-C(18)	-10.7(6)
C(22)-C(17)-C(18)-C(19)	-2.3(7)
N(1)-C(17)-C(18)-C(19)	179.1(4)
C(17)-C(18)-C(19)-C(20)	1.7(7)
C(18)-C(19)-C(20)-C(21)	-0.6(7)
C(18)-C(19)-C(20)-Br(1)	179.1(4)
C(19)-C(20)-C(21)-C(22)	0.0(6)
Br(1)-C(20)-C(21)-C(22)	-179.8(3)
C(18)-C(17)-C(22)-C(21)	1.7(6)
N(1)-C(17)-C(22)-C(21)	-179.5(3)
C(18)-C(17)-C(22)-C(9)	-173.6(4)
N(1)-C(17)-C(22)-C(9)	5.1(4)
C(20)-C(21)-C(22)-C(17)	-0.5(5)
C(20)-C(21)-C(22)-C(9)	173.7(4)
C(23)-C(9)-C(22)-C(17)	-7.5(4)
C(10)-C(9)-C(22)-C(17)	-126.3(3)
C(8)-C(9)-C(22)-C(17)	106.0(3)
C(23)-C(9)-C(22)-C(21)	177.9(4)
C(10)-C(9)-C(22)-C(21)	59.1(5)
C(8)-C(9)-C(22)-C(21)	-68.6(5)
C(17)-N(1)-C(23)-O(5)	175.8(4)
P(1)-N(1)-C(23)-O(5)	5.4(6)
C(17)-N(1)-C(23)-C(9)	-4.8(4)
P(1)-N(1)-C(23)-C(9)	-175.2(3)
C(22)-C(9)-C(23)-O(5)	-173.2(4)
C(10)-C(9)-C(23)-O(5)	-46.6(5)
C(8)-C(9)-C(23)-O(5)	70.2(5)
C(22)-C(9)-C(23)-N(1)	7.4(4)
C(10)-C(9)-C(23)-N(1)	134.0(3)

C(8)-C(9)-C(23)-N(1)	-109.2(3)
P(1)-O(3)-C(24)-C(25)	153.5(6)
P(1)-O(1)-C(26)-C(27)	-174.2(9)

Symmetry transformations used to generate equivalent atoms:

Supplementary Table 23. Hydrogen bonds for major-**5o** [Å and deg.].

D-H...A	d(D-H)	d(H...A)	d(D...A)	<(DHA)

Tables for Single-Crystal X-ray Crystallography of enantiomer enriched 4e

Supplementary Table 24. Crystal data and structure refinement for enriched **4e**.

Identification code	enriched **4e**	
Empirical formula	$C_{32}H_{29}ClN_2O_4S$	
Formula weight	573.08	
Temperature	293(2) K	
Wavelength	0.71073 Å	
Crystal system	Orthorhombic	
Space group	P 21 21 21	
Unit cell dimensions	a = 8.9464(9) Å	α = 90°.
	b = 16.7038(15) Å	β = 90°.
	c = 19.5947(18) Å	γ = 90°.
Volume	2928.2(5) $Å^3$	
Z	4	
Density (calculated)	1.300 Mg/m^3	
Absorption coefficient	0.241 mm^{-1}	
F(000)	1200	
Crystal size	0.170 x 0.130 x 0.100 mm^3	
Theta range for data collection	1.602 to 25.988°.	
Index ranges	-11<=h<=10, -20<=k<=20, -20<=l<=24	
Reflections collected	17637	
Independent reflections	5749 [R(int) = 0.0446]	
Completeness to theta = 25.242°	100.0 %	

Absorption correction	Semi-empirical from equivalents
Max. and min. transmission	0.7456 and 0.6439
Refinement method	Full-matrix least-squares on F^2
Data / restraints / parameters	5749 / 0 / 364
Goodness-of-fit on F^2	1.047
Final R indices [I>2sigma(I)]	R_1 = 0.0529, wR_2 = 0.1206
R indices (all data)	R_1 = 0.0670, wR_2 = 0.1283
Absolute structure parameter	0.02(4)
Extinction coefficient	n/a
Largest diff. peak and hole	0.329 and -0.205 e.Å^{-3}

Supplementary Table 25. Atomic coordinates (x10^4) and equivalent isotropic displacement parameters (Å^{2}x10^3) for enriched **4e**. U(eq) is defined as one third of the trace of the orthogonalized U^{ij} tensor.

	x	y	z	U(eq)
S(1)	327(1)	2418(1)	1374(1)	45(1)
Cl(1)	-256(2)	5110(1)	4199(1)	87(1)
N(1)	530(4)	3381(2)	1619(2)	46(1)
N(2)	-866(4)	6006(2)	1445(2)	46(1)
O(1)	1325(4)	3903(2)	588(2)	54(1)
O(2)	444(3)	6449(2)	1200(2)	50(1)
O(3)	-246(4)	2430(2)	704(1)	58(1)
O(4)	-495(4)	2051(2)	1905(2)	57(1)
C(1)	307(5)	3698(2)	2287(2)	45(1)
C(2)	56(6)	3298(2)	2888(2)	59(1)
C(3)	-132(6)	3745(3)	3473(2)	65(1)
C(4)	-31(6)	4570(2)	3443(2)	55(1)
C(5)	233(5)	4965(2)	2847(2)	49(1)
C(6)	399(5)	4528(2)	2256(2)	42(1)
C(7)	682(4)	4792(2)	1529(2)	41(1)
C(8)	912(5)	3990(2)	1160(2)	43(1)

C(9)	2050(5)	5325(2)	1420(2)	47(1)
C(10)	1720(5)	6200(2)	1580(2)	46(1)
C(11)	-707(5)	5187(2)	1178(2)	41(1)
C(12)	-2132(5)	4715(2)	1271(2)	43(1)
C(13)	-2941(5)	4735(3)	1872(2)	52(1)
C(14)	-4234(6)	4304(3)	1939(3)	65(1)
C(15)	-4746(6)	3830(3)	1414(3)	72(1)
C(16)	-3949(6)	3809(3)	813(3)	74(2)
C(17)	-2664(5)	4249(3)	745(2)	56(1)
C(18)	2949(5)	6773(2)	1386(2)	48(1)
C(19)	3536(7)	6810(3)	739(3)	78(2)
C(20)	4552(7)	7397(4)	561(3)	81(2)
C(21)	5029(7)	7944(3)	1011(3)	74(2)
C(22)	4521(9)	7869(4)	1667(3)	107(2)
C(23)	3500(8)	7303(3)	1854(3)	85(2)
C(24)	6108(9)	8593(4)	817(4)	119(3)
C(25)	-2091(6)	6438(3)	1110(3)	63(1)
C(26)	2135(5)	2034(2)	1363(2)	42(1)
C(27)	2682(6)	1659(3)	1946(2)	58(1)
C(28)	4087(6)	1329(3)	1921(3)	70(1)
C(29)	4957(5)	1355(2)	1341(3)	61(1)
C(30)	4390(5)	1747(2)	779(2)	56(1)
C(31)	2987(5)	2084(2)	783(2)	51(1)
C(32)	6476(6)	979(4)	1321(4)	91(2)

Supplementary Table 26. Bond lengths [Å] and angles [°] for enriched **4e**.

S(1)-O(3)	1.409(3)
S(1)-O(4)	1.415(3)
S(1)-N(1)	1.689(3)
S(1)-C(26)	1.740(4)
Cl(1)-C(4)	1.746(4)
N(1)-C(8)	1.399(5)
N(1)-C(1)	1.426(5)
N(2)-C(25)	1.467(5)
N(2)-O(2)	1.466(4)
N(2)-C(11)	1.472(5)
O(1)-C(8)	1.190(5)

O(2)-C(10)	1.424(5)
C(1)-C(2)	1.373(5)
C(1)-C(6)	1.391(5)
C(2)-C(3)	1.377(6)
C(2)-H(2)	0.9300
C(3)-C(4)	1.383(6)
C(3)-H(3)	0.9300
C(4)-C(5)	1.360(6)
C(5)-C(6)	1.377(5)
C(5)-H(5)	0.9300
C(6)-C(7)	1.512(5)
C(7)-C(9)	1.528(5)
C(7)-C(8)	1.536(5)
C(7)-C(11)	1.566(6)
C(9)-C(10)	1.525(5)
C(9)-H(9A)	0.9700
C(9)-H(9B)	0.9700
C(10)-C(18)	1.506(6)
C(10)-H(10)	0.9800
C(11)-C(12)	1.510(6)
C(11)-H(11)	0.9800
C(12)-C(17)	1.376(6)
C(12)-C(13)	1.384(6)
C(13)-C(14)	1.369(7)
C(13)-H(13)	0.9300
C(14)-C(15)	1.376(7)
C(14)-H(14)	0.9300
C(15)-C(16)	1.376(8)
C(15)-H(15)	0.9300
C(16)-C(17)	1.372(7)
C(16)-H(16)	0.9300
C(17)-H(17)	0.9300
C(18)-C(23)	1.368(7)
C(18)-C(19)	1.374(7)
C(19)-C(20)	1.383(8)
C(19)-H(19)	0.9300
C(20)-C(21)	1.339(8)
C(20)-H(20)	0.9300

C(21)-C(22)	1.370(8)
C(21)-C(24)	1.501(8)
C(22)-C(23)	1.365(8)
C(22)-H(22)	0.9300
C(23)-H(23)	0.9300
C(24)-H(24A)	0.9600
C(24)-H(24B)	0.9600
C(24)-H(24C)	0.9600
C(25)-H(25A)	0.9600
C(25)-H(25B)	0.9600
C(25)-H(25C)	0.9600
C(26)-C(31)	1.371(6)
C(26)-C(27)	1.390(6)
C(27)-C(28)	1.374(7)
C(27)-H(27)	0.9300
C(28)-C(29)	1.378(7)
C(28)-H(28)	0.9300
C(29)-C(30)	1.379(7)
C(29)-C(32)	1.497(7)
C(30)-C(31)	1.375(7)
C(30)-H(30)	0.9300
C(31)-H(31)	0.9300
C(32)-H(32A)	0.9600
C(32)-H(32B)	0.9600
C(32)-H(32C)	0.9600
O(3)-S(1)-O(4)	120.2(2)
O(3)-S(1)-N(1)	106.82(16)
O(4)-S(1)-N(1)	105.02(17)
O(3)-S(1)-C(26)	109.5(2)
O(4)-S(1)-C(26)	109.4(2)
N(1)-S(1)-C(26)	104.73(19)
C(8)-N(1)-C(1)	110.7(3)
C(8)-N(1)-S(1)	122.5(3)
C(1)-N(1)-S(1)	126.8(3)
C(25)-N(2)-O(2)	101.7(3)
C(25)-N(2)-C(11)	111.7(3)
O(2)-N(2)-C(11)	106.0(3)
C(10)-O(2)-N(2)	108.9(3)

C(2)-C(1)-C(6)	122.1(4)
C(2)-C(1)-N(1)	129.1(3)
C(6)-C(1)-N(1)	108.8(3)
C(1)-C(2)-C(3)	118.0(4)
C(1)-C(2)-H(2)	121.0
C(3)-C(2)-H(2)	121.0
C(4)-C(3)-C(2)	119.8(4)
C(4)-C(3)-H(3)	120.1
C(2)-C(3)-H(3)	120.1
C(5)-C(4)-C(3)	122.0(4)
C(5)-C(4)-Cl(1)	119.8(3)
C(3)-C(4)-Cl(1)	118.1(3)
C(4)-C(5)-C(6)	118.9(4)
C(4)-C(5)-H(5)	120.5
C(6)-C(5)-H(5)	120.5
C(5)-C(6)-C(1)	119.0(4)
C(5)-C(6)-C(7)	131.0(3)
C(1)-C(6)-C(7)	110.0(3)
C(6)-C(7)-C(9)	115.8(3)
C(6)-C(7)-C(8)	102.2(3)
C(9)-C(7)-C(8)	109.5(3)
C(6)-C(7)-C(11)	113.9(3)
C(9)-C(7)-C(11)	109.2(3)
C(8)-C(7)-C(11)	105.5(3)
O(1)-C(8)-N(1)	126.3(4)
O(1)-C(8)-C(7)	126.3(4)
N(1)-C(8)-C(7)	107.4(3)
C(10)-C(9)-C(7)	112.0(3)
C(10)-C(9)-H(9A)	109.2
C(7)-C(9)-H(9A)	109.2
C(10)-C(9)-H(9B)	109.2
C(7)-C(9)-H(9B)	109.2
H(9A)-C(9)-H(9B)	107.9
O(2)-C(10)-C(18)	105.6(3)
O(2)-C(10)-C(9)	109.1(3)
C(18)-C(10)-C(9)	114.6(3)
O(2)-C(10)-H(10)	109.1
C(18)-C(10)-H(10)	109.1

C(9)-C(10)-H(10)	109.1
N(2)-C(11)-C(12)	111.2(3)
N(2)-C(11)-C(7)	108.2(3)
C(12)-C(11)-C(7)	113.4(3)
N(2)-C(11)-H(11)	108.0
C(12)-C(11)-H(11)	108.0
C(7)-C(11)-H(11)	108.0
C(17)-C(12)-C(13)	118.1(4)
C(17)-C(12)-C(11)	119.8(4)
C(13)-C(12)-C(11)	122.1(4)
C(14)-C(13)-C(12)	120.7(4)
C(14)-C(13)-H(13)	119.7
C(12)-C(13)-H(13)	119.7
C(13)-C(14)-C(15)	120.8(5)
C(13)-C(14)-H(14)	119.6
C(15)-C(14)-H(14)	119.6
C(16)-C(15)-C(14)	118.7(5)
C(16)-C(15)-H(15)	120.6
C(14)-C(15)-H(15)	120.6
C(17)-C(16)-C(15)	120.3(5)
C(17)-C(16)-H(16)	119.8
C(15)-C(16)-H(16)	119.8
C(16)-C(17)-C(12)	121.3(5)
C(16)-C(17)-H(17)	119.4
C(12)-C(17)-H(17)	119.4
C(23)-C(18)-C(19)	116.9(4)
C(23)-C(18)-C(10)	120.3(4)
C(19)-C(18)-C(10)	122.7(4)
C(18)-C(19)-C(20)	121.1(5)
C(18)-C(19)-H(19)	119.5
C(20)-C(19)-H(19)	119.5
C(21)-C(20)-C(19)	121.8(5)
C(21)-C(20)-H(20)	119.1
C(19)-C(20)-H(20)	119.1
C(20)-C(21)-C(22)	116.8(5)
C(20)-C(21)-C(24)	122.1(6)
C(22)-C(21)-C(24)	121.1(6)
C(23)-C(22)-C(21)	122.5(5)

C(23)-C(22)-H(22)	118.7
C(21)-C(22)-H(22)	118.7
C(22)-C(23)-C(18)	120.6(5)
C(22)-C(23)-H(23)	119.7
C(18)-C(23)-H(23)	119.7
C(21)-C(24)-H(24A)	109.5
C(21)-C(24)-H(24B)	109.5
H(24A)-C(24)-H(24B)	109.5
C(21)-C(24)-H(24C)	109.5
H(24A)-C(24)-H(24C)	109.5
H(24B)-C(24)-H(24C)	109.5
N(2)-C(25)-H(25A)	109.5
N(2)-C(25)-H(25B)	109.5
H(25A)-C(25)-H(25B)	109.5
N(2)-C(25)-H(25C)	109.5
H(25A)-C(25)-H(25C)	109.5
H(25B)-C(25)-H(25C)	109.5
C(31)-C(26)-C(27)	120.8(4)
C(31)-C(26)-S(1)	120.3(3)
C(27)-C(26)-S(1)	118.9(3)
C(28)-C(27)-C(26)	118.3(5)
C(28)-C(27)-H(27)	120.9
C(26)-C(27)-H(27)	120.9
C(29)-C(28)-C(27)	122.2(5)
C(29)-C(28)-H(28)	118.9
C(27)-C(28)-H(28)	118.9
C(28)-C(29)-C(30)	117.8(4)
C(28)-C(29)-C(32)	121.4(5)
C(30)-C(29)-C(32)	120.8(5)
C(31)-C(30)-C(29)	121.7(4)
C(31)-C(30)-H(30)	119.2
C(29)-C(30)-H(30)	119.2
C(26)-C(31)-C(30)	119.2(4)
C(26)-C(31)-H(31)	120.4
C(30)-C(31)-H(31)	120.4
C(29)-C(32)-H(32A)	109.5
C(29)-C(32)-H(32B)	109.5
H(32A)-C(32)-H(32B)	109.5

C(29)-C(32)-H(32C)	109.5
H(32A)-C(32)-H(32C)	109.5
H(32B)-C(32)-H(32C)	109.5

Symmetry transformations used to generate equivalent atoms:

Supplementary Table 27. Anisotropic displacement parameters ($Å^2$x 10^3) for enriched **4e**. The anisotropicdispl acement factor exponent takes the form: $-2\pi^2[h^2 a^{*2}U^{11} + ... + 2 h k a^* b^* U^{12}]$

	U^{11}	U^{22}	U^{33}	U^{23}	U^{13}	U^{12}
S(1)	51(1)	31(1)	52(1)	-2(1)	-1(1)	-1(1)
Cl(1)	135(1)	77(1)	49(1)	-14(1)	11(1)	-2(1)
N(1)	62(2)	30(1)	48(2)	-1(1)	1(2)	1(2)
N(2)	44(2)	33(2)	61(2)	1(2)	-1(2)	3(1)
O(1)	66(2)	46(2)	50(2)	-6(1)	6(2)	0(1)
O(2)	51(2)	36(1)	65(2)	8(1)	1(2)	-4(1)
O(3)	65(2)	45(1)	63(2)	-6(1)	-14(2)	-2(2)
O(4)	60(2)	38(1)	72(2)	2(1)	12(2)	-5(1)
C(1)	49(2)	40(2)	45(2)	0(2)	-3(2)	4(2)
C(2)	84(4)	38(2)	55(3)	8(2)	-1(2)	4(2)
C(3)	89(4)	62(2)	44(2)	10(2)	2(2)	2(3)
C(4)	71(3)	51(2)	44(2)	-5(2)	0(2)	0(2)
C(5)	56(3)	40(2)	50(2)	-6(2)	-5(2)	-2(2)
C(6)	41(2)	38(2)	46(2)	2(2)	-6(2)	2(2)
C(7)	43(2)	35(2)	43(2)	0(2)	-2(2)	1(2)
C(8)	44(2)	36(2)	49(3)	0(2)	-5(2)	1(2)
C(9)	46(2)	42(2)	54(2)	3(2)	1(2)	-1(2)
C(10)	50(3)	40(2)	49(2)	0(2)	-2(2)	-3(2)
C(11)	46(2)	39(2)	39(2)	-1(2)	-4(2)	0(2)
C(12)	43(2)	40(2)	45(2)	2(2)	-7(2)	5(2)
C(13)	49(3)	50(2)	58(3)	-6(2)	-4(2)	0(2)
C(14)	57(3)	68(3)	70(3)	6(2)	8(3)	-3(2)
C(15)	57(3)	59(3)	100(4)	-3(3)	-4(3)	-9(2)
C(16)	69(4)	64(3)	88(4)	-20(3)	-13(3)	-14(3)
C(17)	56(3)	55(2)	58(3)	-11(2)	-4(2)	-3(2)
C(18)	54(3)	39(2)	52(2)	2(2)	-3(2)	-3(2)

C(19)	91(4)	87(4)	55(3)	-3(3)	-1(3)	-41(3)
C(20)	87(4)	96(4)	58(3)	16(3)	6(3)	-24(4)
C(21)	73(4)	51(2)	96(4)	4(3)	14(3)	-13(3)
C(22)	124(6)	88(4)	109(5)	-52(4)	41(4)	-58(4)
C(23)	102(5)	82(4)	71(3)	-27(3)	31(3)	-38(3)
C(24)	110(6)	93(5)	155(7)	0(5)	33(5)	-45(4)
C(25)	56(3)	45(2)	90(4)	10(2)	-7(3)	8(2)
C(26)	51(2)	26(2)	48(2)	-3(2)	1(2)	-2(2)
C(27)	61(3)	63(3)	51(3)	12(2)	5(2)	4(2)
C(28)	67(3)	78(3)	63(3)	23(3)	-10(3)	5(3)
C(29)	52(3)	52(2)	80(3)	-2(2)	0(3)	-2(2)
C(30)	58(3)	51(2)	59(3)	-7(2)	6(2)	-2(2)
C(31)	64(3)	47(2)	42(2)	-1(2)	-1(2)	-2(2)
C(32)	56(3)	89(4)	128(5)	4(4)	0(4)	10(3)

Supplementary Table 28. Hydrogen coordinates ($x10^4$) and isotropic displacement parameters ($Å^2 x10^3$) for enriched **4e**.

	x	y	z	U(eq)
H(2)	15	2742	2901	71
H(3)	-326	3492	3886	78
H(5)	300	5520	2840	58
H(9A)	2857	5140	1710	57
H(9B)	2377	5279	950	57
H(10)	1509	6255	2068	56
H(11)	-502	5224	687	50
H(13)	-2603	5045	2235	63
H(14)	-4774	4331	2344	78
H(15)	-5614	3529	1464	86
H(16)	-4284	3496	452	88
H(17)	-2142	4233	335	68
H(19)	3245	6433	415	93
H(20)	4915	7414	116	97
H(22)	4884	8218	1998	128
H(23)	3177	7277	2305	102

H(24A)	6745	8407	456	179
H(24B)	6706	8733	1206	179
H(24C)	5564	9055	665	179
H(25A)	-2051	6993	1235	95
H(25B)	-3030	6215	1253	95
H(25C)	-1995	6388	624	95
H(27)	2111	1633	2342	70
H(28)	4464	1079	2309	83
H(30)	4971	1786	387	67
H(31)	2620	2341	397	61
H(32A)	6681	793	867	137
H(32B)	6508	536	1632	137
H(32C)	7214	1368	1451	137

Supplementary Table 29. Torsion angles [°] for enriched **4e**.

O(3)-S(1)-N(1)-C(8)	-33.3(4)
O(4)-S(1)-N(1)-C(8)	-162.0(4)
C(26)-S(1)-N(1)-C(8)	82.7(4)
O(3)-S(1)-N(1)-C(1)	144.7(4)
O(4)-S(1)-N(1)-C(1)	16.0(4)
C(26)-S(1)-N(1)-C(1)	-99.3(4)
C(25)-N(2)-O(2)-C(10)	169.1(3)
C(11)-N(2)-O(2)-C(10)	-73.9(4)
C(8)-N(1)-C(1)-C(2)	-173.5(5)
S(1)-N(1)-C(1)-C(2)	8.3(7)
C(8)-N(1)-C(1)-C(6)	5.8(5)
S(1)-N(1)-C(1)-C(6)	-172.4(3)
C(6)-C(1)-C(2)-C(3)	1.1(8)
N(1)-C(1)-C(2)-C(3)	-179.8(5)
C(1)-C(2)-C(3)-C(4)	-1.5(8)
C(2)-C(3)-C(4)-C(5)	0.8(8)
C(2)-C(3)-C(4)-Cl(1)	-179.3(4)
C(3)-C(4)-C(5)-C(6)	0.2(8)
Cl(1)-C(4)-C(5)-C(6)	-179.7(4)
C(4)-C(5)-C(6)-C(1)	-0.6(7)
C(4)-C(5)-C(6)-C(7)	179.4(4)

C(2)-C(1)-C(6)-C(5)	0.0(7)
N(1)-C(1)-C(6)-C(5)	-179.3(4)
C(2)-C(1)-C(6)-C(7)	180.0(4)
N(1)-C(1)-C(6)-C(7)	0.7(5)
C(5)-C(6)-C(7)-C(9)	55.0(6)
C(1)-C(6)-C(7)-C(9)	-125.0(4)
C(5)-C(6)-C(7)-C(8)	174.0(5)
C(1)-C(6)-C(7)-C(8)	-6.0(5)
C(5)-C(6)-C(7)-C(11)	-72.8(6)
C(1)-C(6)-C(7)-C(11)	107.2(4)
C(1)-N(1)-C(8)-O(1)	171.1(4)
S(1)-N(1)-C(8)-O(1)	-10.6(6)
C(1)-N(1)-C(8)-C(7)	-9.6(5)
S(1)-N(1)-C(8)-C(7)	168.7(3)
C(6)-C(7)-C(8)-O(1)	-171.4(4)
C(9)-C(7)-C(8)-O(1)	-48.1(6)
C(11)-C(7)-C(8)-O(1)	69.3(5)
C(6)-C(7)-C(8)-N(1)	9.3(4)
C(9)-C(7)-C(8)-N(1)	132.6(4)
C(11)-C(7)-C(8)-N(1)	-110.0(4)
C(6)-C(7)-C(9)-C(10)	-82.5(4)
C(8)-C(7)-C(9)-C(10)	162.7(3)
C(11)-C(7)-C(9)-C(10)	47.6(5)
N(2)-O(2)-C(10)-C(18)	-170.7(3)
N(2)-O(2)-C(10)-C(9)	65.7(4)
C(7)-C(9)-C(10)-O(2)	-53.4(5)
C(7)-C(9)-C(10)-C(18)	-171.5(4)
C(25)-N(2)-C(11)-C(12)	-58.4(4)
O(2)-N(2)-C(11)-C(12)	-168.4(3)
C(25)-N(2)-C(11)-C(7)	176.5(3)
O(2)-N(2)-C(11)-C(7)	66.5(4)
C(6)-C(7)-C(11)-N(2)	76.3(4)
C(9)-C(7)-C(11)-N(2)	-54.9(4)
C(8)-C(7)-C(11)-N(2)	-172.5(3)
C(6)-C(7)-C(11)-C(12)	-47.5(4)
C(9)-C(7)-C(11)-C(12)	-178.7(3)
C(8)-C(7)-C(11)-C(12)	63.7(4)
N(2)-C(11)-C(12)-C(17)	135.7(4)

C(7)-C(11)-C(12)-C(17)	-102.2(4)
N(2)-C(11)-C(12)-C(13)	-44.0(5)
C(7)-C(11)-C(12)-C(13)	78.1(5)
C(17)-C(12)-C(13)-C(14)	-0.1(6)
C(11)-C(12)-C(13)-C(14)	179.6(4)
C(12)-C(13)-C(14)-C(15)	1.1(7)
C(13)-C(14)-C(15)-C(16)	-1.3(8)
C(14)-C(15)-C(16)-C(17)	0.5(8)
C(15)-C(16)-C(17)-C(12)	0.5(8)
C(13)-C(12)-C(17)-C(16)	-0.7(7)
C(11)-C(12)-C(17)-C(16)	179.6(4)
O(2)-C(10)-C(18)-C(23)	112.3(5)
C(9)-C(10)-C(18)-C(23)	-127.6(5)
O(2)-C(10)-C(18)-C(19)	-65.7(5)
C(9)-C(10)-C(18)-C(19)	54.4(6)
C(23)-C(18)-C(19)-C(20)	-4.8(9)
C(10)-C(18)-C(19)-C(20)	173.2(5)
C(18)-C(19)-C(20)-C(21)	1.3(10)
C(19)-C(20)-C(21)-C(22)	3.2(10)
C(19)-C(20)-C(21)-C(24)	-178.7(6)
C(20)-C(21)-C(22)-C(23)	-4.3(11)
C(24)-C(21)-C(22)-C(23)	177.7(7)
C(21)-C(22)-C(23)-C(18)	0.7(12)
C(19)-C(18)-C(23)-C(22)	3.8(10)
C(10)-C(18)-C(23)-C(22)	-174.3(6)
O(3)-S(1)-C(26)-C(31)	24.9(4)
O(4)-S(1)-C(26)-C(31)	158.5(3)
N(1)-S(1)-C(26)-C(31)	-89.3(3)
O(3)-S(1)-C(26)-C(27)	-153.4(3)
O(4)-S(1)-C(26)-C(27)	-19.7(4)
N(1)-S(1)-C(26)-C(27)	92.4(3)
C(31)-C(26)-C(27)-C(28)	-1.0(7)
S(1)-C(26)-C(27)-C(28)	177.2(4)
C(26)-C(27)-C(28)-C(29)	-0.3(8)
C(27)-C(28)-C(29)-C(30)	1.7(8)
C(27)-C(28)-C(29)-C(32)	-179.0(5)
C(28)-C(29)-C(30)-C(31)	-1.9(7)
C(32)-C(29)-C(30)-C(31)	178.8(5)

C(27)-C(26)-C(31)-C(30)	0.9(6)
S(1)-C(26)-C(31)-C(30)	-177.4(3)
C(29)-C(30)-C(31)-C(26)	0.6(6)

Symmetry transformations used to generate equivalent atoms:

Supplementary Table 30. Hydrogen bonds for enriched **4e** [Å and °].

D-H...A	d(D-H)	d(H...A)	d(D...A)	<(DHA)

Tables for Single-Crystal X-ray Crystallography of 12a

Supplementary Table 31. Crystal data and structure refinement for **12a**.

Identification code	**12a**	
Empirical formula	$C_{33}H_{31}ClN_2O_4S$	
Formula weight	587.11	
Temperature	296 K	
Wavelength	0.71073 Å	
Crystal system	Orthorhombic	
Space group	P 21 21 21	
Unit cell dimensions	a = 8.9148(6) Å	α = 90°.
	b = 16.9063(12) Å	β = 90°.
	c = 19.8682(14) Å	γ = 90°.
Volume	2994.5(4) Å^3	
Z	4	
Density (calculated)	1.302 Mg/m^3	
Absorption coefficient	0.238 mm^{-1}	
F(000)	1232	
Crystal size	0.33 x 0.28 x 0.25 mm^3	
Theta range for data collection	2.050 to 30.579°.	
Index ranges	-12<=h<=11, -24<=k<=24, -28<=l<=28	
Reflections collected	30547	
Independent reflections	9195 [R(int) = 0.0370]	

Completeness to theta = 26.000°	100.0 %
Absorption correction	Semi-empirical from equivalents
Max. and min. transmission	0.7461 and 0.6833
Refinement method	Full-matrix least-squares on F^2
Data / restraints / parameters	9195 / 0 / 374
Goodness-of-fit on F^2	1.009
Final R indices [I>2sigma(I)]	R_1 = 0.0438, wR_2 = 0.1001
R indices (all data)	R_1 = 0.0682, wR_2 = 0.1130
Absolute structure parameter	-0.01(2)
Extinction coefficient	n/a
Largest diff. peak and hole	0.238 and -0.311 e.Å^{-3}

Supplementary Table 32. Atomic coordinates (x10^4) and equivalent isotropic displacement parameters (Å^{2}x 10^3) for **12a**. U(eq) is defined as one third of the trace of the orthogonalized U^{ij} tensor.

	x	y	z	U(eq)
Cl(1)	5392(1)	4987(1)	9146(1)	82(1)
S(1)	4714(1)	7641(1)	6338(1)	40(1)
O(1)	4600(2)	3621(1)	6298(1)	46(1)
O(2)	3483(2)	6196(1)	5616(1)	49(1)
O(3)	5284(2)	7613(1)	5669(1)	53(1)
O(4)	5550(2)	8020(1)	6856(1)	52(1)
N(1)	5903(2)	4103(1)	6497(1)	40(1)
N(2)	4539(3)	6693(1)	6593(1)	41(1)
C(1)	3293(3)	3904(2)	6642(1)	42(1)
C(2)	2966(3)	4758(1)	6435(1)	42(1)
C(3)	4359(3)	5291(1)	6506(1)	35(1)
C(4)	5763(3)	4882(1)	6142(1)	36(1)
C(5)	5493(3)	4773(2)	5381(1)	48(1)
C(6)	7155(3)	3613(2)	6248(2)	53(1)
C(7)	4051(3)	6088(1)	6157(1)	38(1)

C(8)	4685(3)	5556(1)	7222(1)	36(1)
C(9)	4855(3)	5126(2)	7811(1)	43(1)
C(10)	5170(3)	5527(2)	8401(1)	50(1)
C(11)	5327(4)	6337(2)	8422(1)	55(1)
C(12)	5126(3)	6780(2)	7838(1)	50(1)
C(13)	4808(3)	6377(1)	7249(1)	38(1)
C(14)	2891(3)	8027(1)	6328(1)	38(1)
C(15)	2388(3)	8446(2)	6885(1)	50(1)
C(16)	977(3)	8788(2)	6860(2)	57(1)
C(17)	77(3)	8726(2)	6295(2)	50(1)
C(18)	603(3)	8293(2)	5753(1)	50(1)
C(19)	2008(3)	7944(2)	5760(1)	45(1)
C(20)	-1439(4)	9118(2)	6264(2)	74(1)
C(21)	2062(3)	3327(2)	6463(1)	44(1)
C(22)	1381(3)	2884(2)	6953(2)	52(1)
C(23)	281(4)	2338(2)	6793(2)	60(1)
C(24)	-141(3)	2197(2)	6140(2)	57(1)
C(25)	517(5)	2651(3)	5647(2)	79(1)
C(26)	1587(4)	3217(3)	5806(2)	77(1)
C(27)	-1321(5)	1588(2)	5978(2)	88(1)
C(28)	7191(3)	5361(1)	6277(1)	38(1)
C(29)	7768(3)	5870(2)	5793(2)	55(1)
C(30)	9052(4)	6309(2)	5918(2)	69(1)
C(31)	9779(4)	6255(2)	6519(2)	66(1)
C(32)	9230(3)	5746(2)	7010(2)	56(1)
C(33)	7951(3)	5309(2)	6889(1)	45(1)

Supplementary Table 33. Bond lengths [Å] and angles [°] for **12a**.

Cl(1)-C(10)	1.751(3)
S(1)-O(3)	1.423(2)
S(1)-O(4)	1.423(2)
S(1)-N(2)	1.689(2)
S(1)-C(14)	1.751(3)
O(1)-N(1)	1.473(3)
O(1)-C(1)	1.433(3)
O(2)-C(7)	1.202(3)

N(1)-C(4)	1.499(3)
N(1)-C(6)	1.476(3)
N(2)-C(7)	1.408(3)
N(2)-C(13)	1.430(3)
C(1)-H(1)	0.9800
C(1)-C(2)	1.528(3)
C(1)-C(21)	1.511(3)
C(2)-H(2A)	0.9700
C(2)-H(2B)	0.9700
C(2)-C(3)	1.541(3)
C(3)-C(4)	1.602(3)
C(3)-C(7)	1.539(3)
C(3)-C(8)	1.520(3)
C(4)-C(5)	1.542(3)
C(4)-C(28)	1.532(3)
C(5)-H(5A)	0.9600
C(5)-H(5B)	0.9600
C(5)-H(5C)	0.9600
C(6)-H(6A)	0.9600
C(6)-H(6B)	0.9600
C(6)-H(6C)	0.9600
C(8)-C(9)	1.387(3)
C(8)-C(13)	1.393(3)
C(9)-H(9)	0.9300
C(9)-C(10)	1.382(4)
C(10)-C(11)	1.376(4)
C(11)-H(11)	0.9300
C(11)-C(12)	1.392(4)
C(12)-H(12)	0.9300
C(12)-C(13)	1.383(3)
C(14)-C(15)	1.389(4)
C(14)-C(19)	1.384(4)
C(15)-H(15)	0.9300
C(15)-C(16)	1.385(4)
C(16)-H(16)	0.9300
C(16)-C(17)	1.384(4)
C(17)-C(18)	1.384(4)
C(17)-C(20)	1.506(4)

C(18)-H(18)	0.9300
C(18)-C(19)	1.385(4)
C(19)-H(19)	0.9300
C(20)-H(20A)	0.9600
C(20)-H(20B)	0.9600
C(20)-H(20C)	0.9600
C(21)-C(22)	1.370(4)
C(21)-C(26)	1.385(4)
C(22)-H(22)	0.9300
C(22)-C(23)	1.384(4)
C(23)-H(23)	0.9300
C(23)-C(24)	1.372(5)
C(24)-C(25)	1.376(5)
C(24)-C(27)	1.507(4)
C(25)-H(25)	0.9300
C(25)-C(26)	1.387(5)
C(26)-H(26)	0.9300
C(27)-H(27A)	0.9600
C(27)-H(27B)	0.9600
C(27)-H(27C)	0.9600
C(28)-C(29)	1.389(4)
C(28)-C(33)	1.395(4)
C(29)-H(29)	0.9300
C(29)-C(30)	1.387(4)
C(30)-H(30)	0.9300
C(30)-C(31)	1.362(5)
C(31)-H(31)	0.9300
C(31)-C(32)	1.390(4)
C(32)-H(32)	0.9300
C(32)-C(33)	1.380(4)
C(33)-H(33)	0.9300
O(3)-S(1)-N(2)	106.31(11)
O(3)-S(1)-C(14)	109.47(13)
O(4)-S(1)-O(3)	120.29(13)
O(4)-S(1)-N(2)	105.00(11)
O(4)-S(1)-C(14)	109.02(12)
N(2)-S(1)-C(14)	105.70(11)
C(1)-O(1)-N(1)	109.12(17)

O(1)-N(1)-C(4)	107.17(17)
O(1)-N(1)-C(6)	101.28(18)
C(6)-N(1)-C(4)	113.5(2)
C(7)-N(2)-S(1)	122.28(17)
C(7)-N(2)-C(13)	109.93(19)
C(13)-N(2)-S(1)	127.77(16)
O(1)-C(1)-H(1)	109.2
O(1)-C(1)-C(2)	110.0(2)
O(1)-C(1)-C(21)	105.2(2)
C(2)-C(1)-H(1)	109.2
C(21)-C(1)-H(1)	109.2
C(21)-C(1)-C(2)	114.0(2)
C(1)-C(2)-H(2A)	109.2
C(1)-C(2)-H(2B)	109.2
C(1)-C(2)-C(3)	112.0(2)
H(2A)-C(2)-H(2B)	107.9
C(3)-C(2)-H(2A)	109.2
C(3)-C(2)-H(2B)	109.2
C(2)-C(3)-C(4)	109.61(18)
C(7)-C(3)-C(2)	109.11(19)
C(7)-C(3)-C(4)	108.30(19)
C(8)-C(3)-C(2)	114.4(2)
C(8)-C(3)-C(4)	113.58(19)
C(8)-C(3)-C(7)	101.36(18)
N(1)-C(4)-C(3)	103.42(18)
N(1)-C(4)-C(5)	111.6(2)
N(1)-C(4)-C(28)	108.21(19)
C(5)-C(4)-C(3)	111.8(2)
C(28)-C(4)-C(3)	110.05(18)
C(28)-C(4)-C(5)	111.3(2)
C(4)-C(5)-H(5A)	109.5
C(4)-C(5)-H(5B)	109.5
C(4)-C(5)-H(5C)	109.5
H(5A)-C(5)-H(5B)	109.5
H(5A)-C(5)-H(5C)	109.5
H(5B)-C(5)-H(5C)	109.5
N(1)-C(6)-H(6A)	109.5
N(1)-C(6)-H(6B)	109.5

N(1)-C(6)-H(6C)	109.5
H(6A)-C(6)-H(6B)	109.5
H(6A)-C(6)-H(6C)	109.5
H(6B)-C(6)-H(6C)	109.5
O(2)-C(7)-N(2)	124.7(2)
O(2)-C(7)-C(3)	127.6(2)
N(2)-C(7)-C(3)	107.7(2)
C(9)-C(8)-C(3)	131.0(2)
C(9)-C(8)-C(13)	118.8(2)
C(13)-C(8)-C(3)	110.2(2)
C(8)-C(9)-H(9)	120.7
C(10)-C(9)-C(8)	118.7(2)
C(10)-C(9)-H(9)	120.7
C(9)-C(10)-Cl(1)	118.9(2)
C(11)-C(10)-Cl(1)	118.8(2)
C(11)-C(10)-C(9)	122.3(2)
C(10)-C(11)-H(11)	120.1
C(10)-C(11)-C(12)	119.8(2)
C(12)-C(11)-H(11)	120.1
C(11)-C(12)-H(12)	121.1
C(13)-C(12)-C(11)	117.8(2)
C(13)-C(12)-H(12)	121.1
C(8)-C(13)-N(2)	108.9(2)
C(12)-C(13)-N(2)	128.5(2)
C(12)-C(13)-C(8)	122.6(2)
C(15)-C(14)-S(1)	118.8(2)
C(19)-C(14)-S(1)	119.9(2)
C(19)-C(14)-C(15)	121.2(2)
C(14)-C(15)-H(15)	120.7
C(16)-C(15)-C(14)	118.5(3)
C(16)-C(15)-H(15)	120.7
C(15)-C(16)-H(16)	119.2
C(17)-C(16)-C(15)	121.6(3)
C(17)-C(16)-H(16)	119.2
C(16)-C(17)-C(18)	118.3(3)
C(16)-C(17)-C(20)	121.4(3)
C(18)-C(17)-C(20)	120.3(3)
C(17)-C(18)-H(18)	119.2

C(17)-C(18)-C(19)	121.6(3)
C(19)-C(18)-H(18)	119.2
C(14)-C(19)-C(18)	118.7(2)
C(14)-C(19)-H(19)	120.7
C(18)-C(19)-H(19)	120.7
C(17)-C(20)-H(20A)	109.5
C(17)-C(20)-H(20B)	109.5
C(17)-C(20)-H(20C)	109.5
H(20A)-C(20)-H(20B)	109.5
H(20A)-C(20)-H(20C)	109.5
H(20B)-C(20)-H(20C)	109.5
C(22)-C(21)-C(1)	120.5(2)
C(22)-C(21)-C(26)	117.4(3)
C(26)-C(21)-C(1)	122.1(3)
C(21)-C(22)-H(22)	119.4
C(21)-C(22)-C(23)	121.1(3)
C(23)-C(22)-H(22)	119.4
C(22)-C(23)-H(23)	119.1
C(24)-C(23)-C(22)	121.7(3)
C(24)-C(23)-H(23)	119.1
C(23)-C(24)-C(25)	117.4(3)
C(23)-C(24)-C(27)	120.8(3)
C(25)-C(24)-C(27)	121.8(3)
C(24)-C(25)-H(25)	119.5
C(24)-C(25)-C(26)	121.1(3)
C(26)-C(25)-H(25)	119.5
C(21)-C(26)-C(25)	121.2(3)
C(21)-C(26)-H(26)	119.4
C(25)-C(26)-H(26)	119.4
C(24)-C(27)-H(27A)	109.5
C(24)-C(27)-H(27B)	109.5
C(24)-C(27)-H(27C)	109.5
H(27A)-C(27)-H(27B)	109.5
H(27A)-C(27)-H(27C)	109.5
H(27B)-C(27)-H(27C)	109.5
C(29)-C(28)-C(4)	120.9(2)
C(29)-C(28)-C(33)	117.5(2)
C(33)-C(28)-C(4)	121.5(2)

C(28)-C(29)-H(29)	119.5
C(30)-C(29)-C(28)	120.9(3)
C(30)-C(29)-H(29)	119.5
C(29)-C(30)-H(30)	119.5
C(31)-C(30)-C(29)	120.9(3)
C(31)-C(30)-H(30)	119.5
C(30)-C(31)-H(31)	120.4
C(30)-C(31)-C(32)	119.3(3)
C(32)-C(31)-H(31)	120.4
C(31)-C(32)-H(32)	120.0
C(33)-C(32)-C(31)	120.0(3)
C(33)-C(32)-H(32)	120.0
C(28)-C(33)-H(33)	119.3
C(32)-C(33)-C(28)	121.3(3)
C(32)-C(33)-H(33)	119.3

Symmetry transformations used to generate equivalent atoms:

Supplementary Table 34. Anisotropic displacement parameters ($Å^2$x 10^3) for **12a**. The anisotropic displacement factor exponent takes the form: $-2pi^2$[h^2 $a^{*2}U^{11}$ + ... + 2 h k a* b* U^{12}]

	U^{11}	U^{22}	U^{33}	U^{23}	U^{13}	U^{12}
Cl(1)	128(1)	74(1)	44(1)	12(1)	-12(1)	-3(1)
S(1)	43(1)	30(1)	47(1)	2(1)	2(1)	-2(1)
O(1)	44(1)	31(1)	63(1)	-6(1)	1(1)	-3(1)
O(2)	60(1)	40(1)	48(1)	5(1)	-11(1)	2(1)
O(3)	58(1)	45(1)	57(1)	4(1)	15(1)	-1(1)
O(4)	52(1)	39(1)	64(1)	1(1)	-9(1)	-8(1)
N(1)	36(1)	30(1)	55(1)	1(1)	1(1)	2(1)
N(2)	50(1)	29(1)	43(1)	1(1)	-3(1)	0(1)
C(1)	44(1)	36(1)	47(1)	0(1)	2(1)	-5(1)
C(2)	38(1)	36(1)	52(1)	-1(1)	0(1)	1(1)
C(3)	38(1)	29(1)	39(1)	2(1)	0(1)	3(1)
C(4)	40(1)	31(1)	38(1)	-1(1)	3(1)	0(1)
C(5)	53(2)	50(1)	40(1)	-5(1)	2(1)	-1(1)
C(6)	49(2)	39(1)	73(2)	-3(1)	4(1)	9(1)
C(7)	39(1)	32(1)	44(1)	0(1)	2(1)	1(1)
C(8)	34(1)	35(1)	39(1)	-2(1)	2(1)	2(1)

C(9)	49(1)	38(1)	43(1)	5(1)	3(1)	2(1)
C(10)	59(2)	52(2)	38(1)	5(1)	2(1)	3(1)
C(11)	68(2)	52(2)	44(1)	-10(1)	-2(1)	0(2)
C(12)	61(2)	38(1)	49(1)	-6(1)	-1(1)	-1(1)
C(13)	39(1)	33(1)	42(1)	-1(1)	2(1)	2(1)
C(14)	44(1)	27(1)	43(1)	3(1)	2(1)	-1(1)
C(15)	50(2)	52(2)	47(1)	-9(1)	-1(1)	-2(1)
C(16)	54(2)	59(2)	59(2)	-14(1)	10(1)	-1(1)
C(17)	44(1)	40(1)	66(2)	6(1)	4(1)	-4(1)
C(18)	57(2)	43(1)	49(1)	7(1)	-11(1)	-2(1)
C(19)	59(2)	37(1)	38(1)	0(1)	-4(1)	2(1)
C(20)	49(2)	71(2)	101(3)	8(2)	10(2)	10(2)
C(21)	44(1)	38(1)	51(1)	-2(1)	2(1)	-7(1)
C(22)	53(2)	48(2)	54(2)	6(1)	-6(1)	-8(1)
C(23)	62(2)	46(1)	73(2)	10(1)	3(2)	-16(2)
C(24)	48(2)	44(1)	79(2)	-18(1)	3(2)	-6(1)
C(25)	82(2)	100(3)	54(2)	-25(2)	3(2)	-34(2)
C(26)	85(2)	100(3)	46(2)	-4(2)	7(2)	-41(2)
C(27)	77(2)	69(2)	117(3)	-35(2)	6(2)	-25(2)
C(28)	38(1)	32(1)	44(1)	2(1)	4(1)	2(1)
C(29)	55(2)	56(2)	52(2)	12(1)	-1(1)	-9(1)
C(30)	66(2)	67(2)	74(2)	17(2)	9(2)	-23(2)
C(31)	50(2)	60(2)	89(2)	2(2)	1(2)	-17(2)
C(32)	47(2)	57(2)	63(2)	-2(1)	-6(1)	-4(1)
C(33)	43(1)	45(1)	48(1)	4(1)	0(1)	-3(1)

Supplementary Table 35. Hydrogen coordinates (x10^4) and isotropic displacement parameters (Å^{2}x10^3) for **12a**.

	x	y	z	U(eq)
H(1)	3471	3885	7129	51
H(2A)	2164	4966	6713	50
H(2B)	2628	4766	5971	50
H(5A)	6401	4594	5171	72
H(5B)	5194	5268	5187	72
H(5C)	4716	4388	5312	72
H(6A)	7160	3116	6482	80

H(6B)	8087	3882	6326	80
H(6C)	7033	3521	5774	80
H(9)	4759	4578	7810	52
H(11)	5567	6587	8825	66
H(12)	5204	7328	7844	60
H(15)	2984	8497	7267	60
H(16)	626	9067	7232	68
H(18)	-2	8236	5375	60
H(19)	2351	7660	5390	54
H(20A)	-2204	8736	6364	111
H(20B)	-1599	9328	5821	111
H(20C)	-1480	9540	6587	111
H(22)	1663	2951	7400	62
H(23)	-186	2059	7138	72
H(25)	241	2578	5200	95
H(26)	1992	3528	5465	92
H(27A)	-847	1116	5814	131
H(27B)	-1983	1793	5638	131
H(27C)	-1885	1468	6376	131
H(29)	7286	5917	5380	66
H(30)	9422	6645	5586	83
H(31)	10634	6555	6600	79
H(32)	9725	5700	7420	67
H(33)	7588	4973	7222	54

Supplementary Table 36. Torsion angles [°] for **12a**.

Cl(1)-C(10)-C(11)-C(12)	-178.7(3)
S(1)-N(2)-C(7)-O(2)	-13.1(4)
S(1)-N(2)-C(7)-C(3)	167.67(17)
S(1)-N(2)-C(13)-C(8)	-173.67(19)
S(1)-N(2)-C(13)-C(12)	7.0(4)
S(1)-C(14)-C(15)-C(16)	176.4(2)
S(1)-C(14)-C(19)-C(18)	-176.4(2)
O(1)-N(1)-C(4)-C(3)	69.5(2)
O(1)-N(1)-C(4)-C(5)	-50.9(3)
O(1)-N(1)-C(4)-C(28)	-173.73(17)
O(1)-C(1)-C(2)-C(3)	-51.4(3)
O(1)-C(1)-C(21)-C(22)	117.4(3)

O(1)-C(1)-C(21)-C(26)	-61.9(4)
O(3)-S(1)-N(2)-C(7)	-38.2(2)
O(3)-S(1)-N(2)-C(13)	143.2(2)
O(3)-S(1)-C(14)-C(15)	-150.0(2)
O(3)-S(1)-C(14)-C(19)	26.8(2)
O(4)-S(1)-N(2)-C(7)	-166.7(2)
O(4)-S(1)-N(2)-C(13)	14.7(3)
O(4)-S(1)-C(14)-C(15)	-16.6(2)
O(4)-S(1)-C(14)-C(19)	160.3(2)
N(1)-O(1)-C(1)-C(2)	62.3(2)
N(1)-O(1)-C(1)-C(21)	-174.46(19)
N(1)-C(4)-C(28)-C(29)	146.4(2)
N(1)-C(4)-C(28)-C(33)	-34.3(3)
N(2)-S(1)-C(14)-C(15)	95.8(2)
N(2)-S(1)-C(14)-C(19)	-87.3(2)
C(1)-O(1)-N(1)-C(4)	-74.9(2)
C(1)-O(1)-N(1)-C(6)	165.9(2)
C(1)-C(2)-C(3)-C(4)	50.1(3)
C(1)-C(2)-C(3)-C(7)	168.6(2)
C(1)-C(2)-C(3)-C(8)	-78.8(3)
C(1)-C(21)-C(22)-C(23)	-178.2(3)
C(1)-C(21)-C(26)-C(25)	176.2(3)
C(2)-C(1)-C(21)-C(22)	-122.0(3)
C(2)-C(1)-C(21)-C(26)	58.7(4)
C(2)-C(3)-C(4)-N(1)	-58.3(2)
C(2)-C(3)-C(4)-C(5)	62.0(2)
C(2)-C(3)-C(4)-C(28)	-173.67(19)
C(2)-C(3)-C(7)-O(2)	-44.7(3)
C(2)-C(3)-C(7)-N(2)	134.5(2)
C(2)-C(3)-C(8)-C(9)	53.1(4)
C(2)-C(3)-C(8)-C(13)	-126.4(2)
C(3)-C(4)-C(28)-C(29)	-101.3(3)
C(3)-C(4)-C(28)-C(33)	78.0(3)
C(3)-C(8)-C(9)-C(10)	179.2(3)
C(3)-C(8)-C(13)-N(2)	1.7(3)
C(3)-C(8)-C(13)-C(12)	-178.9(2)
C(4)-C(3)-C(7)-O(2)	74.6(3)
C(4)-C(3)-C(7)-N(2)	-106.3(2)
C(4)-C(3)-C(8)-C(9)	-73.7(3)
C(4)-C(3)-C(8)-C(13)	106.7(2)
C(4)-C(28)-C(29)-C(30)	179.3(3)
C(4)-C(28)-C(33)-C(32)	-179.5(2)
C(5)-C(4)-C(28)-C(29)	23.4(3)

C(5)-C(4)-C(28)-C(33)	-157.4(2)
C(6)-N(1)-C(4)-C(3)	-179.5(2)
C(6)-N(1)-C(4)-C(5)	60.1(3)
C(6)-N(1)-C(4)-C(28)	-62.8(3)
C(7)-N(2)-C(13)-C(8)	7.6(3)
C(7)-N(2)-C(13)-C(12)	-171.7(3)
C(7)-C(3)-C(4)-N(1)	-177.19(18)
C(7)-C(3)-C(4)-C(5)	-56.9(3)
C(7)-C(3)-C(4)-C(28)	67.4(2)
C(7)-C(3)-C(8)-C(9)	170.3(3)
C(7)-C(3)-C(8)-C(13)	-9.2(3)
C(8)-C(3)-C(4)-N(1)	71.0(2)
C(8)-C(3)-C(4)-C(5)	-168.7(2)
C(8)-C(3)-C(4)-C(28)	-44.4(3)
C(8)-C(3)-C(7)-O(2)	-165.7(3)
C(8)-C(3)-C(7)-N(2)	13.5(2)
C(8)-C(9)-C(10)-Cl(1)	-179.8(2)
C(8)-C(9)-C(10)-C(11)	-0.3(5)
C(9)-C(8)-C(13)-N(2)	-177.9(2)
C(9)-C(8)-C(13)-C(12)	1.5(4)
C(9)-C(10)-C(11)-C(12)	1.8(5)
C(10)-C(11)-C(12)-C(13)	-1.5(5)
C(11)-C(12)-C(13)-N(2)	179.2(3)
C(11)-C(12)-C(13)-C(8)	0.0(4)
C(13)-N(2)-C(7)-O(2)	165.7(2)
C(13)-N(2)-C(7)-C(3)	-13.5(3)
C(13)-C(8)-C(9)-C(10)	-1.3(4)
C(14)-S(1)-N(2)-C(7)	78.1(2)
C(14)-S(1)-N(2)-C(13)	-100.5(2)
C(14)-C(15)-C(16)-C(17)	-0.5(4)
C(15)-C(14)-C(19)-C(18)	0.4(4)
C(15)-C(16)-C(17)-C(18)	1.6(4)
C(15)-C(16)-C(17)-C(20)	-178.3(3)
C(16)-C(17)-C(18)-C(19)	-1.6(4)
C(17)-C(18)-C(19)-C(14)	0.7(4)
C(19)-C(14)-C(15)-C(16)	-0.4(4)
C(20)-C(17)-C(18)-C(19)	178.3(3)
C(21)-C(1)-C(2)-C(3)	-169.3(2)
C(21)-C(22)-C(23)-C(24)	2.2(5)
C(22)-C(21)-C(26)-C(25)	-3.2(6)
C(22)-C(23)-C(24)-C(25)	-3.4(5)
C(22)-C(23)-C(24)-C(27)	178.8(3)
C(23)-C(24)-C(25)-C(26)	1.3(6)

C(24)-C(25)-C(26)-C(21)	2.0(7)
C(26)-C(21)-C(22)-C(23)	1.1(5)
C(27)-C(24)-C(25)-C(26)	179.1(4)
C(28)-C(29)-C(30)-C(31)	-0.3(5)
C(29)-C(28)-C(33)-C(32)	-0.1(4)
C(29)-C(30)-C(31)-C(32)	0.6(6)
C(30)-C(31)-C(32)-C(33)	-0.7(5)
C(31)-C(32)-C(33)-C(28)	0.5(5)
C(33)-C(28)-C(29)-C(30)	0.0(4)

Symmetry transformations used to generate equivalent atoms:

Supplementary Table 37. Hydrogen bonds for **12a** [Å and °].

D-H...A	d(D-H)	d(H...A)	d(D...A)	<(DHA)
C(12)-H(12)...O(4)	0.93	2.31	2.889(3)	120.3

Symmetry transformations used to generate equivalent atoms:

Tables for Single-Crystal X-ray Crystallography of 6

Supplementary Table 38. Crystal data and structure refinement for **6**.

Identification code	**6**	
Empirical formula	$C_{28}H_{32}ClN_2O_4P$	
Formula weight	526.98	
Temperature	296(2) K	
Wavelength	0.71073 A	
Crystal system, space group	Monoclinic, C2/c	
Unit cell dimensions	*a* = 24.051(2) Å	alpha = 90 deg.
	b = 11.1897(8) Å	beta = 93.412(5) deg.
	c = 20.9345(15) Å	gamma = 90 deg.
Volume	5624.0(8) $Å^3$	
Z, Calculated density	8, 1.245 Mg/m^3	
Absorption coefficient	0.227 mm^{-1}	
F(000)	2224	
Crystal size	0.42 x 0.31 x 0.29 mm	
Theta range for data collection	1.70 to 25.00 deg.	

Limiting indices	-28<=h<=27, -13<=k<=13, -24<=l<=24
Reflections collected / unique	32326 / 4958 [R(int) = 0.0834]
Completeness to theta = 25.00	99.8 %
Absorption correction	Semi-empirical from equivalents
Max. and min. transmission	0.9370 and 0.9105
Refinement method	Full-matrix least-squares on F^2
Data / restraints / parameters	4958 / 23 / 325
Goodness-of-fit on F^2	1.065
Final R indices [I>2sigma(I)]	R_1 = 0.1252, wR_2 = 0.3351
R indices (all data)	R_1 = 0.1833, wR_2 = 0.3965
Largest diff. peak and hole	1.730 and -1.068 e. $Å^{-3}$

Supplementary Table 39. Atomic coordinates ($x10^4$) and equivalent isotropic displacement parameters ($Å^2x10^3$) for **6**. U(eq) is defined as one third of the trace of the orthogonalized U^{ij} tensor.

	x	y	z	U(eq)
Cl(1)	904(1)	4412(2)	1955(1)	88(1)
P(1)	1718(1)	6362(3)	5065(1)	95(1)
O(1)	1549(6)	5222(12)	5193(4)	220(5)
O(2)	1079(3)	7083(9)	4981(4)	121(3)
O(3)	2103(2)	6963(6)	5528(3)	81(2)
O(4)	2553(2)	7722(4)	2222(2)	66(1)
N(1)	1895(2)	6528(5)	4350(3)	55(2)
N(2)	3102(2)	6078(5)	2442(2)	46(1)
C(1)	3314(3)	5971(6)	1820(3)	44(2)
C(2)	3574(3)	6922(7)	1550(3)	63(2)
C(3)	3774(4)	6815(7)	947(4)	70(2)
C(4)	3716(3)	5769(7)	596(3)	63(2)
C(5)	3446(4)	4836(7)	881(4)	69(2)
C(6)	3254(3)	4919(7)	1477(4)	60(2)
C(7)	3749(3)	4678(6)	3041(3)	43(2)
C(8)	3834(3)	3454(6)	3115(3)	51(2)
C(9)	4360(3)	3002(7)	3254(3)	60(2)
C(10)	4818(3)	3748(8)	3324(3)	60(2)
C(11)	4737(3)	4963(7)	3254(3)	56(2)
C(12)	4209(3)	5422(6)	3111(3)	52(2)

C(13)	3950(5)	5660(10)	-53(4)	97(3)
C(14)	5399(3)	3260(10)	3464(5)	88(3)
C(15)	2719(3)	6916(6)	2576(3)	50(2)
C(16)	2521(3)	6643(6)	3239(3)	50(2)
C(17)	2953(3)	5784(7)	3517(3)	56(2)
C(18)	3165(3)	5136(6)	2933(3)	44(2)
C(19)	1937(3)	6107(6)	3215(3)	48(2)
C(20)	1697(3)	5618(6)	2662(4)	56(2)
C(21)	1187(3)	5053(7)	2656(4)	60(2)
C(22)	910(3)	4965(6)	3208(4)	61(2)
C(23)	1142(3)	5467(7)	3762(4)	59(2)
C(24)	1656(3)	6037(6)	3772(3)	49(2)
C(25)	795(4)	7189(11)	5469(5)	108(3)
C(26)	218(5)	6740(20)	5379(7)	203(9)
C(27)	2202(12)	4357(13)	5139(10)	351(14)
C(28)	1892(5)	3820(20)	5673(7)	200(7)

Supplementary Table 40. Bond lengths [Å] and angles [deg] for **6**.

Cl(1)-C(21)	1.735(8)
P(1)-O(1)	1.369(10)
P(1)-O(3)	1.463(6)
P(1)-N(1)	1.591(6)
P(1)-O(2)	1.736(9)
O(1)-C(27)	1.85(2)
O(2)-C(25)	1.268(12)
O(4)-C(15)	1.219(8)
N(1)-C(24)	1.421(9)
N(1)-H(1A)	0.8600
N(2)-C(15)	1.355(8)
N(2)-C(1)	1.431(8)
N(2)-C(18)	1.474(8)
C(1)-C(2)	1.373(9)
C(1)-C(6)	1.382(9)
C(2)-C(3)	1.383(10)
C(2)-H(2A)	0.9300
C(3)-C(4)	1.386(11)
C(3)-H(3A)	0.9300

C(4)-C(5)	1.382(11)
C(4)-C(13)	1.507(11)
C(5)-C(6)	1.361(11)
C(5)-H(5A)	0.9300
C(6)-H(6A)	0.9300
C(7)-C(12)	1.385(9)
C(7)-C(8)	1.391(9)
C(7)-C(18)	1.499(9)
C(8)-C(9)	1.379(10)
C(8)-H(8A)	0.9300
C(9)-C(10)	1.381(11)
C(9)-H(9A)	0.9300
C(10)-C(11)	1.381(11)
C(10)-C(14)	1.513(10)
C(11)-C(12)	1.385(10)
C(11)-H(11A)	0.9300
C(12)-H(12A)	0.9300
C(13)-H(13A)	0.9600
C(13)-H(13B)	0.9600
C(13)-H(13C)	0.9600
C(14)-H(14A)	0.9600
C(14)-H(14B)	0.9600
C(14)-H(14C)	0.9600
C(15)-C(16)	1.525(10)
C(16)-C(17)	1.507(9)
C(16)-C(19)	1.526(9)
C(16)-H(16A)	0.9800
C(17)-C(18)	1.535(9)
C(17)-H(17A)	0.9700
C(17)-H(17B)	0.9700
C(18)-H(18A)	0.9800
C(19)-C(20)	1.376(10)
C(19)-C(24)	1.383(10)
C(20)-C(21)	1.380(11)
C(20)-H(20A)	0.9300
C(21)-C(22)	1.370(11)
C(22)-C(23)	1.377(10)
C(22)-H(22A)	0.9300

C(23)-C(24)	1.388(10)
C(23)-H(23A)	0.9300
C(25)-C(26)	1.477(9)
C(25)-H(25A)	0.9700
C(25)-H(25B)	0.9700
C(26)-H(26A)	0.9600
C(26)-H(26B)	0.9600
C(26)-H(26C)	0.9600
C(27)-C(28)	1.506(10)
C(27)-H(27A)	0.9700
C(27)-H(27B)	0.9700
C(28)-H(28A)	0.9600
C(28)-H(28B)	0.9600
C(28)-H(28C)	0.9600
O(1)-P(1)-O(3)	118.8(5)
O(1)-P(1)-N(1)	113.1(6)
O(3)-P(1)-N(1)	112.1(3)
O(1)-P(1)-O(2)	100.4(8)
O(3)-P(1)-O(2)	112.0(4)
N(1)-P(1)-O(2)	97.7(4)
P(1)-O(1)-C(27)	102.2(10)
C(25)-O(2)-P(1)	118.6(8)
C(24)-N(1)-P(1)	129.7(5)
C(24)-N(1)-H(1A)	115.1
P(1)-N(1)-H(1A)	115.1
C(15)-N(2)-C(1)	122.0(5)
C(15)-N(2)-C(18)	113.2(5)
C(1)-N(2)-C(18)	123.1(5)
C(2)-C(1)-C(6)	118.9(6)
C(2)-C(1)-N(2)	120.4(6)
C(6)-C(1)-N(2)	120.8(6)
C(1)-C(2)-C(3)	120.1(7)
C(1)-C(2)-H(2A)	120.0
C(3)-C(2)-H(2A)	120.0
C(4)-C(3)-C(2)	121.8(7)
C(4)-C(3)-H(3A)	119.1
C(2)-C(3)-H(3A)	119.1
C(5)-C(4)-C(3)	116.4(7)

C(5)-C(4)-C(13)	122.6(8)
C(3)-C(4)-C(13)	121.0(8)
C(6)-C(5)-C(4)	122.6(7)
C(6)-C(5)-H(5A)	118.7
C(4)-C(5)-H(5A)	118.7
C(5)-C(6)-C(1)	120.3(7)
C(5)-C(6)-H(6A)	119.9
C(1)-C(6)-H(6A)	119.9
C(12)-C(7)-C(8)	118.0(6)
C(12)-C(7)-C(18)	123.0(6)
C(8)-C(7)-C(18)	118.9(6)
C(9)-C(8)-C(7)	120.7(7)
C(9)-C(8)-H(8A)	119.6
C(7)-C(8)-H(8A)	119.6
C(8)-C(9)-C(10)	121.1(7)
C(8)-C(9)-H(9A)	119.5
C(10)-C(9)-H(9A)	119.5
C(11)-C(10)-C(9)	118.6(6)
C(11)-C(10)-C(14)	119.9(8)
C(9)-C(10)-C(14)	121.6(8)
C(10)-C(11)-C(12)	120.6(7)
C(10)-C(11)-H(11A)	119.7
C(12)-C(11)-H(11A)	119.7
C(7)-C(12)-C(11)	121.1(7)
C(7)-C(12)-H(12A)	119.5
C(11)-C(12)-H(12A)	119.5
C(4)-C(13)-H(13A)	109.5
C(4)-C(13)-H(13B)	109.5
H(13A)-C(13)-H(13B)	109.5
C(4)-C(13)-H(13C)	109.5
H(13A)-C(13)-H(13C)	109.5
H(13B)-C(13)-H(13C)	109.5
C(10)-C(14)-H(14A)	109.5
C(10)-C(14)-H(14B)	109.5
H(14A)-C(14)-H(14B)	109.5
C(10)-C(14)-H(14C)	109.5
H(14A)-C(14)-H(14C)	109.5
H(14B)-C(14)-H(14C)	109.5

O(4)-C(15)-N(2)	126.0(7)
O(4)-C(15)-C(16)	126.3(6)
N(2)-C(15)-C(16)	107.7(5)
C(17)-C(16)-C(19)	112.0(6)
C(17)-C(16)-C(15)	103.5(5)
C(19)-C(16)-C(15)	112.8(6)
C(17)-C(16)-H(16A)	109.5
C(19)-C(16)-H(16A)	109.5
C(15)-C(16)-H(16A)	109.5
C(16)-C(17)-C(18)	104.4(5)
C(16)-C(17)-H(17A)	110.9
C(18)-C(17)-H(17A)	110.9
C(16)-C(17)-H(17B)	110.9
C(18)-C(17)-H(17B)	110.9
H(17A)-C(17)-H(17B)	108.9
N(2)-C(18)-C(7)	114.0(5)
N(2)-C(18)-C(17)	101.0(5)
C(7)-C(18)-C(17)	113.2(5)
N(2)-C(18)-H(18A)	109.4
C(7)-C(18)-H(18A)	109.4
C(17)-C(18)-H(18A)	109.4
C(20)-C(19)-C(24)	119.0(7)
C(20)-C(19)-C(16)	121.5(6)
C(24)-C(19)-C(16)	119.3(6)
C(19)-C(20)-C(21)	121.1(7)
C(19)-C(20)-H(20A)	119.4
C(21)-C(20)-H(20A)	119.4
C(22)-C(21)-C(20)	120.1(7)
C(22)-C(21)-Cl(1)	119.9(6)
C(20)-C(21)-Cl(1)	120.0(6)
C(21)-C(22)-C(23)	119.2(7)
C(21)-C(22)-H(22A)	120.4
C(23)-C(22)-H(22A)	120.4
C(22)-C(23)-C(24)	121.1(7)
C(22)-C(23)-H(23A)	119.5
C(24)-C(23)-H(23A)	119.5
C(19)-C(24)-C(23)	119.5(6)
C(19)-C(24)-N(1)	120.2(6)

C(23)-C(24)-N(1)	120.4(6)
O(2)-C(25)-C(26)	114.4(10)
O(2)-C(25)-H(25A)	108.7
C(26)-C(25)-H(25A)	108.7
O(2)-C(25)-H(25B)	108.7
C(26)-C(25)-H(25B)	108.7
H(25A)-C(25)-H(25B)	107.6
C(25)-C(26)-H(26A)	109.5
C(25)-C(26)-H(26B)	109.5
H(26A)-C(26)-H(26B)	109.5
C(25)-C(26)-H(26C)	109.5
H(26A)-C(26)-H(26C)	109.5
H(26B)-C(26)-H(26C)	109.5
C(28)-C(27)-O(1)	72.9(13)
C(28)-C(27)-H(27A)	116.3
O(1)-C(27)-H(27A)	116.3
C(28)-C(27)-H(27B)	116.3
O(1)-C(27)-H(27B)	116.3
H(27A)-C(27)-H(27B)	113.2
C(27)-C(28)-H(28A)	109.5
C(27)-C(28)-H(28B)	109.5
H(28A)-C(28)-H(28B)	109.5
C(27)-C(28)-H(28C)	109.5
H(28A)-C(28)-H(28C)	109.5
H(28B)-C(28)-H(28C)	109.5

Symmetry transformations used to generate equivalent atoms:

Supplementary Table 41. Anisotropic displacement parameters ($Å^2x10^3$) for **6**. The anisotropic displacement factor exponent takes the form: $-2 pi^2 [h^2 a^{*2} U^{11} + ... + 2 h k a^* b^* U^{12}]$

	U11	U22	U33	U23	U13	U12
Cl(1)	104(2)	86(2)	72(1)	-11(1)	-18(1)	-20(1)
P(1)	76(2)	148(2)	61(1)	-22(2)	17(1)	-56(2)
O(1)	313(12)	255(7)	82(5)	59(7)	-69(7)	-222(9)
O(2)	75(4)	185(8)	103(5)	-12(5)	6(3)	-7(5)
O(3)	58(3)	119(5)	65(3)	-17(3)	8(3)	-25(3)
O(4)	72(3)	54(3)	71(3)	17(3)	7(3)	17(3)

N(1)	41(3)	59(4)	65(4)	-10(3)	2(3)	-1(3)
N(2)	46(3)	45(3)	47(3)	2(2)	1(2)	5(2)
C(1)	44(4)	42(3)	46(4)	-2(3)	-7(3)	-1(3)
C(2)	86(6)	52(4)	50(4)	-2(3)	9(4)	-14(4)
C(3)	93(6)	59(5)	57(5)	9(4)	4(4)	-13(4)
C(4)	74(5)	66(5)	48(4)	-6(4)	-2(4)	10(4)
C(5)	76(6)	61(5)	70(5)	-26(4)	-1(4)	-3(4)
C(6)	63(5)	50(4)	67(5)	-7(4)	6(4)	-6(3)
C(7)	39(3)	46(4)	43(3)	-1(3)	1(3)	0(3)
C(8)	51(4)	42(4)	59(4)	4(3)	-6(3)	0(3)
C(9)	58(5)	56(4)	65(5)	9(4)	-4(4)	15(4)
C(10)	48(4)	82(5)	48(4)	8(4)	-3(3)	15(4)
C(11)	39(4)	73(5)	55(4)	0(4)	-1(3)	-8(3)
C(12)	51(4)	47(4)	58(4)	1(3)	2(3)	-3(3)
C(13)	125(9)	112(8)	55(5)	-12(5)	19(5)	10(7)
C(14)	53(5)	126(8)	85(6)	10(6)	-4(4)	24(5)
C(15)	49(4)	44(4)	57(4)	1(3)	0(3)	6(3)
C(16)	47(4)	49(4)	54(4)	-3(3)	2(3)	7(3)
C(17)	51(4)	64(4)	53(4)	8(3)	-2(3)	9(3)
C(18)	41(4)	43(3)	49(4)	4(3)	-1(3)	2(3)
C(19)	45(4)	38(3)	62(4)	2(3)	-3(3)	14(3)
C(20)	60(5)	50(4)	58(4)	3(3)	-4(3)	8(3)
C(21)	65(5)	51(4)	64(5)	2(3)	-9(4)	6(4)
C(22)	50(4)	52(4)	80(5)	-4(4)	-8(4)	2(3)
C(23)	43(4)	67(5)	67(5)	-10(4)	2(3)	7(3)
C(24)	40(4)	49(4)	60(4)	-3(3)	4(3)	11(3)
C(25)	106(6)	128(9)	89(6)	0(7)	4(5)	25(7)
C(26)	88(7)	410(30)	116(11)	-25(14)	34(7)	-6(12)
C(27)	810(40)	122(13)	129(15)	7(11)	70(20)	106(18)
C(28)	92(9)	350(18)	153(12)	-114(10)	-42(8)	81(12)

Supplementary Table 42. Hydrogen coordinates (x10^4) and isotropic displacement parameters (Å^{2}x10^3) for **6**.

	x	y	z	U(eq)
H(1A)	2178	6984	4308	66
H(2A)	3614	7640	1772	75
H(3A)	3954	7464	773	84
H(5A)	3394	4124	656	83
H(6A)	3082	4265	1655	72
H(8A)	3532	2936	3069	62

H(9A)	4409	2182	3303	72
H(11A)	5040	5479	3302	67
H(12A)	4163	6243	3062	62
H(13A)	3870	4879	-225	145
H(13B)	4346	5779	-14	145
H(13C)	3783	6254	-335	145
H(14A)	5660	3910	3498	132
H(14B)	5494	2737	3124	132
H(14C)	5412	2825	3860	132
H(16A)	2528	7377	3495	60
H(17A)	2789	5224	3805	68
H(17B)	3253	6209	3748	68
H(18A)	2916	4469	2815	53
H(20A)	1882	5671	2285	68
H(22A)	569	4572	3208	73
H(23A)	953	5422	4136	71
H(25A)	783	8027	5586	129
H(25B)	982	6761	5823	129
H(26A)	31	6843	5768	304
H(26B)	224	5906	5270	304
H(26C)	23	7176	5040	304
H(27A)	2229	3868	4760	422
H(27B)	2545	4773	5265	422
H(28A)	2144	3351	5944	301
H(28B)	1599	3314	5496	301
H(28C)	1736	4444	5920	301

Supplementary Table 43. Torsion angles [deg] for **6**.

O(3)-P(1)-O(1)-C(27)	-67.6(10)
N(1)-P(1)-O(1)-C(27)	66.9(9)
O(2)-P(1)-O(1)-C(27)	170.0(8)
O(1)-P(1)-O(2)-C(25)	67.0(10)
O(3)-P(1)-O(2)-C(25)	-60.1(10)
N(1)-P(1)-O(2)-C(25)	-177.7(9)
O(1)-P(1)-N(1)-C(24)	38.4(10)
O(3)-P(1)-N(1)-C(24)	176.0(6)
O(2)-P(1)-N(1)-C(24)	-66.4(7)
C(15)-N(2)-C(1)-C(2)	57.9(9)
C(18)-N(2)-C(1)-C(2)	-137.8(7)
C(15)-N(2)-C(1)-C(6)	-121.0(7)
C(18)-N(2)-C(1)-C(6)	43.3(9)

C(6)-C(1)-C(2)-C(3)	-0.6(11)
N(2)-C(1)-C(2)-C(3)	-179.5(7)
C(1)-C(2)-C(3)-C(4)	0.9(13)
C(2)-C(3)-C(4)-C(5)	-0.1(12)
C(2)-C(3)-C(4)-C(13)	-178.5(8)
C(3)-C(4)-C(5)-C(6)	-1.0(13)
C(13)-C(4)-C(5)-C(6)	177.3(9)
C(4)-C(5)-C(6)-C(1)	1.3(13)
C(2)-C(1)-C(6)-C(5)	-0.5(11)
N(2)-C(1)-C(6)-C(5)	178.4(7)
C(12)-C(7)-C(8)-C(9)	0.1(10)
C(18)-C(7)-C(8)-C(9)	-176.0(6)
C(7)-C(8)-C(9)-C(10)	-0.1(11)
C(8)-C(9)-C(10)-C(11)	0.4(11)
C(8)-C(9)-C(10)-C(14)	-178.8(7)
C(9)-C(10)-C(11)-C(12)	-0.5(11)
C(14)-C(10)-C(11)-C(12)	178.6(7)
C(8)-C(7)-C(12)-C(11)	-0.3(10)
C(18)-C(7)-C(12)-C(11)	175.6(6)
C(10)-C(11)-C(12)-C(7)	0.5(10)
C(1)-N(2)-C(15)-O(4)	-7.2(10)
C(18)-N(2)-C(15)-O(4)	-172.9(6)
C(1)-N(2)-C(15)-C(16)	170.9(5)
C(18)-N(2)-C(15)-C(16)	5.2(7)
O(4)-C(15)-C(16)-C(17)	-166.5(7)
N(2)-C(15)-C(16)-C(17)	15.4(7)
O(4)-C(15)-C(16)-C(19)	72.3(9)
N(2)-C(15)-C(16)-C(19)	-105.8(6)
C(19)-C(16)-C(17)-C(18)	92.9(7)
C(15)-C(16)-C(17)-C(18)	-28.9(7)
C(15)-N(2)-C(18)-C(7)	-144.8(6)
C(1)-N(2)-C(18)-C(7)	49.6(8)
C(15)-N(2)-C(18)-C(17)	-23.0(7)
C(1)-N(2)-C(18)-C(17)	171.4(5)
C(12)-C(7)-C(18)-N(2)	46.3(8)
C(8)-C(7)-C(18)-N(2)	-137.8(6)
C(12)-C(7)-C(18)-C(17)	-68.5(8)
C(8)-C(7)-C(18)-C(17)	107.4(7)
C(16)-C(17)-C(18)-N(2)	31.2(7)
C(16)-C(17)-C(18)-C(7)	153.5(6)
C(17)-C(16)-C(19)-C(20)	-98.8(7)
C(15)-C(16)-C(19)-C(20)	17.4(8)
C(17)-C(16)-C(19)-C(24)	76.8(8)

C(15)-C(16)-C(19)-C(24)	-166.9(6)
C(24)-C(19)-C(20)-C(21)	-0.8(10)
C(16)-C(19)-C(20)-C(21)	174.9(6)
C(19)-C(20)-C(21)-C(22)	-0.2(11)
C(19)-C(20)-C(21)-Cl(1)	-178.3(5)
C(20)-C(21)-C(22)-C(23)	1.2(11)
Cl(1)-C(21)-C(22)-C(23)	179.3(6)
C(21)-C(22)-C(23)-C(24)	-1.2(11)
C(20)-C(19)-C(24)-C(23)	0.7(10)
C(16)-C(19)-C(24)-C(23)	-175.0(6)
C(20)-C(19)-C(24)-N(1)	179.7(6)
C(16)-C(19)-C(24)-N(1)	3.9(9)
C(22)-C(23)-C(24)-C(19)	0.2(10)
C(22)-C(23)-C(24)-N(1)	-178.7(6)
P(1)-N(1)-C(24)-C(19)	-167.4(5)
P(1)-N(1)-C(24)-C(23)	11.5(10)
P(1)-O(2)-C(25)-C(26)	-126.5(13)
P(1)-O(1)-C(27)-C(28)	143.0(10)

Symmetry transformations used to generate equivalent atoms:

Supplementary Table 44. Hydrogen bonds for **6** [Å and deg.].

D-H...A	d(D-H)	d(H...A)	d(D...A)	<(DHA)

Tables for Single-Crystal X-ray Crystallography of 8

Supplementary Table 45. Crystal data and structure refinement for **8**.

Identification code	**8**	
Empirical formula	$C_{24}H_{19}Cl_2NO_2$	
Formula weight	424.30	
Temperature	296(2) K	
Wavelength	0.71073 Å	
Crystal system, space group	Monoclinic, P2(1)/c	
Unit cell dimensions	*a* = 10.0939(6) Å	*alpha* = 90 deg.
	b = 24.1607(14) Å	*beta* = 112.346(2) deg.
	c = 9.0663(5) Å	*gamma* = 90 deg.
Volume	2045.0(2) Å^3	

Z, Calculated density	4, 1.378 Mg/m^3
Absorption coefficient	0.338 mm^{-1}
F(000)	880
Crystal size	0.47 x 0.41 x 0.19 mm
Theta range for data collection	2.18 to 25.01 deg.
Limiting indices	-12<=h<=12, -28<=k<=28, -10<=l<=10
Reflections collected / unique	23441 / 3604 [R(int) = 0.0447]
Completeness to theta = 25.01	100.0 %
Absorption correction	Semi-empirical from equivalents
Max. and min. transmission	0.9385 and 0.8573
Refinement method	Full-matrix least-squares on F^2
Data / restraints / parameters	3604 / 0 / 262
Goodness-of-fit on F^2	1.040
Final R indices [I>2sigma(I)]	R_1 = 0.0350, wR_2 = 0.0790
R indices (all data)	R_1 = 0.0466, wR_2 = 0.0857
Largest diff. peak and hole	0.256 and -0.208 e.$Å^{-3}$

Supplementary Table 46. Atomic coordinates ($x10^4$) and equivalent isotropic displacement parameters ($Å^2x10^3$) for **8**. U(eq) is defined as one third of the trace of the orthogonalized U^{ij} tensor.

	x	y	z	U(eq)
Cl(1)	5785(1)	1972(1)	4748(1)	44(1)
Cl(2)	6926(1)	752(1)	12181(1)	44(1)
O(1)	-651(1)	656(1)	5585(2)	31(1)
O(2)	1191(1)	2165(1)	7284(1)	25(1)
N(1)	1333(2)	472(1)	5007(2)	30(1)
C(1)	541(2)	3255(1)	6098(2)	29(1)
C(2)	163(2)	3800(1)	5628(2)	35(1)
C(3)	-1156(2)	3931(1)	4471(2)	35(1)
C(4)	-2088(2)	3502(1)	3773(3)	38(1)
C(5)	-1729(2)	2961(1)	4249(2)	34(1)
C(6)	-418(2)	2830(1)	5431(2)	25(1)
C(7)	-134(2)	2237(1)	5950(2)	24(1)

C(8)	-19(2)	1840(1)	4683(2)	28(1)
C(9)	1049(2)	1391(1)	5658(2)	24(1)
C(10)	440(2)	804(1)	5403(2)	26(1)
C(11)	2495(2)	772(1)	4925(2)	27(1)
C(12)	3609(2)	577(1)	4546(2)	35(1)
C(13)	4629(2)	958(1)	4516(2)	35(1)
C(14)	4499(2)	1508(1)	4850(2)	30(1)
C(15)	3379(2)	1702(1)	5243(2)	27(1)
C(16)	2372(2)	1324(1)	5285(2)	24(1)
C(17)	1338(2)	1580(1)	7408(2)	23(1)
C(18)	2733(2)	1395(1)	8636(2)	25(1)
C(19)	2857(2)	851(1)	9171(2)	30(1)
C(20)	4141(2)	651(1)	10254(2)	34(1)
C(21)	5304(2)	1001(1)	10811(2)	32(1)
C(22)	5202(2)	1543(1)	10320(2)	34(1)
C(23)	3916(2)	1737(1)	9228(2)	30(1)
C(24)	-1579(3)	4520(1)	3956(3)	54(1)

Supplementary Table 47. Bond lengths [Å] and angles [deg] for **8**.

Cl(1)-C(14)	1.7433(19)
Cl(2)-C(21)	1.743(2)
O(1)-C(10)	1.227(2)
O(2)-C(17)	1.421(2)
O(2)-C(7)	1.432(2)
N(1)-C(10)	1.352(2)
N(1)-C(11)	1.403(2)
N(1)-H(1B)	0.8600
C(1)-C(6)	1.382(3)
C(1)-C(2)	1.392(3)
C(1)-H(1A)	0.9300
C(2)-C(3)	1.382(3)
C(2)-H(2A)	0.9300
C(3)-C(4)	1.381(3)
C(3)-C(24)	1.509(3)
C(4)-C(5)	1.381(3)
C(4)-H(4A)	0.9300
C(5)-C(6)	1.385(3)

C(5)-H(5A)	0.9300
C(6)-C(7)	1.503(2)
C(7)-C(8)	1.534(2)
C(7)-H(7A)	0.9800
C(8)-C(9)	1.549(2)
C(8)-H(8A)	0.9700
C(8)-H(8B)	0.9700
C(9)-C(16)	1.505(2)
C(9)-C(10)	1.528(2)
C(9)-C(17)	1.568(2)
C(11)-C(12)	1.377(3)
C(11)-C(16)	1.390(2)
C(12)-C(13)	1.388(3)
C(12)-H(12A)	0.9300
C(13)-C(14)	1.381(3)
C(13)-H(13A)	0.9300
C(14)-C(15)	1.389(3)
C(15)-C(16)	1.378(2)
C(15)-H(15A)	0.9300
C(17)-C(18)	1.494(3)
C(17)-H(17A)	0.9800
C(18)-C(23)	1.382(3)
C(18)-C(19)	1.389(3)
C(19)-C(20)	1.382(3)
C(19)-H(19A)	0.9300
C(20)-C(21)	1.376(3)
C(20)-H(20A)	0.9300
C(21)-C(22)	1.376(3)
C(22)-C(23)	1.381(3)
C(22)-H(22A)	0.9300
C(23)-H(23A)	0.9300
C(24)-H(24A)	0.9600
C(24)-H(24B)	0.9600
C(24)-H(24C)	0.9600
C(17)-O(2)-C(7)	102.88(13)
C(10)-N(1)-C(11)	111.31(15)
C(10)-N(1)-H(1B)	124.3
C(11)-N(1)-H(1B)	124.3

C(6)-C(1)-C(2)	120.21(19)
C(6)-C(1)-H(1A)	119.9
C(2)-C(1)-H(1A)	119.9
C(3)-C(2)-C(1)	121.47(19)
C(3)-C(2)-H(2A)	119.3
C(1)-C(2)-H(2A)	119.3
C(4)-C(3)-C(2)	117.85(18)
C(4)-C(3)-C(24)	120.2(2)
C(2)-C(3)-C(24)	121.9(2)
C(5)-C(4)-C(3)	121.0(2)
C(5)-C(4)-H(4A)	119.5
C(3)-C(4)-H(4A)	119.5
C(4)-C(5)-C(6)	121.14(19)
C(4)-C(5)-H(5A)	119.4
C(6)-C(5)-H(5A)	119.4
C(1)-C(6)-C(5)	118.28(17)
C(1)-C(6)-C(7)	123.55(17)
C(5)-C(6)-C(7)	118.15(17)
O(2)-C(7)-C(6)	112.69(15)
O(2)-C(7)-C(8)	104.16(14)
C(6)-C(7)-C(8)	114.93(15)
O(2)-C(7)-H(7A)	108.3
C(6)-C(7)-H(7A)	108.3
C(8)-C(7)-H(7A)	108.3
C(7)-C(8)-C(9)	104.17(14)
C(7)-C(8)-H(8A)	110.9
C(9)-C(8)-H(8A)	110.9
C(7)-C(8)-H(8B)	110.9
C(9)-C(8)-H(8B)	110.9
H(8A)-C(8)-H(8B)	108.9
C(16)-C(9)-C(10)	101.92(14)
C(16)-C(9)-C(8)	115.56(15)
C(10)-C(9)-C(8)	114.31(15)
C(16)-C(9)-C(17)	114.79(15)
C(10)-C(9)-C(17)	109.42(14)
C(8)-C(9)-C(17)	101.22(14)
O(1)-C(10)-N(1)	125.83(17)
O(1)-C(10)-C(9)	125.50(16)

N(1)-C(10)-C(9)	108.62(15)
C(12)-C(11)-C(16)	122.69(17)
C(12)-C(11)-N(1)	127.97(17)
C(16)-C(11)-N(1)	109.34(16)
C(11)-C(12)-C(13)	117.45(18)
C(11)-C(12)-H(12A)	121.3
C(13)-C(12)-H(12A)	121.3
C(14)-C(13)-C(12)	120.05(18)
C(14)-C(13)-H(13A)	120.0
C(12)-C(13)-H(13A)	120.0
C(13)-C(14)-C(15)	122.27(17)
C(13)-C(14)-Cl(1)	118.23(15)
C(15)-C(14)-Cl(1)	119.50(15)
C(16)-C(15)-C(14)	117.76(17)
C(16)-C(15)-H(15A)	121.1
C(14)-C(15)-H(15A)	121.1
C(15)-C(16)-C(11)	119.76(17)
C(15)-C(16)-C(9)	131.46(16)
C(11)-C(16)-C(9)	108.77(15)
O(2)-C(17)-C(18)	113.40(14)
O(2)-C(17)-C(9)	103.64(13)
C(18)-C(17)-C(9)	115.23(14)
O(2)-C(17)-H(17A)	108.1
C(18)-C(17)-H(17A)	108.1
C(9)-C(17)-H(17A)	108.1
C(23)-C(18)-C(19)	118.70(17)
C(23)-C(18)-C(17)	122.74(16)
C(19)-C(18)-C(17)	118.53(16)
C(20)-C(19)-C(18)	120.94(18)
C(20)-C(19)-H(19A)	119.5
C(18)-C(19)-H(19A)	119.5
C(21)-C(20)-C(19)	119.02(18)
C(21)-C(20)-H(20A)	120.5
C(19)-C(20)-H(20A)	120.5
C(20)-C(21)-C(22)	121.15(18)
C(20)-C(21)-Cl(2)	119.28(15)
C(22)-C(21)-Cl(2)	119.56(16)
C(21)-C(22)-C(23)	119.26(18)

C(21)-C(22)-H(22A)	120.4
C(23)-C(22)-H(22A)	120.4
C(22)-C(23)-C(18)	120.90(18)
C(22)-C(23)-H(23A)	119.5
C(18)-C(23)-H(23A)	119.5
C(3)-C(24)-H(24A)	109.5
C(3)-C(24)-H(24B)	109.5
H(24A)-C(24)-H(24B)	109.5
C(3)-C(24)-H(24C)	109.5
H(24A)-C(24)-H(24C)	109.5
H(24B)-C(24)-H(24C)	109.5

Symmetry transformations used to generate equivalent atoms:

Supplementary Table 48. Anisotropic displacement parameters (A^2x10^3) for **8**. The anisotropic displacement factor exponent takes the form: $-2\pi^2[h^2 a^{*2}U^{11} + ... + 2 h k a^* b^* U^{12}]$

	U11	U22	U33	U23	U13	U12
Cl(1)	39(1)	35(1)	65(1)	7(1)	29(1)	-3(1)
Cl(2)	31(1)	57(1)	38(1)	9(1)	6(1)	9(1)
O(1)	27(1)	25(1)	41(1)	-3(1)	12(1)	-4(1)
O(2)	30(1)	21(1)	24(1)	0(1)	8(1)	2(1)
N(1)	30(1)	18(1)	40(1)	-5(1)	13(1)	-3(1)
C(1)	35(1)	28(1)	25(1)	1(1)	12(1)	1(1)
C(2)	50(1)	26(1)	34(1)	-5(1)	19(1)	-6(1)
C(3)	49(1)	26(1)	36(1)	4(1)	24(1)	8(1)
C(4)	35(1)	35(1)	41(1)	8(1)	12(1)	10(1)
C(5)	30(1)	28(1)	42(1)	1(1)	12(1)	0(1)
C(6)	29(1)	25(1)	25(1)	0(1)	16(1)	3(1)
C(7)	24(1)	25(1)	24(1)	1(1)	10(1)	0(1)
C(8)	31(1)	23(1)	25(1)	1(1)	6(1)	3(1)
C(9)	26(1)	20(1)	24(1)	0(1)	8(1)	-1(1)
C(10)	26(1)	22(1)	24(1)	0(1)	4(1)	0(1)
C(11)	29(1)	24(1)	27(1)	-1(1)	8(1)	-2(1)
C(12)	40(1)	24(1)	43(1)	-5(1)	18(1)	2(1)
C(13)	35(1)	36(1)	40(1)	-2(1)	19(1)	4(1)
C(14)	30(1)	29(1)	32(1)	4(1)	12(1)	-2(1)

C(15)	34(1)	21(1)	27(1)	2(1)	10(1)	0(1)
C(16)	28(1)	23(1)	19(1)	0(1)	7(1)	0(1)
C(17)	27(1)	20(1)	24(1)	1(1)	11(1)	-1(1)
C(18)	29(1)	25(1)	22(1)	0(1)	12(1)	0(1)
C(19)	30(1)	28(1)	31(1)	2(1)	9(1)	-4(1)
C(20)	36(1)	29(1)	34(1)	8(1)	11(1)	4(1)
C(21)	28(1)	40(1)	25(1)	2(1)	8(1)	5(1)
C(22)	30(1)	34(1)	34(1)	-2(1)	9(1)	-6(1)
C(23)	34(1)	26(1)	30(1)	0(1)	10(1)	-2(1)
C(24)	78(2)	30(1)	61(2)	10(1)	33(1)	14(1)

Supplementary Table 49. Hydrogen coordinates (x10^4) and isotropic displacement parameters (Å^{2}x10^3) for **8**.

	x	y	z	U(eq)
H(1B)	1205	123	4827	36
H(1A)	1442	3176	6864	35
H(2A)	812	4082	6102	43
H(4A)	-2971	3578	2970	45
H(5A)	-2378	2679	3769	41
H(7A)	-913	2108	6259	29
H(8A)	342	2031	3970	33
H(8B)	-943	1679	4060	33
H(12A)	3675	205	4319	42
H(13A)	5400	842	4271	42
H(15A)	3312	2073	5470	33
H(17A)	566	1434	7703	28
H(19A)	2064	619	8795	37
H(20A)	4218	286	10601	40
H(22A)	5991	1777	10720	41
H(23A)	3845	2103	8886	36
H(24A)	-2527	4526	3147	82
H(24B)	-918	4676	3538	82
H(24C)	-1560	4734	4856	82

Supplementary Table 50. Torsion angles [deg] for **8**.

C(6)-C(1)-C(2)-C(3)	-1.2(3)
C(1)-C(2)-C(3)-C(4)	-0.9(3)
C(1)-C(2)-C(3)-C(24)	179.79(19)
C(2)-C(3)-C(4)-C(5)	1.8(3)
C(24)-C(3)-C(4)-C(5)	-178.9(2)
C(3)-C(4)-C(5)-C(6)	-0.6(3)
C(2)-C(1)-C(6)-C(5)	2.5(3)
C(2)-C(1)-C(6)-C(7)	-175.71(17)
C(4)-C(5)-C(6)-C(1)	-1.6(3)
C(4)-C(5)-C(6)-C(7)	176.70(18)
C(17)-O(2)-C(7)-C(6)	-171.64(14)
C(17)-O(2)-C(7)-C(8)	-46.42(16)
C(1)-C(6)-C(7)-O(2)	4.2(2)
C(5)-C(6)-C(7)-O(2)	-174.00(15)
C(1)-C(6)-C(7)-C(8)	-114.9(2)
C(5)-C(6)-C(7)-C(8)	66.9(2)
O(2)-C(7)-C(8)-C(9)	25.19(17)
C(6)-C(7)-C(8)-C(9)	148.97(15)
C(7)-C(8)-C(9)-C(16)	-121.41(16)
C(7)-C(8)-C(9)-C(10)	120.73(16)
C(7)-C(8)-C(9)-C(17)	3.25(17)
C(11)-N(1)-C(10)-O(1)	-178.86(18)
C(11)-N(1)-C(10)-C(9)	-1.2(2)
C(16)-C(9)-C(10)-O(1)	179.39(17)
C(8)-C(9)-C(10)-O(1)	-55.2(2)
C(17)-C(9)-C(10)-O(1)	57.5(2)
C(16)-C(9)-C(10)-N(1)	1.74(19)
C(8)-C(9)-C(10)-N(1)	127.14(16)
C(17)-C(9)-C(10)-N(1)	-120.19(16)
C(10)-N(1)-C(11)-C(12)	-179.41(19)
C(10)-N(1)-C(11)-C(16)	0.1(2)
C(16)-C(11)-C(12)-C(13)	-0.6(3)
N(1)-C(11)-C(12)-C(13)	178.87(19)
C(11)-C(12)-C(13)-C(14)	-0.4(3)
C(12)-C(13)-C(14)-C(15)	0.9(3)
C(12)-C(13)-C(14)-Cl(1)	-178.44(16)
C(13)-C(14)-C(15)-C(16)	-0.4(3)
Cl(1)-C(14)-C(15)-C(16)	178.94(14)
C(14)-C(15)-C(16)-C(11)	-0.6(3)
C(14)-C(15)-C(16)-C(9)	-179.98(18)
C(12)-C(11)-C(16)-C(15)	1.1(3)

N(1)-C(11)-C(16)-C(15)	-178.45(16)
C(12)-C(11)-C(16)-C(9)	-179.38(18)
N(1)-C(11)-C(16)-C(9)	1.1(2)
C(10)-C(9)-C(16)-C(15)	177.77(19)
C(8)-C(9)-C(16)-C(15)	53.2(3)
C(17)-C(9)-C(16)-C(15)	-64.1(3)
C(10)-C(9)-C(16)-C(11)	-1.67(19)
C(8)-C(9)-C(16)-C(11)	-126.23(17)
C(17)-C(9)-C(16)-C(11)	116.48(17)
C(7)-O(2)-C(17)-C(18)	174.30(14)
C(7)-O(2)-C(17)-C(9)	48.64(15)
C(16)-C(9)-C(17)-O(2)	94.20(16)
C(10)-C(9)-C(17)-O(2)	-151.97(14)
C(8)-C(9)-C(17)-O(2)	-30.98(16)
C(16)-C(9)-C(17)-C(18)	-30.3(2)
C(10)-C(9)-C(17)-C(18)	83.55(18)
C(8)-C(9)-C(17)-C(18)	-155.47(15)
O(2)-C(17)-C(18)-C(23)	-18.8(2)
C(9)-C(17)-C(18)-C(23)	100.4(2)
O(2)-C(17)-C(18)-C(19)	163.01(15)
C(9)-C(17)-C(18)-C(19)	-77.8(2)
C(23)-C(18)-C(19)-C(20)	-1.1(3)
C(17)-C(18)-C(19)-C(20)	177.18(17)
C(18)-C(19)-C(20)-C(21)	0.5(3)
C(19)-C(20)-C(21)-C(22)	0.6(3)
C(19)-C(20)-C(21)-Cl(2)	179.86(15)
C(20)-C(21)-C(22)-C(23)	-1.2(3)
Cl(2)-C(21)-C(22)-C(23)	179.62(15)
C(21)-C(22)-C(23)-C(18)	0.6(3)
C(19)-C(18)-C(23)-C(22)	0.5(3)
C(17)-C(18)-C(23)-C(22)	-177.63(17)

Symmetry transformations used to generate equivalent atoms:

Supplementary Table 51. Hydrogen bonds for **8** [A and deg.].

D-H...A	d(D-H)	d(H...A)	d(D...A)	<(DHA)

Supplementary Figures

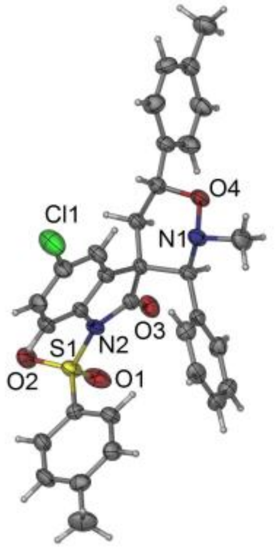

Supplementary Figure 8. X-ray diffraction structure of compound major-**4e** (CCDC-1502102)

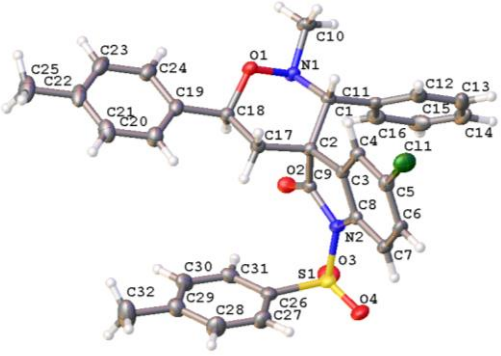

Supplementary Figure 9. X-ray diffraction structure of compound minor-**4e** (CCDC-1551103)

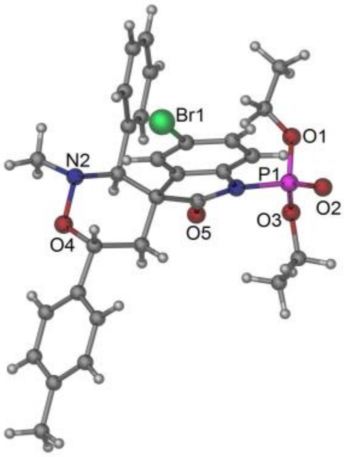

Supplementary Figure 10. X-ray diffraction structure of compound major-**5o** (CCDC-1502103)

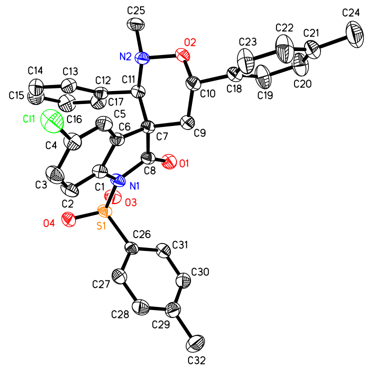

Supplementary Figure 11. X-ray diffraction structure of compound enriched **4e** (CCDC-1502104)

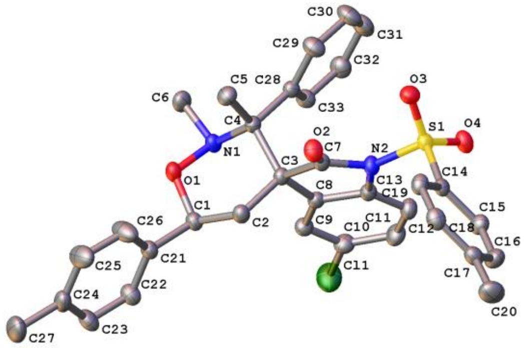

Supplementary Figure 12. X-ray diffraction structure of compound **12a** (CCDC-1502105)

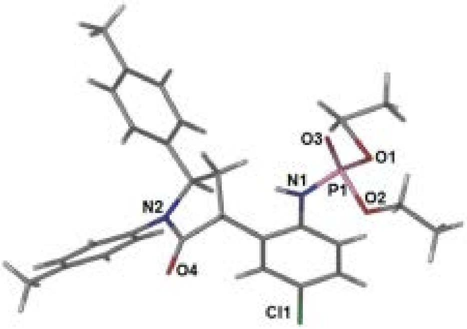

Supplementary Figure 13. X-ray diffraction structure of compound **6** (CCDC-1523864)

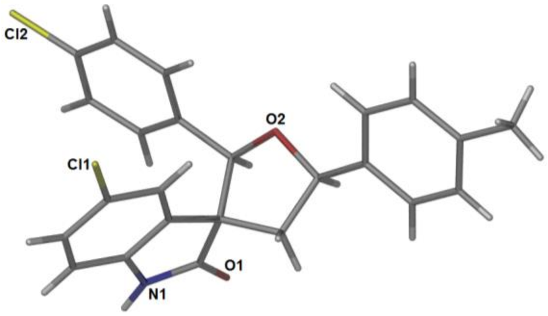

Supplementary Figure 14. X-ray diffraction structure of compound **8** (CCDC-1523855)

^{1}H NMR spectra of the reaction mixture of 3a with *N-p*-tolylsulfonyl oxindole 1e

During the reaction development, we could detect an intermediate by TLC analysis for the reaction of *N-p*-tolylsulfonyl oxindole **1e** and nitrone **3a**. Then we tried ^{1}H NMR analysis of the reaction course by using $CDCl_3$ as the solvent at 25 ºC, in the presence of 10 mol% $Ni(OTf)_2$. As shown below, an obvious peak at δ 5.55 formed and gradually disappeared with the passage of time. Meanwhile, the characteristic peak of cyclopropane **1e** at δ 5.88 gradually disappeared, showing the consumption of starting material, accompanied by the development of a peak at δ 5.43, correlating to the characteristic peak of the desire adduct **4e**. Unfortunately, we could not isolate this intermediate.

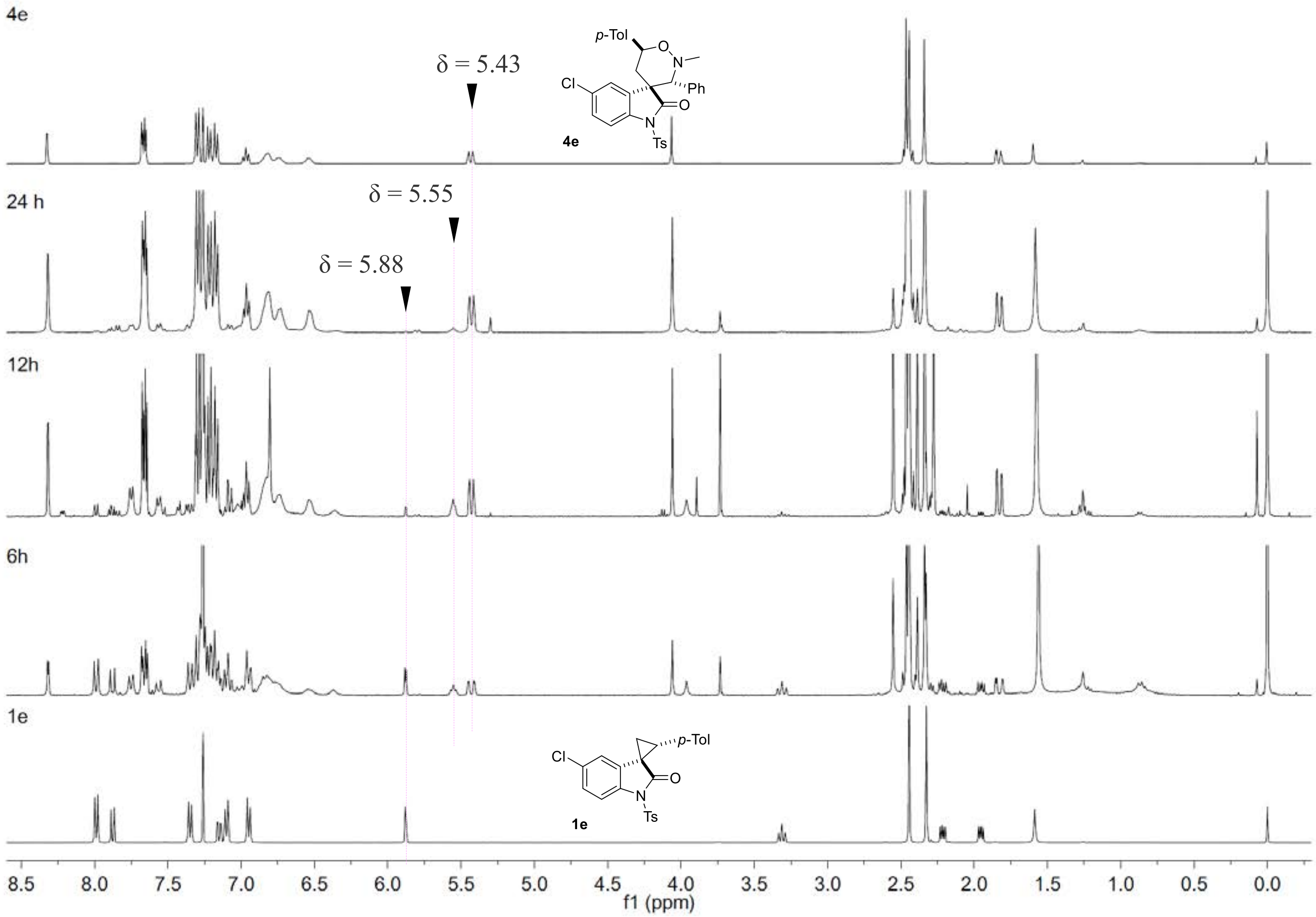

Supplementary Figure 15. ^{1}H NMR (400 MHz, $CDCl_3$) spectra of the reaction process of **3a** with *N-p*-tolylsulfonyl oxindole **1e**

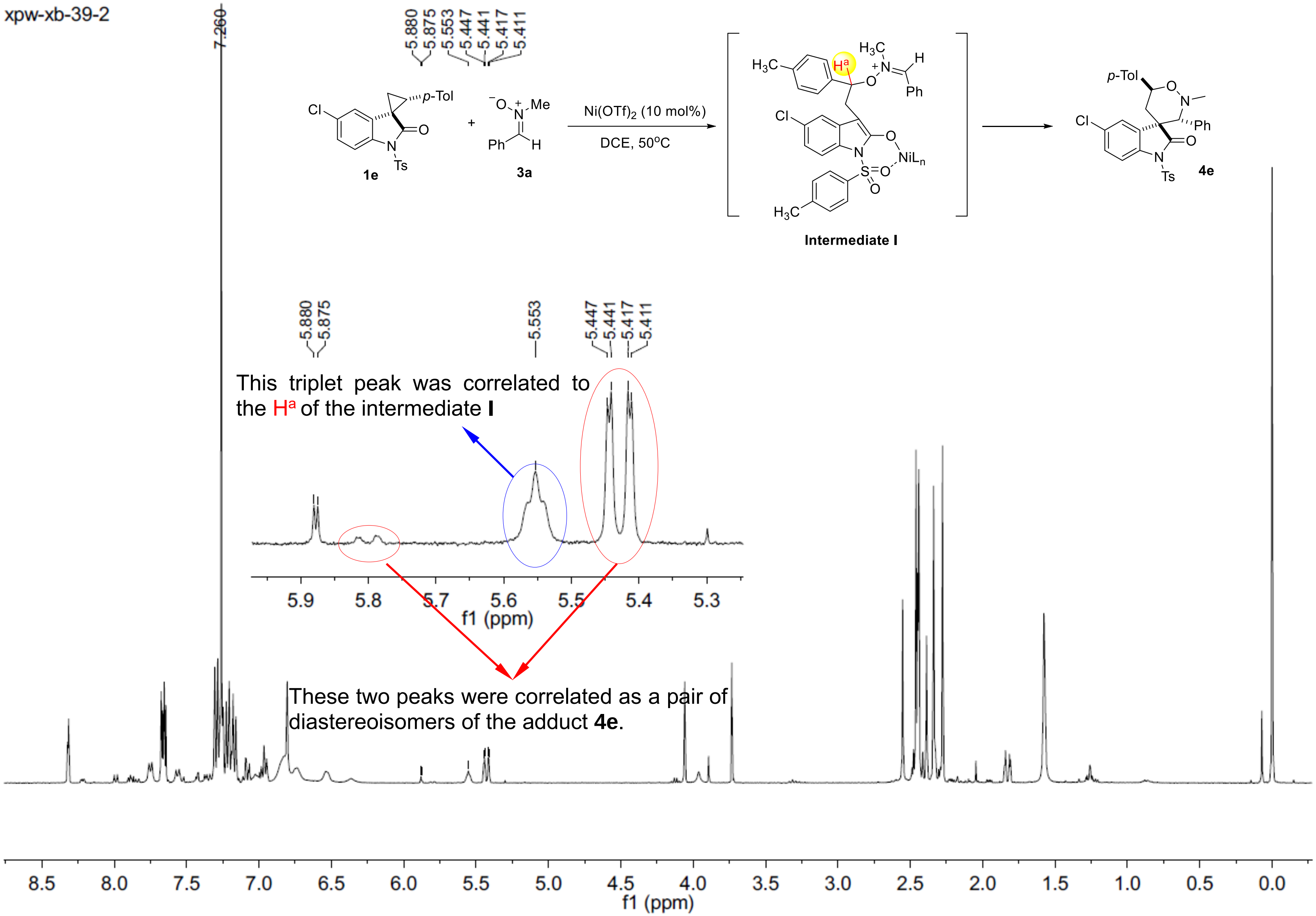

Supplementary Figure 16. ^{1}H NMR (400 MHz, $CDCl_3$) spectra of the reaction mixture of **3a** with *N*-p-tolylsulfonyl oxindole **1e**

NMR spectra

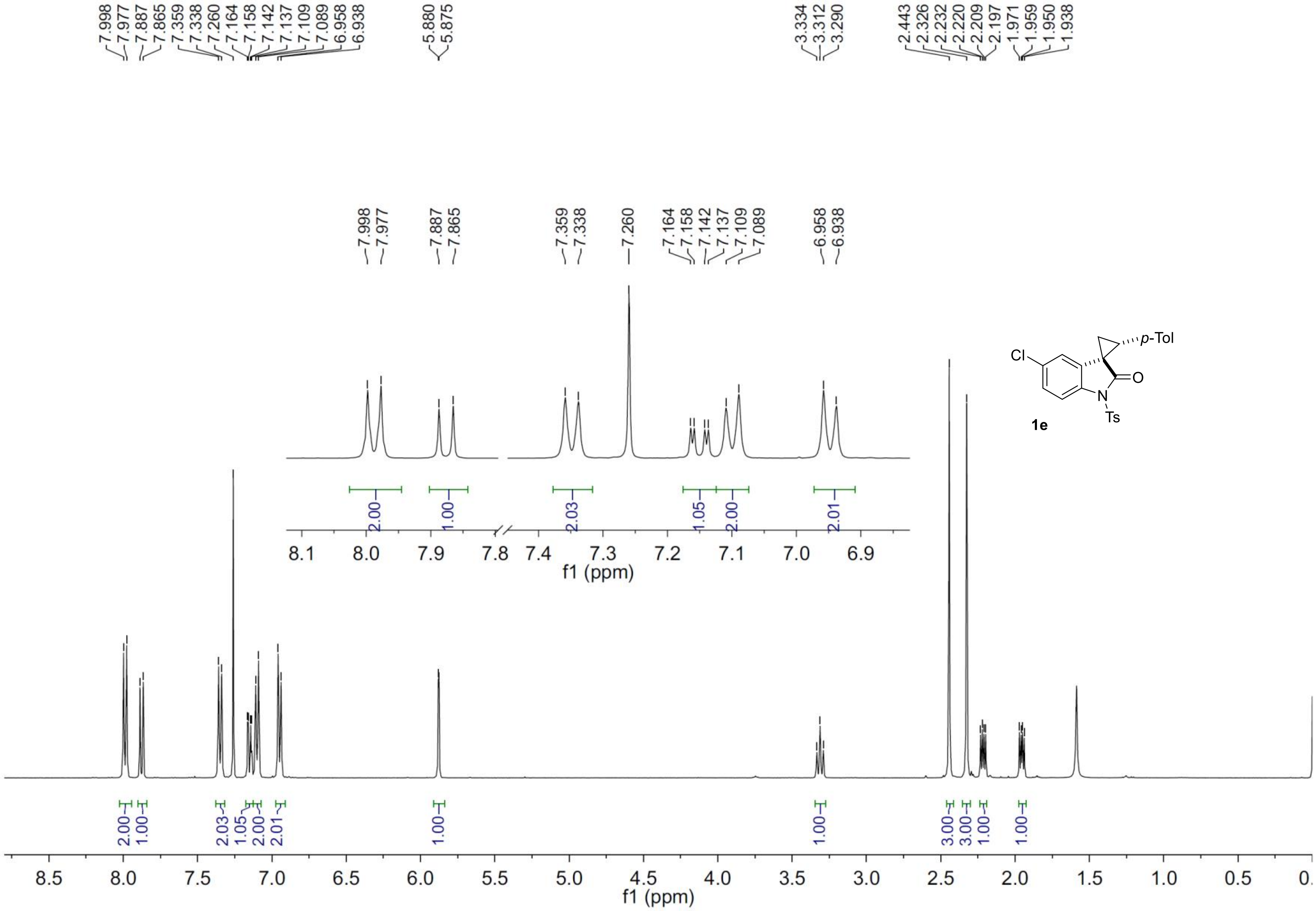

Supplementary Figure 17. ^{1}H NMR (400 MHz, $CDCl_3$) spectra for compound **1e**

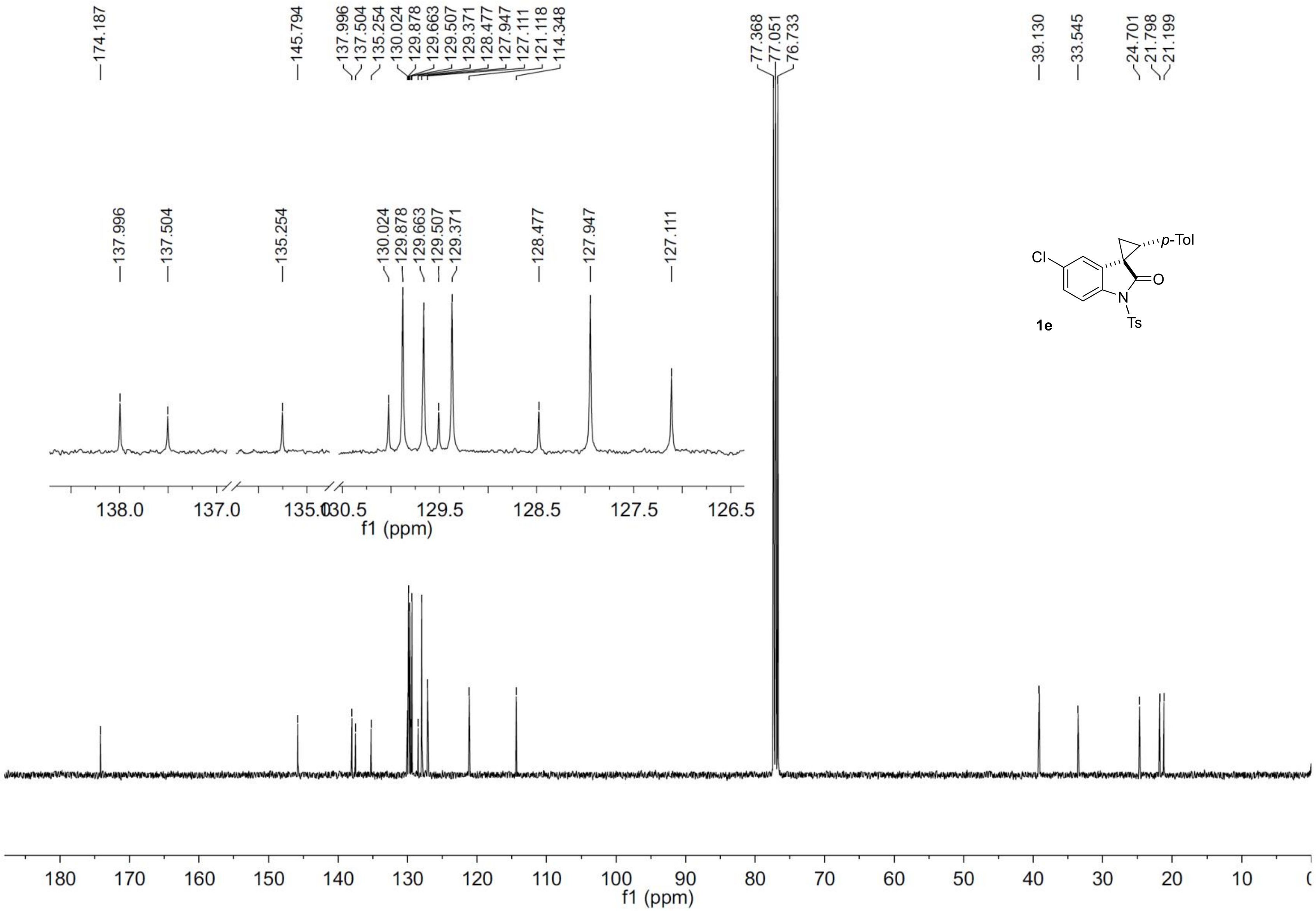

Supplementary Figure 18. ^{13}C NMR (100 MHz, $CDCl_3$) spectra for compound **1e**

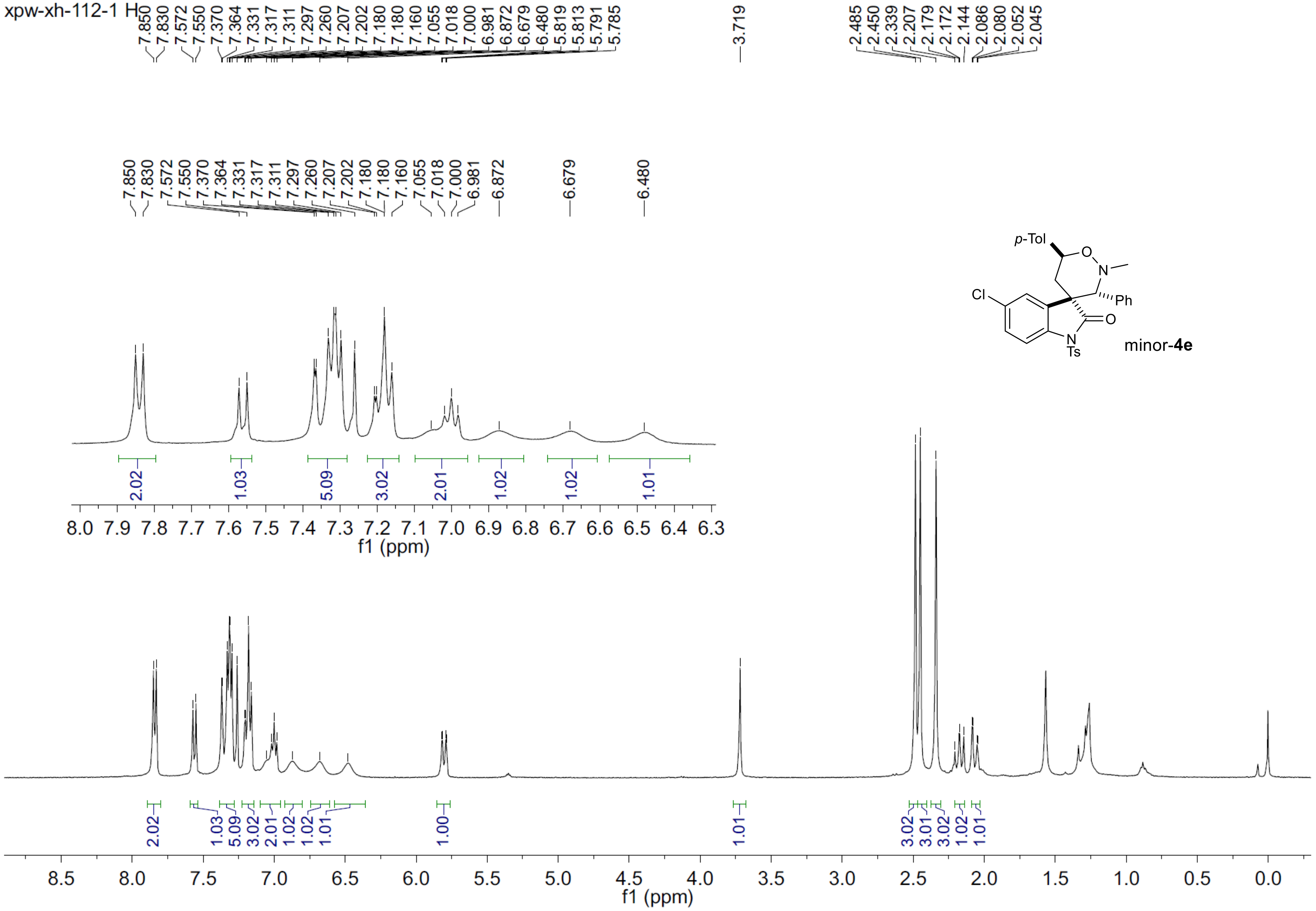

Supplementary Figure 19. ^{1}H NMR (400 MHz, $CDCl_3$) spectra for compound minor-**4e**

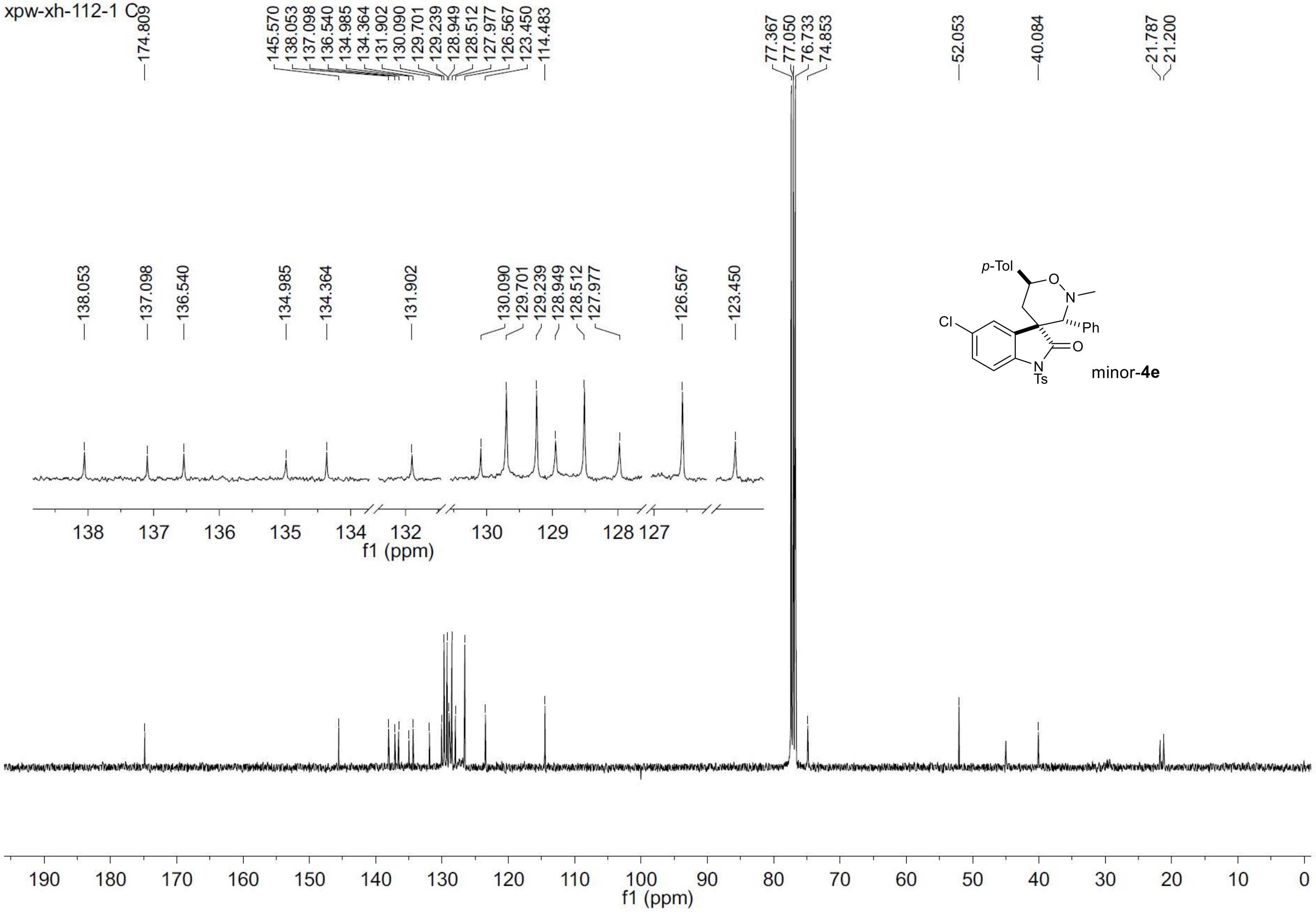

Supplementary Figure 20. ^{13}C NMR (100 MHz, $CDCl_3$) spectra for compound minor-**4e**

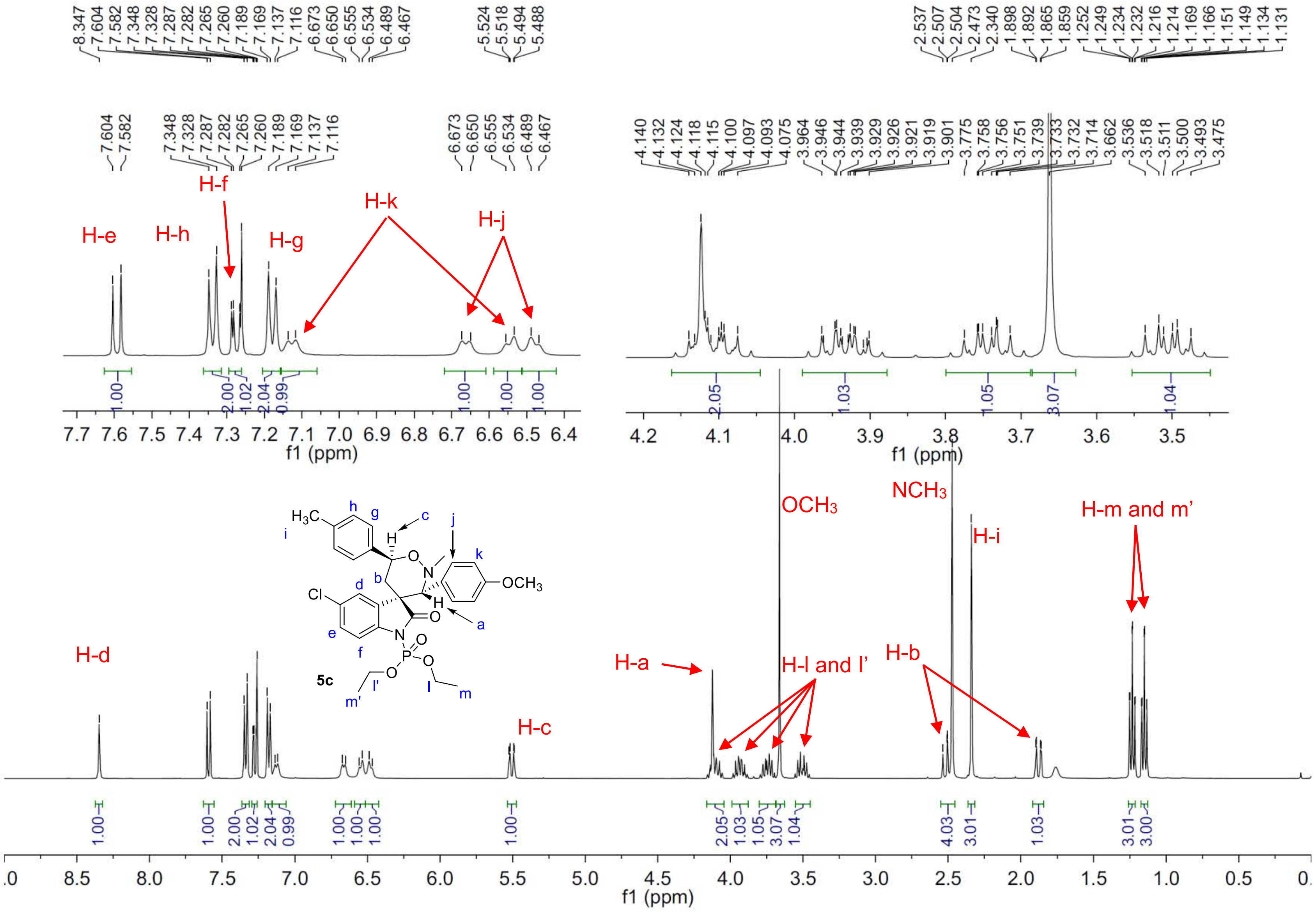

Supplementary Figure 21. ^{1}H NMR (400 MHz, $CDCl_3$) spectra for compound **5c**

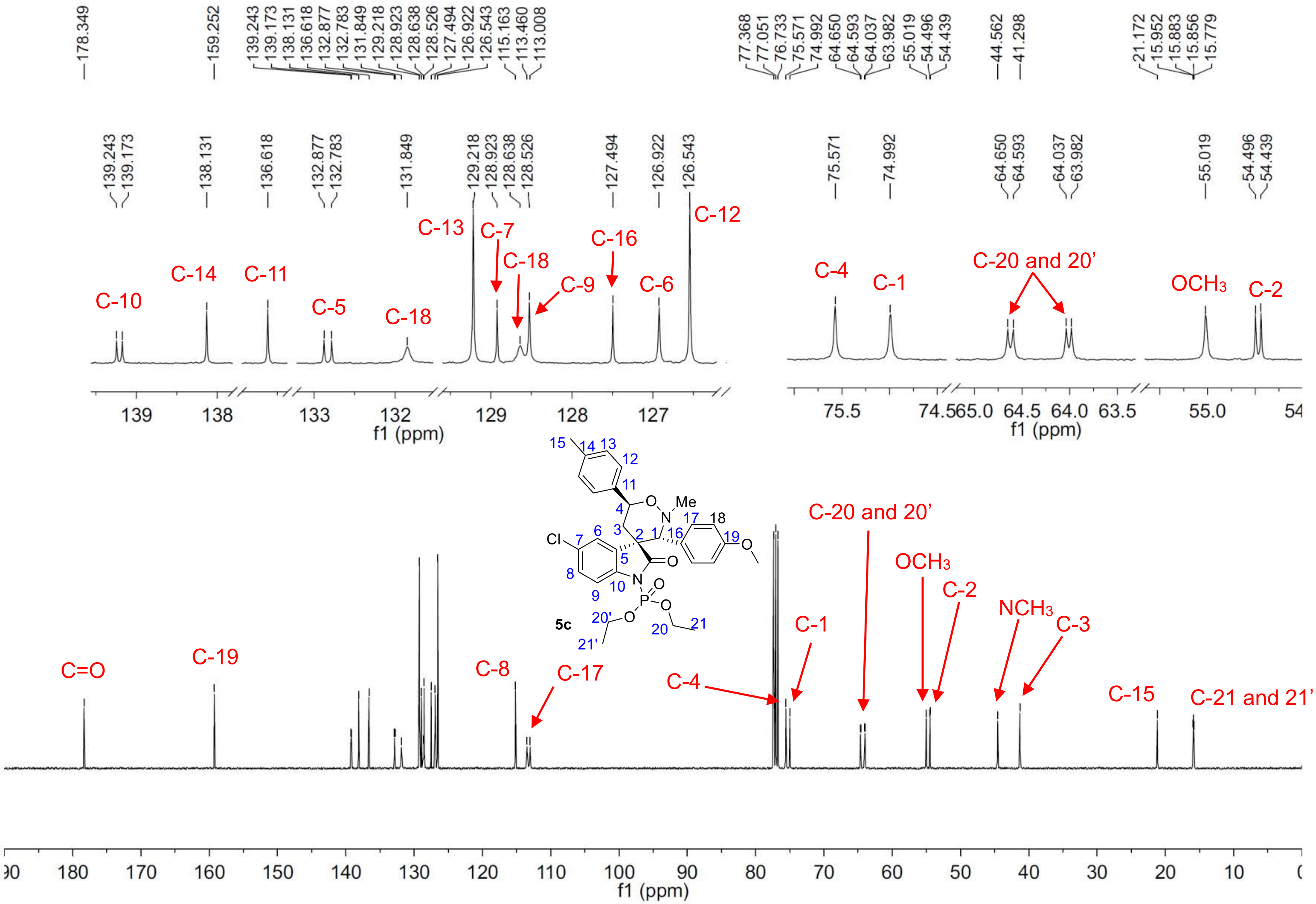

Supplementary Figure 22. ^{13}C NMR (100 MHz, $CDCl_3$) spectra for compound **5c**

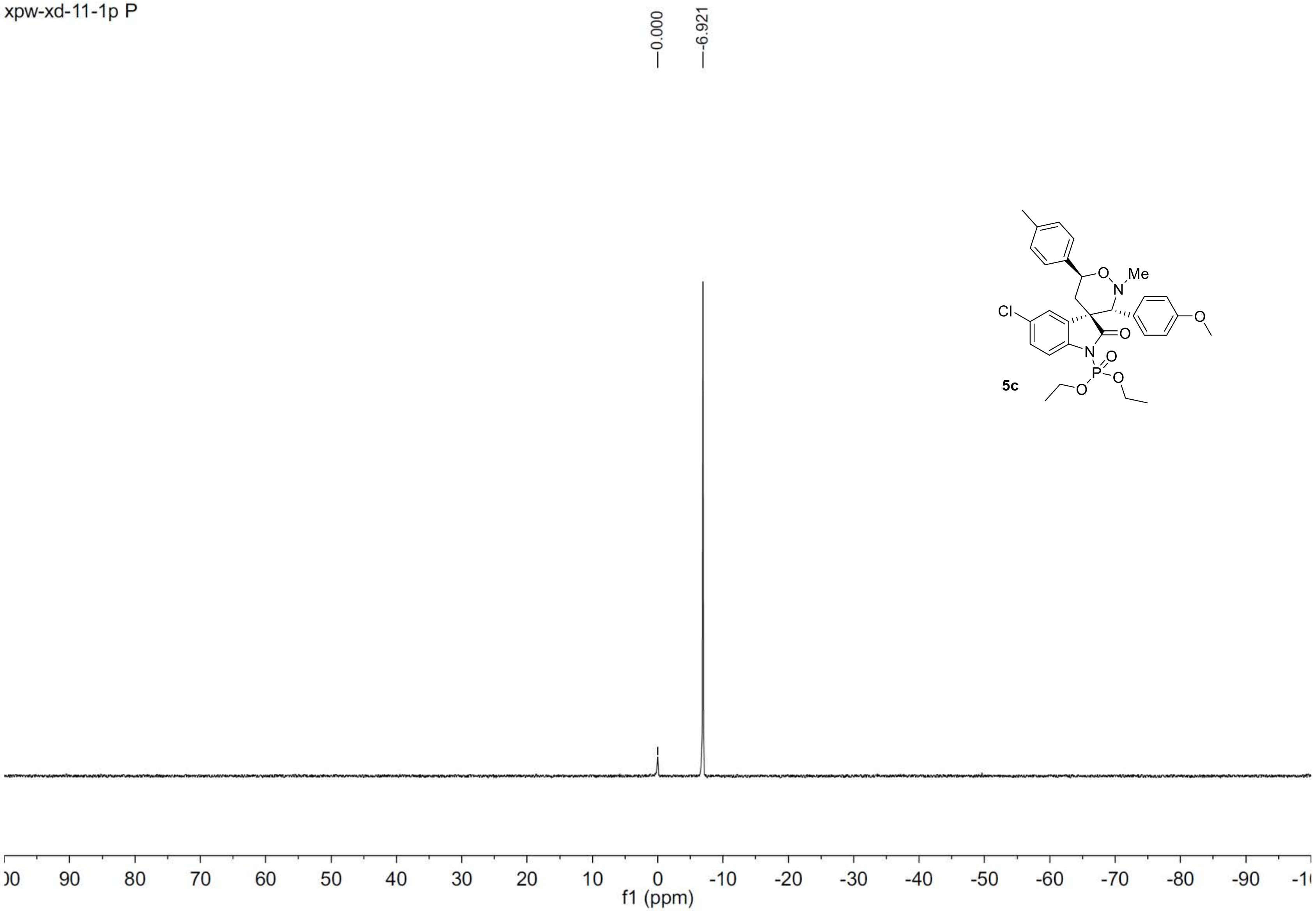

Supplementary Figure 23. ^{31}P NMR (162 MHz, $CDCl_3$) spectra for compound **5c**

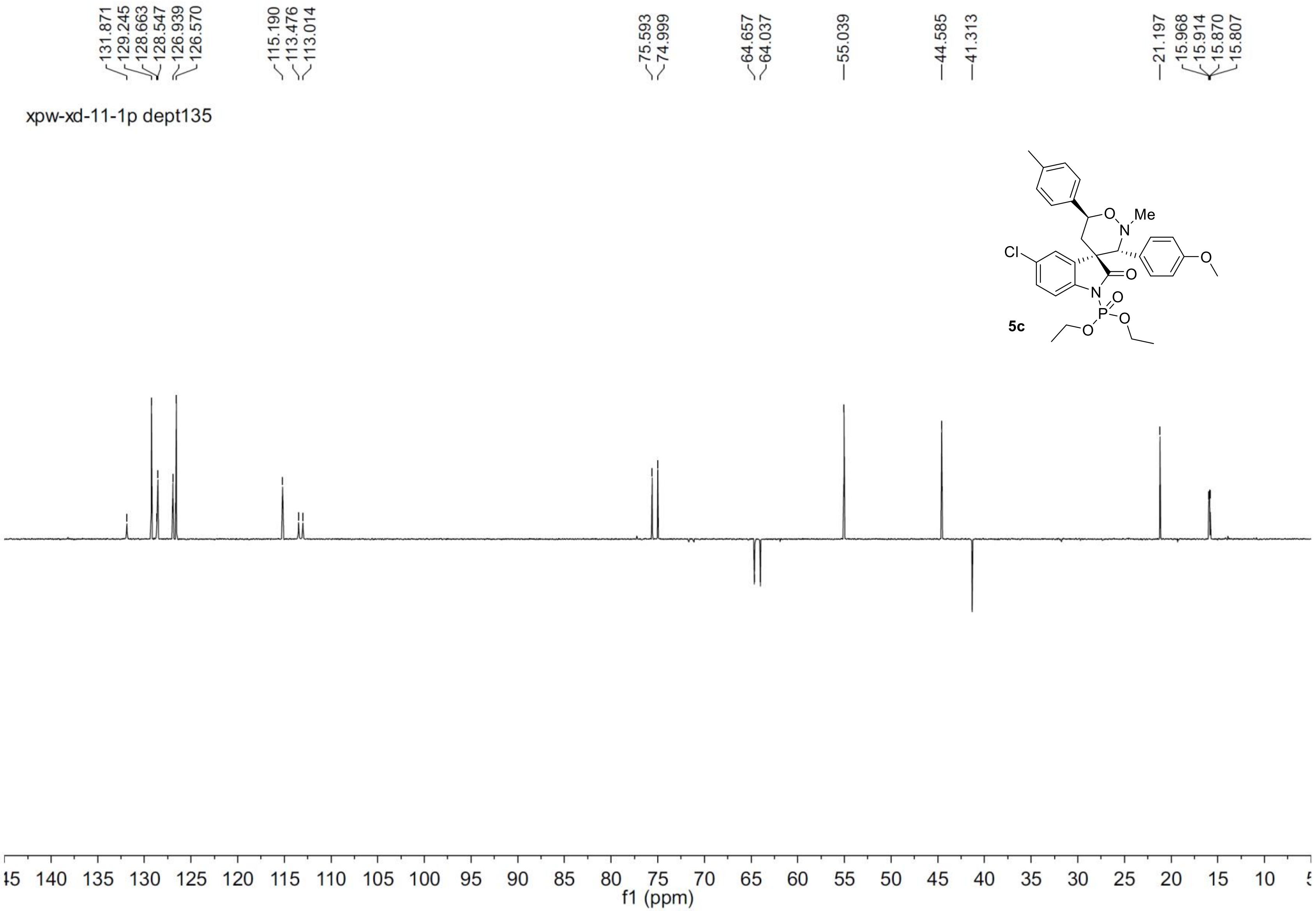

Supplementary Figure 24. DEPT 135° (100 MHz, $CDCl_3$) spectra for compound **5c**

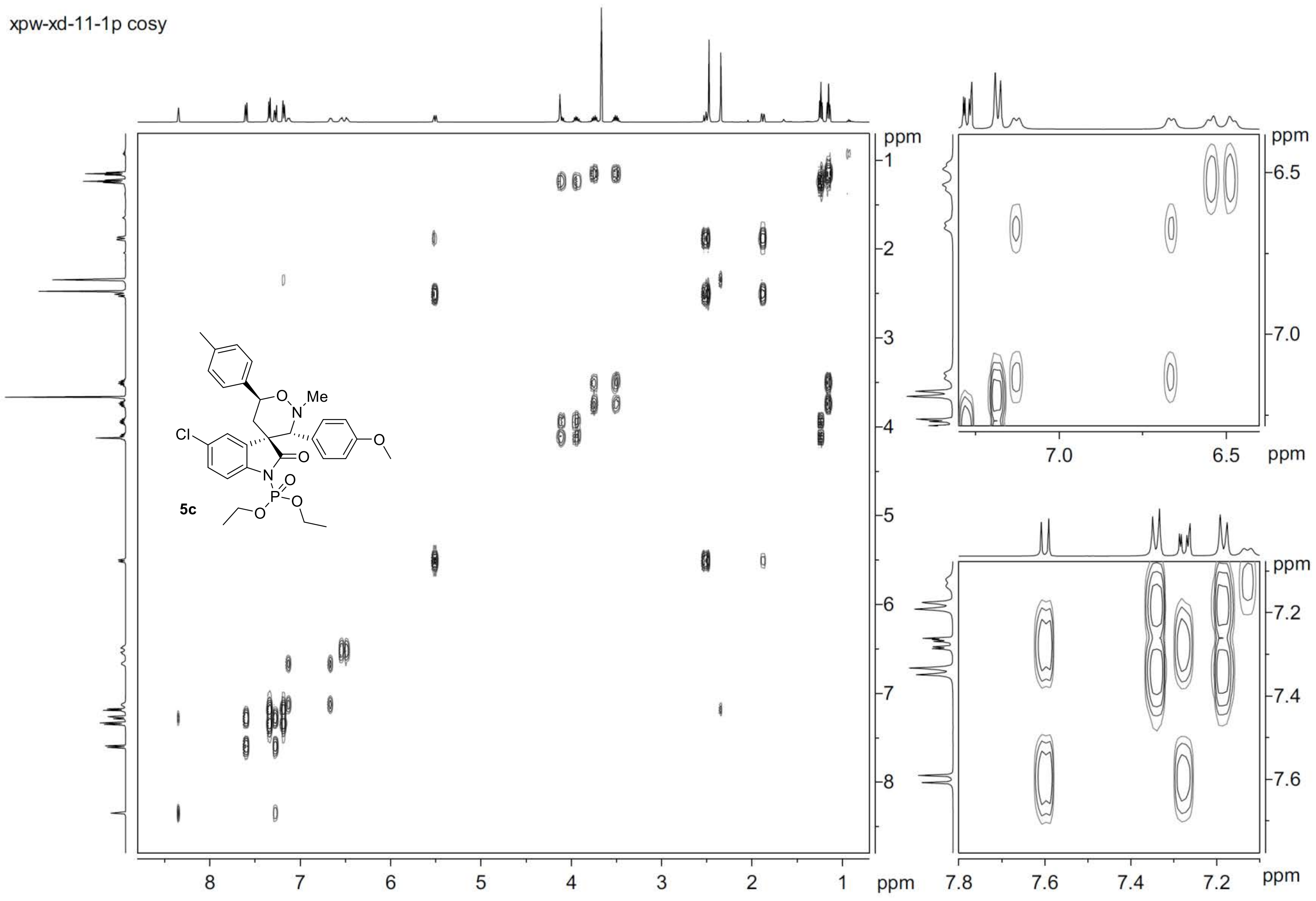

Supplementary Figure 25. COSY spectra for compound **5c**

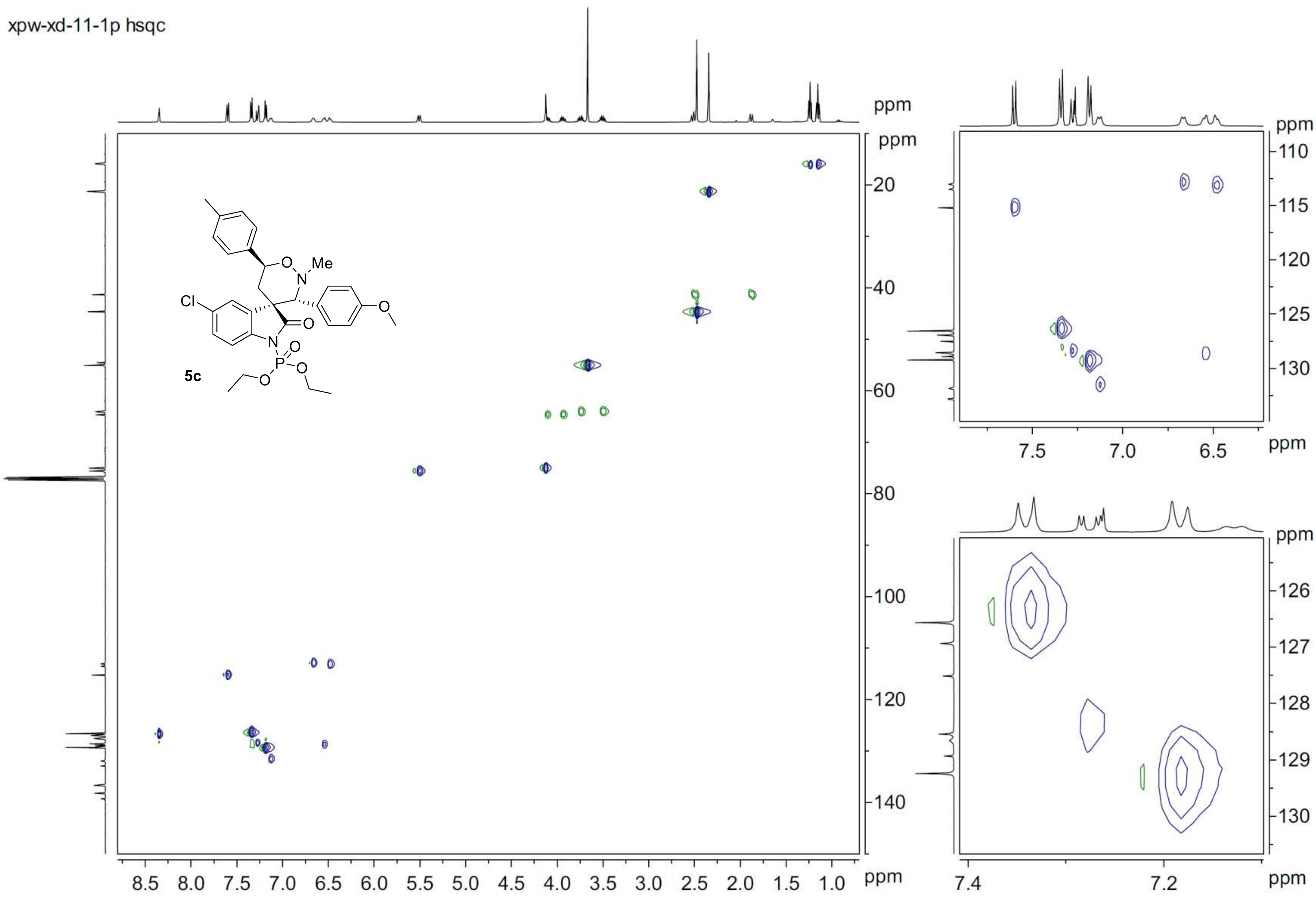

Supplementary Figure 26. HSQC spectra for compound **5c**

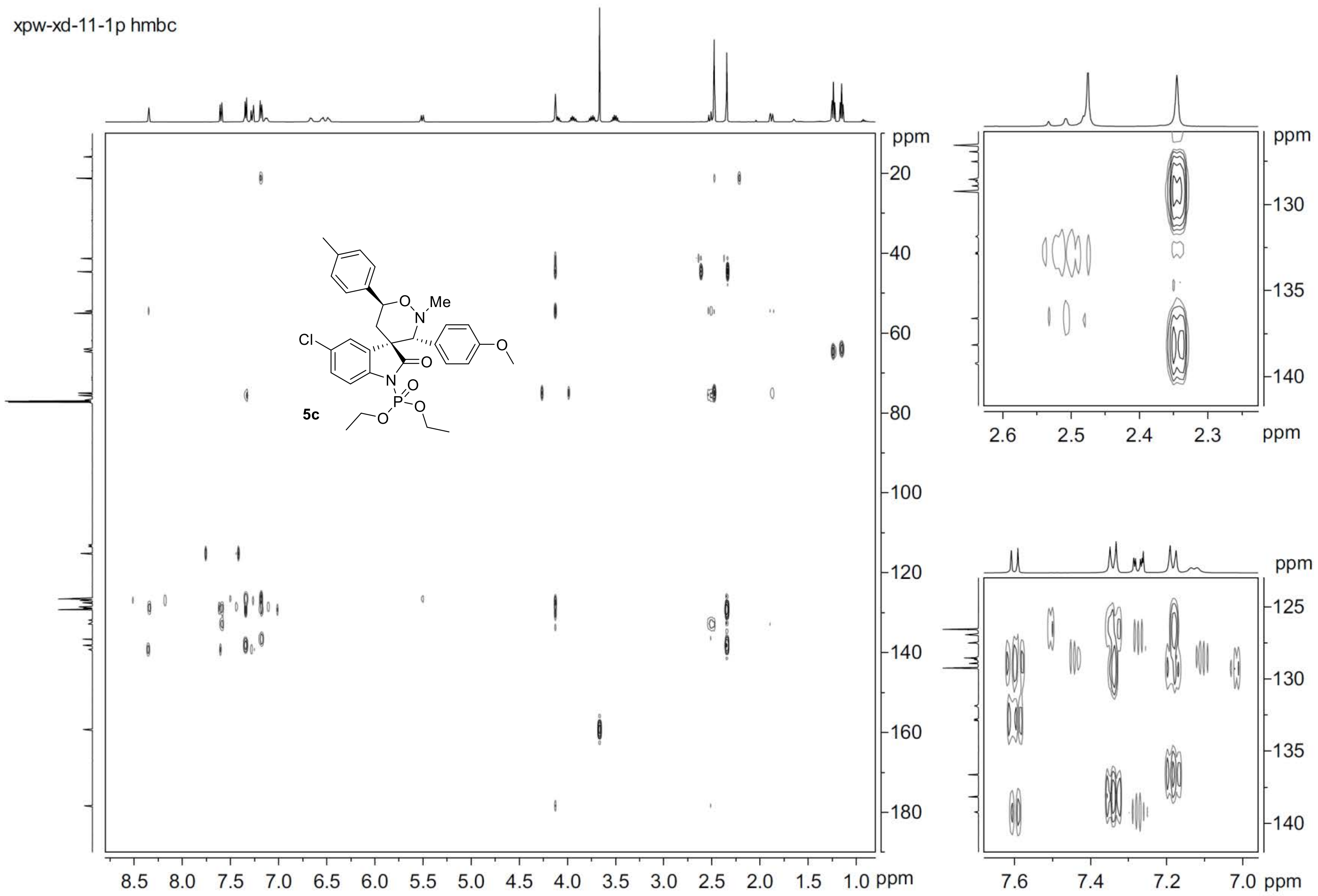

Supplementary Figure 27. HMBC spectra for compound **5c**

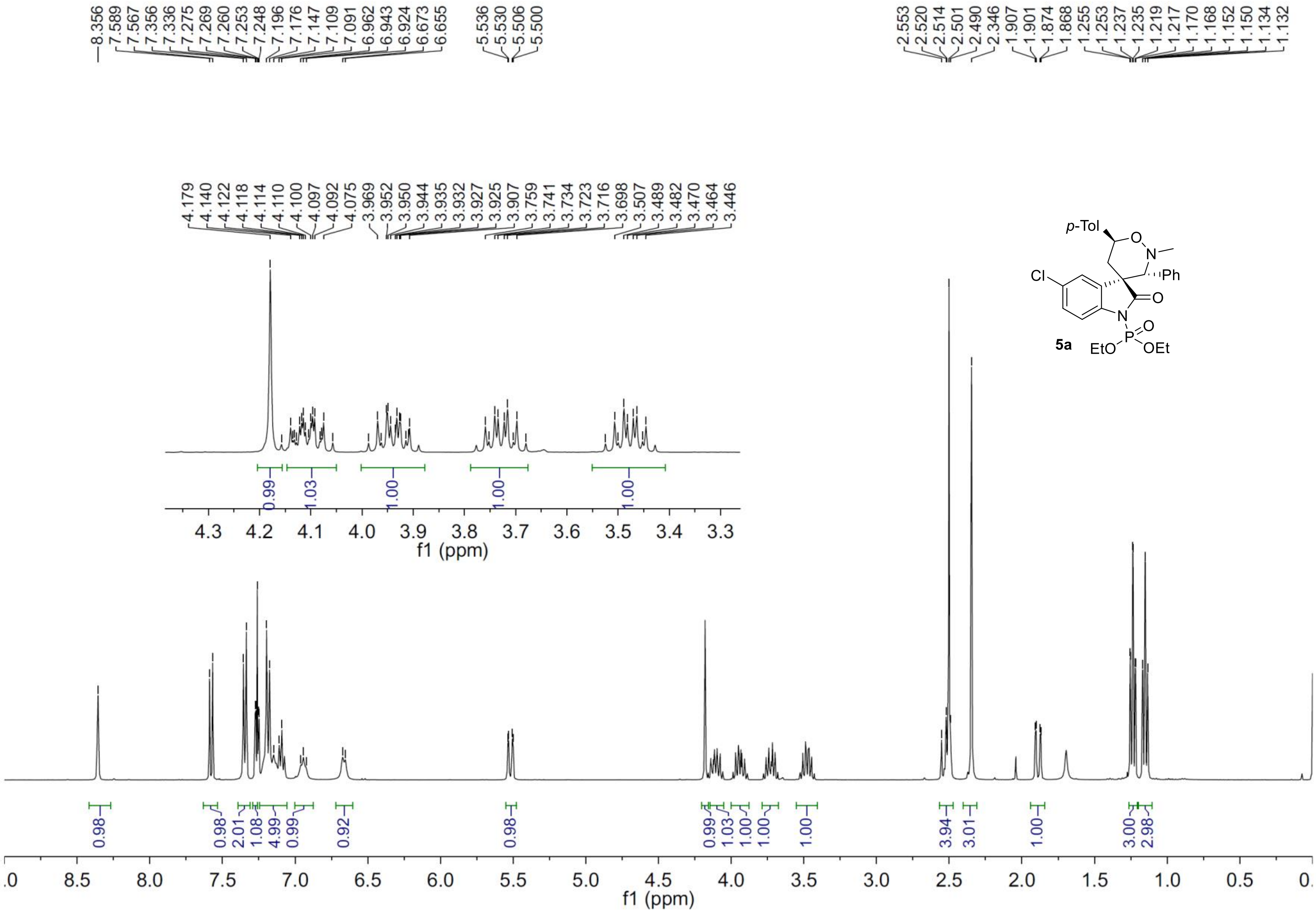

Supplementary Figure 28. ^{1}H NMR (400 MHz, $CDCl_3$) spectra for compound **5a**

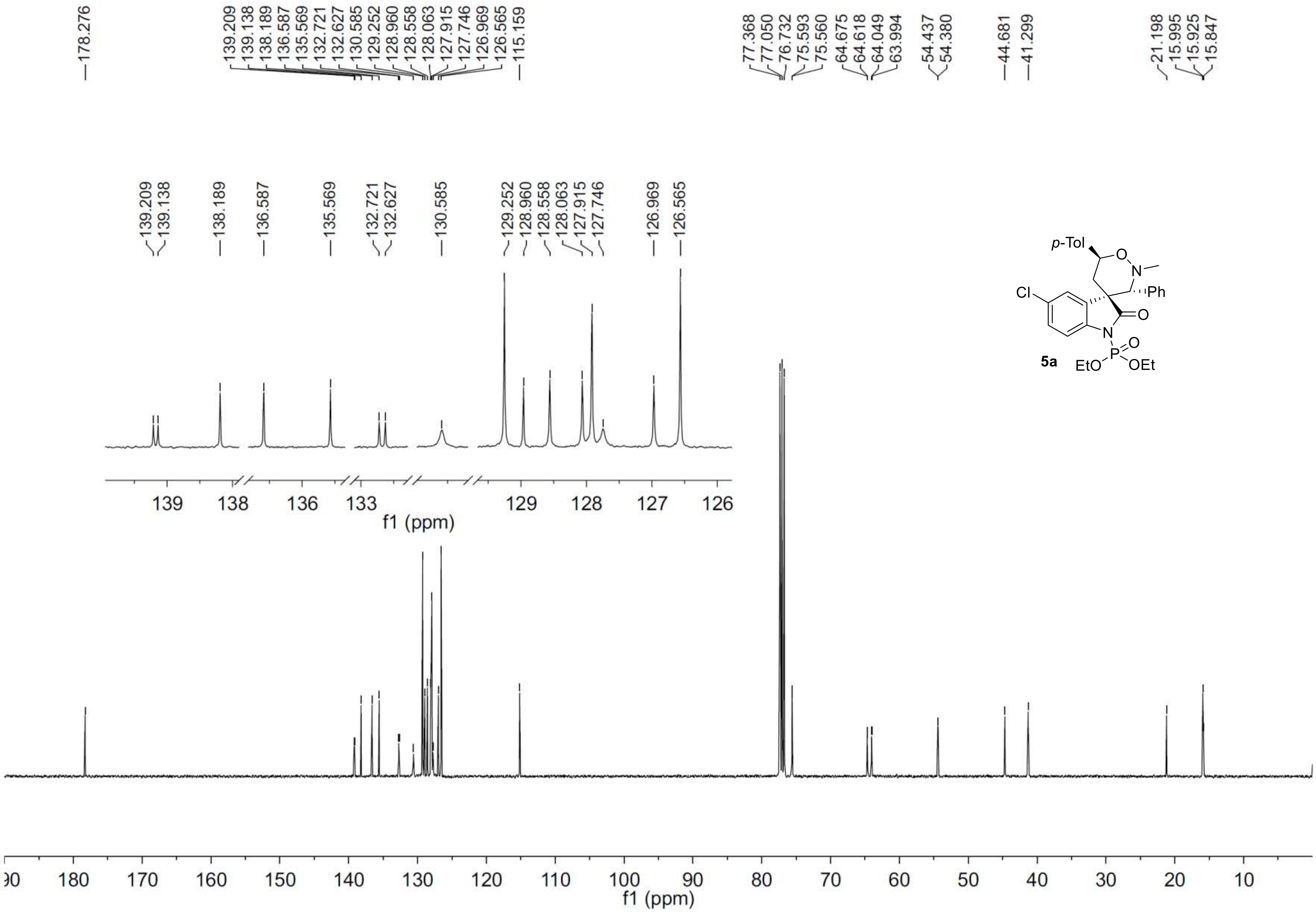

Supplementary Figure 29. ^{13}C NMR (100 MHz, $CDCl_3$) spectra for compound **5a**

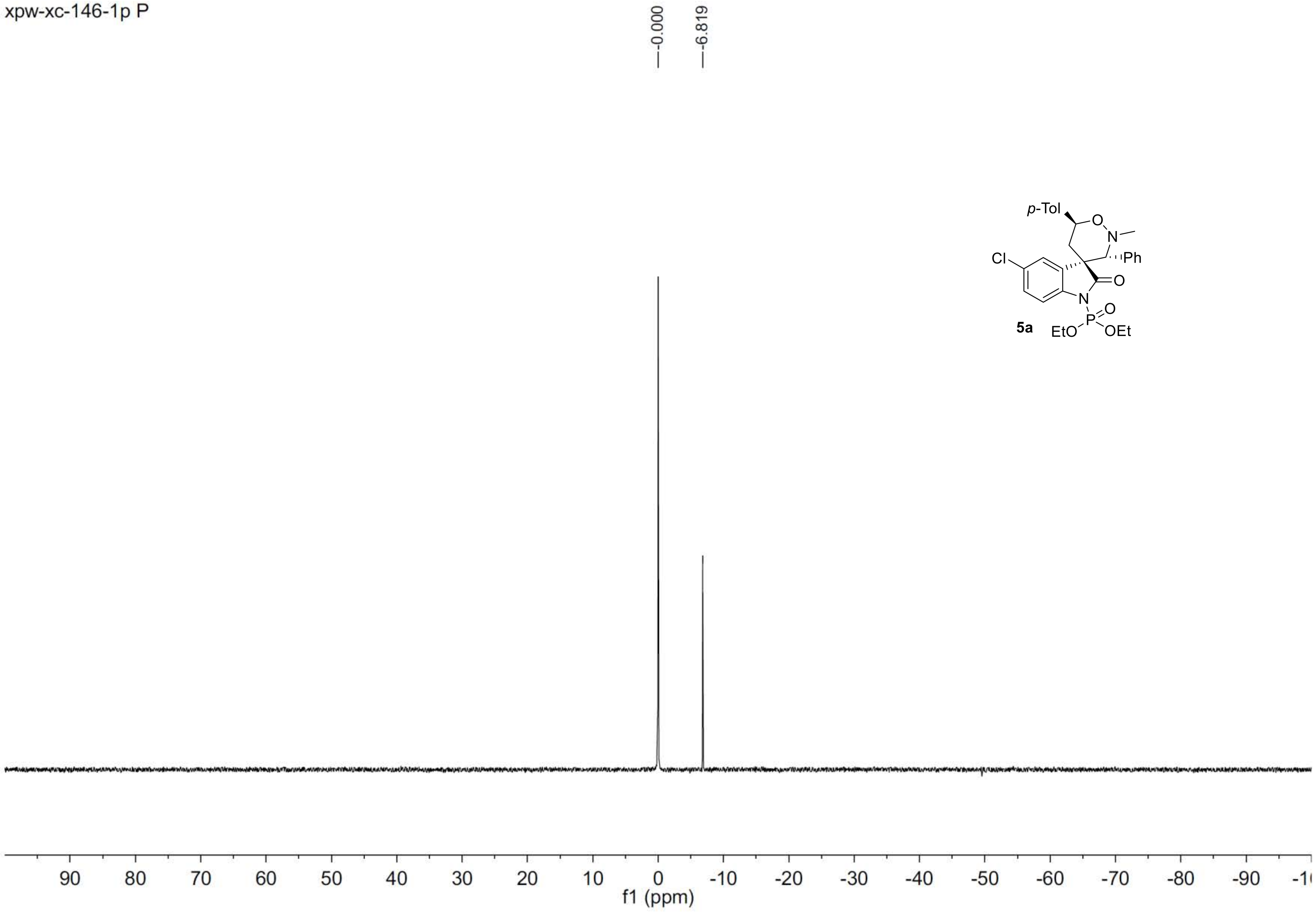

Supplementary Figure 30. ^{31}P NMR (122 MHz, $CDCl_3$) spectra for compound **5a**

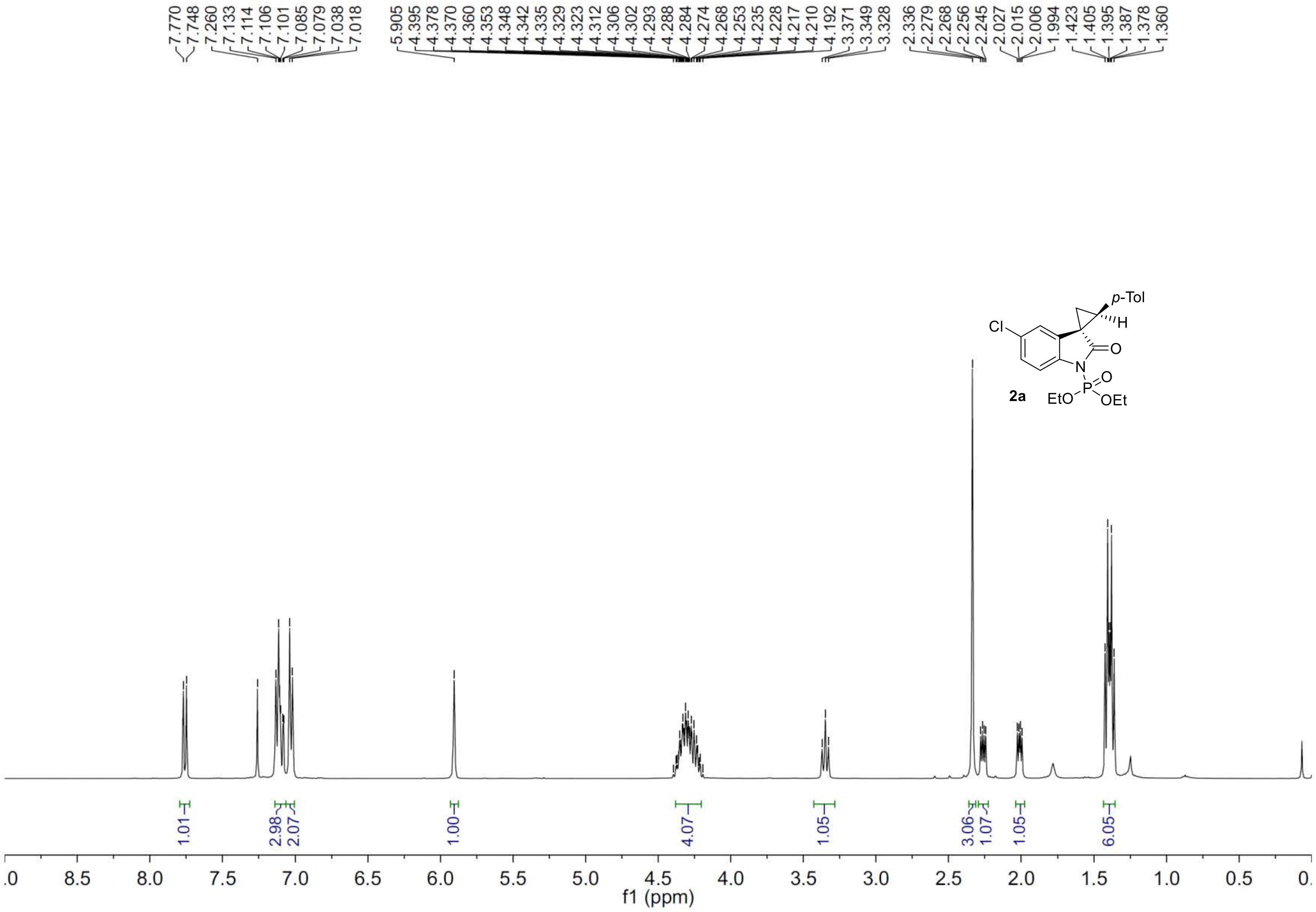

Supplementary Figure 31. ^{1}H NMR (400 MHz, $CDCl_3$) spectra for compound **2a**

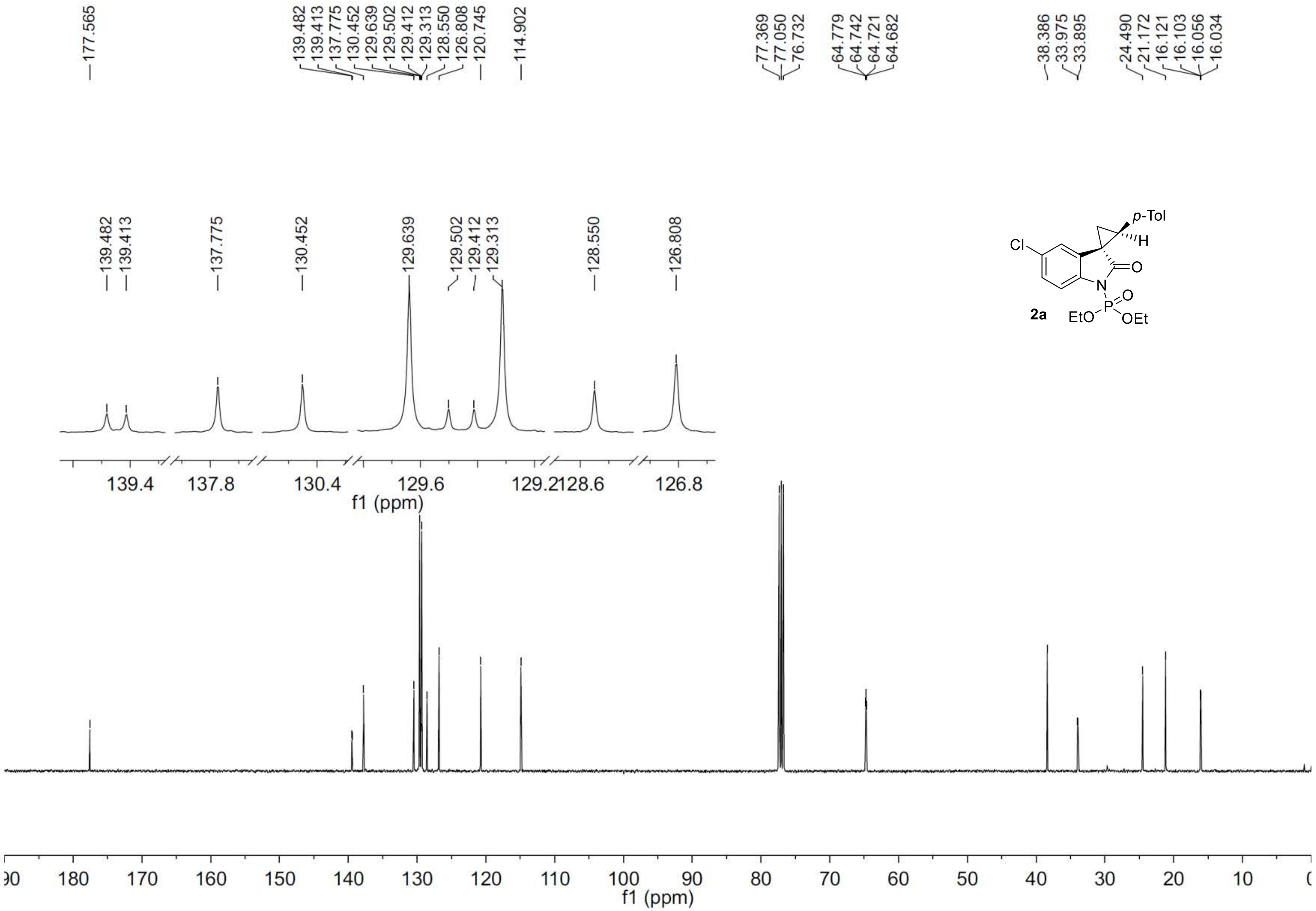

Supplementary Figure 32. ^{13}C NMR (100 MHz, $CDCl_3$) spectra for compound **2a**

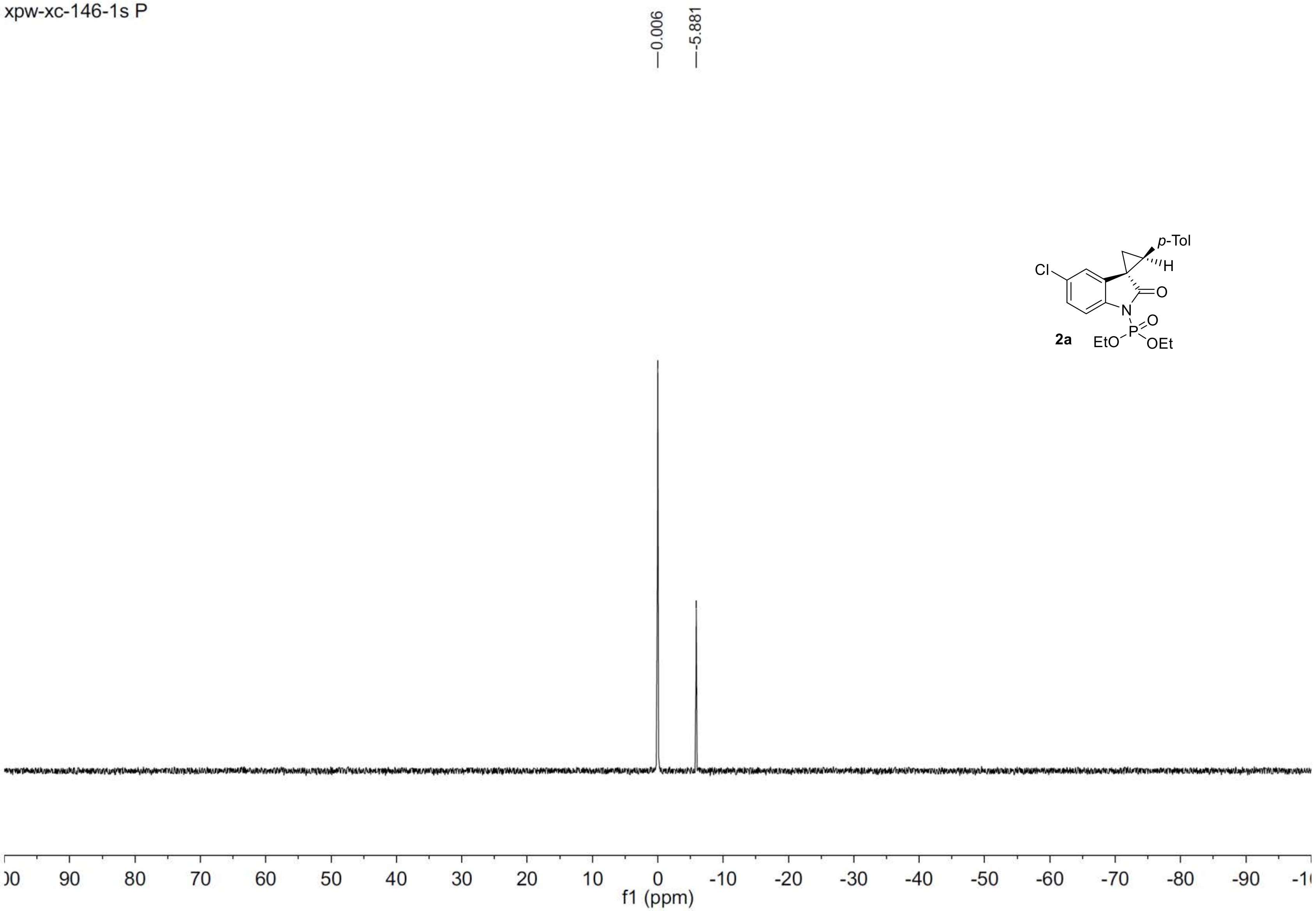

2a

Supplementary Figure 33. ^{31}P NMR (122 MHz, $CDCl_3$) spectra for compound **2a**

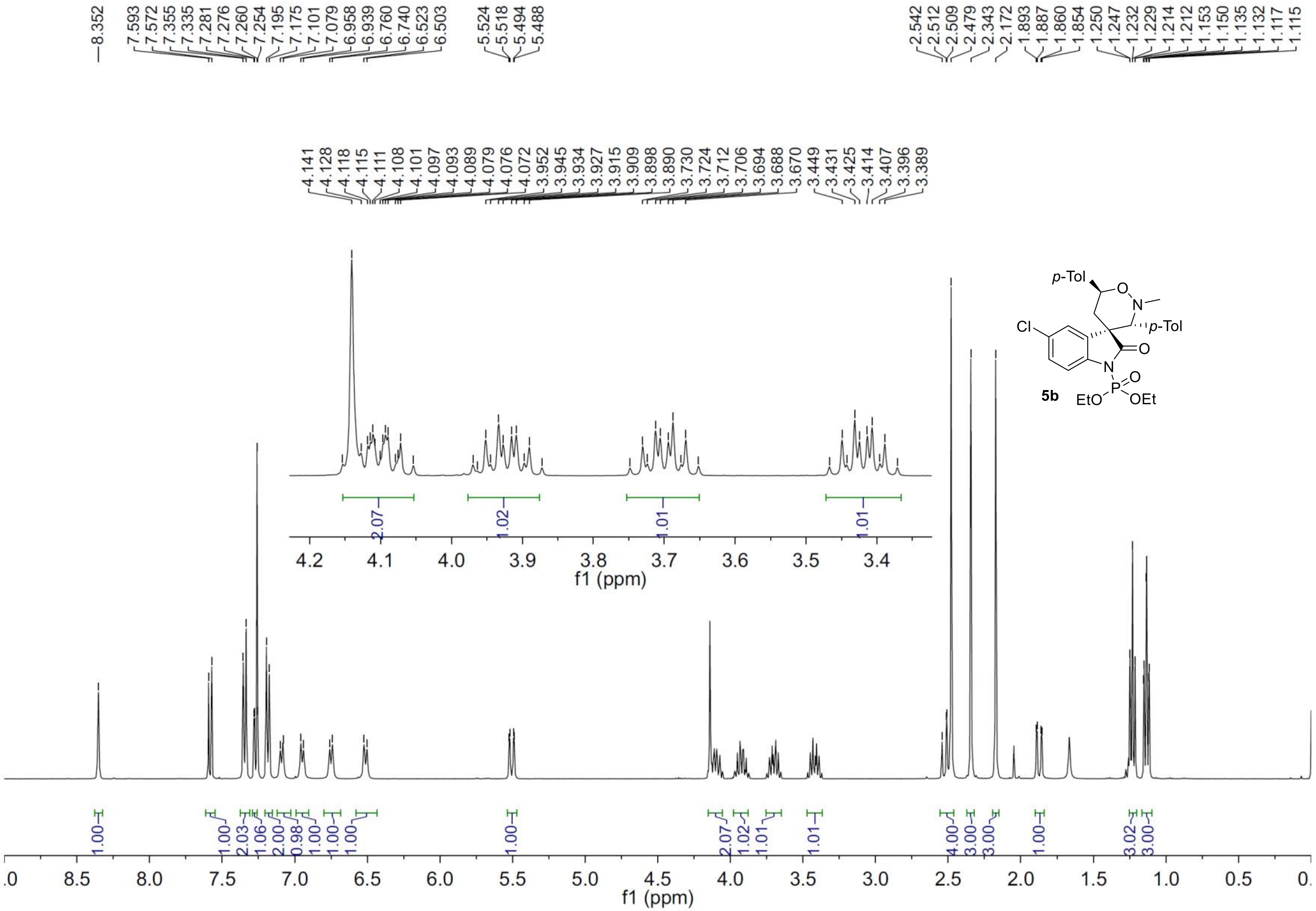

Supplementary Figure 34. ^{1}H NMR (400 MHz, $CDCl_3$) spectra for compound **5b**

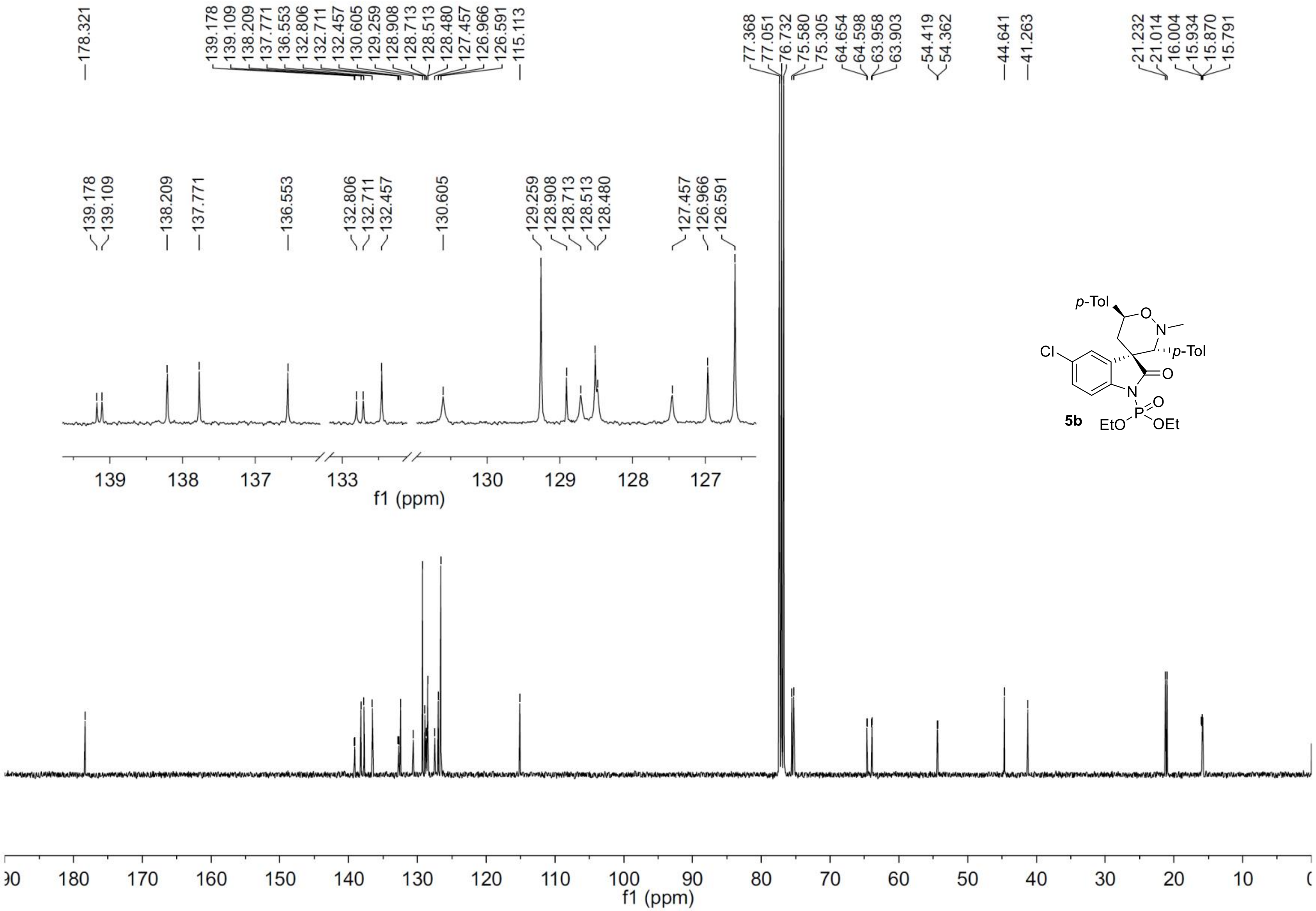

Supplementary Figure 35. ^{13}C NMR (100 MHz, $CDCl_3$) spectra for compound **5b**

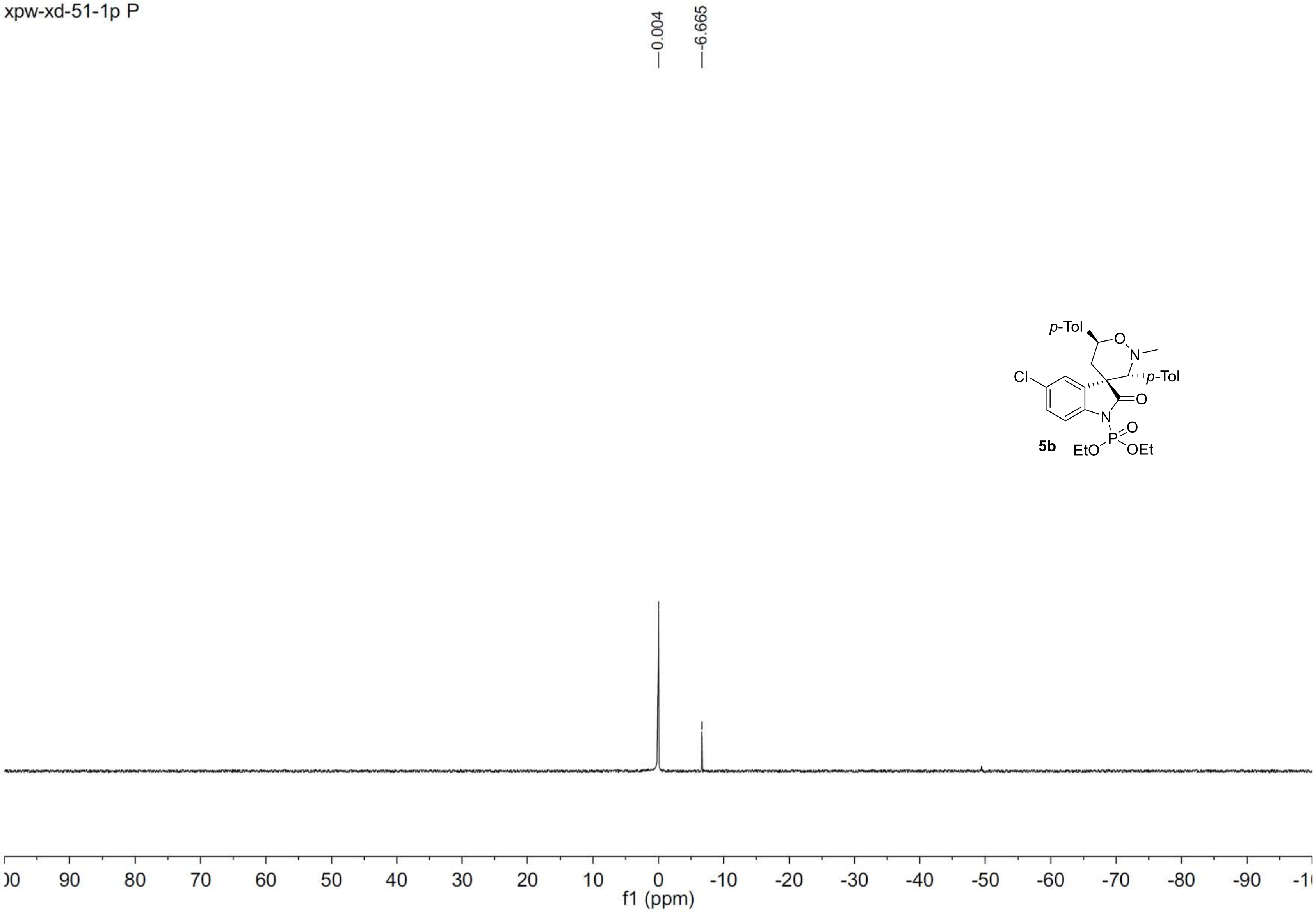

Supplementary Figure 36. ^{31}P NMR (162 MHz, $CDCl_3$) spectra for compound **5b**

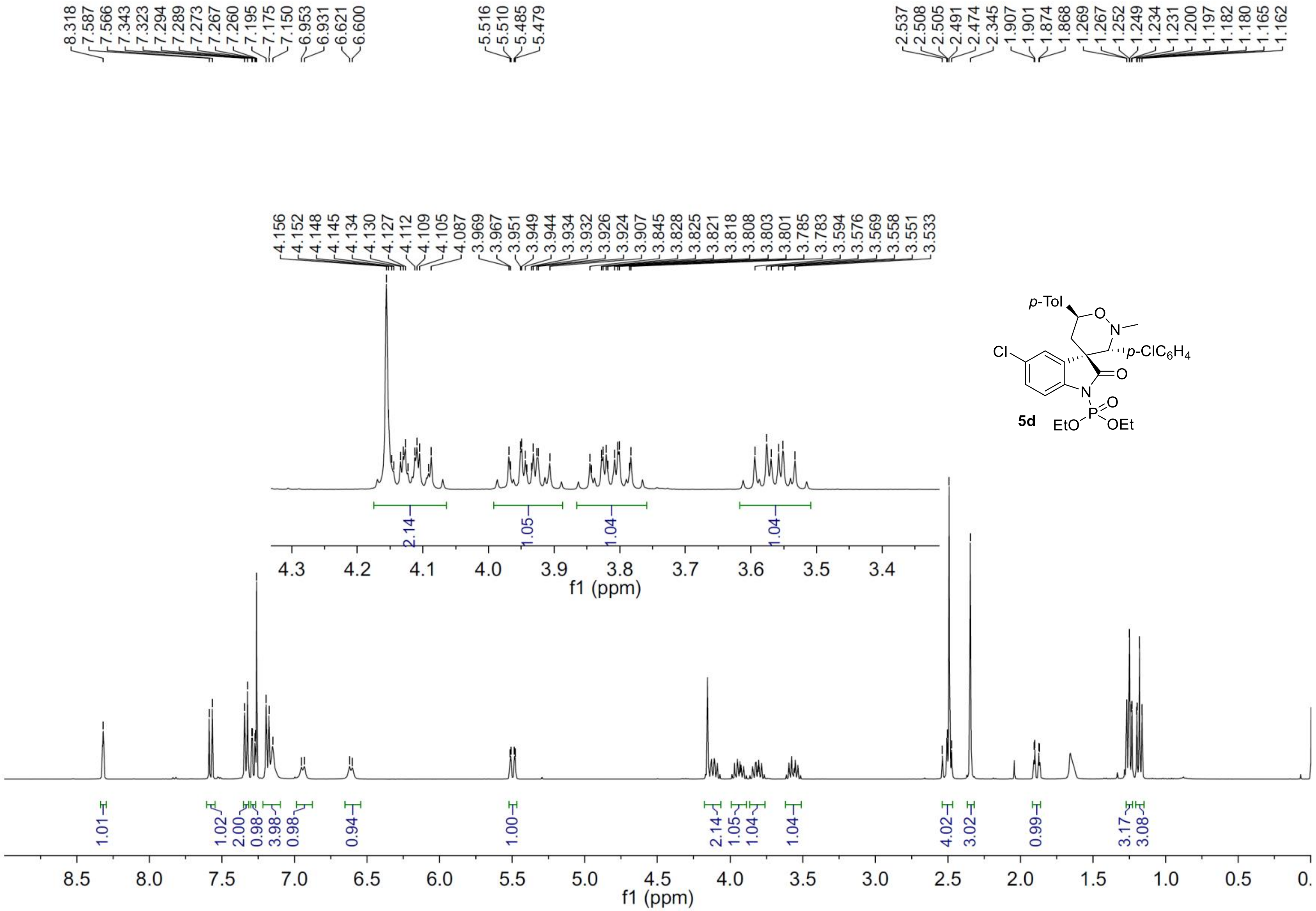

Supplementary Figure 37. ^{1}H NMR (400 MHz, $CDCl_3$) spectra for compound **5d**

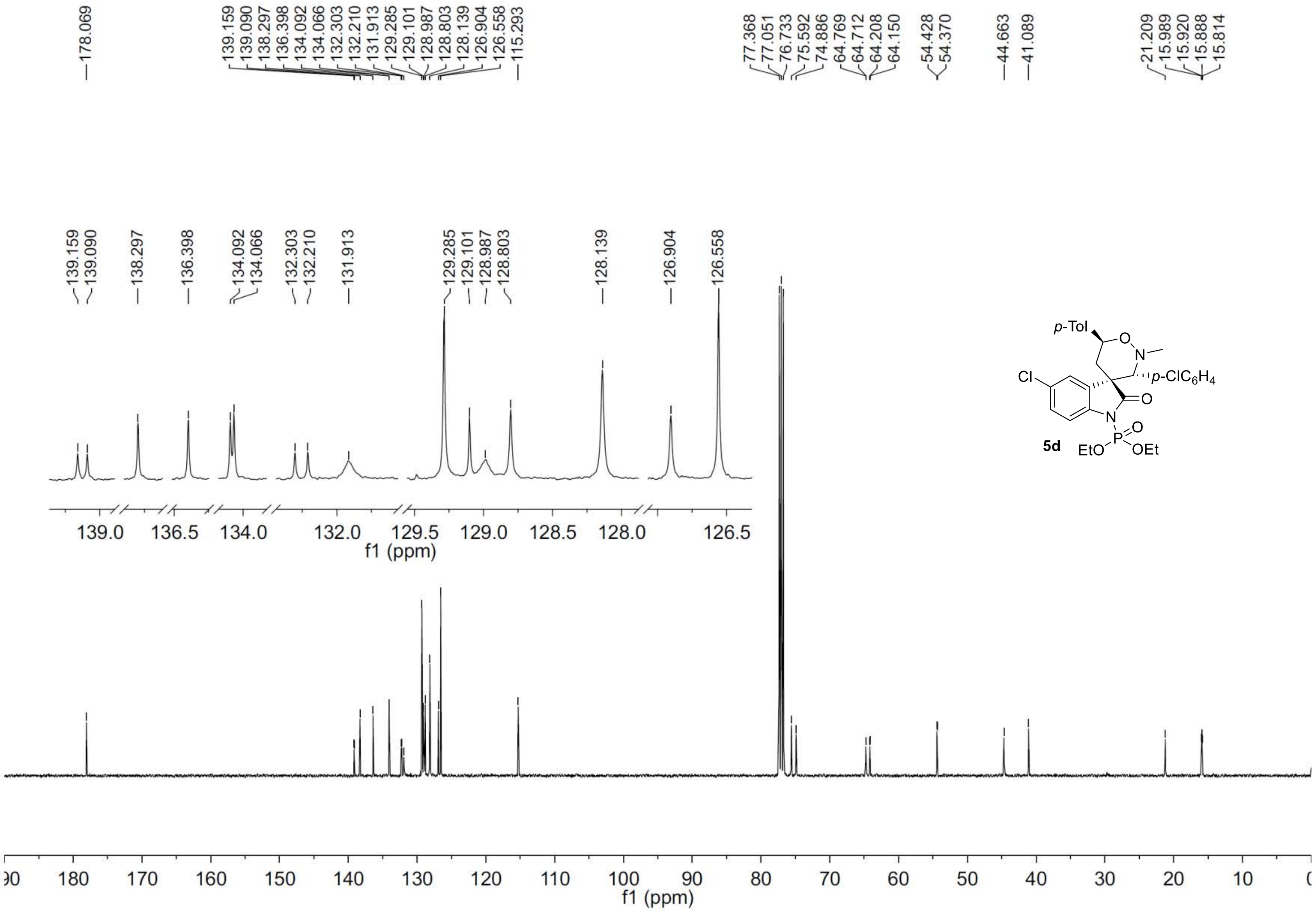

Supplementary Figure 38. ^{13}C NMR (100 MHz, $CDCl_3$) spectra for compound **5d**

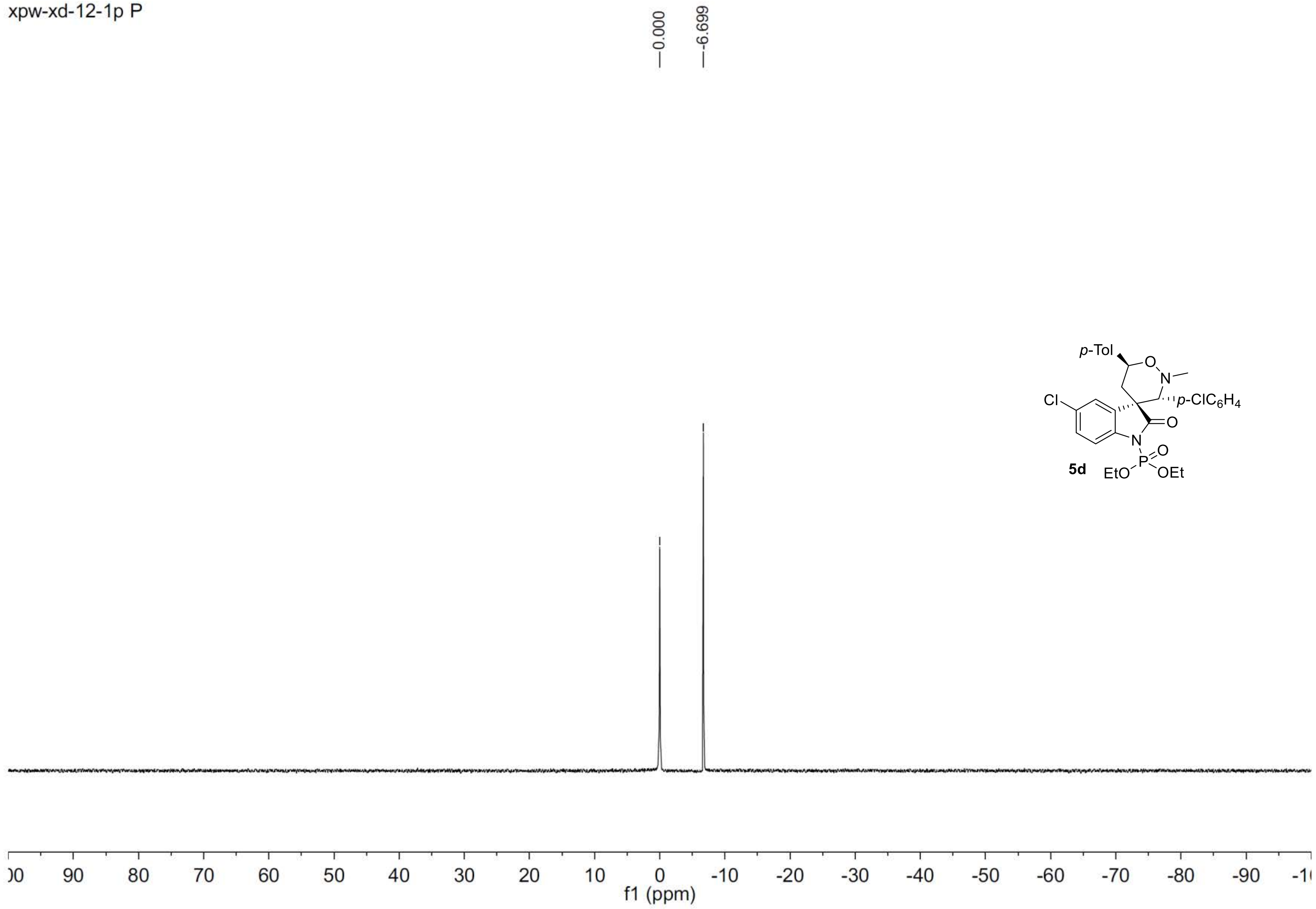

Supplementary Figure 39. ^{31}P NMR (162 MHz, $CDCl_3$) spectra for compound **5d**

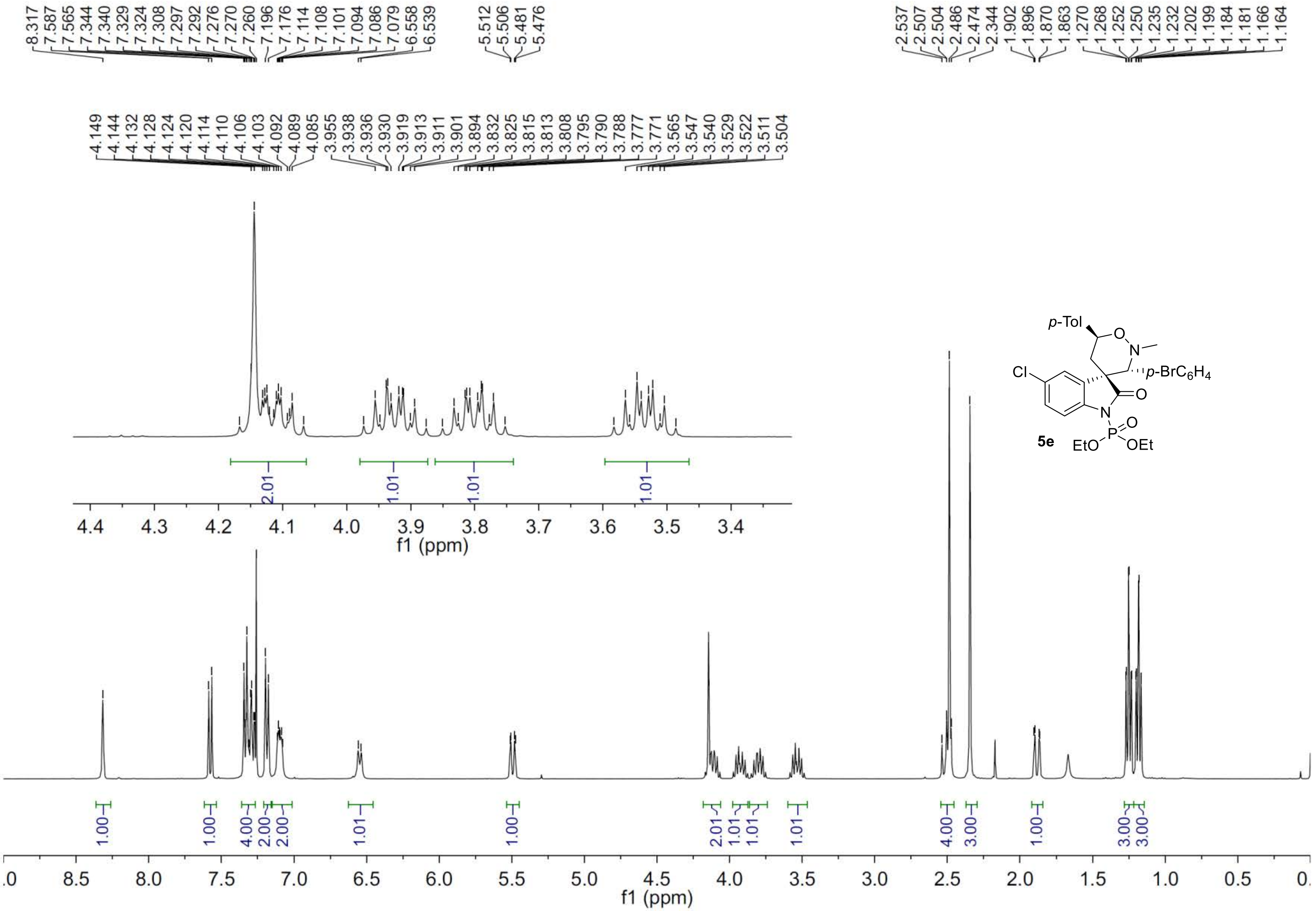

Supplementary Figure 40. ^{1}H NMR (400 MHz, $CDCl_3$) spectra for compound **5e**

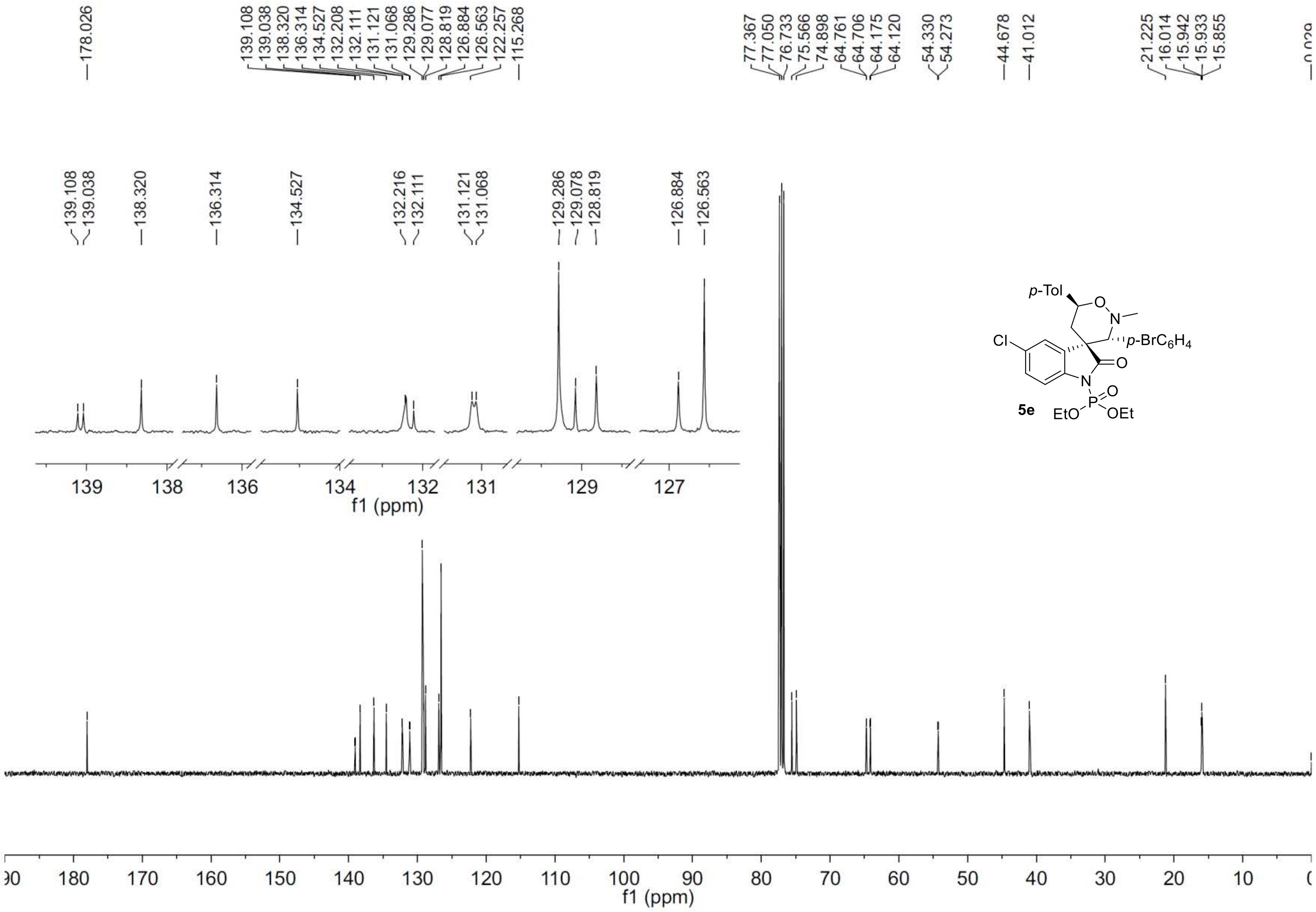

Supplementary Figure 41. ^{13}C NMR (100 MHz, $CDCl_3$) spectra for compound **5e**

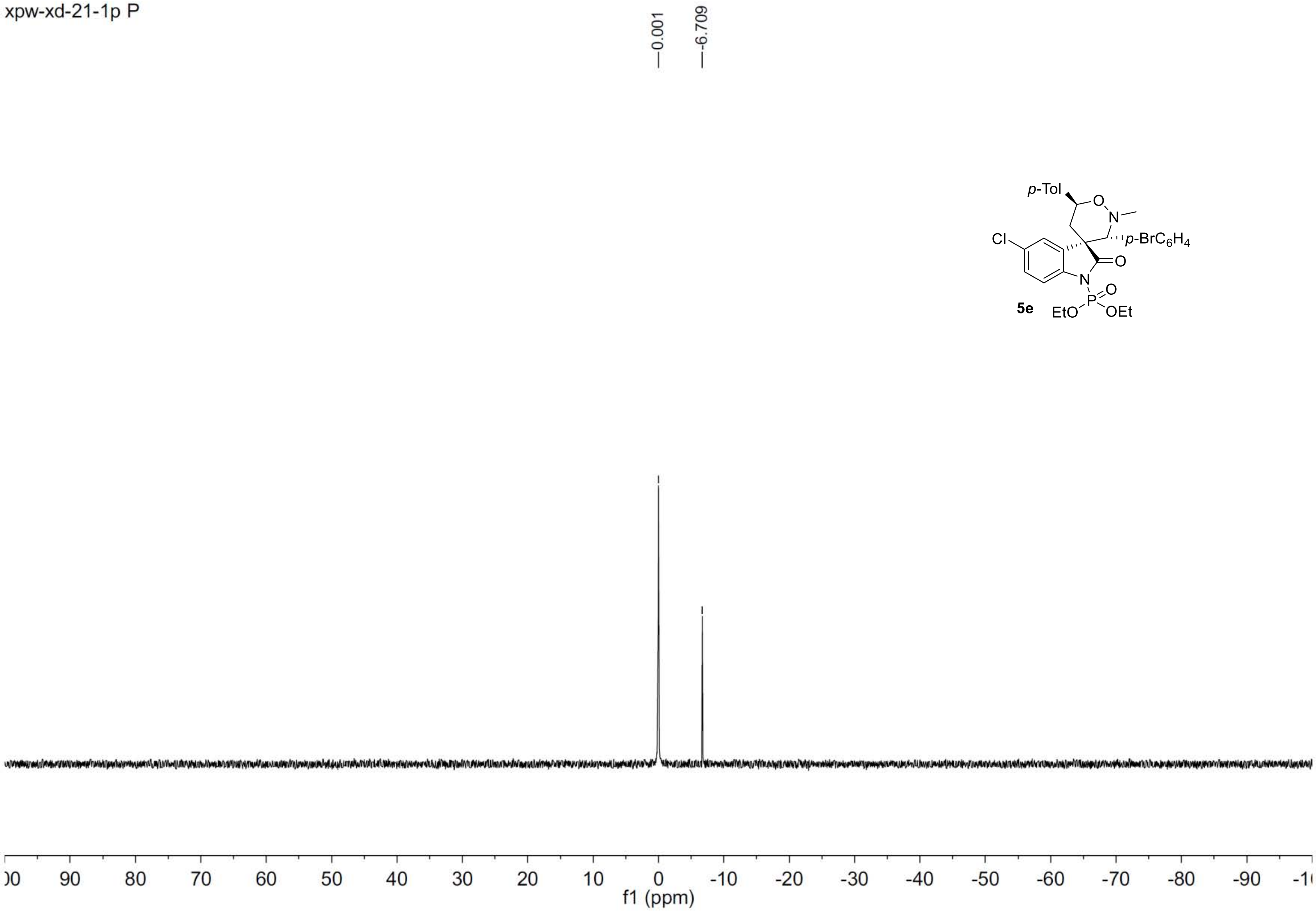

5e

Supplementary Figure 42. ^{31}P NMR (122 MHz, $CDCl_3$) spectra for compound **5e**

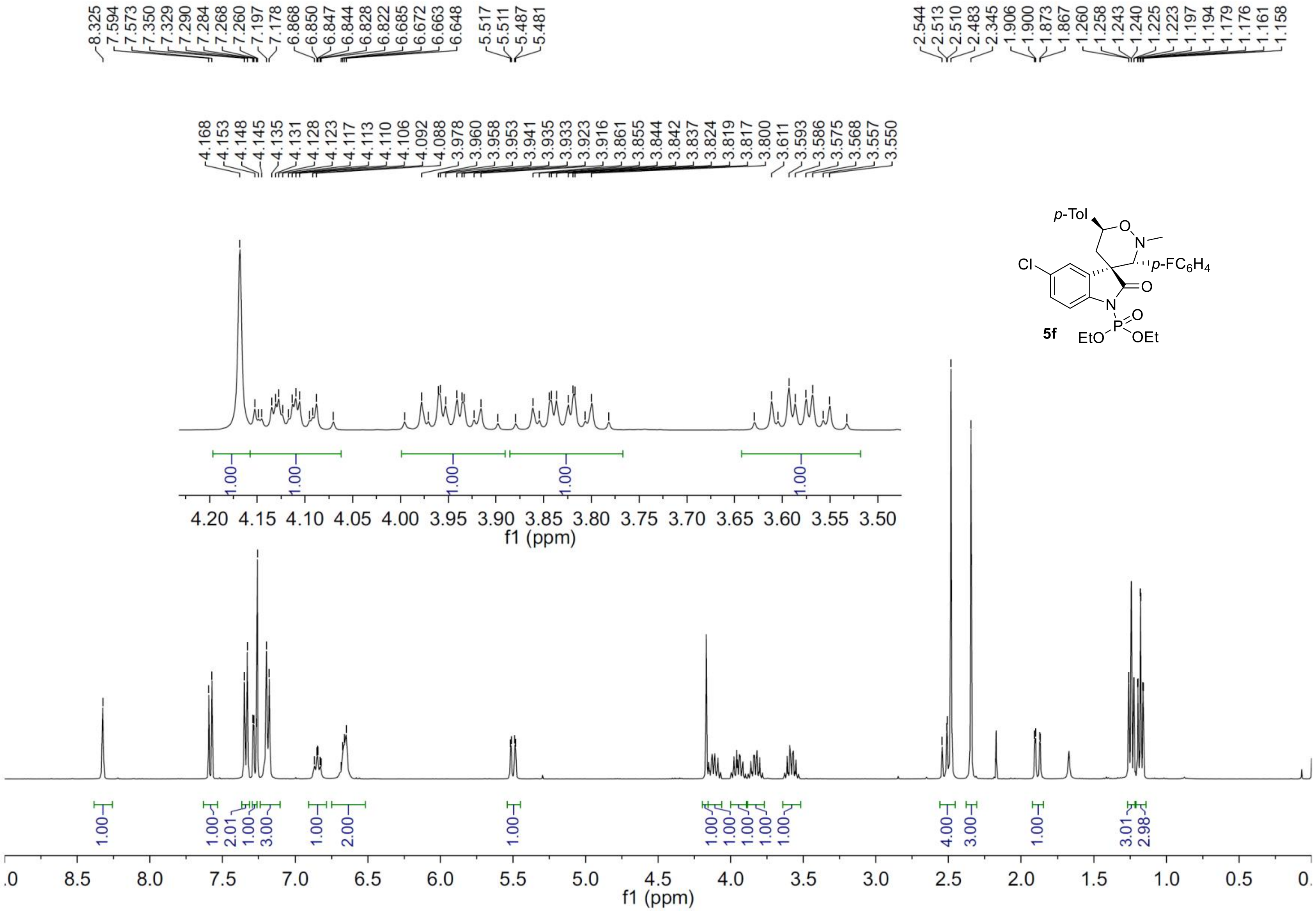

Supplementary Figure 43. ^{1}H NMR (400 MHz, $CDCl_3$) spectra for compound **5f**

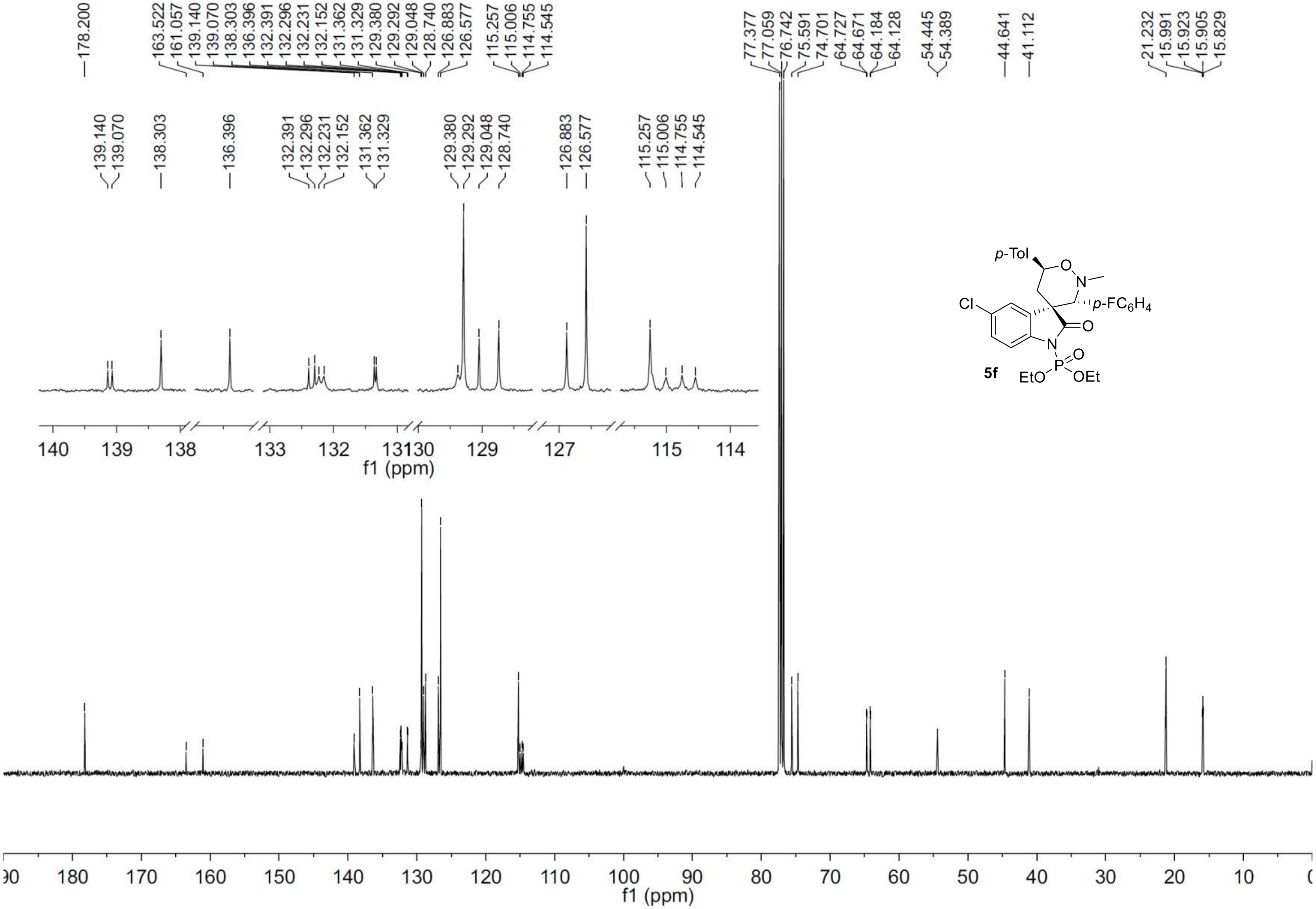

Supplementary Figure 44. ^{13}C NMR (100 MHz, $CDCl_3$) spectra for compound **5f**

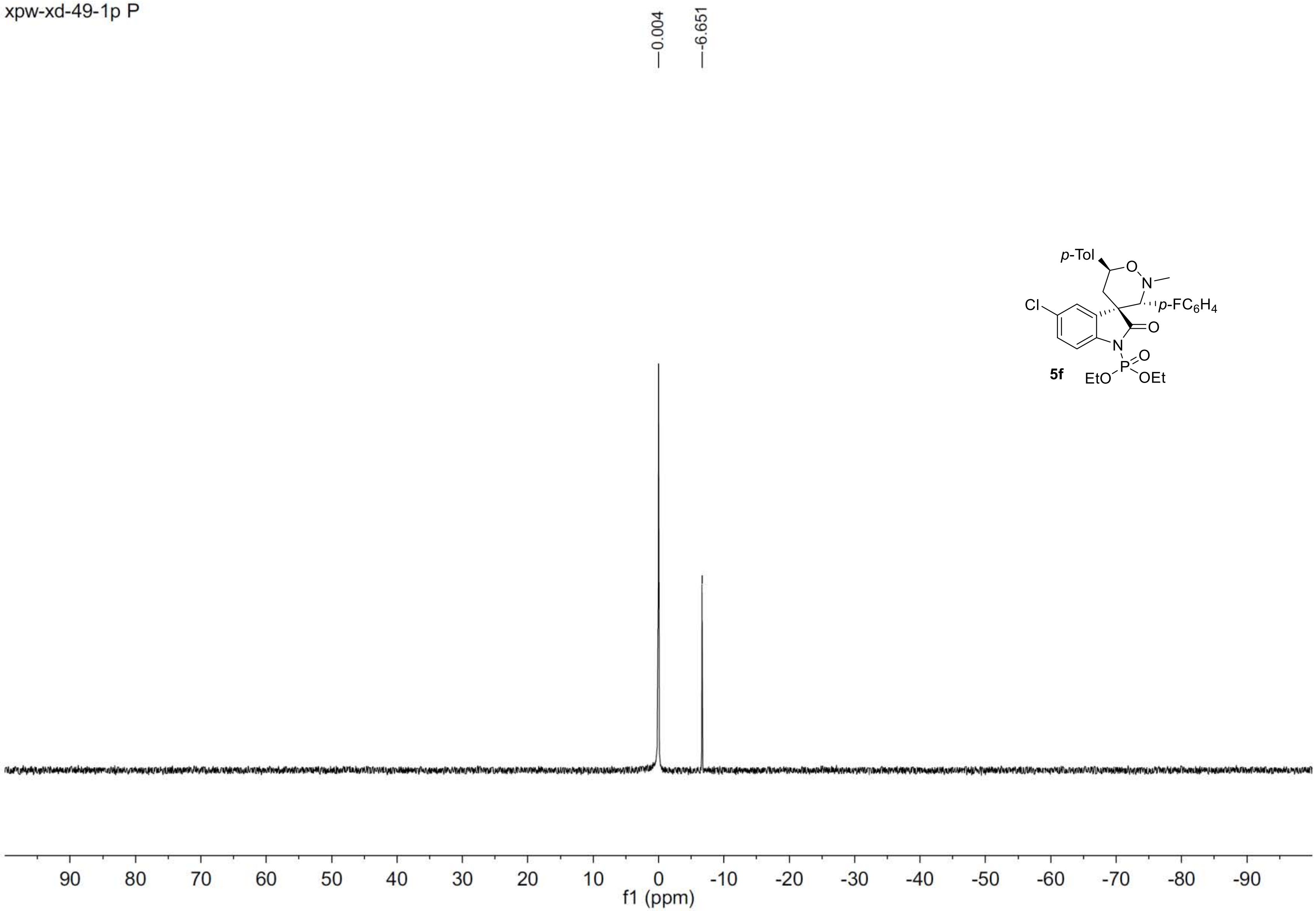

Supplementary Figure 45. ^{31}P NMR (122 MHz, $CDCl_3$) spectra for compound **5f**

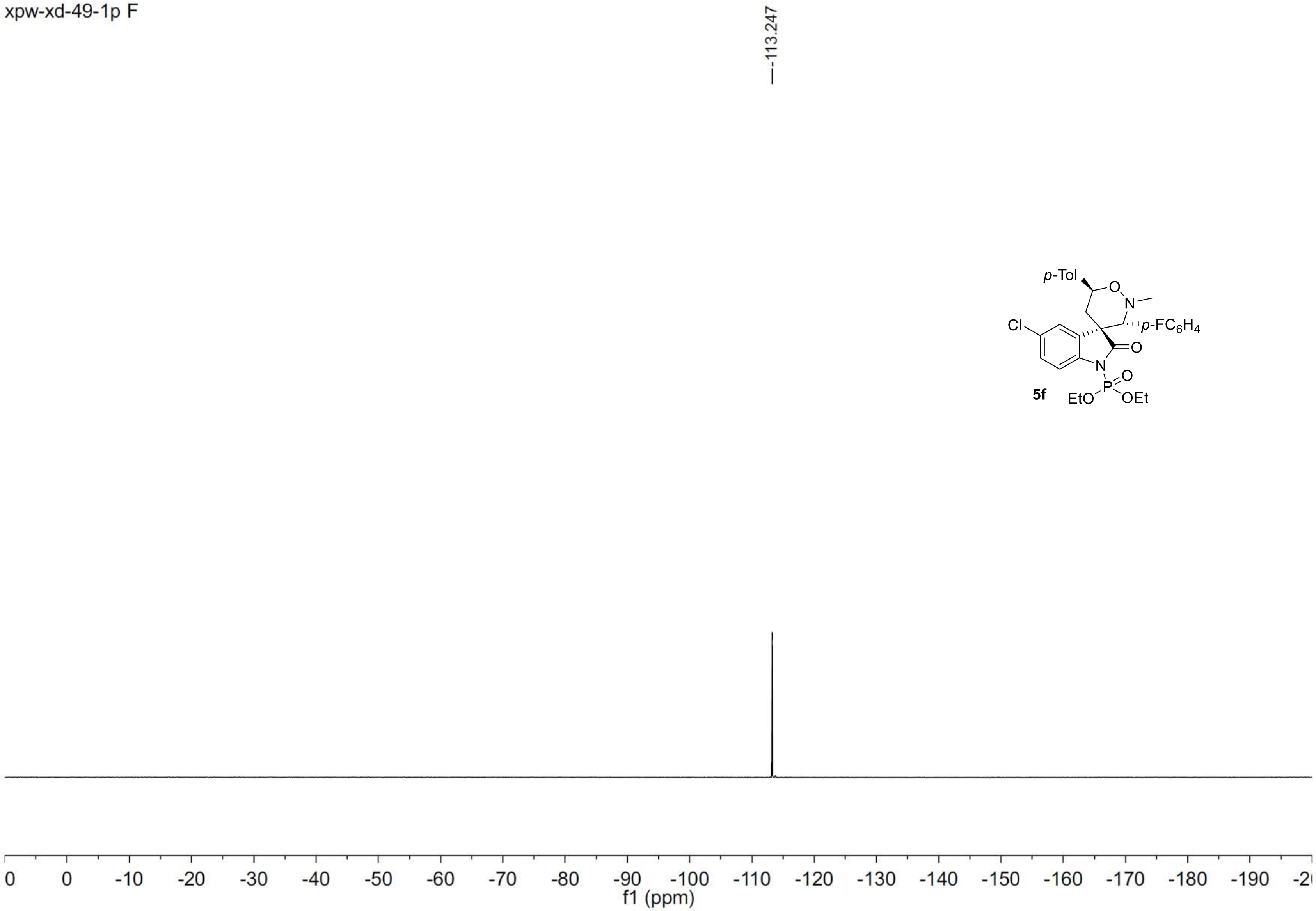

Supplementary Figure 46. ^{19}F NMR (282 MHz, $CDCl_3$) spectra for compound **5f**

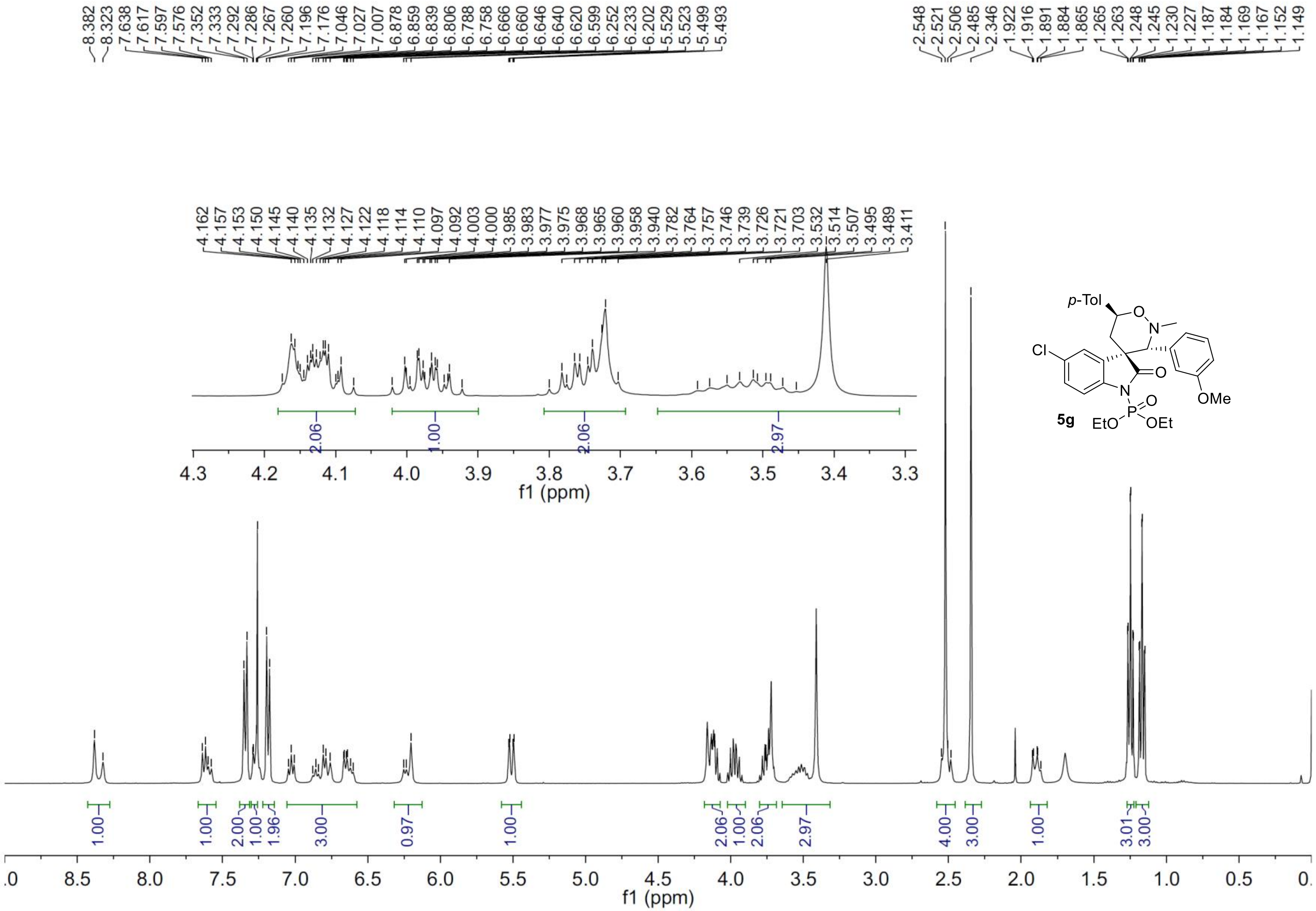

Supplementary Figure 47. ^{1}H NMR (400 MHz, $CDCl_3$) spectra for compound **5g**

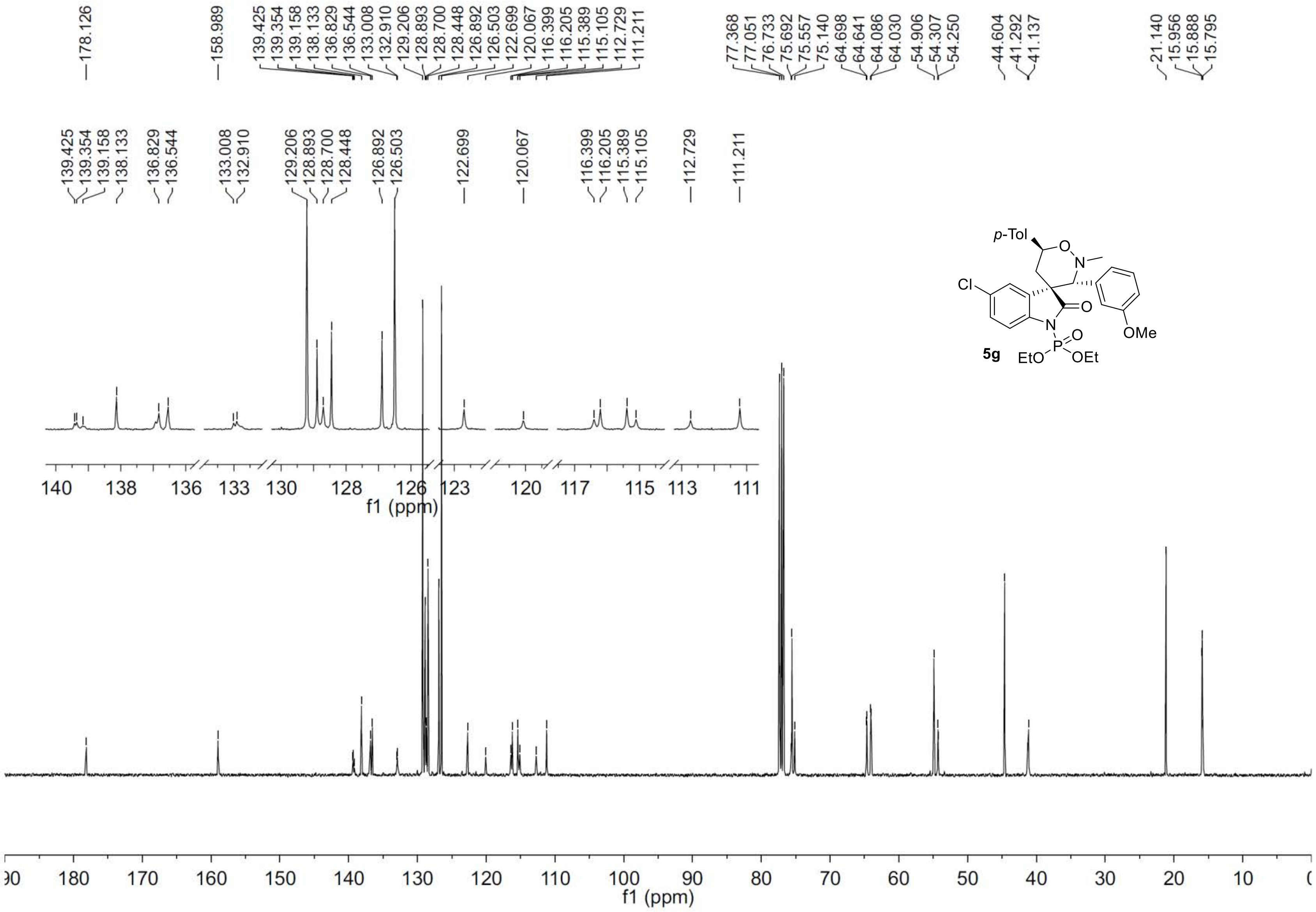

Supplementary Figure 48. ^{13}C NMR (100 MHz, $CDCl_3$) spectra for compound **5g**

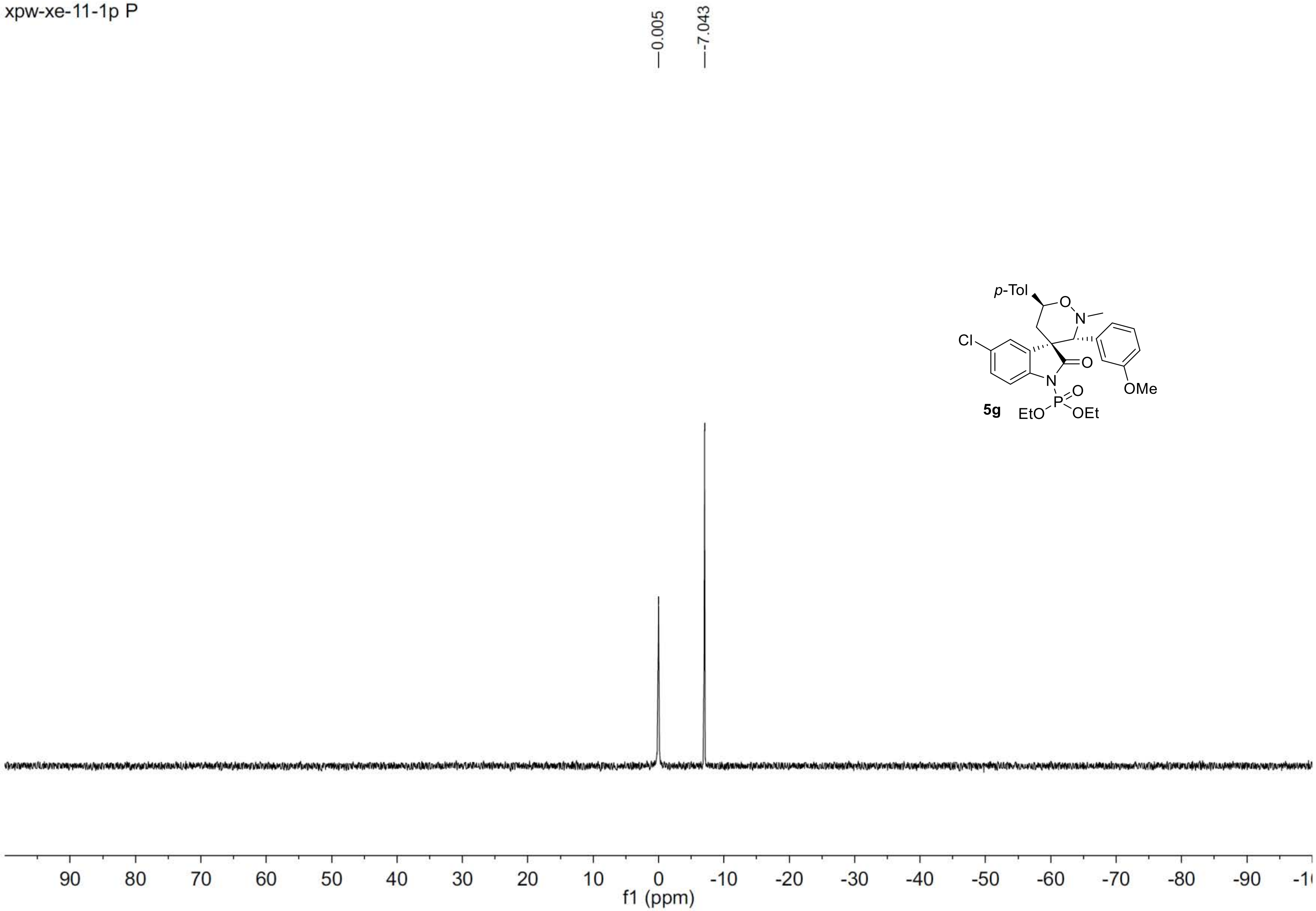

Supplementary Figure 49. ^{31}P NMR (162 MHz, $CDCl_3$) spectra for compound **5g**

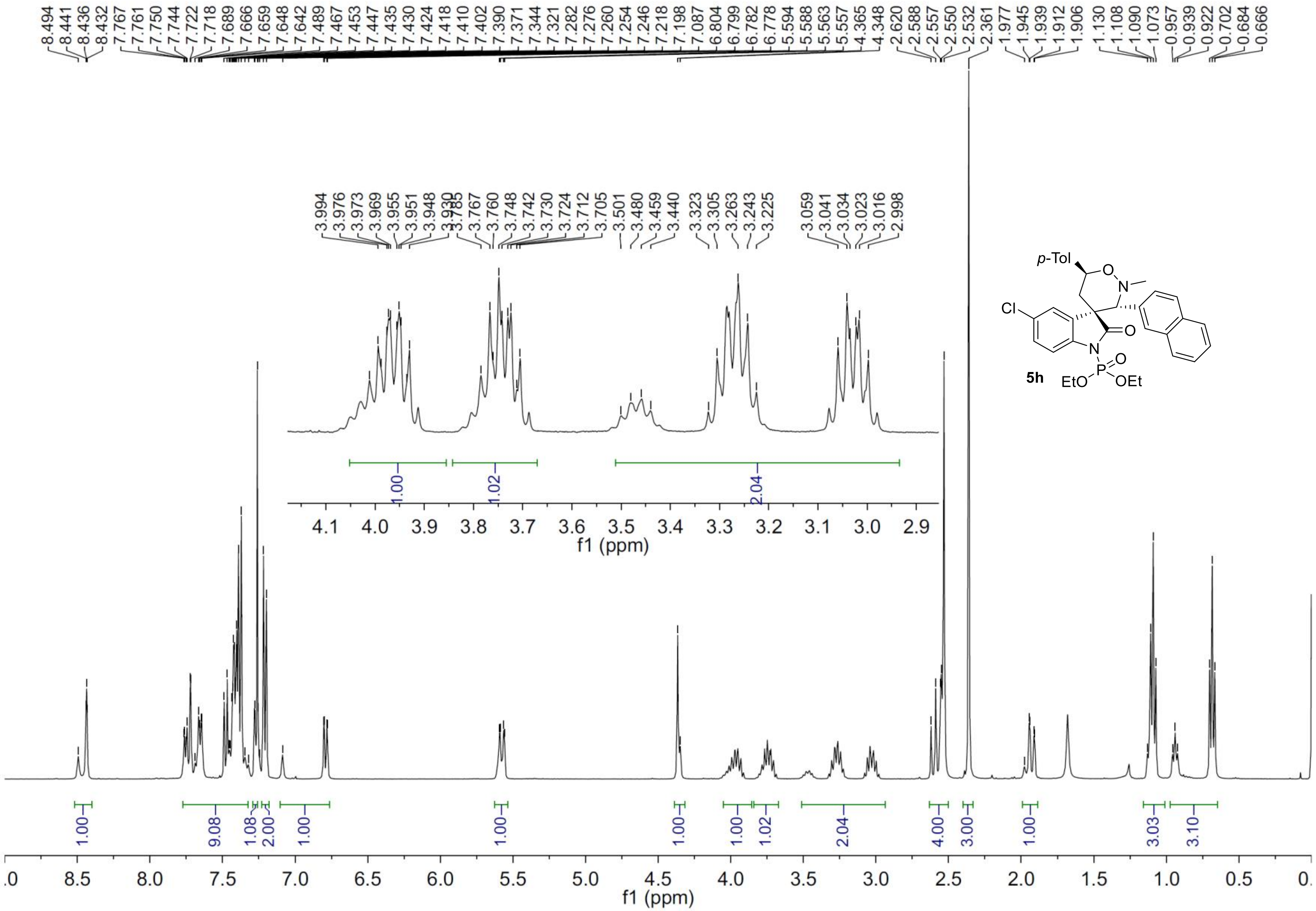

Supplementary Figure 50. ^{1}H NMR (400 MHz, $CDCl_3$) spectra for compound **5h**

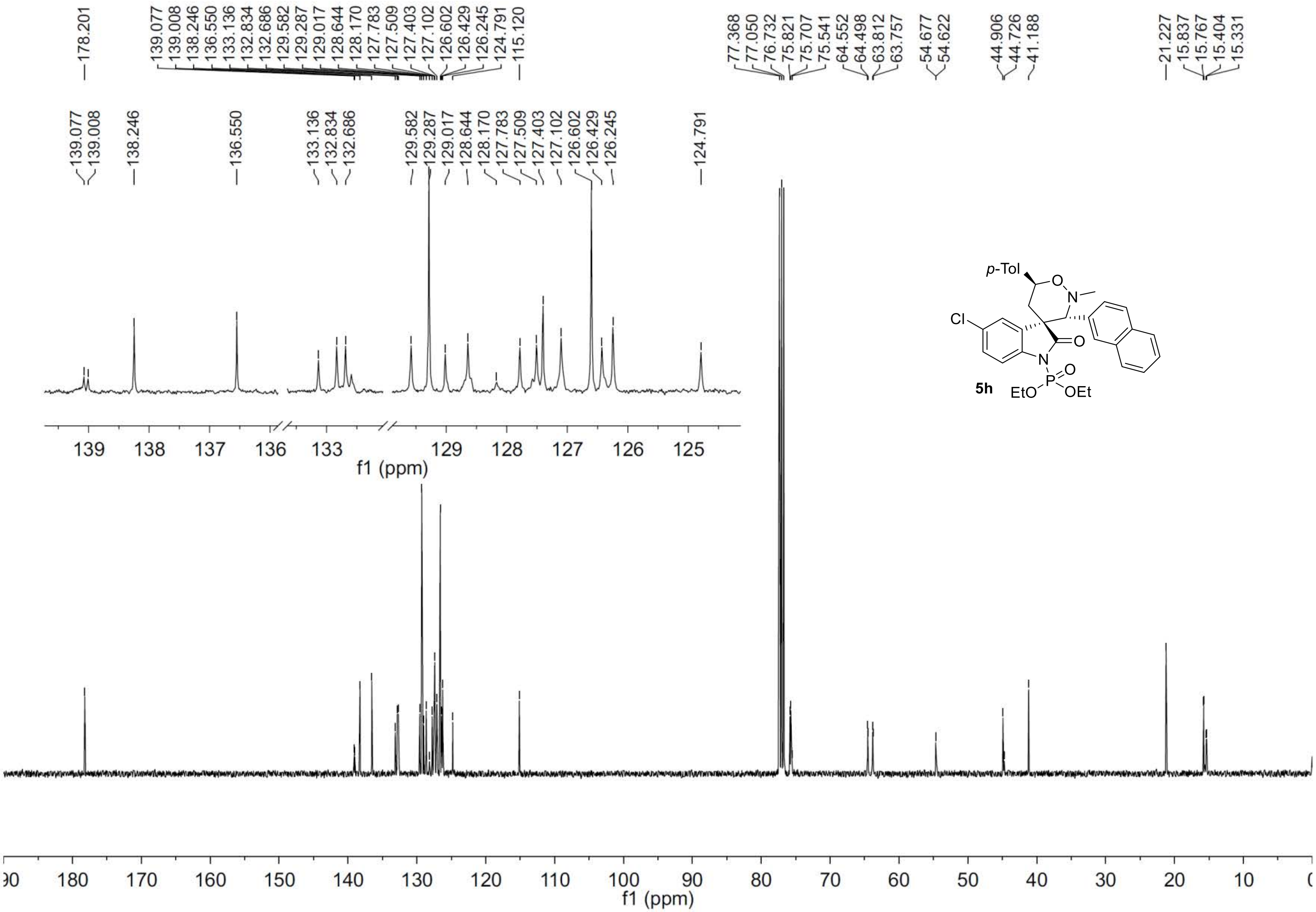

Supplementary Figure 51. ^{13}C NMR (100 MHz, $CDCl_3$) spectra for compound **5h**

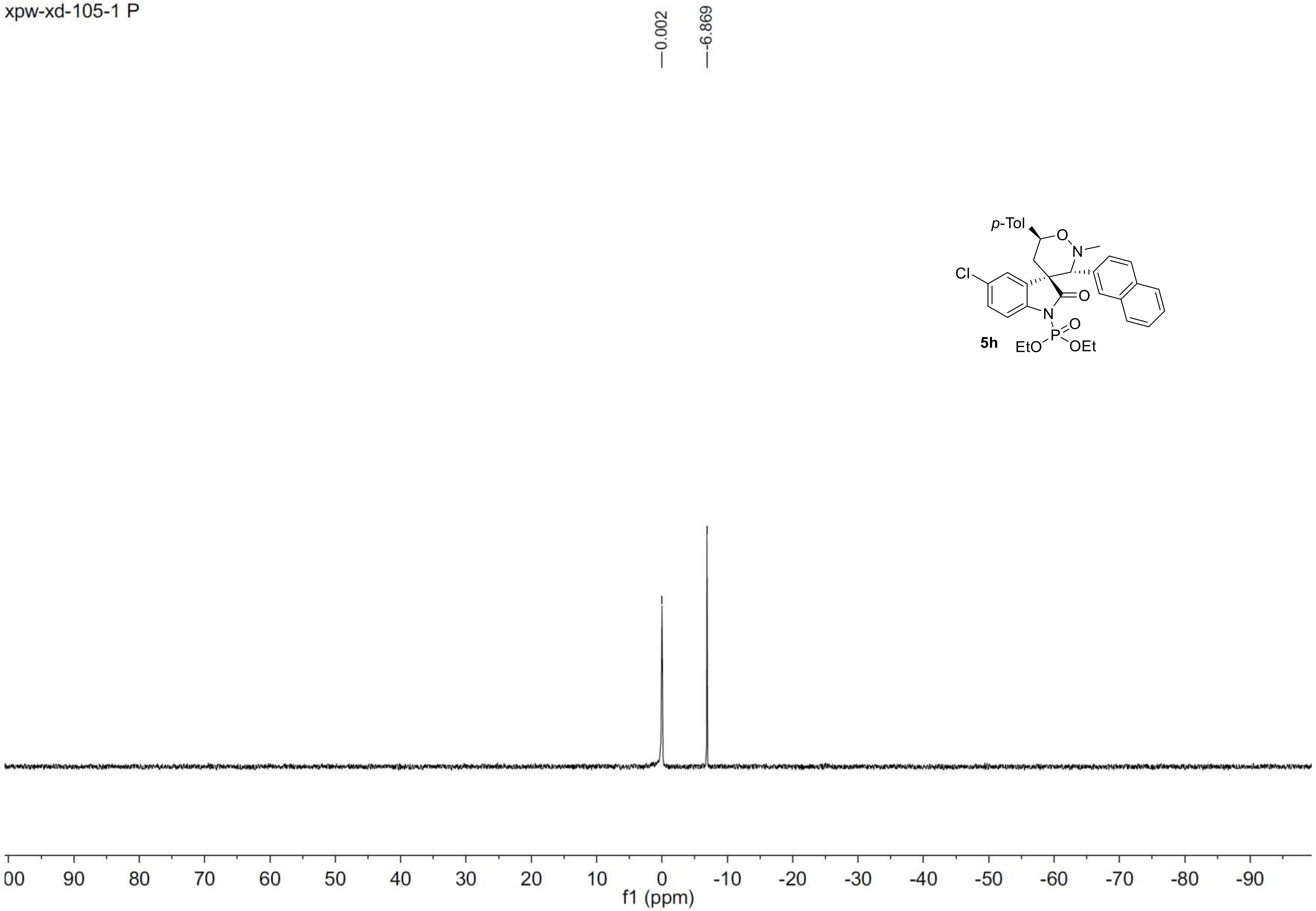

5h

Supplementary Figure 52. ^{31}P NMR (122 MHz, $CDCl_3$) spectra for compound **5h**

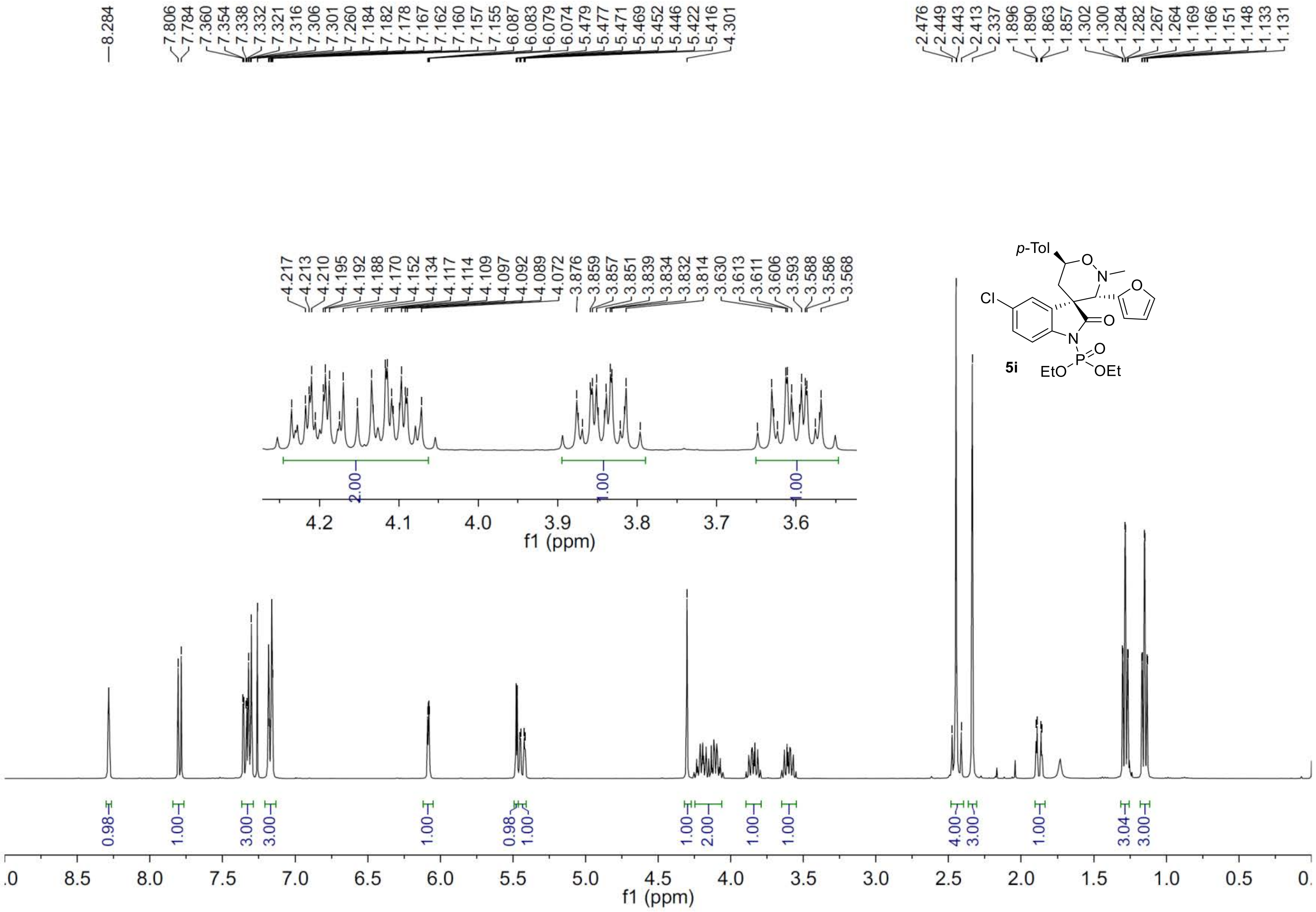

Supplementary Figure 53. ^{1}H NMR (400 MHz, $CDCl_3$) spectra for compound **5i**

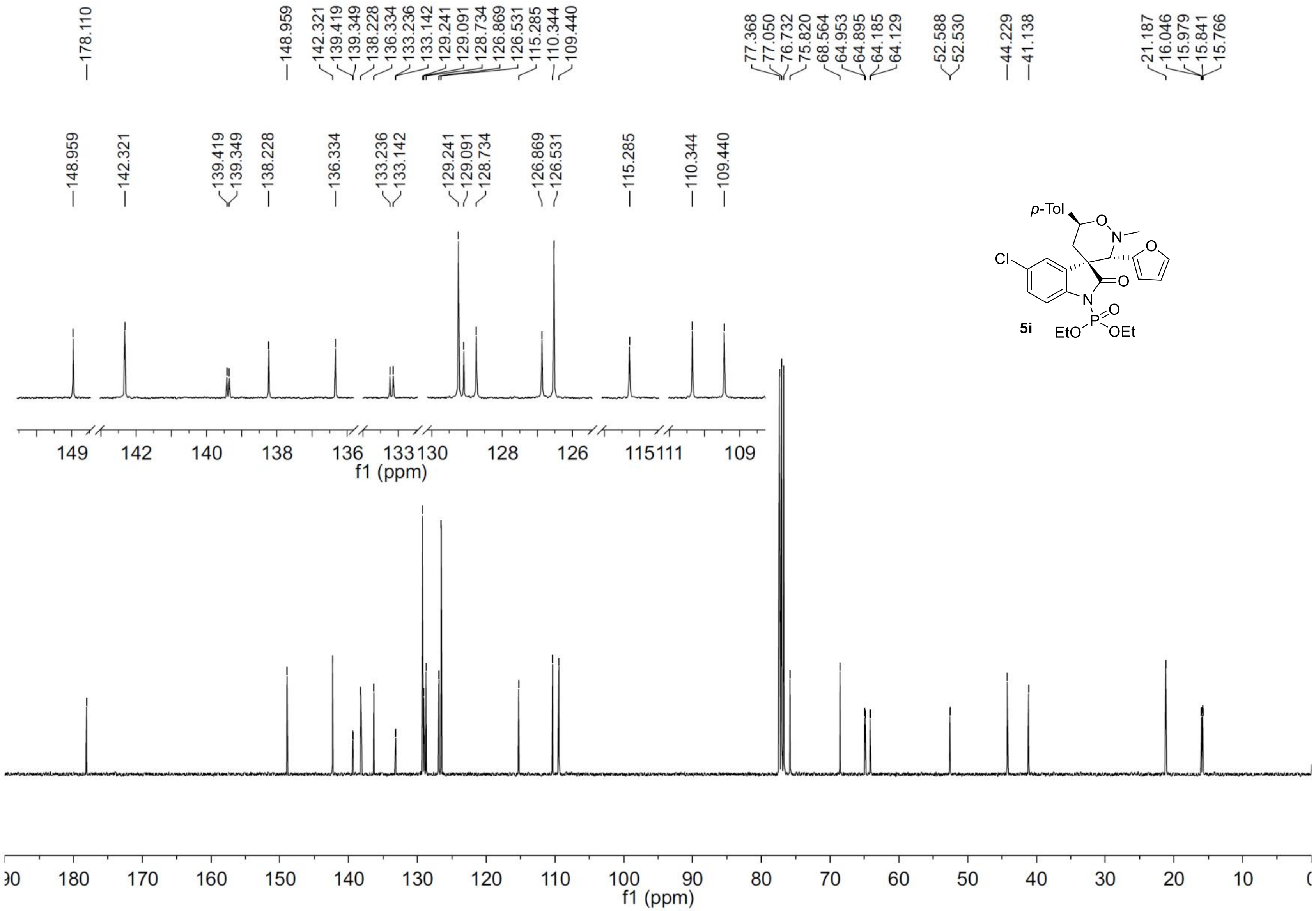

Supplementary Figure 54. ^{13}C NMR (100 MHz, $CDCl_3$) spectra for compound **5i**

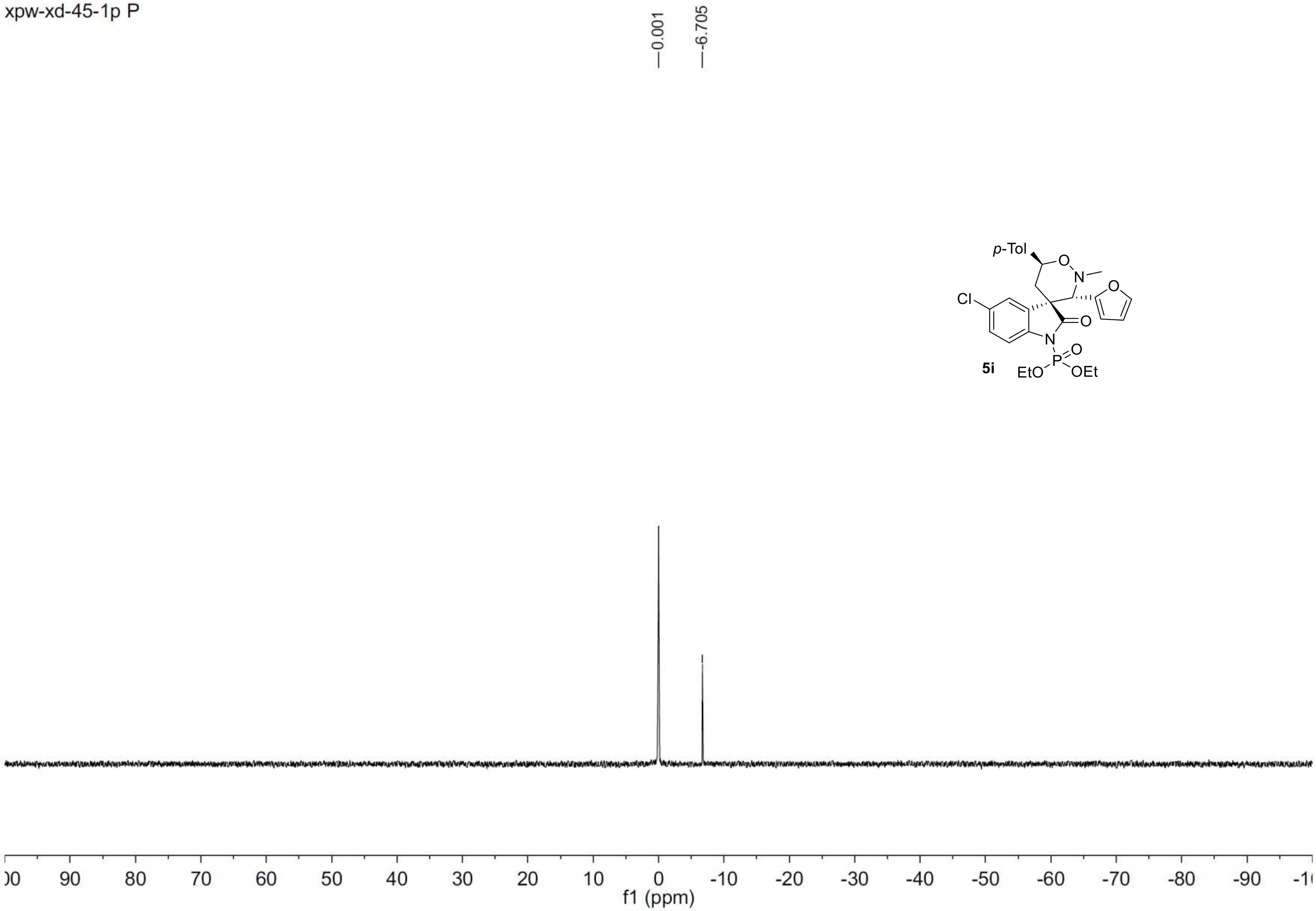

Supplementary Figure 55. ^{31}P NMR (122 MHz, $CDCl_3$) spectra for compound **5i**

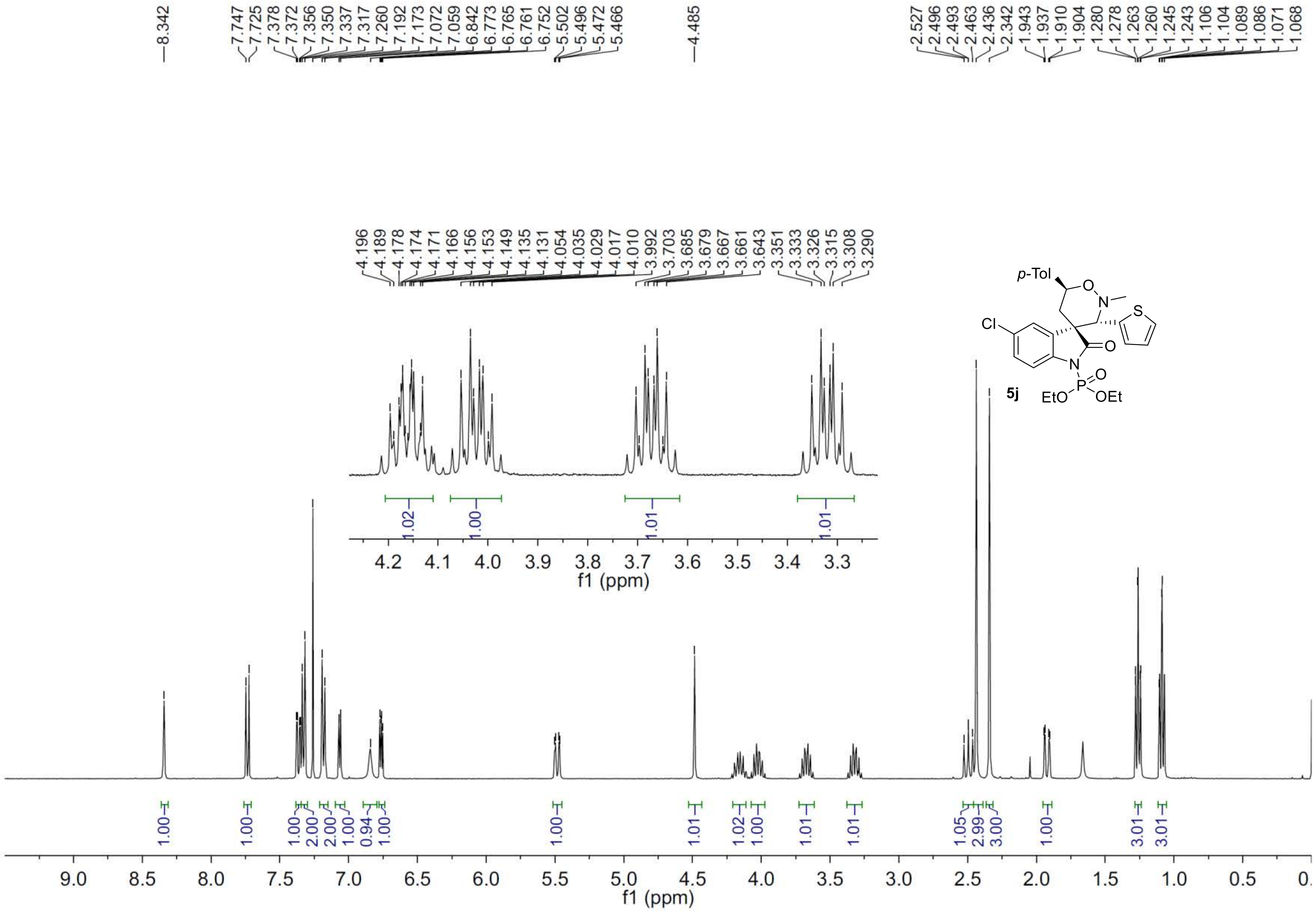

Supplementary Figure 56. ^{1}H NMR (400 MHz, $CDCl_3$) spectra for compound **5j**

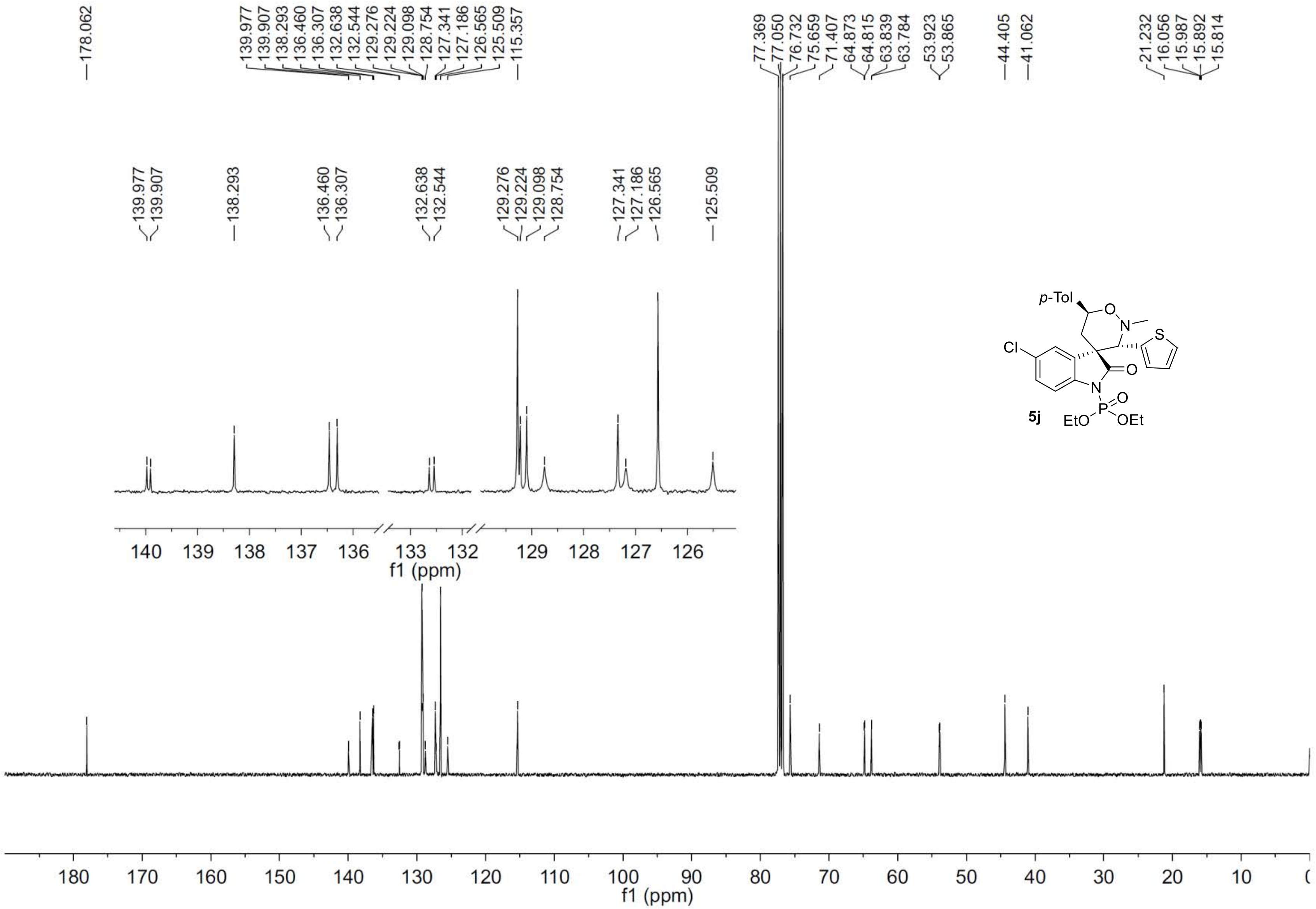

Supplementary Figure 57. ^{13}C NMR (100 MHz, $CDCl_3$) spectra for compound **5j**

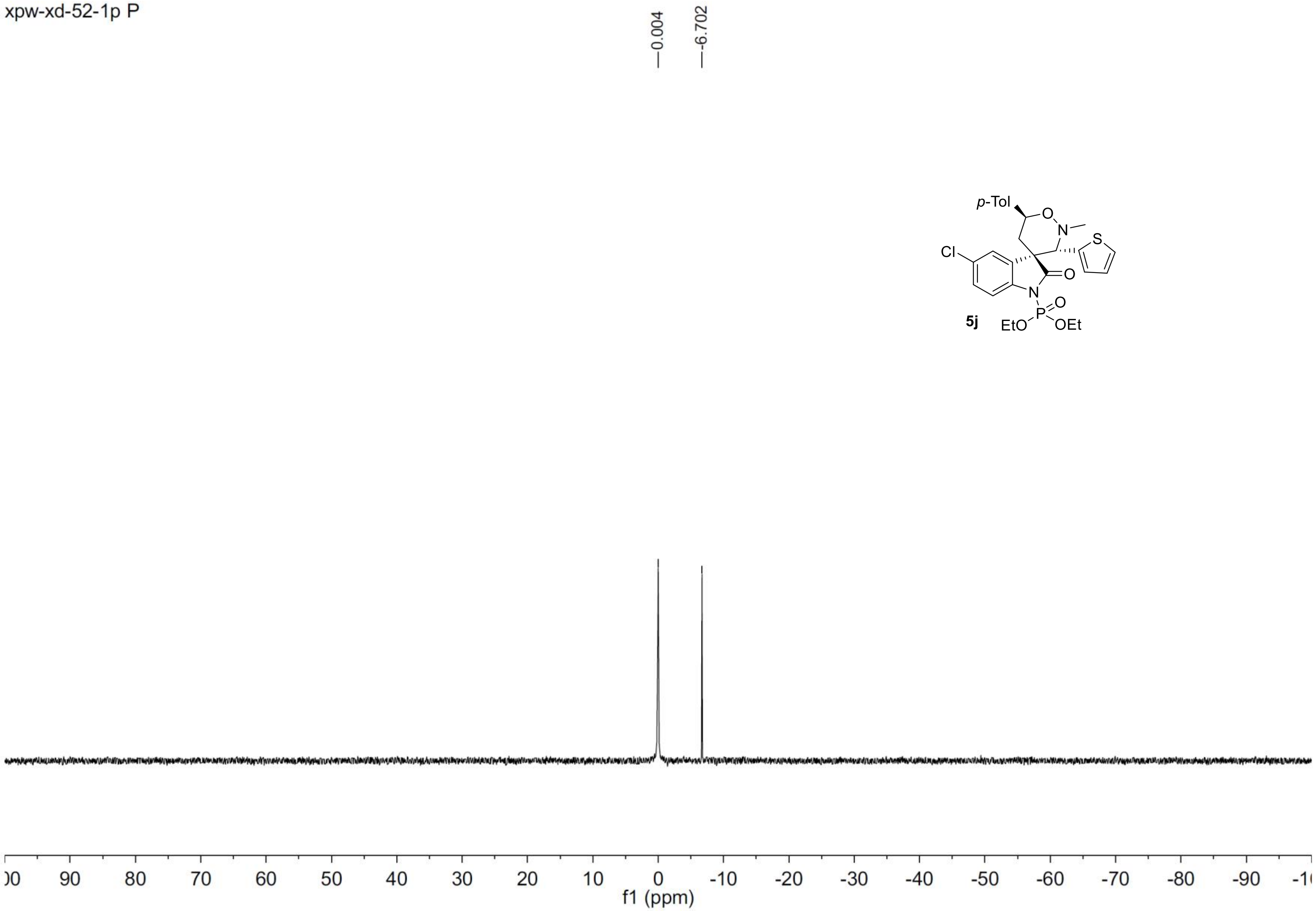

Supplementary Figure 58. ^{31}P NMR (122 MHz, $CDCl_3$) spectra for compound **5j**

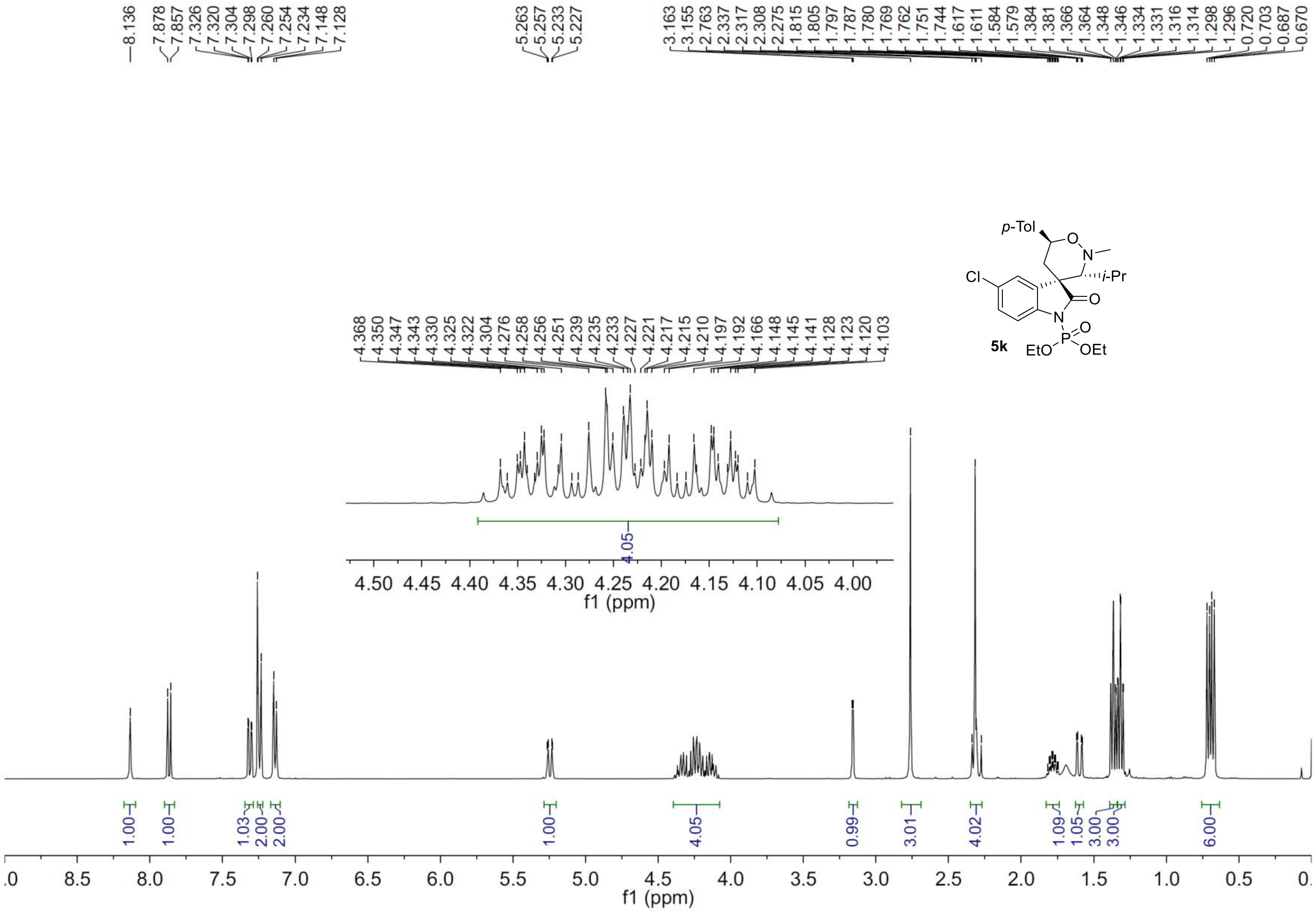

Supplementary Figure 59. ^{1}H NMR (400 MHz, $CDCl_3$) spectra for compound **5k**

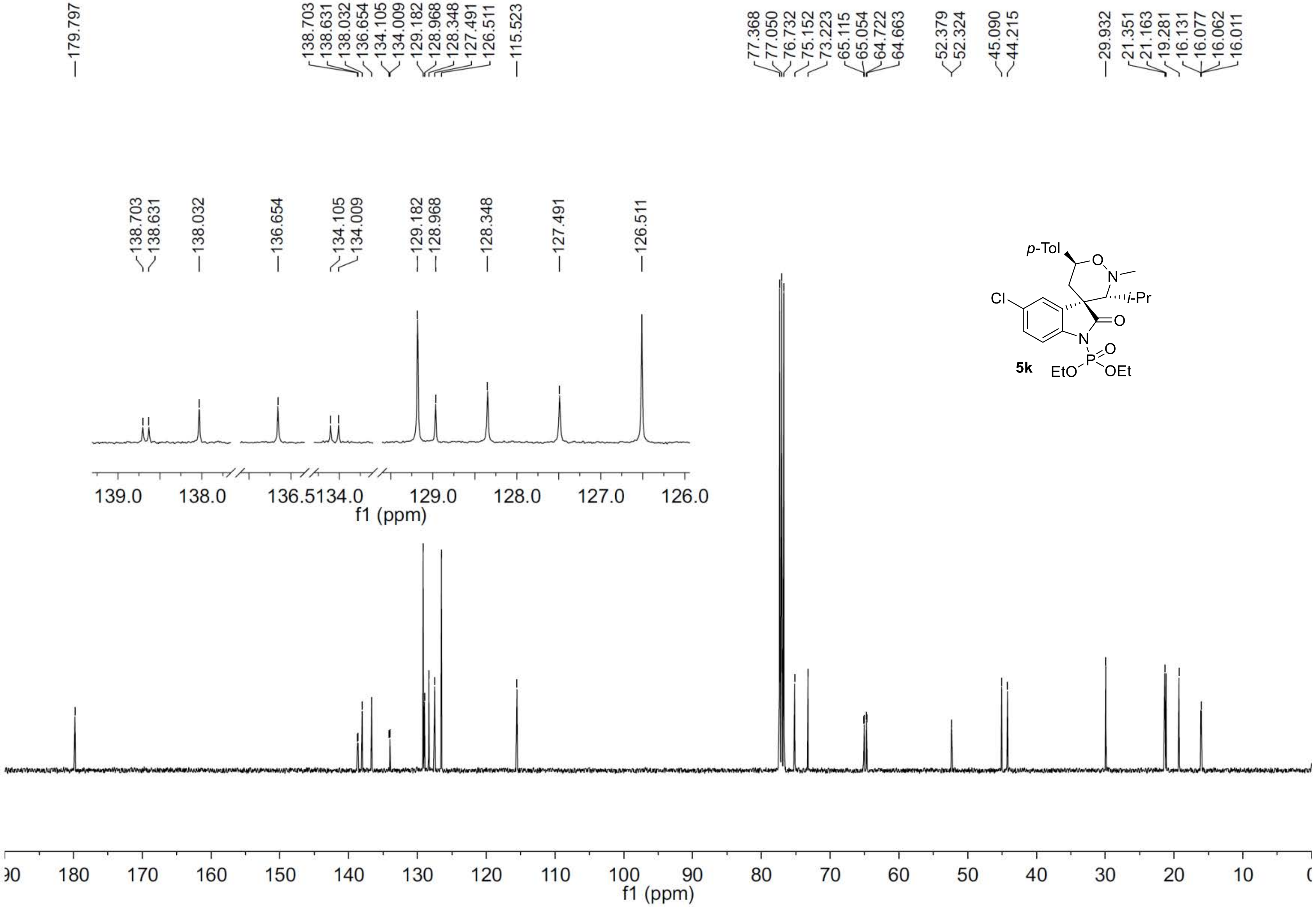

Supplementary Figure 60. ^{13}C NMR (100 MHz, $CDCl_3$) spectra for compound **5k**

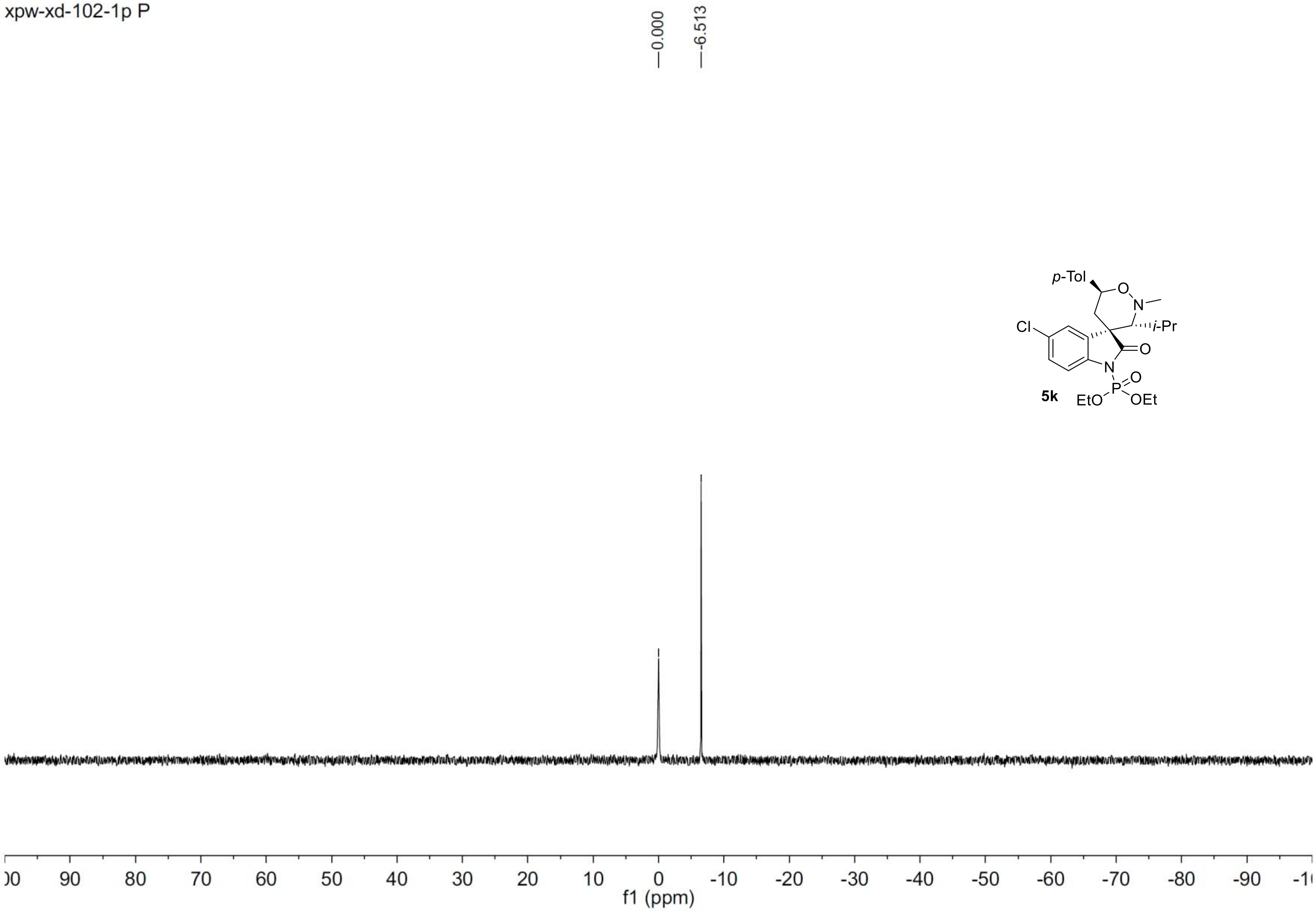

Supplementary Figure 61. ^{31}P NMR (122 MHz, $CDCl_3$) spectra for compound **5k**

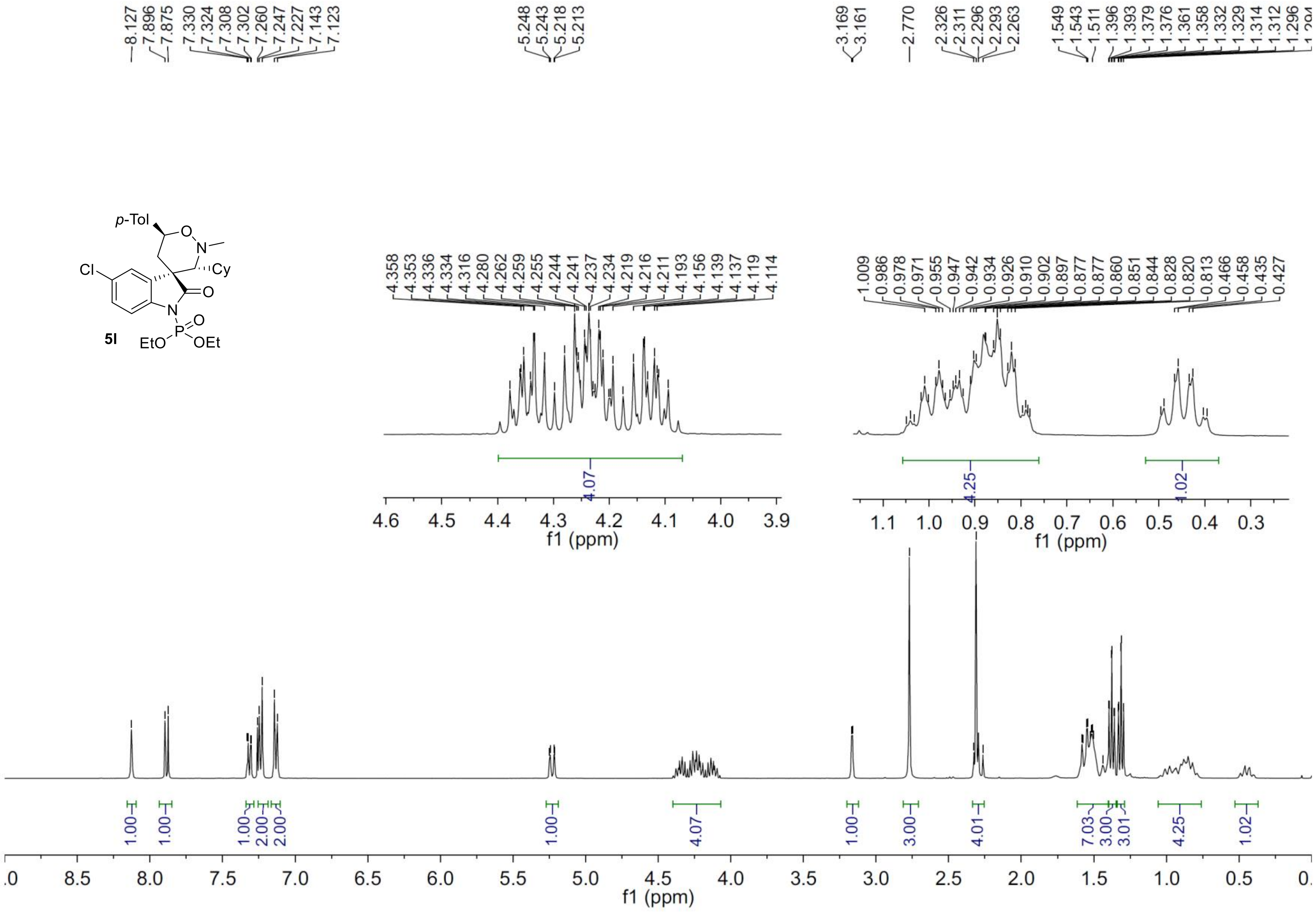

Supplementary Figure 62. ^{1}H NMR (400 MHz, $CDCl_3$) spectra for compound **5l**

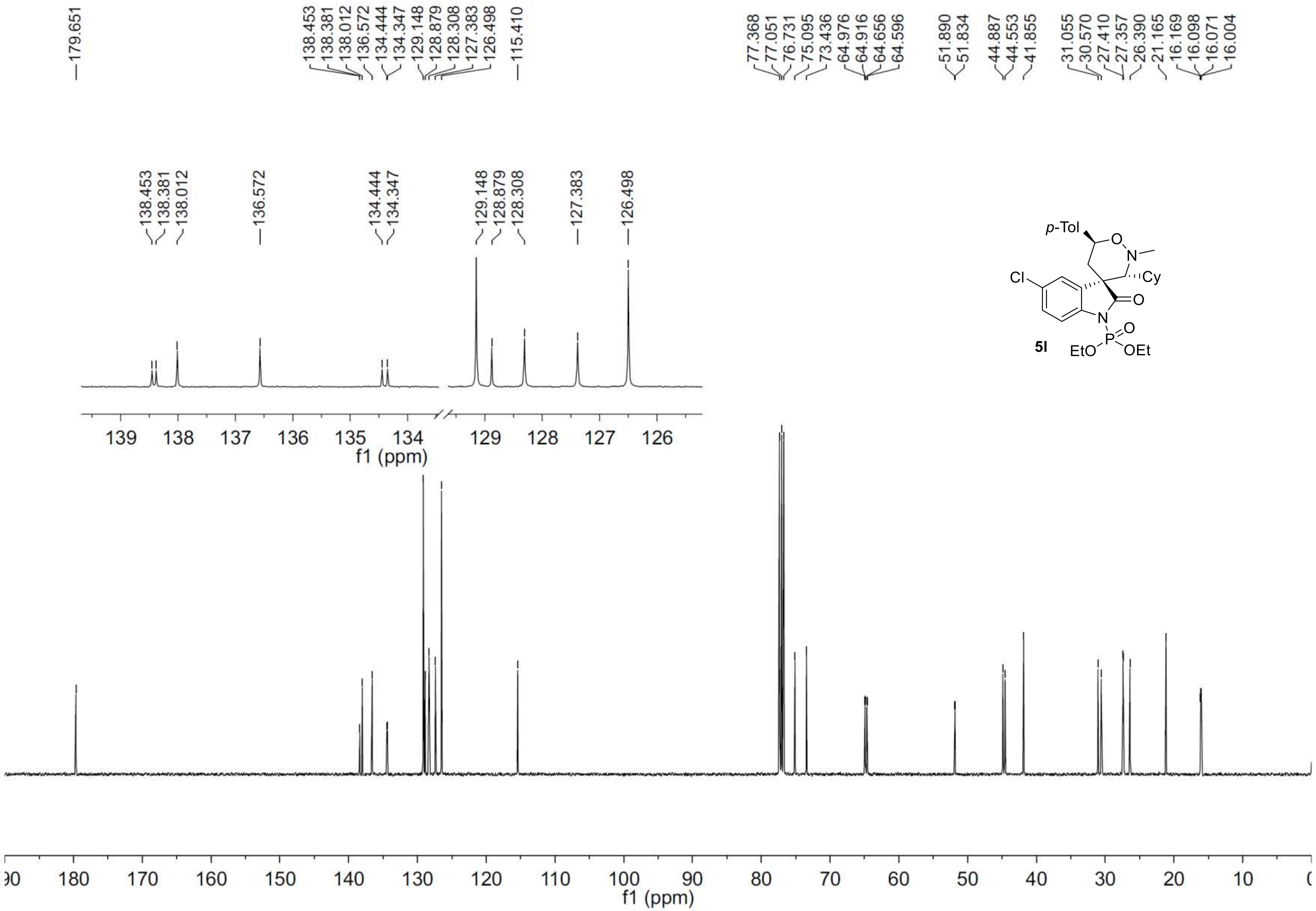

Supplementary Figure 63. ^{13}C NMR (100 MHz, $CDCl_3$) spectra for compound **5l**

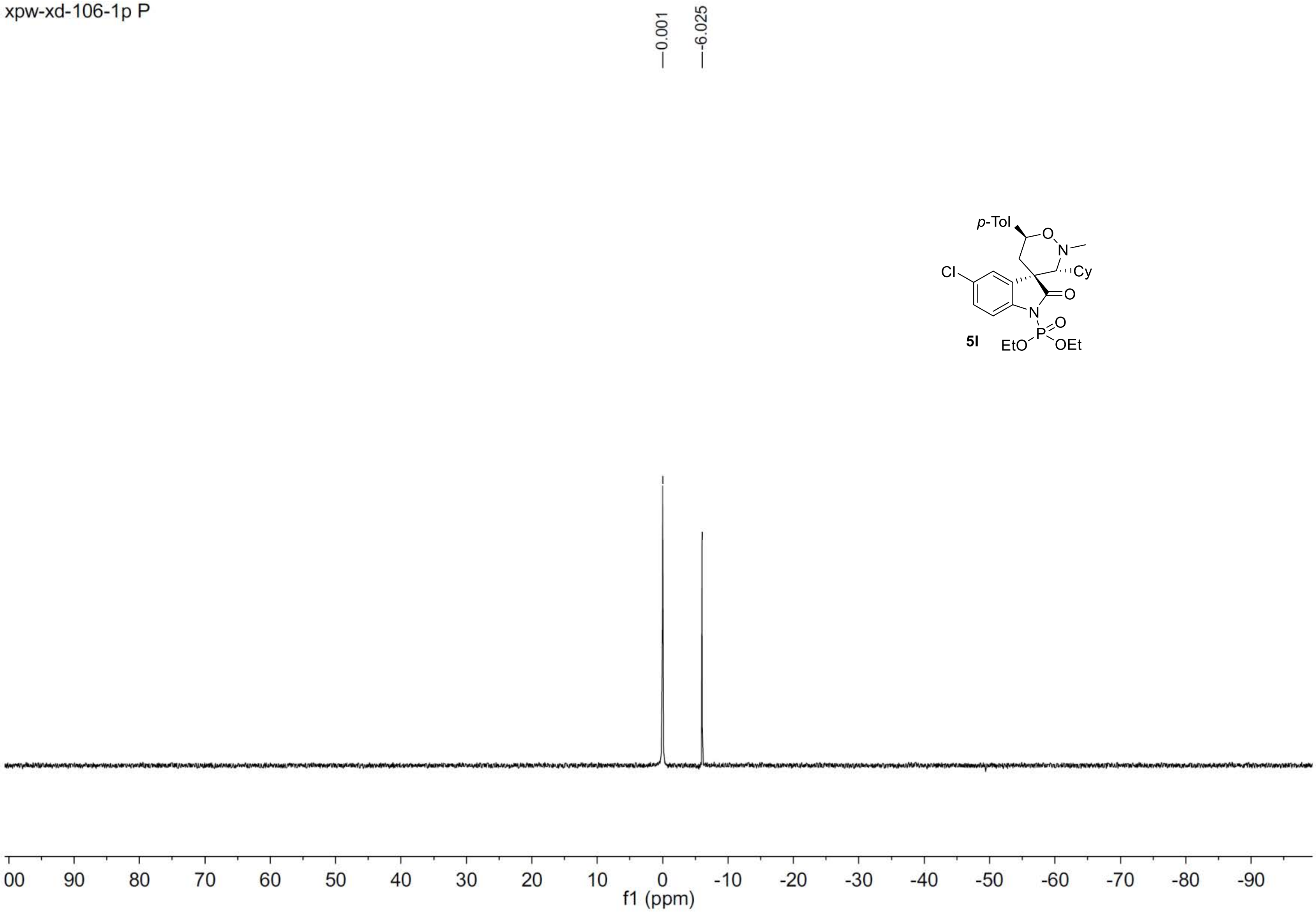

5l

Supplementary Figure 64. ^{31}P NMR (122 MHz, $CDCl_3$) spectra for compound **5l**

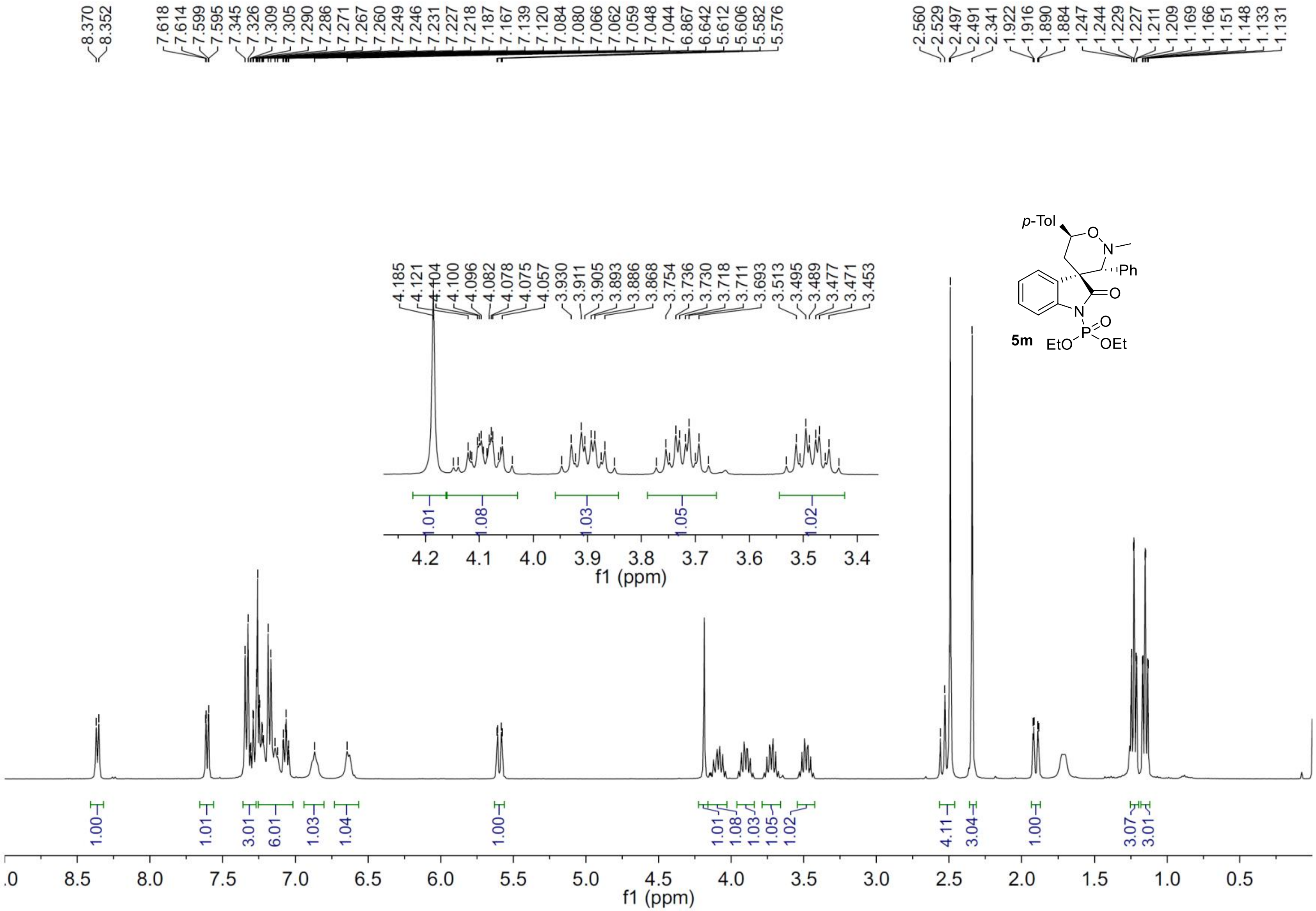

Supplementary Figure 65. ^{1}H NMR (400 MHz, $CDCl_3$) spectra for compound **5m**

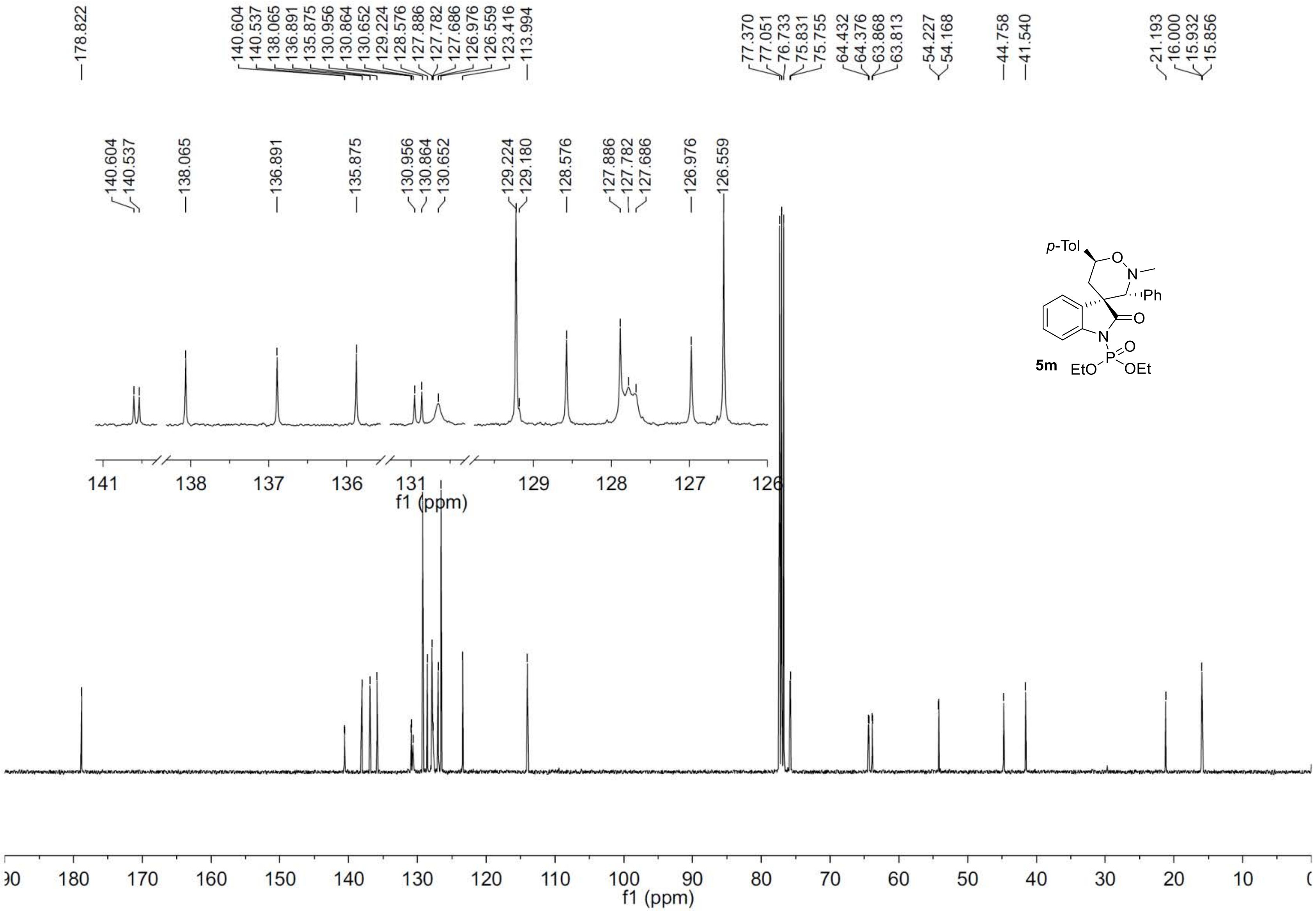

Supplementary Figure 66. ^{13}C NMR (100 MHz, $CDCl_3$) spectra for compound **5m**

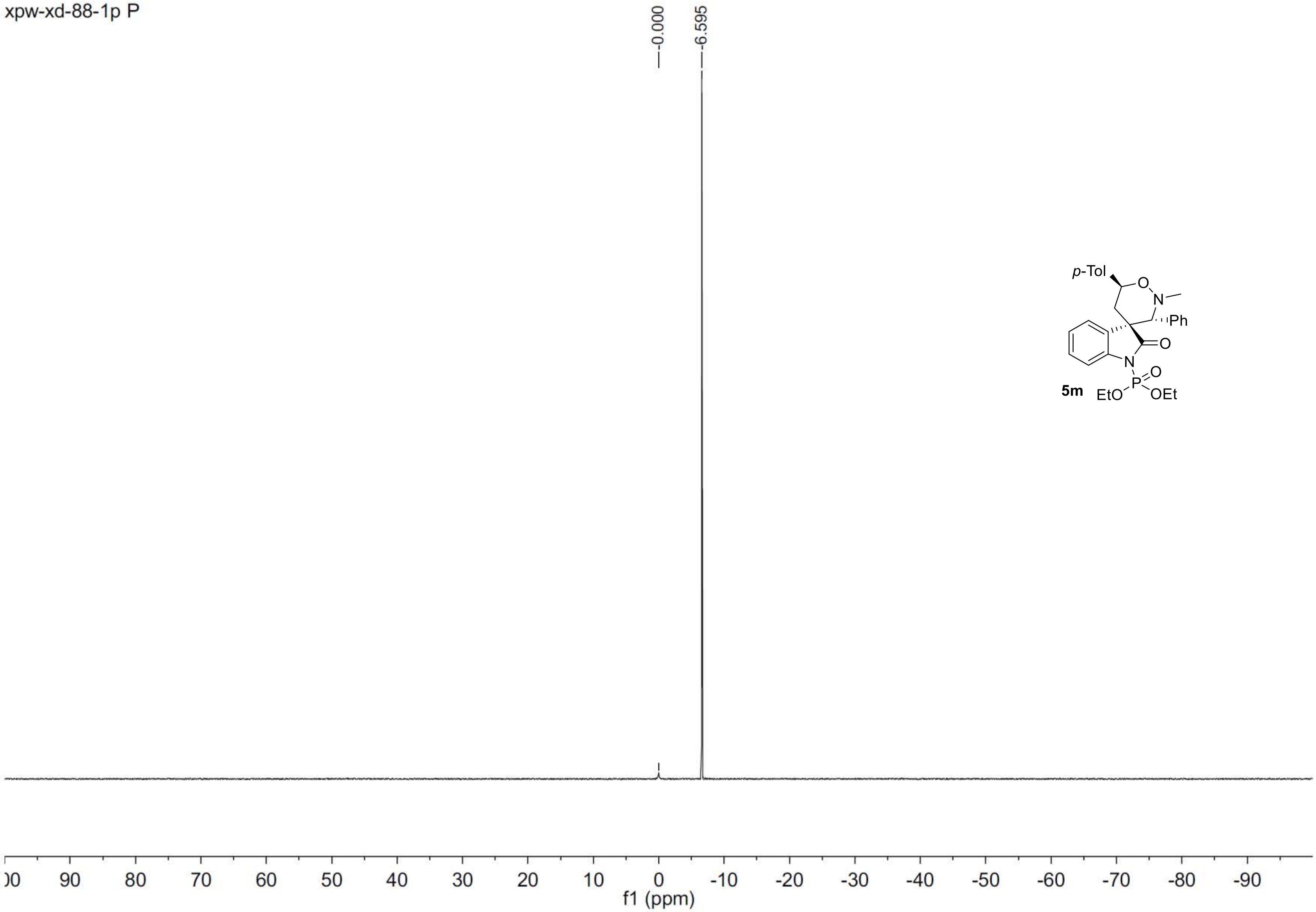

Supplementary Figure 67. ^{31}P NMR (122 MHz, $CDCl_3$) spectra for compound **5m**

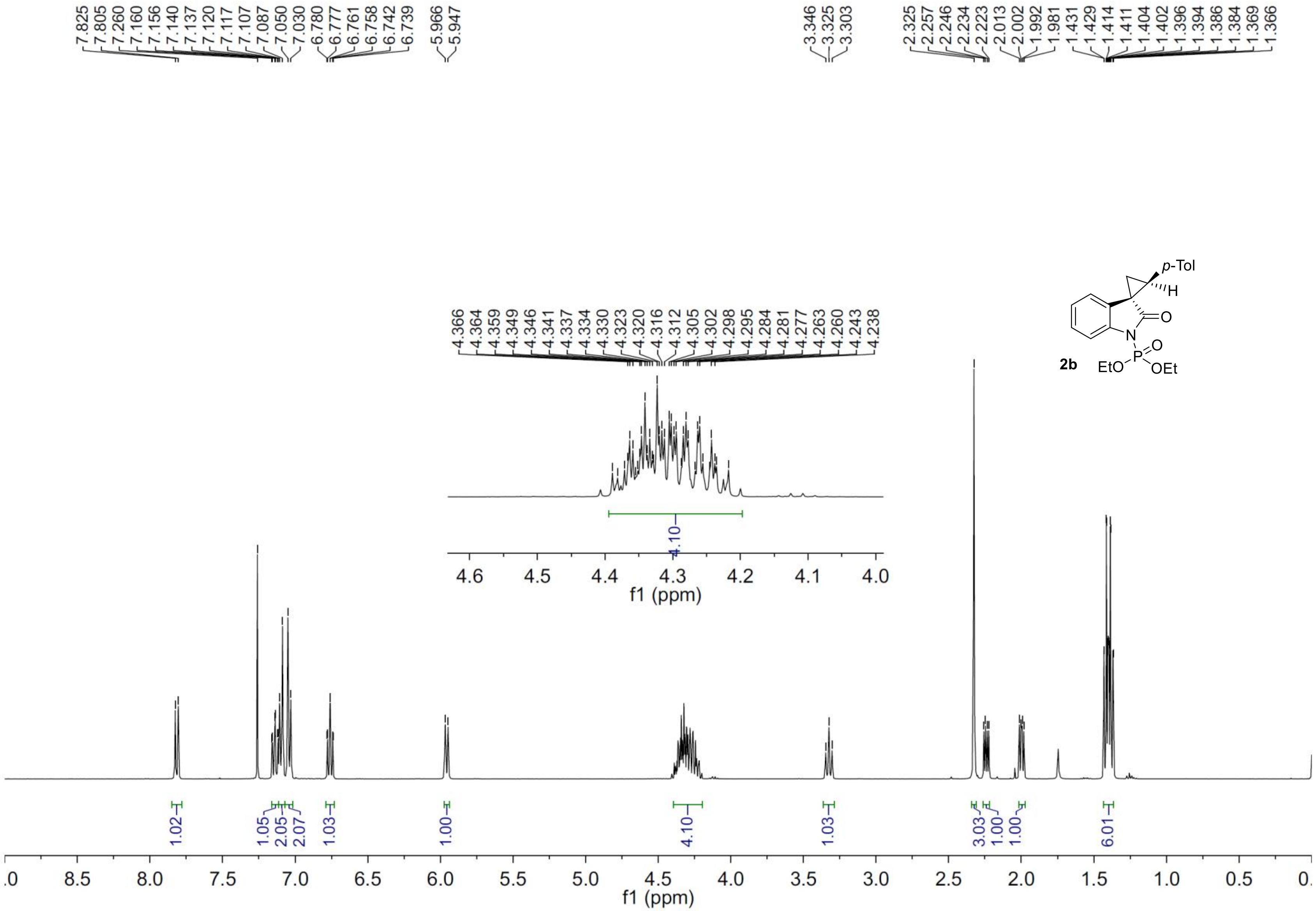

Supplementary Figure 68. ^{1}H NMR (400 MHz, $CDCl_3$) spectra for compound **2b**

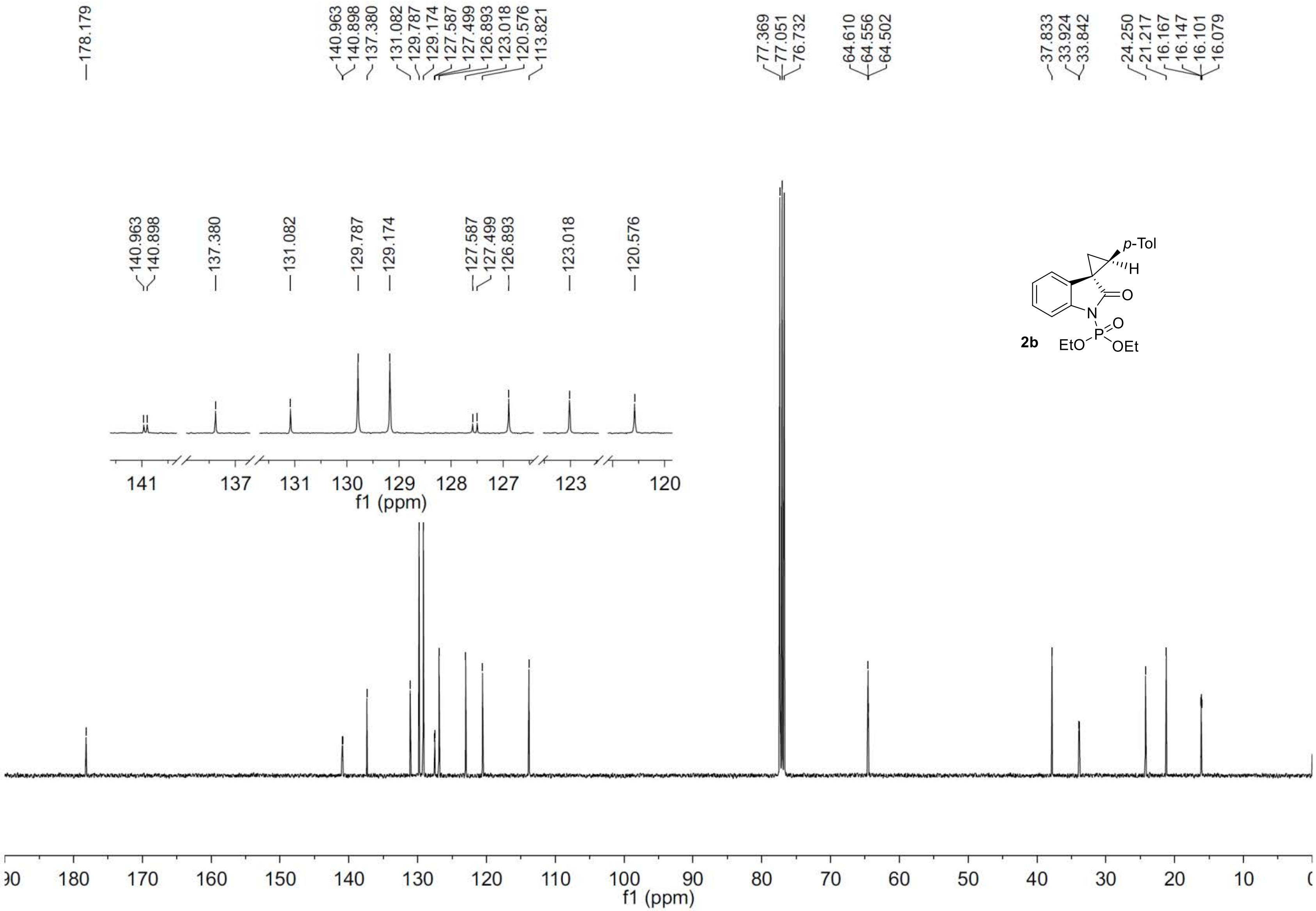

Supplementary Figure 69. ^{13}C NMR (100 MHz, $CDCl_3$) spectra for compound **2b**

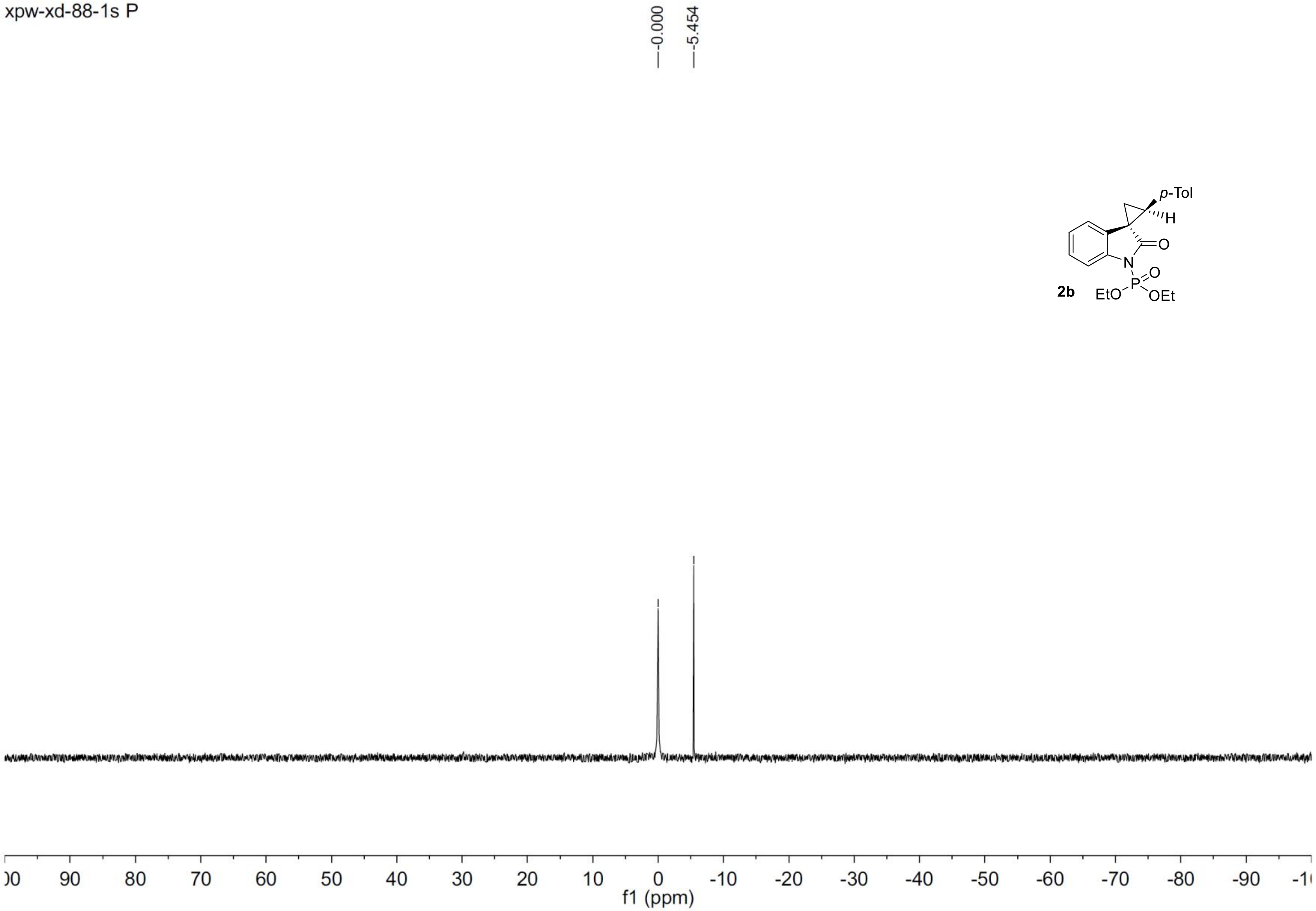

2b

Supplementary Figure 70. ^{31}P NMR (122 MHz, $CDCl_3$) spectra for compound **2b**

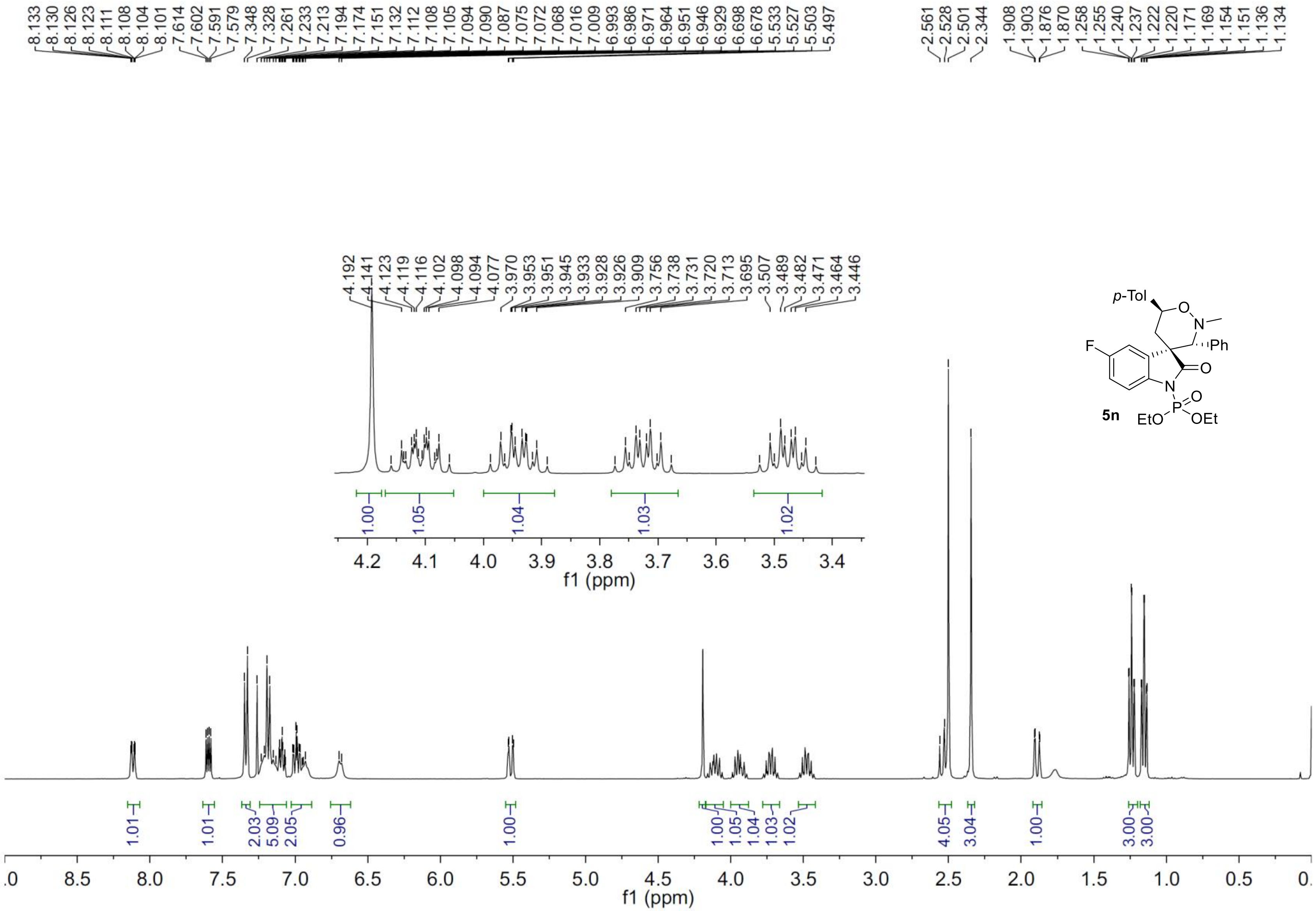

Supplementary Figure 71. ^{1}H NMR (400 MHz, $CDCl_3$) spectra for compound **5n**

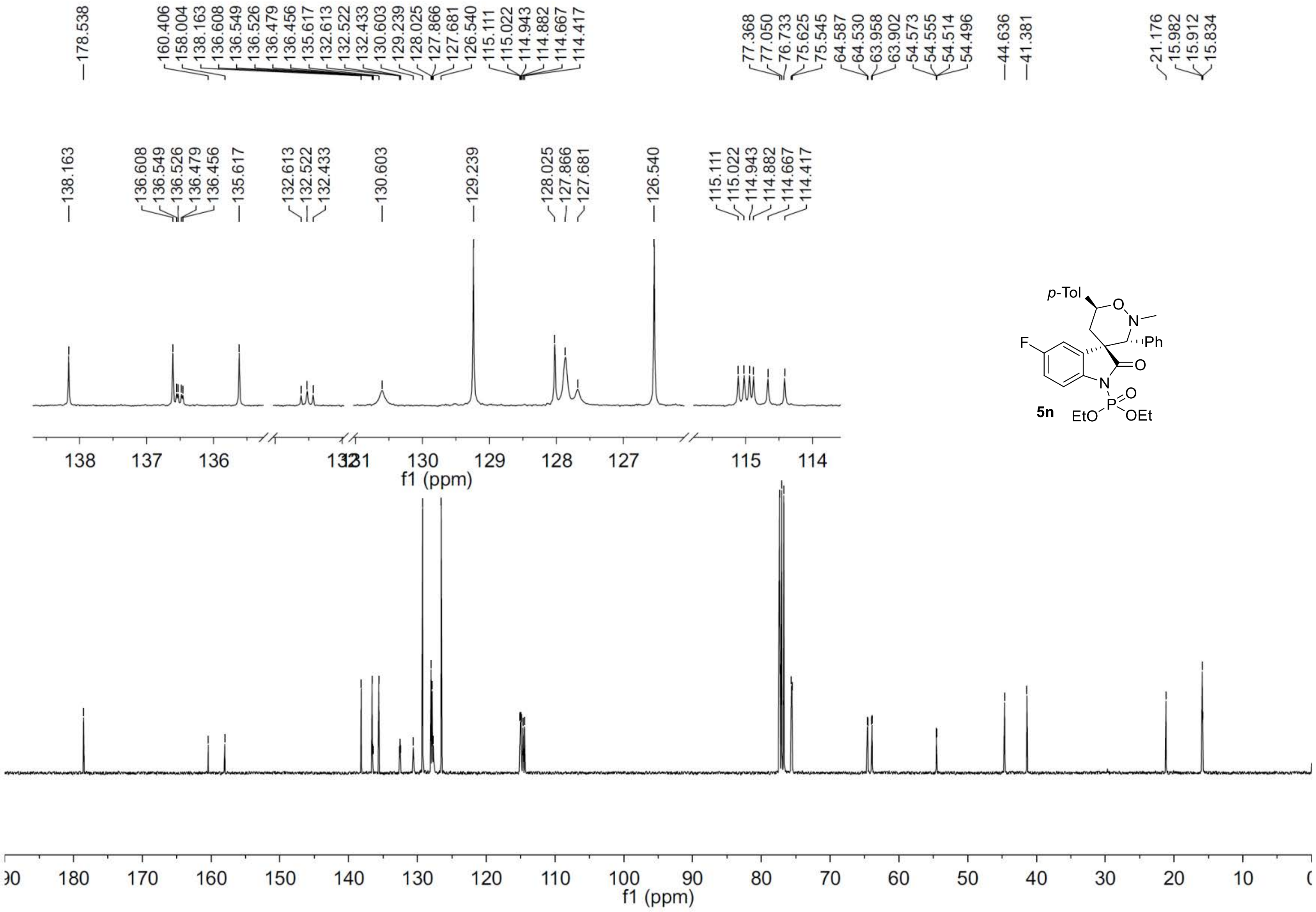

Supplementary Figure 72. ^{13}C NMR (100 MHz, $CDCl_3$) spectra for compound **5n**

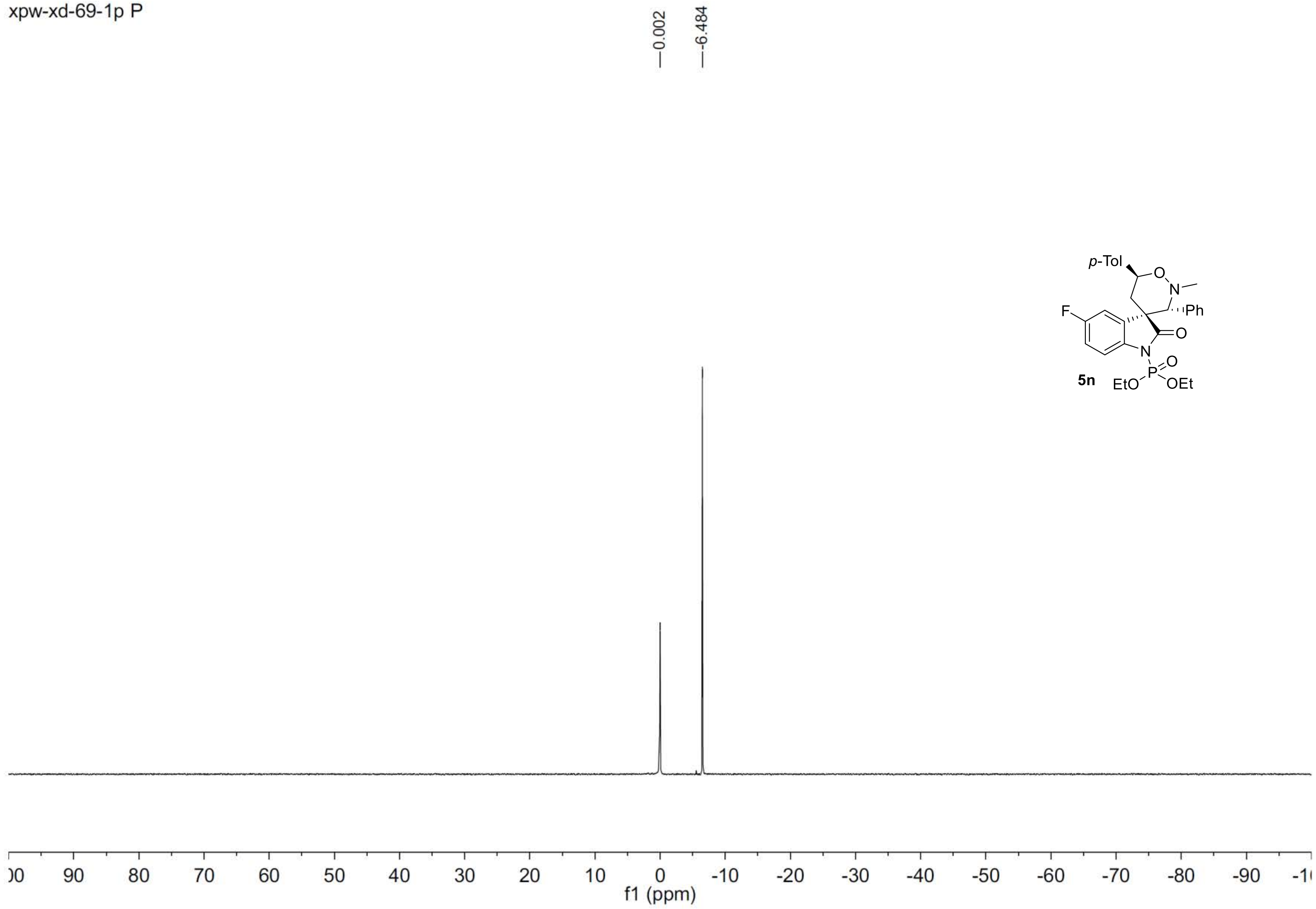

Supplementary Figure 73. ^{31}P NMR (122 MHz, $CDCl_3$) spectra for compound **5n**

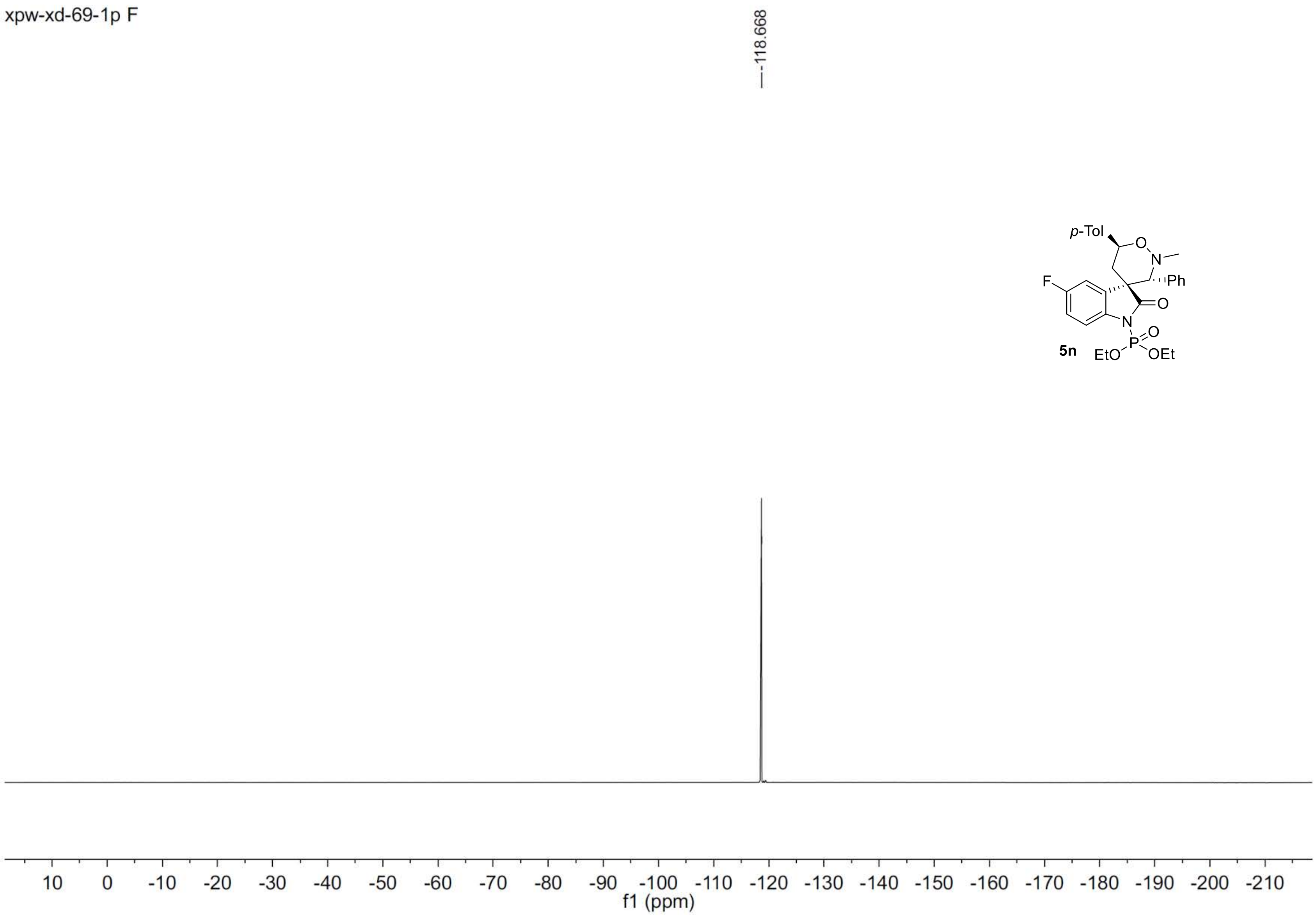

Supplementary Figure 74. ^{19}F NMR (282 MHz, $CDCl_3$) spectra for compound **5n**

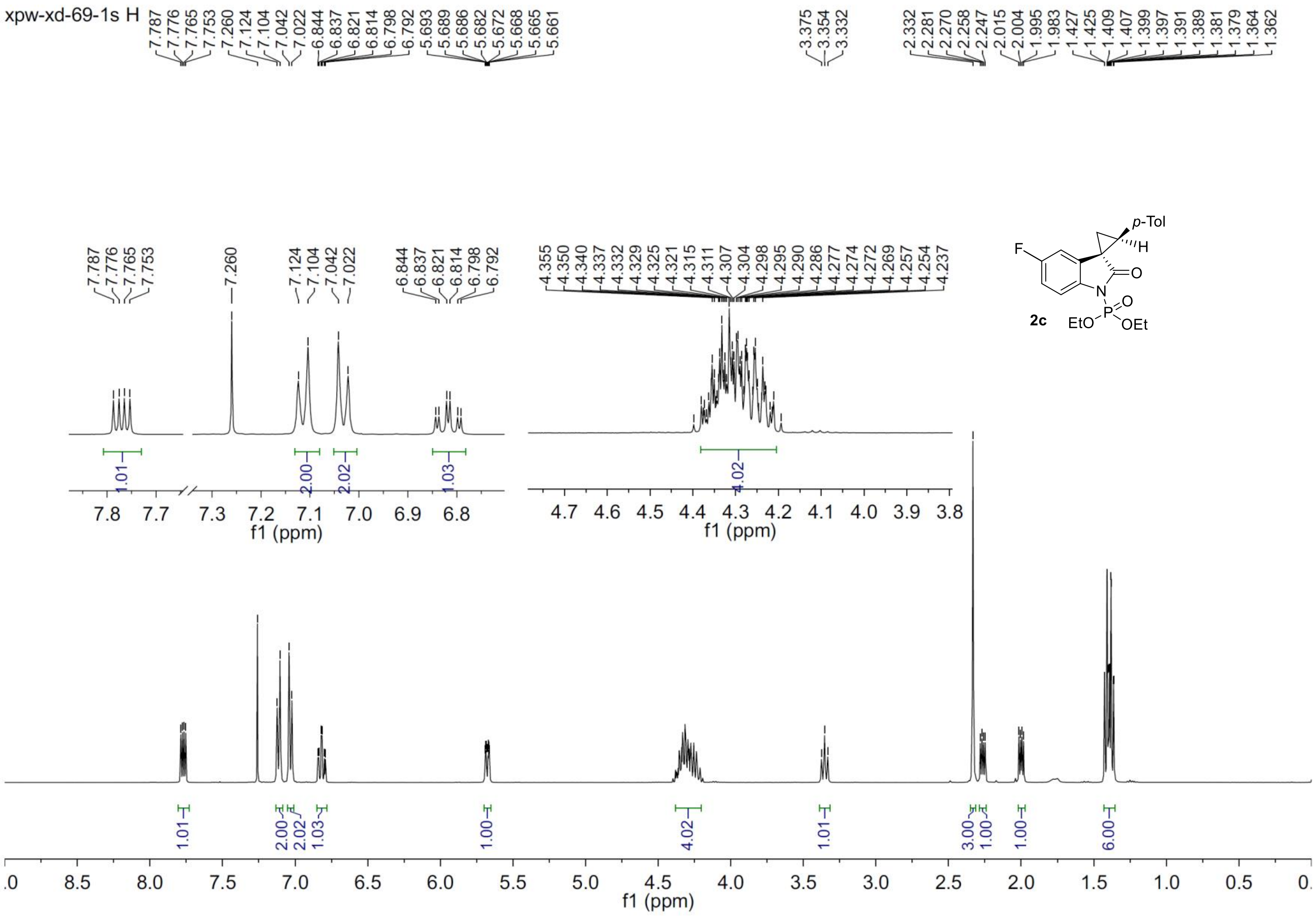

Supplementary Figure 75. ^{1}H NMR (400 MHz, $CDCl_3$) spectra for compound **2c**

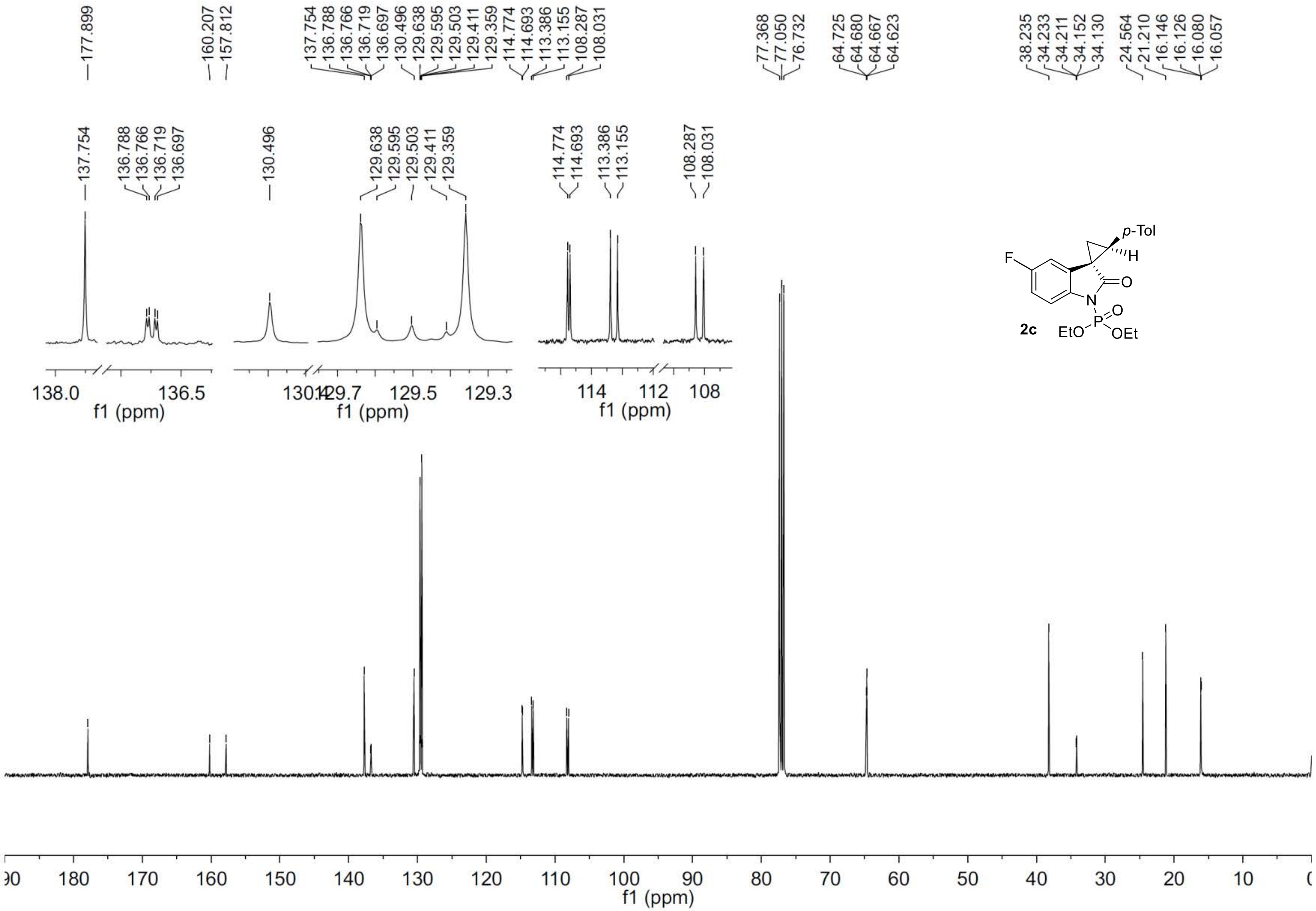

Supplementary Figure 76. ^{13}C NMR (100 MHz, $CDCl_3$) spectra for compound **2c**

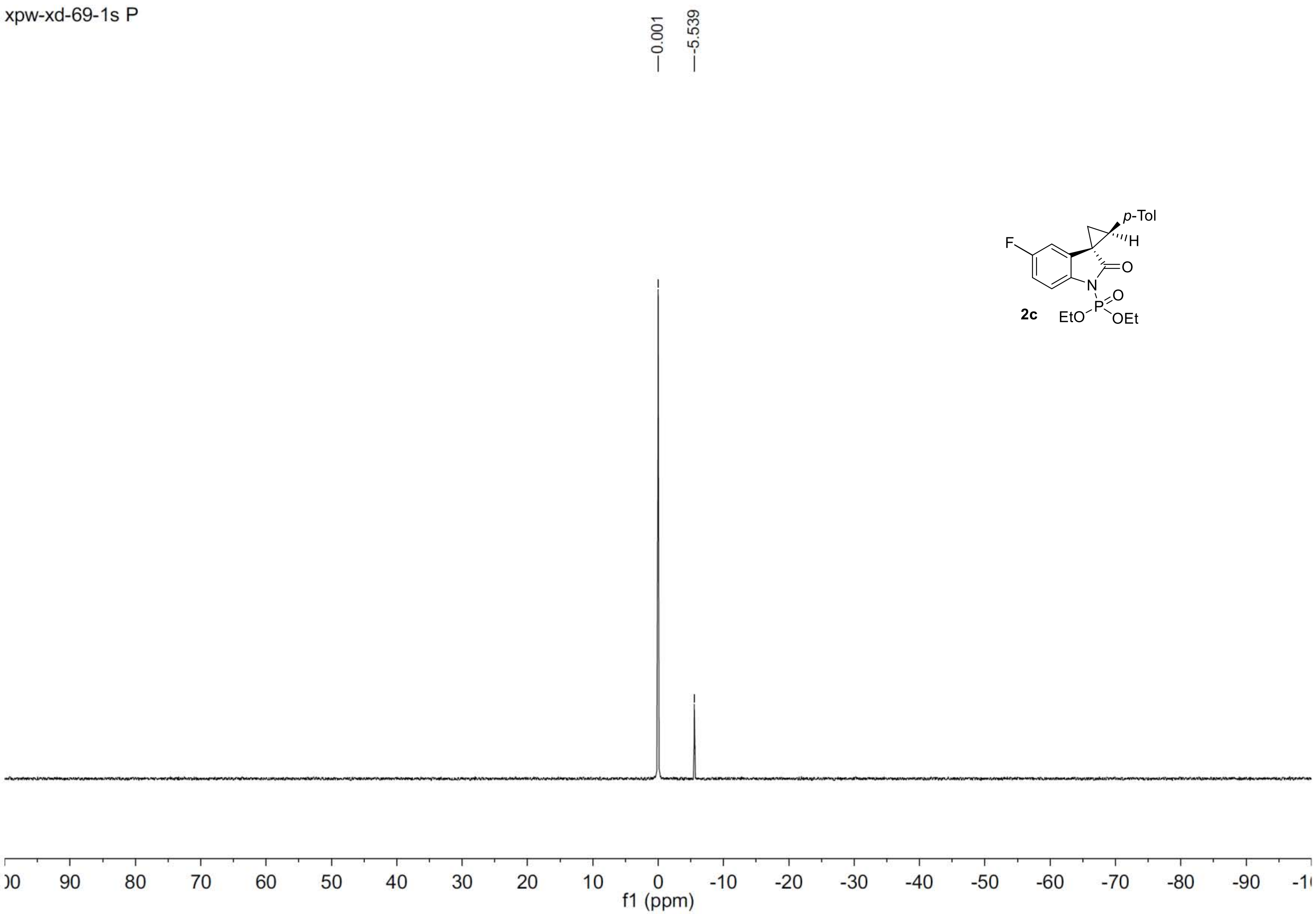

Supplementary Figure 77. ^{31}P NMR (122 MHz, $CDCl_3$) spectra for compound **2c**

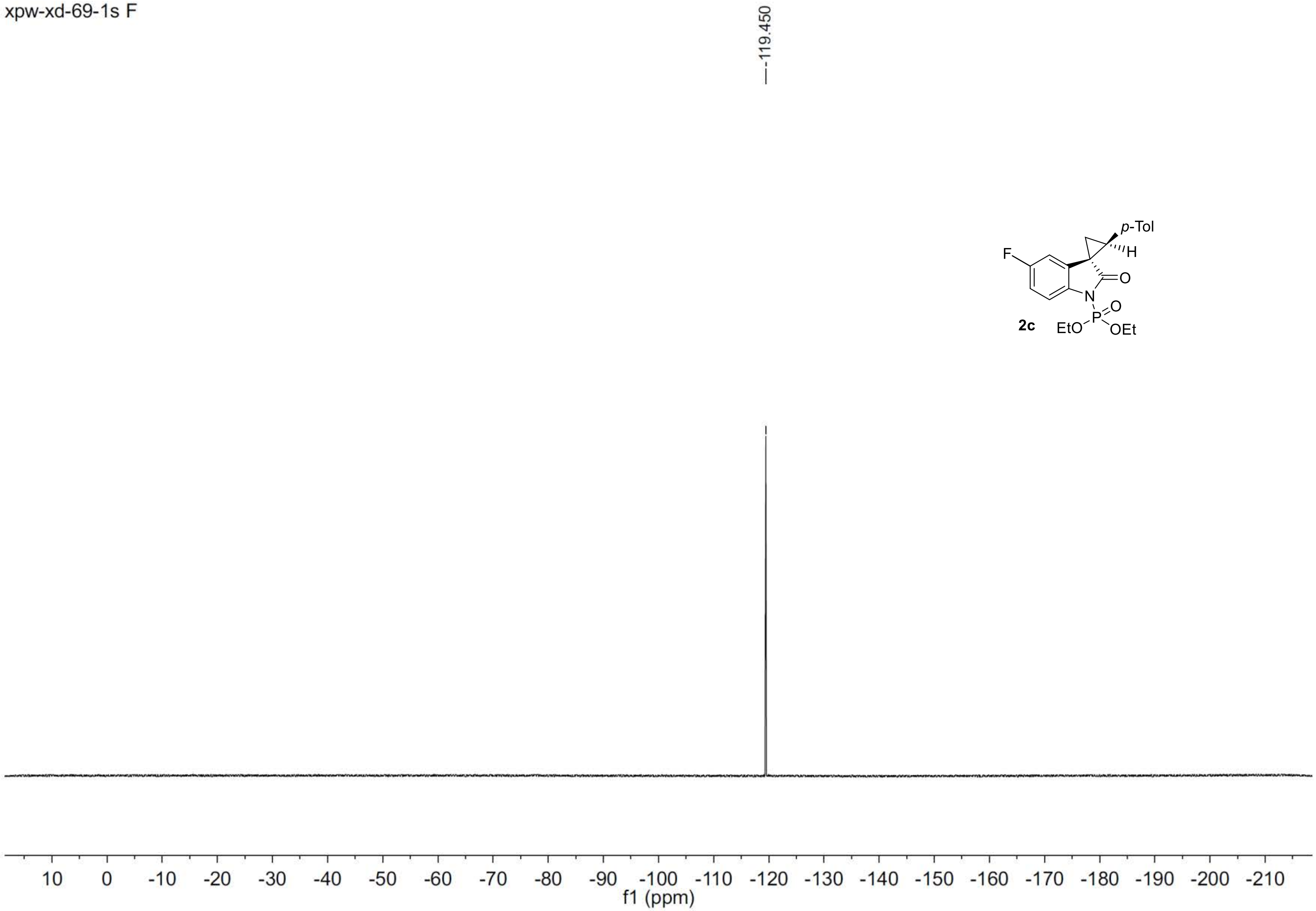

Supplementary Figure 78. ^{19}F NMR (282 MHz, $CDCl_3$) spectra for compound **2c**

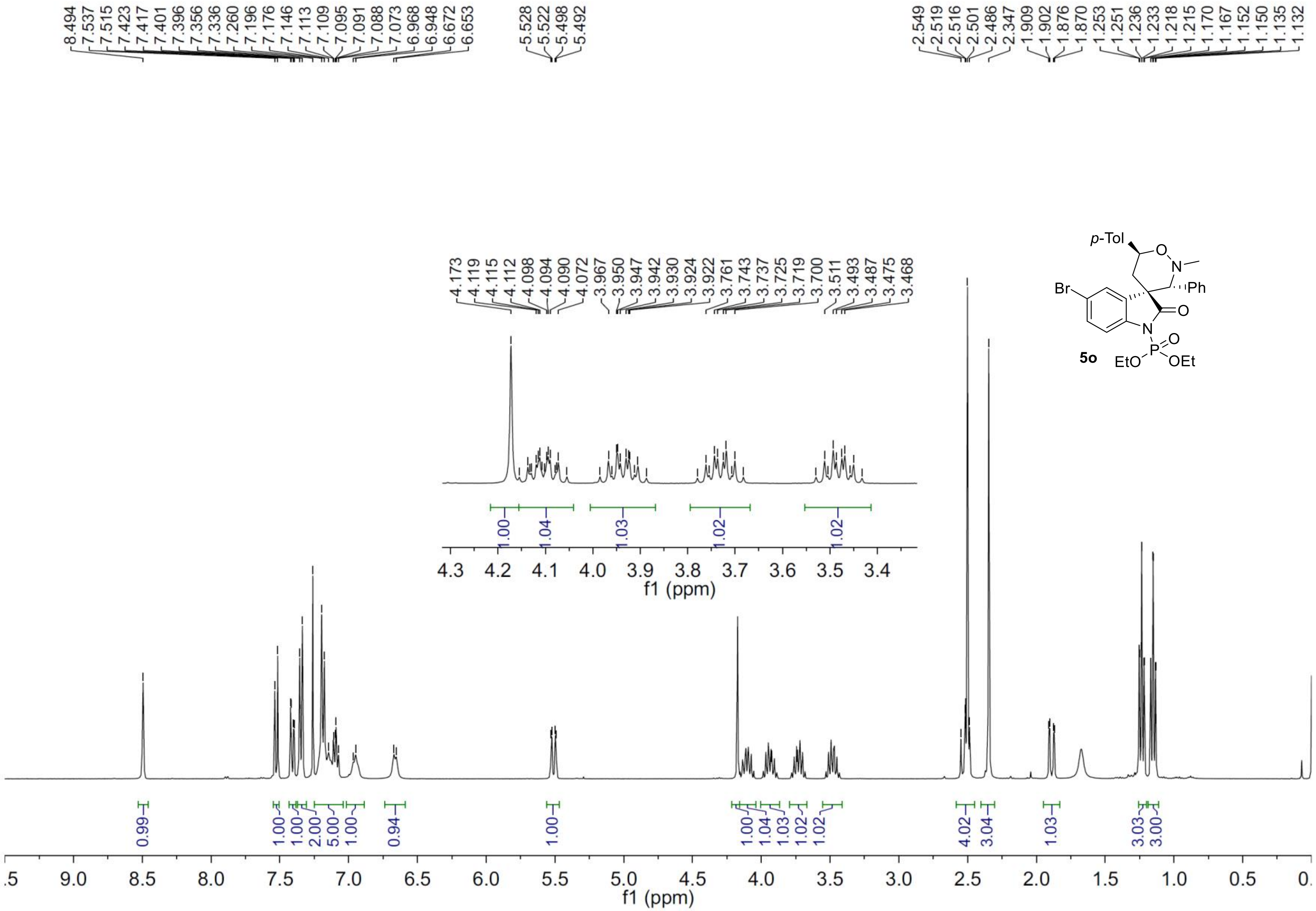

Supplementary Figure 79. ^{1}H NMR (400 MHz, $CDCl_3$) spectra for compound **5o**

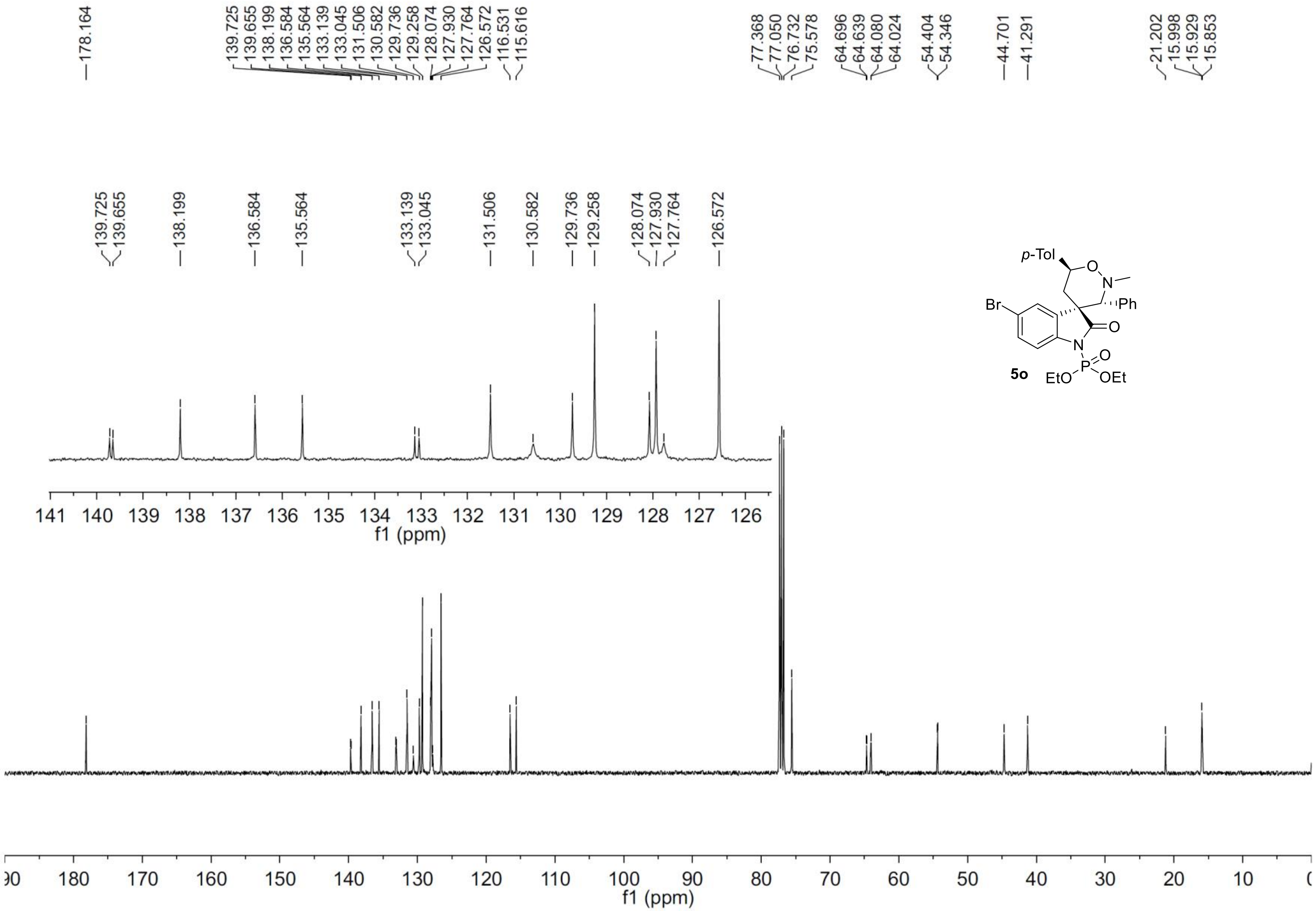

Supplementary Figure 80. ^{13}C NMR (100 MHz, $CDCl_3$) spectra for compound **5o**

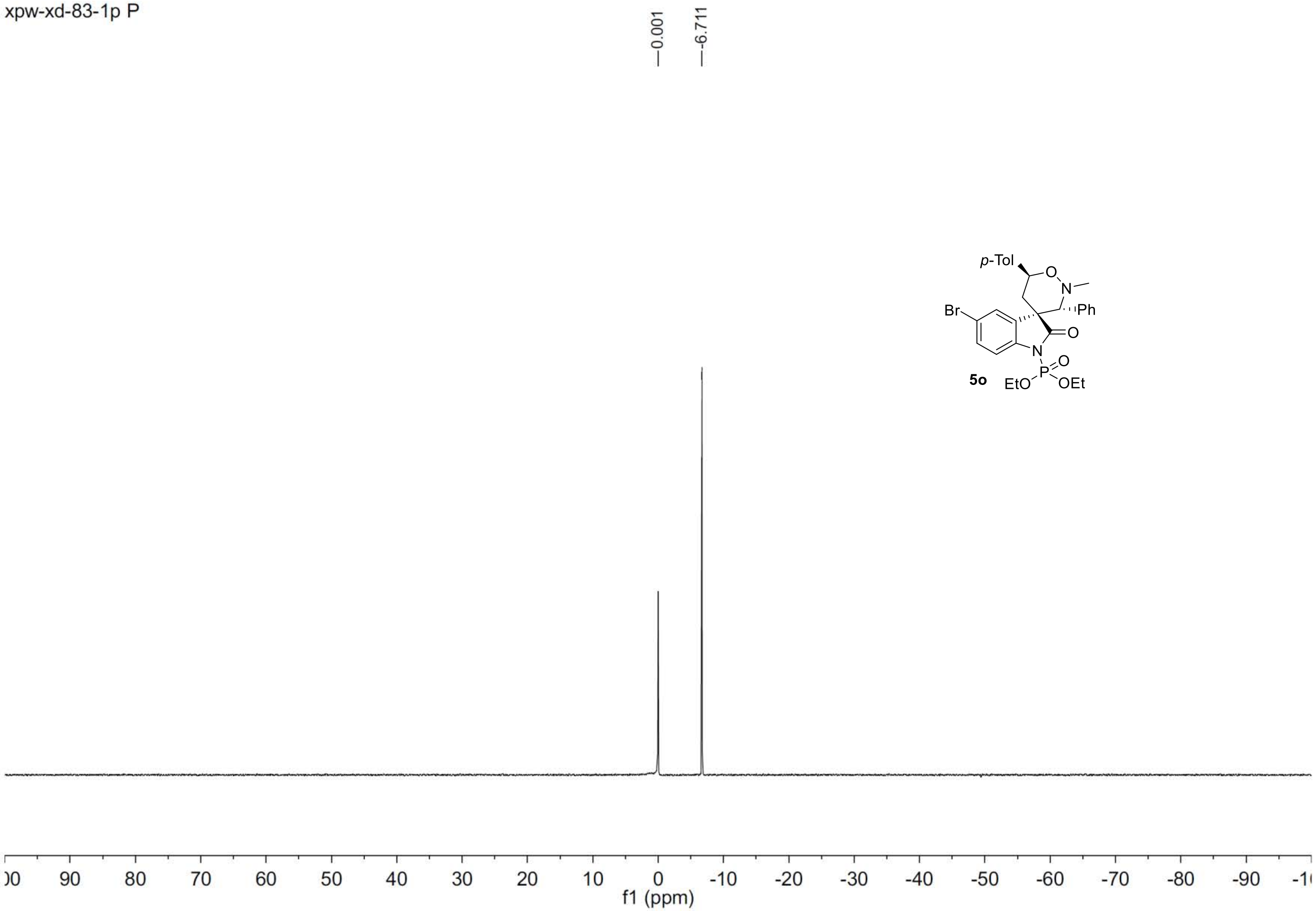

Supplementary Figure 81. ^{31}P NMR (162 MHz, $CDCl_3$) spectra for compound **5o**

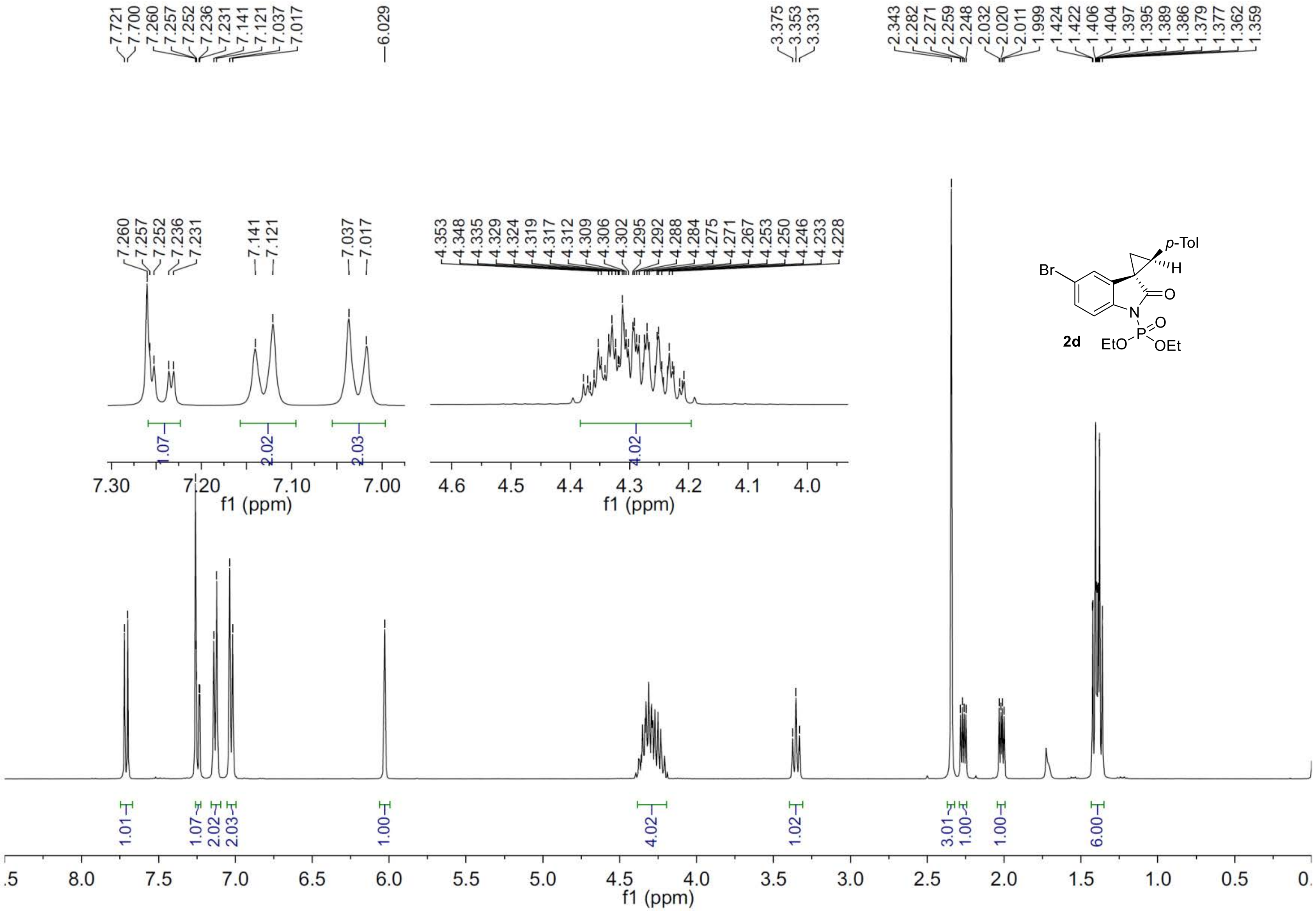

Supplementary Figure 82. ^{1}H NMR (400 MHz, $CDCl_3$) spectra for compound **2d**

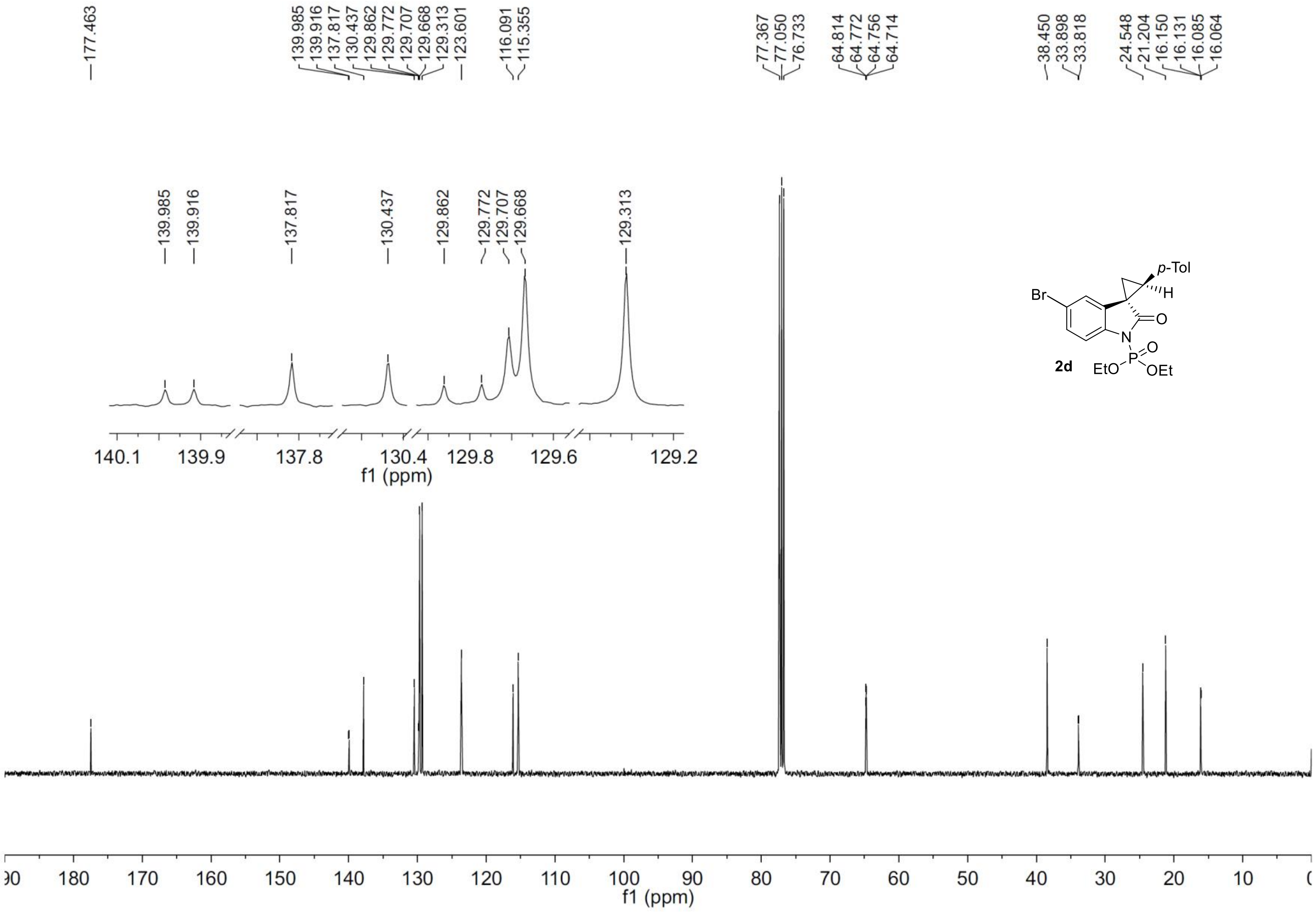

Supplementary Figure 83. ^{13}C NMR (100 MHz, $CDCl_3$) spectra for compound **2d**

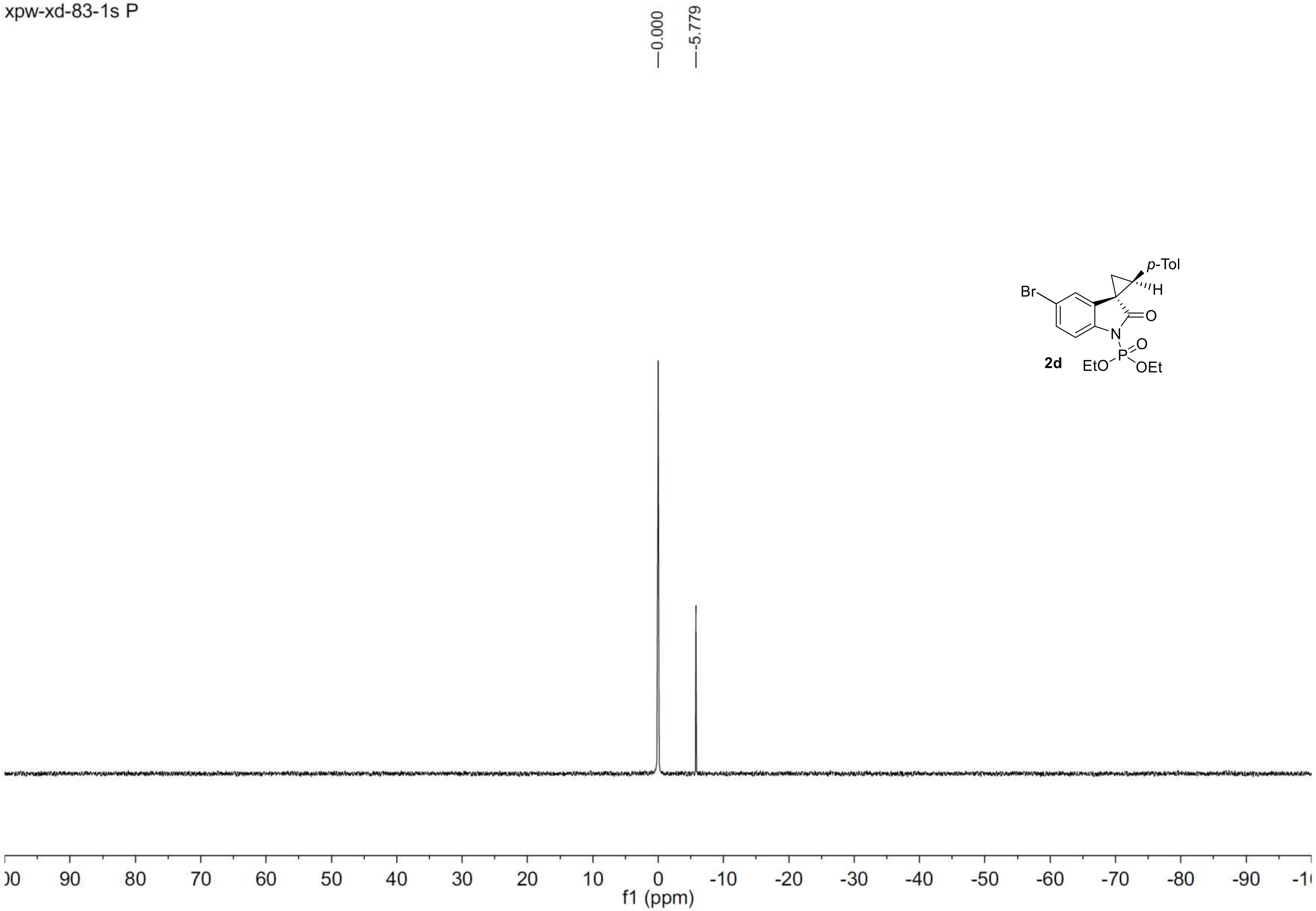

2d

Supplementary Figure 84. ^{31}P NMR (122 MHz, $CDCl_3$) spectra for compound **2d**

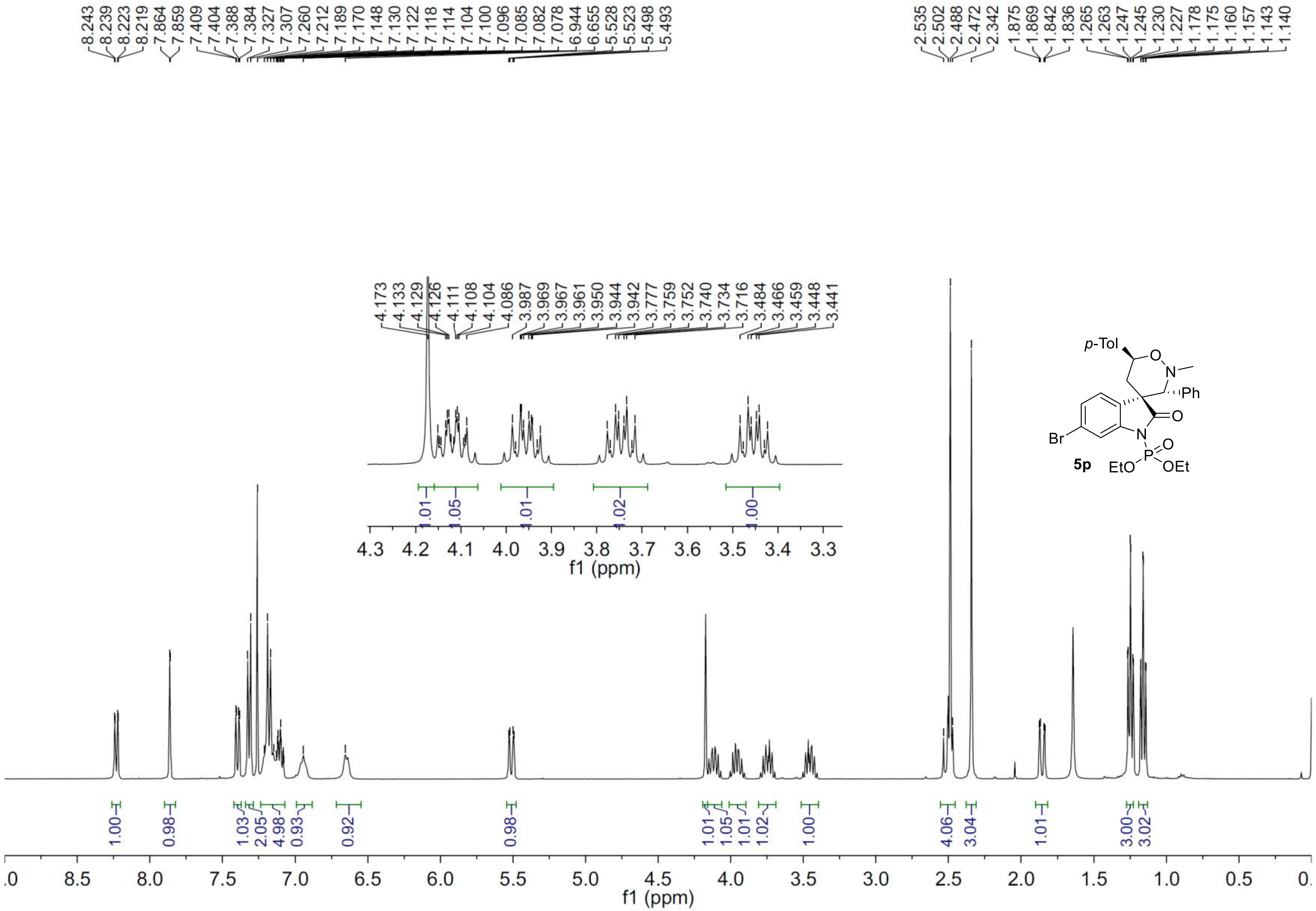

Supplementary Figure 85. ^{1}H NMR (400 MHz, $CDCl_3$) spectra for compound **5p**

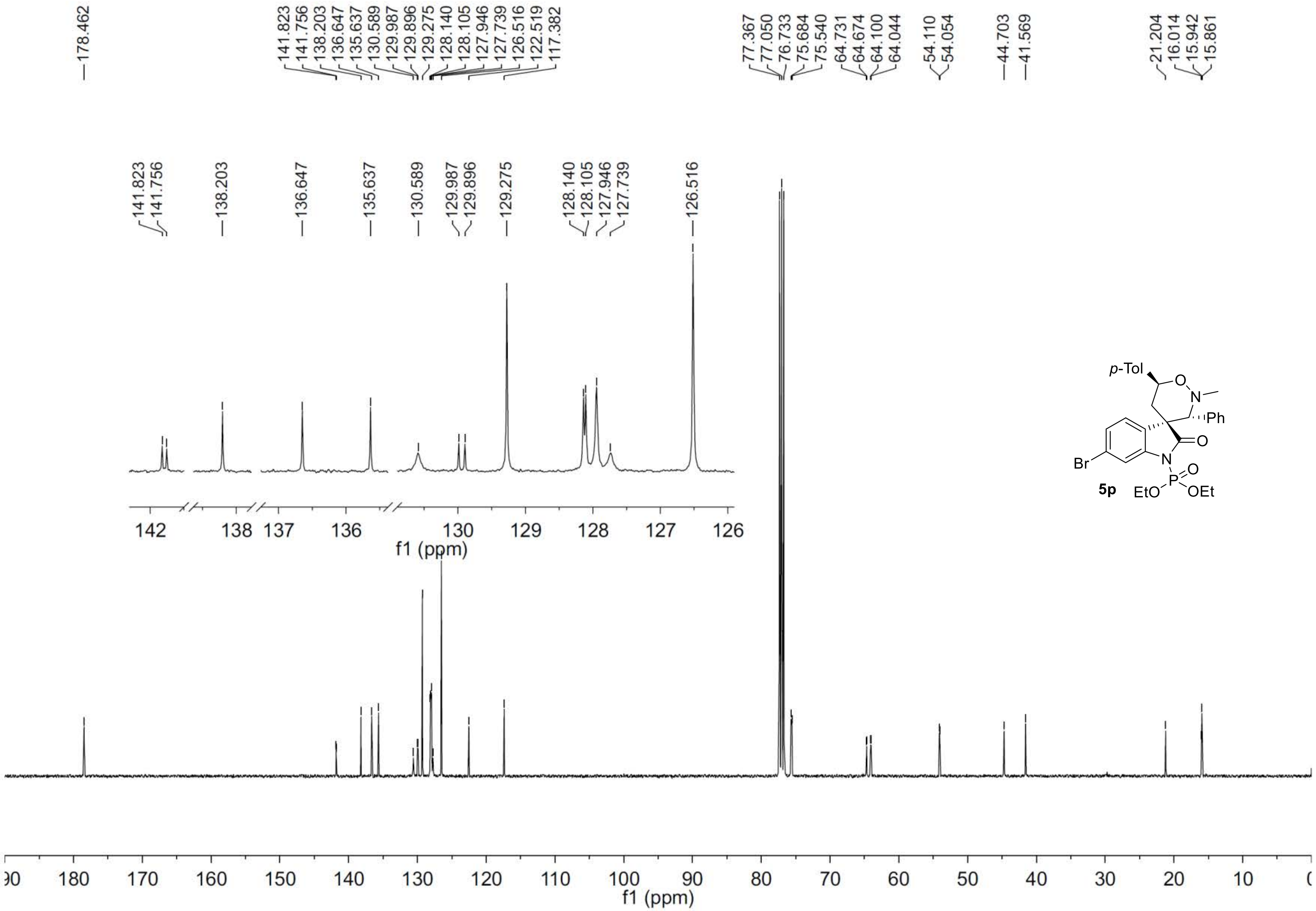

Supplementary Figure 86. ^{13}C NMR (100 MHz, $CDCl_3$) spectra for compound **5p**

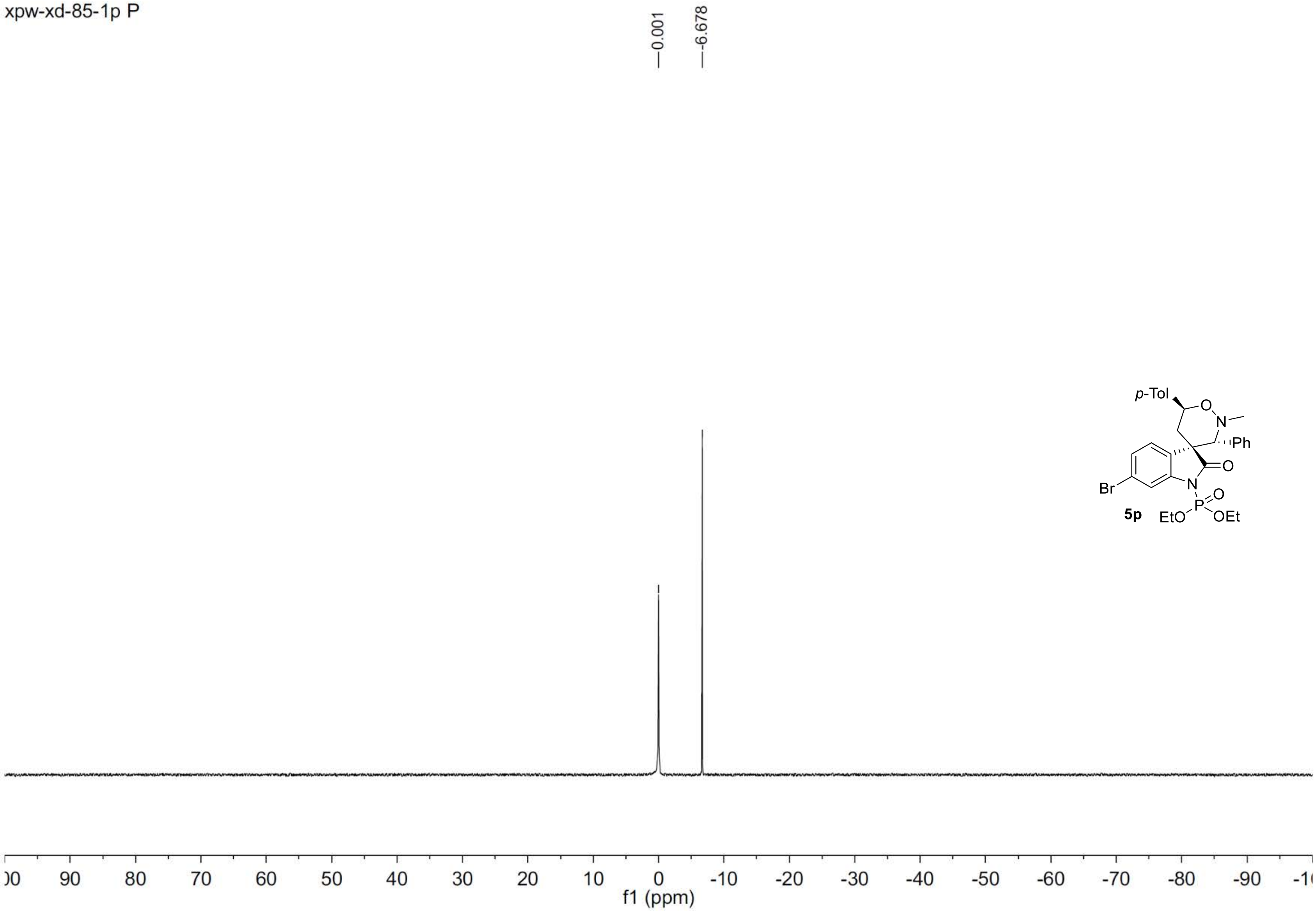

5p

Supplementary Figure 87. ^{31}P NMR (122 MHz, $CDCl_3$) spectra for compound **5p**

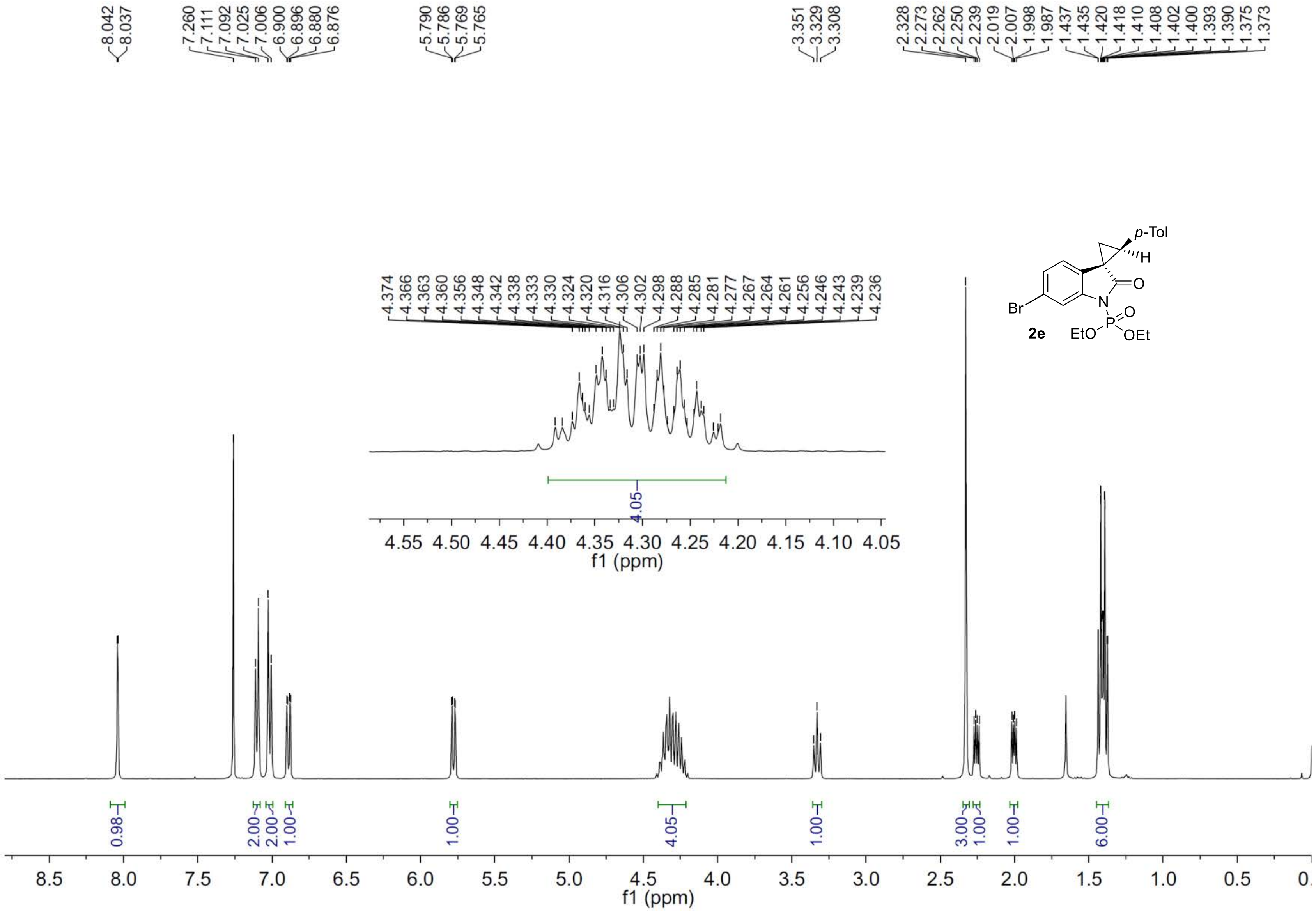

Supplementary Figure 88. ^{1}H NMR (400 MHz, $CDCl_3$) spectra for compound **2e**

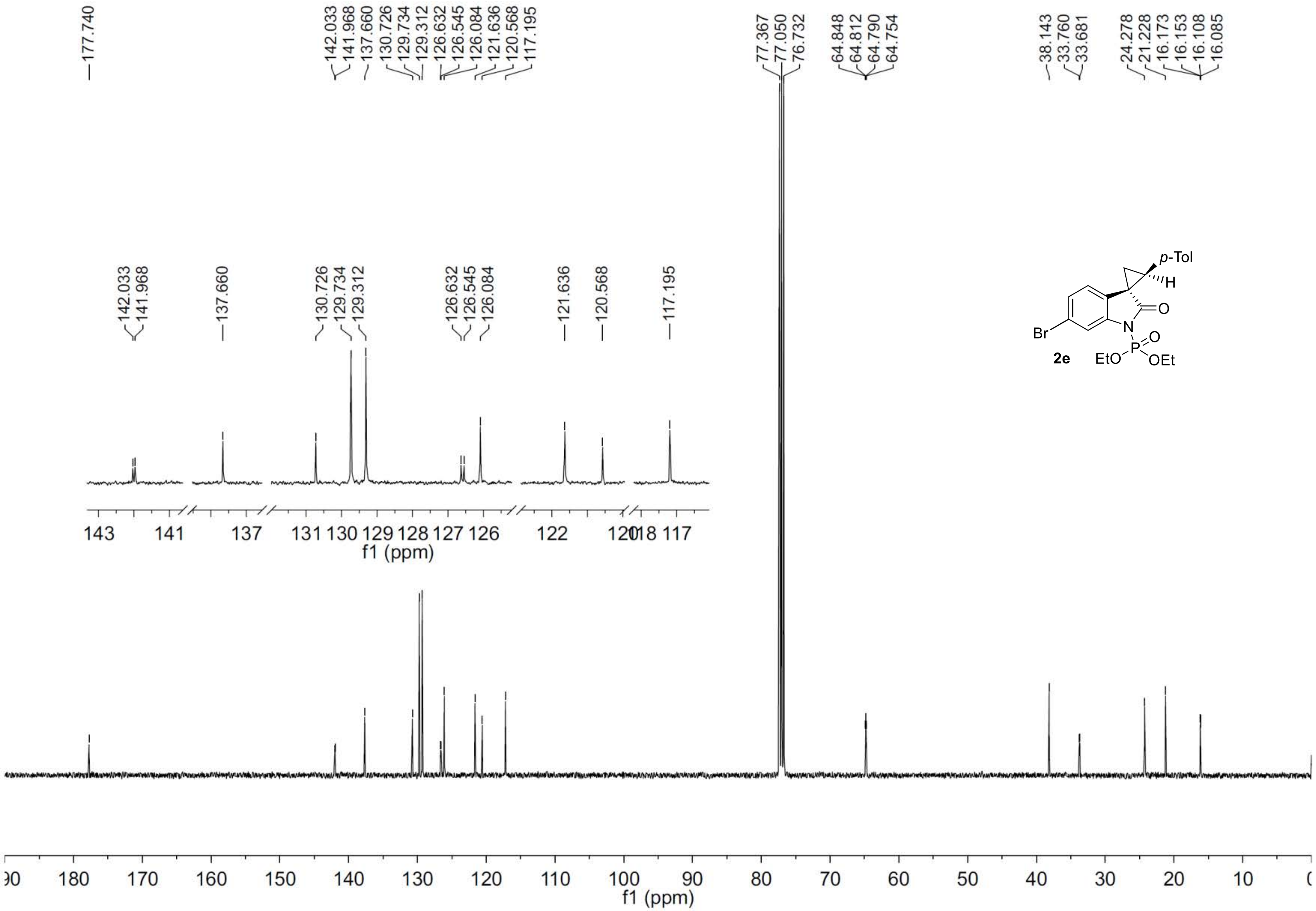

Supplementary Figure 89. ^{13}C NMR (100 MHz, $CDCl_3$) spectra for compound **2e**

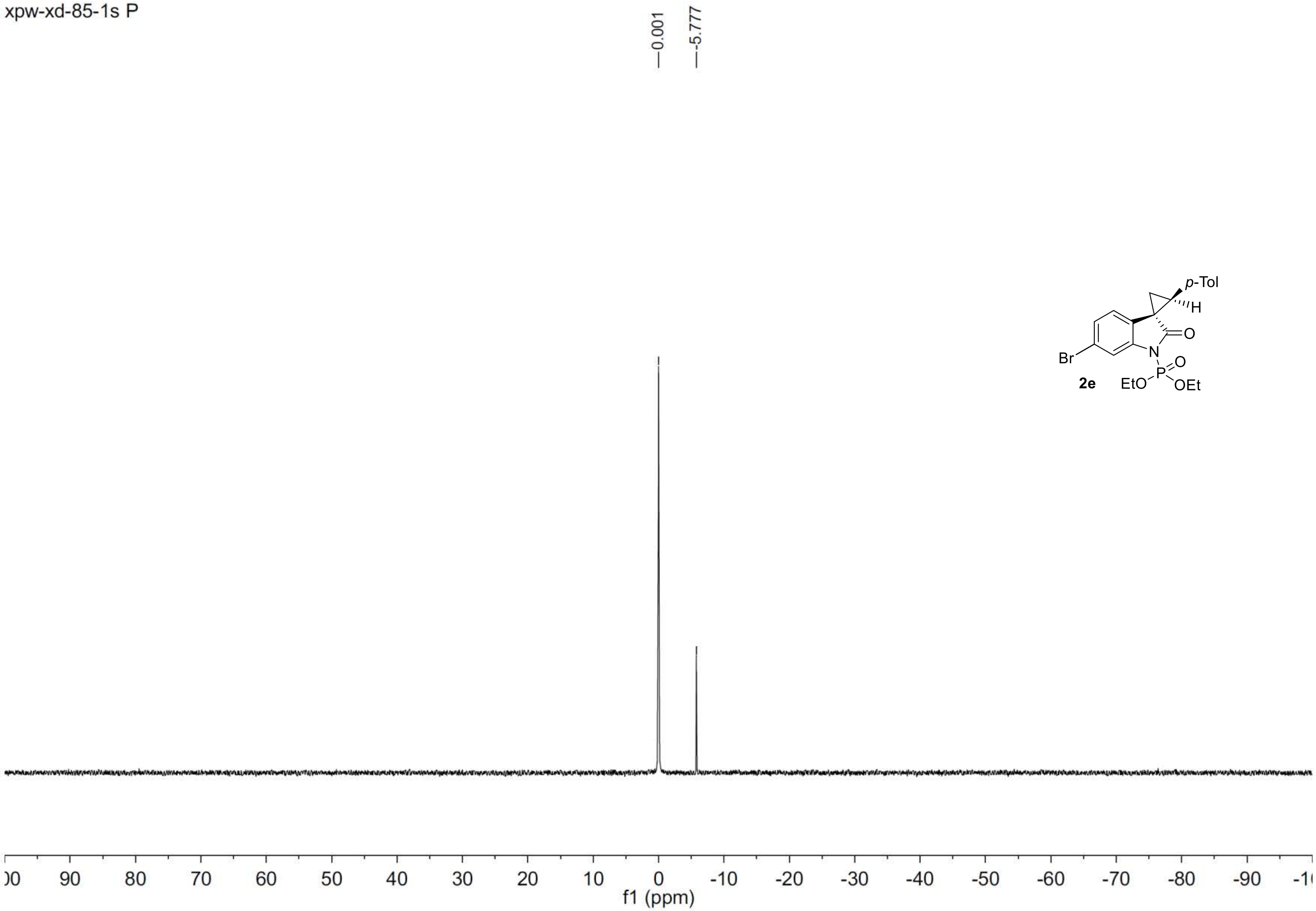

Supplementary Figure 90 ^{31}P NMR (122 MHz, $CDCl_3$) spectra for compound **2e**

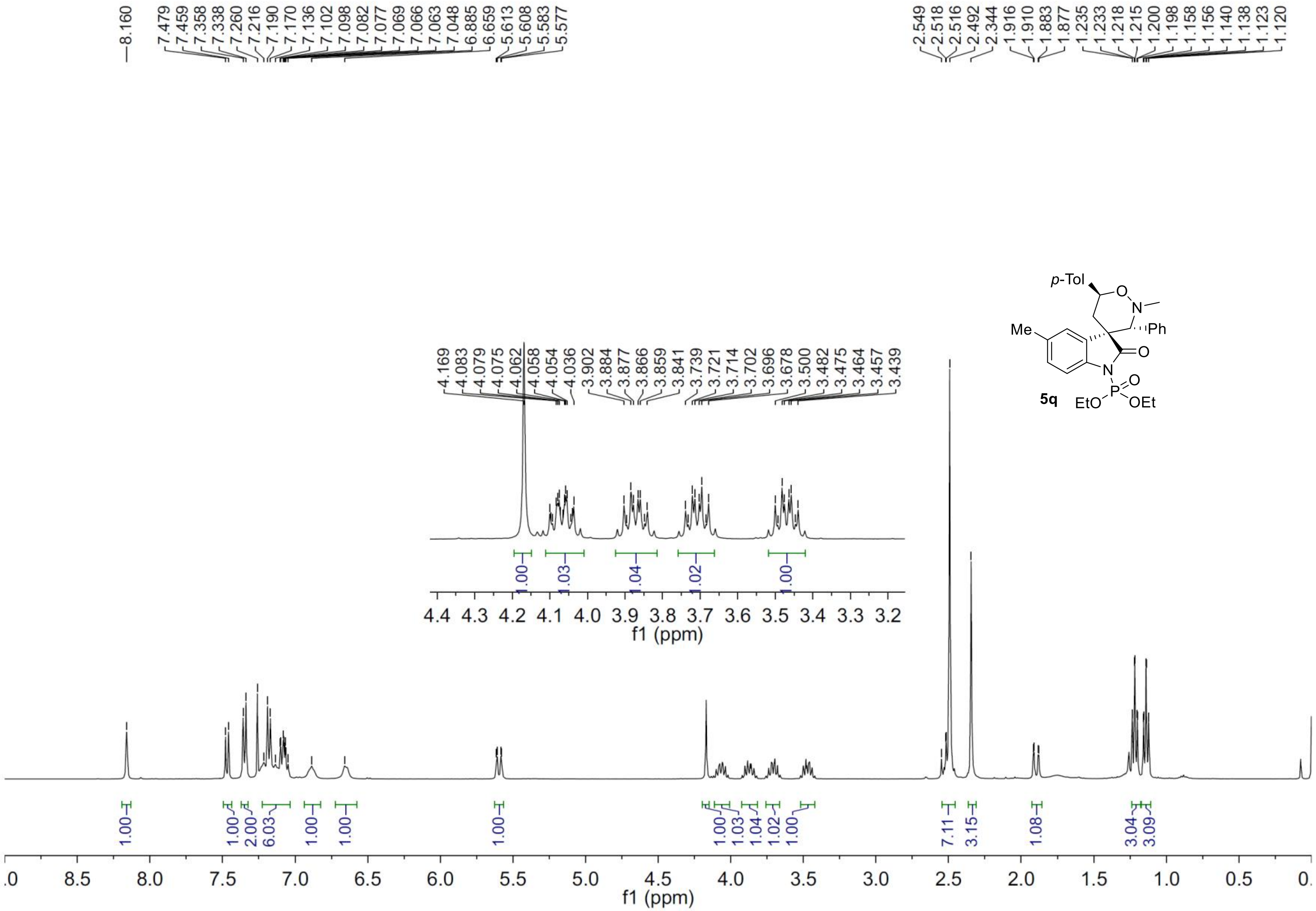

Supplementary Figure 91. ^{1}H NMR (400 MHz, $CDCl_3$) spectra for compound **5q**

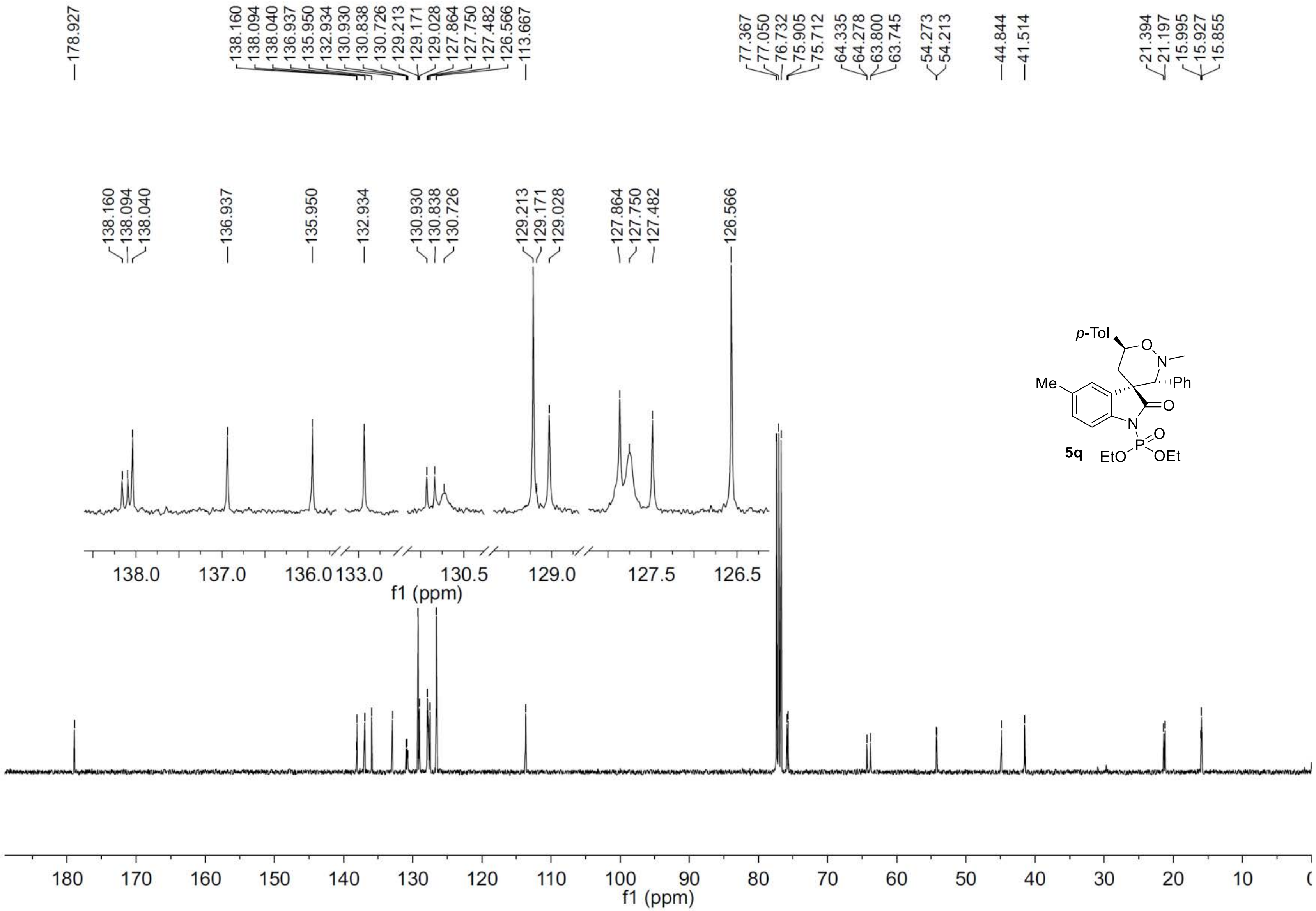

Supplementary Figure 92. ^{13}C NMR (100 MHz, $CDCl_3$) spectra for compound **5q**

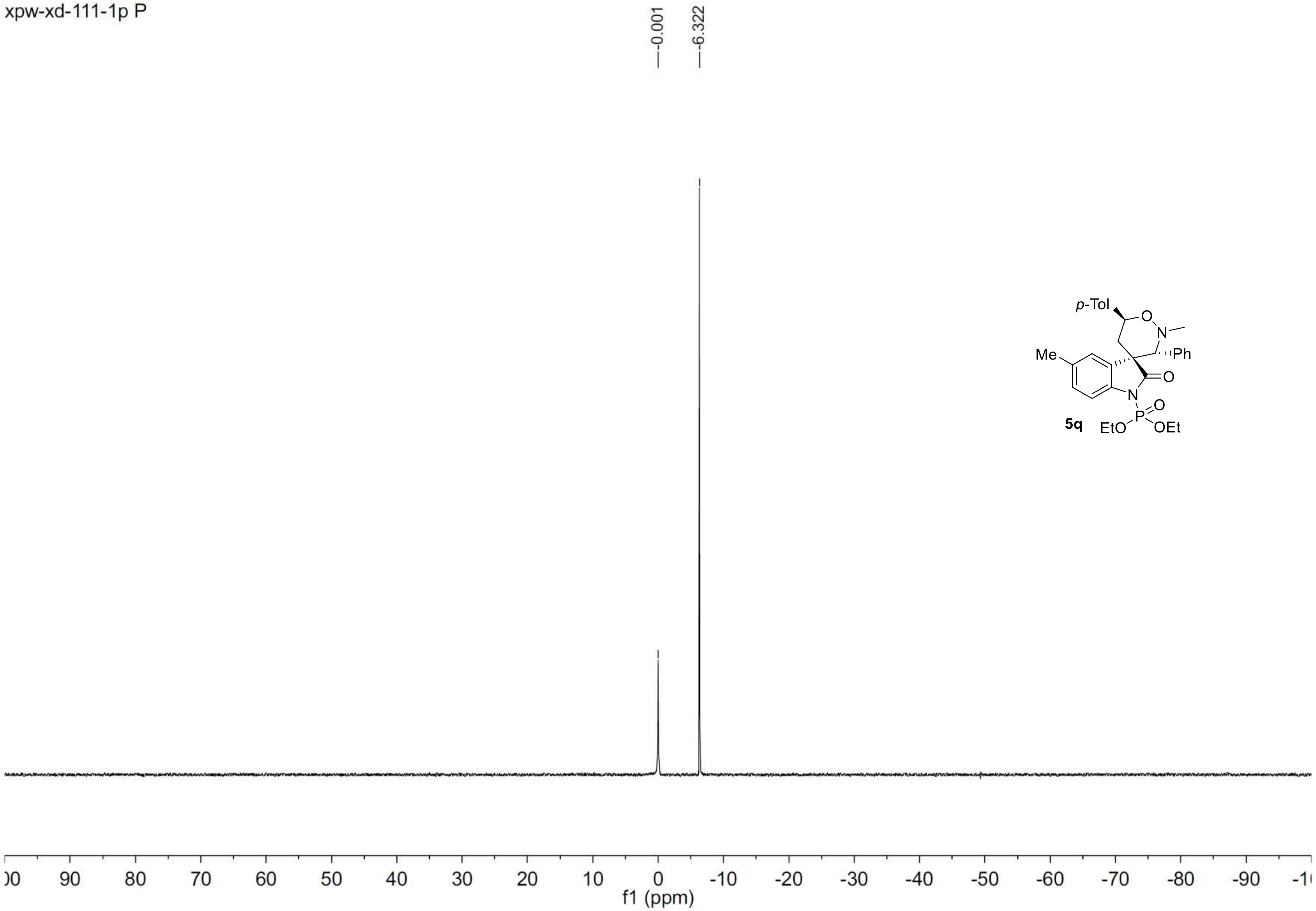

Supplementary Figure 93. ^{31}P NMR (162 MHz, $CDCl_3$) spectra for compound **5q**

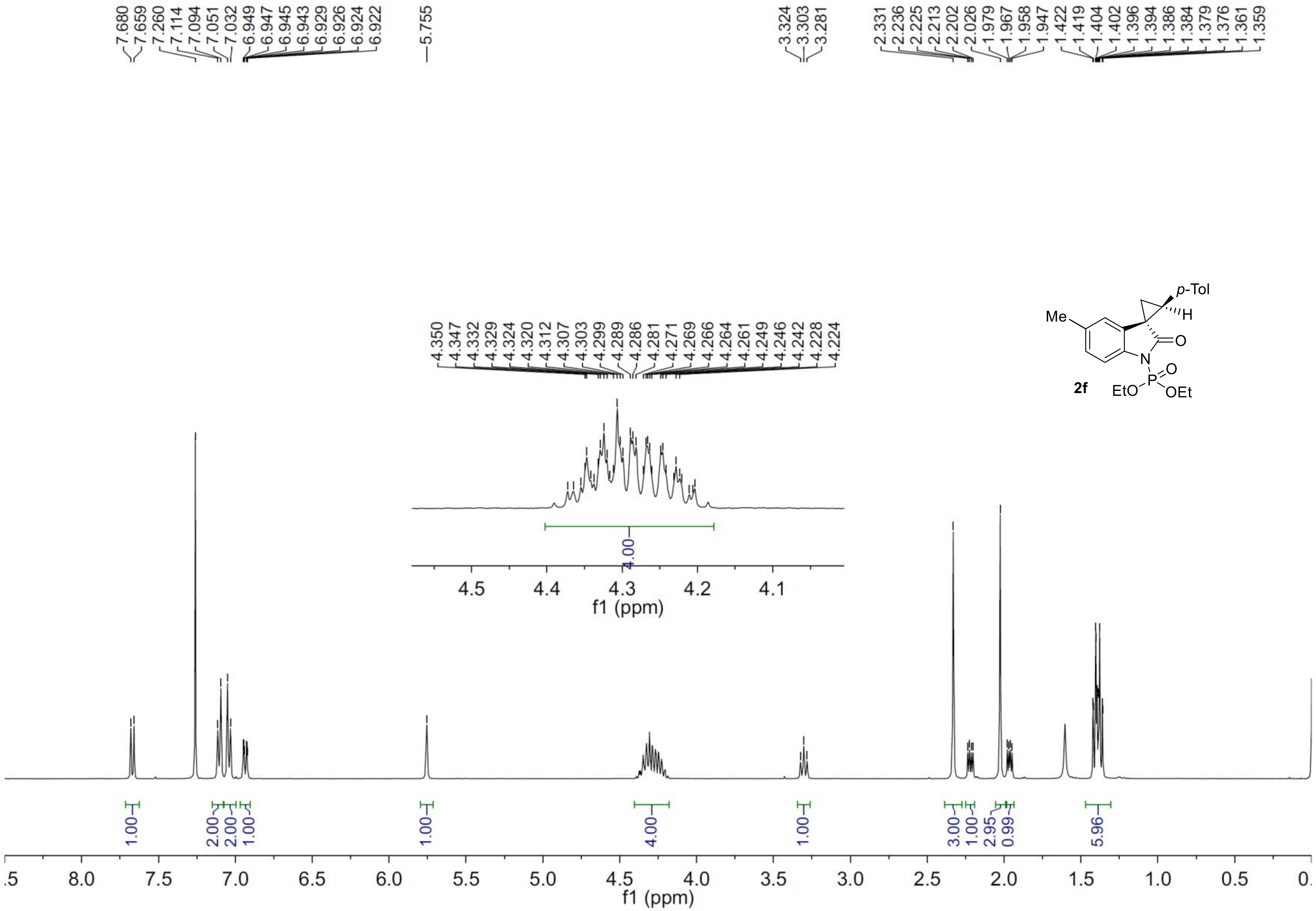

Supplementary Figure 94. ^{1}H NMR (400 MHz, $CDCl_3$) spectra for compound **2f**

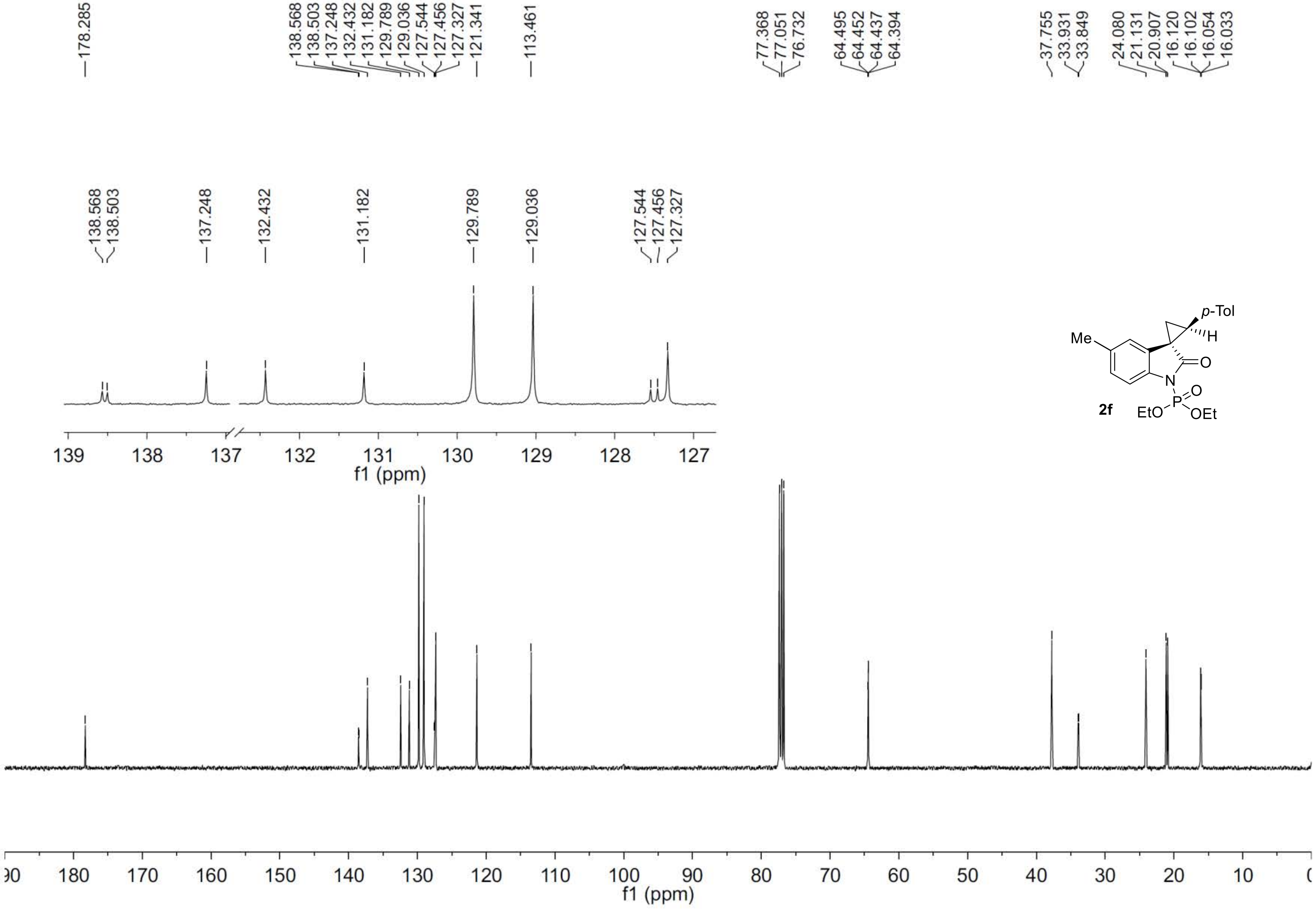

Supplementary Figure 95. ^{13}C NMR (100 MHz, $CDCl_3$) spectra for compound **2f**

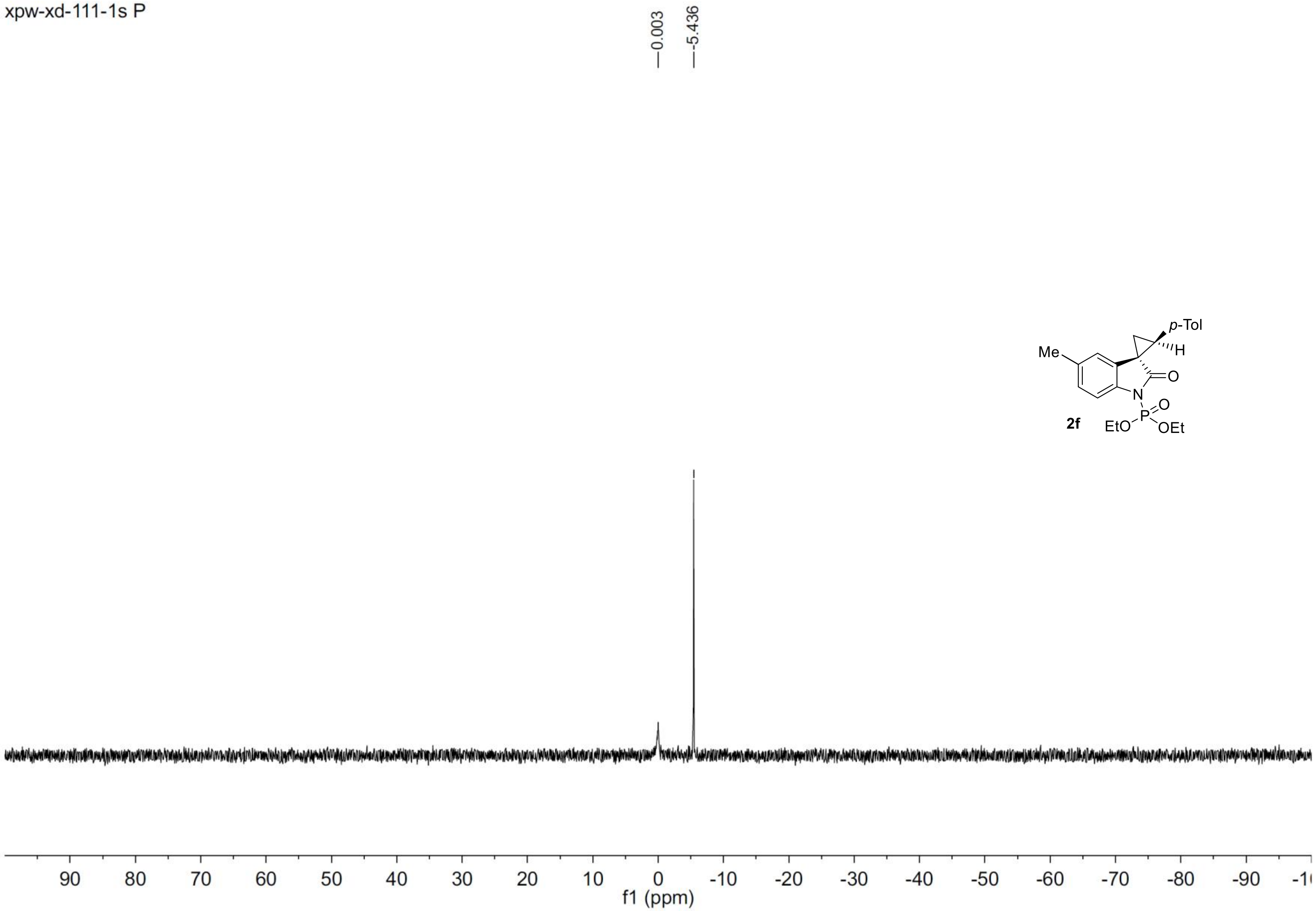

2f

Supplementary Figure 96. ^{31}P NMR (122 MHz, $CDCl_3$) spectra for compound **2f**

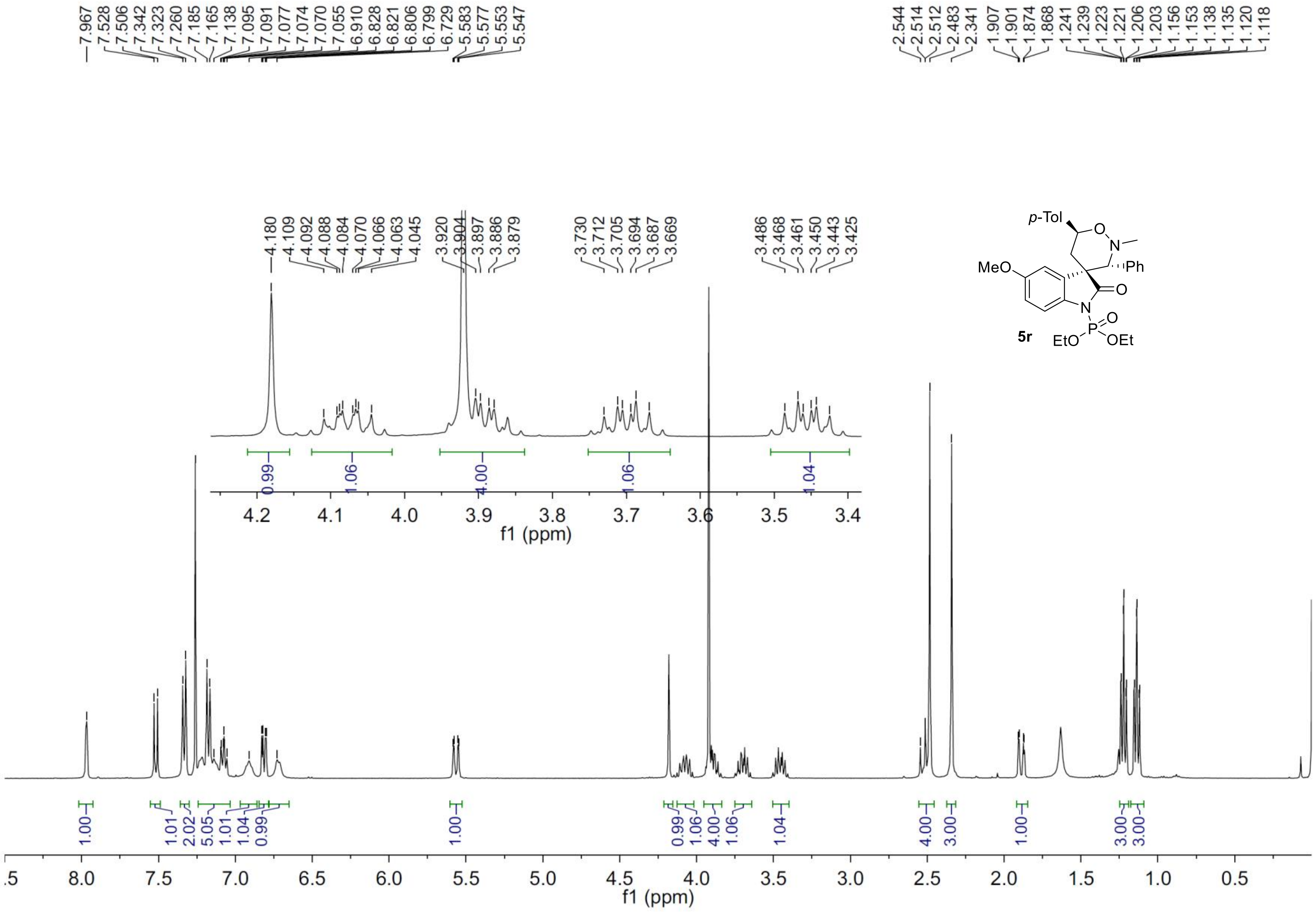

Supplementary Figure 97. ^{1}H NMR (400 MHz, $CDCl_3$) spectra for compound **5r**

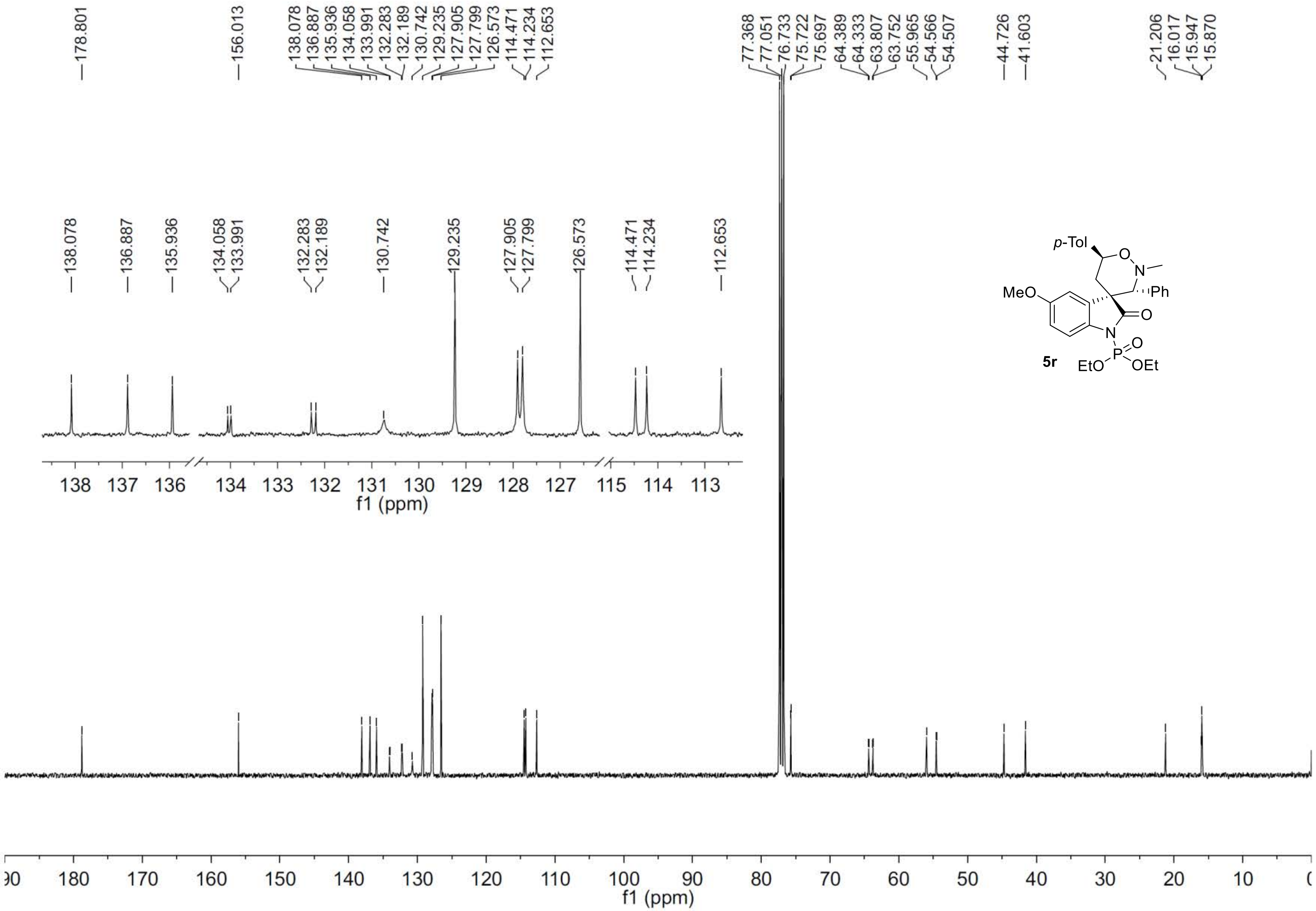

Supplementary Figure 98. ^{13}C NMR (100 MHz, $CDCl_3$) spectra for compound **5r**

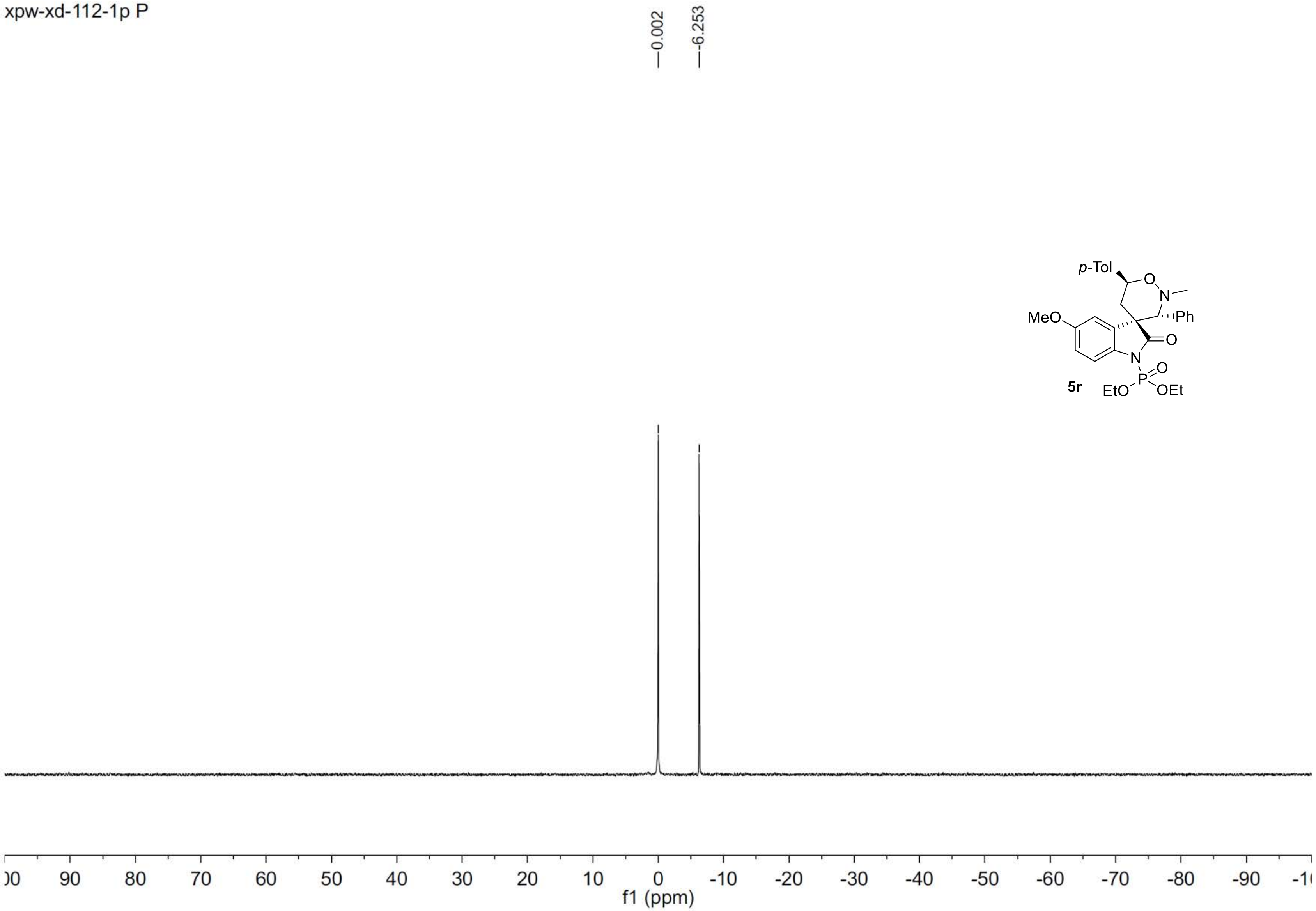

Supplementary Figure 99. ^{31}P NMR (122 MHz, $CDCl_3$) spectra for compound **5r**

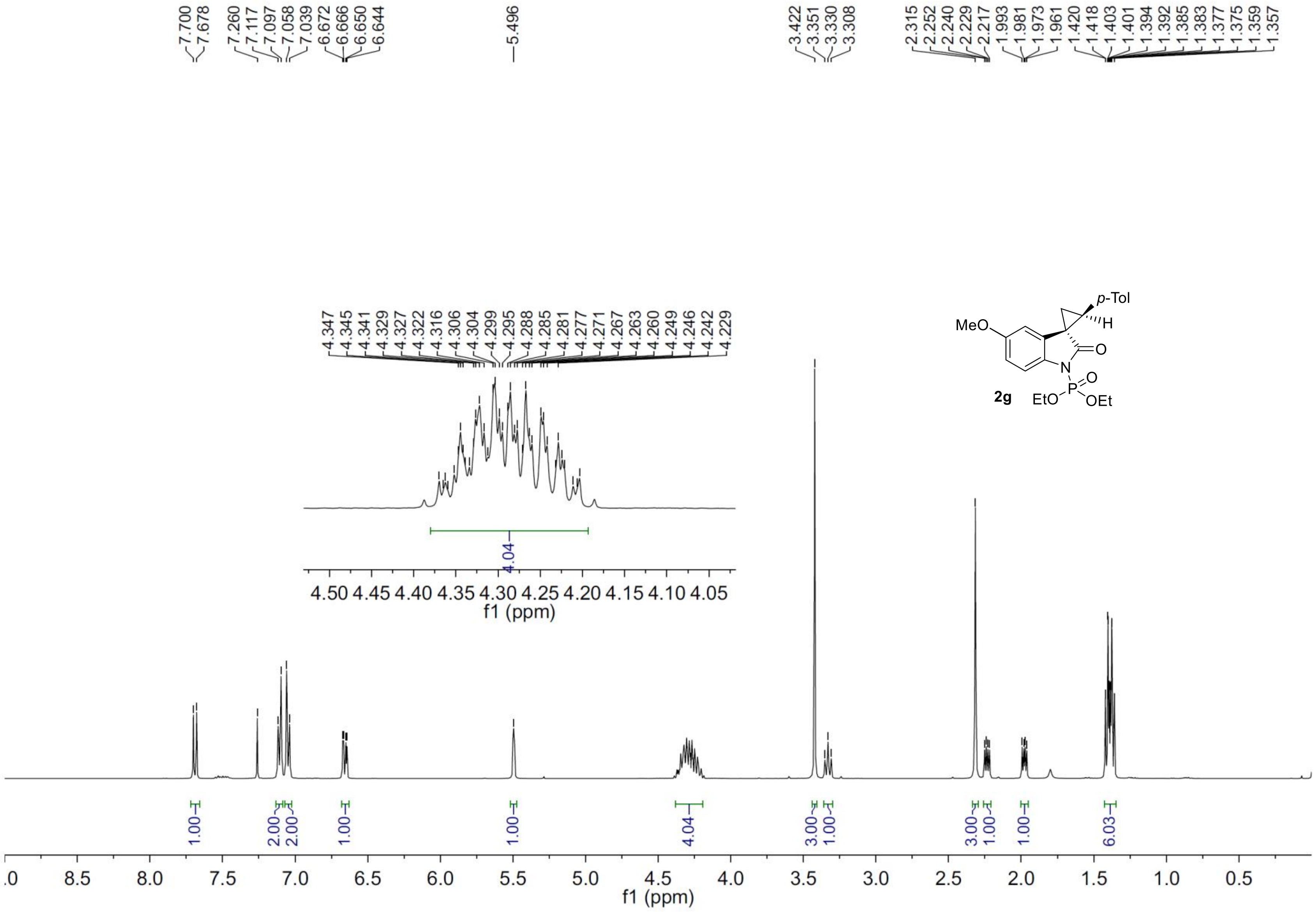

Supplementary Figure 100. ^{1}H NMR (400 MHz, $CDCl_3$) spectra for compound **2g**

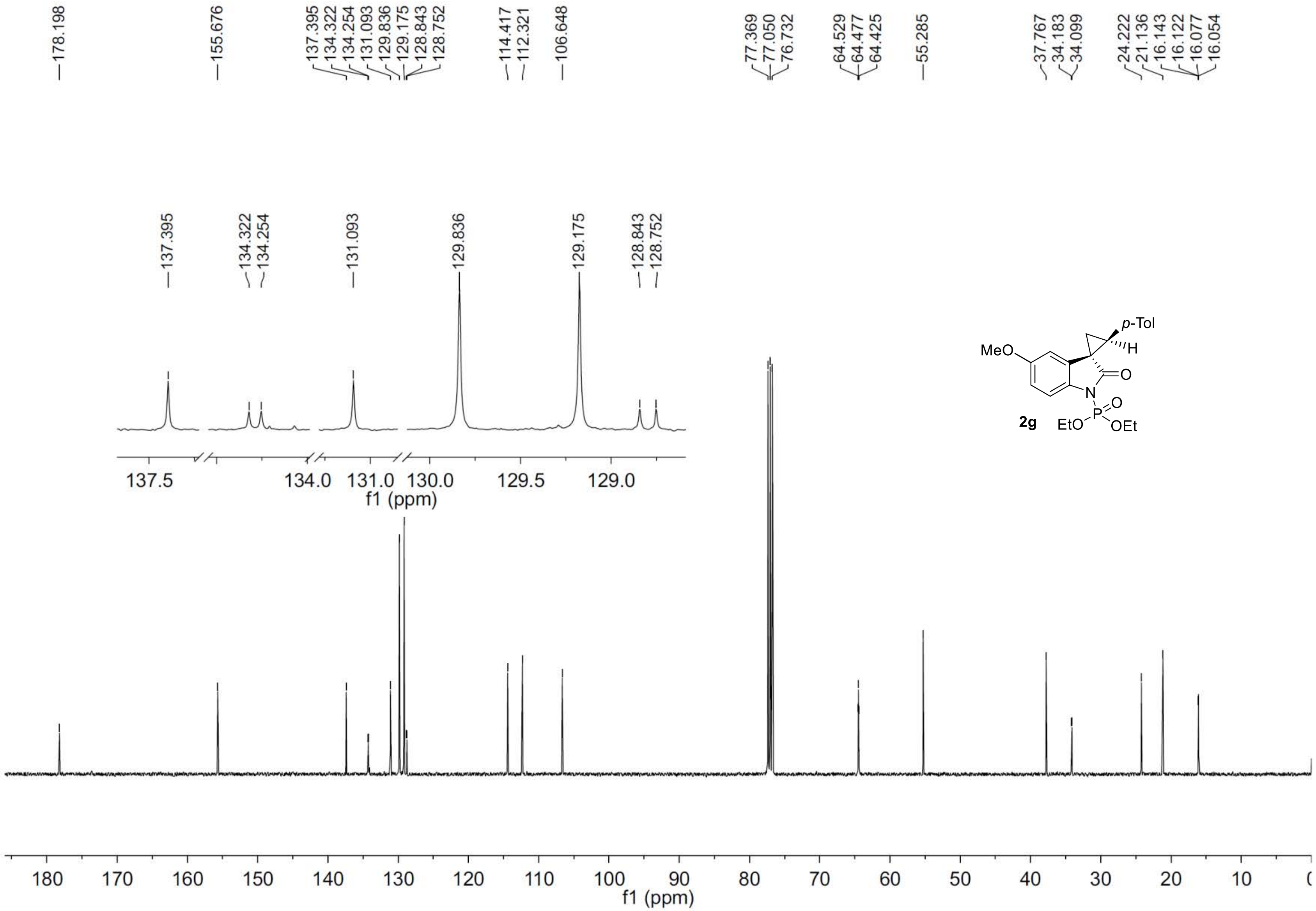

Supplementary Figure 101. ^{13}C NMR (100 MHz, $CDCl_3$) spectra for compound **2g**

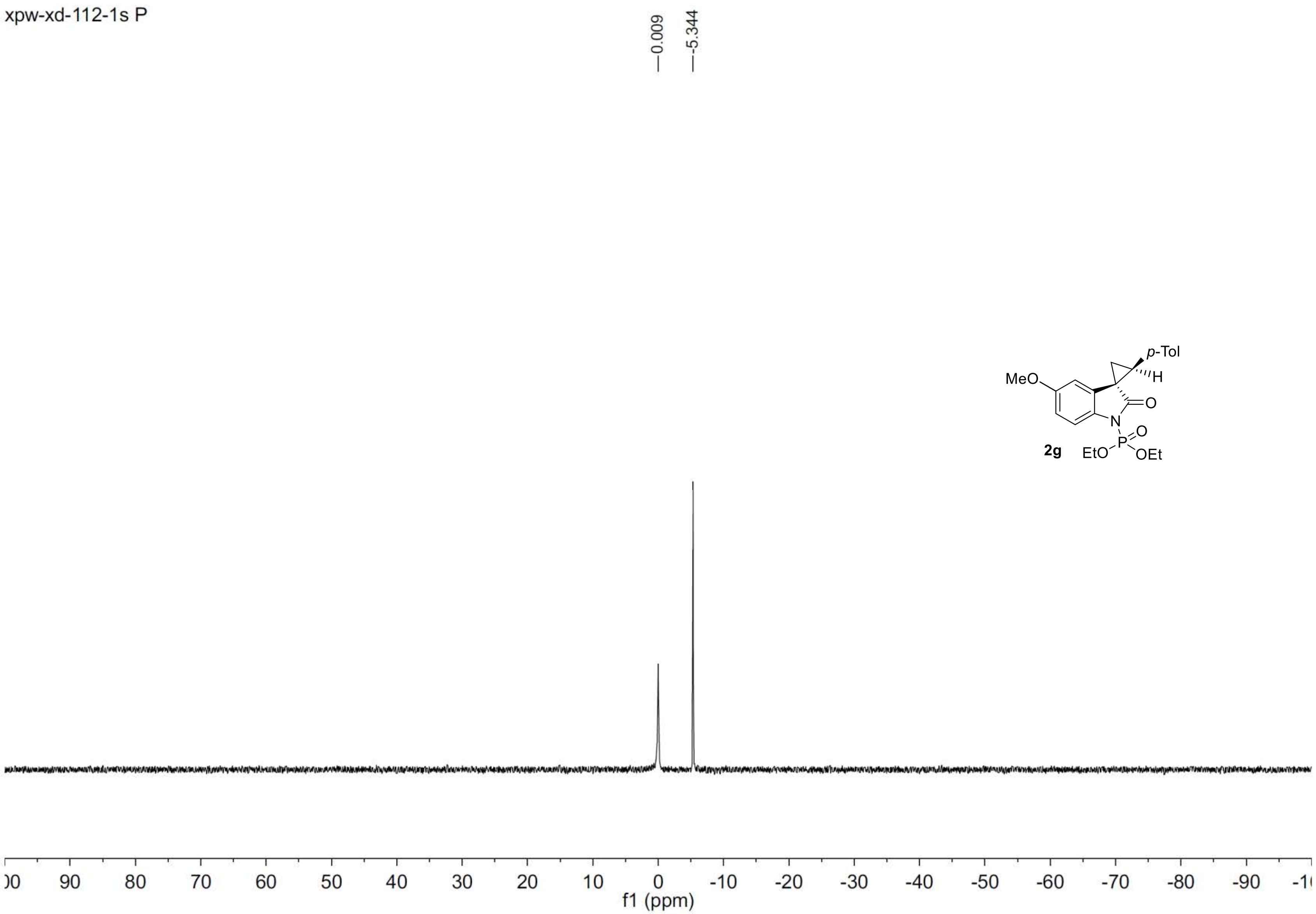

Supplementary Figure 102. ^{31}P NMR (122 MHz, $CDCl_3$) spectra for compound **2g**

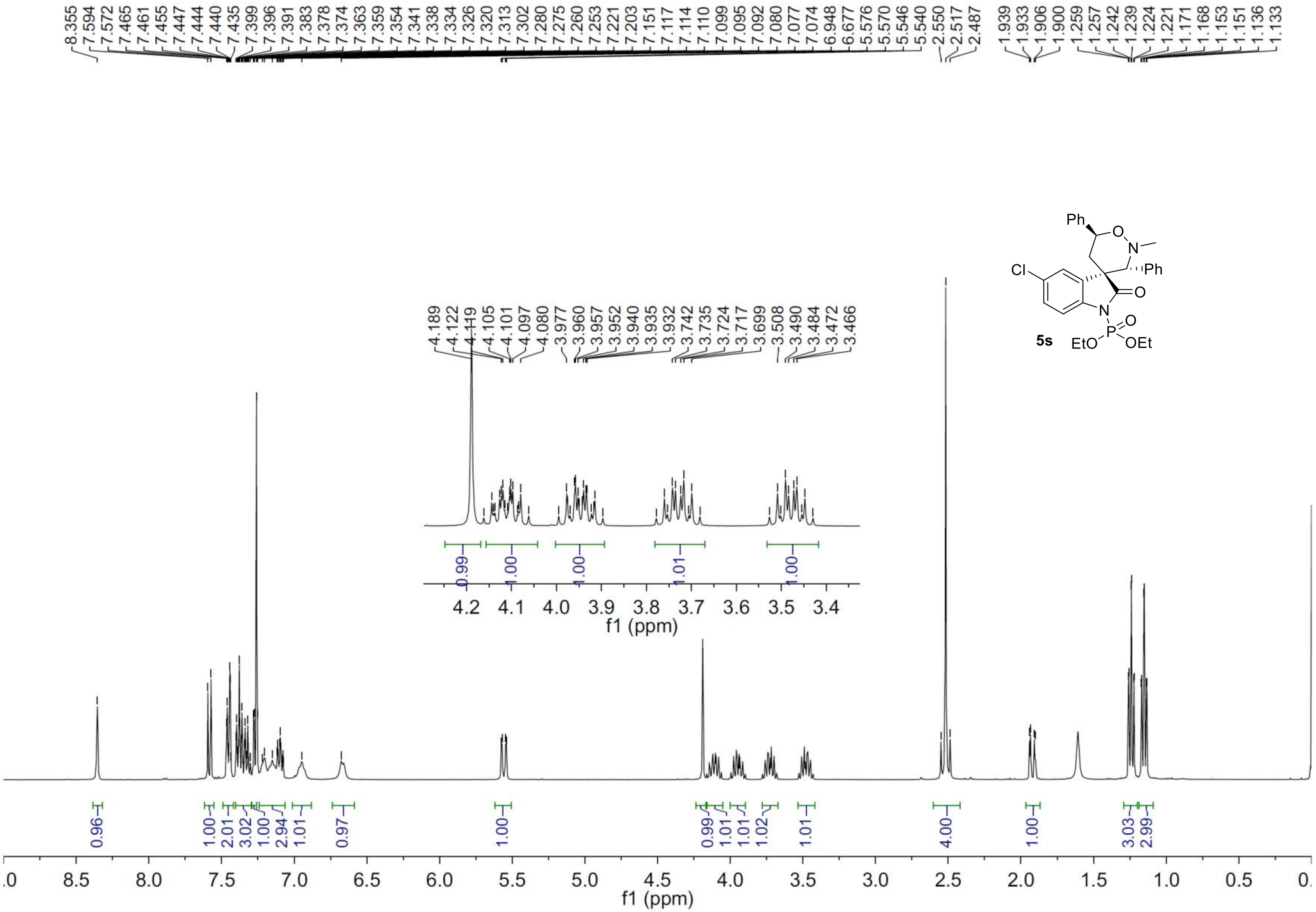

Supplementary Figure 103. ^{1}H NMR (400 MHz, $CDCl_3$) spectra for compound **5s**

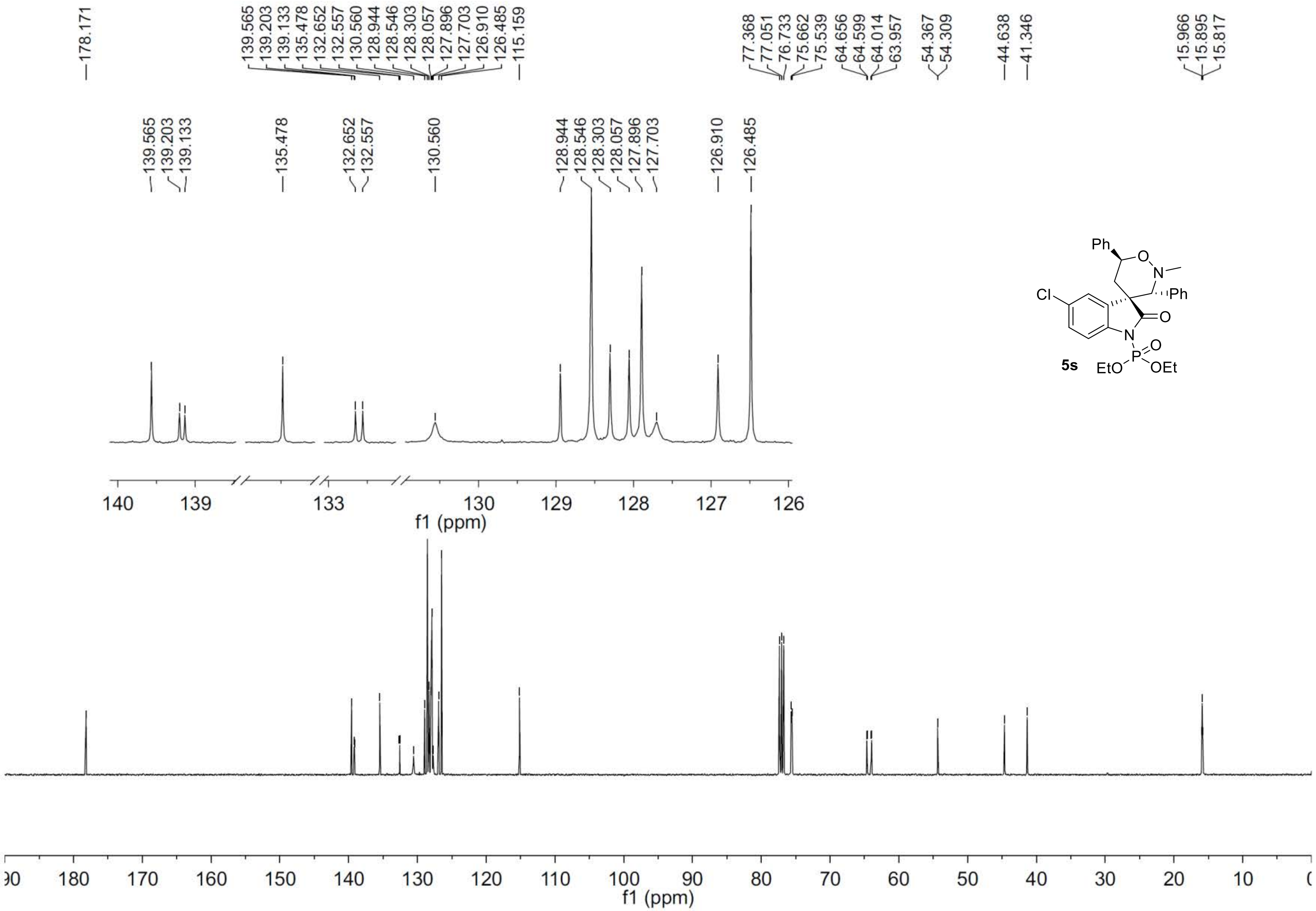

Supplementary Figure 104. ^{13}C NMR (100 MHz, $CDCl_3$) spectra for compound **5s**

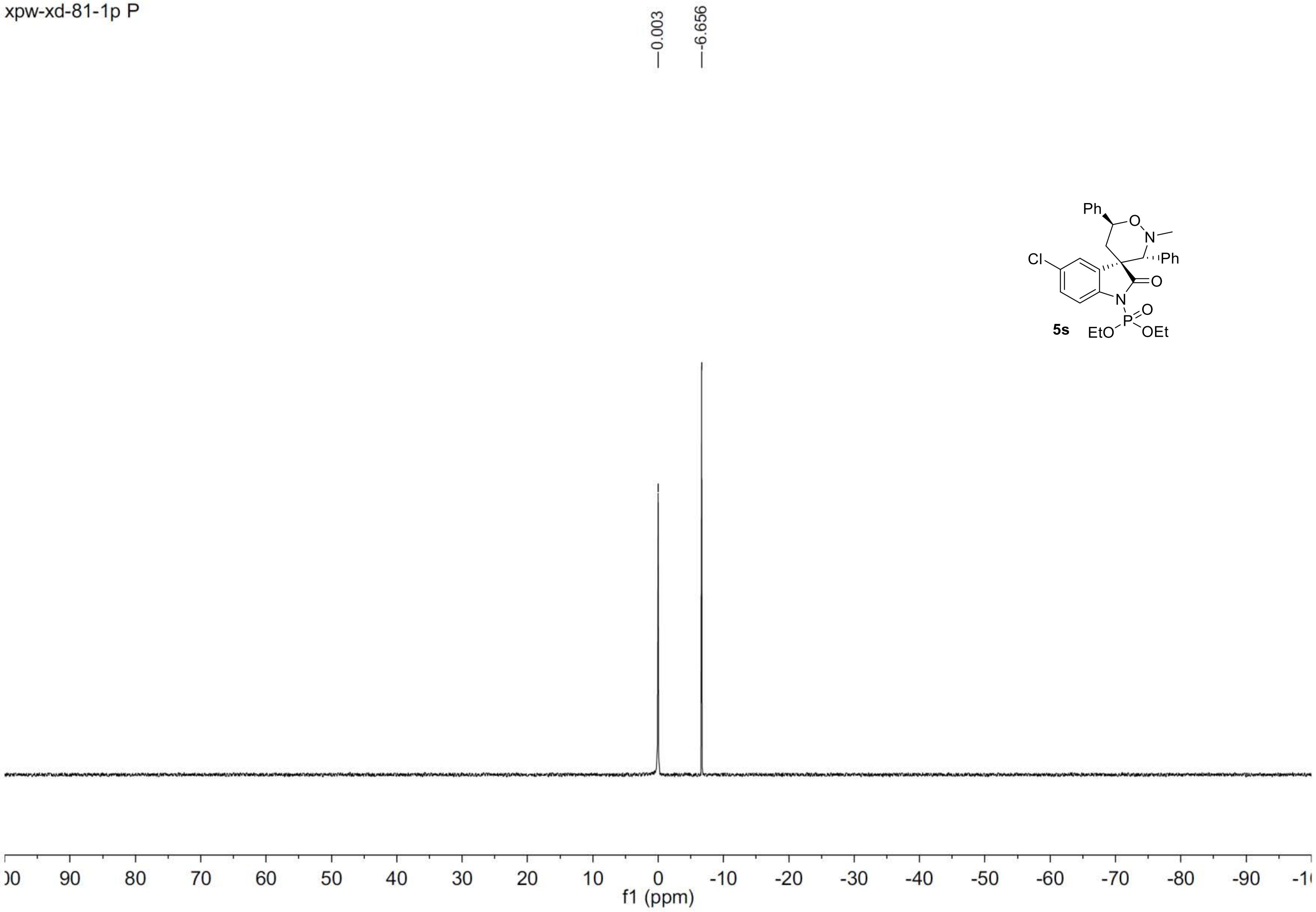

Supplementary Figure 105. ^{31}P NMR (162 MHz, $CDCl_3$) spectra for compound **5s**

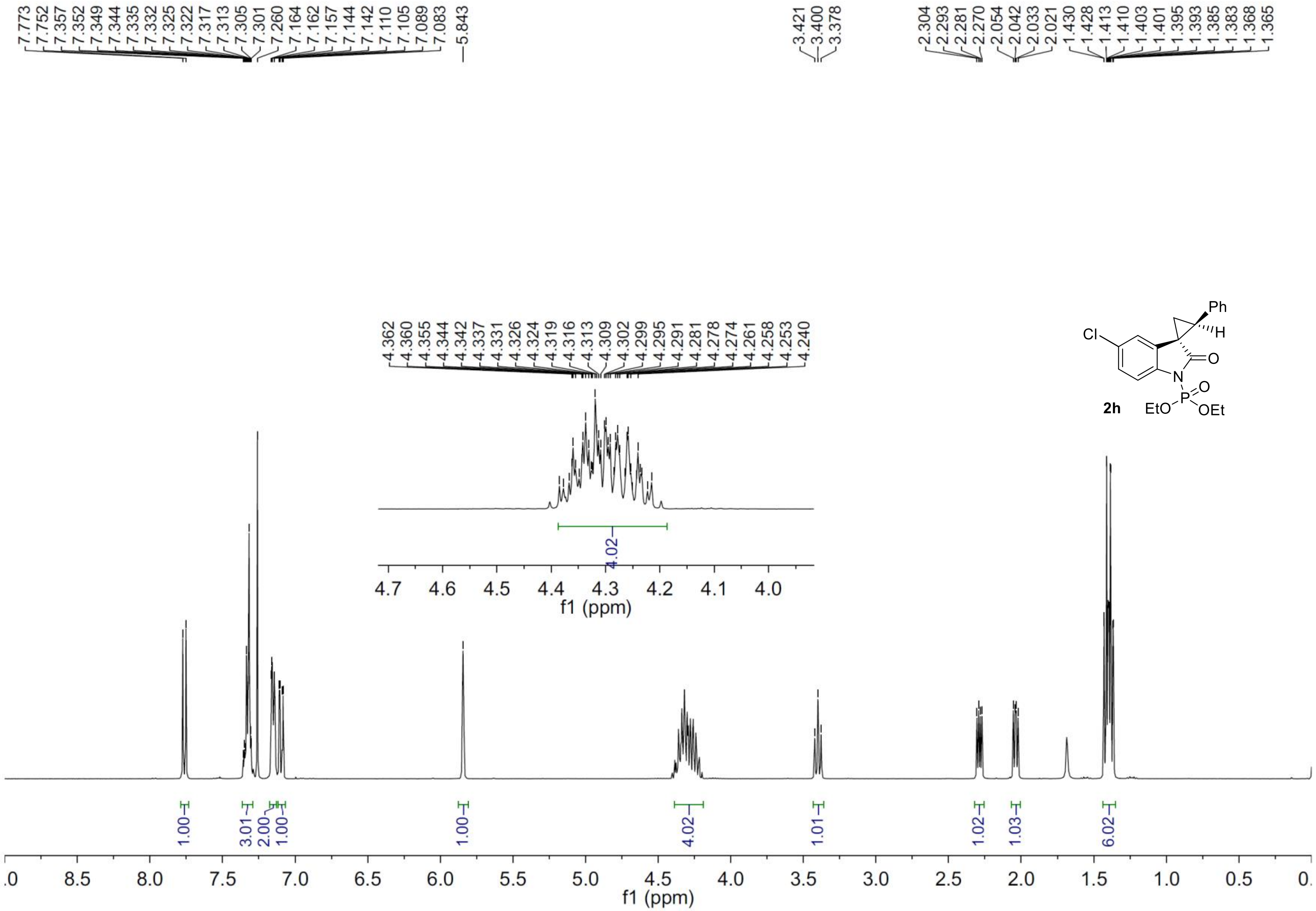

Supplementary Figure 106. ^{1}H NMR (400 MHz, $CDCl_3$) spectra for compound **2h**

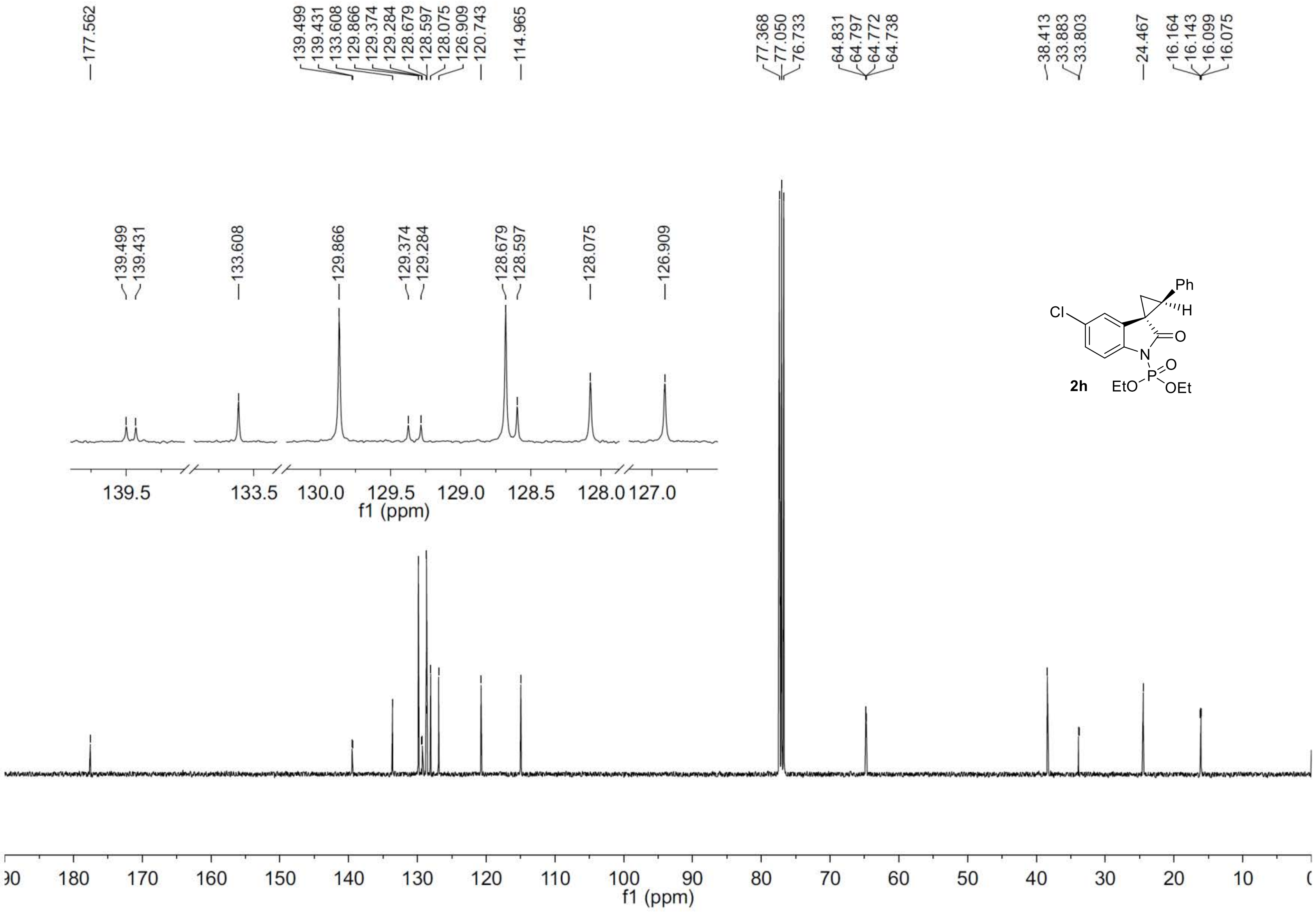

Supplementary Figure 107. ^{13}C NMR (100 MHz, $CDCl_3$) spectra for compound **2h**

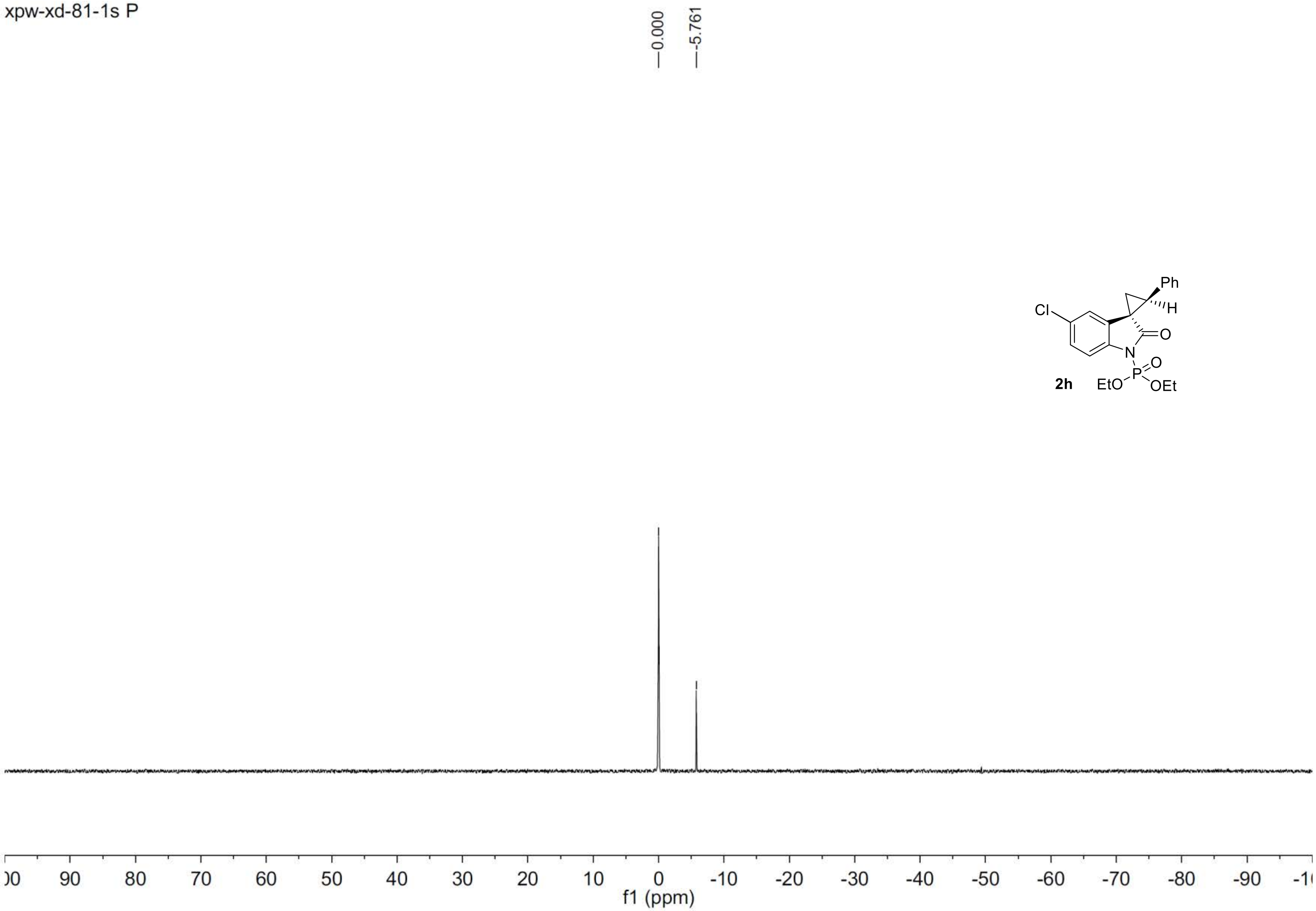

Supplementary Figure 108. ^{31}P NMR (122 MHz, $CDCl_3$) spectra for compound **2h**

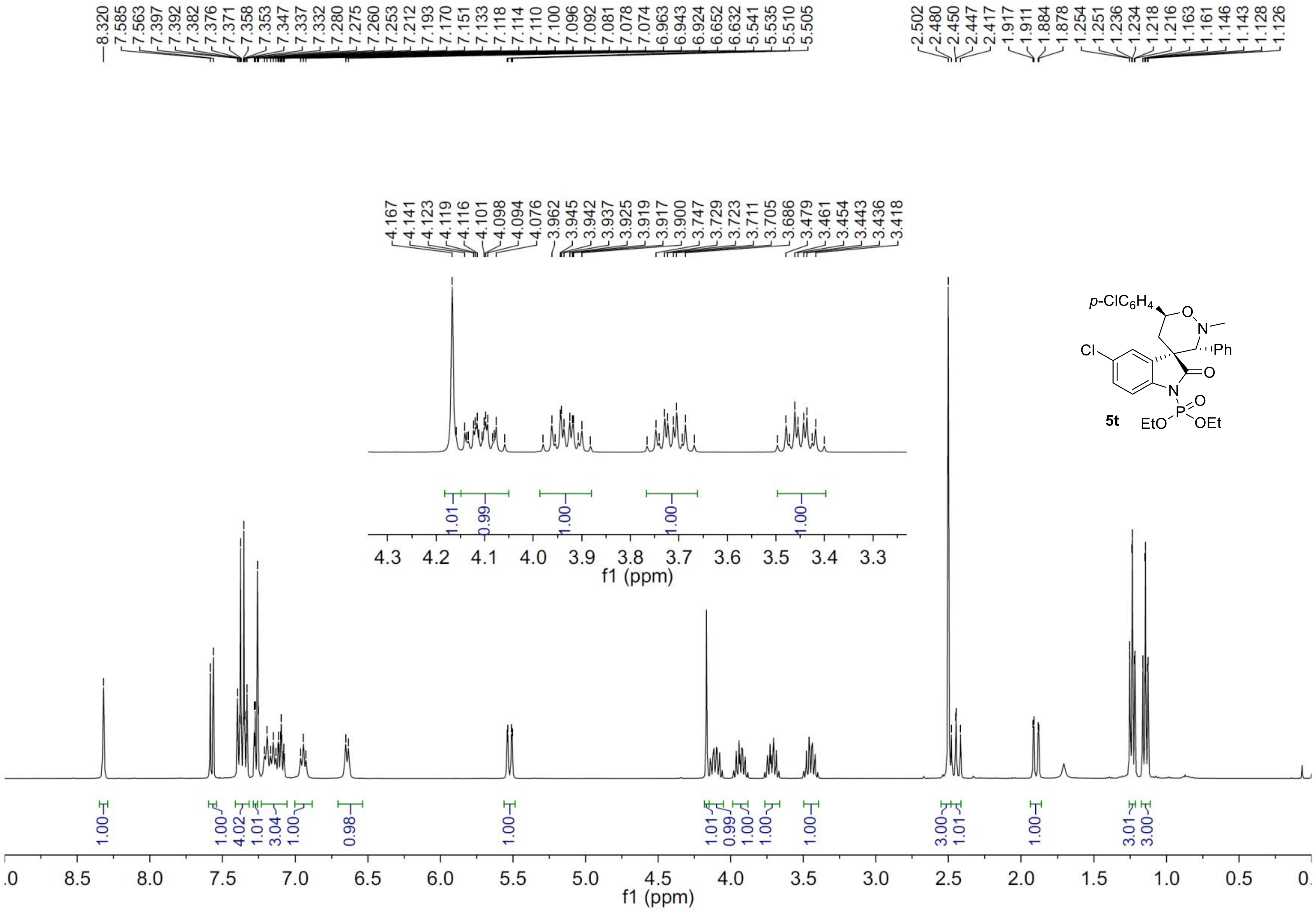

Supplementary Figure 109. ^{1}H NMR (400 MHz, $CDCl_3$) spectra for compound **5t**

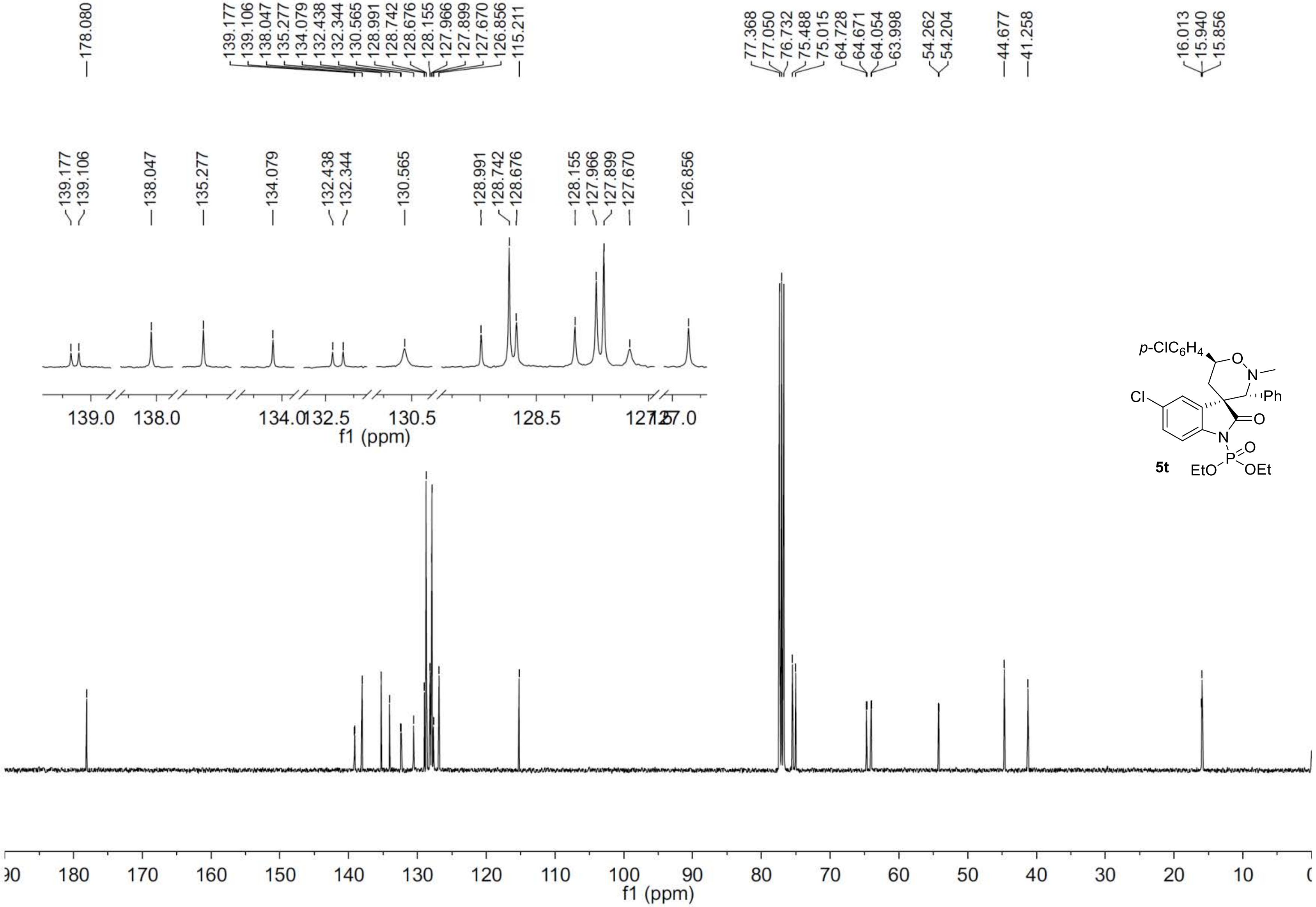

Supplementary Figure 110. ^{13}C NMR (100 MHz, $CDCl_3$) spectra for compound **5t**

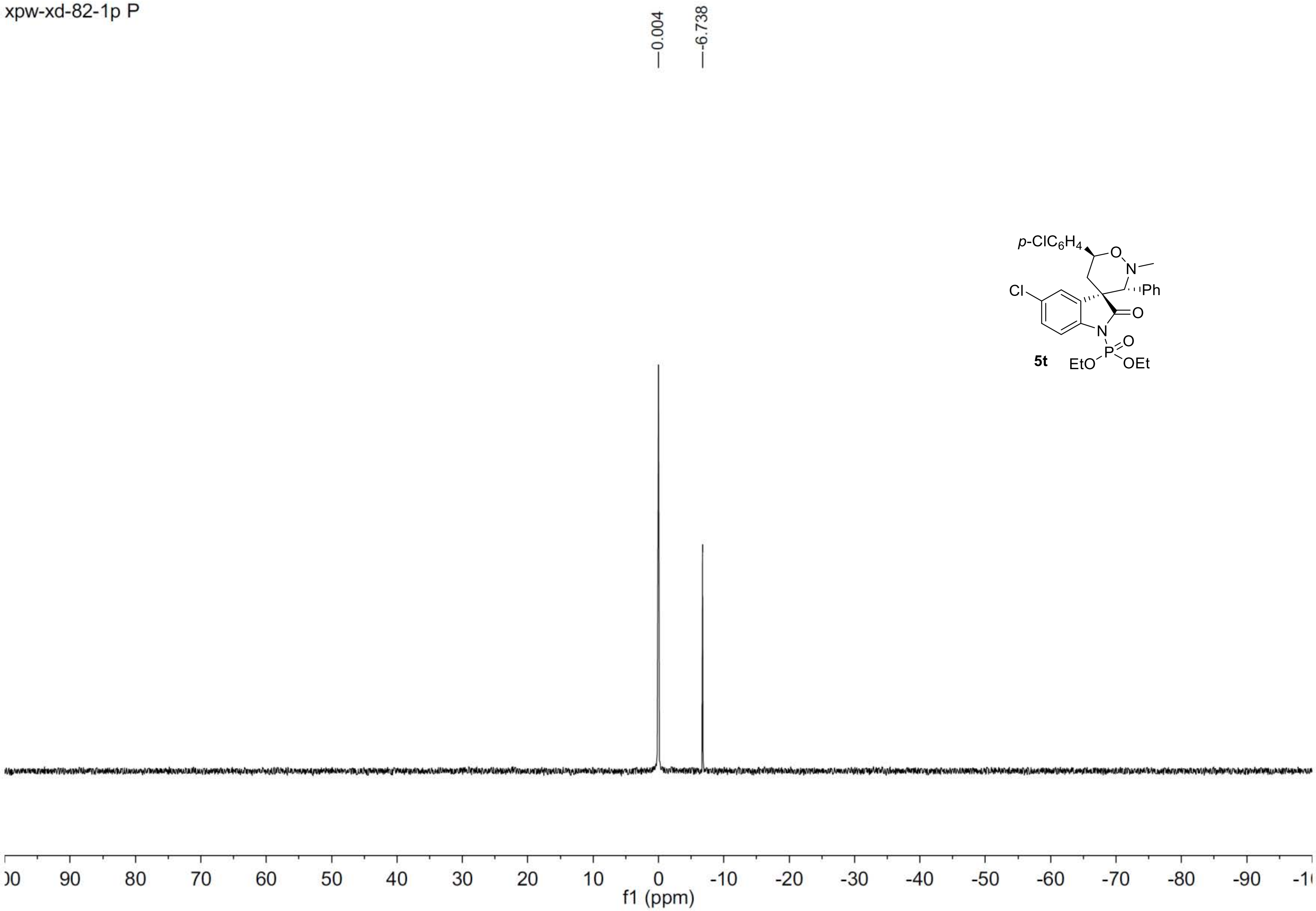

Supplementary Figure 111. ^{31}P NMR (122 MHz, $CDCl_3$) spectra for compound **5t**

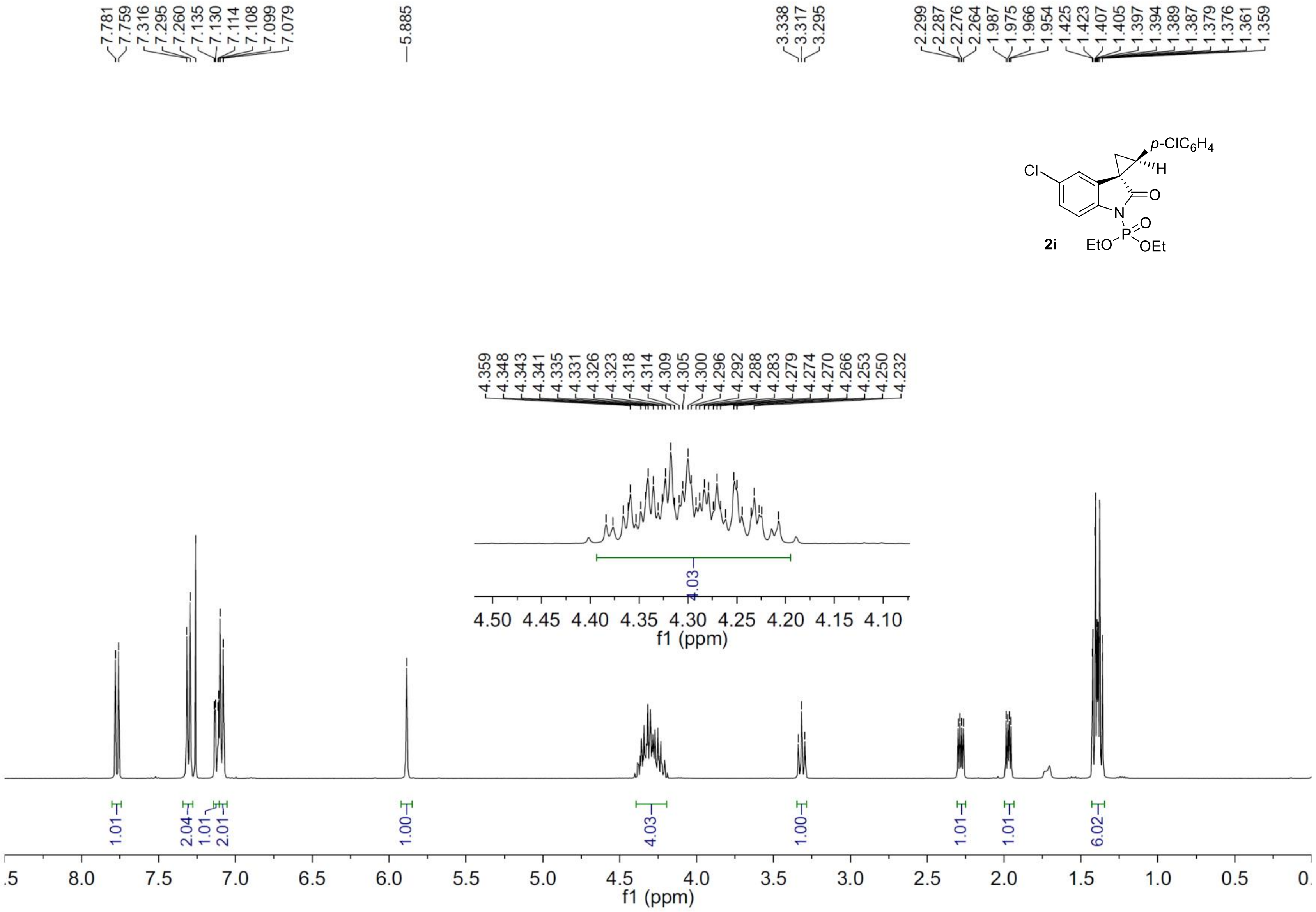

Supplementary Figure 112. ^{1}H NMR (400 MHz, $CDCl_3$) spectra for compound **2i**

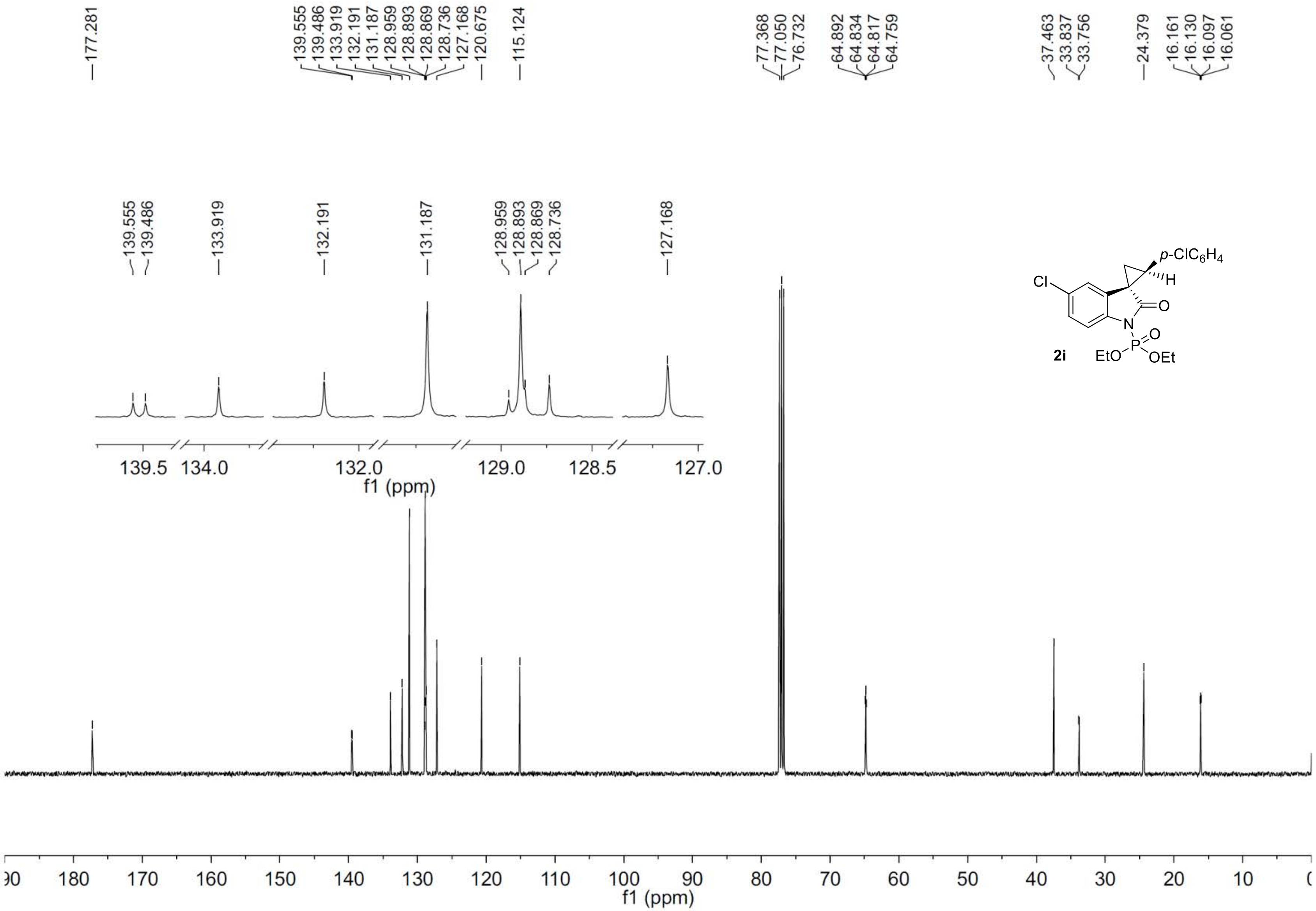

Supplementary Figure 113. ^{13}C NMR (100 MHz, $CDCl_3$) spectra for compound **2i**

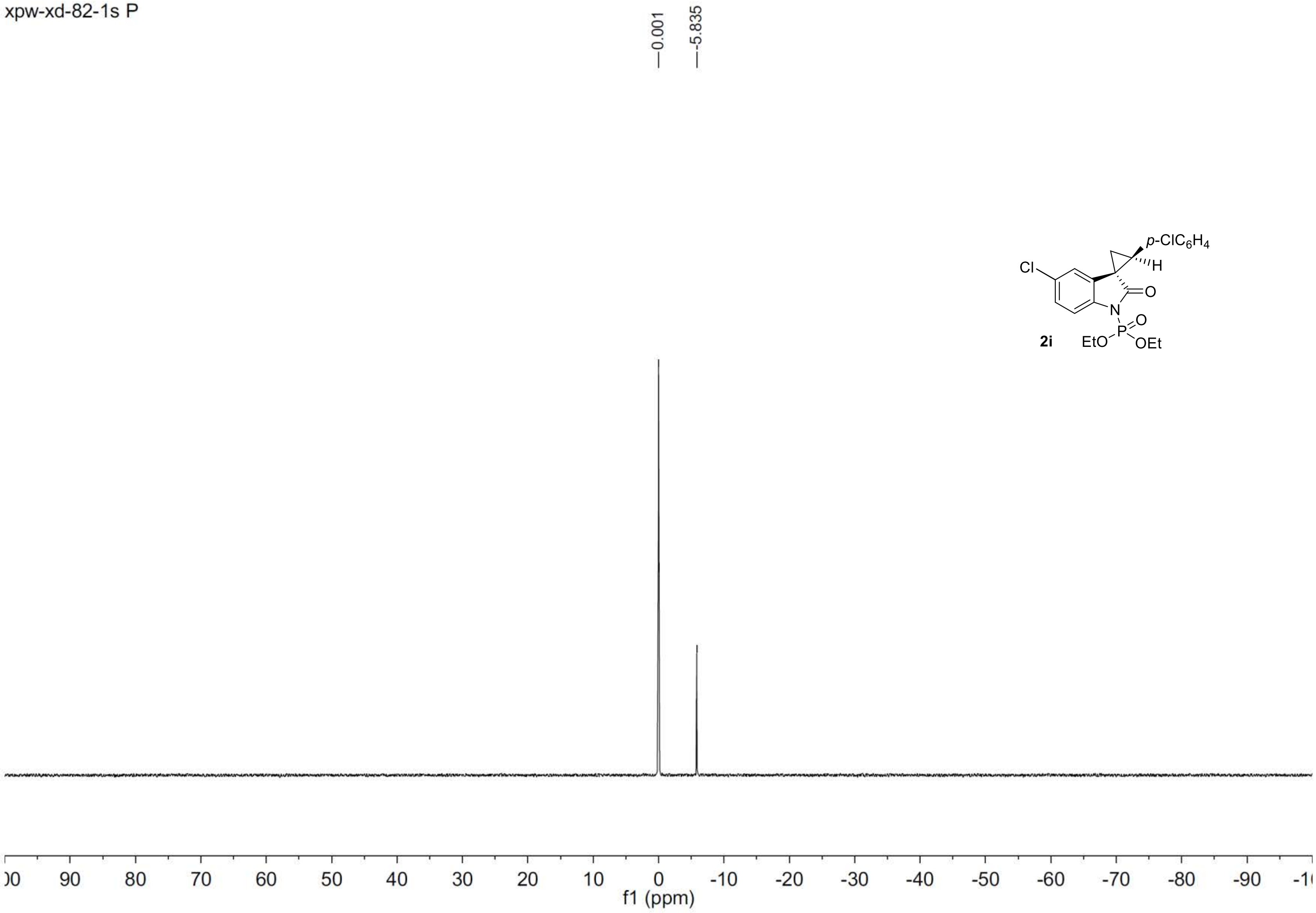

Supplementary Figure 114 ^{31}P NMR (122 MHz, $CDCl_3$) spectra for compound **2i**

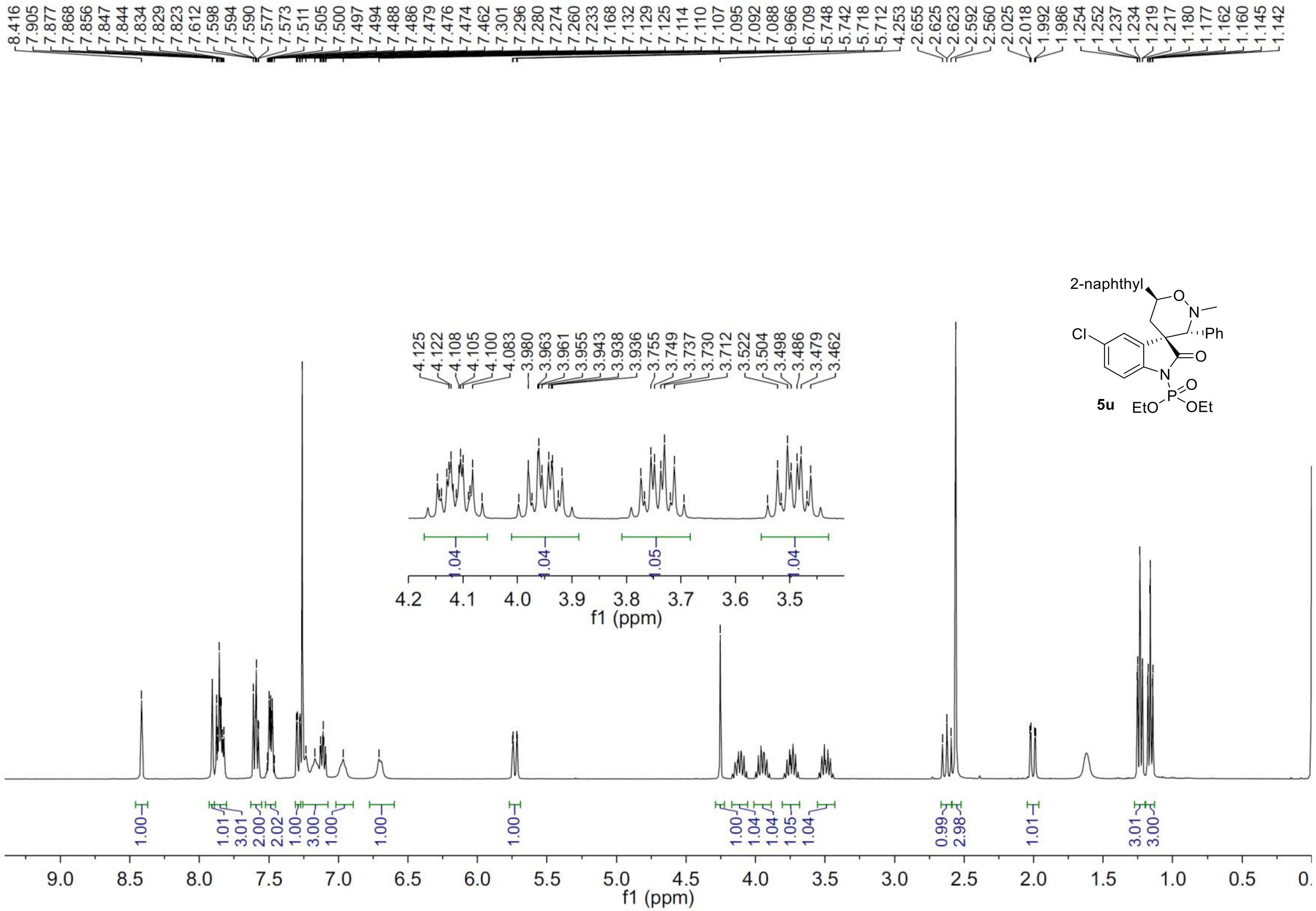

Supplementary Figure 115. ^{1}H NMR (400 MHz, $CDCl_3$) spectra for compound **5u**

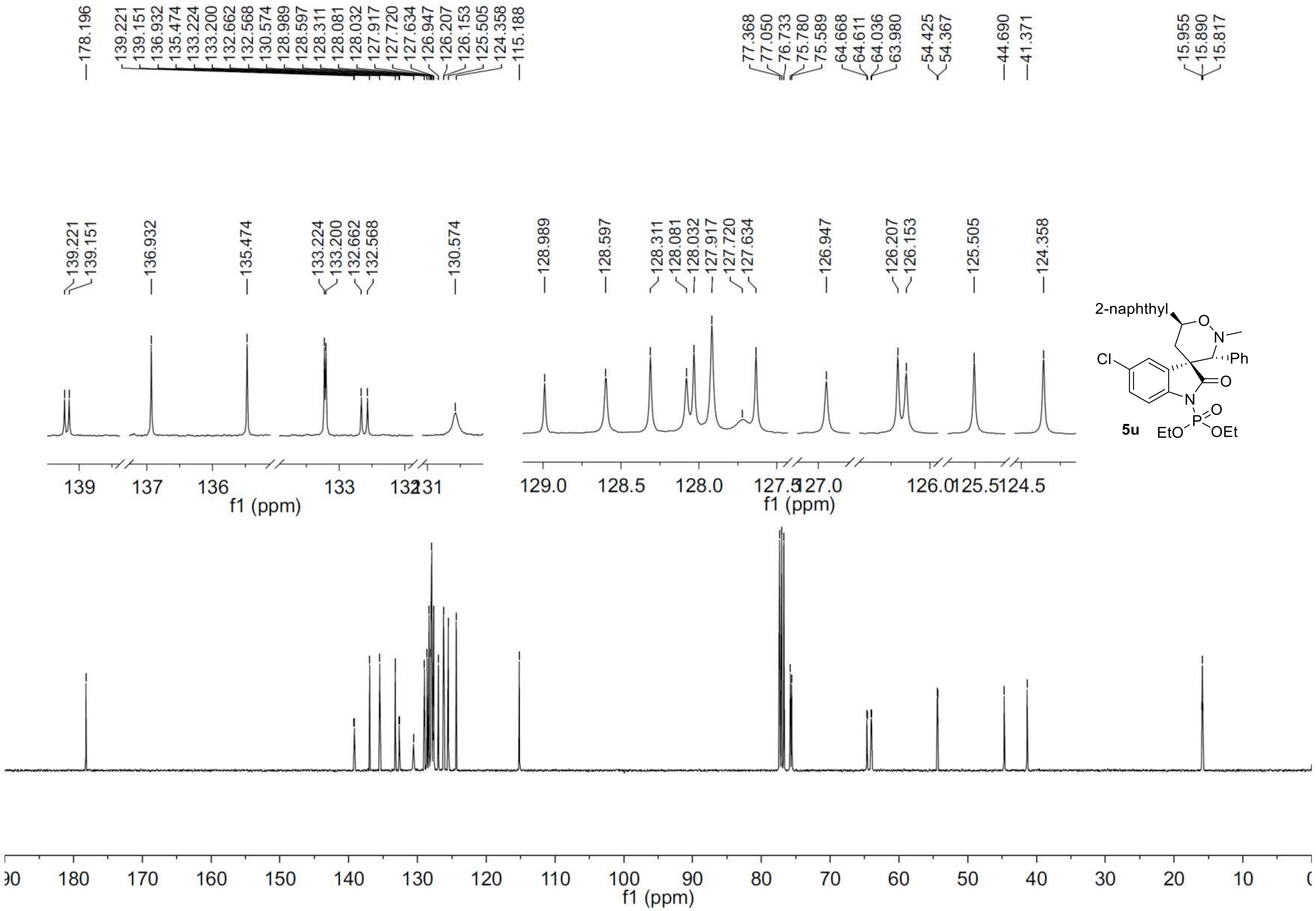

Supplementary Figure 116. ^{13}C NMR (100 MHz, $CDCl_3$) spectra for compound **5u**

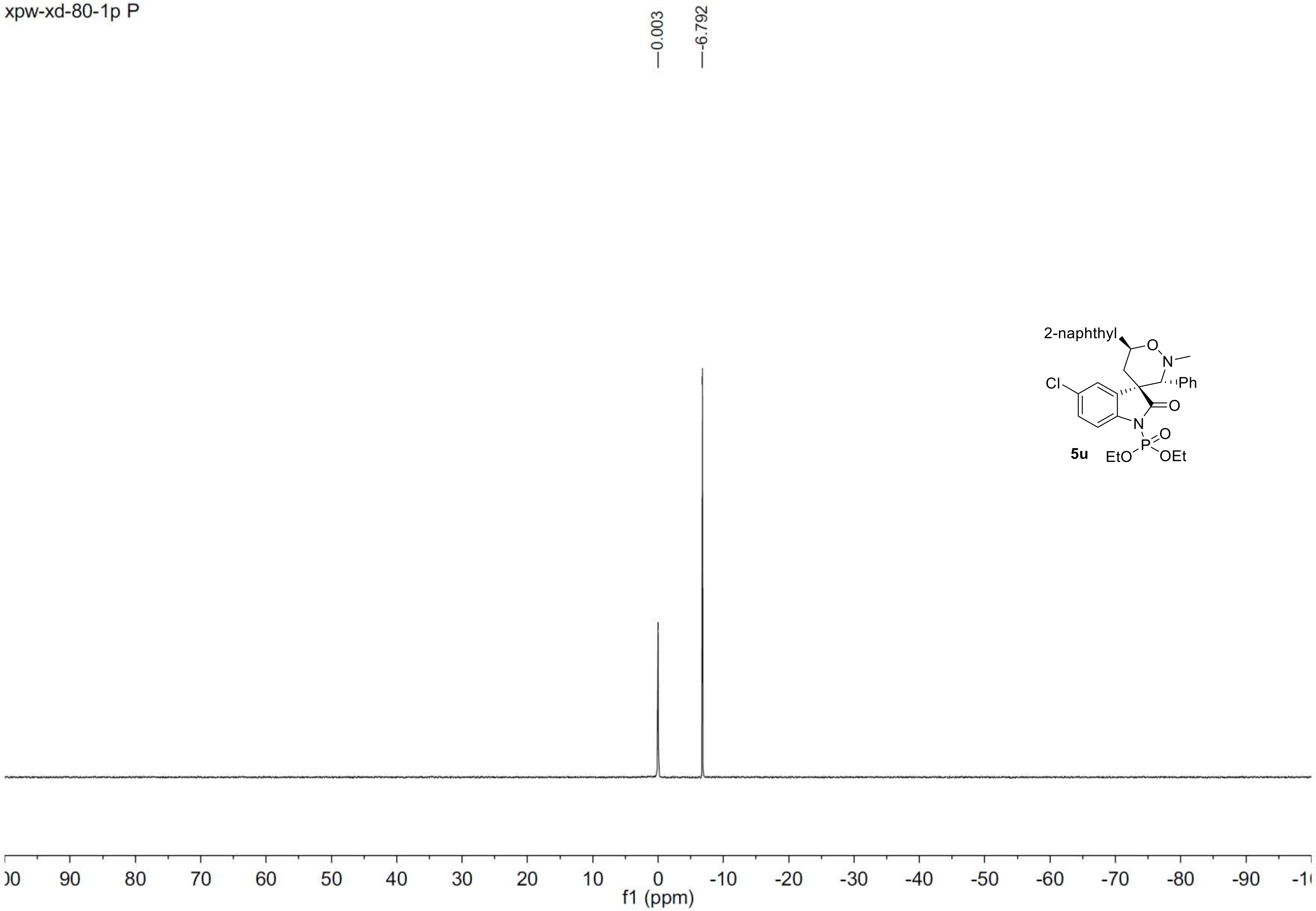

Supplementary Figure 117. ^{31}P NMR (122 MHz, $CDCl_3$) spectra for compound **5u**

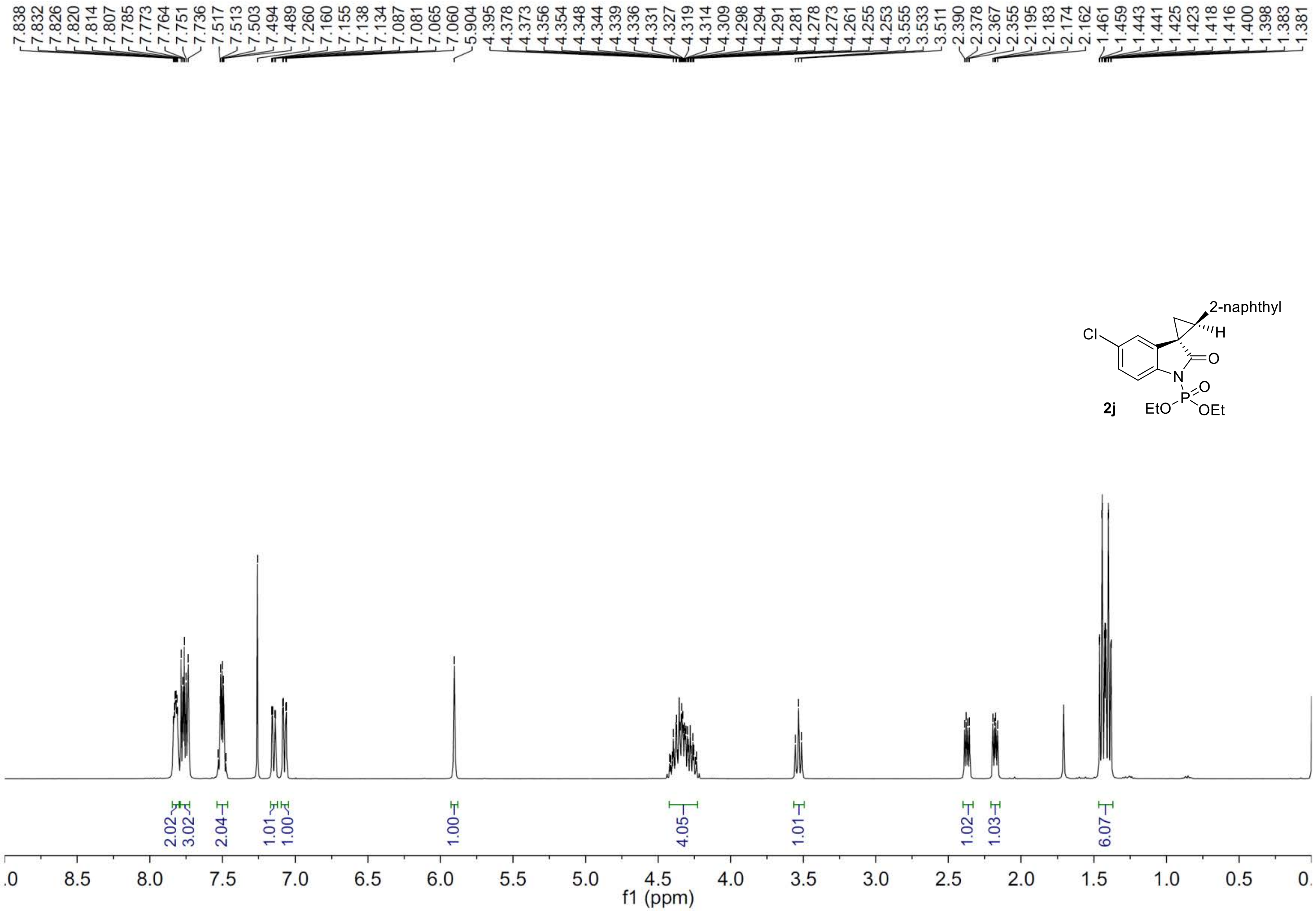

Supplementary Figure 118. ^{1}H NMR (400 MHz, $CDCl_3$) spectra for compound **2j**

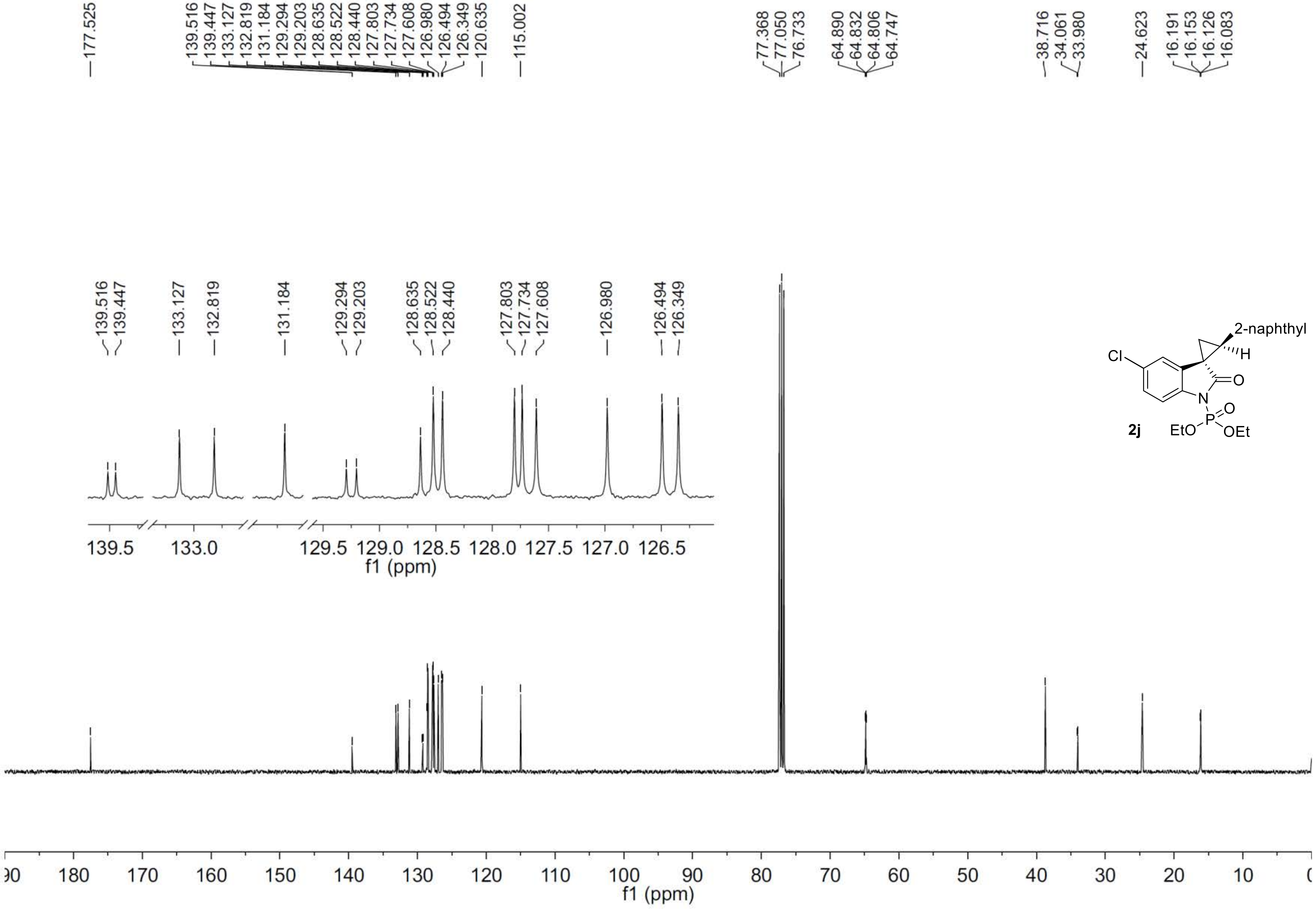

Supplementary Figure 119. ^{13}C NMR (100 MHz, $CDCl_3$) spectra for compound **2j**

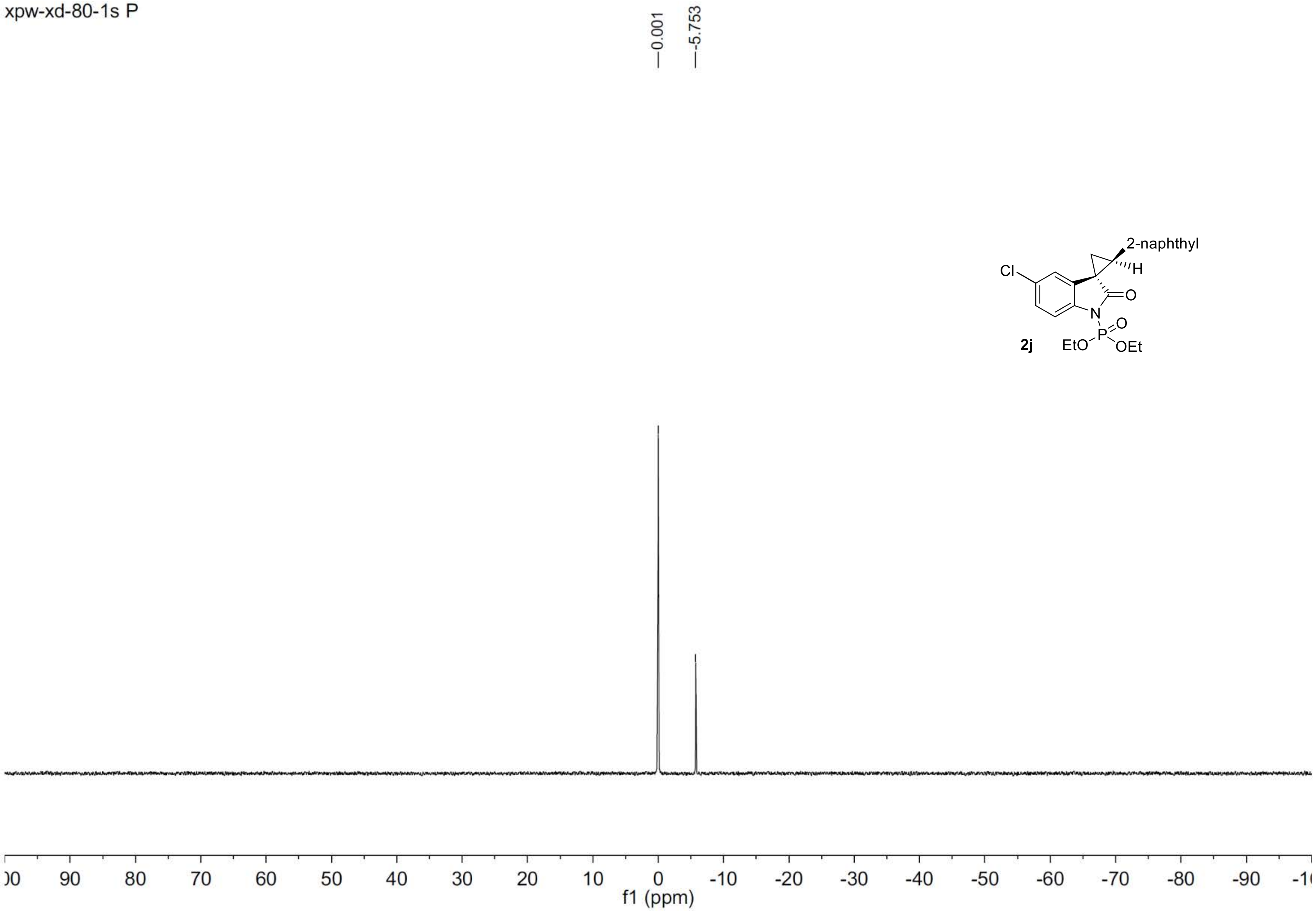

Supplementary Figure 120. ^{31}P NMR (122 MHz, $CDCl_3$) spectra for compound **2j**

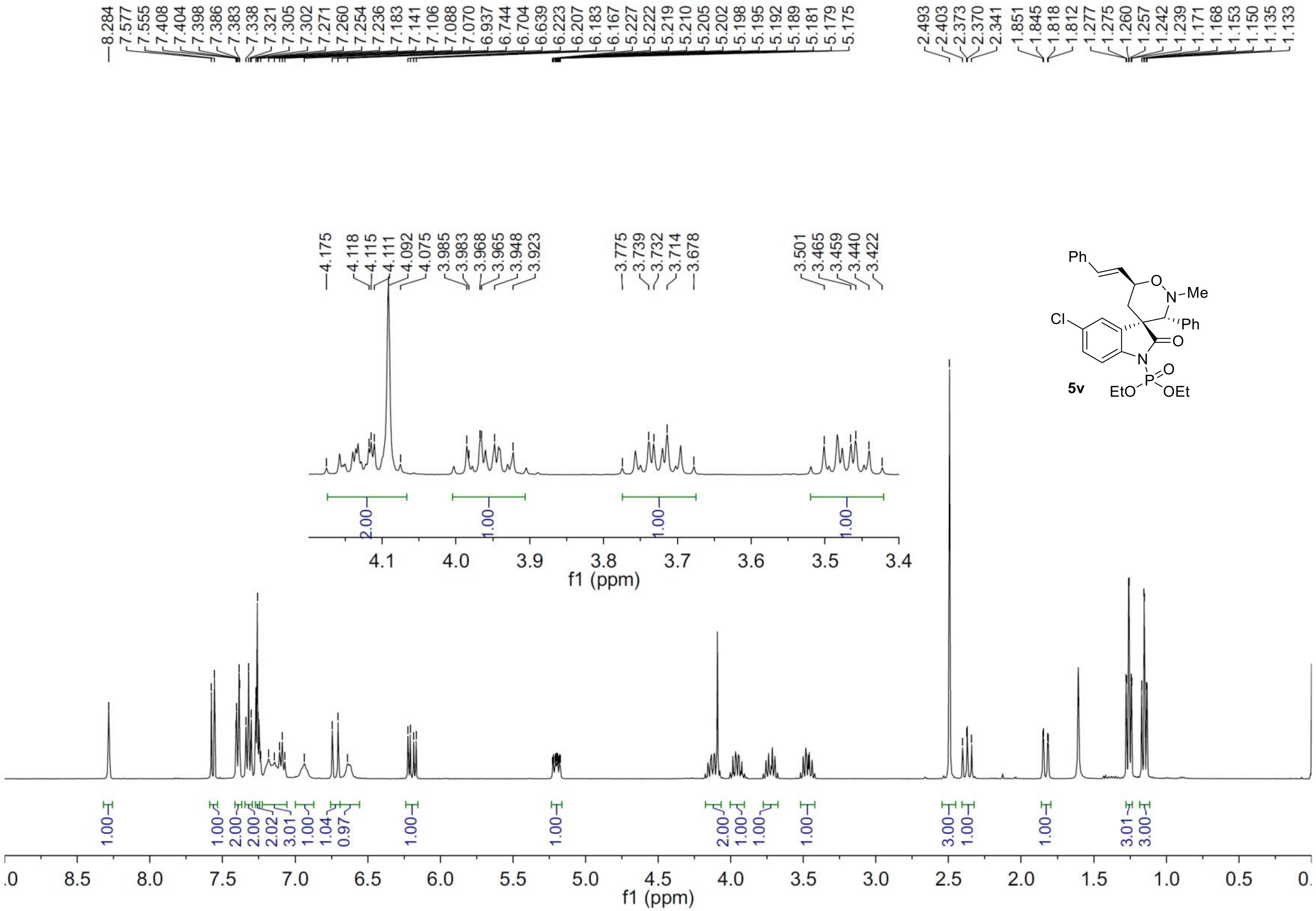

Supplementary Figure 121. ^{1}H NMR (400 MHz, $CDCl_3$) spectra for compound **5v**

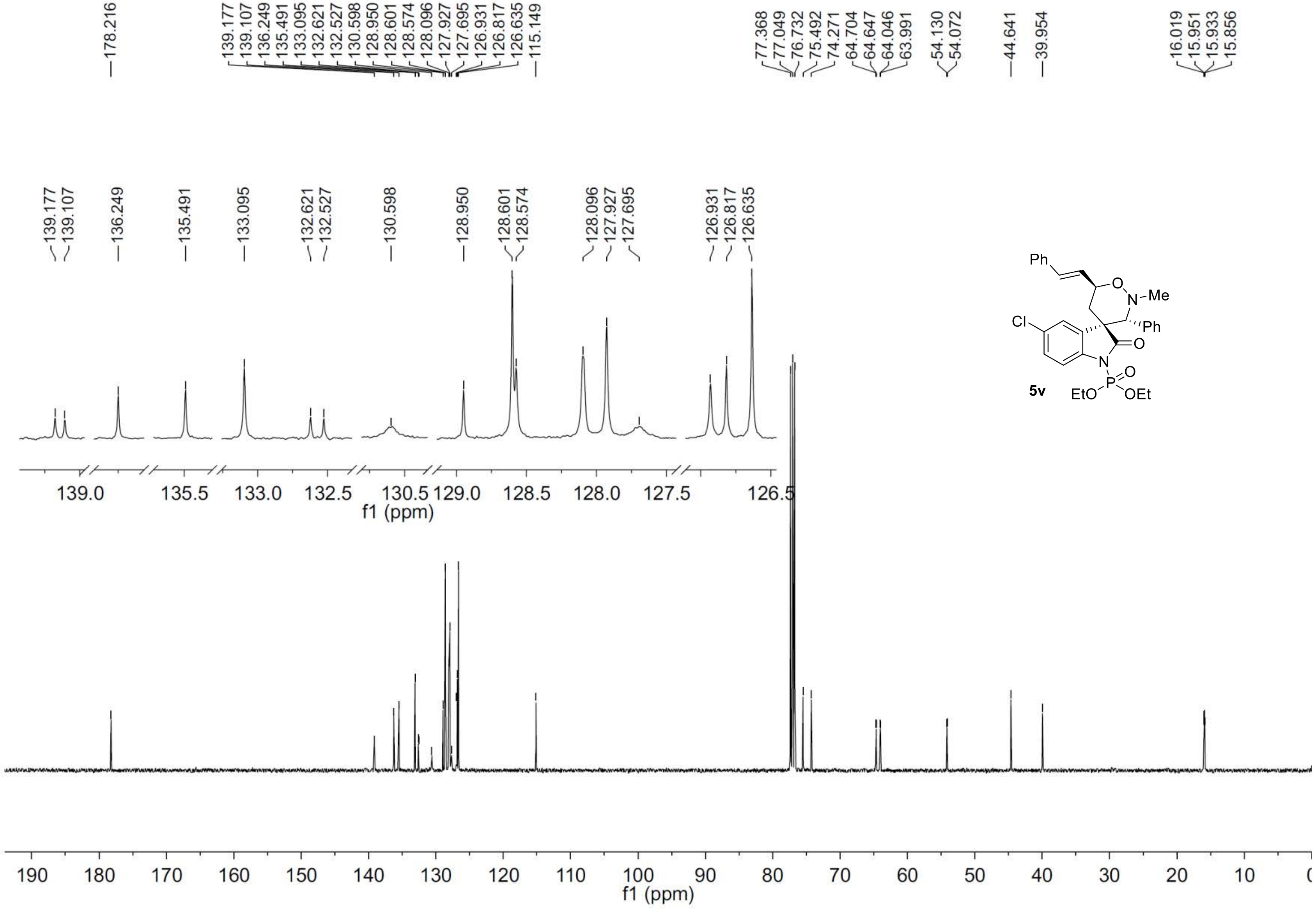

Supplementary Figure 122. ^{13}C NMR (100 MHz, $CDCl_3$) spectra for compound **5v**

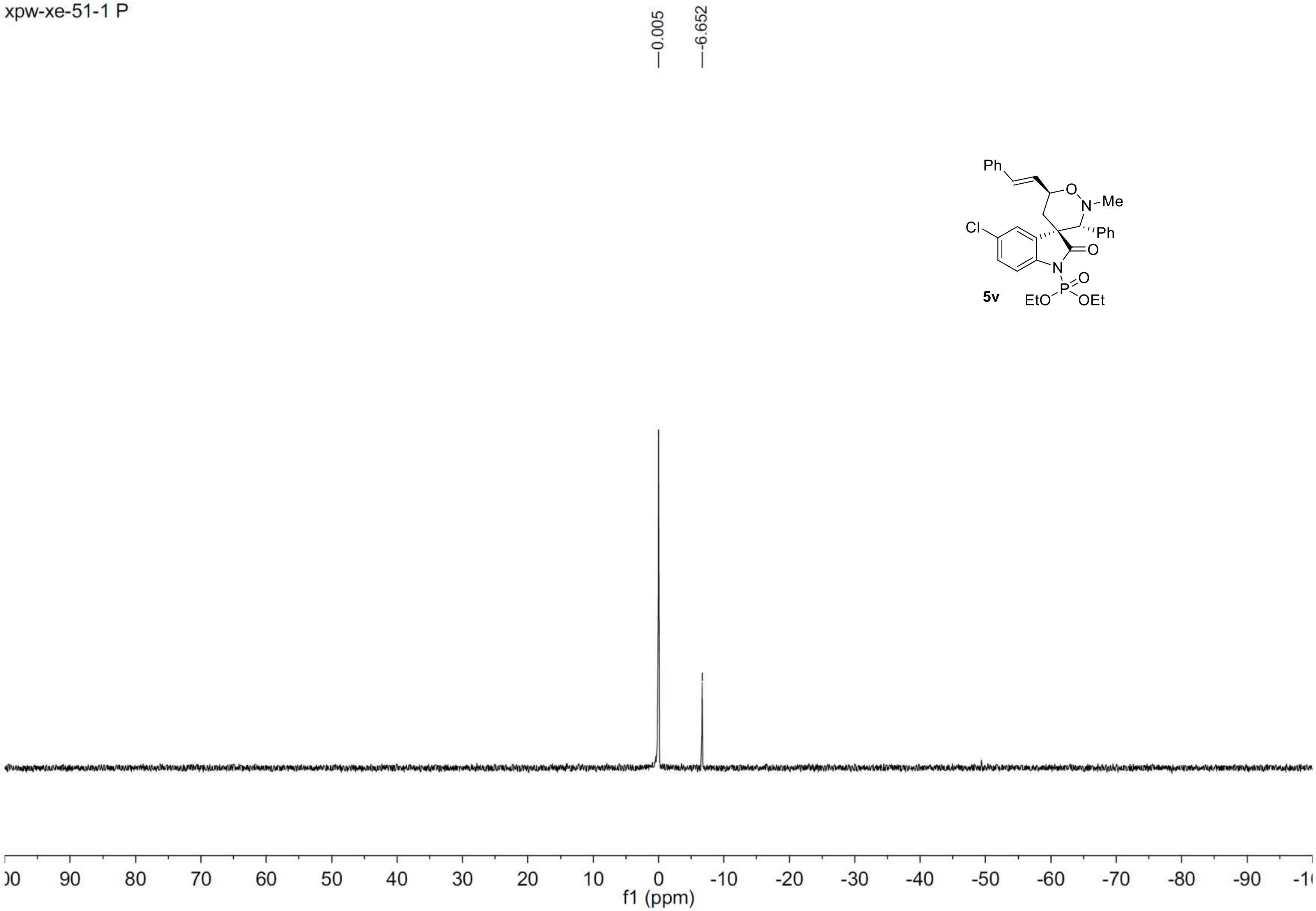

5v

Supplementary Figure 123. ^{31}P NMR (122 MHz, $CDCl_3$) spectra for compound **5v**

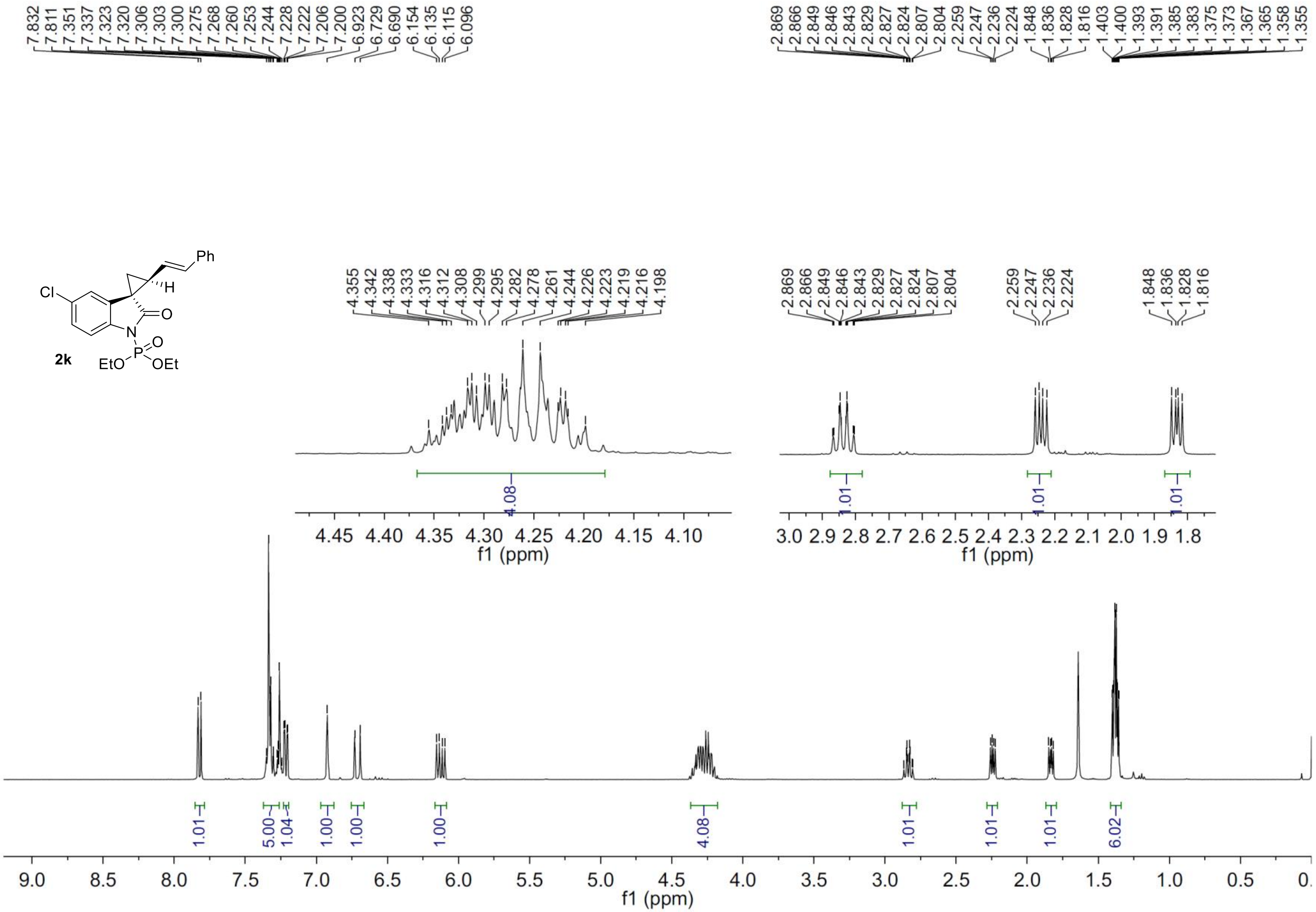

Supplementary Figure 124. ^{1}H NMR (400 MHz, $CDCl_3$) spectra for compound **2k**

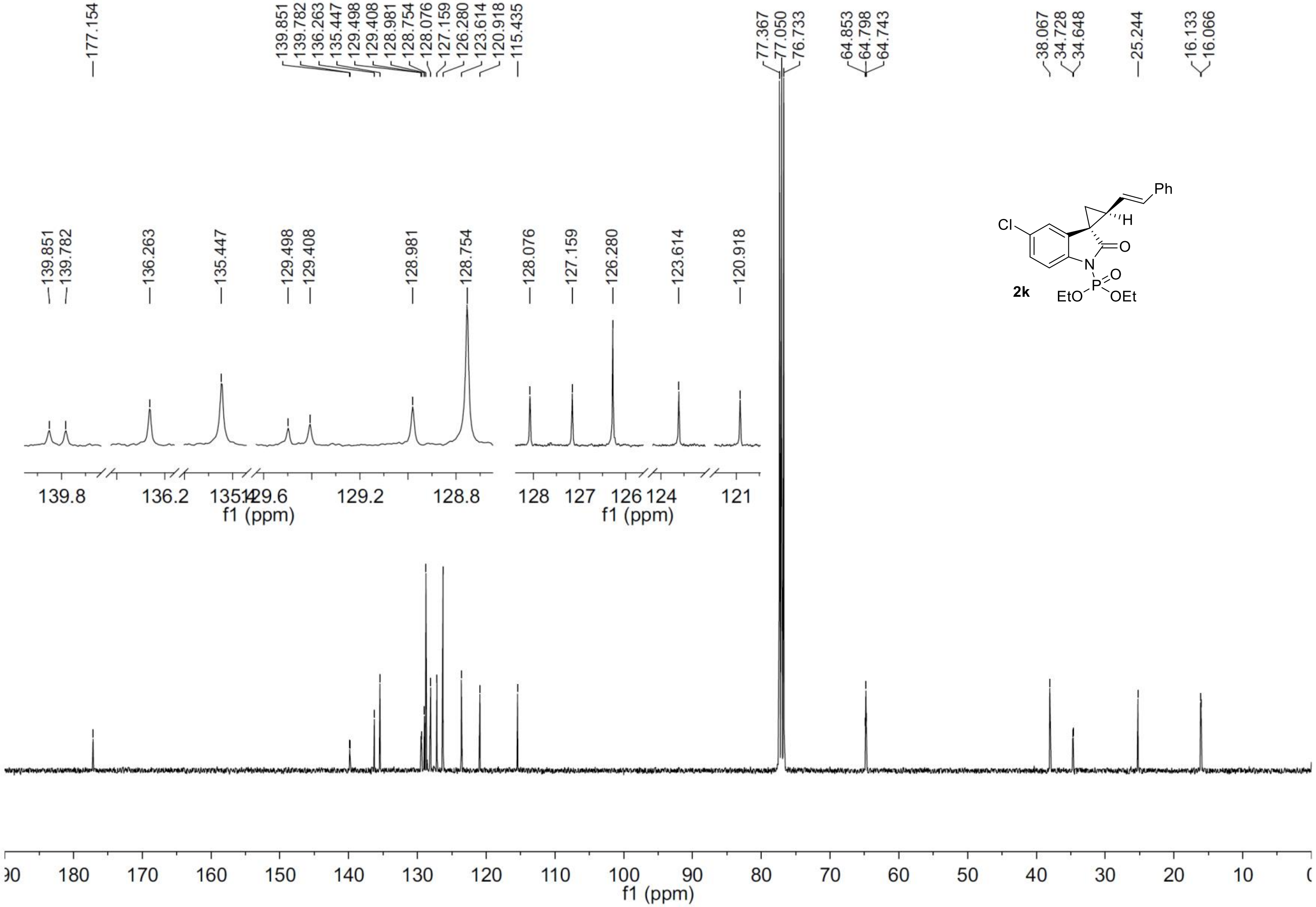

Supplementary Figure 125. ^{13}C NMR (100 MHz, $CDCl_3$) spectra for compound **2k**

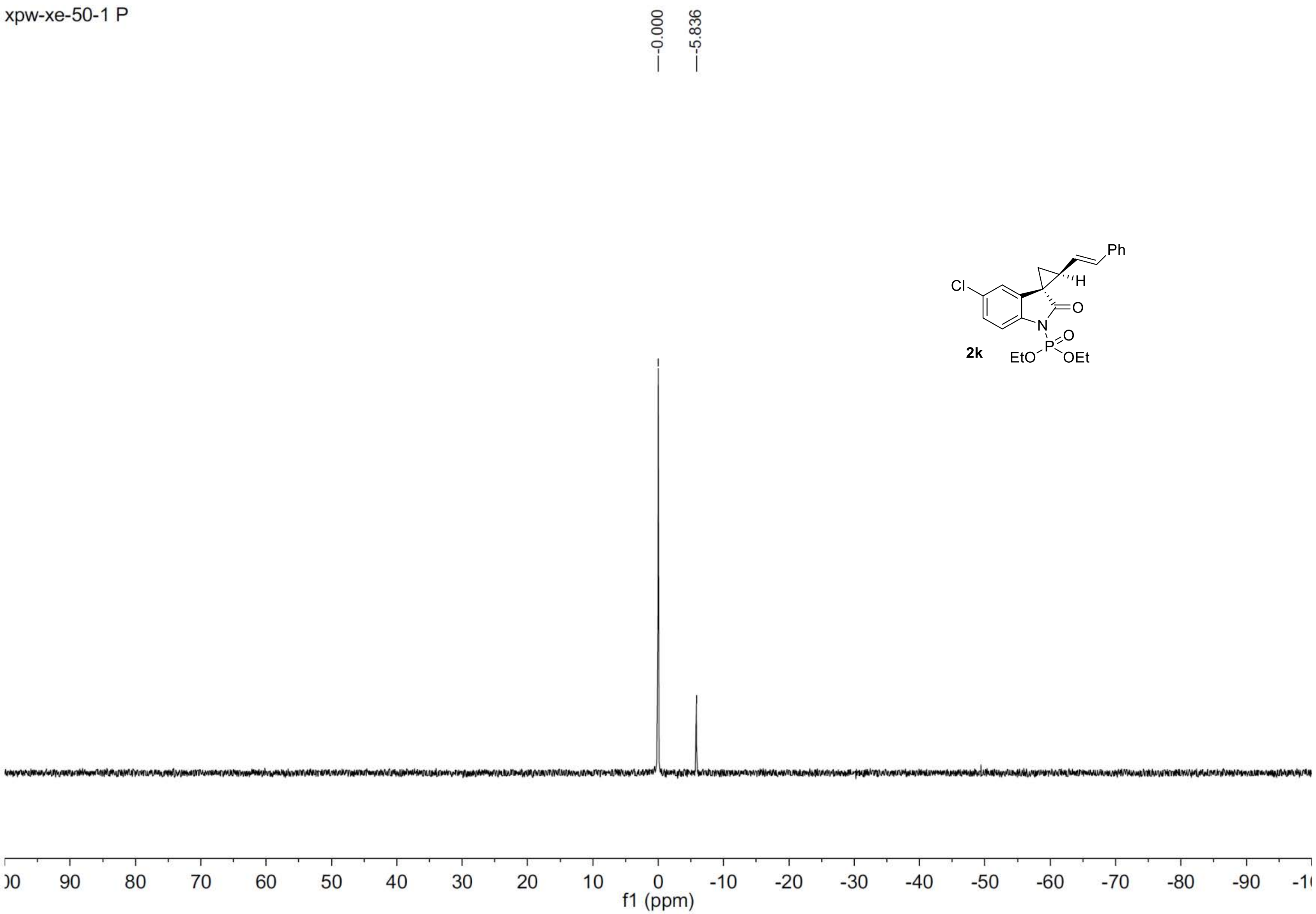

Supplementary Figure 126. ^{31}P NMR (122 MHz, $CDCl_3$) spectra for compound **2k**

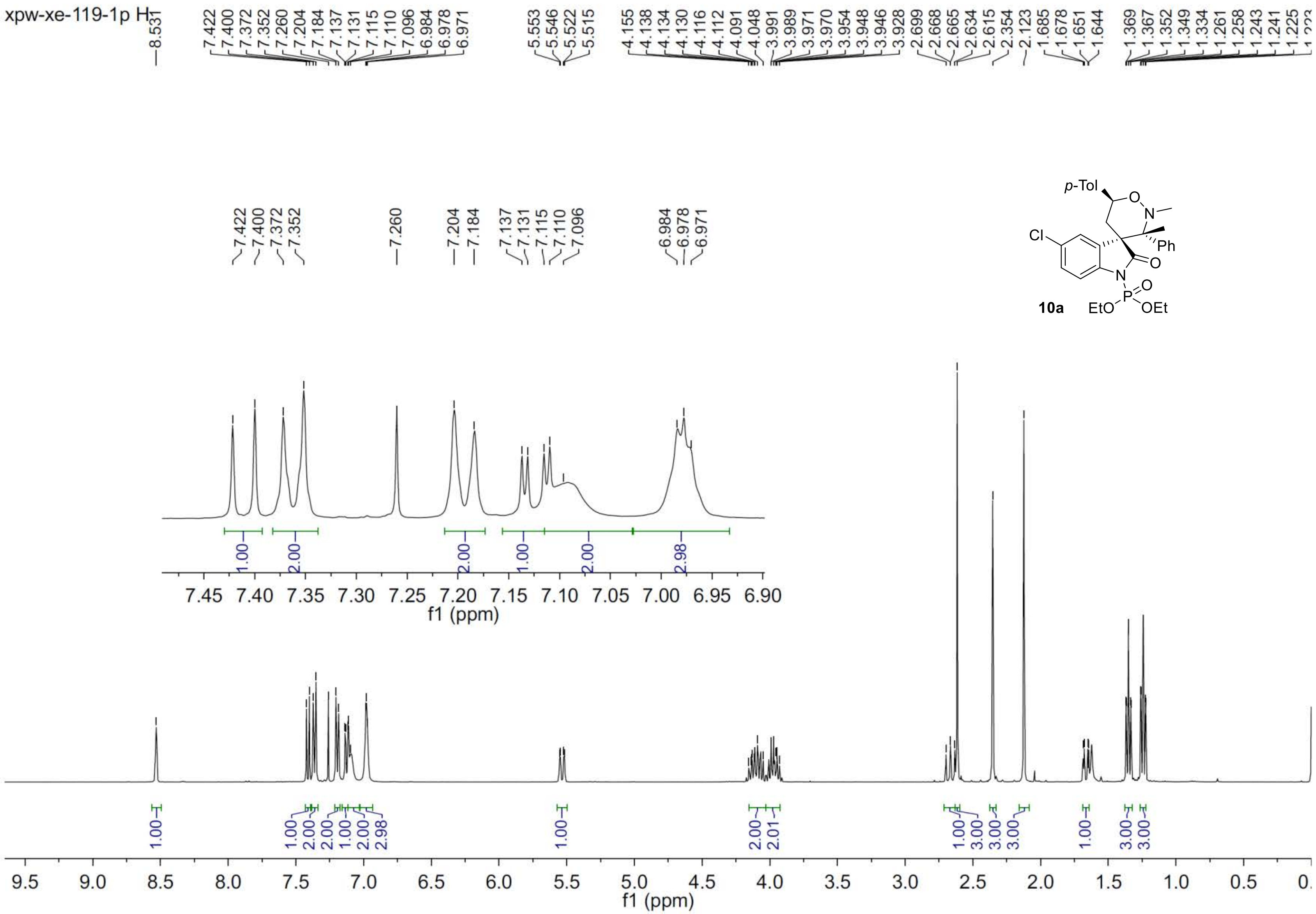

Supplementary Figure 127. ^{1}H NMR (400 MHz, $CDCl_3$) spectra for compound **10a**

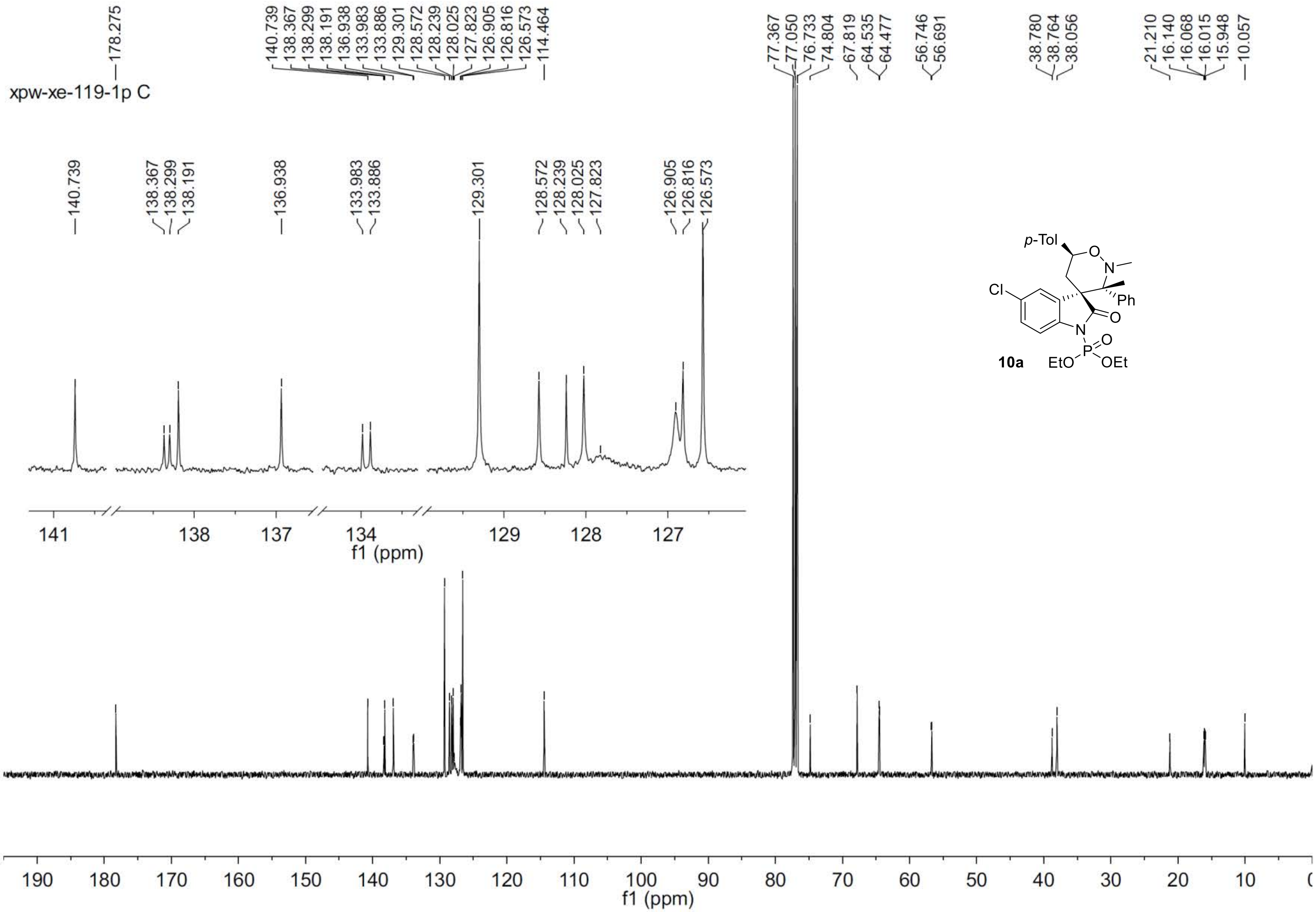

Supplementary Figure 128. ^{13}C NMR (100 MHz, $CDCl_3$) spectra for compound **10a**

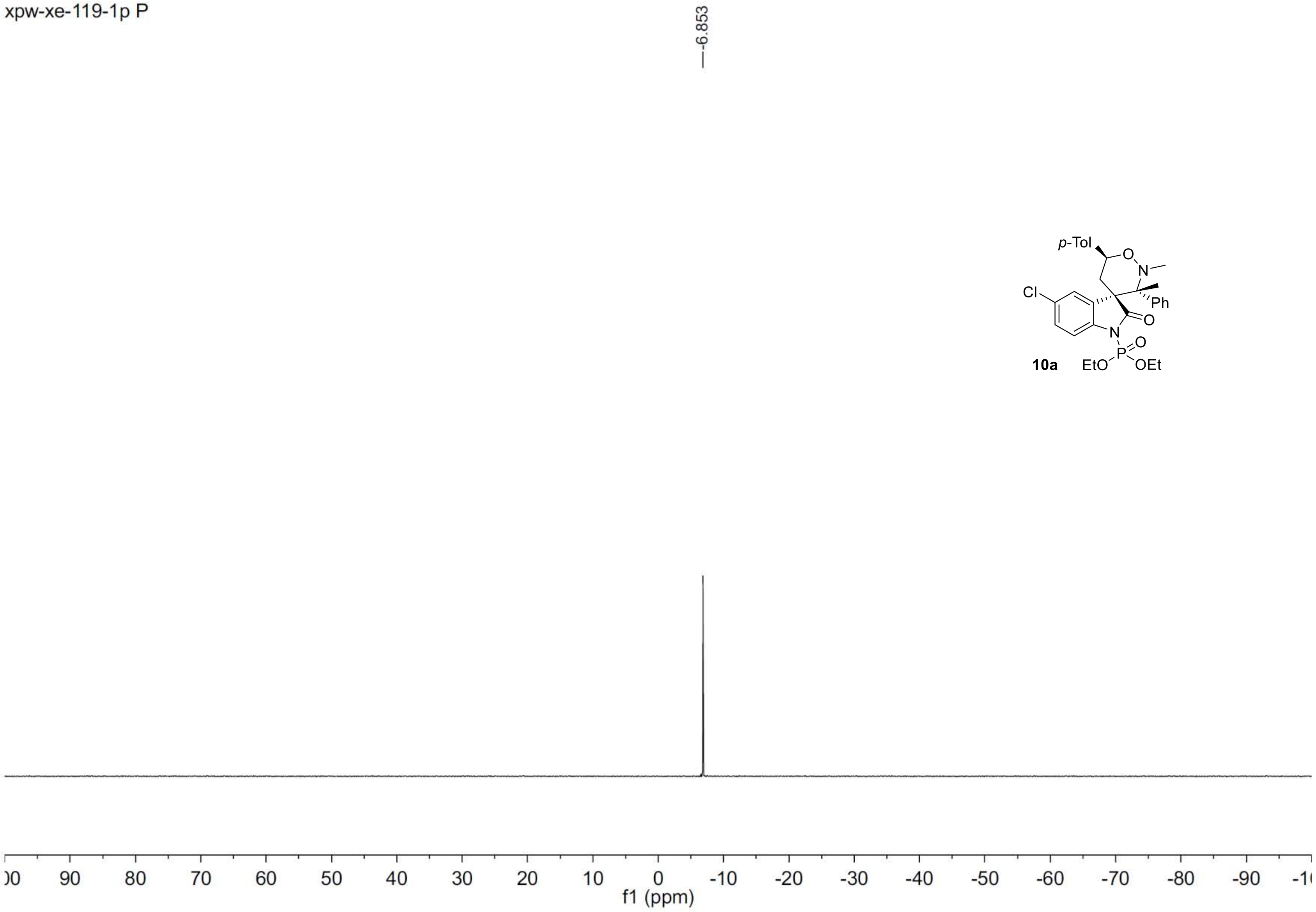

10a

Supplementary Figure 129. ^{31}P NMR (162 MHz, $CDCl_3$) spectra for compound **10a**

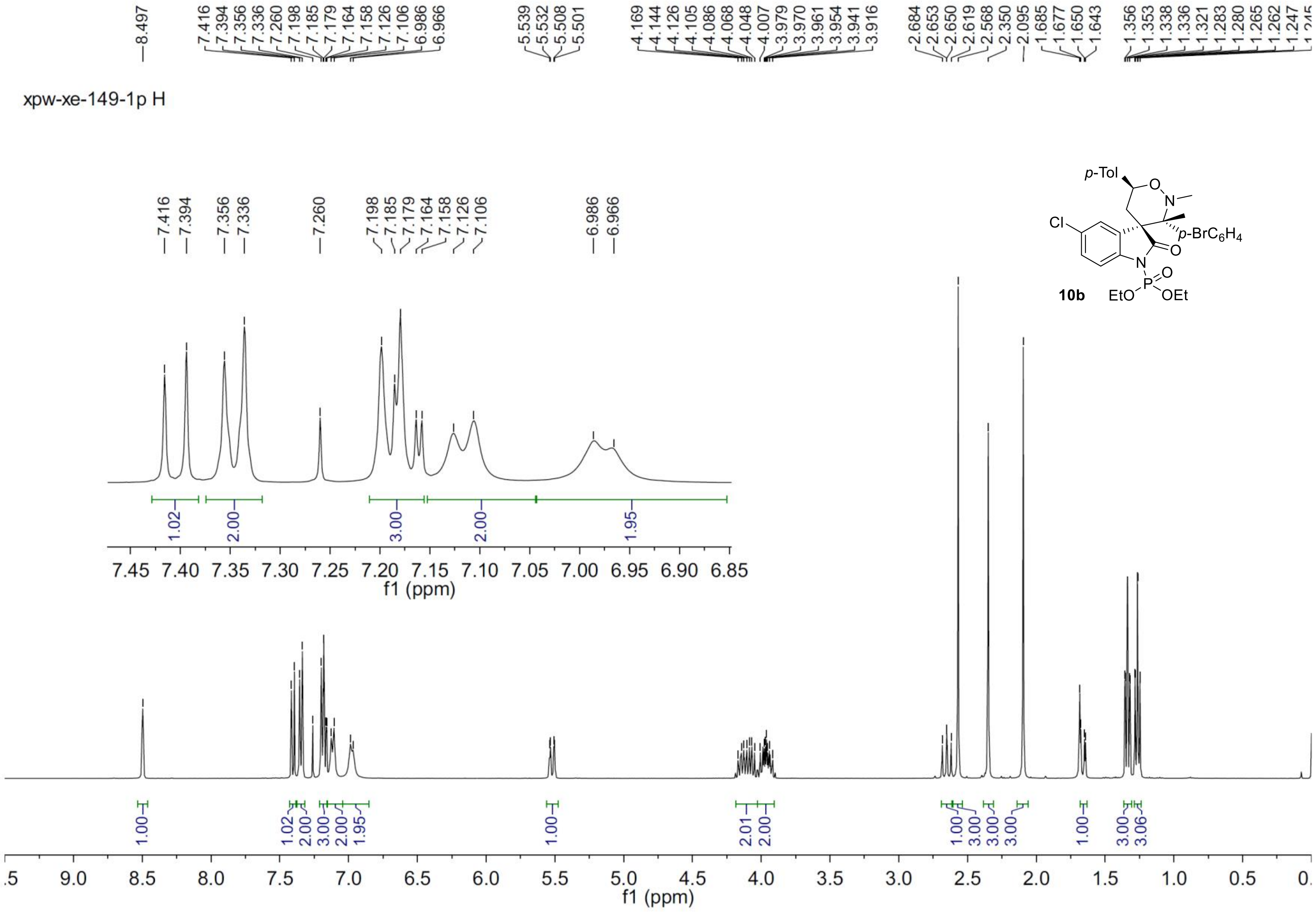

Supplementary Figure 130. ^{1}H NMR (400 MHz, $CDCl_3$) spectra for compound **10b**

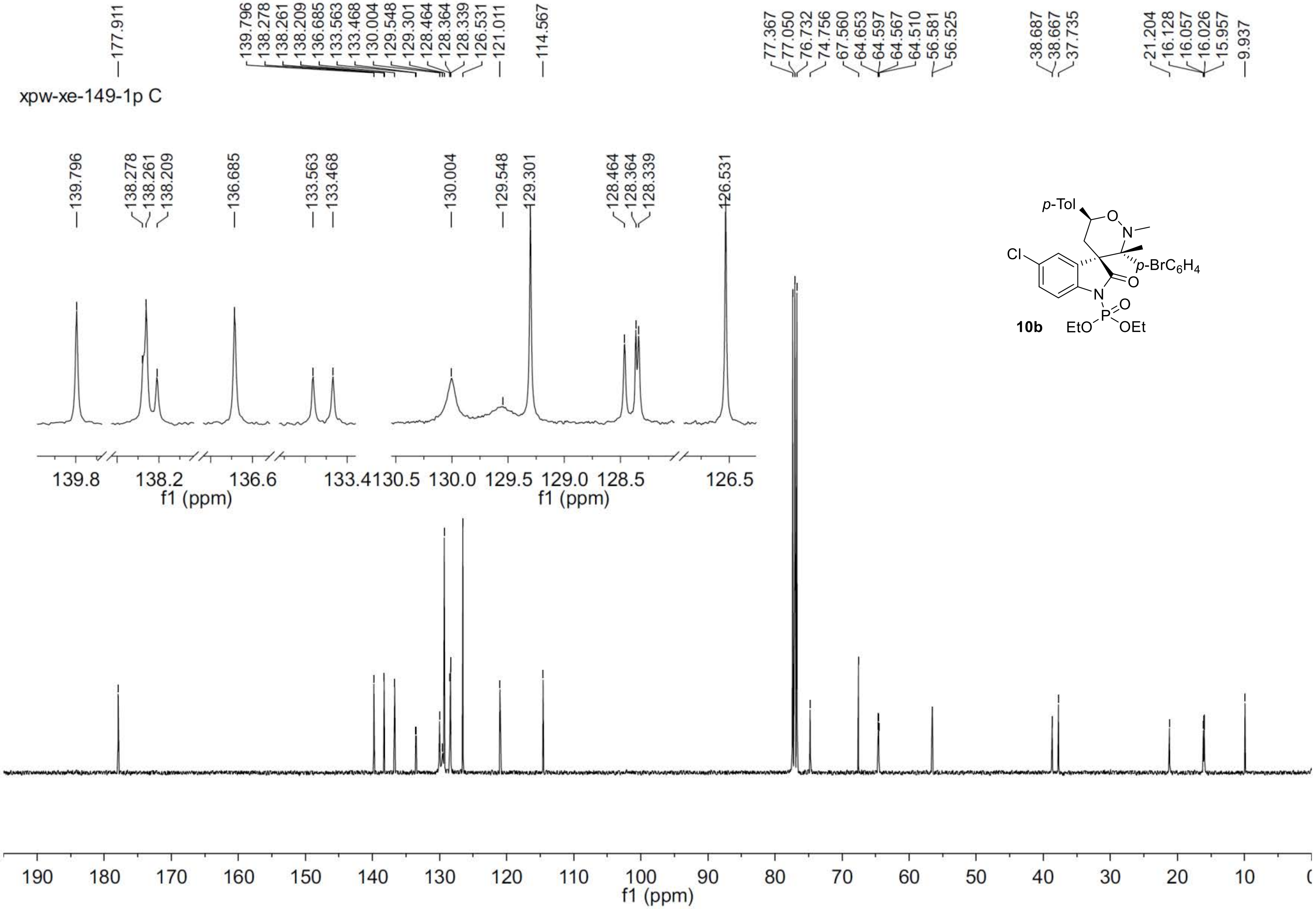

Supplementary Figure 131. ^{13}C NMR (100 MHz, $CDCl_3$) spectra for compound **10b**

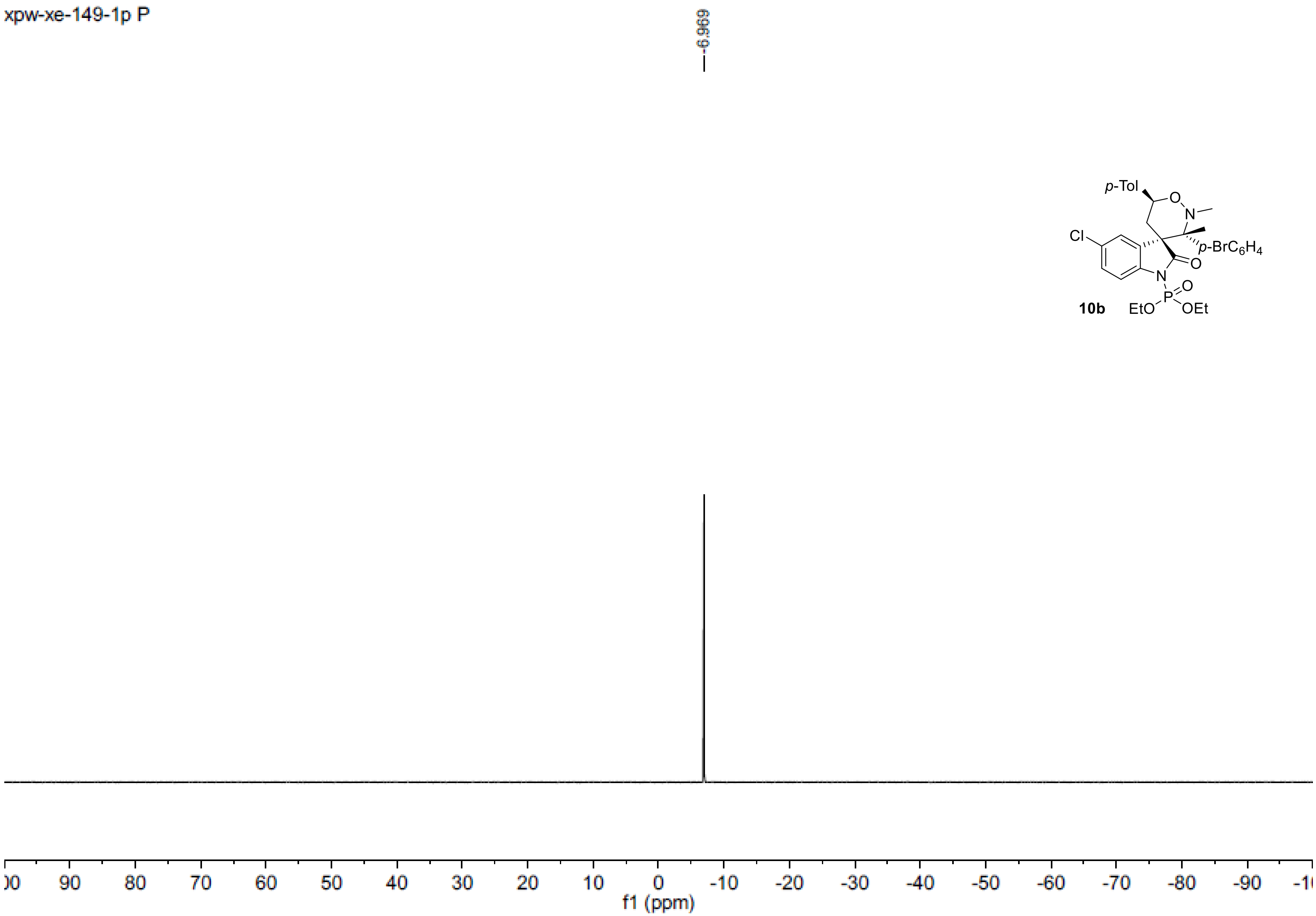

Supplementary Figure 132. ^{31}P NMR (162 MHz, $CDCl_3$) spectra for compound **10b**

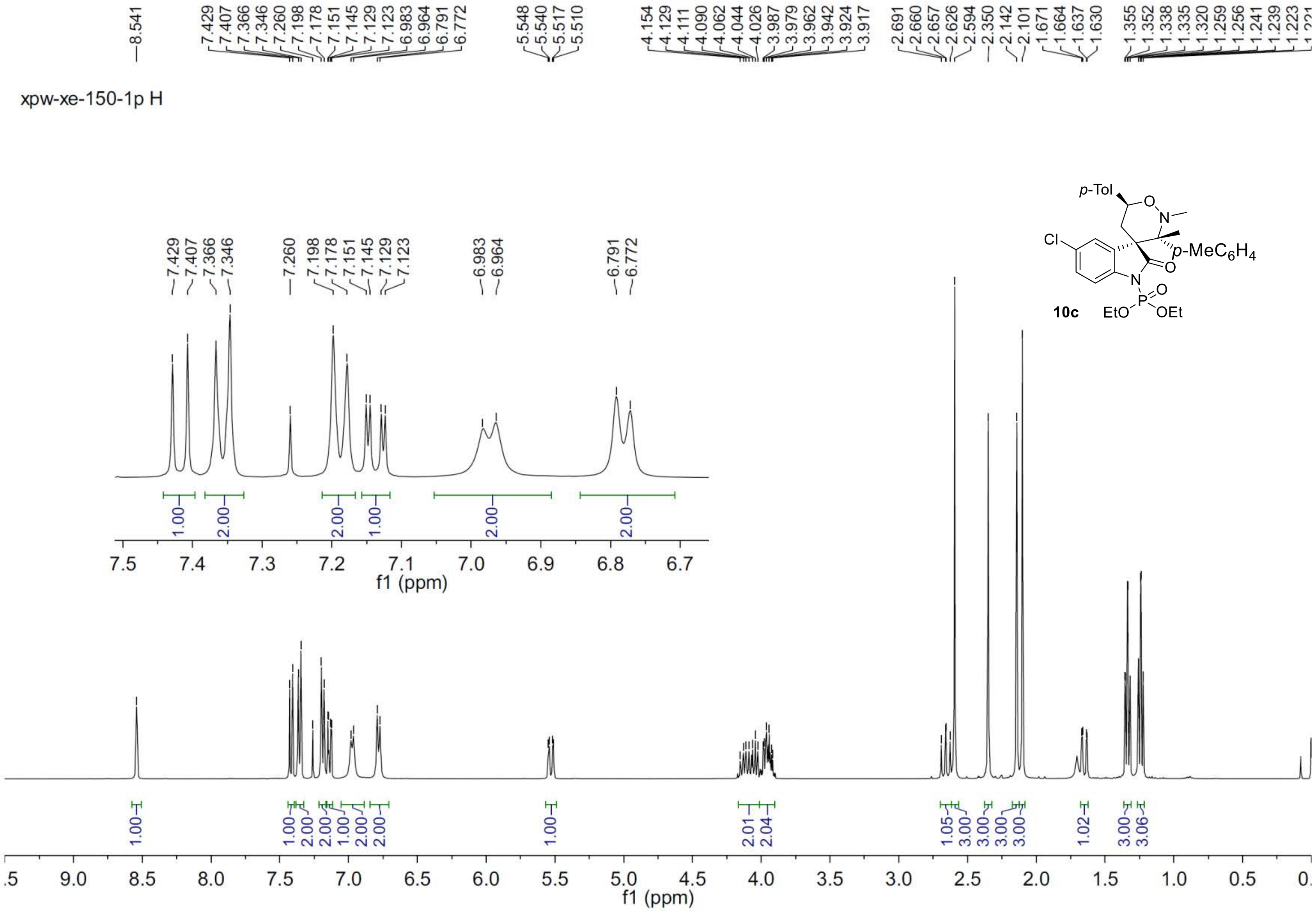

Supplementary Figure 133. ^{1}H NMR (400 MHz, $CDCl_3$) spectra for compound **10c**

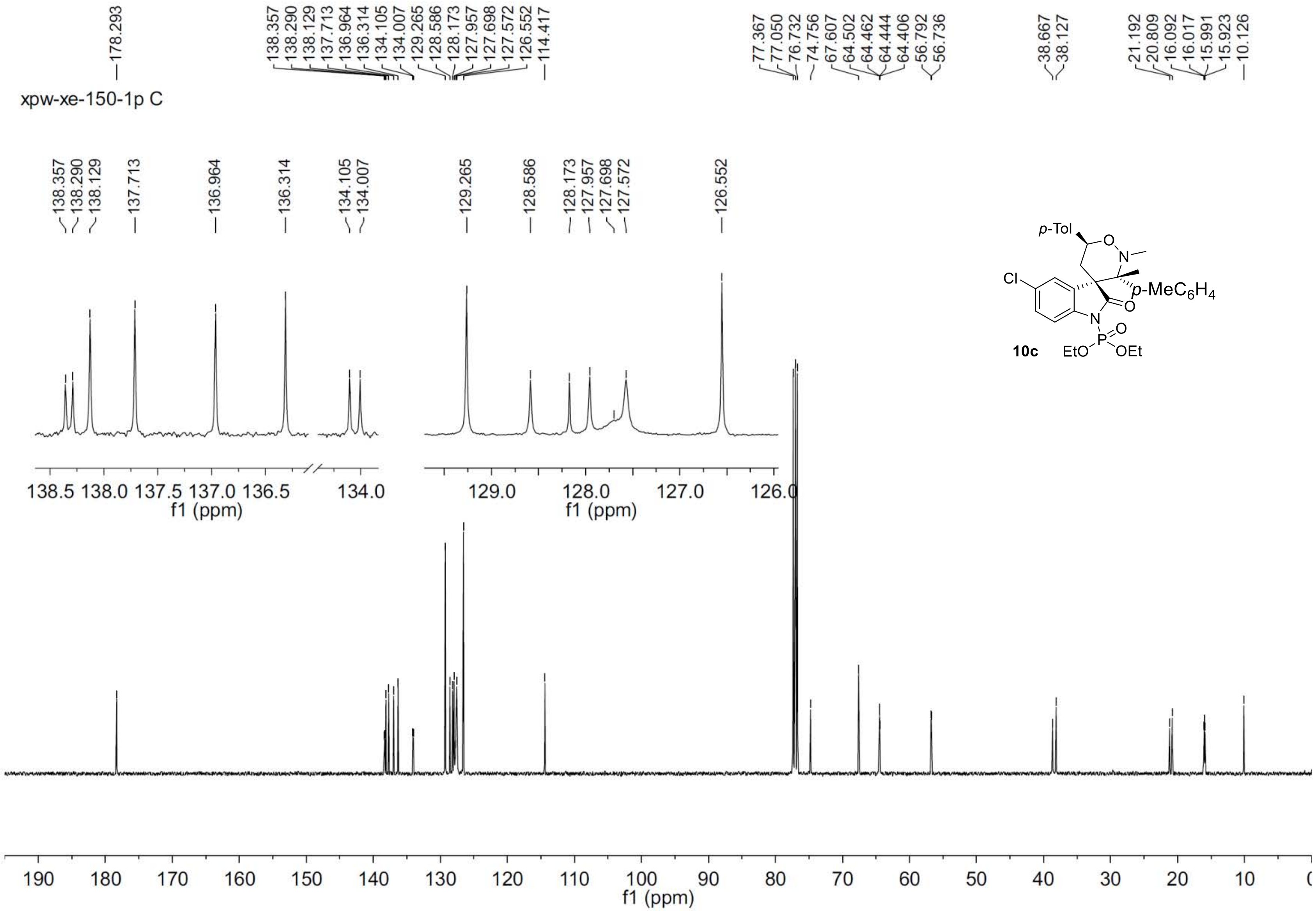

Supplementary Figure 134. ^{13}C NMR (100 MHz, $CDCl_3$) spectra for compound **10c**

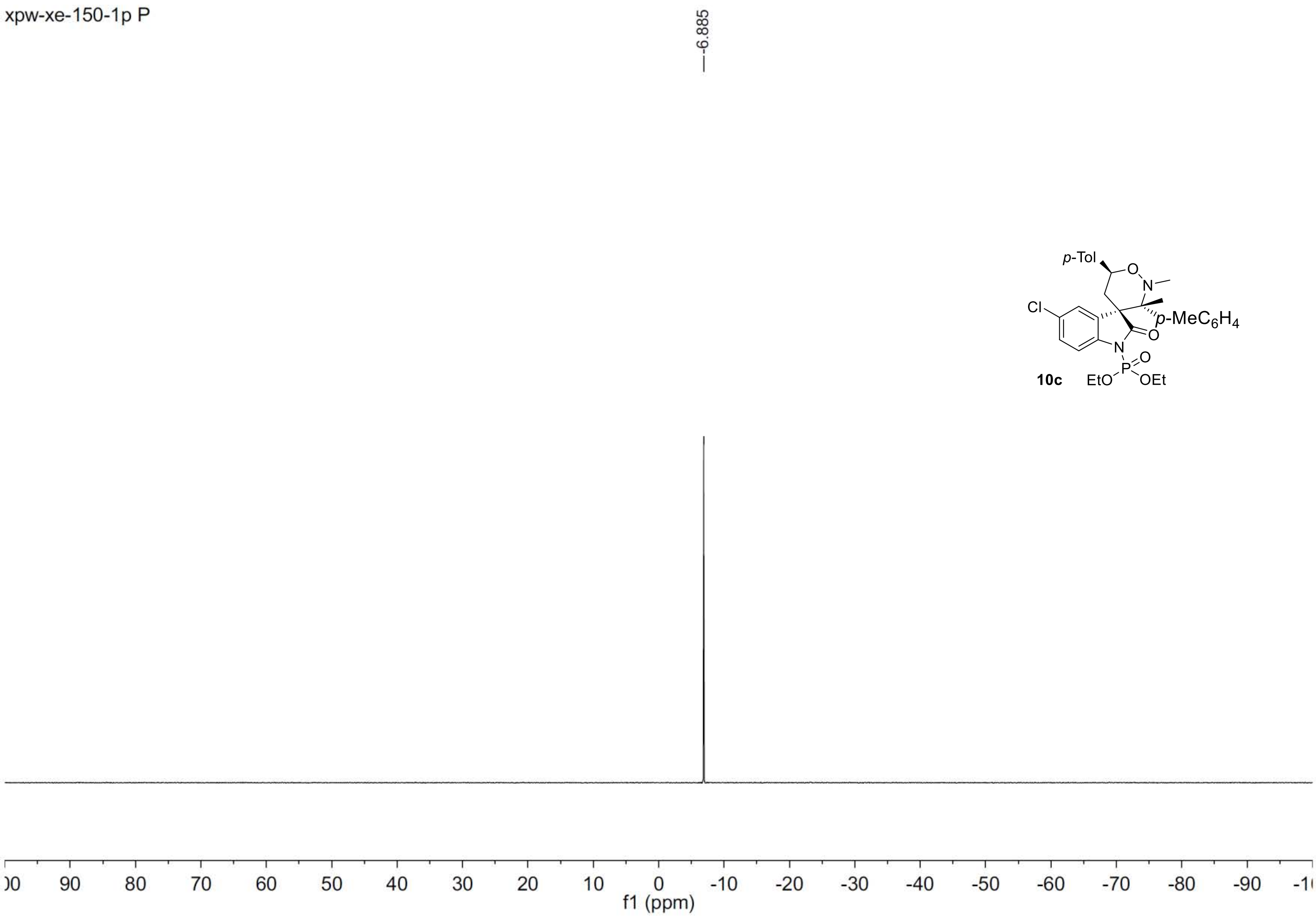

Supplementary Figure 135. ^{31}P NMR (162 MHz, $CDCl_3$) spectra for compound **10c**

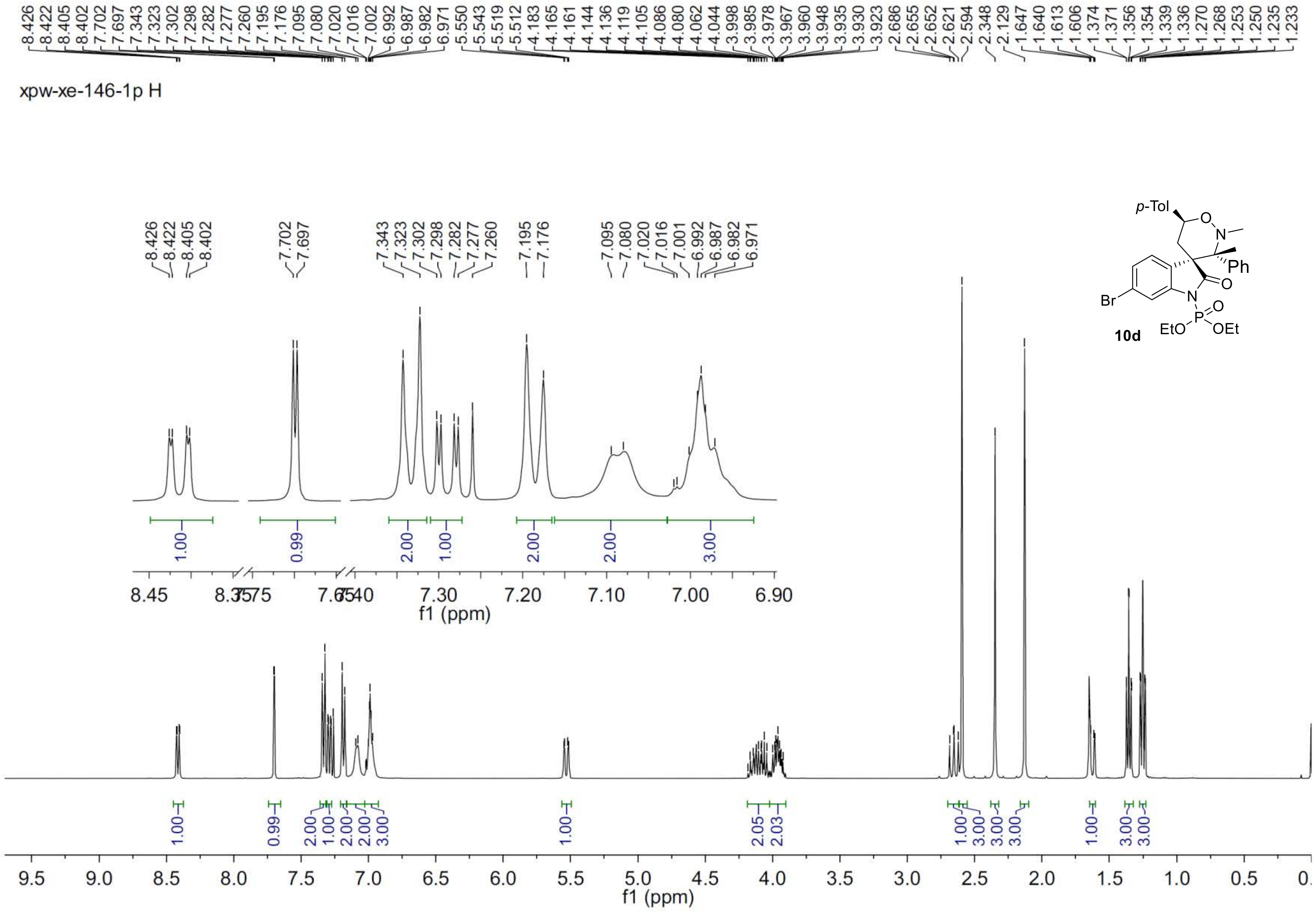

Supplementary Figure 136. ^{1}H NMR (400 MHz, $CDCl_3$) spectra for compound **10d**

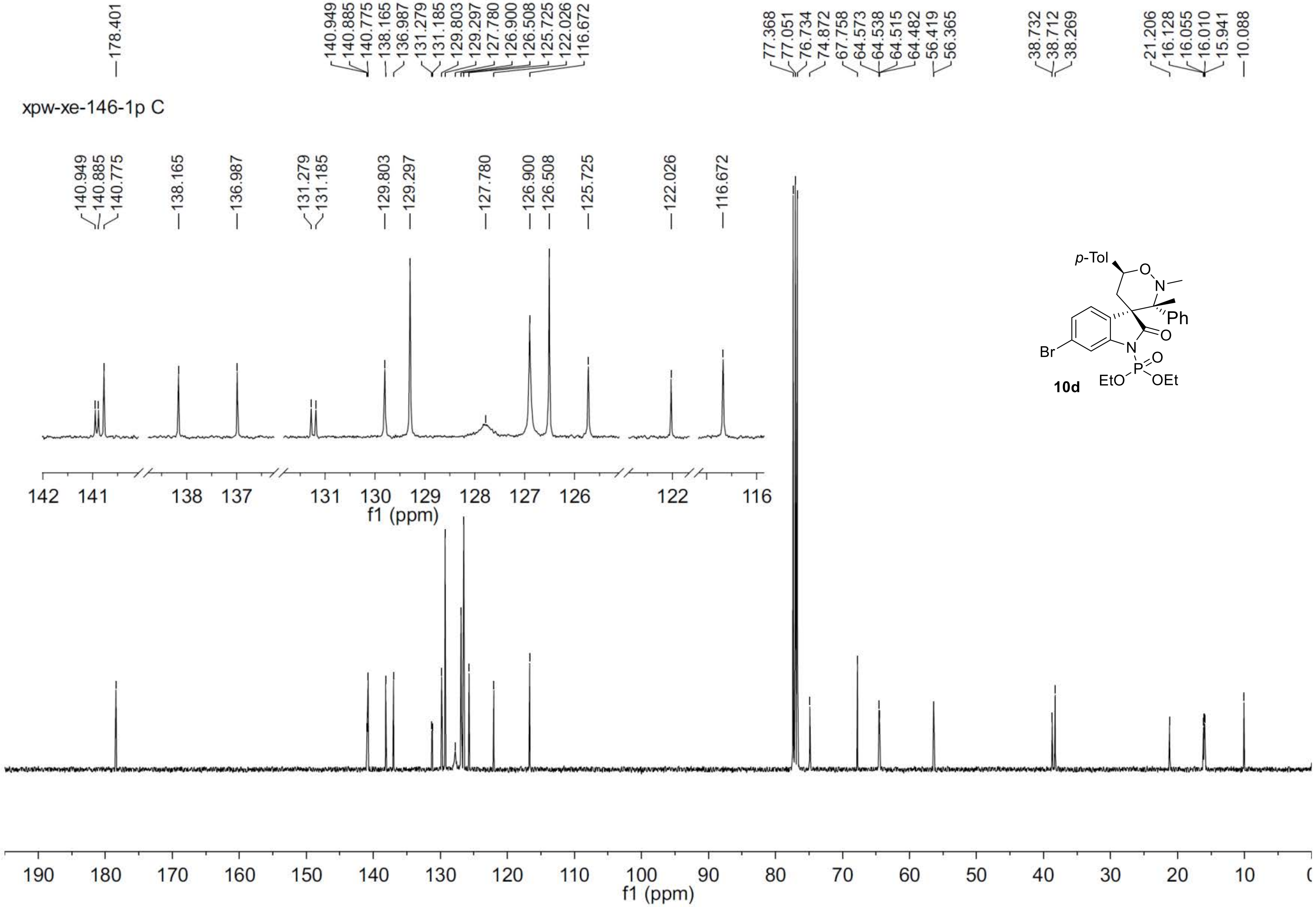

Supplementary Figure 137. ^{13}C NMR (100 MHz, $CDCl_3$) spectra for compound **10d**

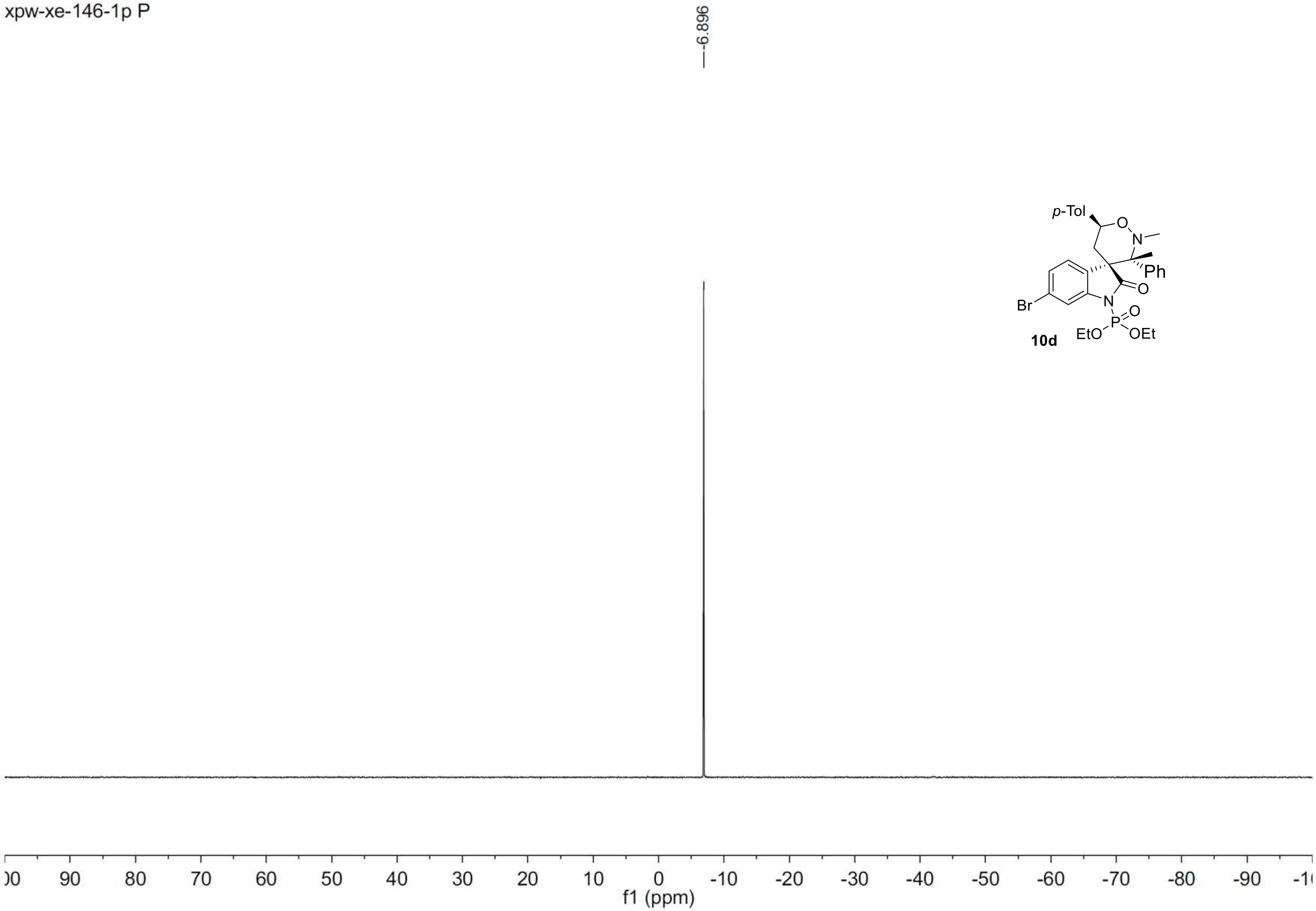

10d

Supplementary Figure 138. ^{31}P NMR (162 MHz, $CDCl_3$) spectra for compound **10d**

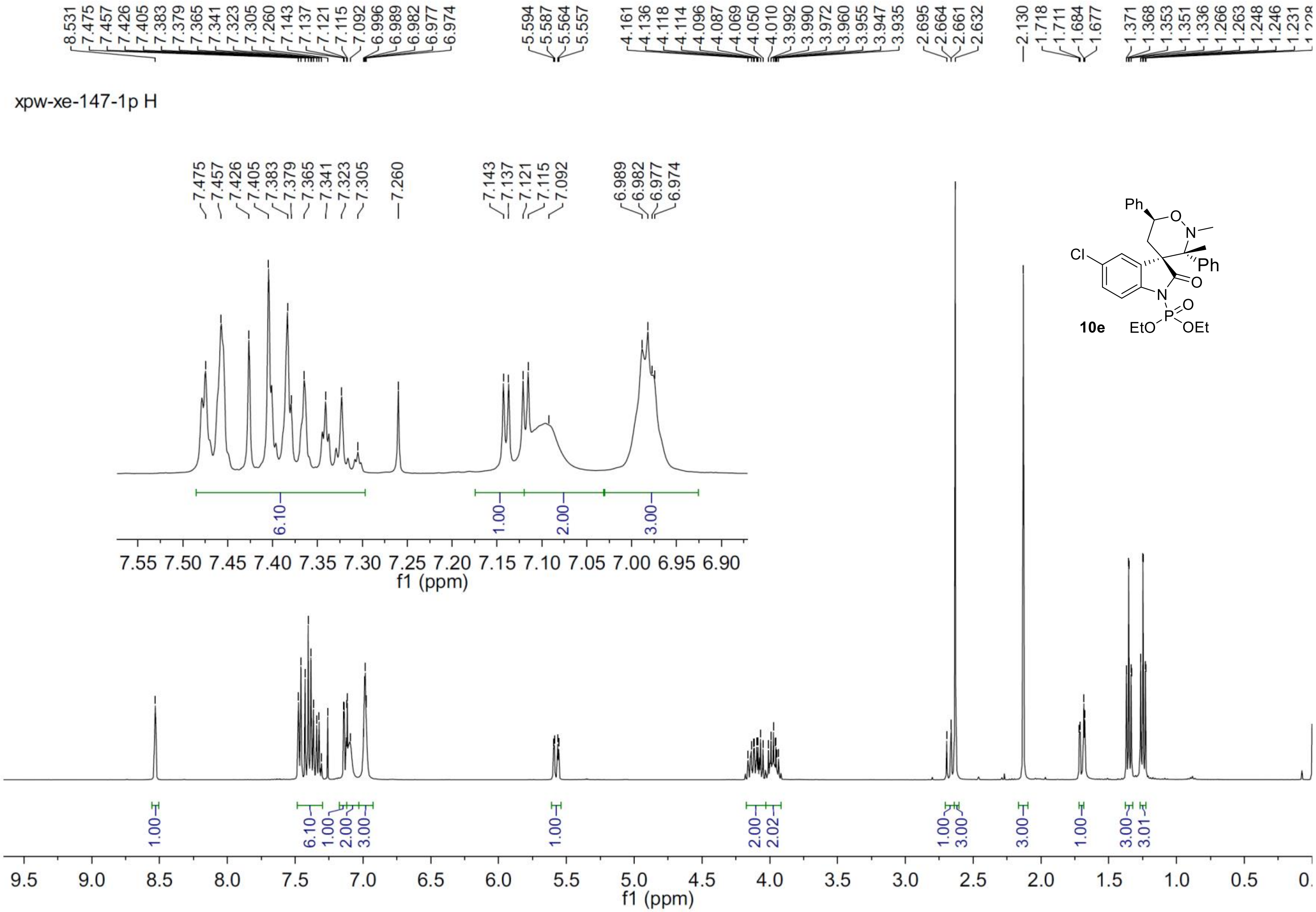

Supplementary Figure 139. ^{1}H NMR (400 MHz, $CDCl_3$) spectra for compound **10e**

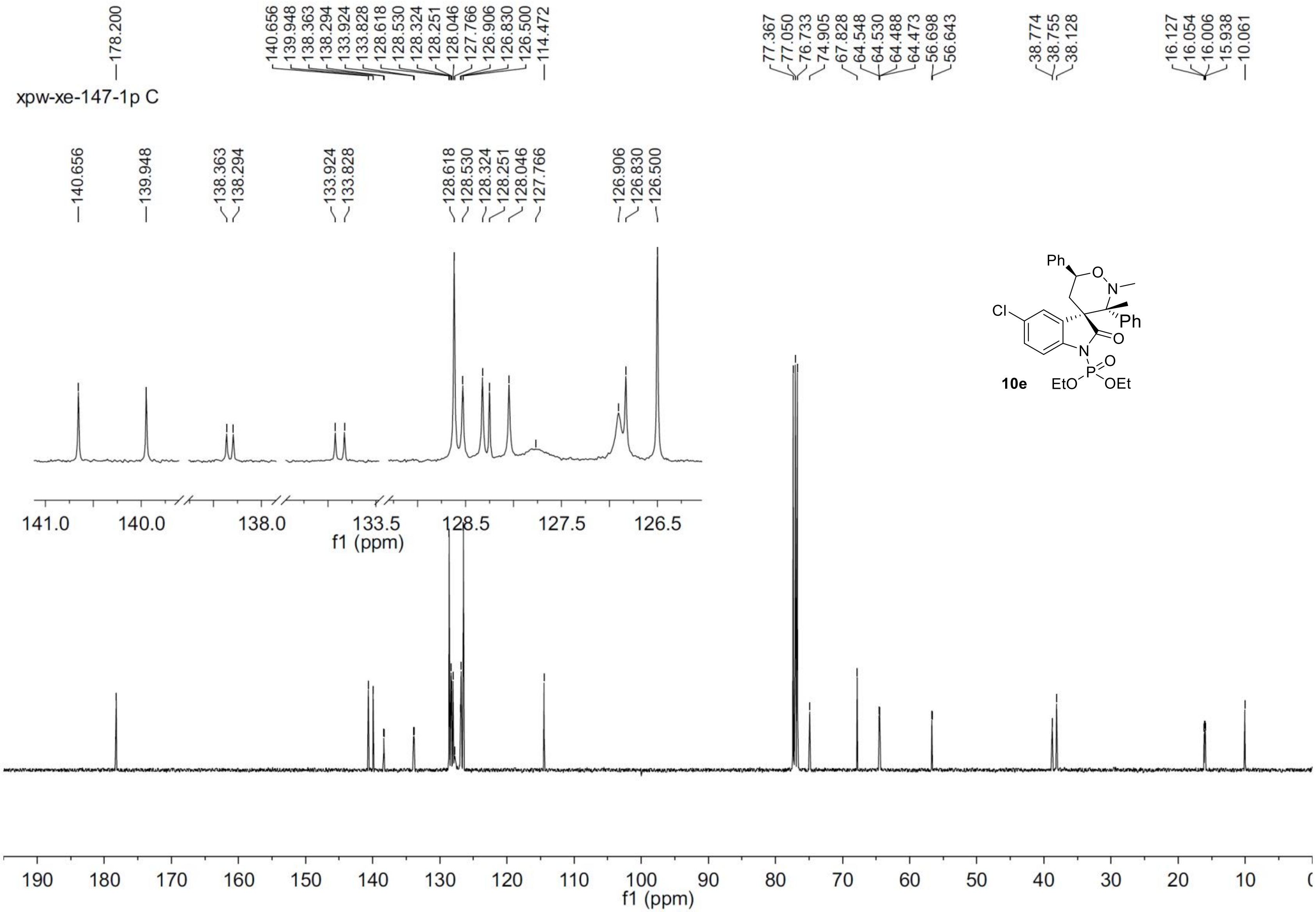

Supplementary Figure 140. ^{13}C NMR (100 MHz, $CDCl_3$) spectra for compound **10e**

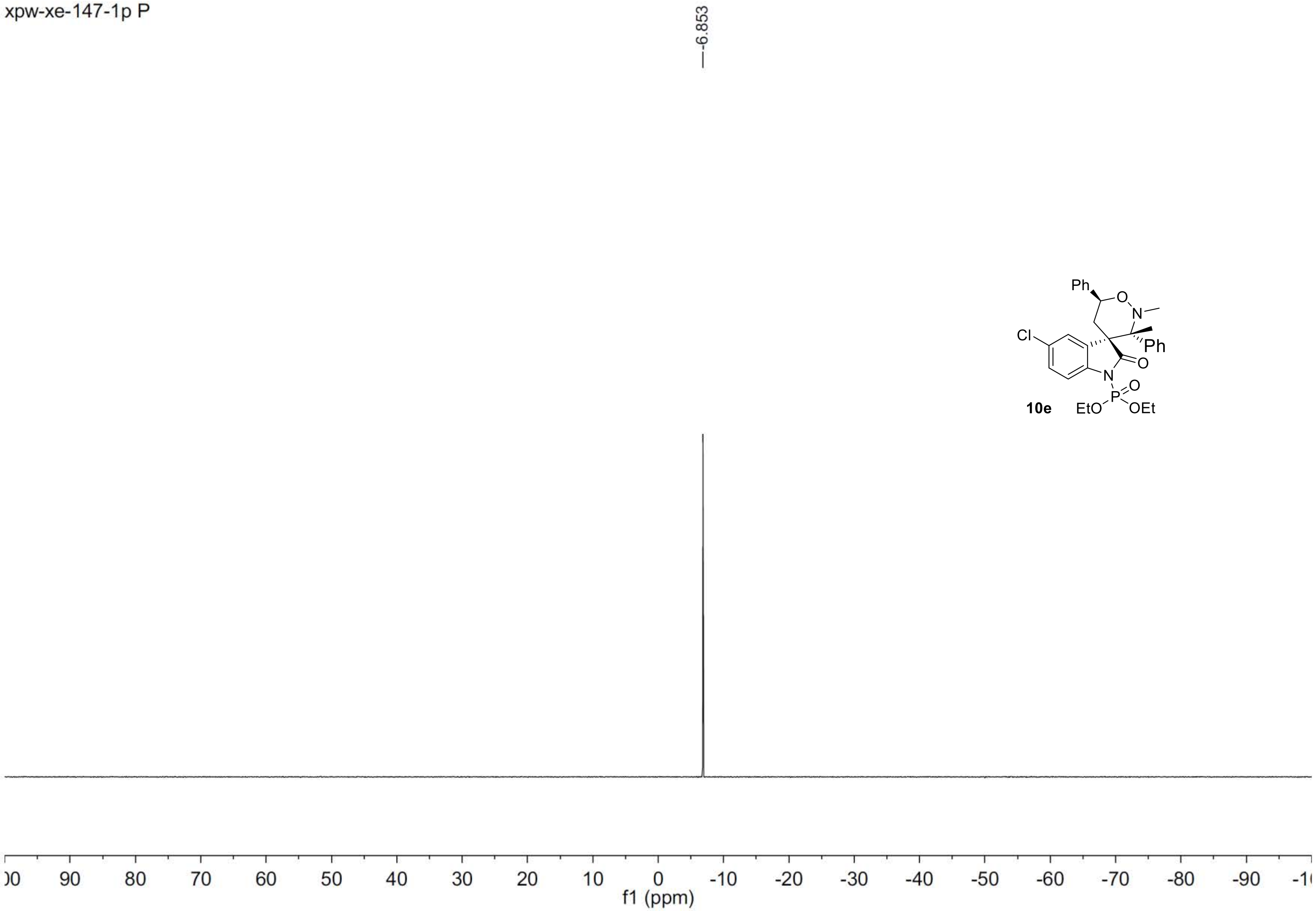

Supplementary Figure 141. ^{31}P NMR (162 MHz, $CDCl_3$) spectra for compound **10e**

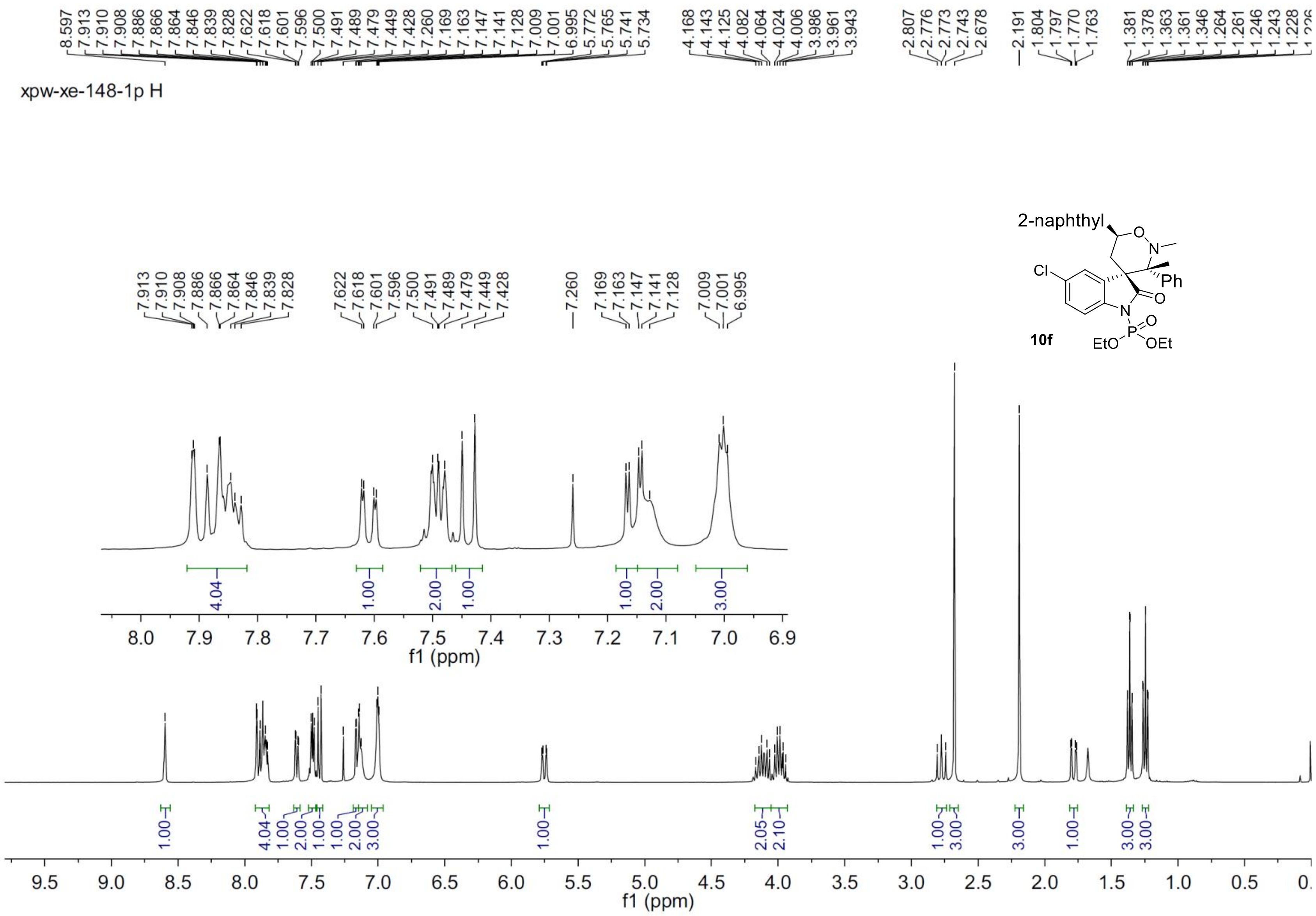

Supplementary Figure 142. ^{1}H NMR (400 MHz, $CDCl_3$) spectra for compound **10f**

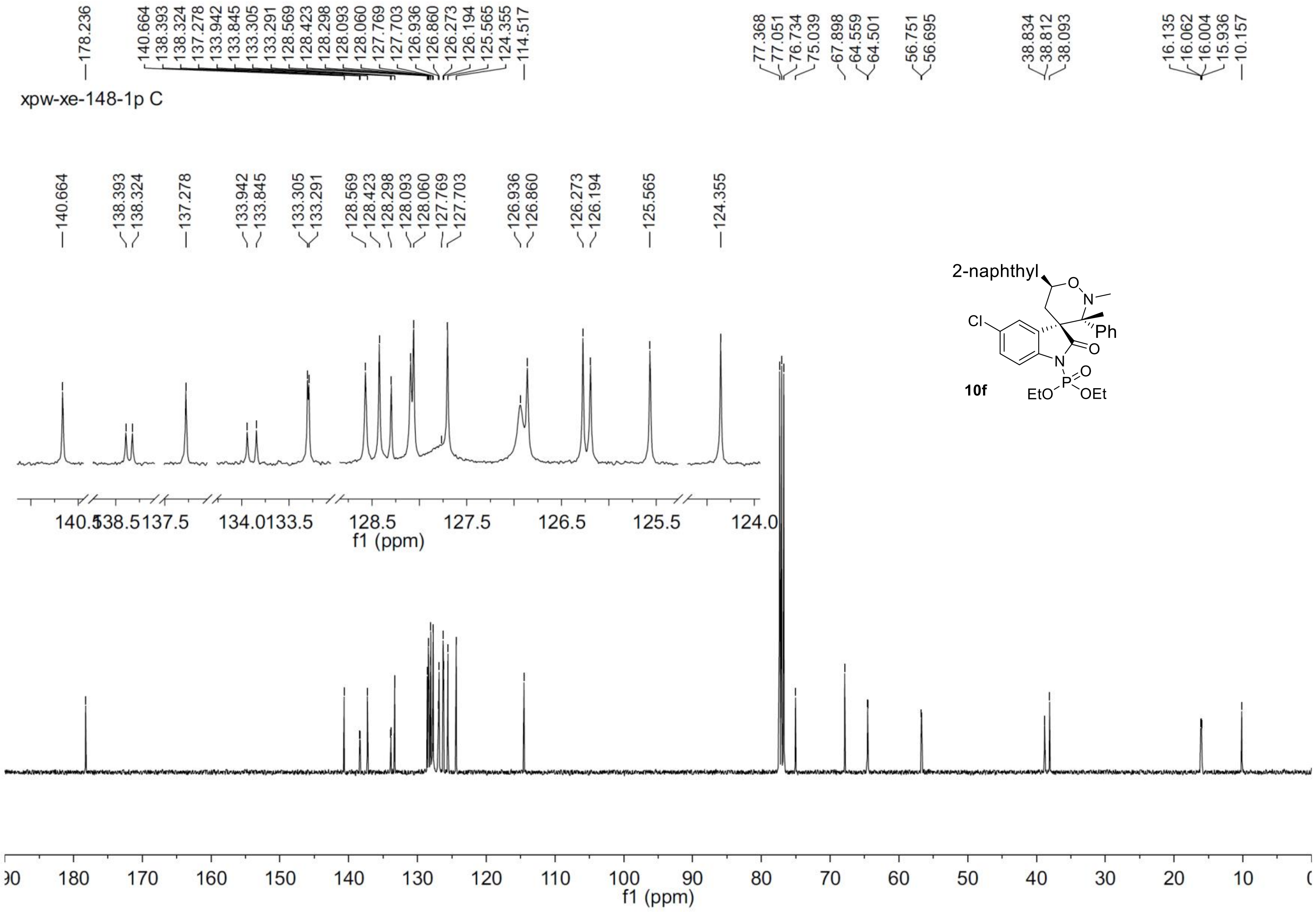

Supplementary Figure 143. ^{13}C NMR (100 MHz, $CDCl_3$) spectra for compound **10f**

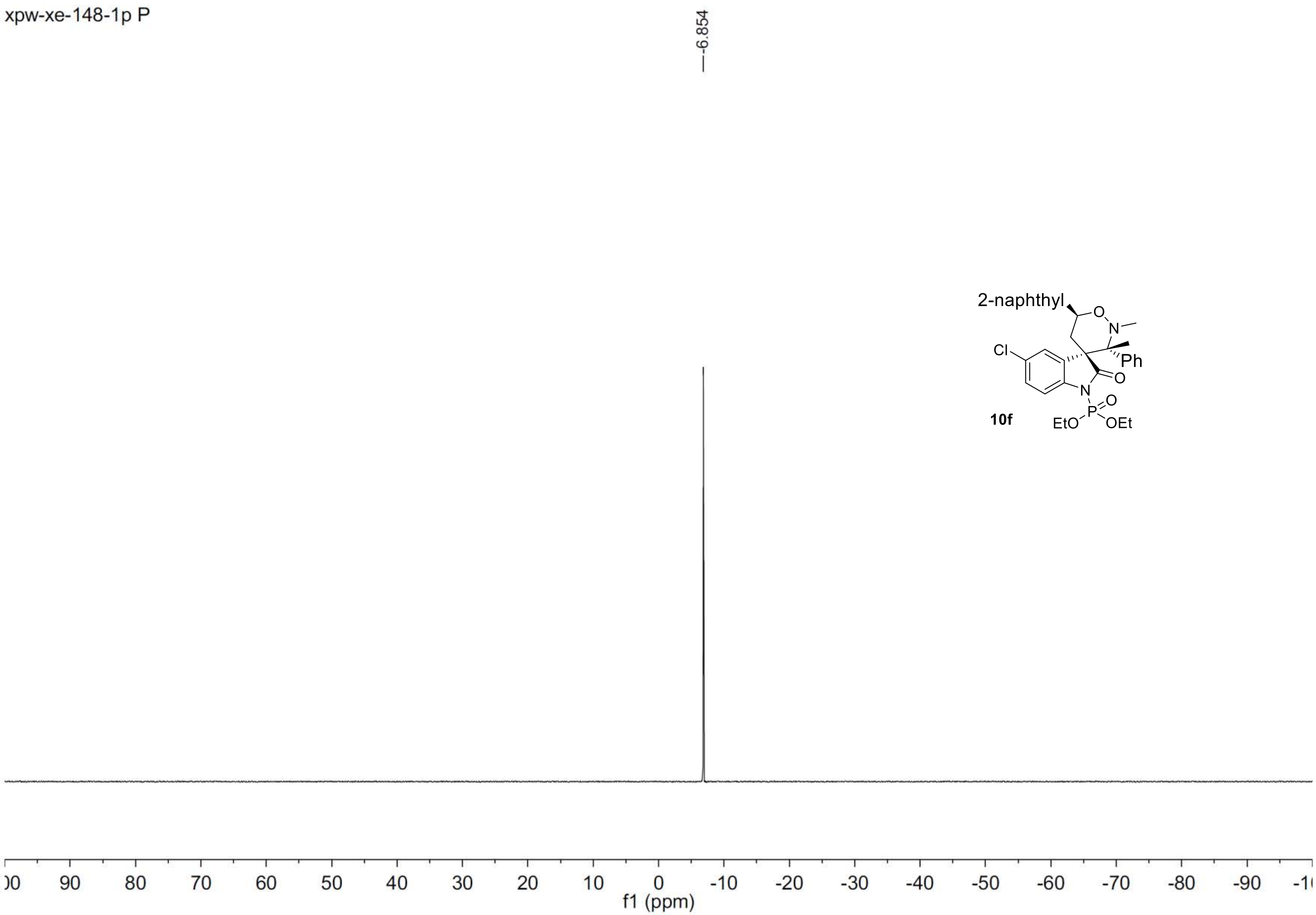

Supplementary Figure 144. ^{31}P NMR (162 MHz, $CDCl_3$) spectra for compound **10f**

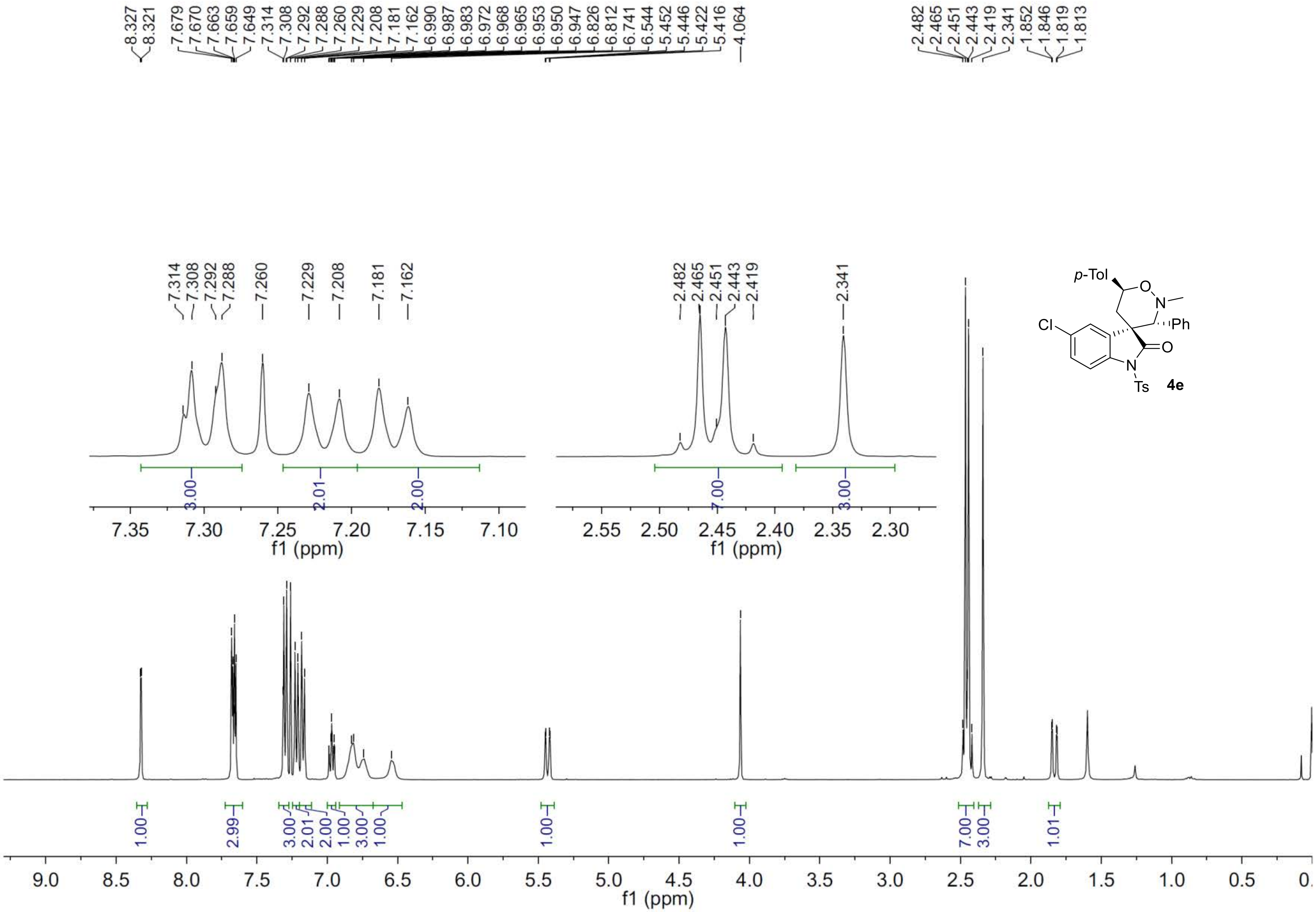

Supplementary Figure 145. ^{1}H NMR (400 MHz, $CDCl_3$) spectra for compound **4e**

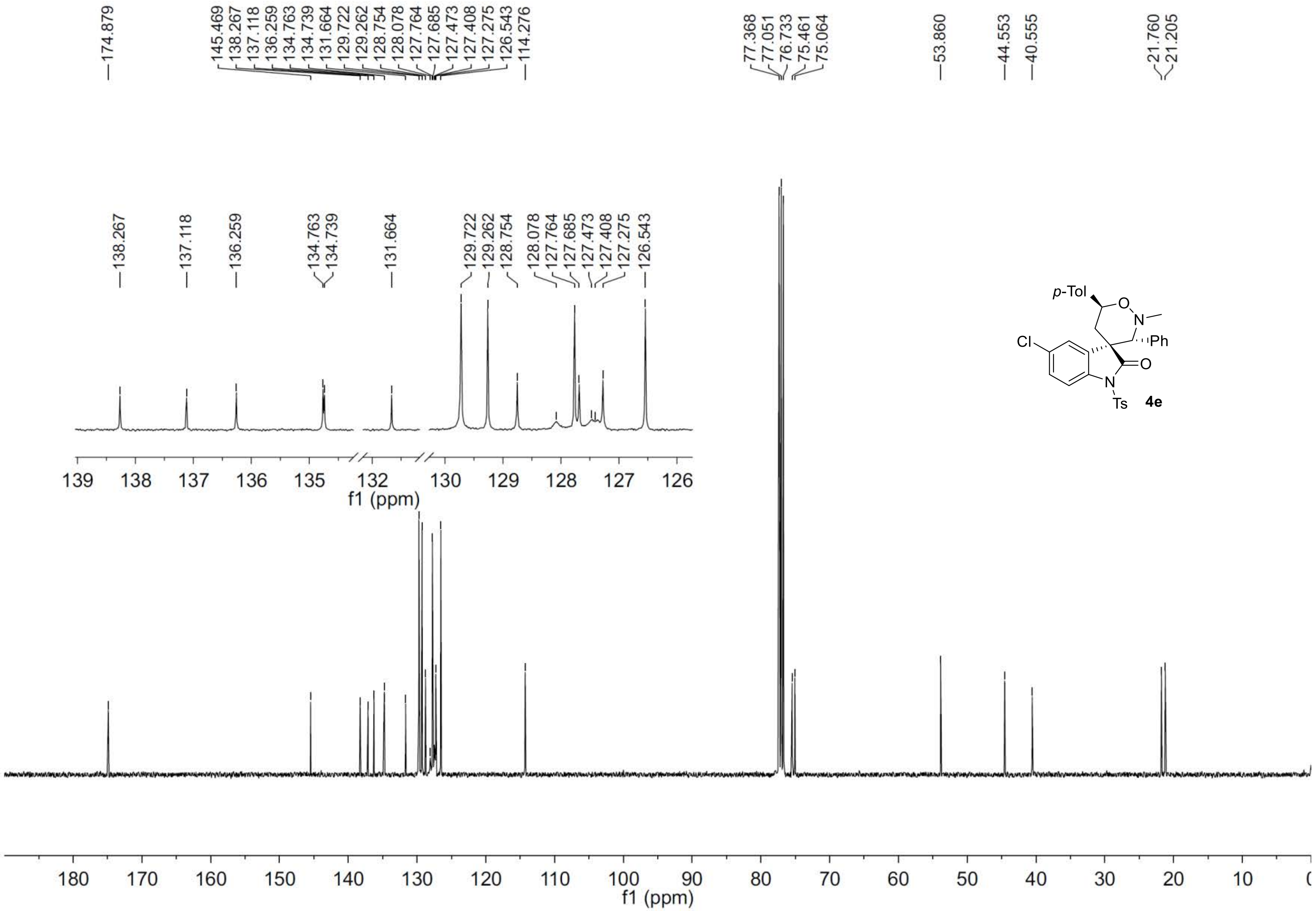

4e

Supplementary Figure 146. ^{13}C NMR (100 MHz, $CDCl_3$) spectra for compound **4e**

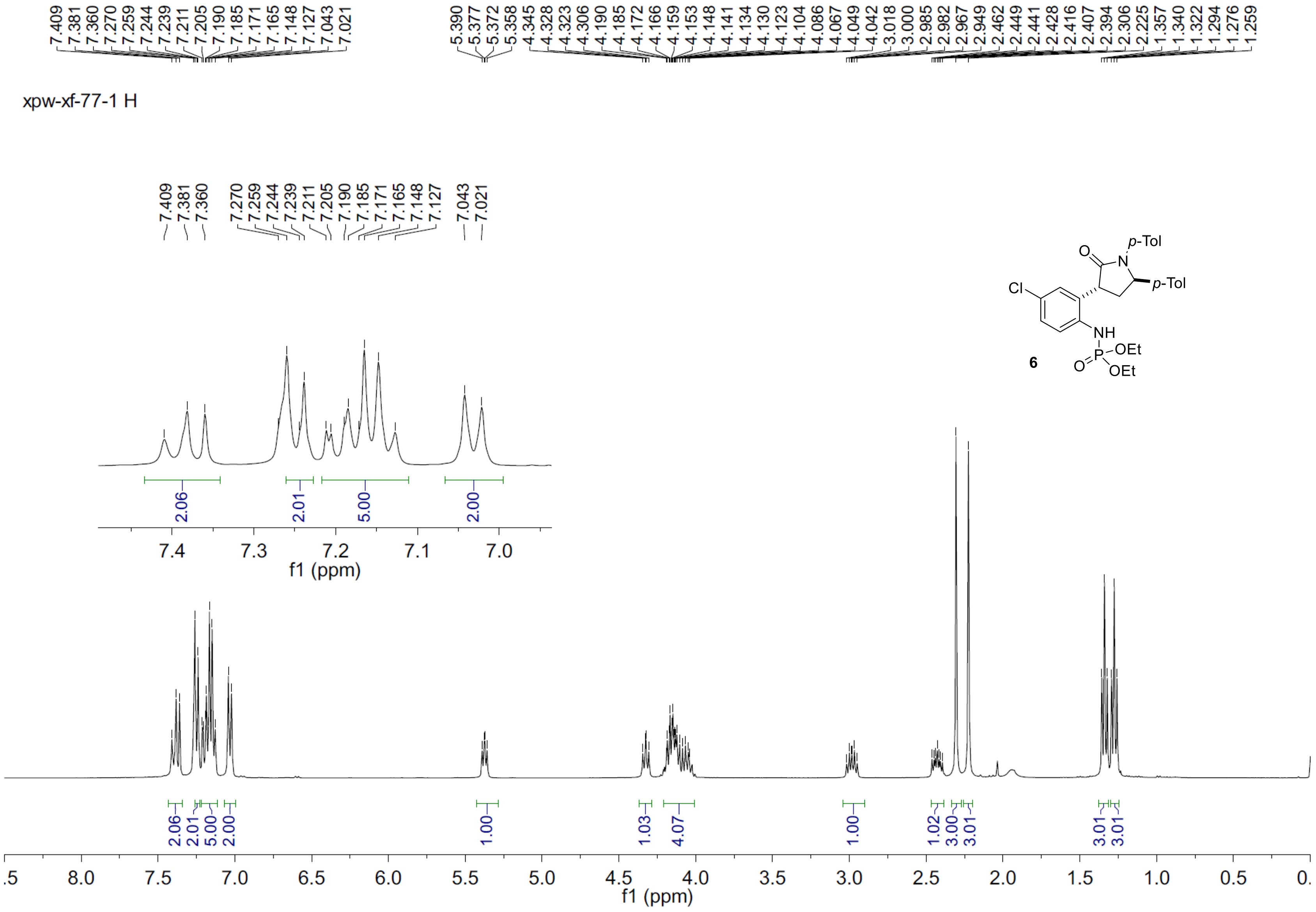

Supplementary Figure 147. ^{1}H NMR (400 MHz, $CDCl_3$) spectra for compound **6**

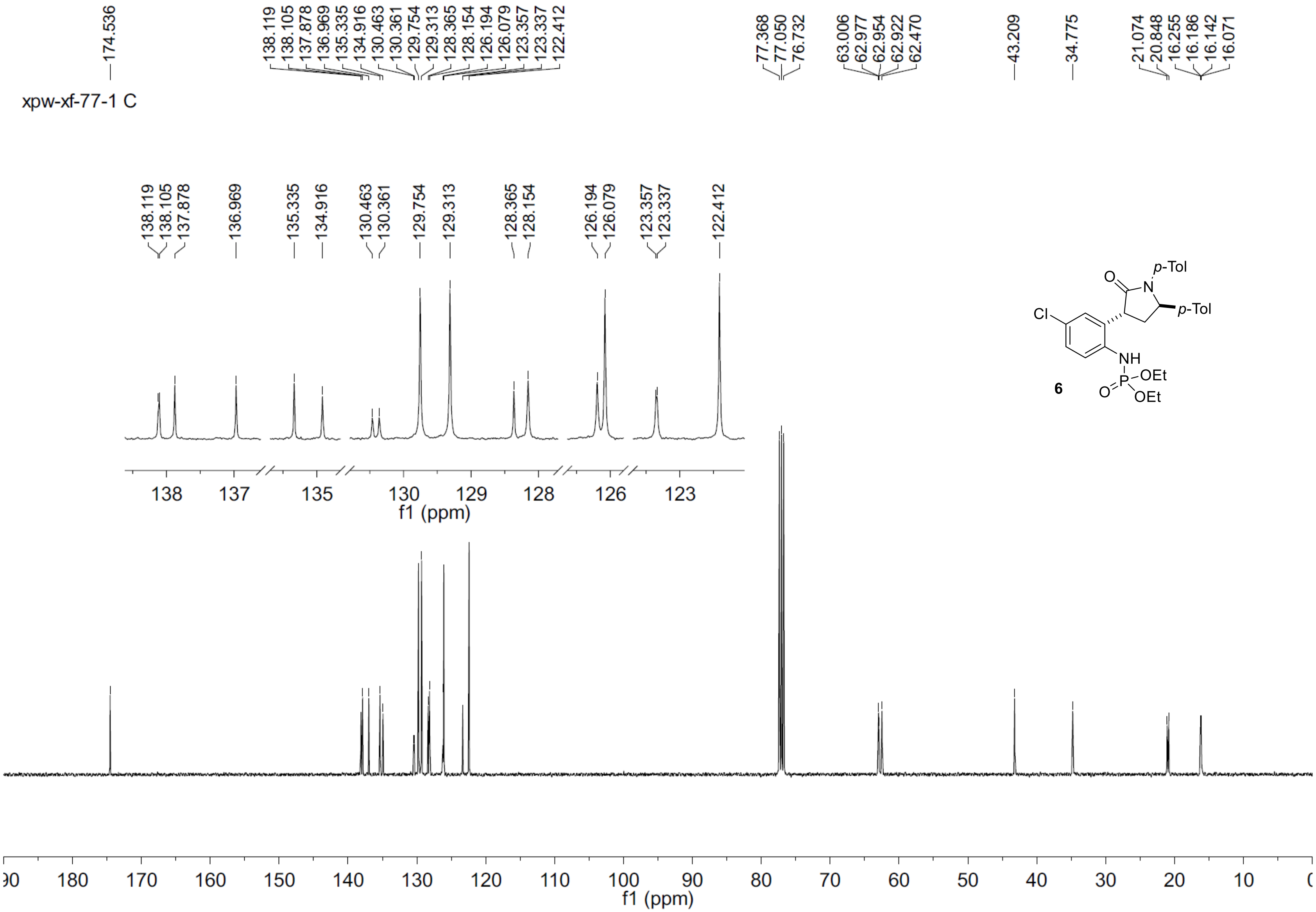

Supplementary Figure 148. ^{13}C NMR (100 MHz, $CDCl_3$) spectra for compound **6**

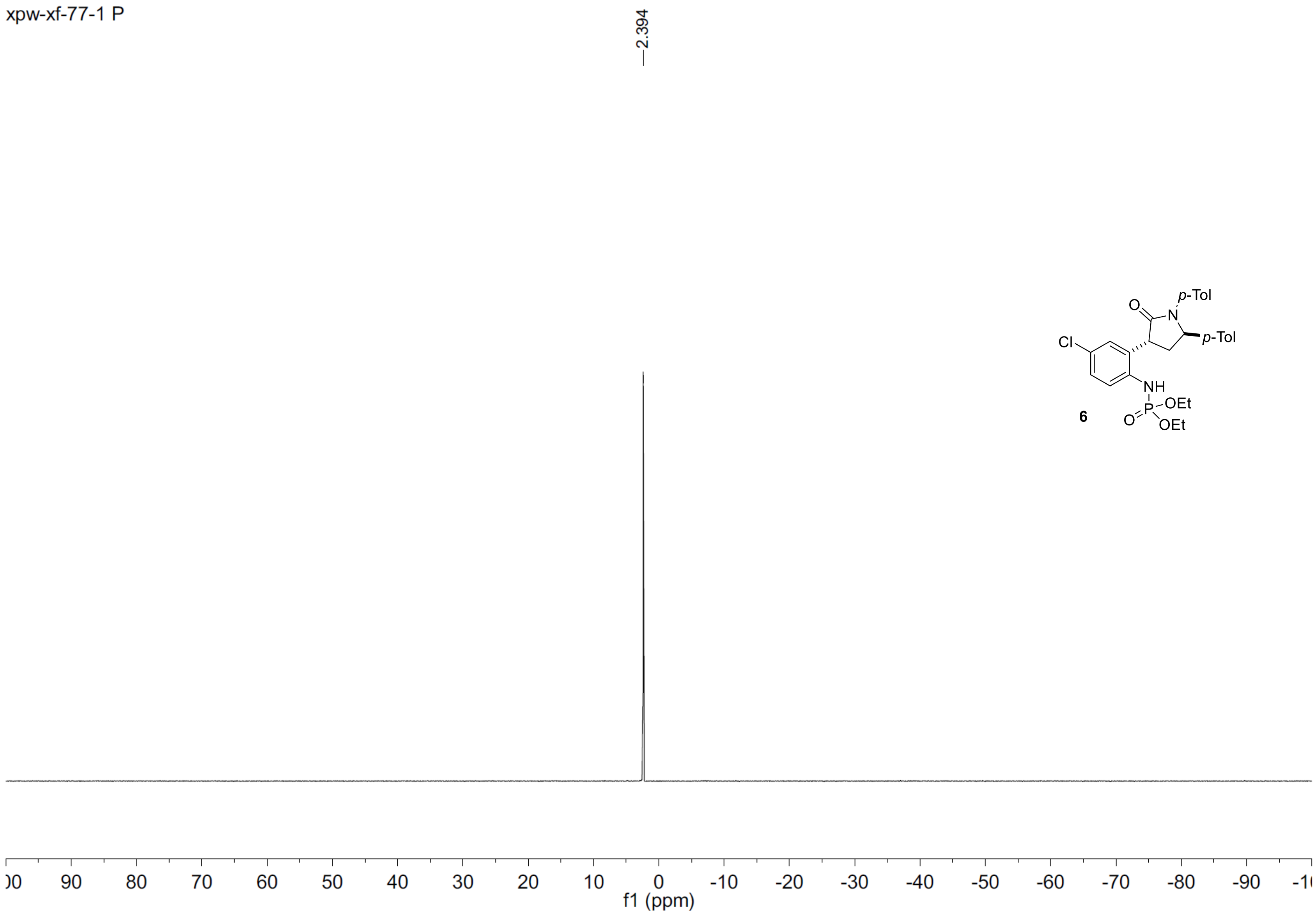

Supplementary Figure 149 ^{31}P NMR (162 MHz, $CDCl_3$) spectra for compound **6**

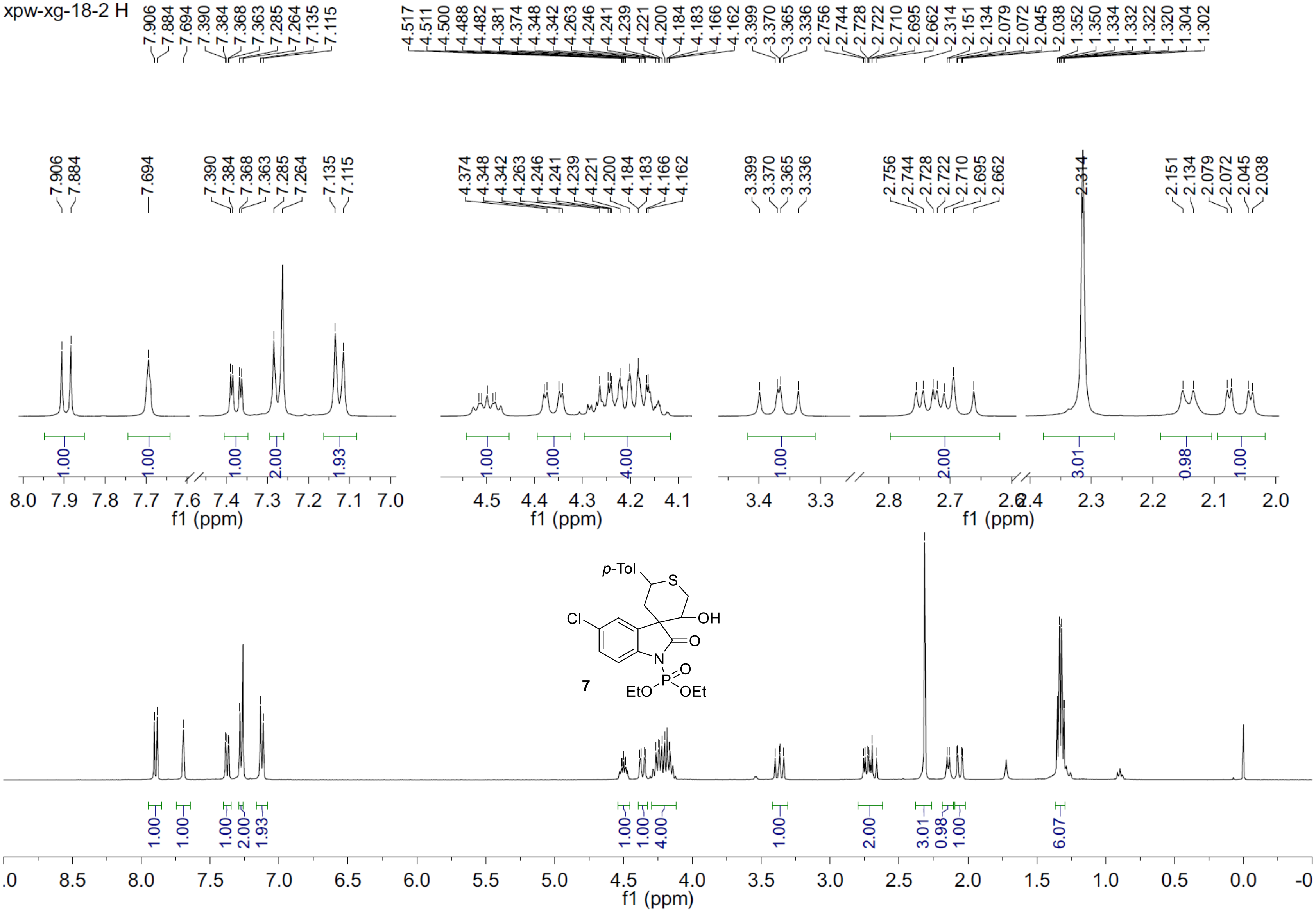

Supplementary Figure 150. ^{1}H NMR (400 MHz, $CDCl_3$) spectra for compound **7**

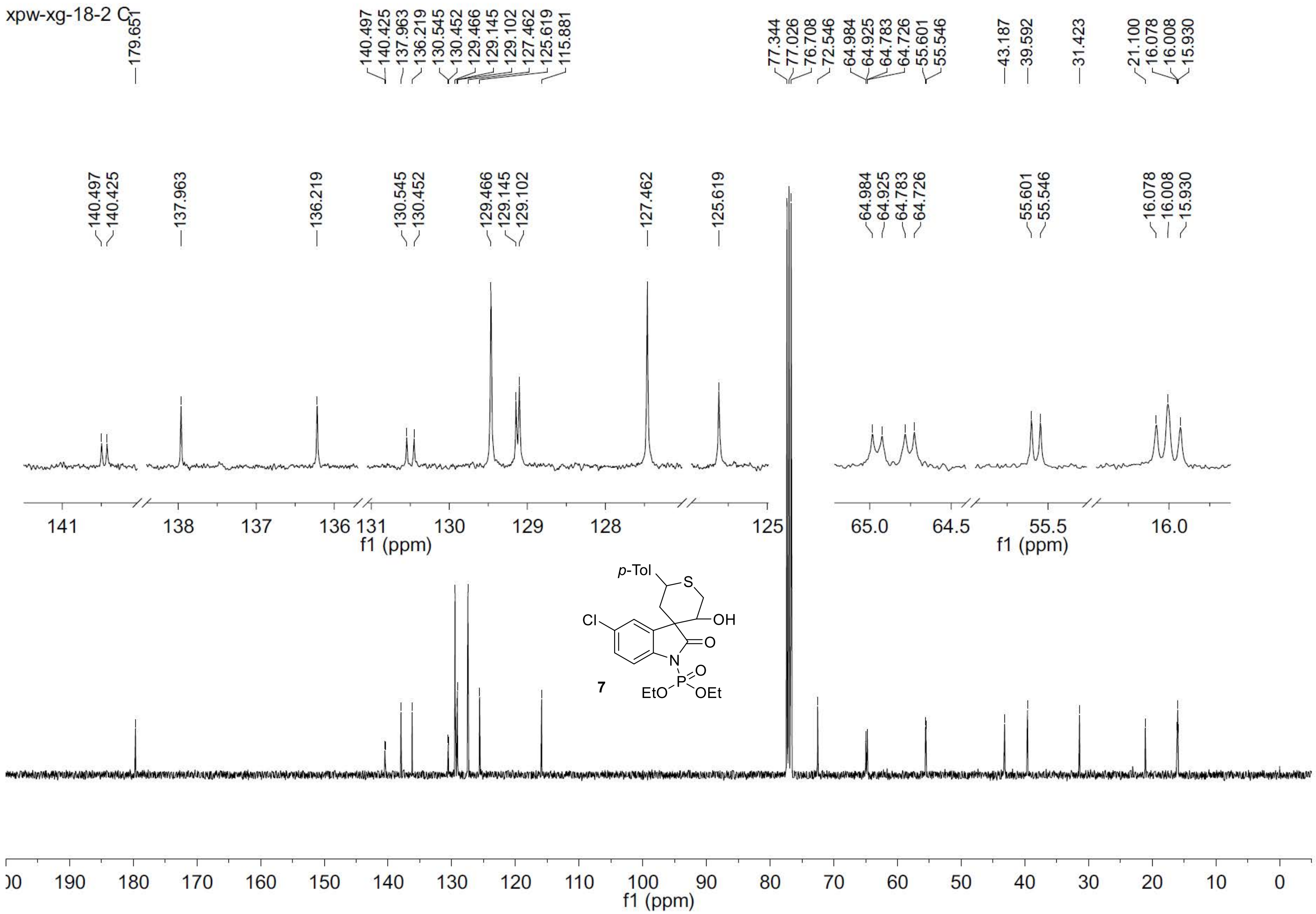

Supplementary Figure 151. ^{13}C NMR (100 MHz, $CDCl_3$) spectra for compound **7**

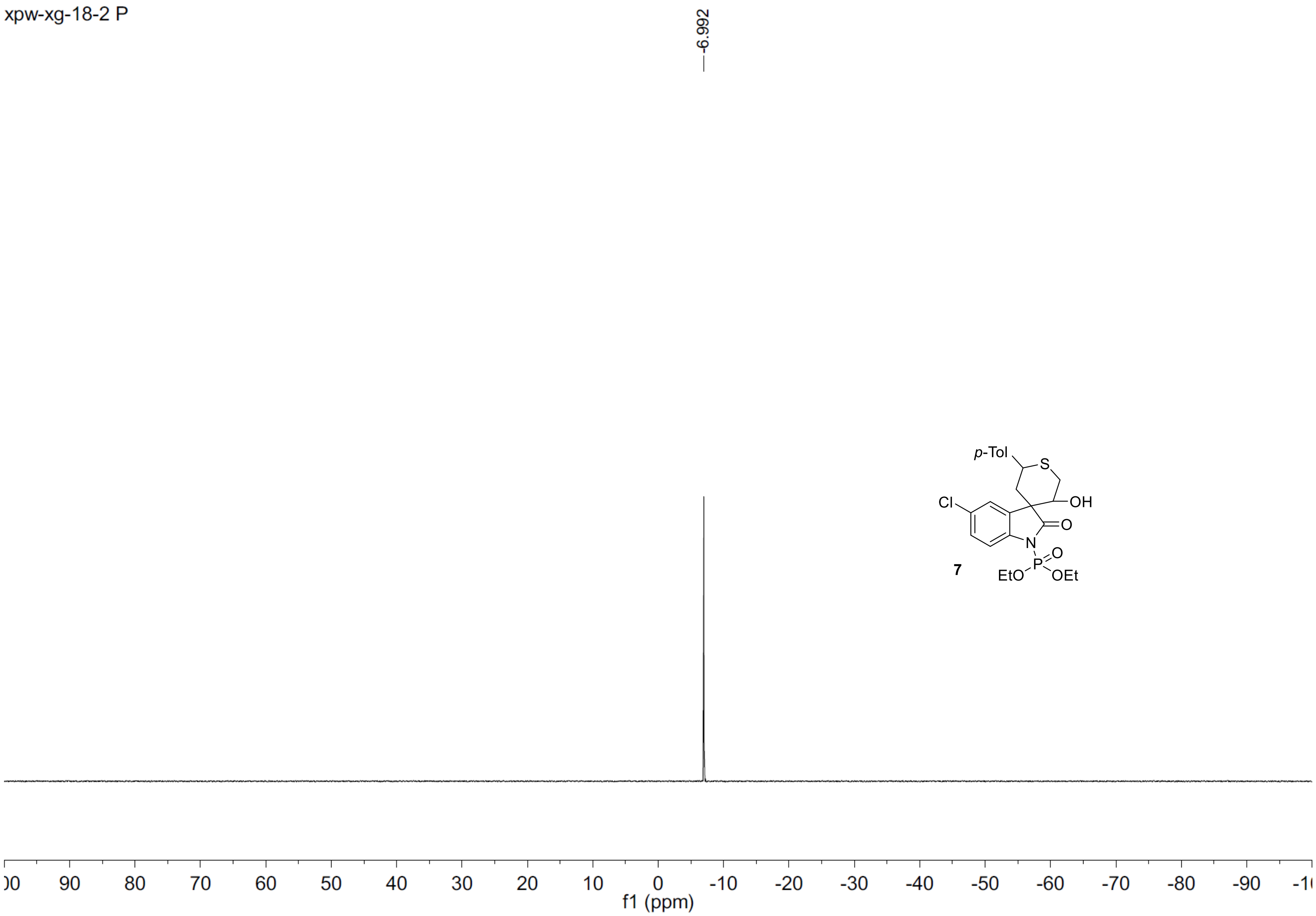

7

Supplementary Figure 152. ^{31}P NMR (162 MHz, $CDCl_3$) spectra for compound **7**

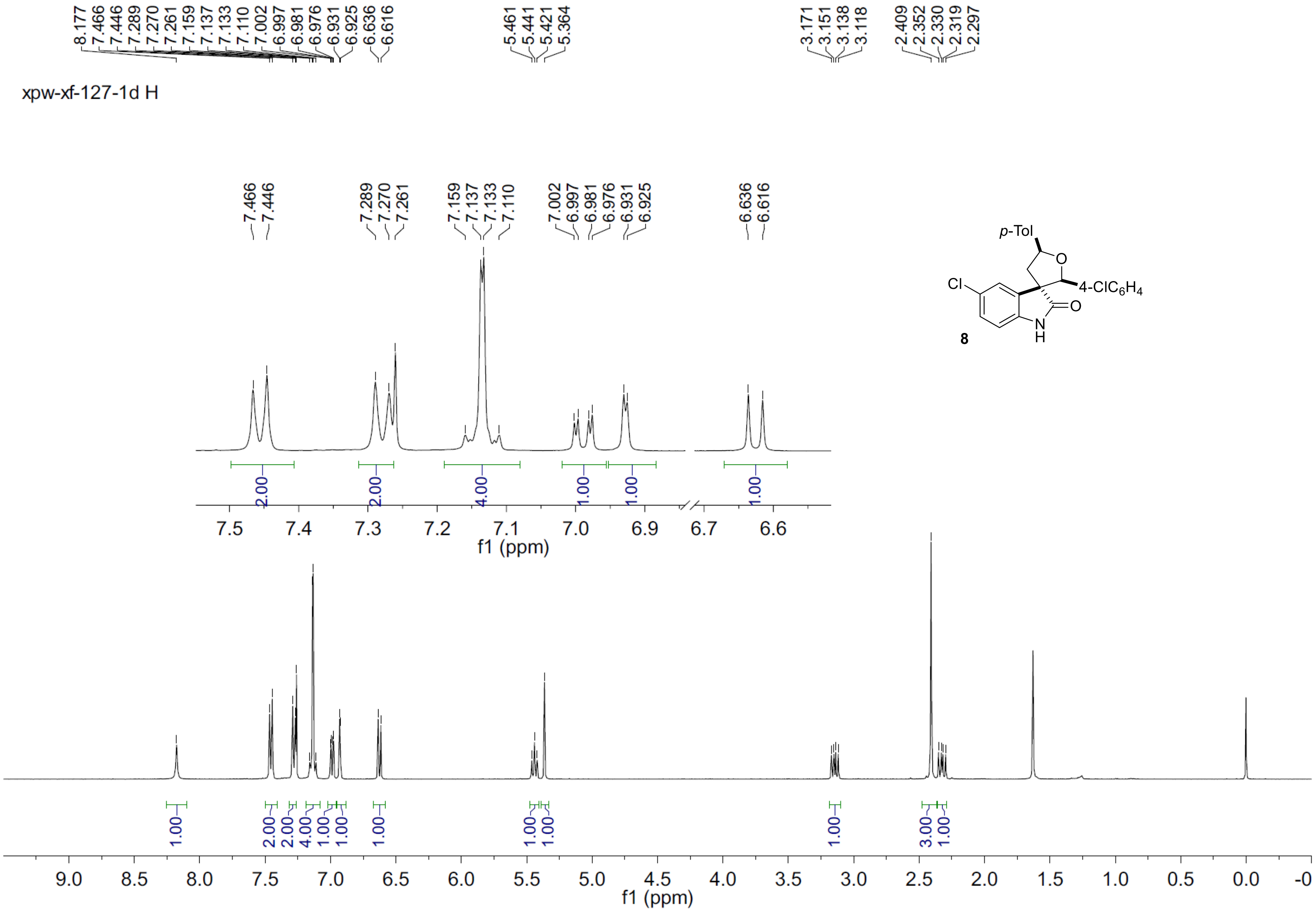

8

Supplementary Figure 153. ^{1}H NMR (400 MHz, $CDCl_3$) spectra for compound **8**

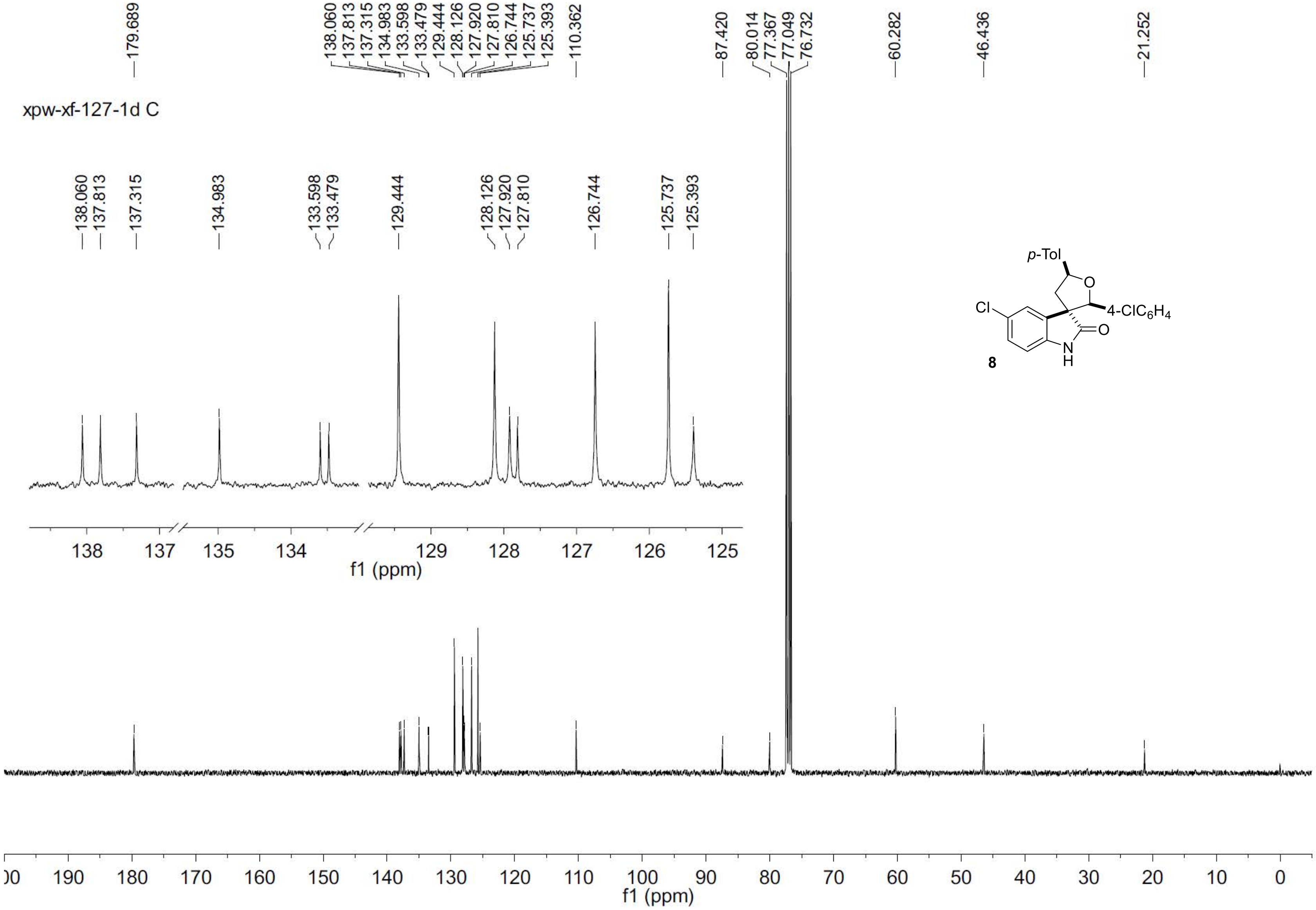

Supplementary Figure 154. ^{13}C NMR (100 MHz, $CDCl_3$) spectra for compound **8**

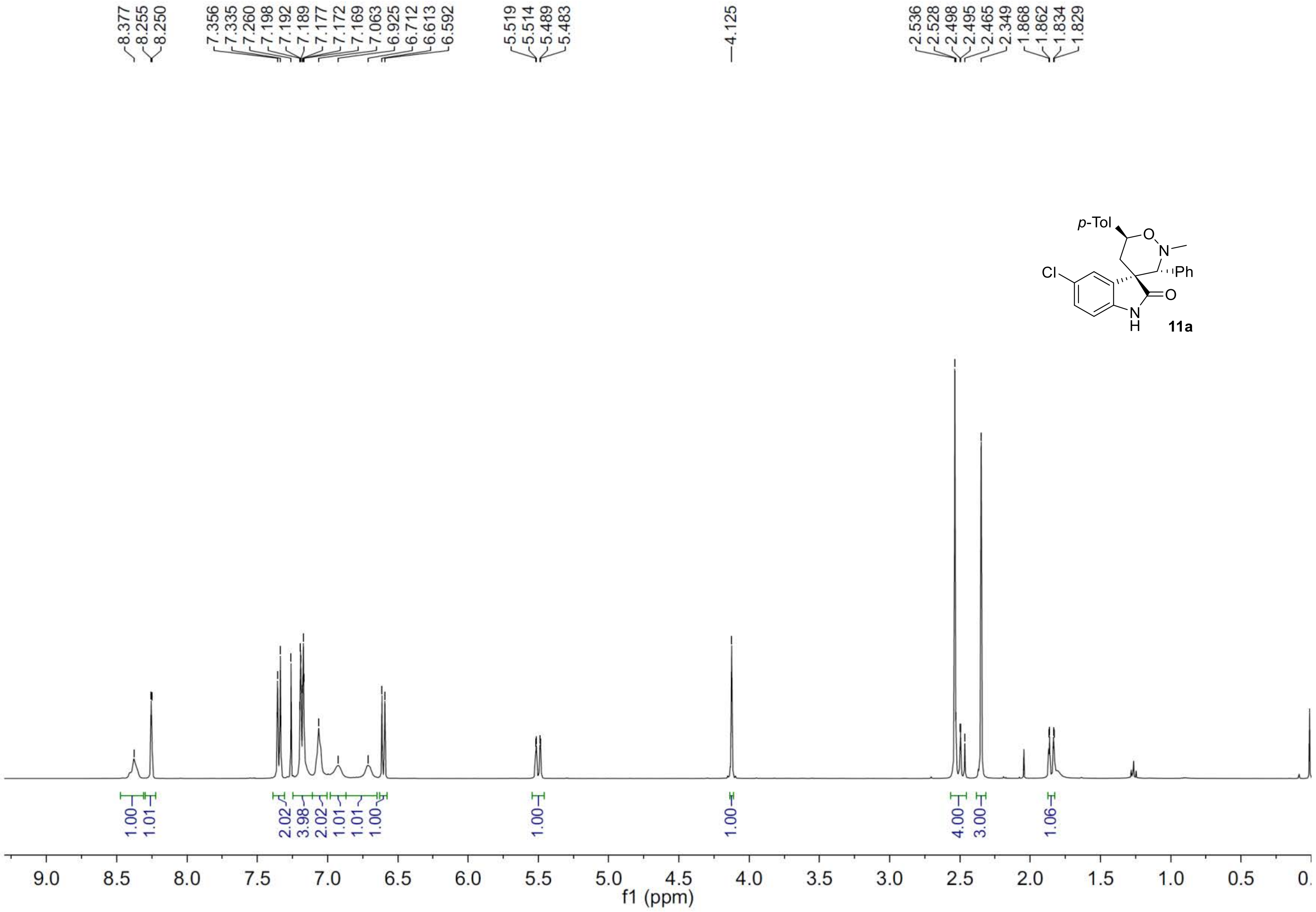

Supplementary Figure 155. ^{1}H NMR (400 MHz, $CDCl_3$) spectra for compound **11a**

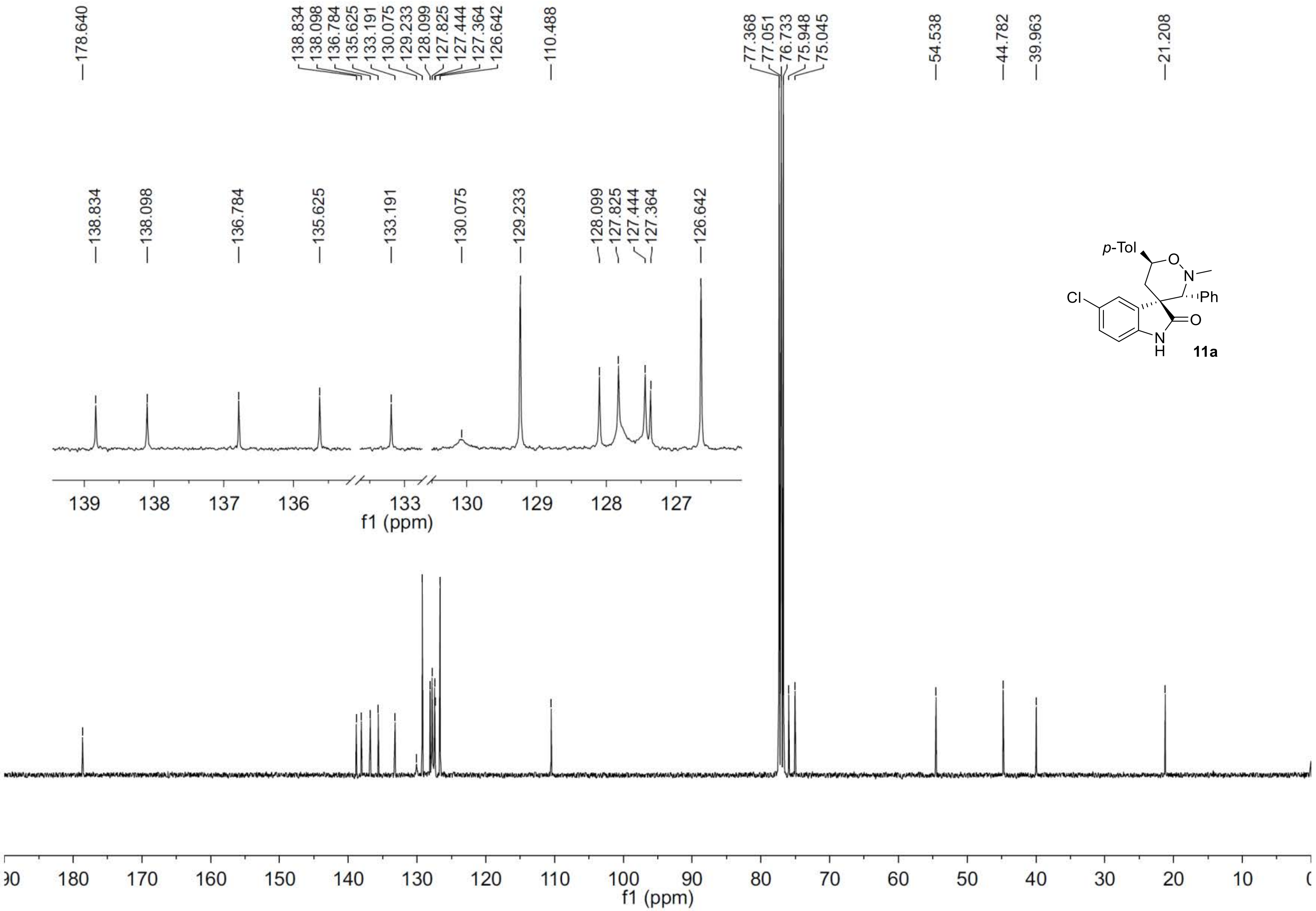

Supplementary Figure 156. ^{13}C NMR (100 MHz, $CDCl_3$) spectra for compound **11a**

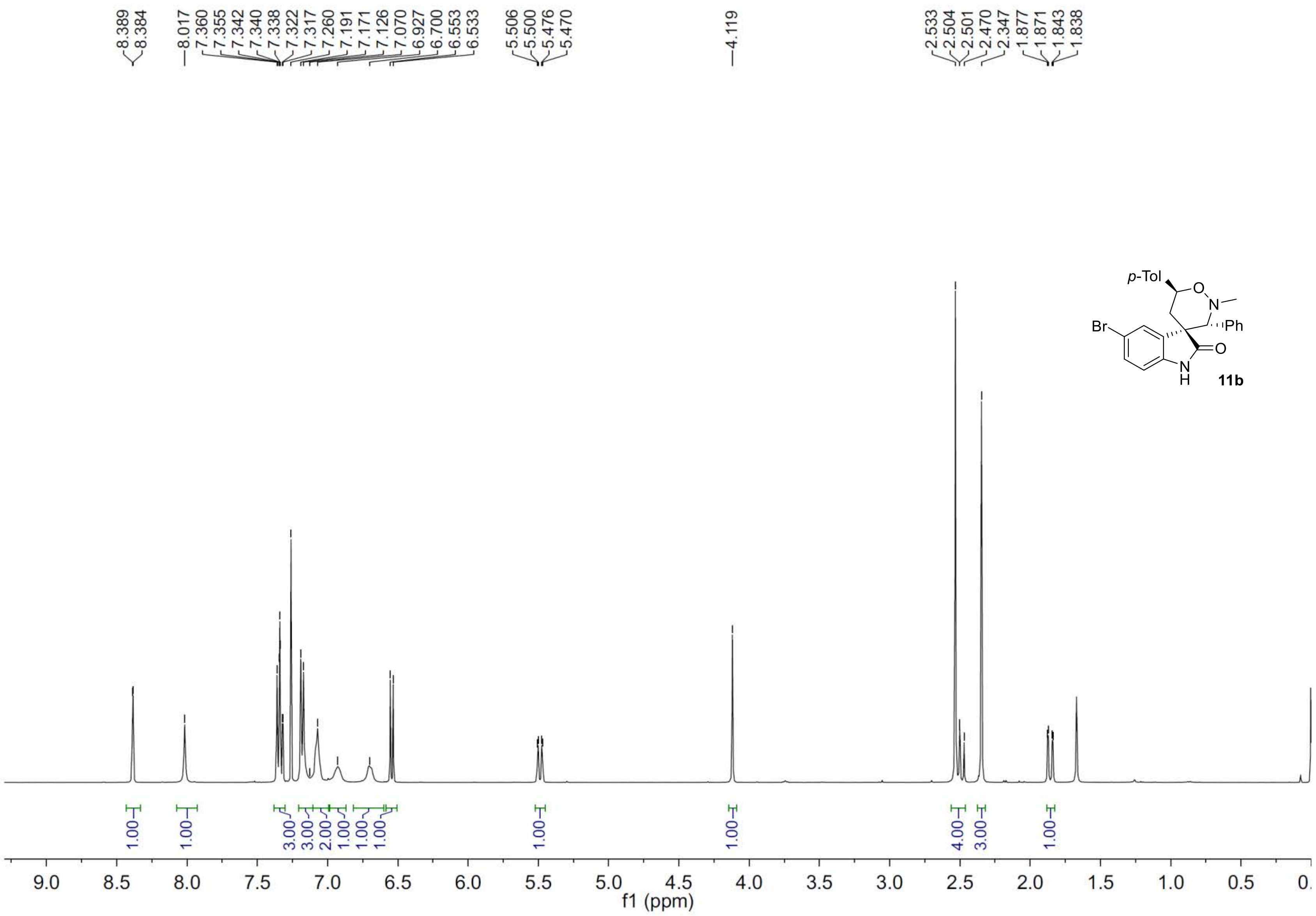

Supplementary Figure 157. ^{1}H NMR (400 MHz, $CDCl_3$) spectra for compound **11b**

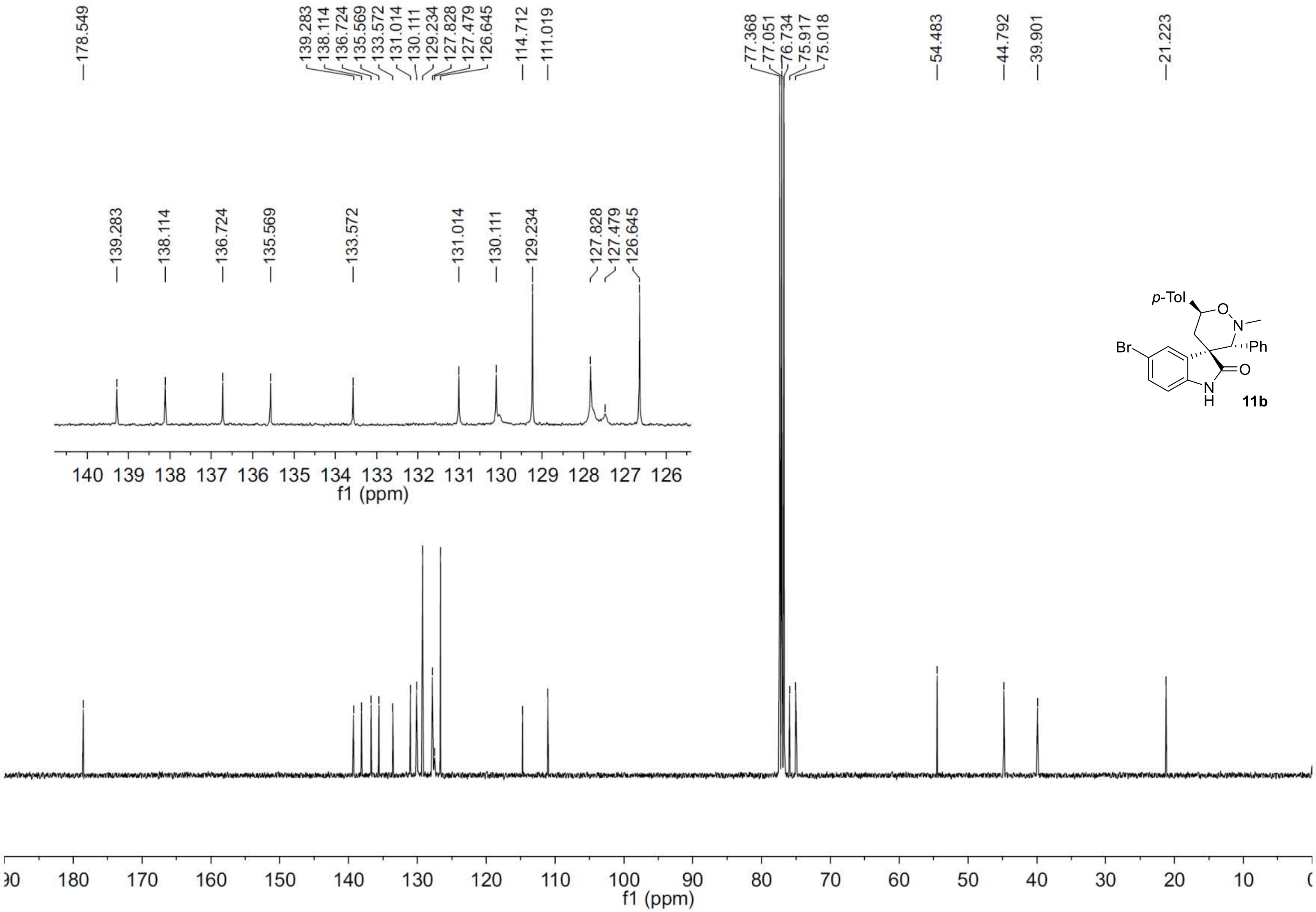

Supplementary Figure 158. ^{13}C NMR (100 MHz, $CDCl_3$) spectra for compound **11b**

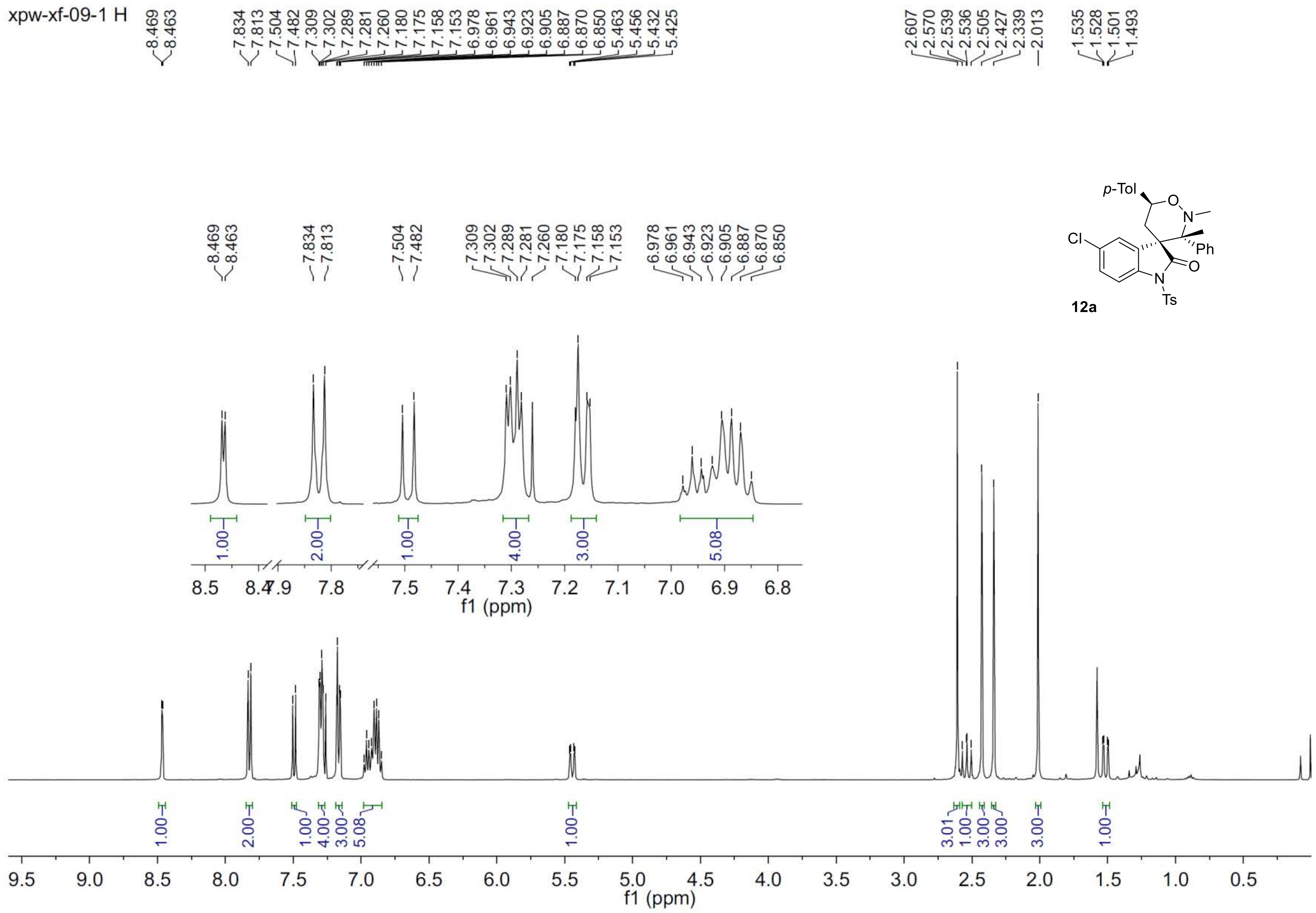

Supplementary Figure 159. ^{1}H NMR (400 MHz, $CDCl_3$) spectra for compound **12a**

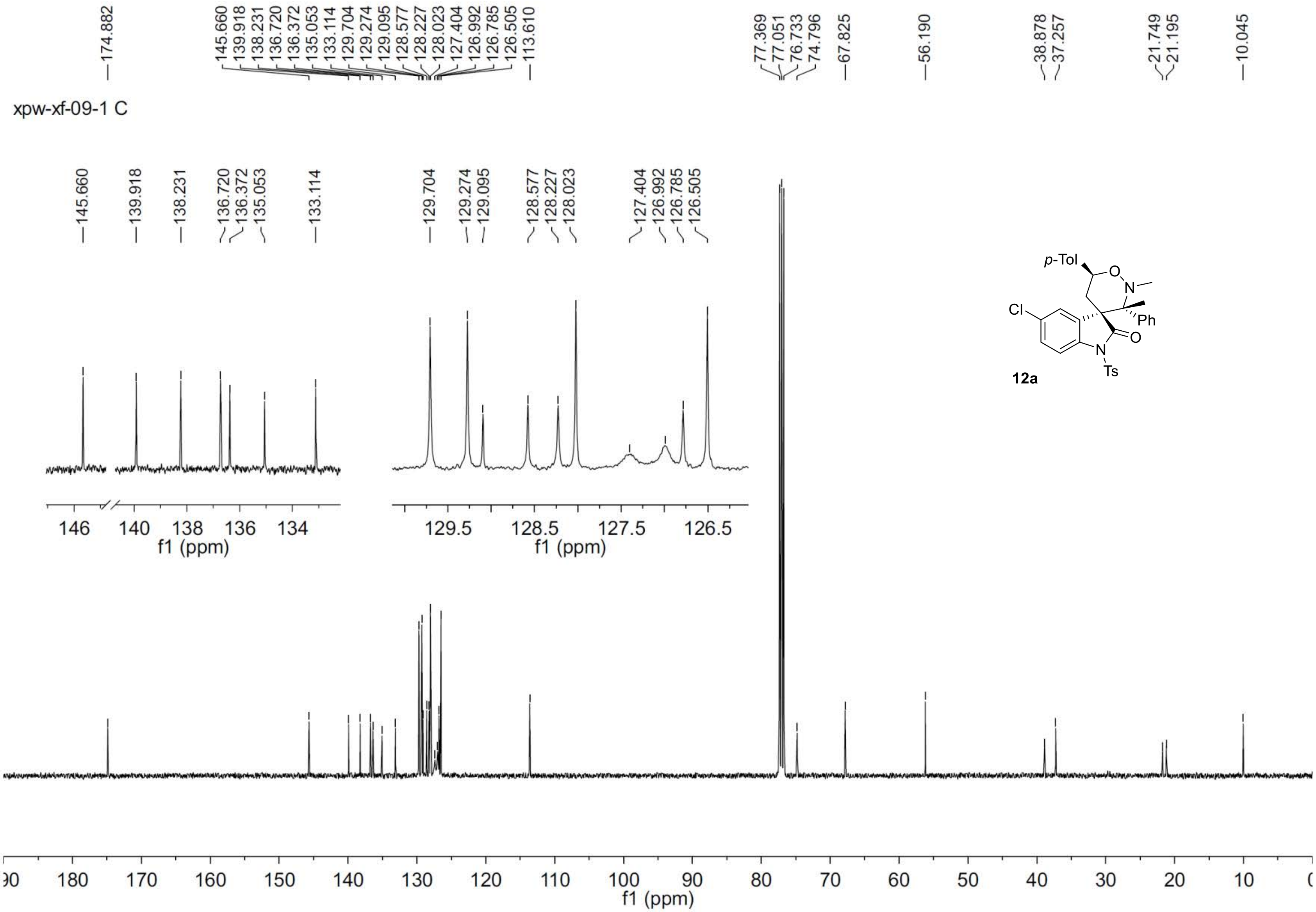

Supplementary Figure 160. ^{13}C NMR (100 MHz, $CDCl_3$) spectra for compound **12a**

HPLC spectra

469 xpw-xc-62-1-asy-ic-80/20-1.0-230

Sample Name:	**xpw-xc-62-1-asy-ic-80/20-1.0-230**	*Injection Volume:*	**20.0**
Vial Number:	**584**	*Channel:*	**UV_VIS_1**
Sample Type:	**standard**	*Wavelength:*	**230**
Control Program:	**Xu Pengwei**	*Bandwidth:*	**n.a.**
Quantif. Method:	**Xu Pengwei**	*Dilution Factor:*	**1.0000**
Recording Time:	**2016-6-14 16:52**	*Sample Weight:*	**1.0000**
Run Time (min):	**23.98**	*Sample Amount:*	**1.0000**

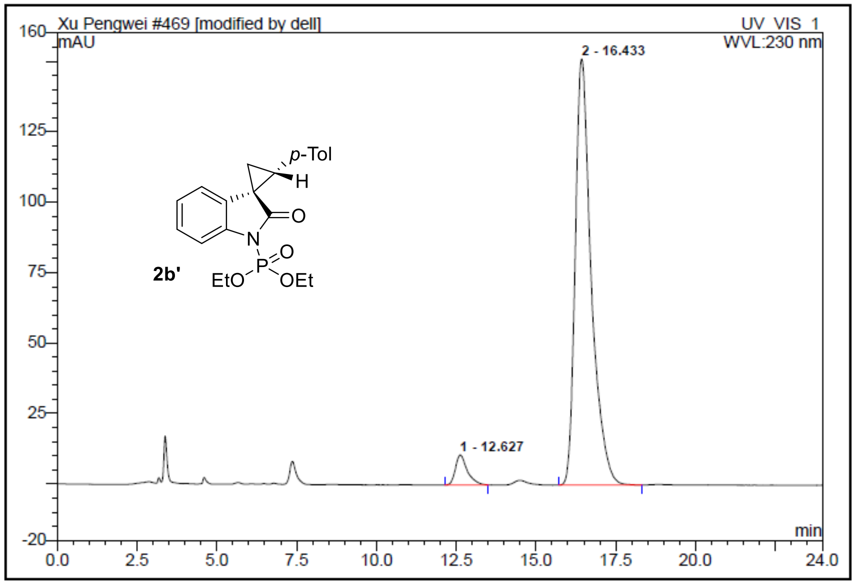

No.	Ret.Time min	Peak Name	Height mAU	Area mAU*min	Rel.Area %	Amount	Type
1	12.63	n.a.	10.644	4.592	4.96	n.a.	BMB
2	16.43	n.a.	151.198	88.021	95.04	n.a.	BMB*
**Total:**			161.842	92.613	100.00	0.000	

470 xpw-xc-62-1-rac-ic-80/20-1.0-230

Sample Name:	**xpw-xc-62-1-rac-ic-80/20-1.0-230**	*Injection Volume:*	**20.0**
Vial Number:	**585**	*Channel:*	**UV_VIS_1**
Sample Type:	**standard**	*Wavelength:*	**230**
Control Program:	**Xu Pengwei**	*Bandwidth:*	**n.a.**
Quantif. Method:	**Xu Pengwei**	*Dilution Factor:*	**1.0000**
Recording Time:	**2016-6-14 17:18**	*Sample Weight:*	**1.0000**
Run Time (min):	**19.93**	*Sample Amount:*	**1.0000**

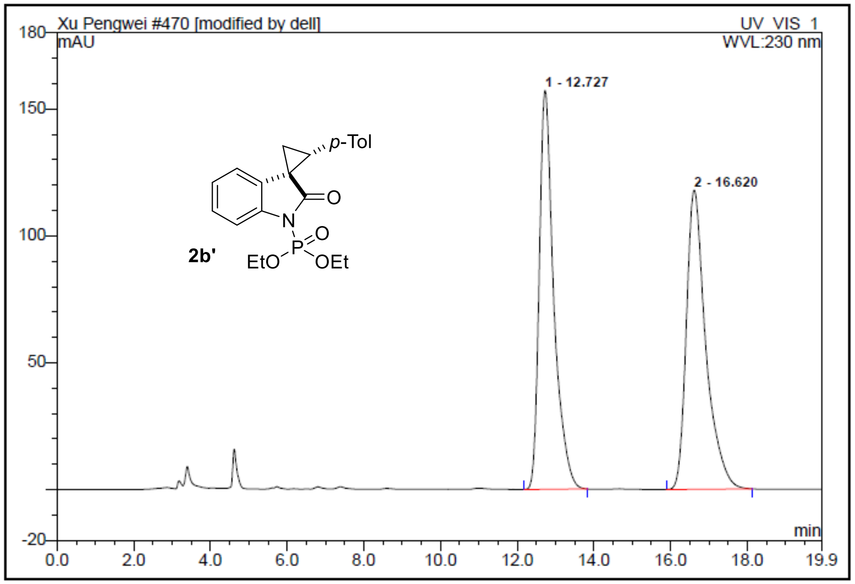

No.	Ret.Time min	Peak Name	Height mAU	Area mAU*min	Rel.Area %	Amount	Type
1	12.73	n.a.	157.239	69.035	49.88	n.a.	BMB*
2	16.62	n.a.	117.890	69.375	50.12	n.a.	BMB
**Total:**			275.129	138.410	100.00	0.000	

Supplementary Figure 161. HPLC analysis for compound **2b'**

478 xpw-xd-50-0-asy-ie-80/20-1.0-230

Sample Name:	**xpw-xd-50-0-asy-ie-80/20-1.0-230**	*Injection Volume:*	**20.0**
Vial Number:	**593**	*Channel:*	**UV_VIS_1**
Sample Type:	**standard**	*Wavelength:*	**230**
Control Program:	**Xu Pengwei**	*Bandwidth:*	**n.a.**
Quantif. Method:	**Xu Pengwei**	*Dilution Factor:*	**1.0000**
Recording Time:	**2016-6-15 19:17**	*Sample Weight:*	**1.0000**
Run Time (min):	**21.95**	*Sample Amount:*	**1.0000**

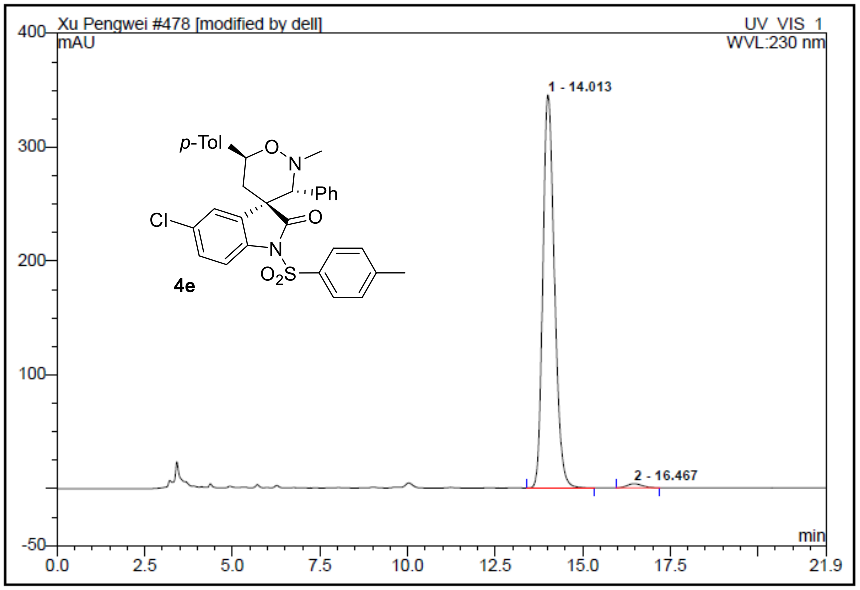

No.	Ret.Time min	Peak Name	Height mAU	Area mAU*min	Rel.Area %	Amount	Type
1	14.01	n.a.	345.396	132.693	98.65	n.a.	BMB*
2	16.47	n.a.	3.622	1.816	1.35	n.a.	BMB*
**Total:**			349.018	134.510	100.00	0.000	

480 xpw-xd-50-0-rac-ie-80/20-1.0-230

Sample Name:	**xpw-xd-50-0-rac-ie-80/20-1.0-230**	*Injection Volume:*	**20.0**
Vial Number:	**595**	*Channel:*	**UV_VIS_1**
Sample Type:	**standard**	*Wavelength:*	**230**
Control Program:	**Xu Pengwei**	*Bandwidth:*	**n.a.**
Quantif. Method:	**Xu Pengwei**	*Dilution Factor:*	**1.0000**
Recording Time:	**2016-6-15 20:08**	*Sample Weight:*	**1.0000**
Run Time (min):	**18.97**	*Sample Amount:*	**1.0000**

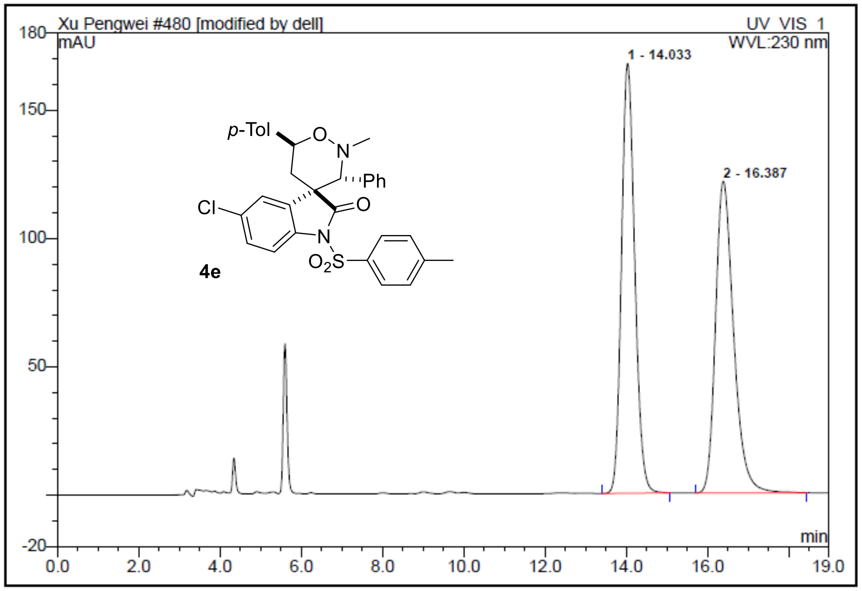

No.	Ret.Time min	Peak Name	Height mAU	Area mAU*min	Rel.Area %	Amount	Type
1	14.03	n.a.	167.510	63.285	50.17	n.a.	BMB
2	16.39	n.a.	121.473	62.856	49.83	n.a.	BMB*
**Total:**			288.983	126.141	100.00	0.000	

Supplementary Figure 162. HPLC analysis for compound **4e**

466 xpw-xd-50-1-asy-ie-80/20-1.0-230

Sample Name:	**xpw-xd-50-1-asy-ie-80/20-1.0-230**	*Injection Volume:*	**20.0**
Vial Number:	**581**	*Channel:*	**UV_VIS_1**
Sample Type:	**standard**	*Wavelength:*	**230**
Control Program:	**Xu Pengwei**	*Bandwidth:*	**n.a.**
Quantif. Method:	**Xu Pengwei**	*Dilution Factor:*	**1.0000**
Recording Time:	**2016-6-14 10:55**	*Sample Weight:*	**1.0000**
Run Time (min):	**20.52**	*Sample Amount:*	**1.0000**

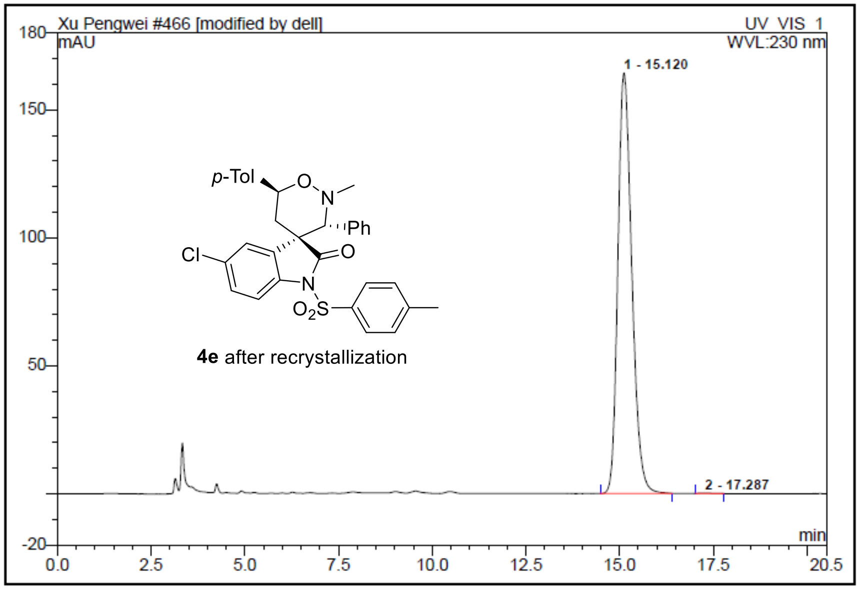

No.	Ret.Time min	Peak Name	Height mAU	Area mAU*min	Rel.Area %	Amount	Type
1	15.12	n.a.	164.317	70.805	99.90	n.a.	BMB*
2	17.29	n.a.	0.177	0.070	0.10	n.a.	BMB*
**Total:**			164.494	70.875	100.00	0.000	

467 xpw-xd-50-1-rac-ie-80/20-1.0-230

Sample Name:	**xpw-xd-50-1-rac-ie-80/20-1.0-230**	*Injection Volume:*	**20.0**
Vial Number:	**582**	*Channel:*	**UV_VIS_1**
Sample Type:	**standard**	*Wavelength:*	**230**
Control Program:	**Xu Pengwei**	*Bandwidth:*	**n.a.**
Quantif. Method:	**Xu Pengwei**	*Dilution Factor:*	**1.0000**
Recording Time:	**2016-6-14 11:16**	*Sample Weight:*	**1.0000**
Run Time (min):	**20.07**	*Sample Amount:*	**1.0000**

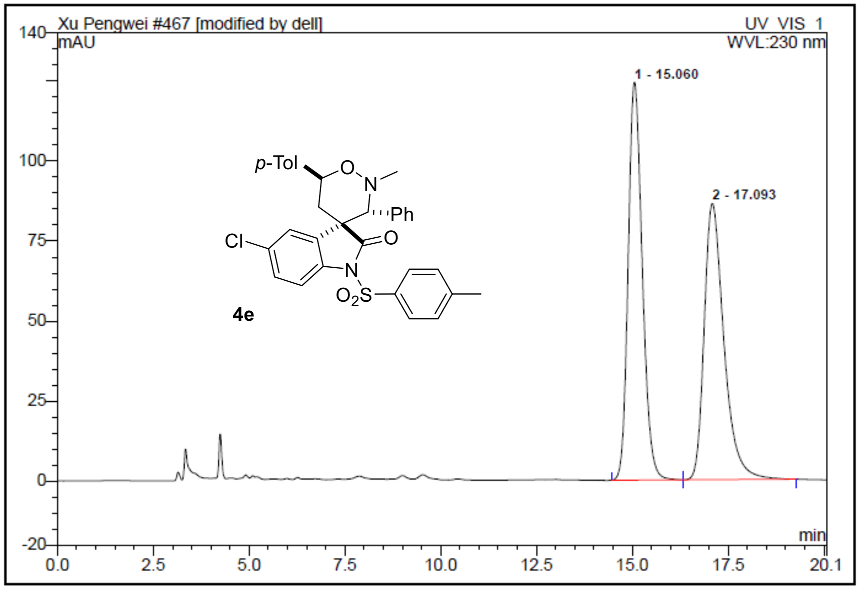

No.	Ret.Time min	Peak Name	Height mAU	Area mAU*min	Rel.Area %	Amount	Type
1	15.06	n.a.	124.350	53.236	50.76	n.a.	BM *
2	17.09	n.a.	86.355	51.639	49.24	n.a.	MB*
**Total:**			210.705	104.876	100.00	0.000	

Supplementary Figure 163. HPLC analysis for compound **4e** (after recrystallization)

106 xpw-xc-146-1p-asy-ic-90/10-1.0-230

Sample Name:	**xpw-xc-146-1p-asy-ic-90/10-1.0-230**	*Injection Volume:*	**20.0**
Vial Number:	**203**	*Channel:*	**UV_VIS_1**
Sample Type:	**standard**	*Wavelength:*	**230**
Control Program:	**Xu Pengwei**	*Bandwidth:*	**n.a.**
Quantif. Method:	**Xu Pengwei**	*Dilution Factor:*	**1.0000**
Recording Time:	**2015-12-3 11:19**	*Sample Weight:*	**1.0000**
Run Time (min):	**19.78**	*Sample Amount:*	**1.0000**

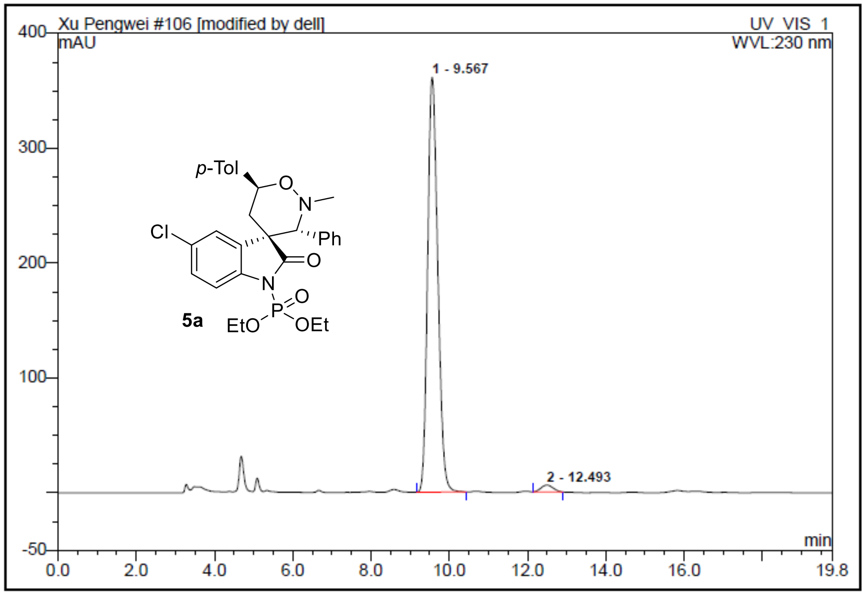

No.	Ret.Time min	Peak Name	Height mAU	Area mAU*min	Rel.Area %	Amount	Type
1	9.57	n.a.	361.352	108.557	98.10	n.a.	BMB*
2	12.49	n.a.	5.931	2.105	1.90	n.a.	BMB*
Total:			367.283	110.662	100.00	0.000	

107 xpw-xc-146-1p-rac-ic-90/10-1.0-230

Sample Name:	**xpw-xc-146-1p-rac-ic-90/10-1.0-230**	*Injection Volume:*	**20.0**
Vial Number:	**204**	*Channel:*	**UV_VIS_1**
Sample Type:	**standard**	*Wavelength:*	**230**
Control Program:	**Xu Pengwei**	*Bandwidth:*	**n.a.**
Quantif. Method:	**Xu Pengwei**	*Dilution Factor:*	**1.0000**
Recording Time:	**2015-12-3 11:41**	*Sample Weight:*	**1.0000**
Run Time (min):	**15.59**	*Sample Amount:*	**1.0000**

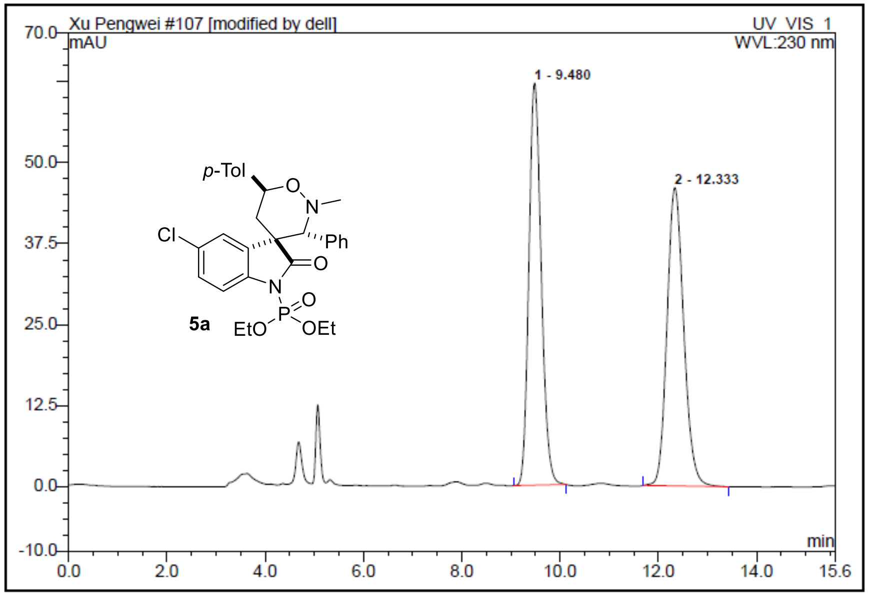

No.	Ret.Time min	Peak Name	Height mAU	Area mAU*min	Rel.Area %	Amount	Type
1	9.48	n.a.	62.131	18.068	49.90	n.a.	BMB*
2	12.33	n.a.	46.066	18.139	50.10	n.a.	BMB*
Total:			108.197	36.207	100.00	0.000	

Supplementary Figure 164. HPLC analysis for compound **5a**

110 xpw-xc-146-1s-asy-ic-90/10-1.0-230

Sample Name:	xpw-xc-146-1s-asy-ic-90/10-1.0-230	*Injection Volume:*	20.0
Vial Number:	207	*Channel:*	UV_VIS_1
Sample Type:	standard	*Wavelength:*	230
Control Program:	Xu Pengwei	*Bandwidth:*	n.a.
Quantif. Method:	Xu Pengwei	*Dilution Factor:*	1.0000
Recording Time:	2015-12-3 18:30	*Sample Weight:*	1.0000
Run Time (min):	22.94	*Sample Amount:*	1.0000

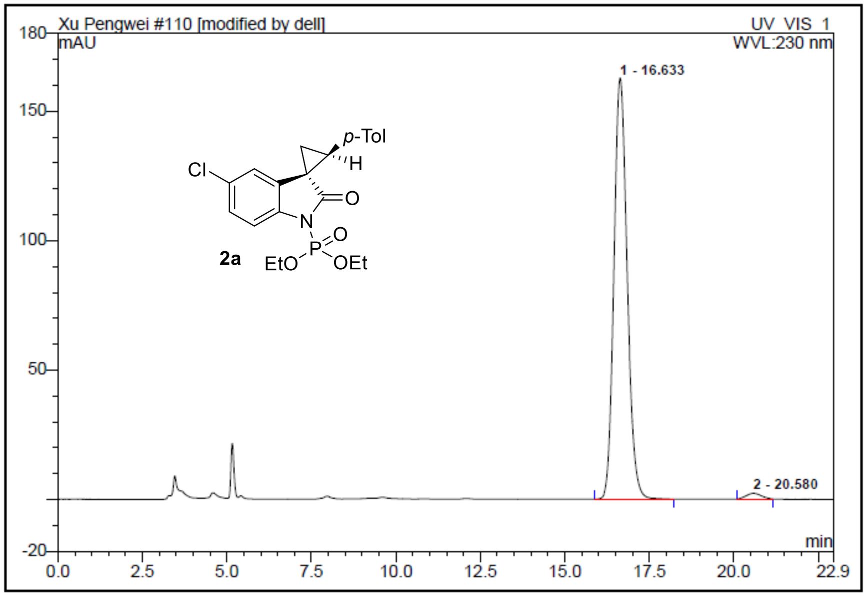

No.	Ret.Time min	Peak Name	Height mAU	Area mAU*min	Rel.Area %	Amount	Type
1	16.63	n.a.	162.642	73.645	98.50	n.a.	BMB*
2	20.58	n.a.	2.174	1.125	1.50	n.a.	BMB*
Total:			164.817	74.770	100.00	0.000	

111 xpw-xc-146-1s-rac-ic-90/10-1.0-230

Sample Name:	xpw-xc-146-1s-rac-ic-90/10-1.0-230	*Injection Volume:*	20.0
Vial Number:	208	*Channel:*	UV_VIS_1
Sample Type:	standard	*Wavelength:*	230
Control Program:	Xu Pengwei	*Bandwidth:*	n.a.
Quantif. Method:	Xu Pengwei	*Dilution Factor:*	1.0000
Recording Time:	2015-12-3 18:55	*Sample Weight:*	1.0000
Run Time (min):	22.65	*Sample Amount:*	1.0000

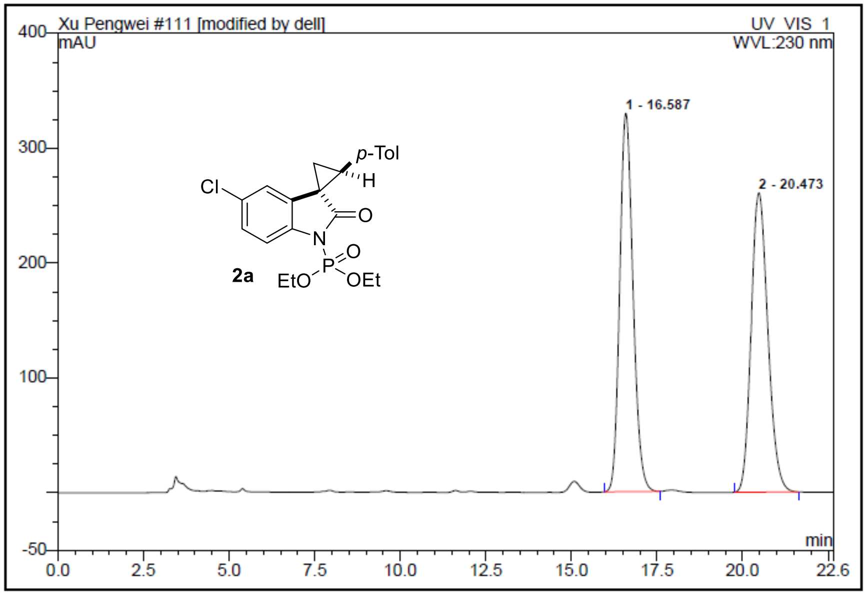

No.	Ret.Time min	Peak Name	Height mAU	Area mAU*min	Rel.Area %	Amount	Type
1	16.59	n.a.	329.556	148.902	50.35	n.a.	BMB*
2	20.47	n.a.	260.618	146.861	49.65	n.a.	BMB*
Total:			590.174	295.762	100.00	0.000	

Supplementary Figure 165. HPLC analysis for compound **2a**

205 xpw-xd-51-1p-asy-ic-90/10-1.0-230			
Sample Name:	xpw-xd-51-1p-asy-ic-90/10-1.0-230	Injection Volume:	20.0
Vial Number:	309	Channel:	UV_VIS_1
Sample Type:	standard	Wavelength:	230
Control Program:	Xu Pengwei	Bandwidth:	n.a.
Quantif. Method:	Xu Pengwei	Dilution Factor:	1.0000
Recording Time:	2016-1-15 20:17	Sample Weight:	1.0000
Run Time (min):	18.92	Sample Amount:	1.0000

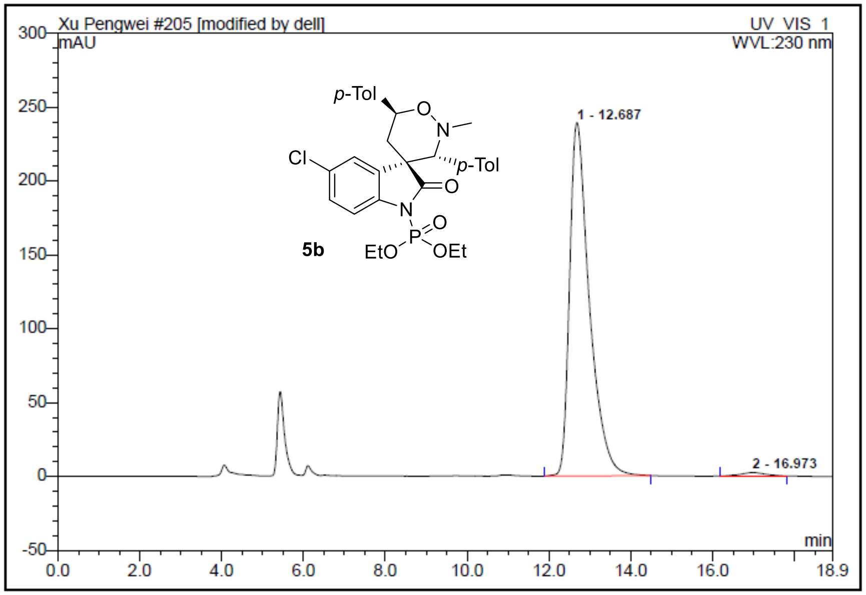

No.	Ret.Time min	Peak Name	Height mAU	Area mAU*min	Rel.Area %	Amount	Type
1	12.69	n.a.	239.316	133.923	98.79	n.a.	BMB*
2	16.97	n.a.	2.309	1.641	1.21	n.a.	BMB*
Total:			241.625	135.564	100.00	0.000	

206 xpw-xd-51-1p-rac-ic-90/10-1.0-230			
Sample Name:	xpw-xd-51-1p-rac-ic-90/10-1.0-230	Injection Volume:	20.0
Vial Number:	310	Channel:	UV_VIS_1
Sample Type:	standard	Wavelength:	230
Control Program:	Xu Pengwei	Bandwidth:	n.a.
Quantif. Method:	Xu Pengwei	Dilution Factor:	1.0000
Recording Time:	2016-1-15 20:59	Sample Weight:	1.0000
Run Time (min):	19.69	Sample Amount:	1.0000

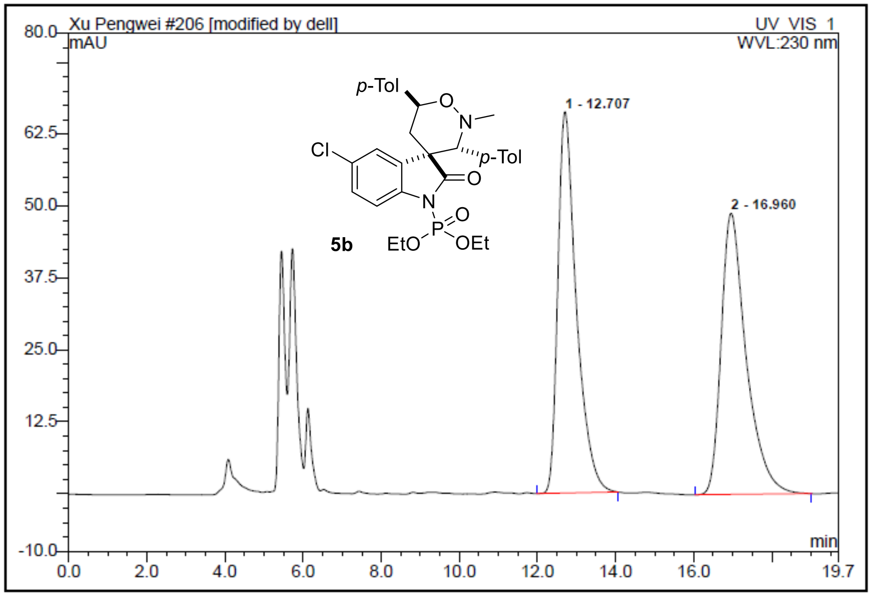

No.	Ret.Time min	Peak Name	Height mAU	Area mAU*min	Rel.Area %	Amount	Type
1	12.71	n.a.	66.282	36.519	50.13	n.a.	BMB*
2	16.96	n.a.	48.859	36.335	49.87	n.a.	BMB*
Total:			115.141	72.854	100.00	0.000	

Supplementary Figure 166. HPLC analysis for compound **5b**

131 xpw-xd-11-1p-asy-ic-90/10-1.0-230

Sample Name:	xpw-xd-11-1p-asy-ic-90/10-1.0-230	Injection Volume:	20.0
Vial Number:	233	Channel:	UV_VIS_1
Sample Type:	standard	Wavelength:	230
Control Program:	Xu Pengwei	Bandwidth:	n.a.
Quantif. Method:	Xu Pengwei	Dilution Factor:	1.0000
Recording Time:	2015-12-16 19:17	Sample Weight:	1.0000
Run Time (min):	19.45	Sample Amount:	1.0000

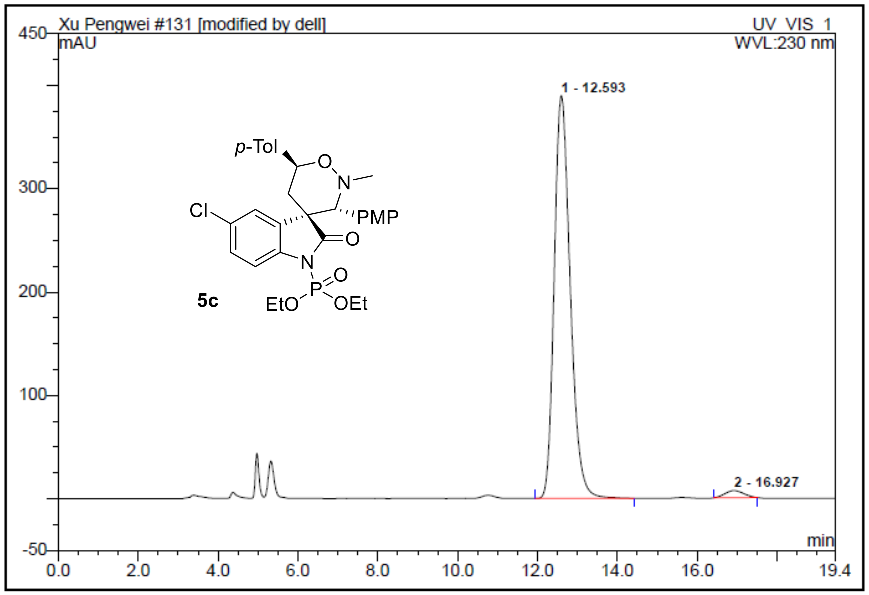

No.	Ret.Time min	Peak Name	Height mAU	Area mAU*min	Rel.Area %	Amount	Type
1	12.59	n.a.	389.586	181.217	97.96	n.a.	BMB*
2	16.93	n.a.	6.834	3.770	2.04	n.a.	BMB*
Total:			396.420	184.987	100.00	0.000	

132 xpw-xd-11-1p-rac-ic-90/10-1.0-230

Sample Name:	xpw-xd-11-1p-rac-ic-90/10-1.0-230	Injection Volume:	20.0
Vial Number:	234	Channel:	UV_VIS_1
Sample Type:	standard	Wavelength:	230
Control Program:	Xu Pengwei	Bandwidth:	n.a.
Quantif. Method:	Xu Pengwei	Dilution Factor:	1.0000
Recording Time:	2015-12-16 19:37	Sample Weight:	1.0000
Run Time (min):	20.65	Sample Amount:	1.0000

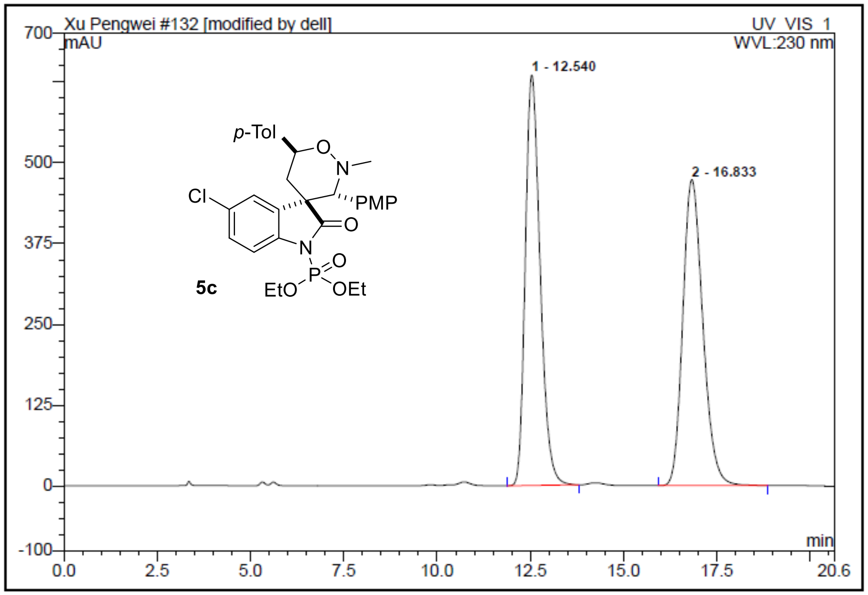

No.	Ret.Time min	Peak Name	Height mAU	Area mAU*min	Rel.Area %	Amount	Type
1	12.54	n.a.	634.827	292.624	49.82	n.a.	BMB*
2	16.83	n.a.	473.317	294.711	50.18	n.a.	BMB*
Total:			1108.144	587.335	100.00	0.000	

Supplementary Figure 167. HPLC analysis for compound **5c**

133 xpw-xd-12-1p-asy-ic-90/10-1.0-230			
Sample Name:	**xpw-xd-12-1p-asy-ic-90/10-1.0-230**	*Injection Volume:*	**20.0**
Vial Number:	**235**	*Channel:*	**UV_VIS_1**
Sample Type:	**standard**	*Wavelength:*	**230**
Control Program:	**Xu Pengwei**	*Bandwidth:*	**n.a.**
Quantif. Method:	**Xu Pengwei**	*Dilution Factor:*	**1.0000**
Recording Time:	**2015-12-16 19:59**	*Sample Weight:*	**1.0000**
Run Time (min):	**15.93**	*Sample Amount:*	**1.0000**

134 xpw-xd-12-1p-rac-ic-90/10-1.0-230			
Sample Name:	**xpw-xd-12-1p-rac-ic-90/10-1.0-230**	*Injection Volume:*	**20.0**
Vial Number:	**236**	*Channel:*	**UV_VIS_1**
Sample Type:	**standard**	*Wavelength:*	**230**
Control Program:	**Xu Pengwei**	*Bandwidth:*	**n.a.**
Quantif. Method:	**Xu Pengwei**	*Dilution Factor:*	**1.0000**
Recording Time:	**2015-12-16 20:16**	*Sample Weight:*	**1.0000**
Run Time (min):	**15.14**	*Sample Amount:*	**1.0000**

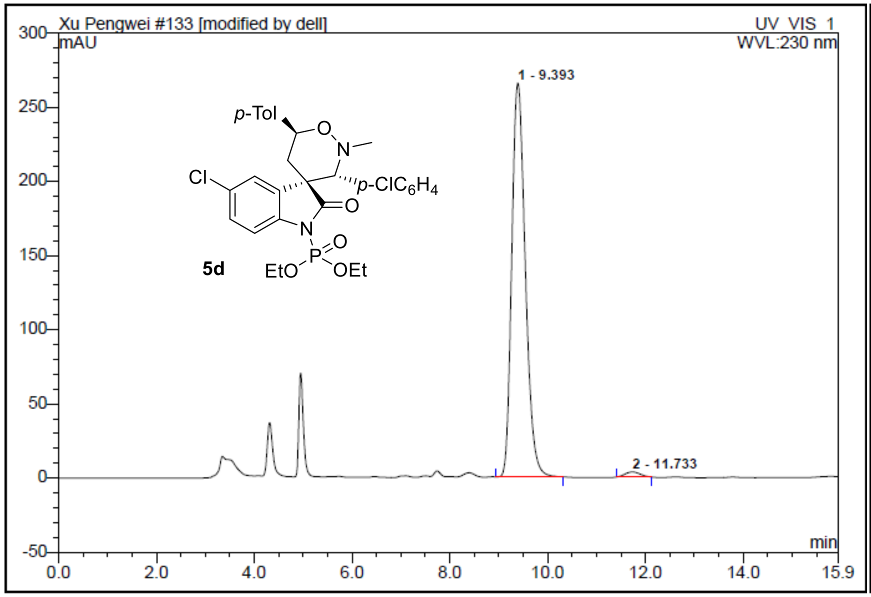

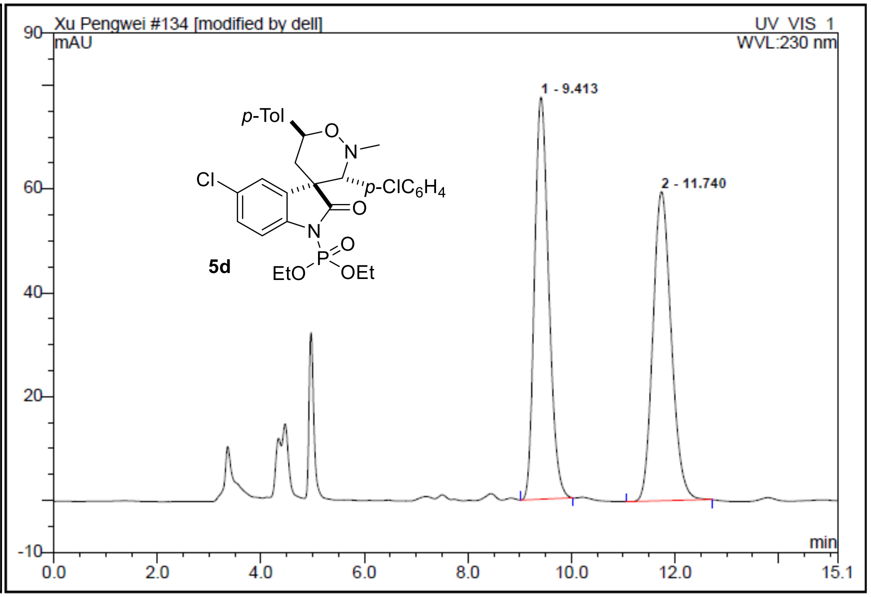

No.	Ret.Time min	Peak Name	Height mAU	Area mAU*min	Rel.Area %	Amount	Type
1	9.39	n.a.	265.638	85.363	98.59	n.a.	BMB*
2	11.73	n.a.	3.436	1.223	1.41	n.a.	BMB*
**Total:**			269.074	86.587	100.00	0.000	

No.	Ret.Time min	Peak Name	Height mAU	Area mAU*min	Rel.Area %	Amount	Type
1	9.41	n.a.	77.470	24.557	50.08	n.a.	BMB*
2	11.74	n.a.	59.577	24.481	49.92	n.a.	BMB*
**Total:**			137.047	49.038	100.00	0.000	

Supplementary Figure 168. HPLC analysis for compound **5d**

149 xpw-xd-21-1p-asy-ic-90/10-1.0-230

Sample Name:	xpw-xd-21-1p-asy-ic-90/10-1.0-230	*Injection Volume:*	20.0
Vial Number:	251	*Channel:*	UV_VIS_1
Sample Type:	standard	*Wavelength:*	230
Control Program:	Xu Pengwei	*Bandwidth:*	n.a.
Quantif. Method:	Xu Pengwei	*Dilution Factor:*	1.0000
Recording Time:	2015-12-29 21:52	*Sample Weight:*	1.0000
Run Time (min):	15.16	*Sample Amount:*	1.0000

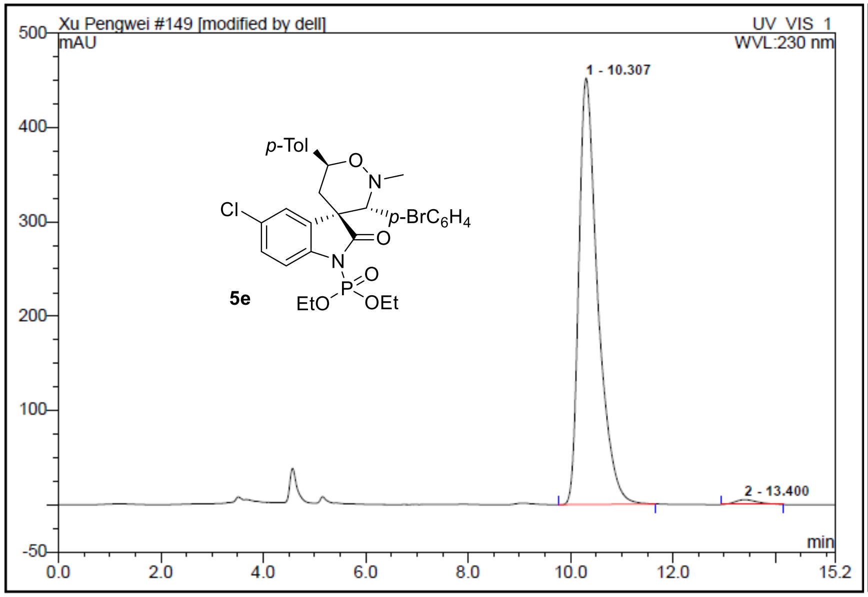

No.	Ret.Time min	Peak Name	Height mAU	Area mAU*min	Rel.Area %	Amount	Type
1	10.31	n.a.	452.264	193.288	98.66	n.a.	BMB*
2	13.40	n.a.	5.072	2.616	1.34	n.a.	BMB*
Total:			457.336	195.904	100.00	0.000	

150 xpw-xd-21-1p-rac-ic-90/10-1.0-230

Sample Name:	xpw-xd-21-1p-rac-ic-90/10-1.0-230	*Injection Volume:*	20.0
Vial Number:	252	*Channel:*	UV_VIS_1
Sample Type:	standard	*Wavelength:*	230
Control Program:	Xu Pengwei	*Bandwidth:*	n.a.
Quantif. Method:	Xu Pengwei	*Dilution Factor:*	1.0000
Recording Time:	2015-12-29 22:08	*Sample Weight:*	1.0000
Run Time (min):	16.11	*Sample Amount:*	1.0000

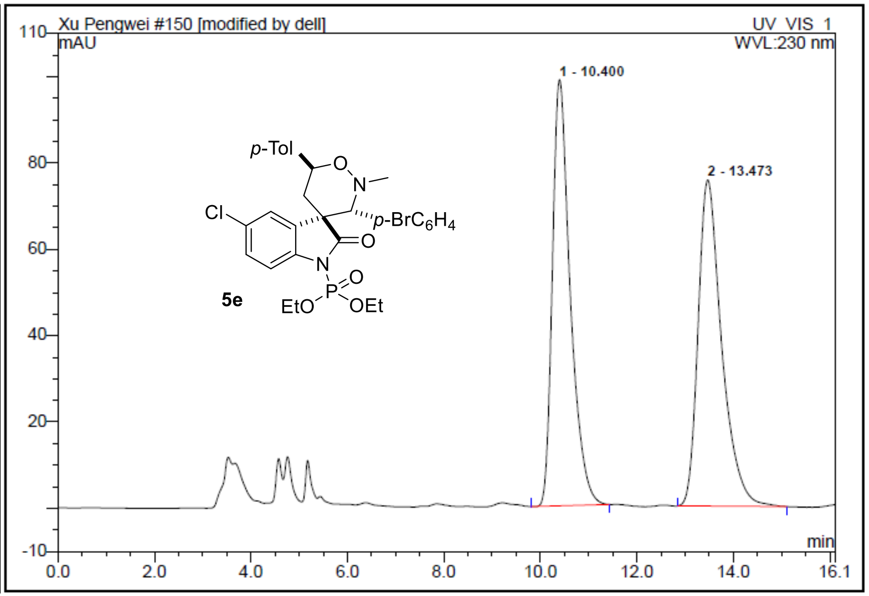

No.	Ret.Time min	Peak Name	Height mAU	Area mAU*min	Rel.Area %	Amount	Type
1	10.40	n.a.	98.749	42.356	49.72	n.a.	BMB*
2	13.47	n.a.	75.552	42.833	50.28	n.a.	BMB*
Total:			174.301	85.189	100.00	0.000	

Supplementary Figure 169. HPLC analysis for compound **5e**

196 xpw-xd-49-1p-asy-ic-90/10-1.0-230

Sample Name:	**xpw-xd-49-1p-asy-ic-90/10-1.0-230**	*Injection Volume:*	**20.0**
Vial Number:	**299**	*Channel:*	**UV_VIS_1**
Sample Type:	**standard**	*Wavelength:*	**230**
Control Program:	**Xu Pengwei**	*Bandwidth:*	**n.a.**
Quantif. Method:	**Xu Pengwei**	*Dilution Factor:*	**1.0000**
Recording Time:	**2016-1-15 11:28**	*Sample Weight:*	**1.0000**
Run Time (min):	**16.51**	*Sample Amount:*	**1.0000**

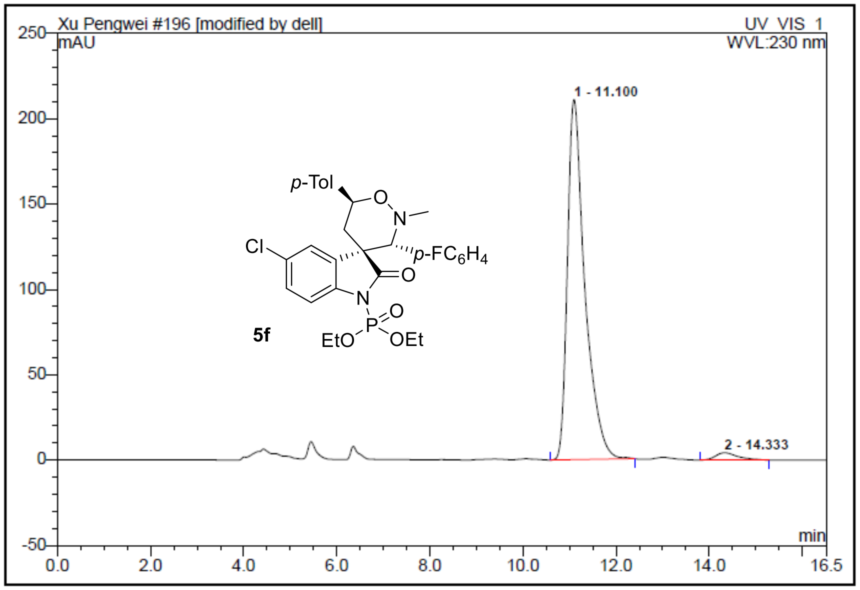

No.	Ret.Time min	Peak Name	Height mAU	Area mAU*min	Rel.Area %	Amount	Type
1	11.10	n.a.	210.866	91.360	97.51	n.a.	BMB*
2	14.33	n.a.	4.272	2.336	2.49	n.a.	BMB*
**Total:**			215.138	93.695	100.00	0.000	

197 xpw-xd-49-1p-rac-ic-90/10-1.0-230

Sample Name:	**xpw-xd-49-1p-rac-ic-90/10-1.0-230**	*Injection Volume:*	**20.0**
Vial Number:	**300**	*Channel:*	**UV_VIS_1**
Sample Type:	**standard**	*Wavelength:*	**230**
Control Program:	**Xu Pengwei**	*Bandwidth:*	**n.a.**
Quantif. Method:	**Xu Pengwei**	*Dilution Factor:*	**1.0000**
Recording Time:	**2016-1-15 11:45**	*Sample Weight:*	**1.0000**
Run Time (min):	**16.72**	*Sample Amount:*	**1.0000**

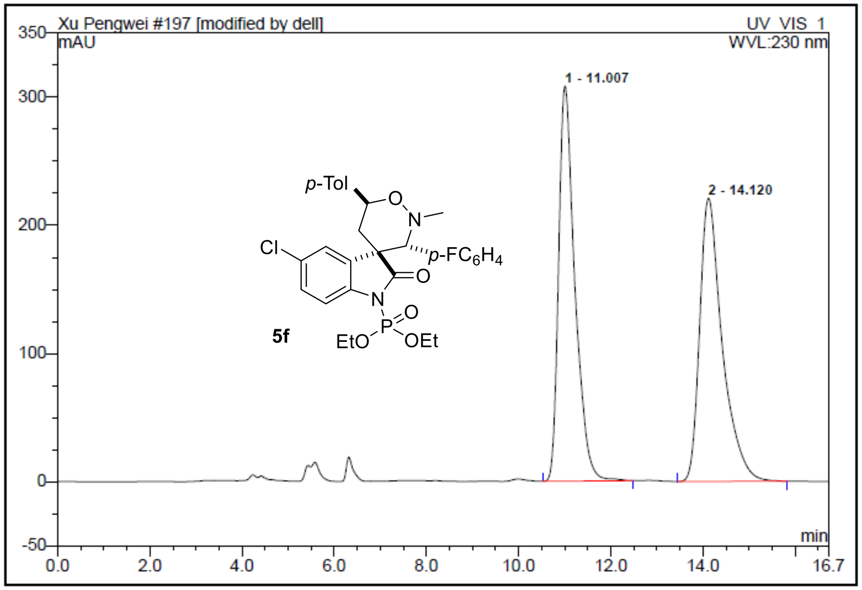

No.	Ret.Time min	Peak Name	Height mAU	Area mAU*min	Rel.Area %	Amount	Type
1	11.01	n.a.	308.252	125.922	50.08	n.a.	BMB*
2	14.12	n.a.	220.912	125.503	49.92	n.a.	BMB*
**Total:**			529.164	251.426	100.00	0.000	

Supplementary Figure 170. HPLC analysis for compound **5f**

391 xpw-xe-11-1p-asy-ie-85/15-1.0-230			
Sample Name:	xpw-xe-11-1p-asy-ie-85/15-1.0-230	*Injection Volume:*	20.0
Vial Number:	506	*Channel:*	UV_VIS_1
Sample Type:	standard	*Wavelength:*	230
Control Program:	Xu Pengwei	*Bandwidth:*	n.a.
Quantif. Method:	Xu Pengwei	*Dilution Factor:*	1.0000
Recording Time:	2016-4-15 16:00	*Sample Weight:*	1.0000
Run Time (min):	22.23	*Sample Amount:*	1.0000

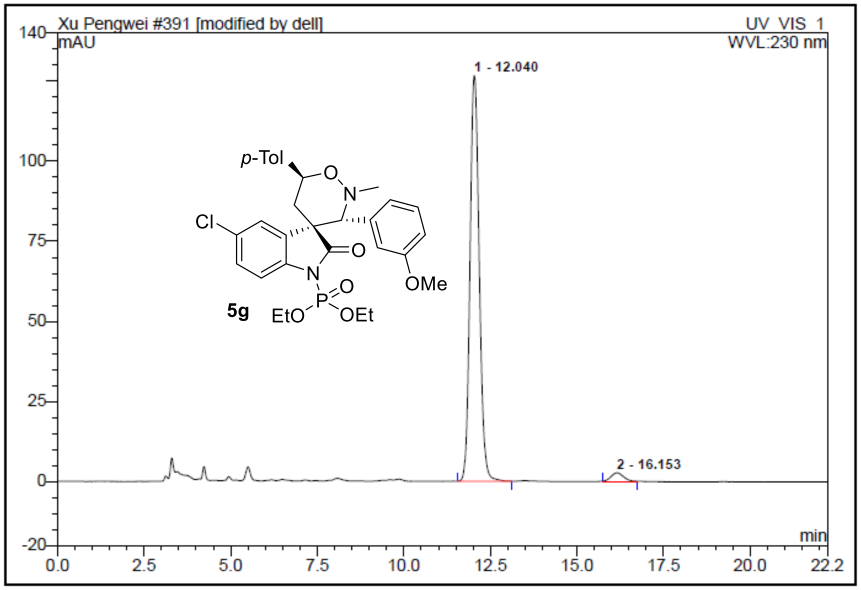

No.	Ret.Time min	Peak Name	Height mAU	Area mAU*min	Rel.Area %	Amount	Type
1	12.04	n.a.	126.682	39.024	97.30	n.a.	BMB*
2	16.15	n.a.	2.641	1.083	2.70	n.a.	BMB*
**Total:**			129.323	40.107	100.00	0.000	

392 xpw-xe-11-1p-rac-ie-85/15-1.0-230			
Sample Name:	xpw-xe-11-1p-rac-ie-85/15-1.0-230	*Injection Volume:*	20.0
Vial Number:	507	*Channel:*	UV_VIS_1
Sample Type:	standard	*Wavelength:*	230
Control Program:	Xu Pengwei	*Bandwidth:*	n.a.
Quantif. Method:	Xu Pengwei	*Dilution Factor:*	1.0000
Recording Time:	2016-4-15 16:23	*Sample Weight:*	1.0000
Run Time (min):	17.95	*Sample Amount:*	1.0000

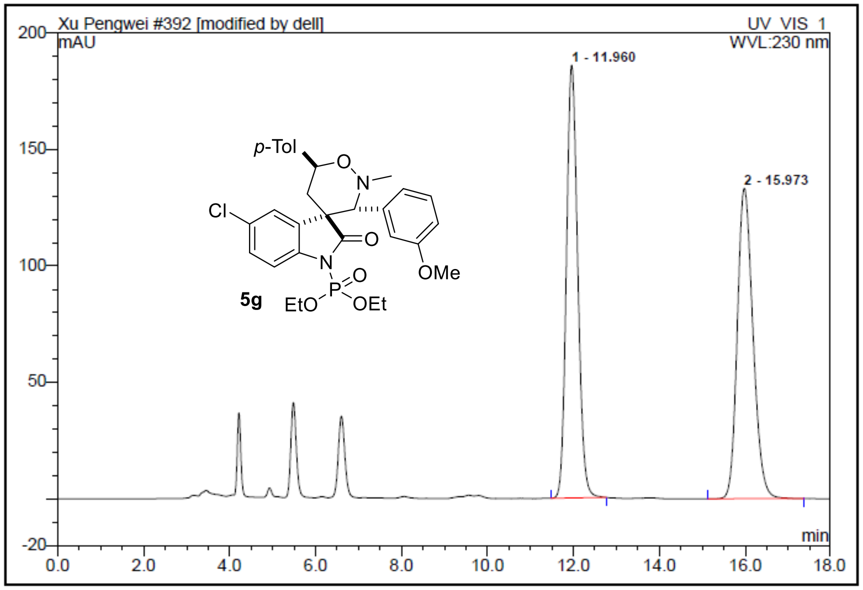

No.	Ret.Time min	Peak Name	Height mAU	Area mAU*min	Rel.Area %	Amount	Type
1	11.96	n.a.	185.890	56.666	50.15	n.a.	BMB*
2	15.97	n.a.	133.255	56.334	49.85	n.a.	BMB*
**Total:**			319.145	113.000	100.00	0.000	

Supplementary Figure 171. HPLC analysis for compound **5g**

221 xpw-xd-53-1p-asy-ic-90/10-1.0-230			
Sample Name:	**xpw-xd-53-1p-asy-ic-90/10-1.0-230**	*Injection Volume:*	**20.0**
Vial Number:	**325**	*Channel:*	**UV_VIS_1**
Sample Type:	**standard**	*Wavelength:*	**230**
Control Program:	**Xu Pengwei**	*Bandwidth:*	**n.a.**
Quantif. Method:	**Xu Pengwei**	*Dilution Factor:*	**1.0000**
Recording Time:	**2016-1-20 22:47**	*Sample Weight:*	**1.0000**
Run Time (min):	**18.17**	*Sample Amount:*	**1.0000**

222 xpw-xd-53-1p-rac-ic-90/10-1.0-230			
Sample Name:	**xpw-xd-53-1p-rac-ic-90/10-1.0-230**	*Injection Volume:*	**20.0**
Vial Number:	**326**	*Channel:*	**UV_VIS_1**
Sample Type:	**standard**	*Wavelength:*	**230**
Control Program:	**Xu Pengwei**	*Bandwidth:*	**n.a.**
Quantif. Method:	**Xu Pengwei**	*Dilution Factor:*	**1.0000**
Recording Time:	**2016-1-20 23:06**	*Sample Weight:*	**1.0000**
Run Time (min):	**18.92**	*Sample Amount:*	**1.0000**

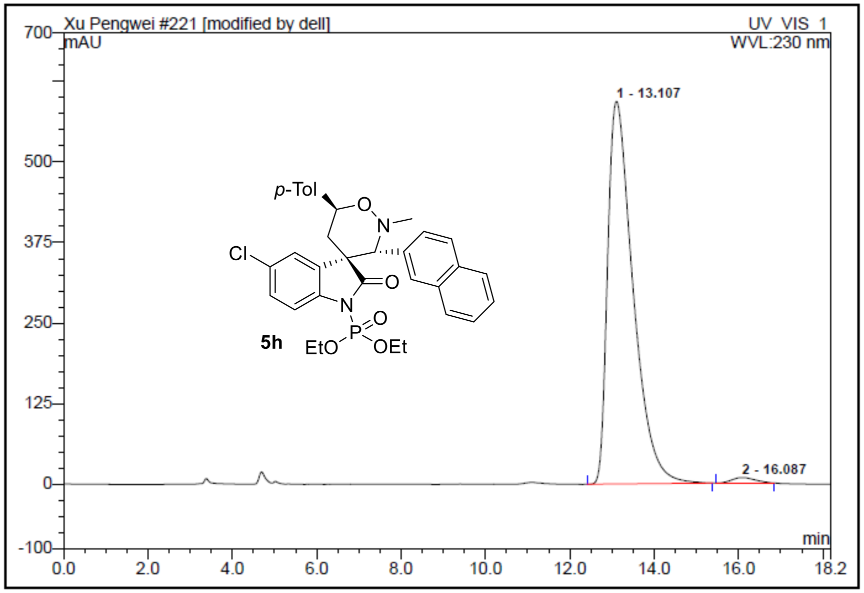

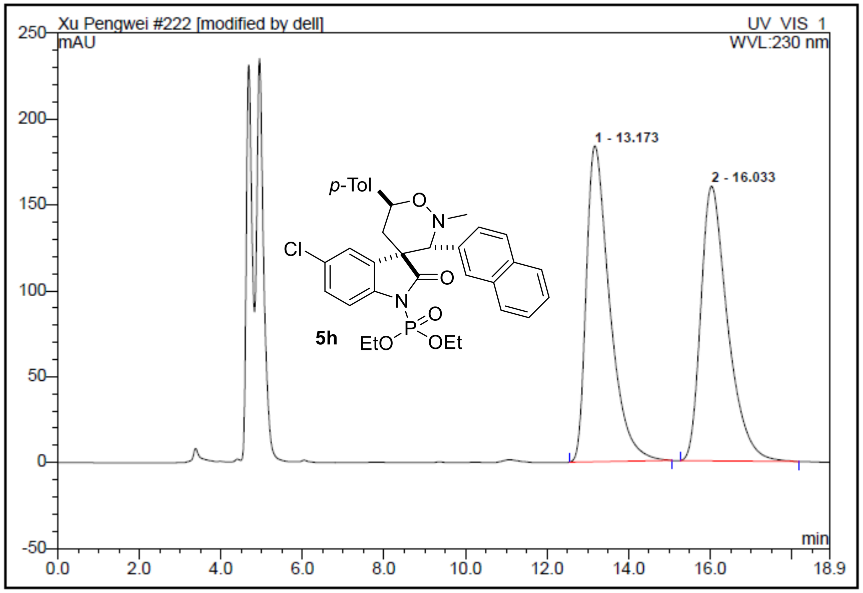

No.	Ret.Time min	Peak Name	Height mAU	Area mAU*min	Rel.Area %	Amount	Type
1	13.11	n.a.	594.152	414.209	98.69	n.a.	BMB*
2	16.09	n.a.	8.603	5.511	1.31	n.a.	BMB*
Total:			602.755	419.720	100.00	0.000	

No.	Ret.Time min	Peak Name	Height mAU	Area mAU*min	Rel.Area %	Amount	Type
1	13.17	n.a.	183.922	122.261	50.11	n.a.	BMB*
2	16.03	n.a.	159.882	121.712	49.89	n.a.	BMB*
Total:			343.804	243.973	100.00	0.000	

Supplementary Figure 172. HPLC analysis for compound **5h**

181 xpw-xd-45-1p-asy-ic-90/10-1.0-230			
Sample Name:	xpw-xd-45-1p-asy-ic-90/10-1.0-230	Injection Volume:	20.0
Vial Number:	284	Channel:	UV_VIS_1
Sample Type:	standard	Wavelength:	230
Control Program:	Xu Pengwei	Bandwidth:	n.a.
Quantif. Method:	Xu Pengwei	Dilution Factor:	1.0000
Recording Time:	2016-1-11 22:58	Sample Weight:	1.0000
Run Time (min):	16.51	Sample Amount:	1.0000

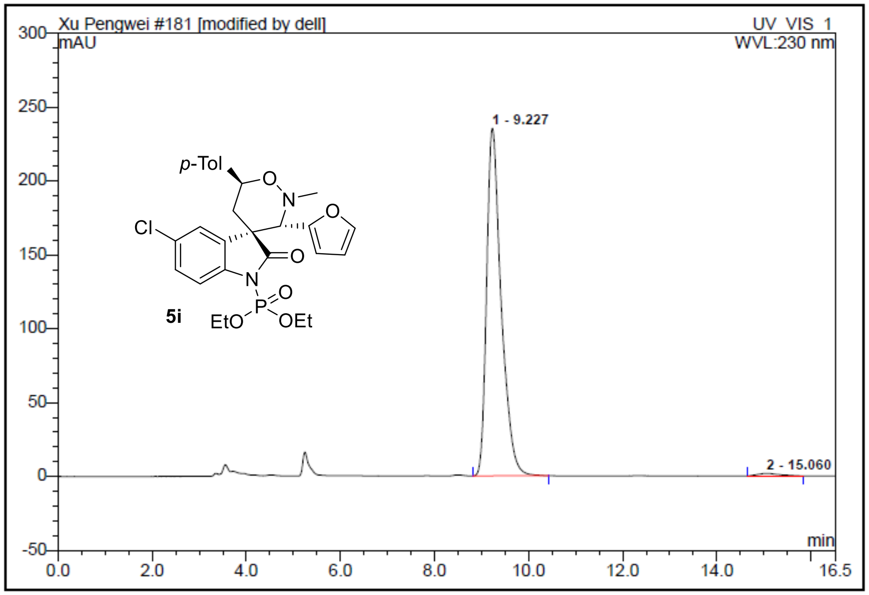

No.	Ret.Time min	Peak Name	Height mAU	Area mAU*min	Rel.Area %	Amount	Type
1	9.23	n.a.	235.277	84.228	98.95	n.a.	BMB*
2	15.06	n.a.	1.630	0.893	1.05	n.a.	BMB*
Total:			236.907	85.120	100.00	0.000	

182 xpw-xd-45-1p-rac-ic-90/10-1.0-230			
Sample Name:	xpw-xd-45-1p-rac-ic-90/10-1.0-230	Injection Volume:	20.0
Vial Number:	285	Channel:	UV_VIS_1
Sample Type:	standard	Wavelength:	230
Control Program:	Xu Pengwei	Bandwidth:	n.a.
Quantif. Method:	Xu Pengwei	Dilution Factor:	1.0000
Recording Time:	2016-1-11 23:16	Sample Weight:	1.0000
Run Time (min):	16.99	Sample Amount:	1.0000

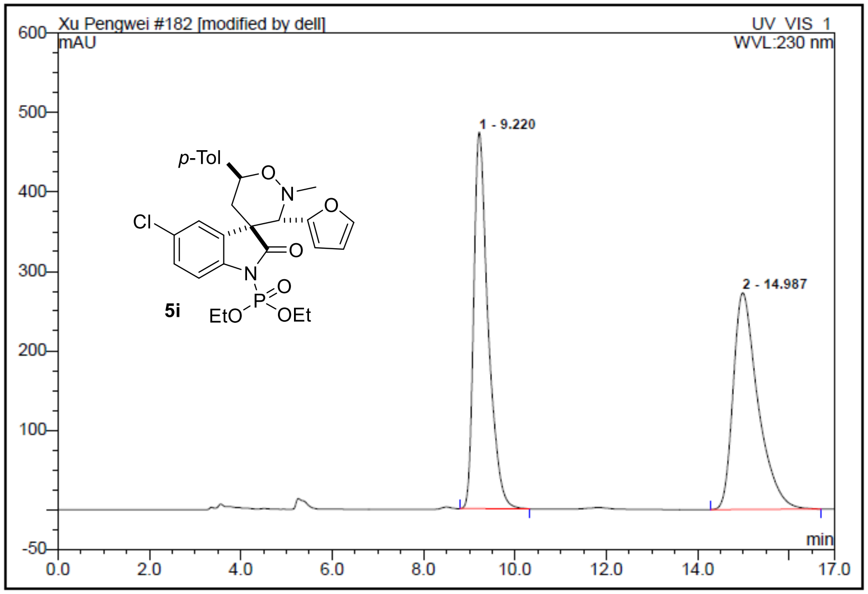

No.	Ret.Time min	Peak Name	Height mAU	Area mAU*min	Rel.Area %	Amount	Type
1	9.22	n.a.	474.373	172.674	50.17	n.a.	BMB*
2	14.99	n.a.	272.638	171.535	49.83	n.a.	BMB*
Total:			747.011	344.209	100.00	0.000	

Supplementary Figure 173. HPLC analysis for compound **5i**

202 xpw-xd-52-1p-asy-ic-90/10-1.0-230

Sample Name:	**xpw-xd-52-1p-asy-ic-90/10-1.0-230**	*Injection Volume:*	**20.0**
Vial Number:	**306**	*Channel:*	**UV_VIS_1**
Sample Type:	**standard**	*Wavelength:*	**230**
Control Program:	**Xu Pengwei**	*Bandwidth:*	**n.a.**
Quantif. Method:	**Xu Pengwei**	*Dilution Factor:*	**1.0000**
Recording Time:	**2016-1-16 19:48**	*Sample Weight:*	**1.0000**
Run Time (min):	**17.10**	*Sample Amount:*	**1.0000**

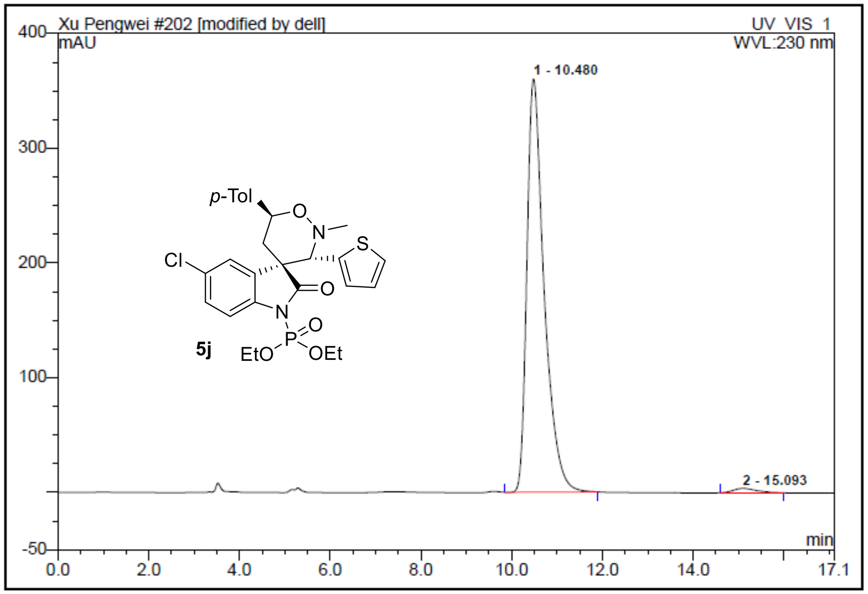

No.	Ret.Time min	Peak Name	Height mAU	Area mAU*min	Rel.Area %	Amount	Type
1	10.48	n.a.	360.289	158.344	98.59	n.a.	BMB*
2	15.09	n.a.	3.823	2.272	1.41	n.a.	BMB*
**Total:**			364.112	160.615	100.00	0.000	

201 xpw-xd-52-1p-rac-ic-90/10-1.0-230

Sample Name:	**xpw-xd-52-1p-rac-ic-90/10-1.0-230**	*Injection Volume:*	**20.0**
Vial Number:	**305**	*Channel:*	**UV_VIS_1**
Sample Type:	**standard**	*Wavelength:*	**230**
Control Program:	**Xu Pengwei**	*Bandwidth:*	**n.a.**
Quantif. Method:	**Xu Pengwei**	*Dilution Factor:*	**1.0000**
Recording Time:	**2016-1-16 20:06**	*Sample Weight:*	**1.0000**
Run Time (min):	**18.71**	*Sample Amount:*	**1.0000**

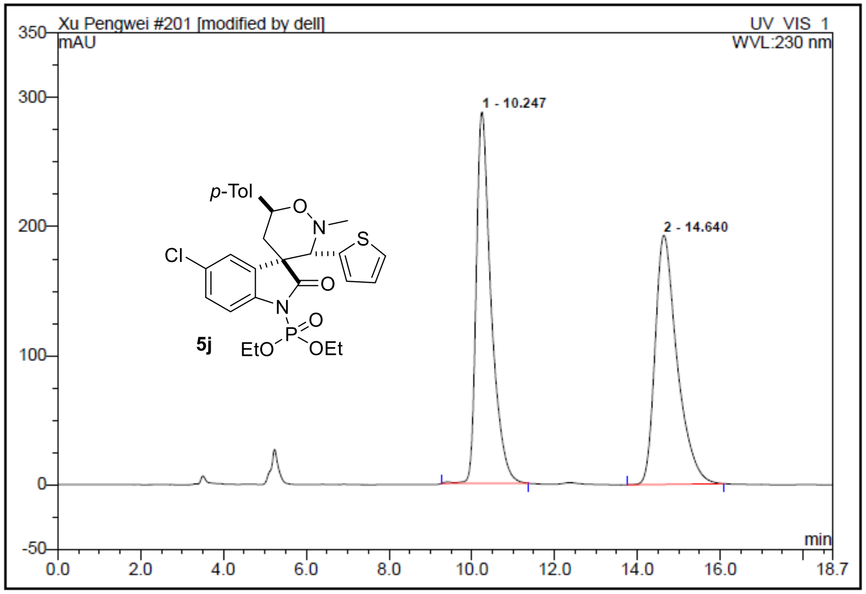

No.	Ret.Time min	Peak Name	Height mAU	Area mAU*min	Rel.Area %	Amount	Type
1	10.25	n.a.	287.793	119.805	50.17	n.a.	BMB*
2	14.64	n.a.	193.042	118.971	49.83	n.a.	BMB*
**Total:**			480.835	238.776	100.00	0.000	

Supplementary Figure 174. HPLC analysis for compound **5j**

331 xpw-xd-102-1p-asy-ic-90/10-1.0-230			
Sample Name:	**xpw-xd-102-1p-asy-ic-90/10-1.0-230**	*Injection Volume:*	**20.0**
Vial Number:	**435**	*Channel:*	**UV_VIS_1**
Sample Type:	**standard**	*Wavelength:*	**230**
Control Program:	**Xu Pengwei**	*Bandwidth:*	**n.a.**
Quantif. Method:	**Xu Pengwei**	*Dilution Factor:*	**1.0000**
Recording Time:	**2016-3-4 20:28**	*Sample Weight:*	**1.0000**
Run Time (min):	**11.60**	*Sample Amount:*	**1.0000**

332 xpw-xd-102-1p-rac-ic-90/10-1.0-230			
Sample Name:	**xpw-xd-102-1p-rac-ic-90/10-1.0-230**	*Injection Volume:*	**20.0**
Vial Number:	**436**	*Channel:*	**UV_VIS_1**
Sample Type:	**standard**	*Wavelength:*	**230**
Control Program:	**Xu Pengwei**	*Bandwidth:*	**n.a.**
Quantif. Method:	**Xu Pengwei**	*Dilution Factor:*	**1.0000**
Recording Time:	**2016-3-4 20:43**	*Sample Weight:*	**1.0000**
Run Time (min):	**9.47**	*Sample Amount:*	**1.0000**

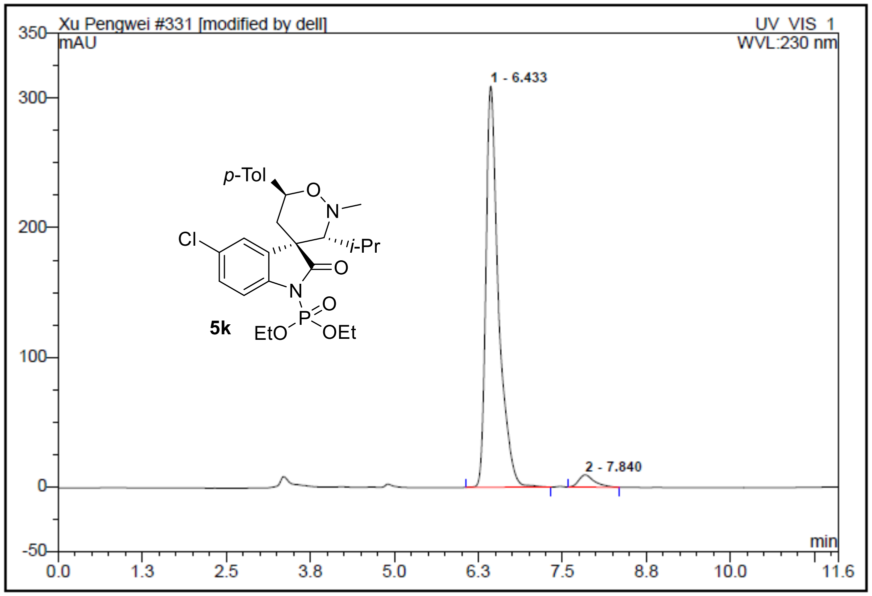

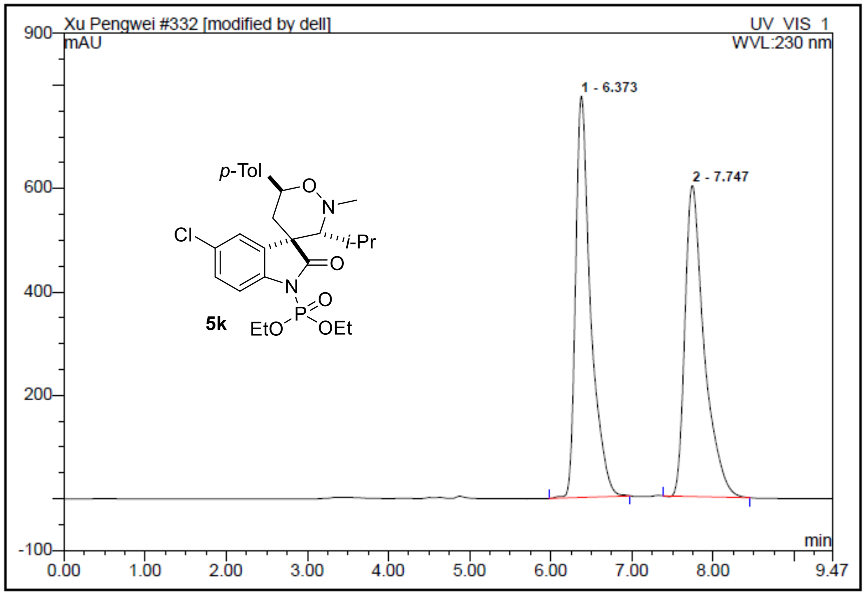

No.	Ret.Time min	Peak Name	Height mAU	Area mAU*min	Rel.Area %	Amount	Type
1	6.43	n.a.	309.951	67.624	96.38	n.a.	BMB*
2	7.84	n.a.	9.426	2.537	3.62	n.a.	BMB*
**Total:**			319.376	70.162	100.00	0.000	

No.	Ret.Time min	Peak Name	Height mAU	Area mAU*min	Rel.Area %	Amount	Type
1	6.37	n.a.	776.008	167.014	50.19	n.a.	BMB*
2	7.75	n.a.	601.322	165.751	49.81	n.a.	BMB*
**Total:**			1377.330	332.765	100.00	0.000	

Supplementary Figure 175. HPLC analysis for compound **5k**

344 xpw-xd-106-1p-asy-ic-85/15-1.0-230

Sample Name:	xpw-xd-106-1p-asy-ic-85/15-1.0-230	*Injection Volume:*	20.0
Vial Number:	450	*Channel:*	UV_VIS_1
Sample Type:	standard	*Wavelength:*	230
Control Program:	Xu Pengwei	*Bandwidth:*	n.a.
Quantif. Method:	Xu Pengwei	*Dilution Factor:*	1.0000
Recording Time:	2016-3-7 16:01	*Sample Weight:*	1.0000
Run Time (min):	6.98	*Sample Amount:*	1.0000

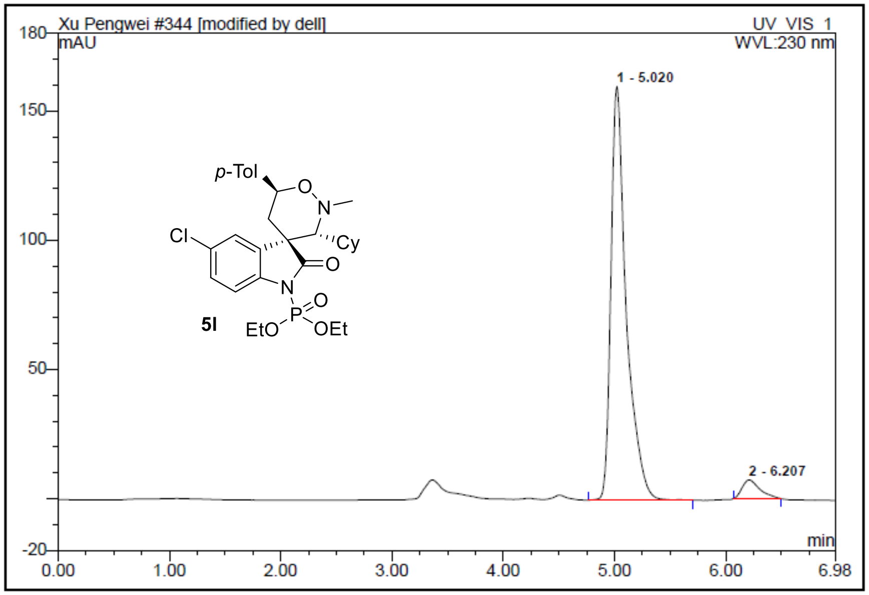

No.	Ret.Time min	Peak Name	Height mAU	Area mAU*min	Rel.Area %	Amount	Type
1	5.02	n.a.	160.060	25.586	95.02	n.a.	BMB*
2	6.21	n.a.	7.262	1.340	4.98	n.a.	BMB*
Total:			167.323	26.927	100.00	0.000	

345 xpw-xd-106-1p-rac-ic-85/15-1.0-230

Sample Name:	xpw-xd-106-1p-rac-ic-85/15-1.0-230	*Injection Volume:*	20.0
Vial Number:	453	*Channel:*	UV_VIS_1
Sample Type:	standard	*Wavelength:*	230
Control Program:	Xu Pengwei	*Bandwidth:*	n.a.
Quantif. Method:	Xu Pengwei	*Dilution Factor:*	1.0000
Recording Time:	2016-3-7 16:29	*Sample Weight:*	1.0000
Run Time (min):	6.94	*Sample Amount:*	1.0000

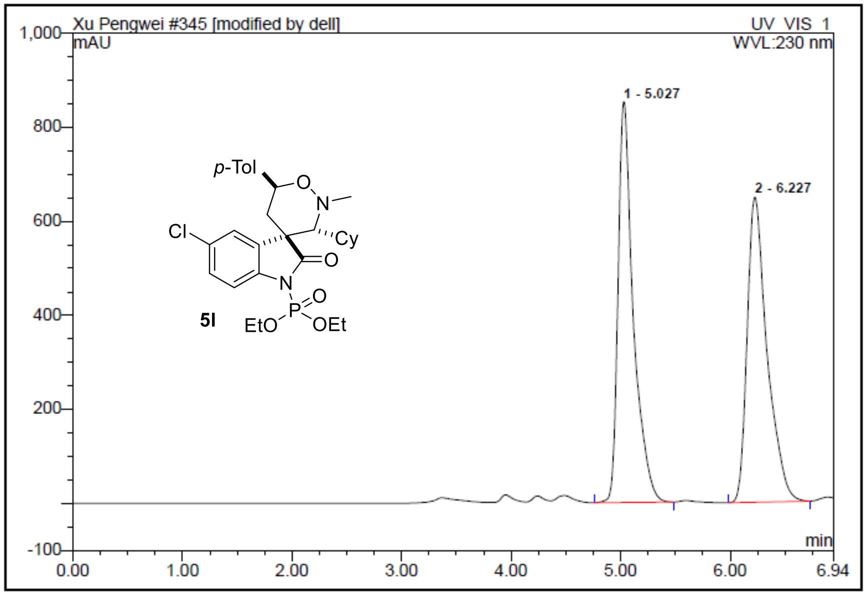

No.	Ret.Time min	Peak Name	Height mAU	Area mAU*min	Rel.Area %	Amount	Type
1	5.03	n.a.	852.473	137.782	50.15	n.a.	BMB*
2	6.23	n.a.	649.350	136.960	49.85	n.a.	BMB
Total:			1501.823	274.742	100.00	0.000	

Supplementary Figure 176. HPLC analysis for compound **5l**

353 xpw-xd-88-1p-asy-ic-90/10-1.0-230

Sample Name:	xpw-xd-88-1p-asy-ic-90/10-1.0-230	*Injection Volume:*	20.0
Vial Number:	464	*Channel:*	UV_VIS_1
Sample Type:	standard	*Wavelength:*	230
Control Program:	Xu Pengwei	*Bandwidth:*	n.a.
Quantif. Method:	Xu Pengwei	*Dilution Factor:*	1.0000
Recording Time:	2016-3-17 17:03	*Sample Weight:*	1.0000
Run Time (min):	20.64	*Sample Amount:*	1.0000

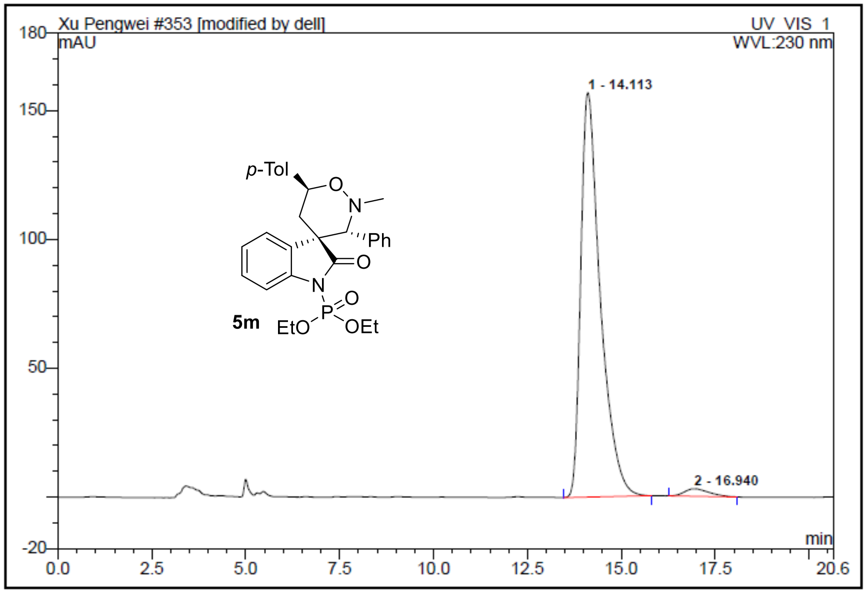

No.	Ret.Time min	Peak Name	Height mAU	Area mAU*min	Rel.Area %	Amount	Type
1	14.11	n.a.	156.968	94.905	97.70	n.a.	BMB*
2	16.94	n.a.	2.945	2.237	2.30	n.a.	BMB
Total:			159.912	97.142	100.00	0.000	

354 xpw-xd-88-1p-rac-ic-90/10-1.0-230

Sample Name:	xpw-xd-88-1p-rac-ic-90/10-1.0-230	*Injection Volume:*	20.0
Vial Number:	465	*Channel:*	UV_VIS_1
Sample Type:	standard	*Wavelength:*	230
Control Program:	Xu Pengwei	*Bandwidth:*	n.a.
Quantif. Method:	Xu Pengwei	*Dilution Factor:*	1.0000
Recording Time:	2016-3-17 17:25	*Sample Weight:*	1.0000
Run Time (min):	20.92	*Sample Amount:*	1.0000

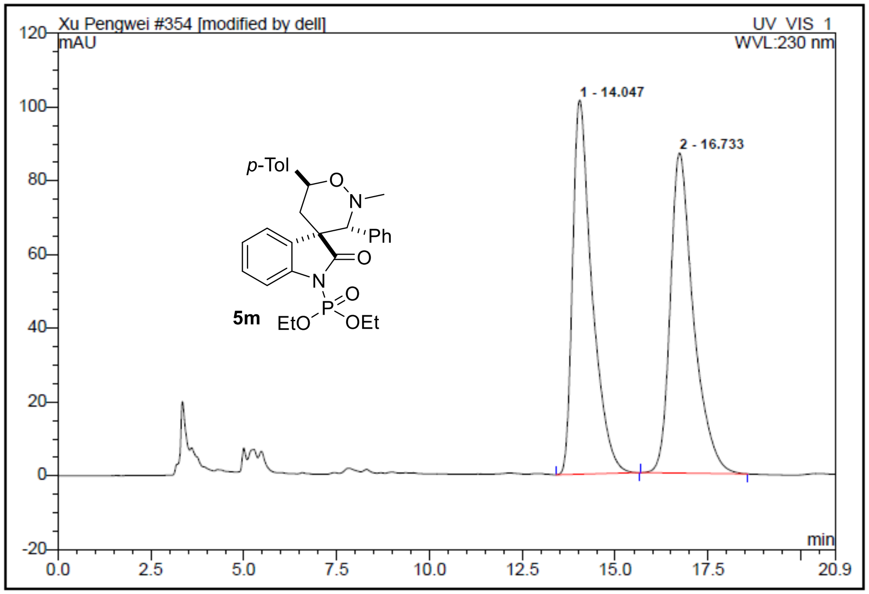

No.	Ret.Time min	Peak Name	Height mAU	Area mAU*min	Rel.Area %	Amount	Type
1	14.05	n.a.	101.510	61.531	49.35	n.a.	BMB
2	16.73	n.a.	86.882	63.145	50.65	n.a.	BMB
Total:			188.392	124.676	100.00	0.000	

Supplementary Figure 177. HPLC analysis for compound **5m**

355 xpw-xd-88-1s-asy-ic-80/20-1.0-230

Sample Name:	xpw-xd-88-1s-asy-ic-80/20-1.0-230	*Injection Volume:*	20.0
Vial Number:	466	*Channel:*	UV_VIS_1
Sample Type:	standard	*Wavelength:*	230
Control Program:	Xu Pengwei	*Bandwidth:*	n.a.
Quantif. Method:	Xu Pengwei	*Dilution Factor:*	1.0000
Recording Time:	2016-3-17 20:22	*Sample Weight:*	1.0000
Run Time (min):	17.85	*Sample Amount:*	1.0000

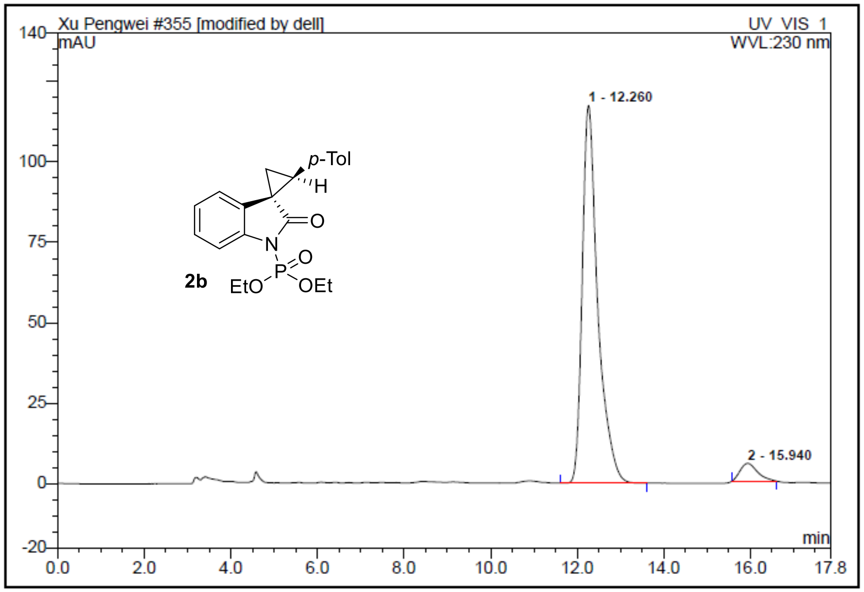

No.	Ret.Time min	Peak Name	Height mAU	Area mAU*min	Rel.Area %	Amount	Type
1	12.26	n.a.	117.428	48.886	94.99	n.a.	BMB*
2	15.94	n.a.	5.543	2.577	5.01	n.a.	BMB*
Total:			122.971	51.464	100.00	0.000	

356 xpw-xd-88-1s-rac-ic-80/20-1.0-230

Sample Name:	xpw-xd-88-1s-rac-ic-80/20-1.0-230	*Injection Volume:*	20.0
Vial Number:	467	*Channel:*	UV_VIS_1
Sample Type:	standard	*Wavelength:*	230
Control Program:	Xu Pengwei	*Bandwidth:*	n.a.
Quantif. Method:	Xu Pengwei	*Dilution Factor:*	1.0000
Recording Time:	2016-3-17 20:43	*Sample Weight:*	1.0000
Run Time (min):	17.95	*Sample Amount:*	1.0000

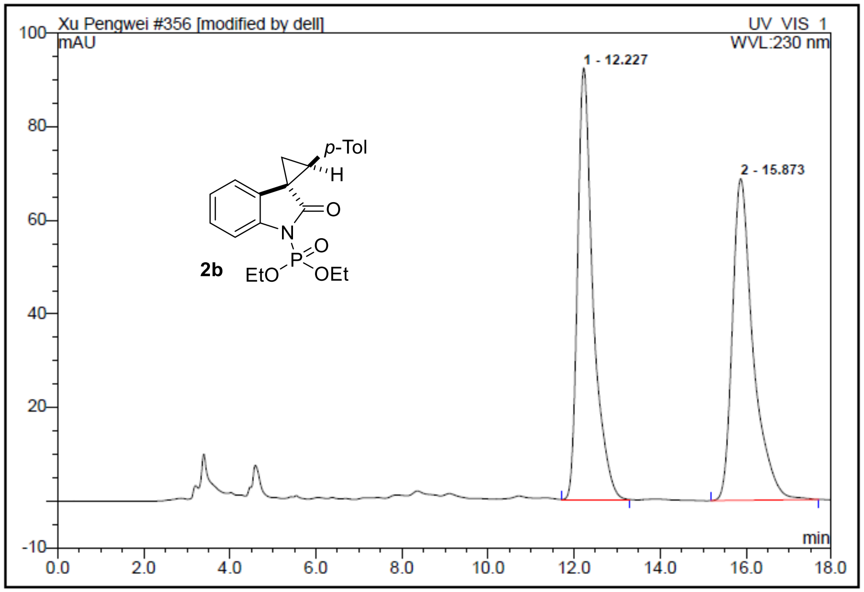

No.	Ret.Time min	Peak Name	Height mAU	Area mAU*min	Rel.Area %	Amount	Type
1	12.23	n.a.	92.279	38.408	49.92	n.a.	BMB
2	15.87	n.a.	68.760	38.536	50.08	n.a.	BMB
Total:			161.038	76.944	100.00	0.000	

Supplementary Figure 178. HPLC analysis for compound **2b**

278 xpw-xd-69-1p-asy-ic-85/15-1.0-230

Sample Name:	xpw-xd-69-1p-asy-ic-85/15-1.0-230	*Injection Volume:*	20.0
Vial Number:	382	*Channel:*	UV_VIS_1
Sample Type:	standard	*Wavelength:*	230
Control Program:	Xu Pengwei	*Bandwidth:*	n.a.
Quantif. Method:	Xu Pengwei	*Dilution Factor:*	1.0000
Recording Time:	2016-1-29 14:51	*Sample Weight:*	1.0000
Run Time (min):	16.89	*Sample Amount:*	1.0000

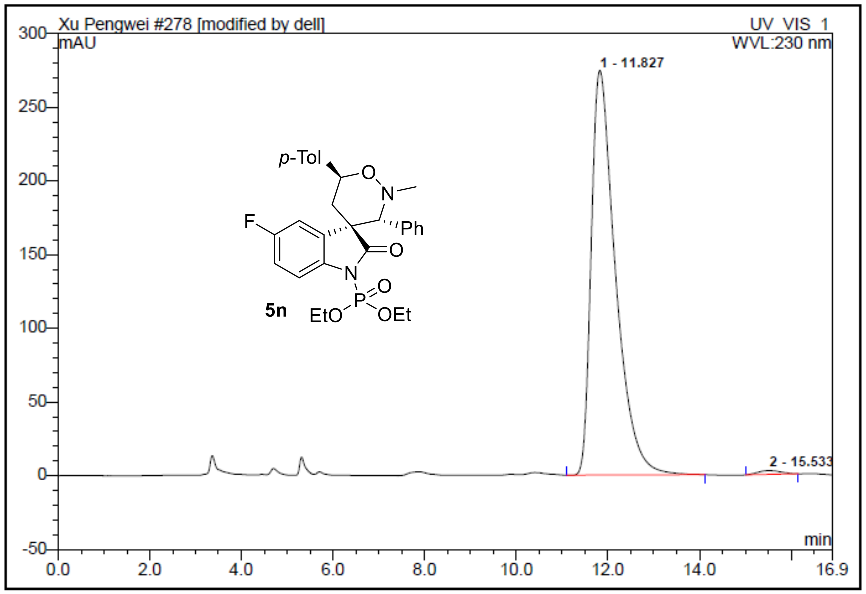

No.	Ret.Time min	Peak Name	Height mAU	Area mAU*min	Rel.Area %	Amount	Type
1	11.83	n.a.	274.543	168.548	99.10	n.a.	BMB*
2	15.53	n.a.	2.591	1.525	0.90	n.a.	BMB*
**Total:**			277.134	170.073	100.00	0.000	

279 xpw-xd-69-1p-rac-ic-85/15-1.0-230

Sample Name:	xpw-xd-69-1p-rac-ic-85/15-1.0-230	*Injection Volume:*	20.0
Vial Number:	383	*Channel:*	UV_VIS_1
Sample Type:	standard	*Wavelength:*	230
Control Program:	Xu Pengwei	*Bandwidth:*	n.a.
Quantif. Method:	Xu Pengwei	*Dilution Factor:*	1.0000
Recording Time:	2016-1-29 15:10	*Sample Weight:*	1.0000
Run Time (min):	18.64	*Sample Amount:*	1.0000

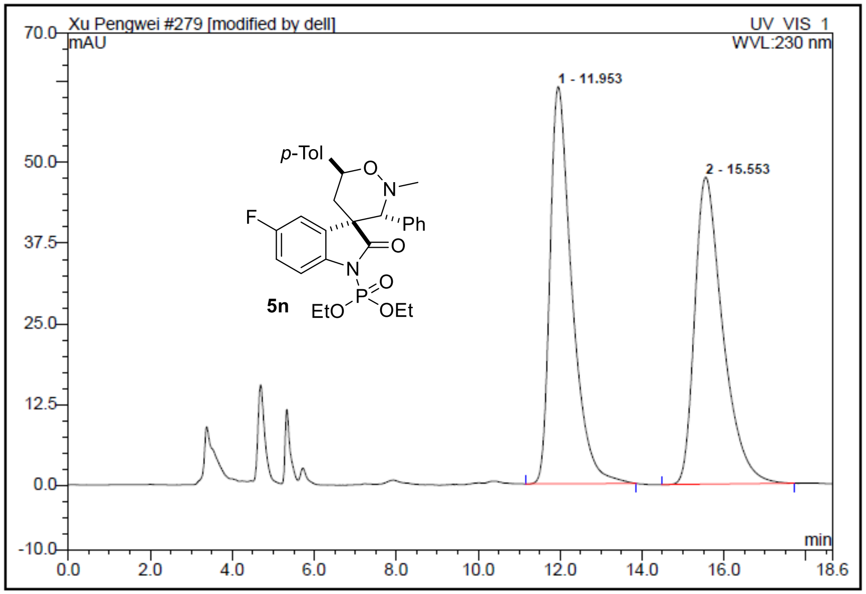

No.	Ret.Time min	Peak Name	Height mAU	Area mAU*min	Rel.Area %	Amount	Type
1	11.95	n.a.	61.541	38.106	50.14	n.a.	BMB*
2	15.55	n.a.	47.578	37.893	49.86	n.a.	BMB*
**Total:**			109.119	75.999	100.00	0.000	

Supplementary Figure 179. HPLC analysis for compound **5n**

276 xpw-xd-69-1s-asy-ic-85/15-1.0-230

Sample Name:	xpw-xd-69-1s-asy-ic-85/15-1.0-230	*Injection Volume:*	20.0
Vial Number:	380	*Channel:*	UV_VIS_1
Sample Type:	standard	*Wavelength:*	230
Control Program:	Xu Pengwei	*Bandwidth:*	n.a.
Quantif. Method:	Xu Pengwei	*Dilution Factor:*	1.0000
Recording Time:	2016-1-29 13:02	*Sample Weight:*	1.0000
Run Time (min):	26.64	*Sample Amount:*	1.0000

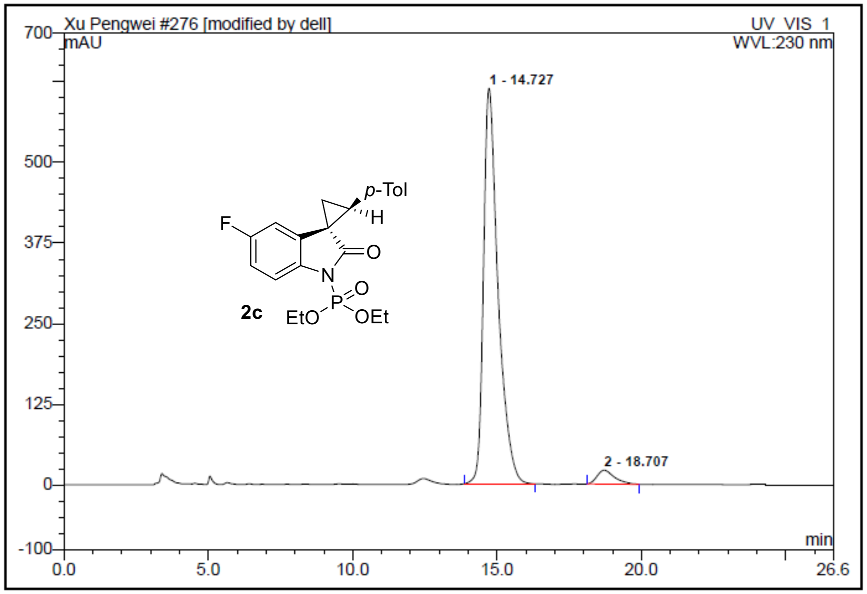

No.	Ret.Time min	Peak Name	Height mAU	Area mAU*min	Rel.Area %	Amount	Type
1	14.73	n.a.	612.858	358.428	96.03	n.a.	BMB*
2	18.71	n.a.	21.207	14.812	3.97	n.a.	BMB*
Total:			634.065	373.240	100.00	0.000	

277 xpw-xd-69-1s-rac-ic-85/15-1.0-230

Sample Name:	xpw-xd-69-1s-rac-ic-85/15-1.0-230	*Injection Volume:*	20.0
Vial Number:	381	*Channel:*	UV_VIS_1
Sample Type:	standard	*Wavelength:*	230
Control Program:	Xu Pengwei	*Bandwidth:*	n.a.
Quantif. Method:	Xu Pengwei	*Dilution Factor:*	1.0000
Recording Time:	2016-1-29 14:01	*Sample Weight:*	1.0000
Run Time (min):	21.20	*Sample Amount:*	1.0000

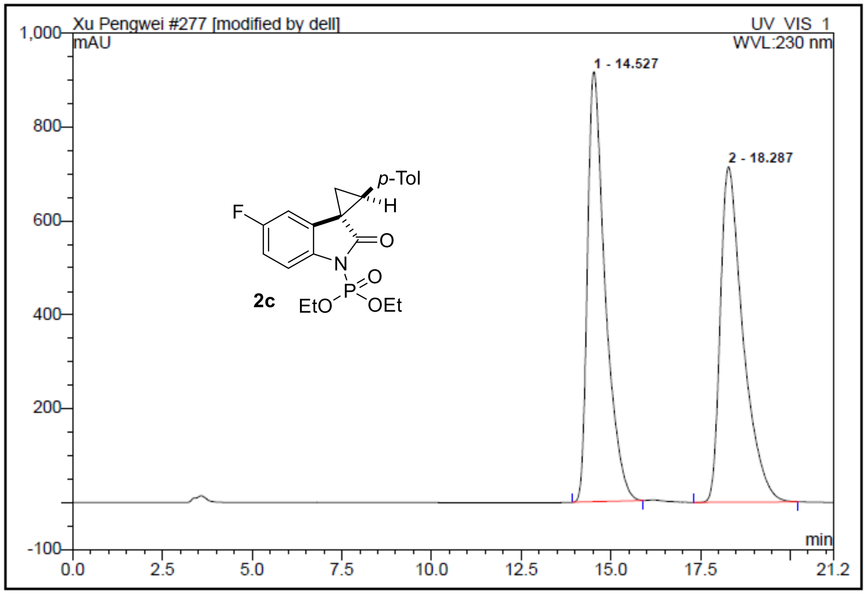

No.	Ret.Time min	Peak Name	Height mAU	Area mAU*min	Rel.Area %	Amount	Type
1	14.53	n.a.	916.389	525.232	49.95	n.a.	BMB*
2	18.29	n.a.	714.674	526.249	50.05	n.a.	BMB*
Total:			1631.063	1051.481	100.00	0.000	

Supplementary Figure 180. HPLC analysis for compound **2c**

280 xpw-xd-83-1p-asy-ic-90/10-1.0-230

Sample Name:	xpw-xd-83-1p-asy-ic-90/10-1.0-230	Injection Volume:	20.0
Vial Number:	384	Channel:	UV_VIS_1
Sample Type:	standard	Wavelength:	230
Control Program:	Xu Pengwei	Bandwidth:	n.a.
Quantif. Method:	Xu Pengwei	Dilution Factor:	1.0000
Recording Time:	2016-2-19 17:11	Sample Weight:	1.0000
Run Time (min):	16.46	Sample Amount:	1.0000

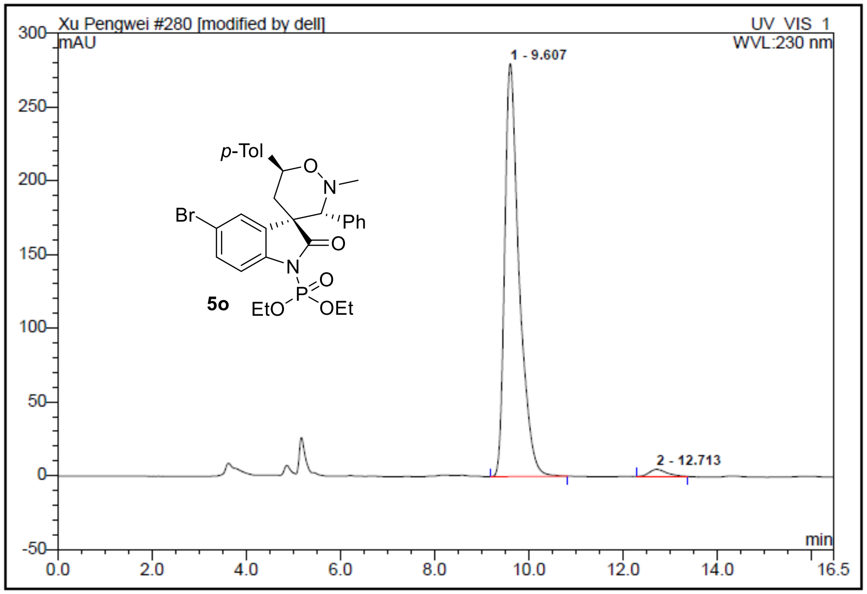

No.	Ret.Time min	Peak Name	Height mAU	Area mAU*min	Rel.Area %	Amount	Type
1	9.61	n.a.	279.991	99.573	97.94	n.a.	BMB*
2	12.71	n.a.	4.807	2.098	2.06	n.a.	BMB*
Total:			284.797	101.671	100.00	0.000	

281 xpw-xd-83-1p-rac-ic-90/10-1.0-230

Sample Name:	xpw-xd-83-1p-rac-ic-90/10-1.0-230	Injection Volume:	20.0
Vial Number:	385	Channel:	UV_VIS_1
Sample Type:	standard	Wavelength:	230
Control Program:	Xu Pengwei	Bandwidth:	n.a.
Quantif. Method:	Xu Pengwei	Dilution Factor:	1.0000
Recording Time:	2016-2-19 17:29	Sample Weight:	1.0000
Run Time (min):	14.13	Sample Amount:	1.0000

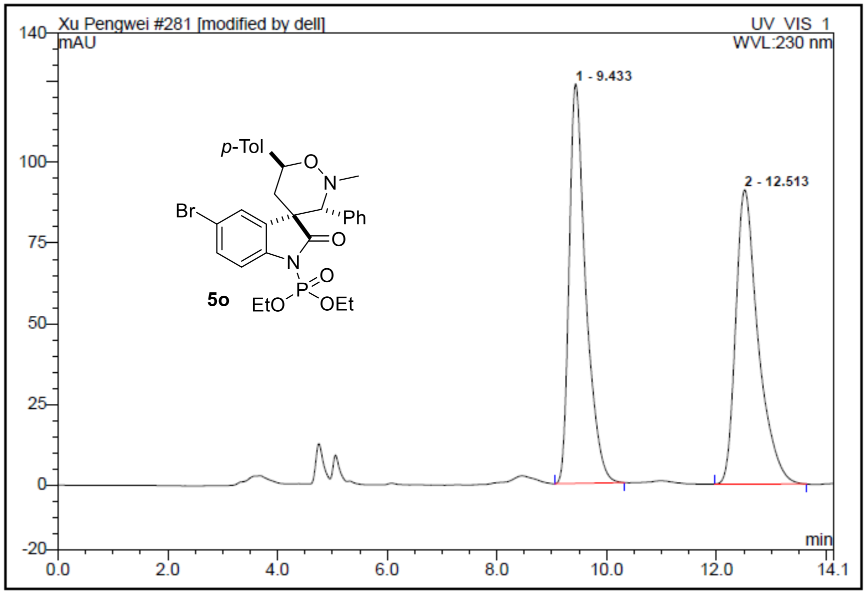

No.	Ret.Time min	Peak Name	Height mAU	Area mAU*min	Rel.Area %	Amount	Type
1	9.43	n.a.	123.858	43.356	50.01	n.a.	BMB*
2	12.51	n.a.	91.218	43.345	49.99	n.a.	BMB*
Total:			215.076	86.701	100.00	0.000	

Supplementary Figure 181. HPLC analysis for compound **5o**

282 xpw-xd-83-1s-asy-ic-90/10-1.0-230

Sample Name:	xpw-xd-83-1s-asy-ic-90/10-1.0-230	*Injection Volume:*	20.0
Vial Number:	386	*Channel:*	UV_VIS_1
Sample Type:	standard	*Wavelength:*	230
Control Program:	Xu Pengwei	*Bandwidth:*	n.a.
Quantif. Method:	Xu Pengwei	*Dilution Factor:*	1.0000
Recording Time:	2016-2-19 18:54	*Sample Weight:*	1.0000
Run Time (min):	23.09	*Sample Amount:*	1.0000

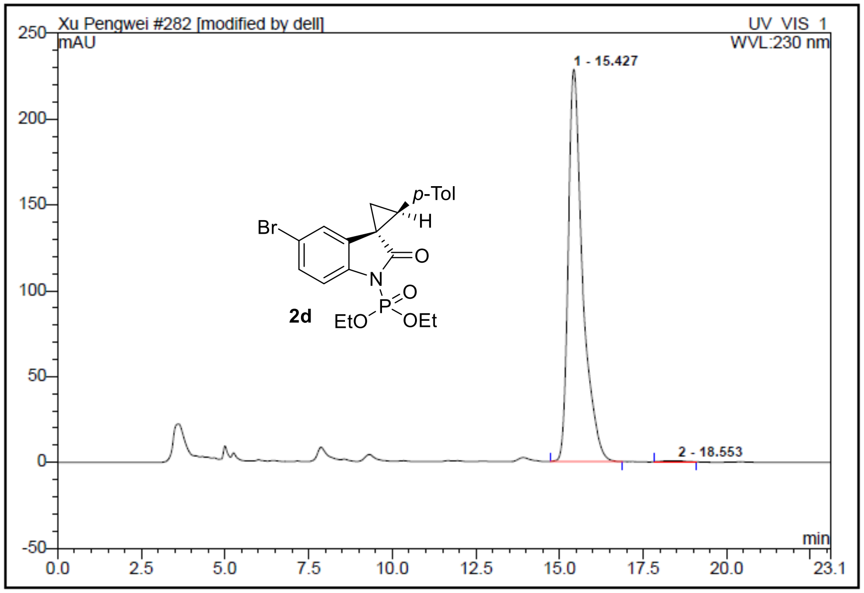

No.	Ret.Time min	Peak Name	Height mAU	Area mAU*min	Rel.Area %	Amount	Type
1	15.43	n.a.	228.400	113.712	99.61	n.a.	BMB*
2	18.55	n.a.	0.548	0.443	0.39	n.a.	BMB*
Total:			228.948	114.155	100.00	0.000	

283 xpw-xd-83-1s-rac-ic-90/10-1.0-230

Sample Name:	xpw-xd-83-1s-rac-ic-90/10-1.0-230	*Injection Volume:*	20.0
Vial Number:	387	*Channel:*	UV_VIS_1
Sample Type:	standard	*Wavelength:*	230
Control Program:	Xu Pengwei	*Bandwidth:*	n.a.
Quantif. Method:	Xu Pengwei	*Dilution Factor:*	1.0000
Recording Time:	2016-2-19 19:18	*Sample Weight:*	1.0000
Run Time (min):	20.27	*Sample Amount:*	1.0000

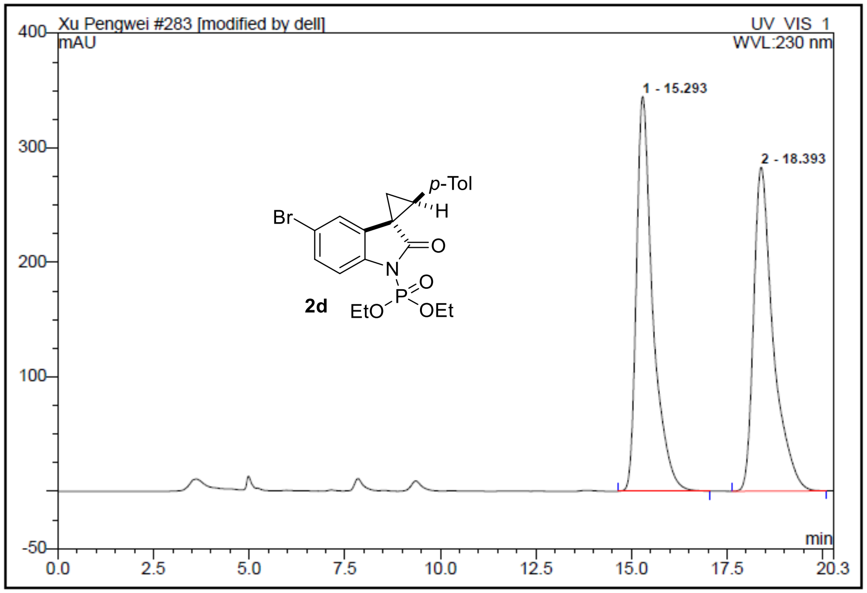

No.	Ret.Time min	Peak Name	Height mAU	Area mAU*min	Rel.Area %	Amount	Type
1	15.29	n.a.	344.649	171.111	50.47	n.a.	BMB*
2	18.39	n.a.	282.870	167.952	49.53	n.a.	BMB*
Total:			627.518	339.062	100.00	0.000	

Supplementary Figure 182. HPLC analysis for compound **2d**

310 xpw-xd-85-1p-asy-ic-90/10-0.5-230			
Sample Name:	**xpw-xd-85-1p-asy-ic-90/10-0.5-230**	*Injection Volume:*	**20.0**
Vial Number:	**414**	*Channel:*	**UV_VIS_1**
Sample Type:	**standard**	*Wavelength:*	**230**
Control Program:	**Xu Pengwei**	*Bandwidth:*	**n.a.**
Quantif. Method:	**Xu Pengwei**	*Dilution Factor:*	**1.0000**
Recording Time:	**2016-2-24 17:20**	*Sample Weight:*	**1.0000**
Run Time (min):	**24.09**	*Sample Amount:*	**1.0000**

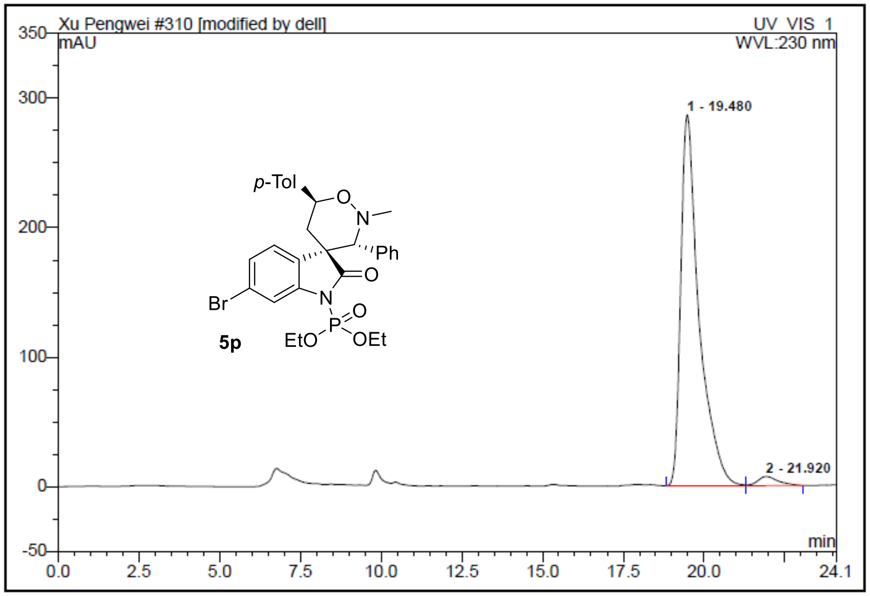

No.	Ret.Time min	Peak Name	Height mAU	Area mAU*min	Rel.Area %	Amount	Type
1	19.48	n.a.	286.510	187.694	97.35	n.a.	BM *
2	21.92	n.a.	7.016	5.106	2.65	n.a.	MB*
Total:			293.526	192.799	100.00	0.000	

311 xpw-xd-85-1p-rac-ic-90/10-0.5-230			
Sample Name:	**xpw-xd-85-1p-rac-ic-90/10-0.5-230**	*Injection Volume:*	**20.0**
Vial Number:	**415**	*Channel:*	**UV_VIS_1**
Sample Type:	**standard**	*Wavelength:*	**230**
Control Program:	**Xu Pengwei**	*Bandwidth:*	**n.a.**
Quantif. Method:	**Xu Pengwei**	*Dilution Factor:*	**1.0000**
Recording Time:	**2016-2-24 17:44**	*Sample Weight:*	**1.0000**
Run Time (min):	**33.45**	*Sample Amount:*	**1.0000**

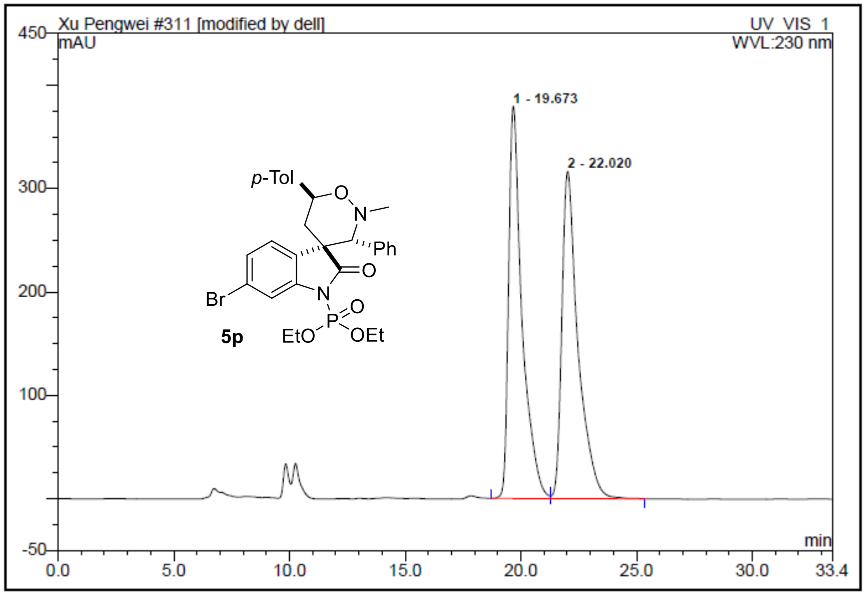

No.	Ret.Time min	Peak Name	Height mAU	Area mAU*min	Rel.Area %	Amount	Type
1	19.67	n.a.	379.344	256.135	49.92	n.a.	BM *
2	22.02	n.a.	316.420	256.956	50.08	n.a.	MB*
Total:			695.765	513.091	100.00	0.000	

Supplementary Figure 183. HPLC analysis for compound **5p**

305 xpw-xd-85-1s-asy-ic-85/15-1.0-230

Sample Name:	xpw-xd-85-1s-asy-ic-85/15-1.0-230	*Injection Volume:*	20.0
Vial Number:	409	*Channel:*	UV_VIS_1
Sample Type:	standard	*Wavelength:*	230
Control Program:	Xu Pengwei	*Bandwidth:*	n.a.
Quantif. Method:	Xu Pengwei	*Dilution Factor:*	1.0000
Recording Time:	2016-2-24 14:03	*Sample Weight:*	1.0000
Run Time (min):	29.39	*Sample Amount:*	1.0000

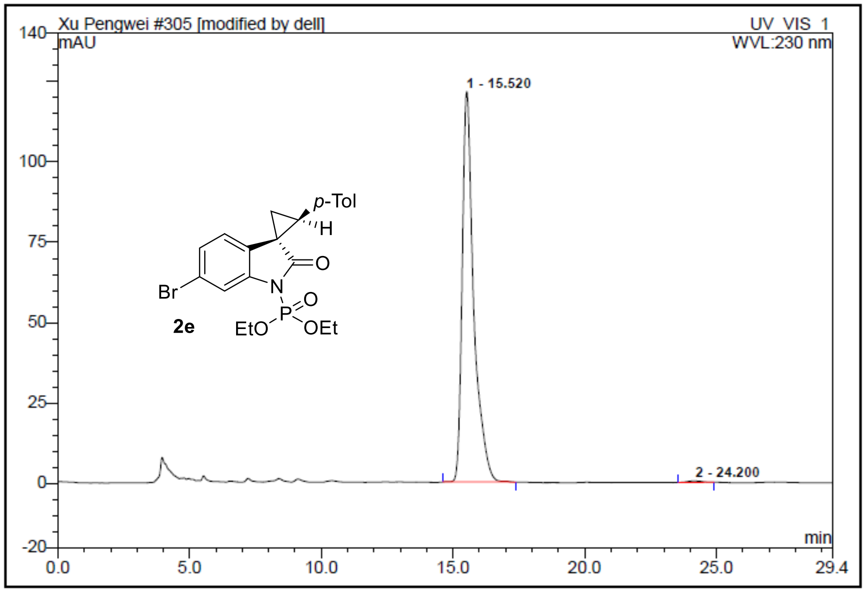

No.	Ret.Time min	Peak Name	Height mAU	Area mAU*min	Rel.Area %	Amount	Type
1	15.52	n.a.	121.498	61.662	99.51	n.a.	BMB*
2	24.20	n.a.	0.494	0.303	0.49	n.a.	BMB*
**Total:**			121.992	61.965	100.00	0.000	

306 xpw-xd-85-1s-rac-ic-85/15-1.0-230

Sample Name:	xpw-xd-85-1s-rac-ic-85/15-1.0-230	*Injection Volume:*	20.0
Vial Number:	410	*Channel:*	UV_VIS_1
Sample Type:	standard	*Wavelength:*	230
Control Program:	Xu Pengwei	*Bandwidth:*	n.a.
Quantif. Method:	Xu Pengwei	*Dilution Factor:*	1.0000
Recording Time:	2016-2-24 14:34	*Sample Weight:*	1.0000
Run Time (min):	27.10	*Sample Amount:*	1.0000

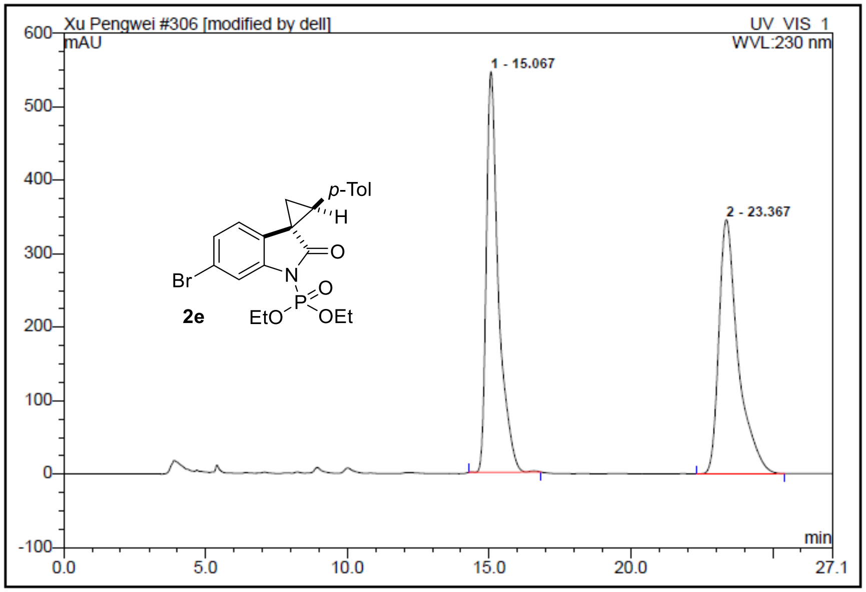

No.	Ret.Time min	Peak Name	Height mAU	Area mAU*min	Rel.Area %	Amount	Type
1	15.07	n.a.	545.443	270.340	49.82	n.a.	BMB*
2	23.37	n.a.	346.158	272.296	50.18	n.a.	BMB*
**Total:**			891.601	542.636	100.00	0.000	

Supplementary Figure 184. HPLC analysis for compound **2e**

361 xpw-xd-111-1p-asy-ic-80/20-1.0-230

Sample Name:	**xpw-xd-111-1p-asy-ic-80/20-1.0-230**	*Injection Volume:*	**20.0**
Vial Number:	**475**	*Channel:*	**UV_VIS_1**
Sample Type:	**standard**	*Wavelength:*	**230**
Control Program:	**Xu Pengwei**	*Bandwidth:*	**n.a.**
Quantif. Method:	**Xu Pengwei**	*Dilution Factor:*	**1.0000**
Recording Time:	**2016-3-18 9:56**	*Sample Weight:*	**1.0000**
Run Time (min):	**10.98**	*Sample Amount:*	**1.0000**

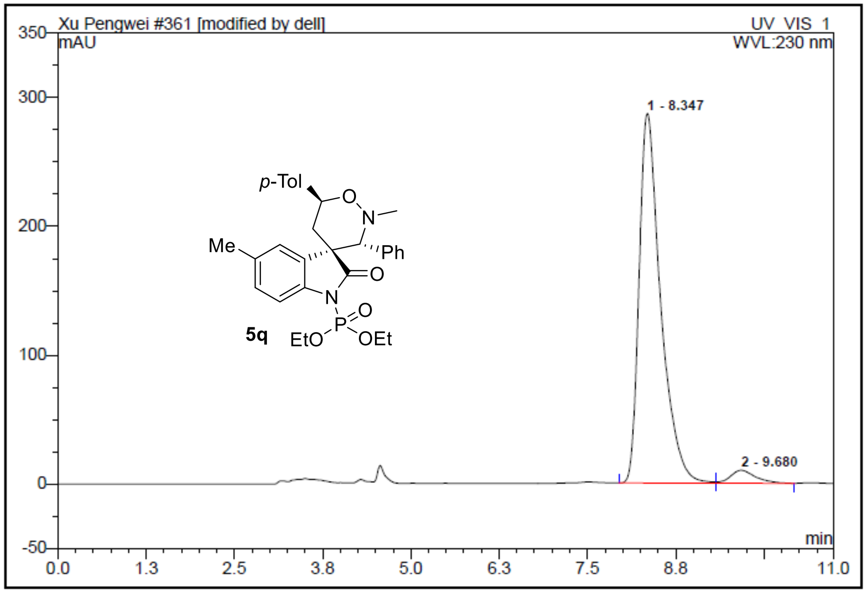

No.	Ret.Time min	Peak Name	Height mAU	Area mAU*min	Rel.Area %	Amount	Type
1	8.35	n.a.	287.086	101.555	96.10	n.a.	BM *
2	9.68	n.a.	10.043	4.124	3.90	n.a.	MB*
**Total:**			297.129	105.679	100.00	0.000	

362 xpw-xd-111-1p-rac-ic-80/20-1.0-230

Sample Name:	**xpw-xd-111-1p-rac-ic-80/20-1.0-230**	*Injection Volume:*	**20.0**
Vial Number:	**476**	*Channel:*	**UV_VIS_1**
Sample Type:	**standard**	*Wavelength:*	**230**
Control Program:	**Xu Pengwei**	*Bandwidth:*	**n.a.**
Quantif. Method:	**Xu Pengwei**	*Dilution Factor:*	**1.0000**
Recording Time:	**2016-3-18 10:09**	*Sample Weight:*	**1.0000**
Run Time (min):	**11.57**	*Sample Amount:*	**1.0000**

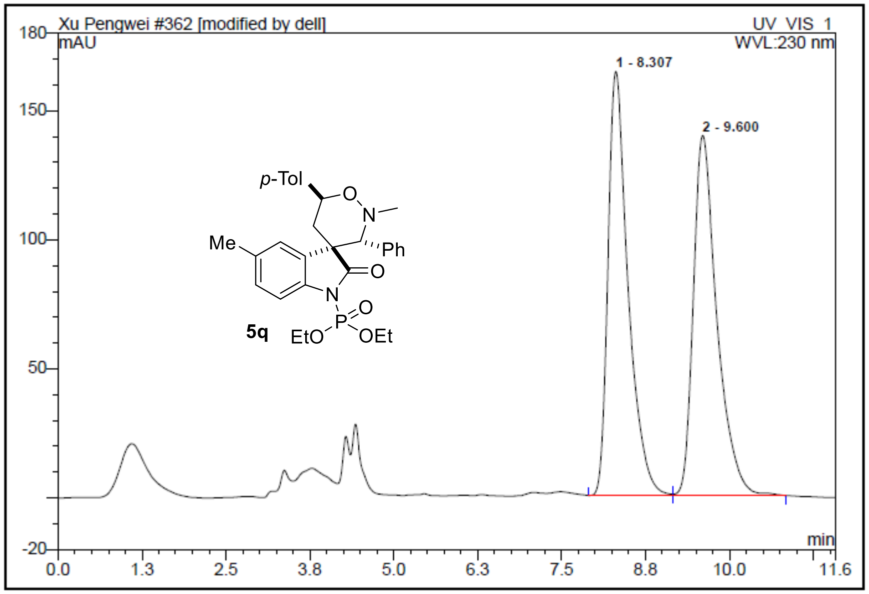

No.	Ret.Time min	Peak Name	Height mAU	Area mAU*min	Rel.Area %	Amount	Type
1	8.31	n.a.	164.333	57.087	50.10	n.a.	BM *
2	9.60	n.a.	139.510	56.850	49.90	n.a.	MB*
**Total:**			303.843	113.938	100.00	0.000	

Supplementary Figure 185. HPLC analysis for compound **5q**

363 xpw-xd-111-1s-asy-ic-80/20-1.0-230			
Sample Name:	xpw-xd-111-1s-asy-ic-80/20-1.0-230	Injection Volume:	20.0
Vial Number:	477	Channel:	UV_VIS_1
Sample Type:	standard	Wavelength:	230
Control Program:	Xu Pengwei	Bandwidth:	n.a.
Quantif. Method:	Xu Pengwei	Dilution Factor:	1.0000
Recording Time:	2016-3-18 14:09	Sample Weight:	1.0000
Run Time (min):	18.15	Sample Amount:	1.0000

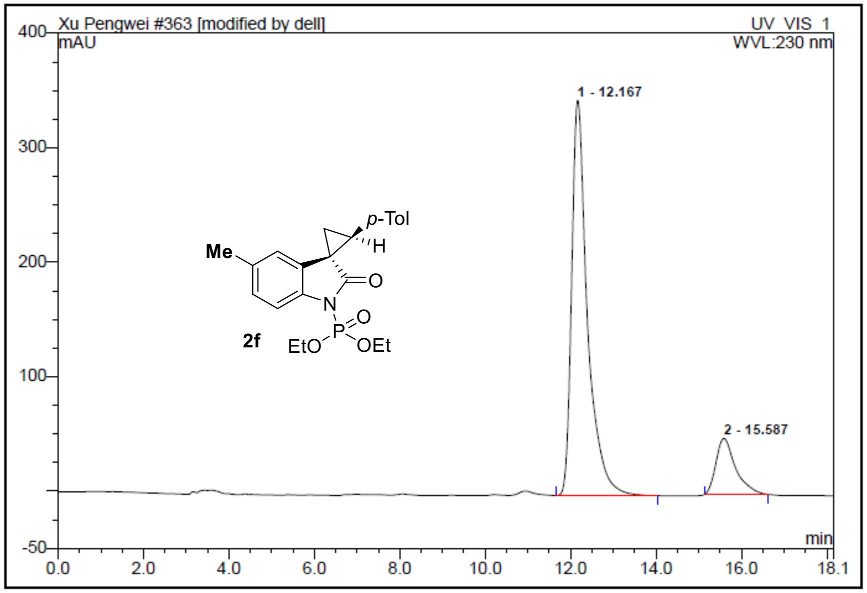

No.	Ret.Time min	Peak Name	Height mAU	Area mAU*min	Rel.Area %	Amount	Type
1	12.17	n.a.	345.349	147.689	85.02	n.a.	BMB
2	15.59	n.a.	48.879	26.018	14.98	n.a.	BMB*
Total:			394.229	173.707	100.00	0.000	

364 xpw-xd-111-1s-rac-ic-80/20-1.0-230			
Sample Name:	xpw-xd-111-1s-rac-ic-80/20-1.0-230	Injection Volume:	20.0
Vial Number:	478	Channel:	UV_VIS_1
Sample Type:	standard	Wavelength:	230
Control Program:	Xu Pengwei	Bandwidth:	n.a.
Quantif. Method:	Xu Pengwei	Dilution Factor:	1.0000
Recording Time:	2016-3-18 14:31	Sample Weight:	1.0000
Run Time (min):	18.22	Sample Amount:	1.0000

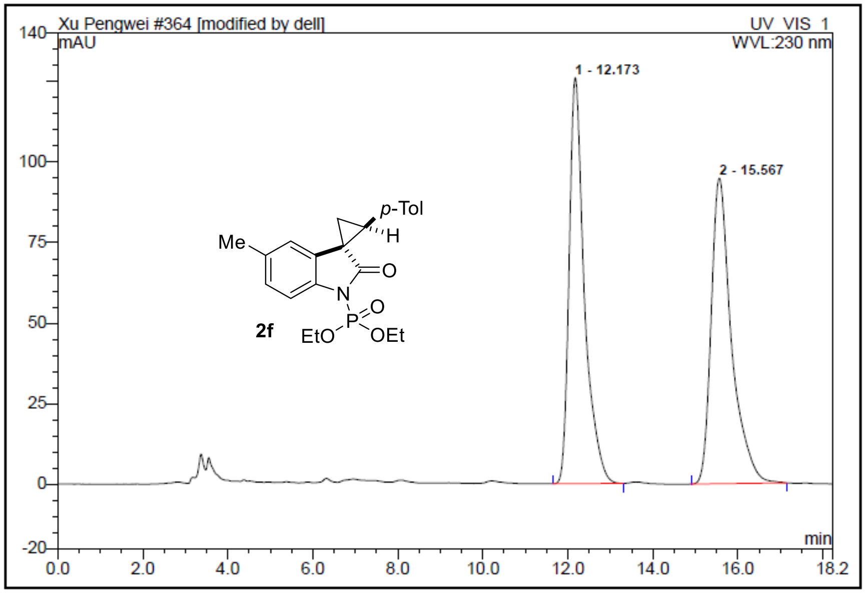

No.	Ret.Time min	Peak Name	Height mAU	Area mAU*min	Rel.Area %	Amount	Type
1	12.17	n.a.	126.108	52.431	49.96	n.a.	BMB*
2	15.57	n.a.	94.882	52.522	50.04	n.a.	BMB*
Total:			220.991	104.952	100.00	0.000	

Supplementary Figure 186. HPLC analysis for compound **2f**

357 xpw-xd-112-1p-asy-ic-80/20-1.0-230			
Sample Name:	**xpw-xd-112-1p-asy-ic-80/20-1.0-230**	*Injection Volume:*	**20.0**
Vial Number:	**468**	*Channel:*	**UV_VIS_1**
Sample Type:	**standard**	*Wavelength:*	**230**
Control Program:	**Xu Pengwei**	*Bandwidth:*	**n.a.**
Quantif. Method:	**Xu Pengwei**	*Dilution Factor:*	**1.0000**
Recording Time:	**2016-3-17 21:09**	*Sample Weight:*	**1.0000**
Run Time (min):	**14.52**	*Sample Amount:*	**1.0000**

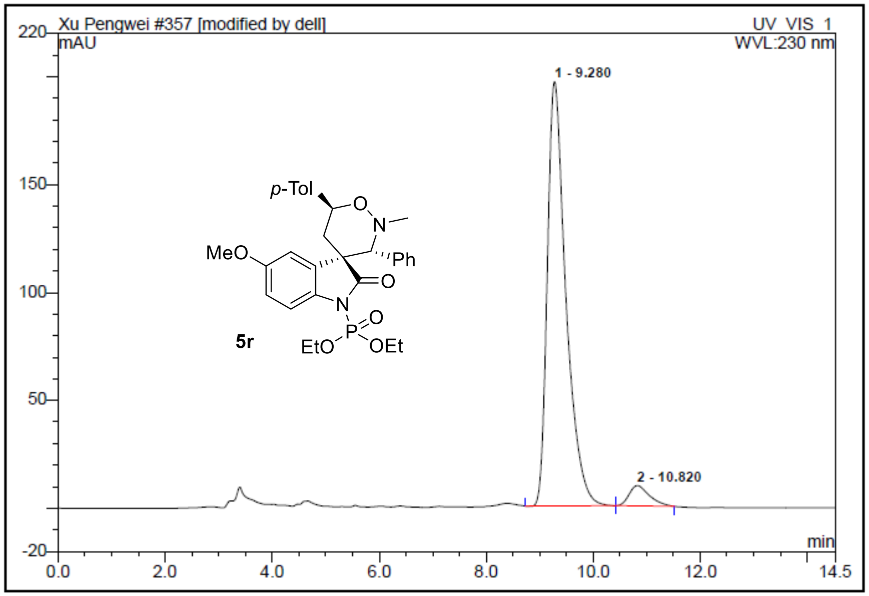

No.	Ret.Time min	Peak Name	Height mAU	Area mAU*min	Rel.Area %	Amount	Type
1	9.28	n.a.	196.595	79.055	95.00	n.a.	BMb*
2	10.82	n.a.	9.454	4.164	5.00	n.a.	bMB*
**Total:**			206.049	83.219	100.00	0.000	

358 xpw-xd-112-1p-rac-ic-80/20-1.0-230			
Sample Name:	**xpw-xd-112-1p-rac-ic-80/20-1.0-230**	*Injection Volume:*	**20.0**
Vial Number:	**470**	*Channel:*	**UV_VIS_1**
Sample Type:	**standard**	*Wavelength:*	**230**
Control Program:	**Xu Pengwei**	*Bandwidth:*	**n.a.**
Quantif. Method:	**Xu Pengwei**	*Dilution Factor:*	**1.0000**
Recording Time:	**2016-3-17 21:43**	*Sample Weight:*	**1.0000**
Run Time (min):	**13.73**	*Sample Amount:*	**1.0000**

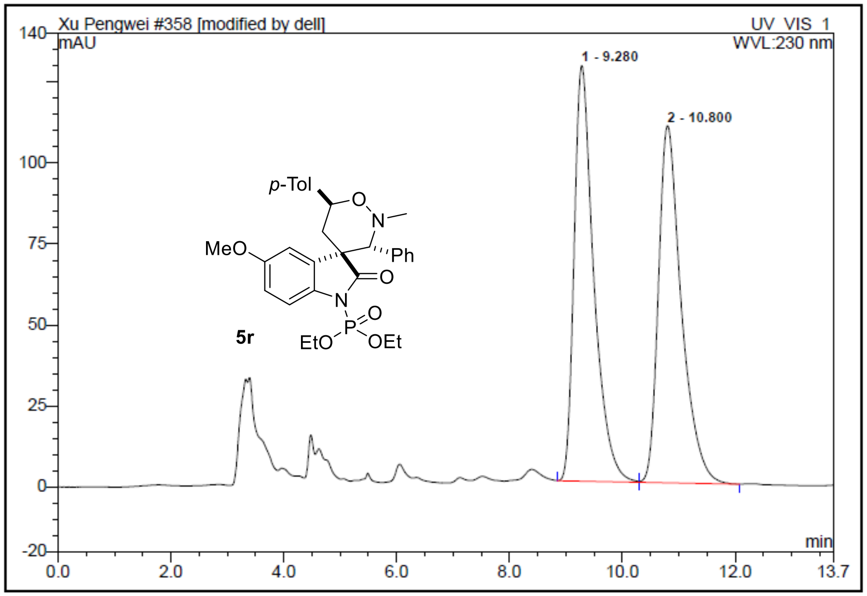

No.	Ret.Time min	Peak Name	Height mAU	Area mAU*min	Rel.Area %	Amount	Type
1	9.28	n.a.	128.411	51.778	49.93	n.a.	BM *
2	10.80	n.a.	110.342	51.921	50.07	n.a.	MB*
**Total:**			238.753	103.699	100.00	0.000	

Supplementary Figure 187. HPLC analysis for compound **5r**

**359 xpw-xd-112-1s-asy-ic-80/20-1.0-230**			
Sample Name:	**xpw-xd-112-1s-asy-ic-80/20-1.0-230**	*Injection Volume:*	**20.0**
Vial Number:	**471**	*Channel:*	**UV_VIS_1**
Sample Type:	**standard**	*Wavelength:*	**230**
Control Program:	**Xu Pengwei**	*Bandwidth:*	**n.a.**
Quantif. Method:	**Xu Pengwei**	*Dilution Factor:*	**1.0000**
Recording Time:	**2016-3-17 21:59**	*Sample Weight:*	**1.0000**
Run Time (min):	**27.93**	*Sample Amount:*	**1.0000**

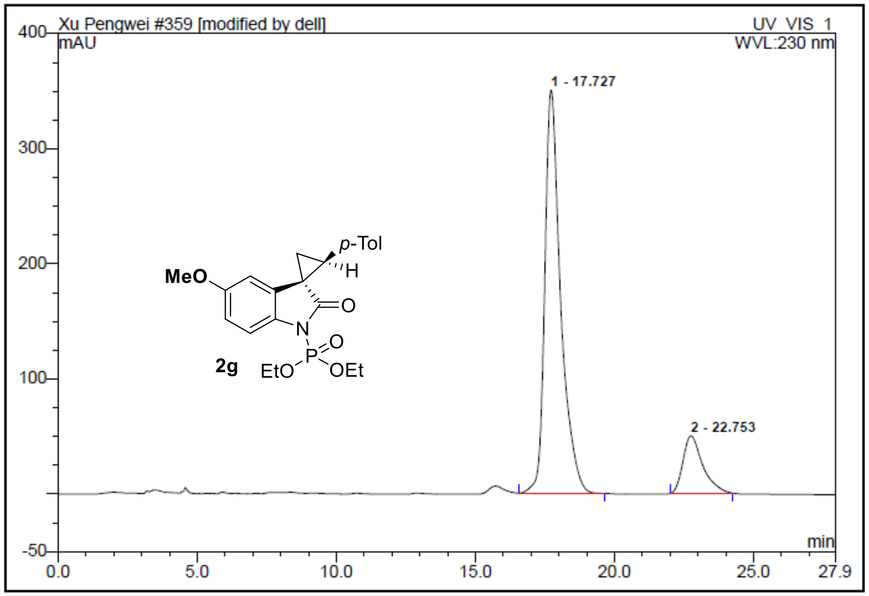

No.	Ret.Time min	Peak Name	Height mAU	Area mAU*min	Rel.Area %	Amount	Type
1	17.73	n.a.	350.410	232.273	85.02	n.a.	MB*
2	22.75	n.a.	49.845	40.913	14.98	n.a.	BMB*
Total:			400.255	273.186	100.00	0.000	

**360 xpw-xd-112-1s-rac-ic-80/20-1.0-230**			
Sample Name:	**xpw-xd-112-1s-rac-ic-80/20-1.0-230**	*Injection Volume:*	**20.0**
Vial Number:	**473**	*Channel:*	**UV_VIS_1**
Sample Type:	**standard**	*Wavelength:*	**230**
Control Program:	**Xu Pengwei**	*Bandwidth:*	**n.a.**
Quantif. Method:	**Xu Pengwei**	*Dilution Factor:*	**1.0000**
Recording Time:	**2016-3-17 22:55**	*Sample Weight:*	**1.0000**
Run Time (min):	**31.00**	*Sample Amount:*	**1.0000**

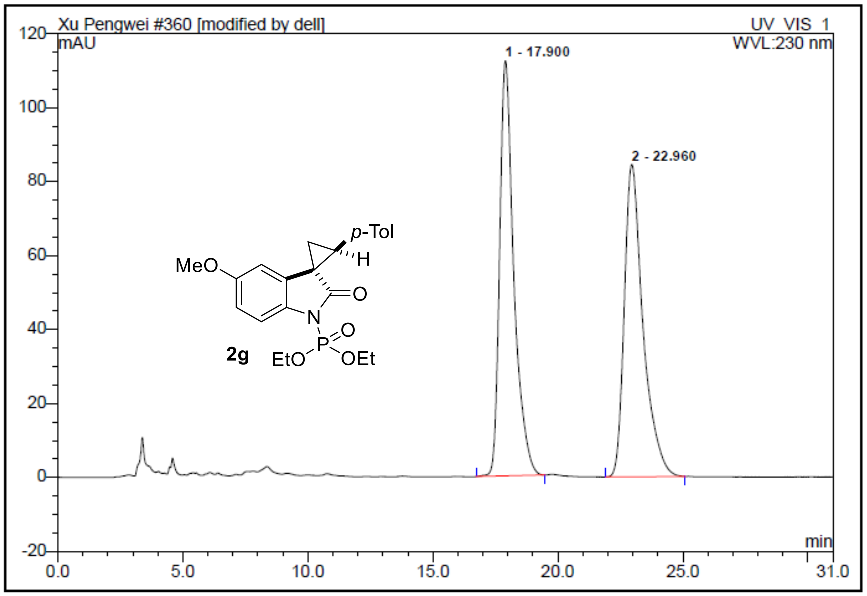

No.	Ret.Time min	Peak Name	Height mAU	Area mAU*min	Rel.Area %	Amount	Type
1	17.90	n.a.	112.333	73.738	50.12	n.a.	BMB*
2	22.96	n.a.	84.494	73.391	49.88	n.a.	BMB
Total:			196.827	147.128	100.00	0.000	

Supplementary Figure 188. HPLC analysis for compound **2g**

294 xpw-xd-81-1p-asy-ic-90/10-1.0-230

Sample Name:	**xpw-xd-81-1p-asy-ic-90/10-1.0-230**	*Injection Volume:*	**20.0**
Vial Number:	**398**	*Channel:*	**UV_VIS_1**
Sample Type:	**standard**	*Wavelength:*	**230**
Control Program:	**Xu Pengwei**	*Bandwidth:*	**n.a.**
Quantif. Method:	**Xu Pengwei**	*Dilution Factor:*	**1.0000**
Recording Time:	**2016-2-22 20:30**	*Sample Weight:*	**1.0000**
Run Time (min):	**14.17**	*Sample Amount:*	**1.0000**

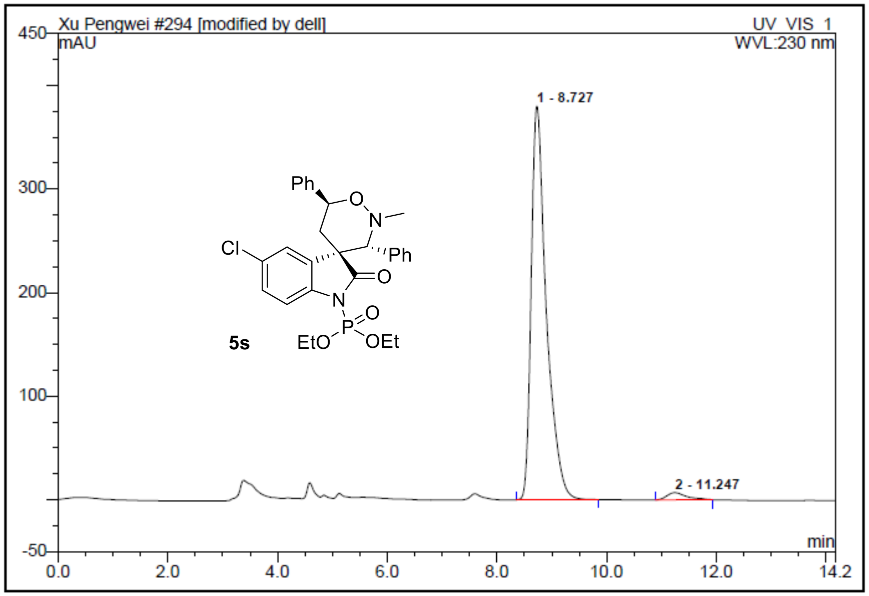

No.	Ret.Time min	Peak Name	Height mAU	Area mAU*min	Rel.Area %	Amount	Type
1	8.73	n.a.	380.001	121.504	97.61	n.a.	BMB*
2	11.25	n.a.	6.985	2.978	2.39	n.a.	BMB*
**Total:**			386.986	124.482	100.00	0.000	

295 xpw-xd-81-1p-rac-ic-90/10-1.0-230

Sample Name:	**xpw-xd-81-1p-rac-ic-90/10-1.0-230**	*Injection Volume:*	**20.0**
Vial Number:	**399**	*Channel:*	**UV_VIS_1**
Sample Type:	**standard**	*Wavelength:*	**230**
Control Program:	**Xu Pengwei**	*Bandwidth:*	**n.a.**
Quantif. Method:	**Xu Pengwei**	*Dilution Factor:*	**1.0000**
Recording Time:	**2016-2-22 20:46**	*Sample Weight:*	**1.0000**
Run Time (min):	**13.86**	*Sample Amount:*	**1.0000**

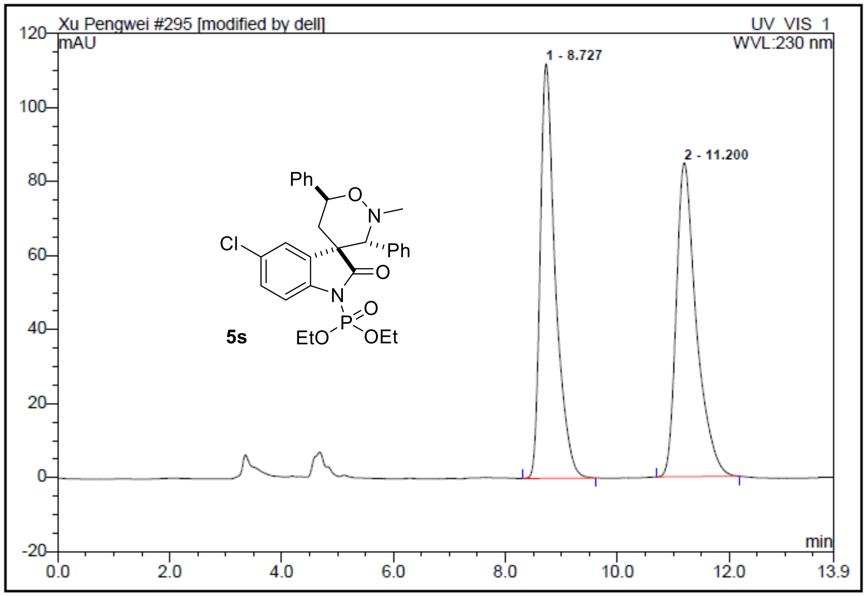

No.	Ret.Time min	Peak Name	Height mAU	Area mAU*min	Rel.Area %	Amount	Type
1	8.73	n.a.	112.008	35.221	49.89	n.a.	BMB*
2	11.20	n.a.	84.882	35.377	50.11	n.a.	BMB*
**Total:**			196.890	70.598	100.00	0.000	

Supplementary Figure 189. HPLC analysis for compound **5s**

290 xpw-xd-81-1s-asy-adh-90/10-1.0-230

Sample Name:	xpw-xd-81-1s-asy-adh-90/10-1.0-230	*Injection Volume:*	20.0
Vial Number:	394	*Channel:*	UV_VIS_1
Sample Type:	standard	*Wavelength:*	230
Control Program:	Xu Pengwei	*Bandwidth:*	n.a.
Quantif. Method:	Xu Pengwei	*Dilution Factor:*	1.0000
Recording Time:	2016-2-22 17:06	*Sample Weight:*	1.0000
Run Time (min):	20.51	*Sample Amount:*	1.0000

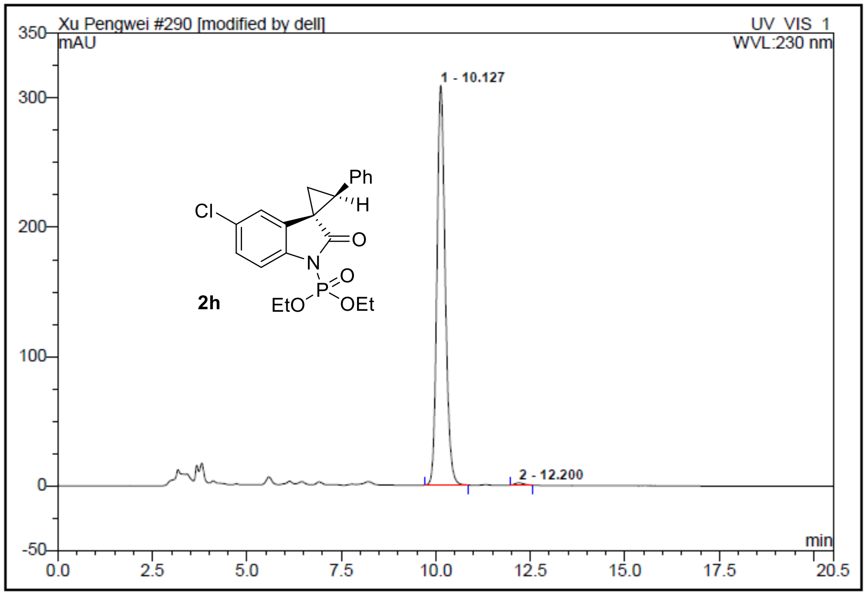

No.	Ret.Time min	Peak Name	Height mAU	Area mAU*min	Rel.Area %	Amount	Type
1	10.13	n.a.	309.123	78.705	99.38	n.a.	BMB*
2	12.20	n.a.	1.759	0.493	0.62	n.a.	BMB*
**Total:**			310.882	79.198	100.00	0.000	

291 xpw-xd-81-1s-rac-adh-90/10-1.0-230

Sample Name:	xpw-xd-81-1s-rac-adh-90/10-1.0-230	*Injection Volume:*	20.0
Vial Number:	395	*Channel:*	UV_VIS_1
Sample Type:	standard	*Wavelength:*	230
Control Program:	Xu Pengwei	*Bandwidth:*	n.a.
Quantif. Method:	Xu Pengwei	*Dilution Factor:*	1.0000
Recording Time:	2016-2-22 17:27	*Sample Weight:*	1.0000
Run Time (min):	14.03	*Sample Amount:*	1.0000

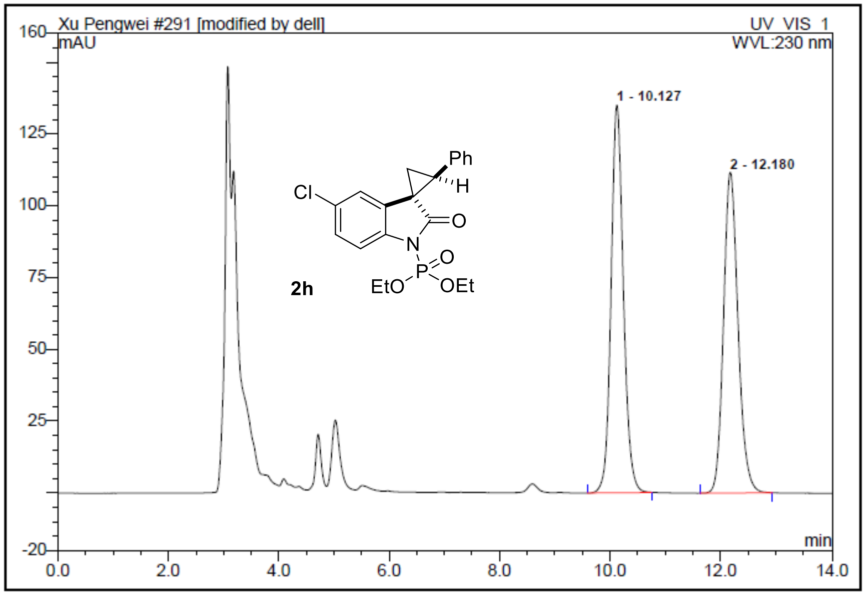

No.	Ret.Time min	Peak Name	Height mAU	Area mAU*min	Rel.Area %	Amount	Type
1	10.13	n.a.	135.018	34.944	50.62	n.a.	BMB*
2	12.18	n.a.	111.686	34.092	49.38	n.a.	BMB*
**Total:**			246.704	69.037	100.00	0.000	

Supplementary Figure 190. HPLC analysis for compound **2h**

284 xpw-xd-82-1p-asy-ic-90/10-1.0-230

Sample Name:	**xpw-xd-82-1p-asy-ic-90/10-1.0-230**	*Injection Volume:*	**20.0**
Vial Number:	**388**	*Channel:*	**UV_VIS_1**
Sample Type:	**standard**	*Wavelength:*	**230**
Control Program:	**Xu Pengwei**	*Bandwidth:*	**n.a.**
Quantif. Method:	**Xu Pengwei**	*Dilution Factor:*	**1.0000**
Recording Time:	**2016-2-20 16:20**	*Sample Weight:*	**1.0000**
Run Time (min):	**15.00**	*Sample Amount:*	**1.0000**

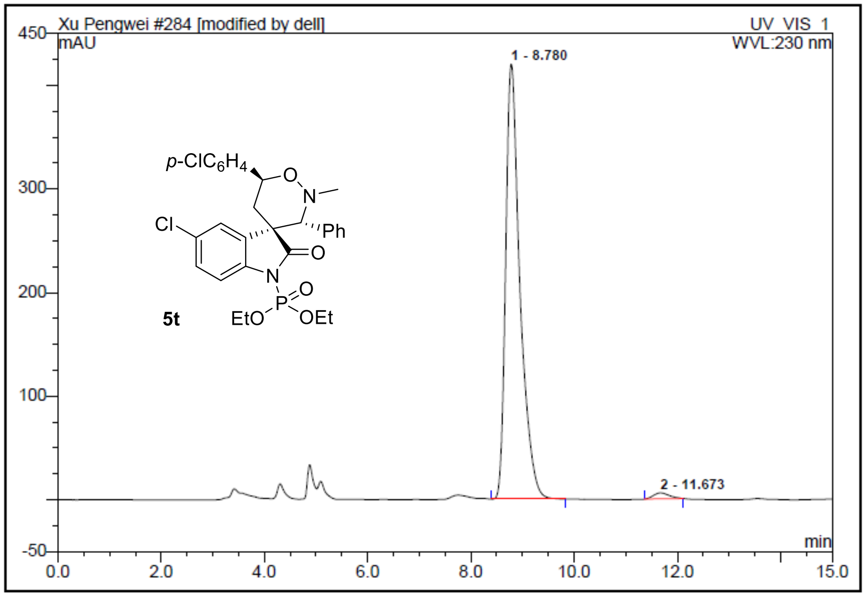

No.	Ret.Time min	Peak Name	Height mAU	Area mAU*min	Rel.Area %	Amount	Type
1	8.78	n.a.	419.851	134.835	98.52	n.a.	BMB*
2	11.67	n.a.	5.753	2.027	1.48	n.a.	BMB*
**Total:**			425.605	136.862	100.00	0.000	

285 xpw-xd-82-1p-rac-ic-90/10-1.0-230

Sample Name:	**xpw-xd-82-1p-rac-ic-90/10-1.0-230**	*Injection Volume:*	**20.0**
Vial Number:	**389**	*Channel:*	**UV_VIS_1**
Sample Type:	**standard**	*Wavelength:*	**230**
Control Program:	**Xu Pengwei**	*Bandwidth:*	**n.a.**
Quantif. Method:	**Xu Pengwei**	*Dilution Factor:*	**1.0000**
Recording Time:	**2016-2-20 16:36**	*Sample Weight:*	**1.0000**
Run Time (min):	**19.23**	*Sample Amount:*	**1.0000**

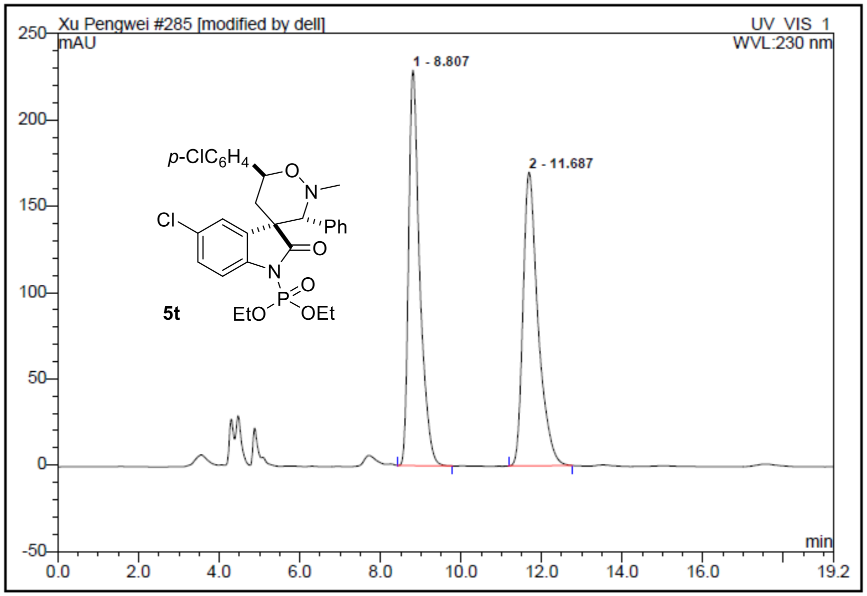

No.	Ret.Time min	Peak Name	Height mAU	Area mAU*min	Rel.Area %	Amount	Type
1	8.81	n.a.	229.082	73.451	50.00	n.a.	BMB*
2	11.69	n.a.	170.170	73.460	50.00	n.a.	BMB*
**Total:**			399.252	146.911	100.00	0.000	

Supplementary Figure 191. HPLC analysis for compound **5t**

286 xpw-xd-82-1s-asy-adh-90/10-1.0-230

Sample Name:	**xpw-xd-82-1s-asy-adh-90/10-1.0-230**	*Injection Volume:*	**20.0**
Vial Number:	**390**	*Channel:*	**UV_VIS_1**
Sample Type:	**standard**	*Wavelength:*	**230**
Control Program:	**Xu Pengwei**	*Bandwidth:*	**n.a.**
Quantif. Method:	**Xu Pengwei**	*Dilution Factor:*	**1.0000**
Recording Time:	**2016-2-20 17:07**	*Sample Weight:*	**1.0000**
Run Time (min):	**15.10**	*Sample Amount:*	**1.0000**

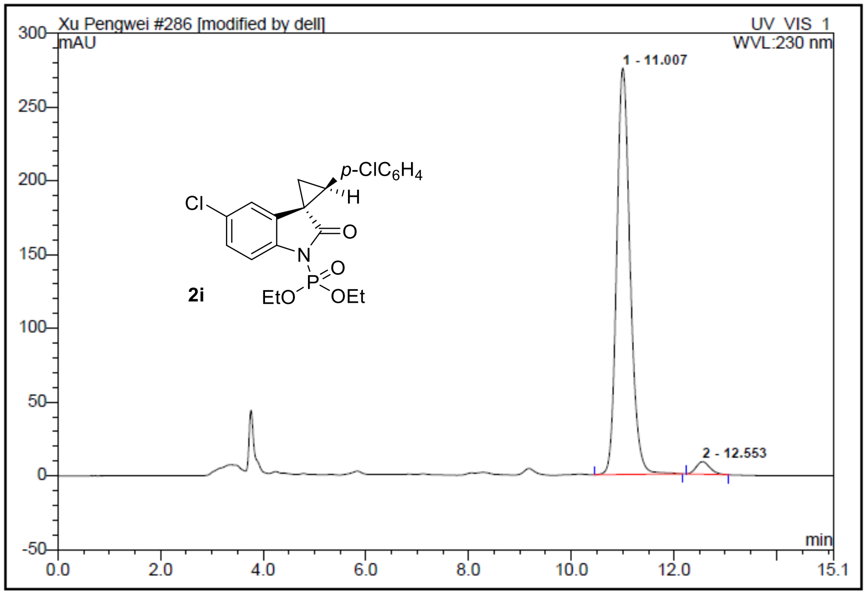

No.	Ret.Time min	Peak Name	Height mAU	Area mAU*min	Rel.Area %	Amount	Type
1	11.01	n.a.	275.606	83.476	96.91	n.a.	BMB*
2	12.55	n.a.	8.527	2.660	3.09	n.a.	BMB*
**Total:**			284.134	86.135	100.00	0.000	

287 xpw-xd-82-1s-rac-adh-90/10-1.0-230

Sample Name:	**xpw-xd-82-1s-rac-adh-90/10-1.0-230**	*Injection Volume:*	**20.0**
Vial Number:	**391**	*Channel:*	**UV_VIS_1**
Sample Type:	**standard**	*Wavelength:*	**230**
Control Program:	**Xu Pengwei**	*Bandwidth:*	**n.a.**
Quantif. Method:	**Xu Pengwei**	*Dilution Factor:*	**1.0000**
Recording Time:	**2016-2-20 17:23**	*Sample Weight:*	**1.0000**
Run Time (min):	**15.23**	*Sample Amount:*	**1.0000**

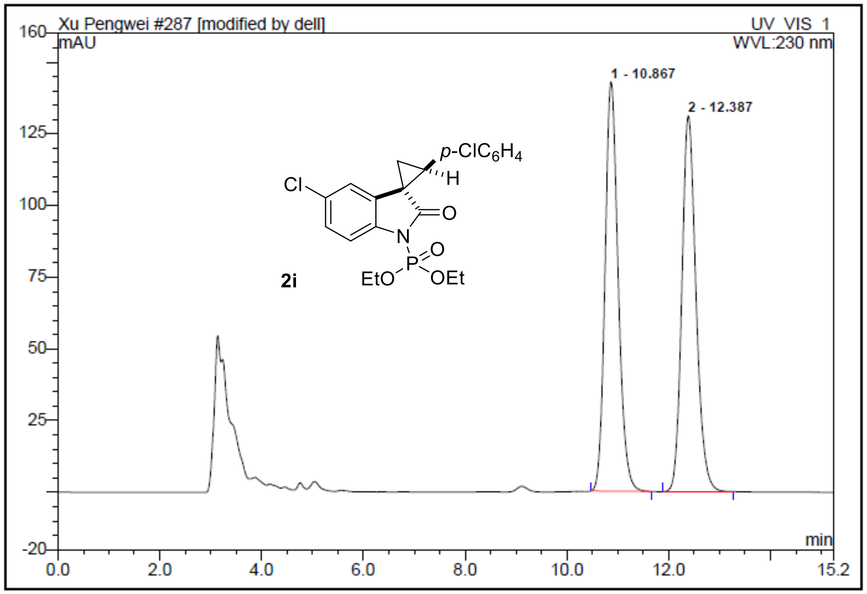

No.	Ret.Time min	Peak Name	Height mAU	Area mAU*min	Rel.Area %	Amount	Type
1	10.87	n.a.	142.708	42.623	50.15	n.a.	BMB*
2	12.39	n.a.	131.024	42.373	49.85	n.a.	BMB*
**Total:**			273.732	84.996	100.00	0.000	

Supplementary Figure 192. HPLC analysis for compound **2i**

296 xpw-xd-80-1p-asy-ic-90/10-1.0-230

Sample Name:	xpw-xd-80-1p-asy-ic-90/10-1.0-230	*Injection Volume:*	20.0
Vial Number:	400	*Channel:*	UV_VIS_1
Sample Type:	standard	*Wavelength:*	230
Control Program:	Xu Pengwei	*Bandwidth:*	n.a.
Quantif. Method:	Xu Pengwei	*Dilution Factor:*	1.0000
Recording Time:	2016-2-23 10:52	*Sample Weight:*	1.0000
Run Time (min):	17.63	*Sample Amount:*	1.0000

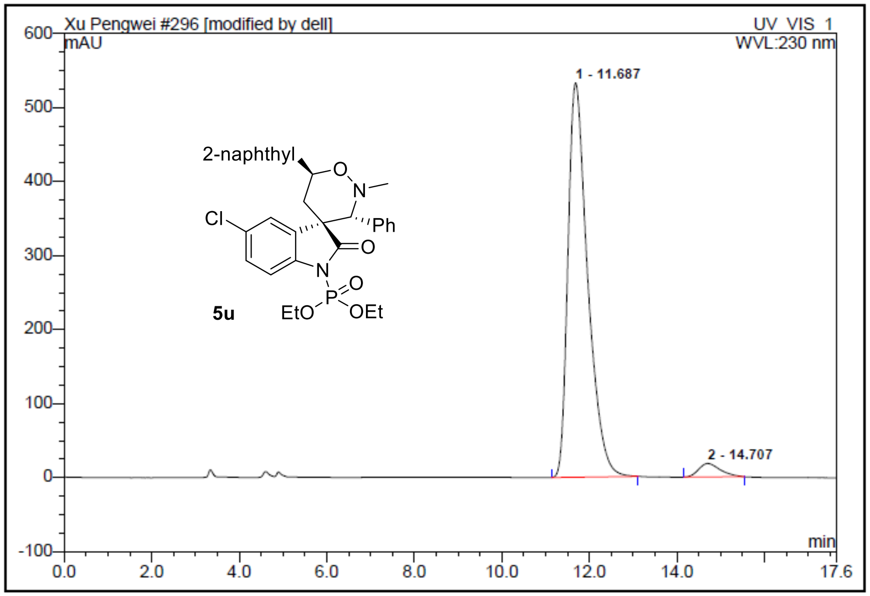

No.	Ret.Time min	Peak Name	Height mAU	Area mAU*min	Rel.Area %	Amount	Type
1	11.69	n.a.	533.107	277.950	96.37	n.a.	BMB*
2	14.71	n.a.	18.184	10.482	3.63	n.a.	BMB*
**Total:**			551.291	288.432	100.00	0.000	

298 xpw-xd-80-1p-rac-ic-90/10-1.0-230

Sample Name:	xpw-xd-80-1p-rac-ic-90/10-1.0-230	*Injection Volume:*	20.0
Vial Number:	402	*Channel:*	UV_VIS_1
Sample Type:	standard	*Wavelength:*	230
Control Program:	Xu Pengwei	*Bandwidth:*	n.a.
Quantif. Method:	Xu Pengwei	*Dilution Factor:*	1.0000
Recording Time:	2016-2-23 11:32	*Sample Weight:*	1.0000
Run Time (min):	17.71	*Sample Amount:*	1.0000

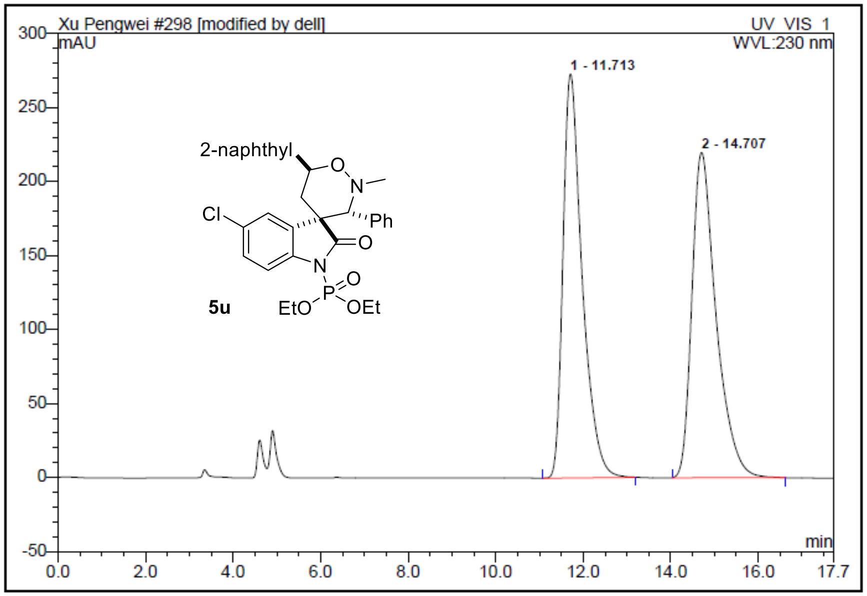

No.	Ret.Time min	Peak Name	Height mAU	Area mAU*min	Rel.Area %	Amount	Type
1	11.71	n.a.	272.998	138.497	49.63	n.a.	BMB*
2	14.71	n.a.	219.741	140.579	50.37	n.a.	BMB*
**Total:**			492.739	279.077	100.00	0.000	

Supplementary Figure 193. HPLC analysis for compound **5u**

288 xpw-xd-80-1s-asy-odh-90/10-1.0-230

Sample Name:	xpw-xd-80-1s-asy-odh-90/10-1.0-230	*Injection Volume:*	20.0
Vial Number:	392	*Channel:*	UV_VIS_1
Sample Type:	standard	*Wavelength:*	230
Control Program:	Xu Pengwei	*Bandwidth:*	n.a.
Quantif. Method:	Xu Pengwei	*Dilution Factor:*	1.0000
Recording Time:	2016-2-22 15:17	*Sample Weight:*	1.0000
Run Time (min):	16.84	*Sample Amount:*	1.0000

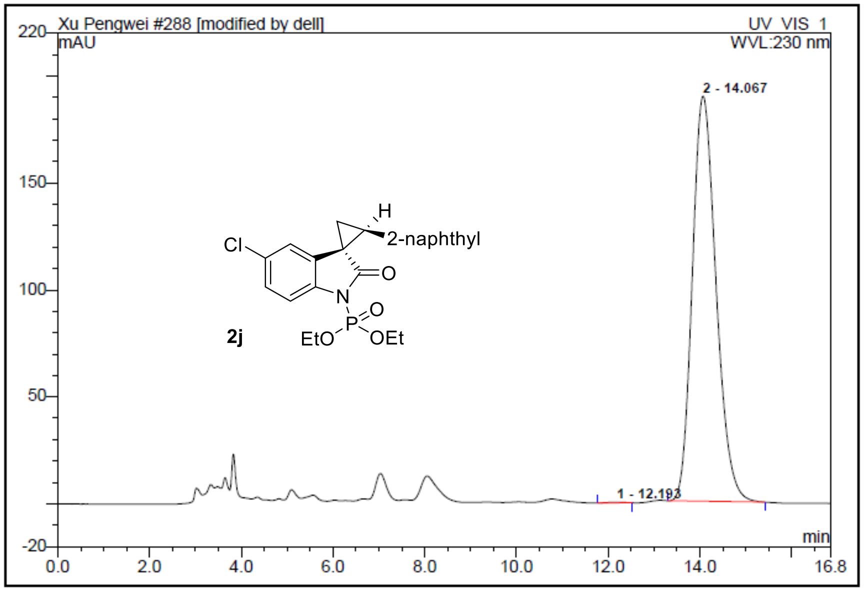

No.	Ret.Time min	Peak Name	Height mAU	Area mAU*min	Rel.Area %	Amount	Type
1	12.19	n.a.	0.305	0.123	0.11	n.a.	BMB*
2	14.07	n.a.	189.399	113.633	99.89	n.a.	BMB*
Total:			189.704	113.757	100.00	0.000	

289 xpw-xd-80-1s-rac-odh-90/10-1.0-230

Sample Name:	xpw-xd-80-1s-rac-odh-90/10-1.0-230	*Injection Volume:*	20.0
Vial Number:	393	*Channel:*	UV_VIS_1
Sample Type:	standard	*Wavelength:*	230
Control Program:	Xu Pengwei	*Bandwidth:*	n.a.
Quantif. Method:	Xu Pengwei	*Dilution Factor:*	1.0000
Recording Time:	2016-2-22 15:34	*Sample Weight:*	1.0000
Run Time (min):	16.57	*Sample Amount:*	1.0000

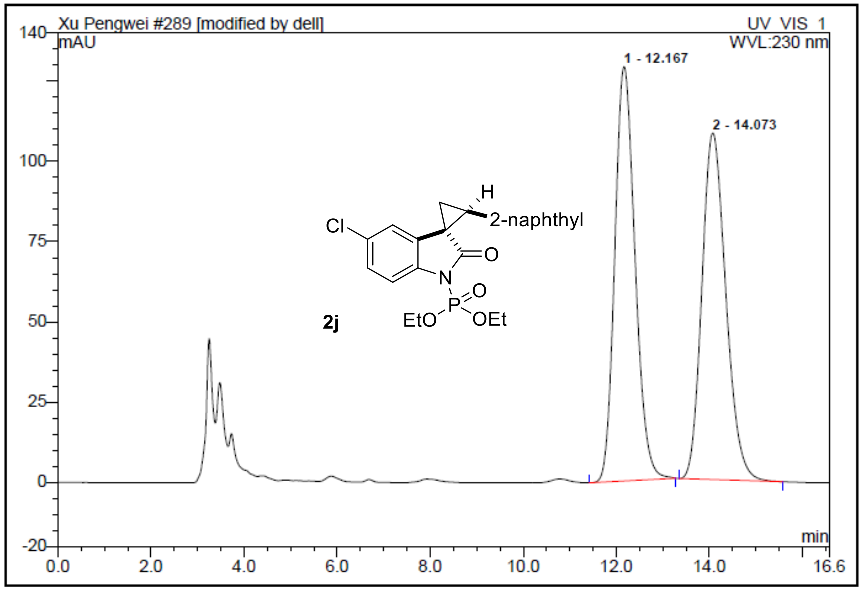

No.	Ret.Time min	Peak Name	Height mAU	Area mAU*min	Rel.Area %	Amount	Type
1	12.17	n.a.	129.114	64.994	50.19	n.a.	BMB*
2	14.07	n.a.	108.037	64.507	49.81	n.a.	BMB*
Total:			237.151	129.501	100.00	0.000	

Supplementary Figure 194. HPLC analysis for compound **2j**

431 xpw-xe-66-1p-asy-ic-90/10-1.0-230

Sample Name:	**xpw-xe-66-1p-asy-ic-90/10-1.0-230**	*Injection Volume:*	**20.0**
Vial Number:	**546**	*Channel:*	**UV_VIS_1**
Sample Type:	**standard**	*Wavelength:*	**230**
Control Program:	**Xu Pengwei**	*Bandwidth:*	**n.a.**
Quantif. Method:	**Xu Pengwei**	*Dilution Factor:*	**1.0000**
Recording Time:	**2016-6-3 9:22**	*Sample Weight:*	**1.0000**
Run Time (min):	**14.61**	*Sample Amount:*	**1.0000**

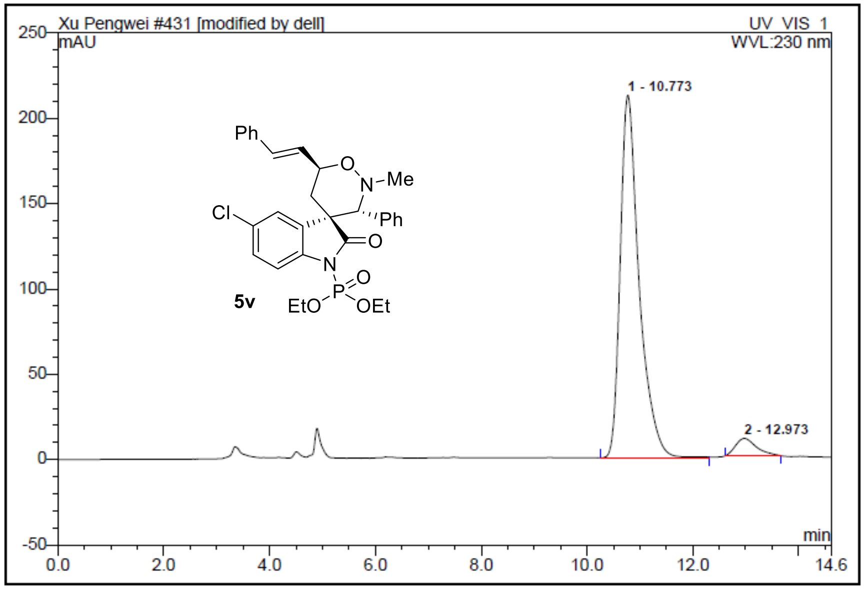

No.	Ret.Time min	Peak Name	Height mAU	Area mAU*min	Rel.Area %	Amount	Type
1	10.77	n.a.	212.477	88.073	95.02	n.a.	BM *
2	12.97	n.a.	10.078	4.614	4.98	n.a.	BMB*
Total:			222.555	92.687	100.00	0.000	

432 xpw-xe-66-1p-rac-ic-90/10-1.0-230

Sample Name:	**xpw-xe-66-1p-rac-ic-90/10-1.0-230**	*Injection Volume:*	**20.0**
Vial Number:	**547**	*Channel:*	**UV_VIS_1**
Sample Type:	**standard**	*Wavelength:*	**230**
Control Program:	**Xu Pengwei**	*Bandwidth:*	**n.a.**
Quantif. Method:	**Xu Pengwei**	*Dilution Factor:*	**1.0000**
Recording Time:	**2016-6-3 9:38**	*Sample Weight:*	**1.0000**
Run Time (min):	**14.62**	*Sample Amount:*	**1.0000**

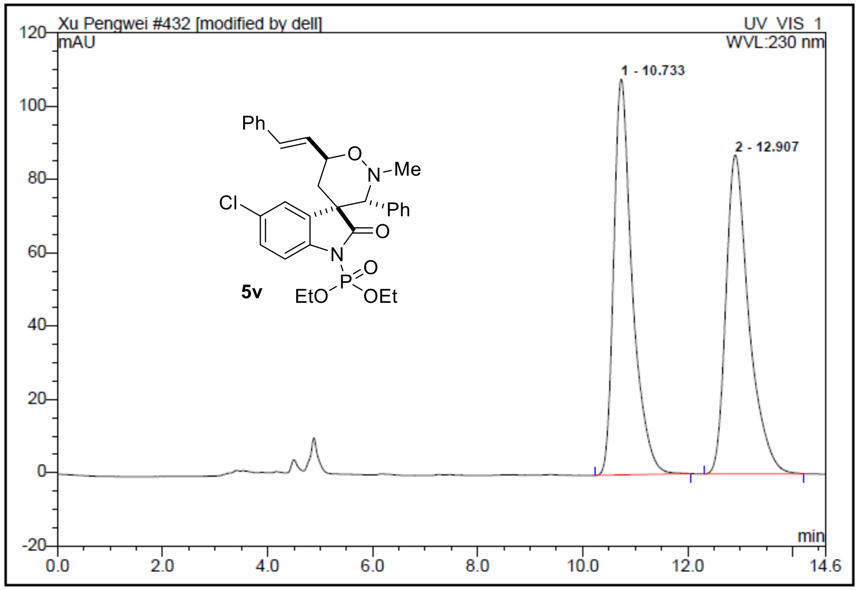

No.	Ret.Time min	Peak Name	Height mAU	Area mAU*min	Rel.Area %	Amount	Type
1	10.73	n.a.	108.063	44.230	50.02	n.a.	BMB
2	12.91	n.a.	87.030	44.193	49.98	n.a.	BMB
Total:			195.094	88.423	100.00	0.000	

Supplementary Figure 195. HPLC analysis for compound **5v**

490 xpw-xd-66-1s-asy-adh-85/15-1.0-230

Sample Name:	xpw-xd-66-1s-asy-adh-85/15-1.0-230	*Injection Volume:*	20.0
Vial Number:	605	*Channel:*	UV_VIS_1
Sample Type:	standard	*Wavelength:*	230
Control Program:	Xu Pengwei	*Bandwidth:*	n.a.
Quantif. Method:	Xu Pengwei	*Dilution Factor:*	1.0000
Recording Time:	2016-6-22 18:33	*Sample Weight:*	1.0000
Run Time (min):	13.29	*Sample Amount:*	1.0000

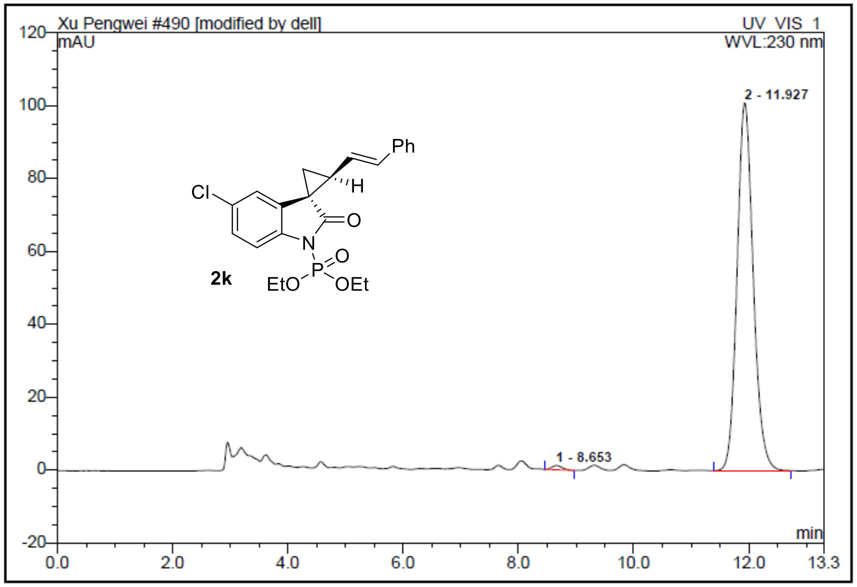

No.	Ret.Time min	Peak Name	Height mAU	Area mAU*min	Rel.Area %	Amount	Type
1	8.65	n.a.	1.147	0.241	0.73	n.a.	BMB*
2	11.93	n.a.	100.985	32.842	99.27	n.a.	BMB*
Total:			102.131	33.083	100.00	0.000	

491 xpw-xd-66-1s-rac-adh-85/15-1.0-230

Sample Name:	xpw-xd-66-1s-rac-adh-85/15-1.0-230	*Injection Volume:*	20.0
Vial Number:	606	*Channel:*	UV_VIS_1
Sample Type:	standard	*Wavelength:*	230
Control Program:	Xu Pengwei	*Bandwidth:*	n.a.
Quantif. Method:	Xu Pengwei	*Dilution Factor:*	1.0000
Recording Time:	2016-6-22 18:48	*Sample Weight:*	1.0000
Run Time (min):	13.53	*Sample Amount:*	1.0000

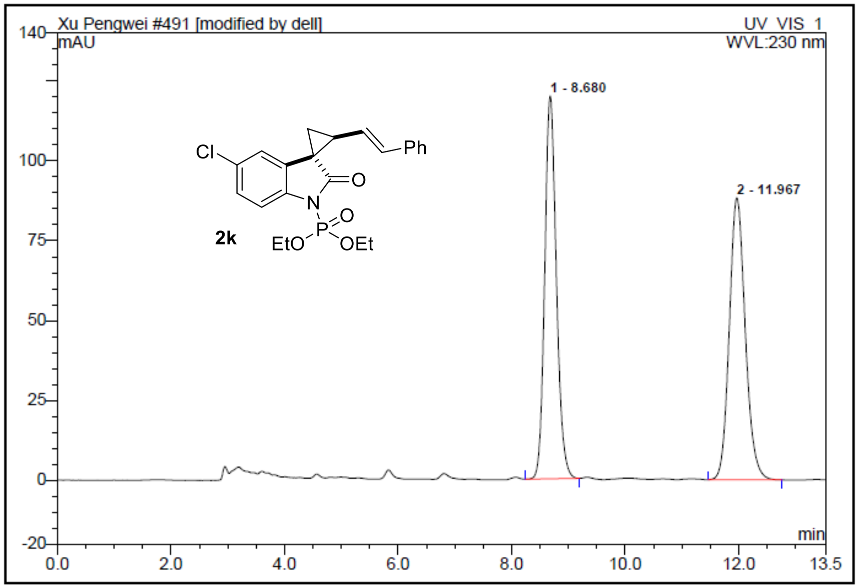

No.	Ret.Time min	Peak Name	Height mAU	Area mAU*min	Rel.Area %	Amount	Type
1	8.68	n.a.	119.950	28.474	49.70	n.a.	BMB*
2	11.97	n.a.	88.287	28.815	50.30	n.a.	BMB
Total:			208.237	57.289	100.00	0.000	

Supplementary Figure 196. HPLC analysis for compound **2k**

570 xpw-xe-134-1p-asy-if-90/10-0.8-230

Sample Name:	**xpw-xe-134-1p-asy-if-90/10-0.8-230**	*Injection Volume:*	**20.0**
Vial Number:	**687**	*Channel:*	**UV_VIS_1**
Sample Type:	**standard**	*Wavelength:*	**230**
Control Program:	**Xu Pengwei**	*Bandwidth:*	**n.a.**
Quantif. Method:	**Xu Pengwei**	*Dilution Factor:*	**1.0000**
Recording Time:	**2016-8-17 20:14**	*Sample Weight:*	**1.0000**
Run Time (min):	**16.05**	*Sample Amount:*	**1.0000**

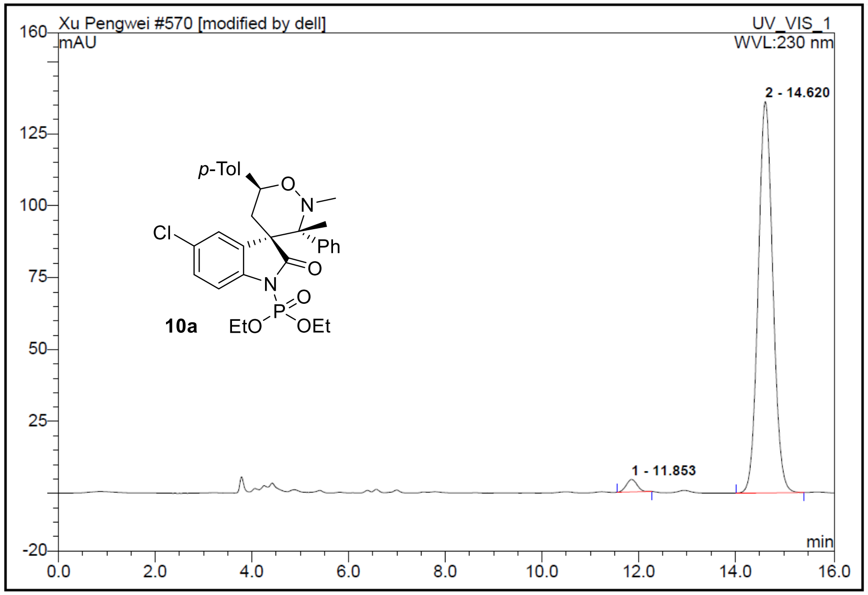

No.	Ret.Time min	Peak Name	Height mAU	Area mAU*min	Rel.Area %	Amount	Type
1	11.85	n.a.	4.304	1.119	2.28	n.a.	BMB*
2	14.62	n.a.	136.108	47.861	97.72	n.a.	BMB*
**Total:**			140.412	48.980	100.00	0.000	

572 xpw-xe-134-1p-rac-if-90/10-0.8-230

Sample Name:	**xpw-xe-134-1p-rac-if-90/10-0.8-230**	*Injection Volume:*	**20.0**
Vial Number:	**689**	*Channel:*	**UV_VIS_1**
Sample Type:	**standard**	*Wavelength:*	**230**
Control Program:	**Xu Pengwei**	*Bandwidth:*	**n.a.**
Quantif. Method:	**Xu Pengwei**	*Dilution Factor:*	**1.0000**
Recording Time:	**2016-8-17 20:47**	*Sample Weight:*	**1.0000**
Run Time (min):	**15.94**	*Sample Amount:*	**1.0000**

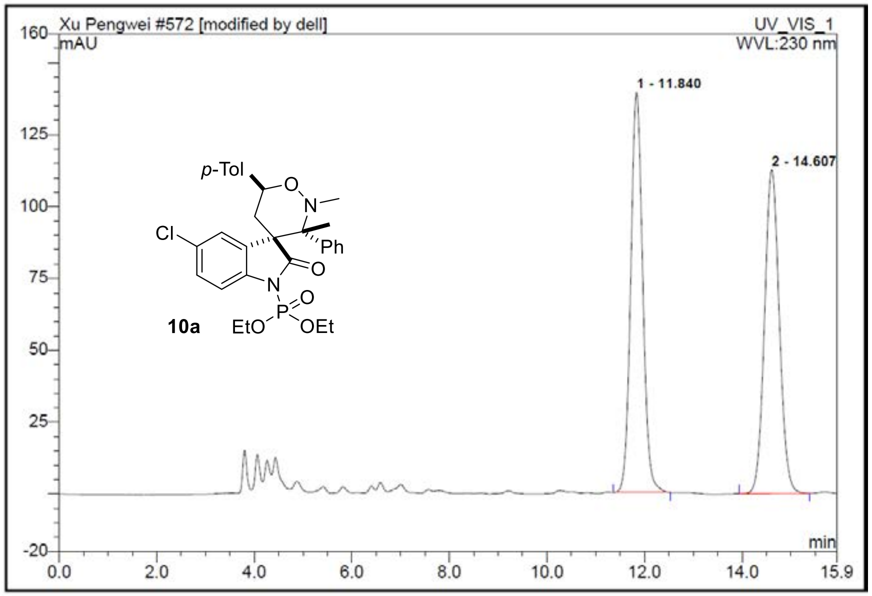

No.	Ret.Time min	Peak Name	Height mAU	Area mAU*min	Rel.Area %	Amount	Type
1	11.84	n.a.	139.231	39.644	50.04	n.a.	BMB*
2	14.61	n.a.	112.699	39.583	49.96	n.a.	BMB*
**Total:**			251.930	79.228	100.00	0.000	

Supplementary Figure 197. HPLC analysis for compound **10a**

597 xpw-xe-149-1p-asy-if-90/10-1.0-230

Sample Name:	**xpw-xe-149-1p-asy-if-90/10-1.0-230**	*Injection Volume:*	**20.0**
Vial Number:	**715**	*Channel:*	**UV_VIS_1**
Sample Type:	**standard**	*Wavelength:*	**230**
Control Program:	**Xu Pengwei**	*Bandwidth:*	**n.a.**
Quantif. Method:	**Xu Pengwei**	*Dilution Factor:*	**1.0000**
Recording Time:	**2016-8-25 16:20**	*Sample Weight:*	**1.0000**
Run Time (min):	**14.00**	*Sample Amount:*	**1.0000**

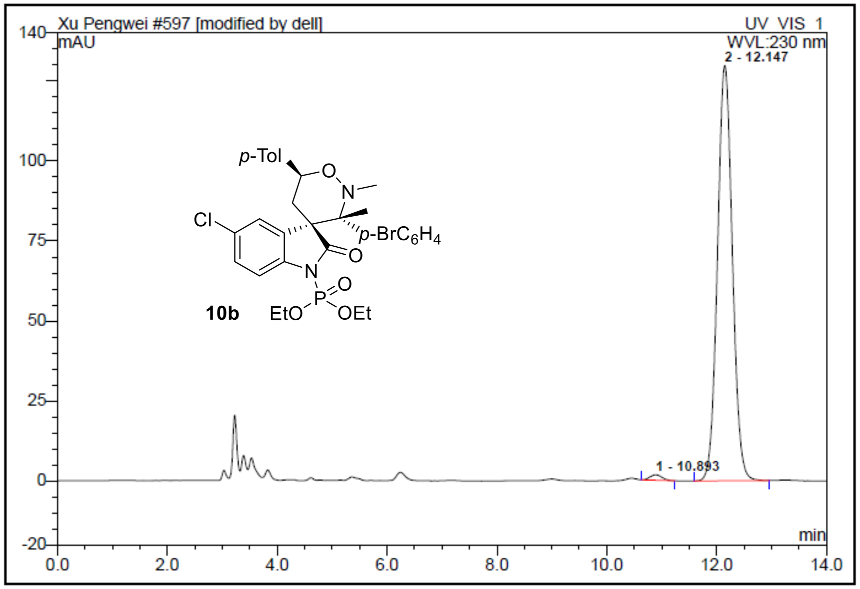

No.	Ret.Time min	Peak Name	Height mAU	Area mAU*min	Rel.Area %	Amount	Type
1	10.89	n.a.	1.713	0.436	1.06	n.a.	BMB*
2	12.15	n.a.	129.818	40.850	98.94	n.a.	BMB*
**Total:**			131.530	41.286	100.00	0.000	

598 xpw-xe-149-1p-rac-if-90/10-1.0-230

Sample Name:	**xpw-xe-149-1p-rac-if-90/10-1.0-230**	*Injection Volume:*	**20.0**
Vial Number:	**716**	*Channel:*	**UV_VIS_1**
Sample Type:	**standard**	*Wavelength:*	**230**
Control Program:	**Xu Pengwei**	*Bandwidth:*	**n.a.**
Quantif. Method:	**Xu Pengwei**	*Dilution Factor:*	**1.0000**
Recording Time:	**2016-8-25 16:36**	*Sample Weight:*	**1.0000**
Run Time (min):	**14.00**	*Sample Amount:*	**1.0000**

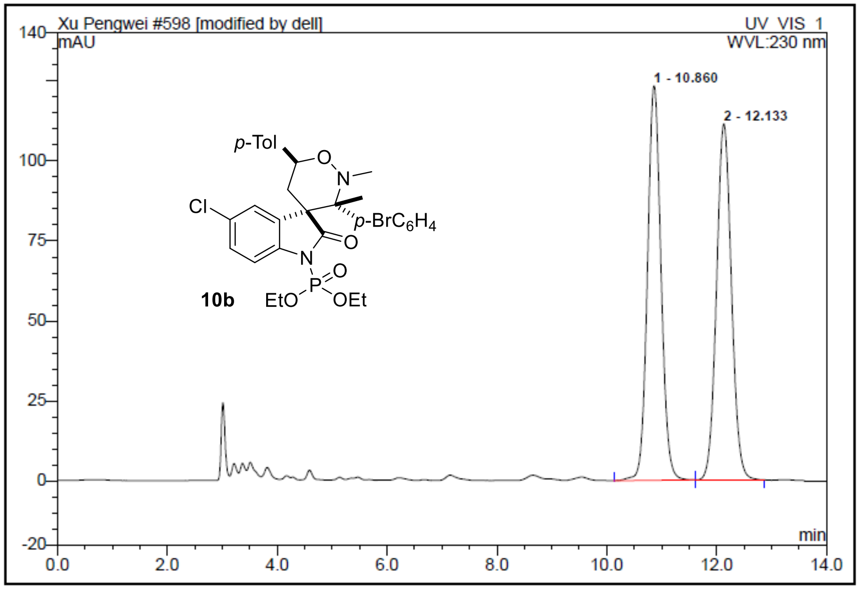

No.	Ret.Time min	Peak Name	Height mAU	Area mAU*min	Rel.Area %	Amount	Type
1	10.86	n.a.	123.383	35.273	50.11	n.a.	BMB
2	12.13	n.a.	111.364	35.118	49.89	n.a.	BMB*
**Total:**			234.747	70.391	100.00	0.000	

Supplementary Figure 198. HPLC analysis for compound **10b**

601 xpw-xe-150-1p-asy-ic+if-90/10-1.0-230			
Sample Name:	xpw-xe-150-1p-asy-ic+if-90/10-1.0-230	*Injection Volume:*	20.0
Vial Number:	719	*Channel:*	UV_VIS_1
Sample Type:	standard	*Wavelength:*	230
Control Program:	Xu Pengwei	*Bandwidth:*	n.a.
Quantif. Method:	Xu Pengwei	*Dilution Factor:*	1.0000
Recording Time:	2016-8-25 19:04	*Sample Weight:*	1.0000
Run Time (min):	23.00	*Sample Amount:*	1.0000

602 xpw-xe-150-1p-rac-ic+if-90/10-1.0-230			
Sample Name:	xpw-xe-150-1p-rac-ic+if-90/10-1.0-230	*Injection Volume:*	20.0
Vial Number:	720	*Channel:*	UV_VIS_1
Sample Type:	standard	*Wavelength:*	230
Control Program:	Xu Pengwei	*Bandwidth:*	n.a.
Quantif. Method:	Xu Pengwei	*Dilution Factor:*	1.0000
Recording Time:	2016-8-25 19:28	*Sample Weight:*	1.0000
Run Time (min):	23.00	*Sample Amount:*	1.0000

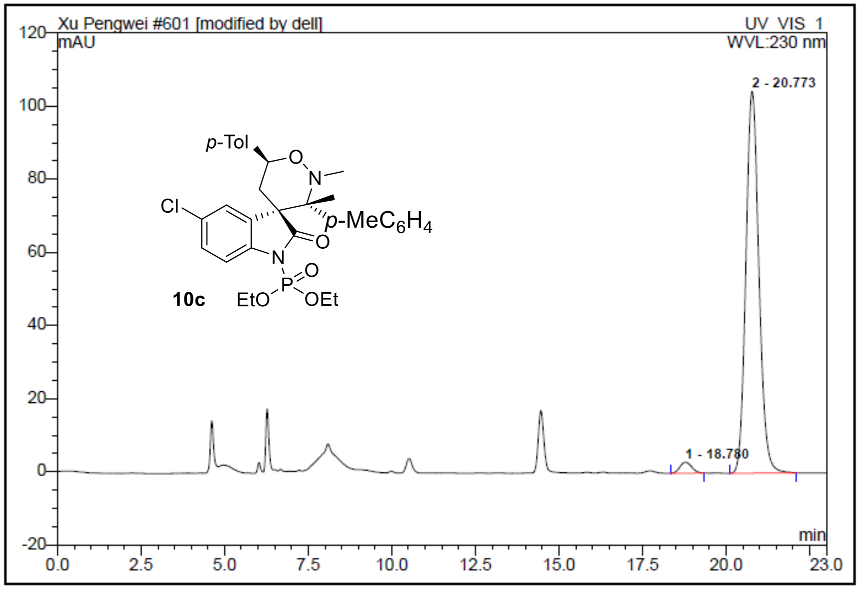

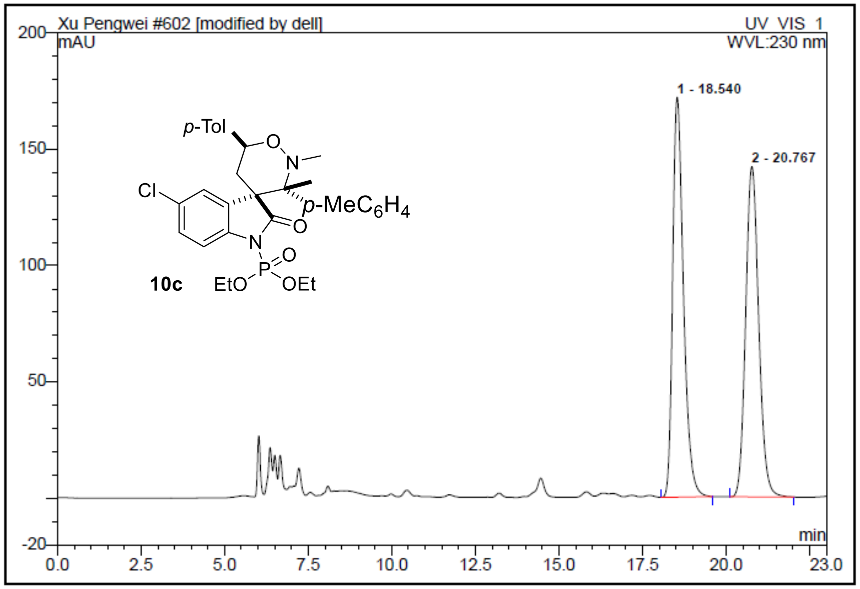

No.	Ret.Time min	Peak Name	Height mAU	Area mAU*min	Rel.Area %	Amount	Type
1	18.78	n.a.	3.037	1.245	2.57	n.a.	BMB
2	20.77	n.a.	104.401	47.243	97.43	n.a.	BMB*
Total:			107.438	48.487	100.00	0.000	

No.	Ret.Time min	Peak Name	Height mAU	Area mAU*min	Rel.Area %	Amount	Type
1	18.54	n.a.	171.865	64.317	50.01	n.a.	BMB*
2	20.77	n.a.	141.976	64.290	49.99	n.a.	BMB
Total:			313.841	128.607	100.00	0.000	

Supplementary Figure 199. HPLC analysis for compound **10c**

587 xpw-xe-146-1p-asy-ic+if-90/10-1.0-230

Sample Name:	xpw-xe-146-1p-asy-ic+if-90/10-1.0-230	*Injection Volume:*	20.0
Vial Number:	705	*Channel:*	UV_VIS_1
Sample Type:	standard	*Wavelength:*	230
Control Program:	Xu Pengwei	*Bandwidth:*	n.a.
Quantif. Method:	Xu Pengwei	*Dilution Factor:*	1.0000
Recording Time:	2016-8-23 13:40	*Sample Weight:*	1.0000
Run Time (min):	22.90	*Sample Amount:*	1.0000

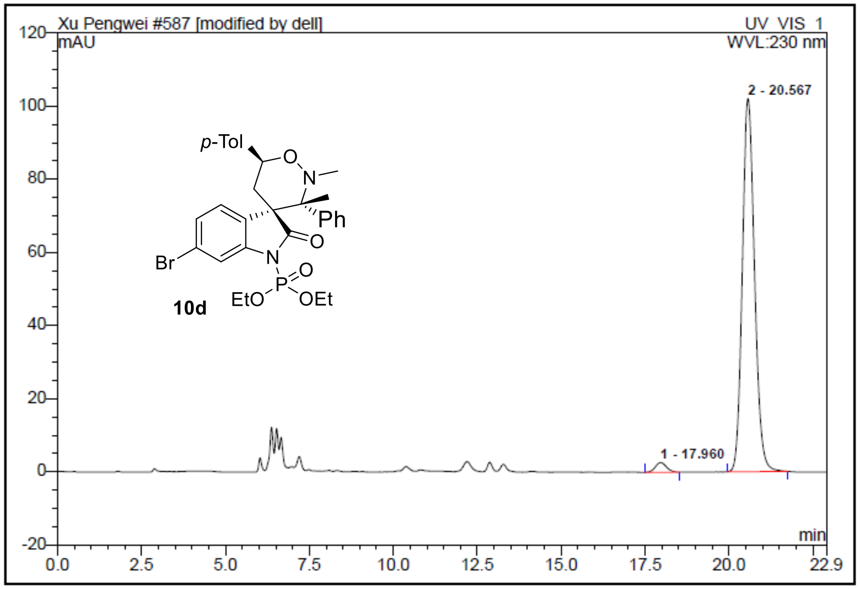

No.	Ret.Time min	Peak Name	Height mAU	Area mAU*min	Rel.Area %	Amount	Type
1	17.96	n.a.	2.660	1.011	2.28	n.a.	BMB
2	20.57	n.a.	102.040	43.349	97.72	n.a.	BMB
Total:			104.700	44.360	100.00	0.000	

589 xpw-xe-146-1p-rac-ic+if-90/10-1.0-230

Sample Name:	xpw-xe-146-1p-rac-ic+if-90/10-1.0-230	*Injection Volume:*	20.0
Vial Number:	707	*Channel:*	UV_VIS_1
Sample Type:	standard	*Wavelength:*	230
Control Program:	Xu Pengwei	*Bandwidth:*	n.a.
Quantif. Method:	Xu Pengwei	*Dilution Factor:*	1.0000
Recording Time:	2016-8-23 14:26	*Sample Weight:*	1.0000
Run Time (min):	23.00	*Sample Amount:*	1.0000

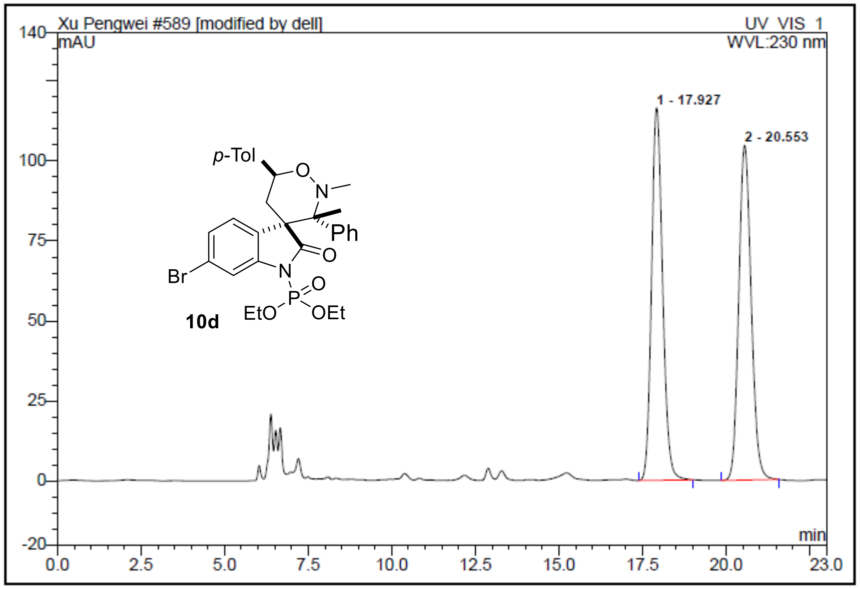

No.	Ret.Time min	Peak Name	Height mAU	Area mAU*min	Rel.Area %	Amount	Type
1	17.93	n.a.	116.510	44.812	49.93	n.a.	BMB
2	20.55	n.a.	104.661	44.935	50.07	n.a.	BMB
Total:			221.171	89.747	100.00	0.000	

Supplementary Figure 200. HPLC analysis for compound **10d**

590 xpw-xe-147-1p-asy-ic+if-90/10-1.0-230

Sample Name:	xpw-xe-147-1p-asy-ic+if-90/10-1.0-230	*Injection Volume:*	20.0
Vial Number:	708	*Channel:*	UV_VIS_1
Sample Type:	standard	*Wavelength:*	230
Control Program:	Xu Pengwei	*Bandwidth:*	n.a.
Quantif. Method:	Xu Pengwei	*Dilution Factor:*	1.0000
Recording Time:	2016-8-23 14:53	*Sample Weight:*	1.0000
Run Time (min):	22.00	*Sample Amount:*	1.0000

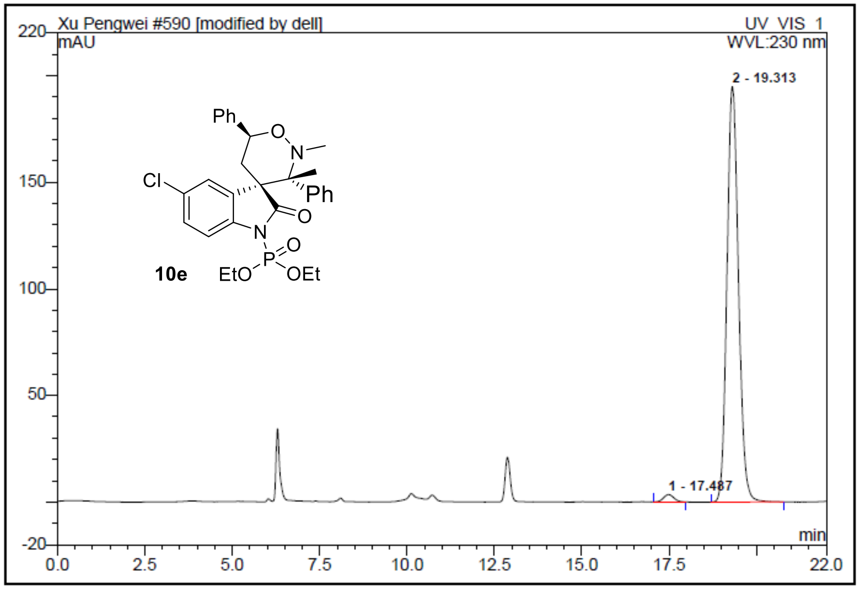

No.	Ret.Time min	Peak Name	Height mAU	Area mAU*min	Rel.Area %	Amount	Type
1	17.49	n.a.	3.481	1.192	1.58	n.a.	BMB
2	19.31	n.a.	194.803	74.342	98.42	n.a.	BMB*
Total:			198.284	75.533	100.00	0.000	

592 xpw-xe-147-1p-rac-ic+if-90/10-1.0-230

Sample Name:	xpw-xe-147-1p-rac-ic+if-90/10-1.0-230	*Injection Volume:*	20.0
Vial Number:	710	*Channel:*	UV_VIS_1
Sample Type:	standard	*Wavelength:*	230
Control Program:	Xu Pengwei	*Bandwidth:*	n.a.
Quantif. Method:	Xu Pengwei	*Dilution Factor:*	1.0000
Recording Time:	2016-8-23 15:37	*Sample Weight:*	1.0000
Run Time (min):	22.00	*Sample Amount:*	1.0000

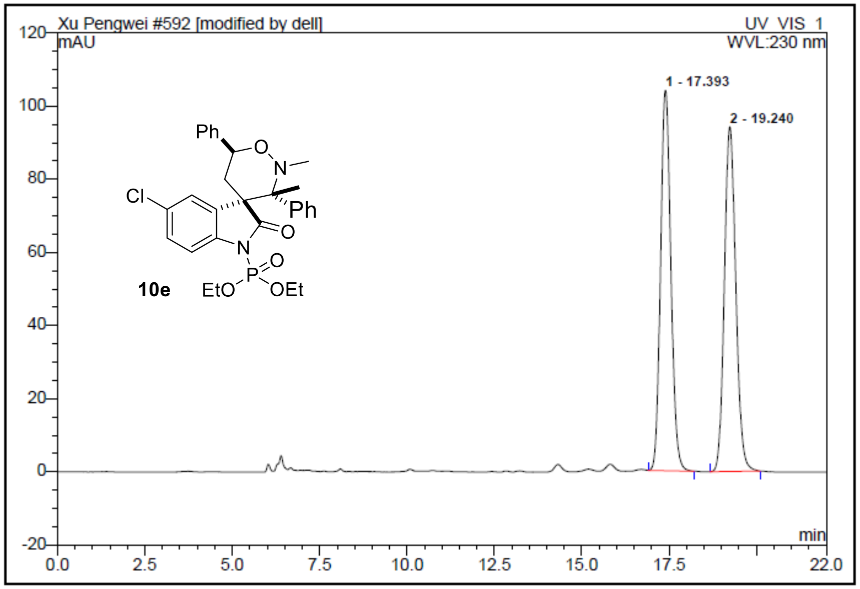

No.	Ret.Time min	Peak Name	Height mAU	Area mAU*min	Rel.Area %	Amount	Type
1	17.39	n.a.	104.054	35.668	49.94	n.a.	BMB*
2	19.24	n.a.	94.313	35.751	50.06	n.a.	BMB
Total:			198.367	71.419	100.00	0.000	

Supplementary Figure 201. HPLC analysis for compound **10e**

583 xpw-xe-148-1p-asy-if-90/10-1.0-230

Sample Name:	xpw-xe-148-1p-asy-if-90/10-1.0-230	*Injection Volume:*	20.0
Vial Number:	700	*Channel:*	UV_VIS_1
Sample Type:	standard	*Wavelength:*	230
Control Program:	Xu Pengwei	*Bandwidth:*	n.a.
Quantif. Method:	Xu Pengwei	*Dilution Factor:*	1.0000
Recording Time:	2016-8-23 9:51	*Sample Weight:*	1.0000
Run Time (min):	22.90	*Sample Amount:*	1.0000

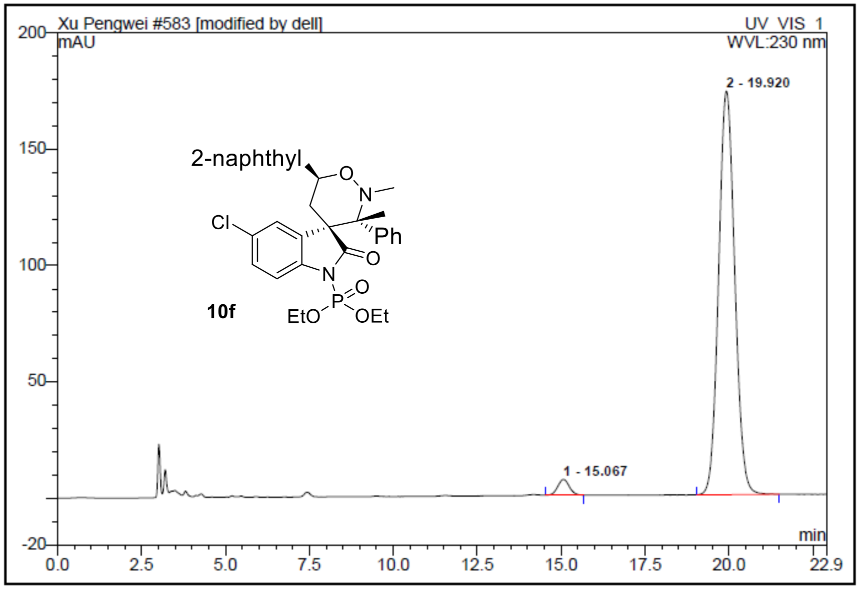

No.	Ret.Time min	Peak Name	Height mAU	Area mAU*min	Rel.Area %	Amount	Type
1	15.07	n.a.	6.791	2.725	2.75	n.a.	BMB*
2	19.92	n.a.	173.484	96.228	97.25	n.a.	BMB*
Total:			180.275	98.953	100.00	0.000	

584 xpw-xe-148-1p-rac-if-90/10-1.0-230

Sample Name:	xpw-xe-148-1p-rac-if-90/10-1.0-230	*Injection Volume:*	20.0
Vial Number:	701	*Channel:*	UV_VIS_1
Sample Type:	standard	*Wavelength:*	230
Control Program:	Xu Pengwei	*Bandwidth:*	n.a.
Quantif. Method:	Xu Pengwei	*Dilution Factor:*	1.0000
Recording Time:	2016-8-23 10:42	*Sample Weight:*	1.0000
Run Time (min):	22.00	*Sample Amount:*	1.0000

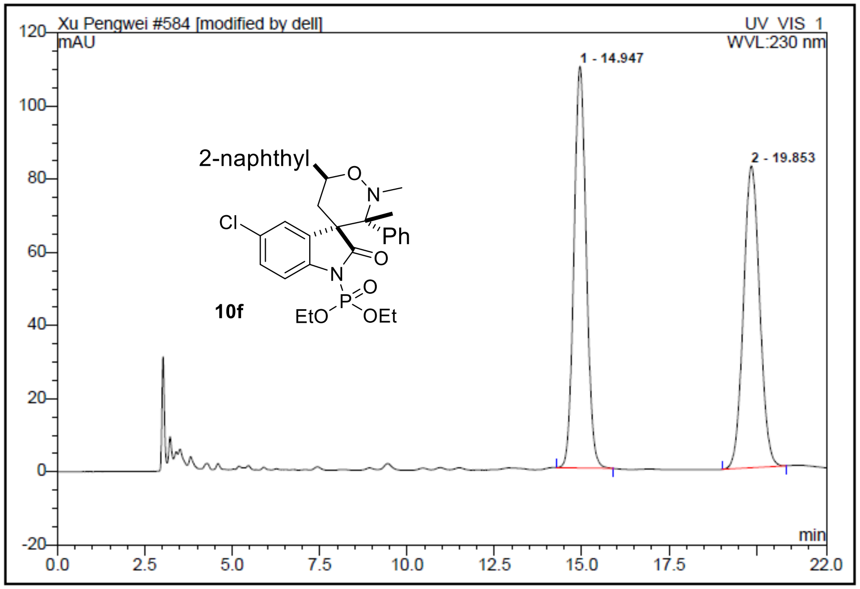

No.	Ret.Time min	Peak Name	Height mAU	Area mAU*min	Rel.Area %	Amount	Type
1	14.95	n.a.	109.866	44.686	50.10	n.a.	BMB
2	19.85	n.a.	82.574	44.513	49.90	n.a.	BMB*
Total:			192.440	89.199	100.00	0.000	

Supplementary Figure 202. HPLC analysis for compound **10f**

384 xpw-xe-12-1-asy-ie-90/10-1.0-230

Sample Name:	xpw-xe-12-1-asy-ie-90/10-1.0-230	*Injection Volume:*	20.0
Vial Number:	498	*Channel:*	UV_VIS_1
Sample Type:	standard	*Wavelength:*	230
Control Program:	Xu Pengwei	*Bandwidth:*	n.a.
Quantif. Method:	Xu Pengwei	*Dilution Factor:*	1.0000
Recording Time:	2016-4-14 15:28	*Sample Weight:*	1.0000
Run Time (min):	9.07	*Sample Amount:*	1.0000

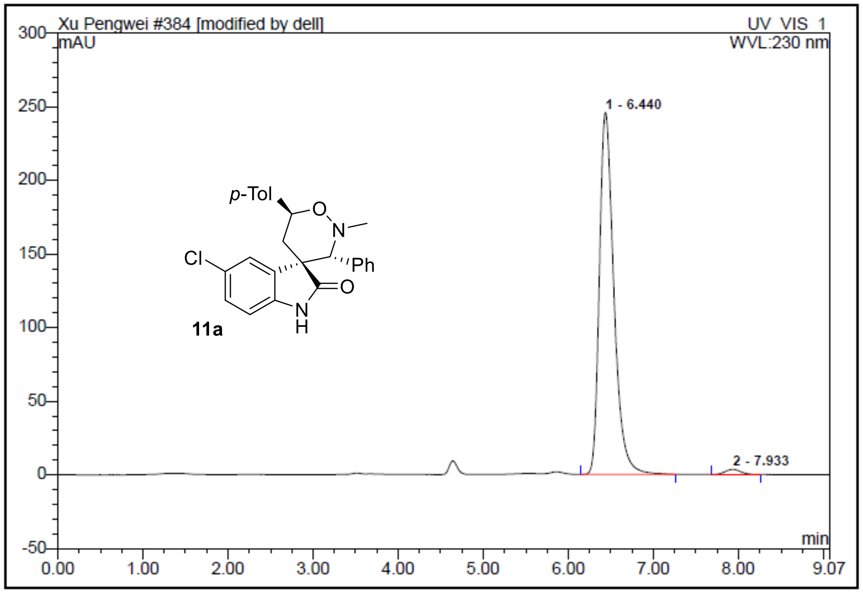

No.	Ret.Time min	Peak Name	Height mAU	Area mAU*min	Rel.Area %	Amount	Type
1	6.44	n.a.	245.857	48.429	98.50	n.a.	BMB*
2	7.93	n.a.	3.437	0.736	1.50	n.a.	BMB*
Total:			249.294	49.165	100.00	0.000	

386 xpw-xe-12-1-rac-ie-90/10-1.0-230

Sample Name:	xpw-xe-12-1-rac-ie-90/10-1.0-230	*Injection Volume:*	20.0
Vial Number:	500	*Channel:*	UV_VIS_1
Sample Type:	standard	*Wavelength:*	230
Control Program:	Xu Pengwei	*Bandwidth:*	n.a.
Quantif. Method:	Xu Pengwei	*Dilution Factor:*	1.0000
Recording Time:	2016-4-14 15:49	*Sample Weight:*	1.0000
Run Time (min):	8.99	*Sample Amount:*	1.0000

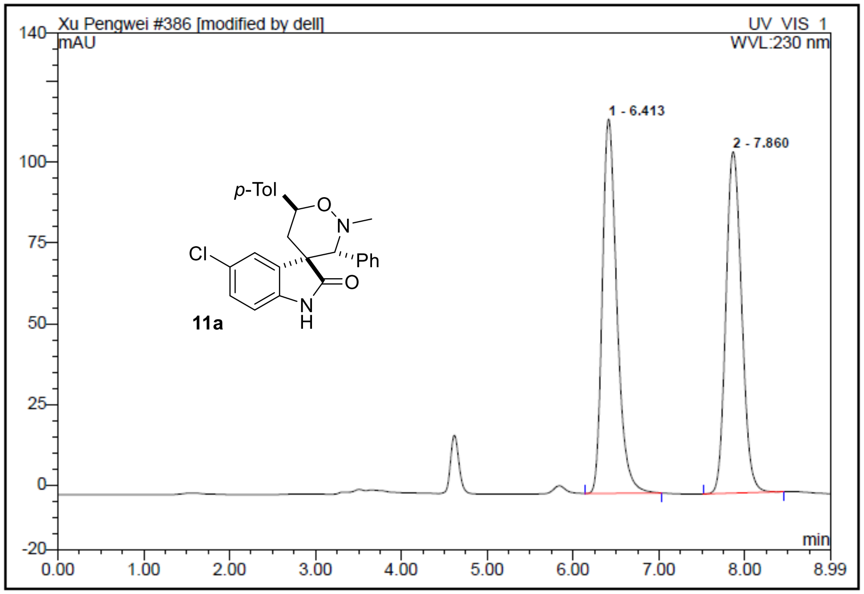

No.	Ret.Time min	Peak Name	Height mAU	Area mAU*min	Rel.Area %	Amount	Type
1	6.41	n.a.	116.092	22.755	50.25	n.a.	BMB*
2	7.86	n.a.	105.825	22.526	49.75	n.a.	BMB*
Total:			221.918	45.281	100.00	0.000	

Supplementary Figure 203. HPLC analysis for compound **11a**

405 xpw-xe-13-1-asy-ie-90/10-1.0-230

Sample Name:	**xpw-xe-13-1-asy-ie-90/10-1.0-230**	*Injection Volume:*	**20.0**
Vial Number:	**520**	*Channel:*	**UV_VIS_1**
Sample Type:	**standard**	*Wavelength:*	**230**
Control Program:	**Xu Pengwei**	*Bandwidth:*	**n.a.**
Quantif. Method:	**Xu Pengwei**	*Dilution Factor:*	**1.0000**
Recording Time:	**2016-4-17 21:51**	*Sample Weight:*	**1.0000**
Run Time (min):	**8.41**	*Sample Amount:*	**1.0000**

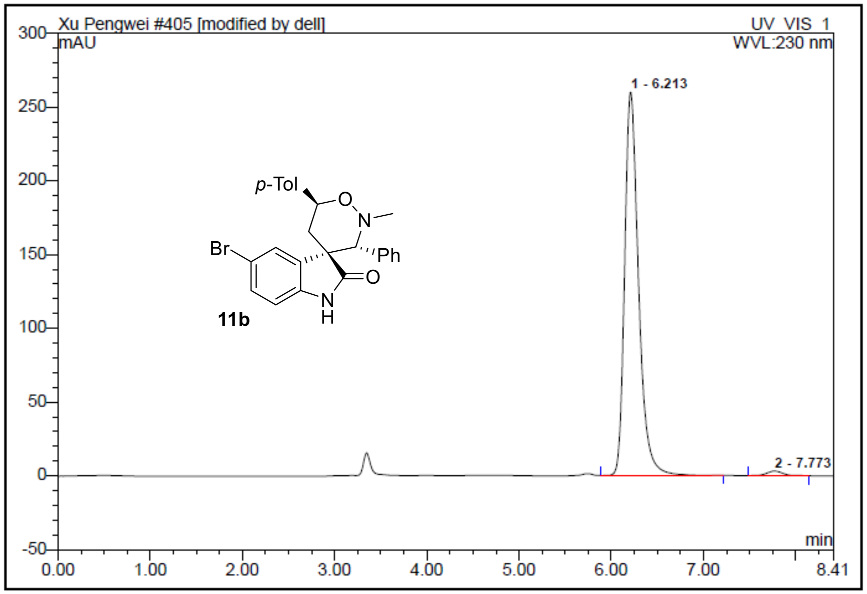

No.	Ret.Time min	Peak Name	Height mAU	Area mAU*min	Rel.Area %	Amount	Type
1	6.21	n.a.	259.981	45.478	98.60	n.a.	BMB*
2	7.77	n.a.	3.148	0.645	1.40	n.a.	BMB*
Total:			263.129	46.123	100.00	0.000	

406 xpw-xe-13-1-rac-ie-90/10-1.0-230

Sample Name:	**xpw-xe-13-1-rac-ie-90/10-1.0-230**	*Injection Volume:*	**20.0**
Vial Number:	**521**	*Channel:*	**UV_VIS_1**
Sample Type:	**standard**	*Wavelength:*	**230**
Control Program:	**Xu Pengwei**	*Bandwidth:*	**n.a.**
Quantif. Method:	**Xu Pengwei**	*Dilution Factor:*	**1.0000**
Recording Time:	**2016-4-17 22:00**	*Sample Weight:*	**1.0000**
Run Time (min):	**8.34**	*Sample Amount:*	**1.0000**

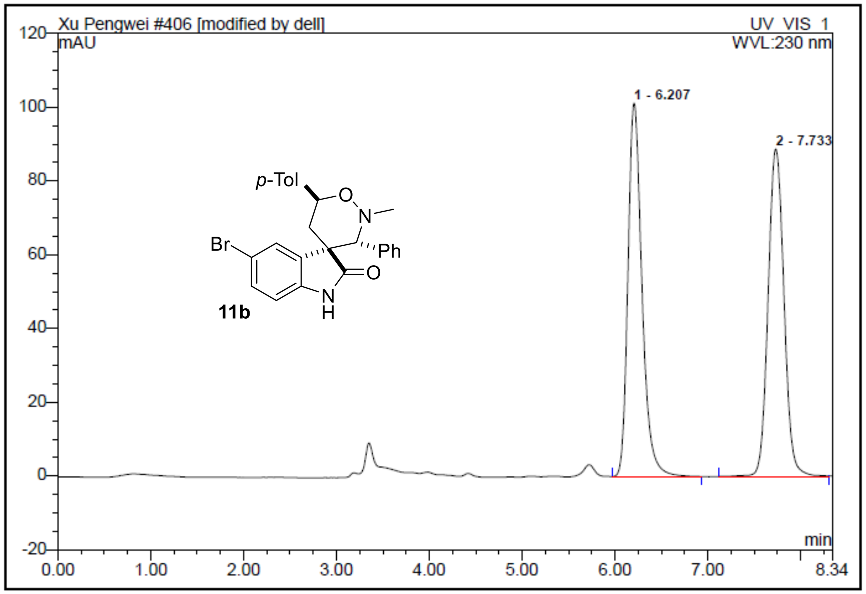

No.	Ret.Time min	Peak Name	Height mAU	Area mAU*min	Rel.Area %	Amount	Type
1	6.21	n.a.	101.335	17.763	50.13	n.a.	BMB*
2	7.73	n.a.	88.896	17.673	49.87	n.a.	BM *
Total:			190.231	35.436	100.00	0.000	

Supplementary Figure 204 HPLC analysis for compound **11b**

609 xpw-xf-09-1p-asy-ie-90/10-1.0-230			
Sample Name:	**xpw-xf-09-1p-asy-ie-90/10-1.0-230**	*Injection Volume:*	**20.0**
Vial Number:	**727**	*Channel:*	**UV_VIS_1**
Sample Type:	**standard**	*Wavelength:*	**230**
Control Program:	**Xu Pengwei**	*Bandwidth:*	**n.a.**
Quantif. Method:	**Xu Pengwei**	*Dilution Factor:*	**1.0000**
Recording Time:	**2016-8-27 21:36**	*Sample Weight:*	**1.0000**
Run Time (min):	**18.07**	*Sample Amount:*	**1.0000**

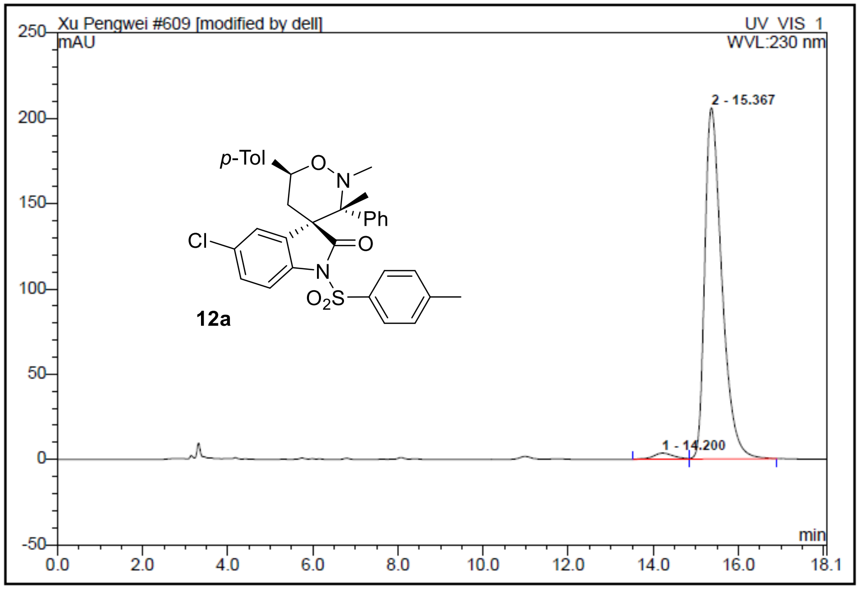

No.	Ret.Time min	Peak Name	Height mAU	Area mAU*min	Rel.Area %	Amount	Type
1	14.20	n.a.	3.537	1.936	1.98	n.a.	BM
2	15.37	n.a.	205.819	95.965	98.02	n.a.	MB
**Total:**			209.356	97.901	100.00	0.000	

611 xpw-xf-09-1p-rac-ie-90/10-1.0-230			
Sample Name:	**xpw-xf-09-1p-rac-ie-90/10-1.0-230**	*Injection Volume:*	**20.0**
Vial Number:	**729**	*Channel:*	**UV_VIS_1**
Sample Type:	**standard**	*Wavelength:*	**230**
Control Program:	**Xu Pengwei**	*Bandwidth:*	**n.a.**
Quantif. Method:	**Xu Pengwei**	*Dilution Factor:*	**1.0000**
Recording Time:	**2016-8-27 22:18**	*Sample Weight:*	**1.0000**
Run Time (min):	**18.01**	*Sample Amount:*	**1.0000**

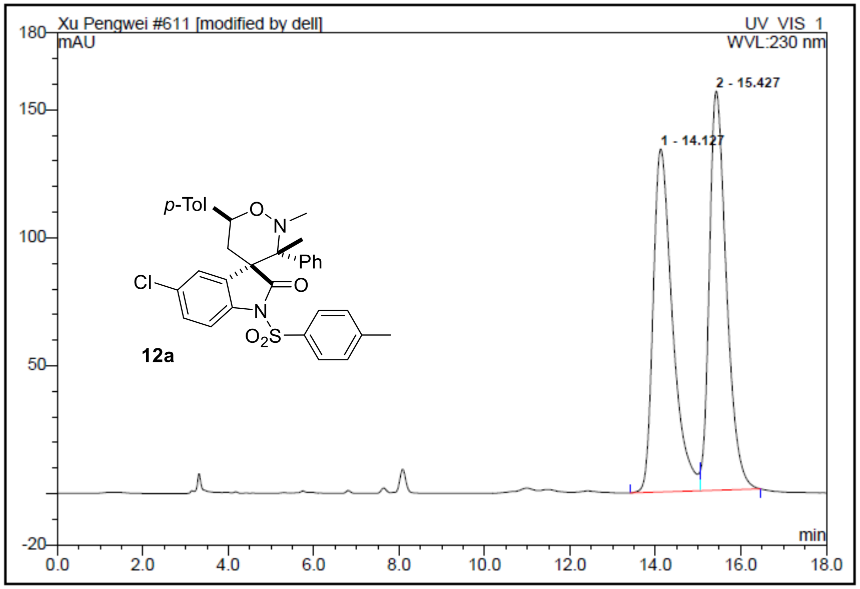

No.	Ret.Time min	Peak Name	Height mAU	Area mAU*min	Rel.Area %	Amount	Type
1	14.13	n.a.	134.005	70.595	49.37	n.a.	BM *
2	15.43	n.a.	155.985	72.392	50.63	n.a.	MB*
**Total:**			289.990	142.987	100.00	0.000	

Supplementary Figure 205. HPLC analysis for compound **12a**

614 xpw-xf-09-1p-asy-ie-90/10-1.0-230

Sample Name:	**xpw-xf-09-1p-asy-ie-90/10-1.0-230**	*Injection Volume:*	**20.0**
Vial Number:	**732**	*Channel:*	**UV_VIS_1**
Sample Type:	**standard**	*Wavelength:*	**230**
Control Program:	**Xu Pengwei**	*Bandwidth:*	**n.a.**
Quantif. Method:	**Xu Pengwei**	*Dilution Factor:*	**1.0000**
Recording Time:	**2016-8-28 12:36**	*Sample Weight:*	**1.0000**
Run Time (min):	**17.91**	*Sample Amount:*	**1.0000**

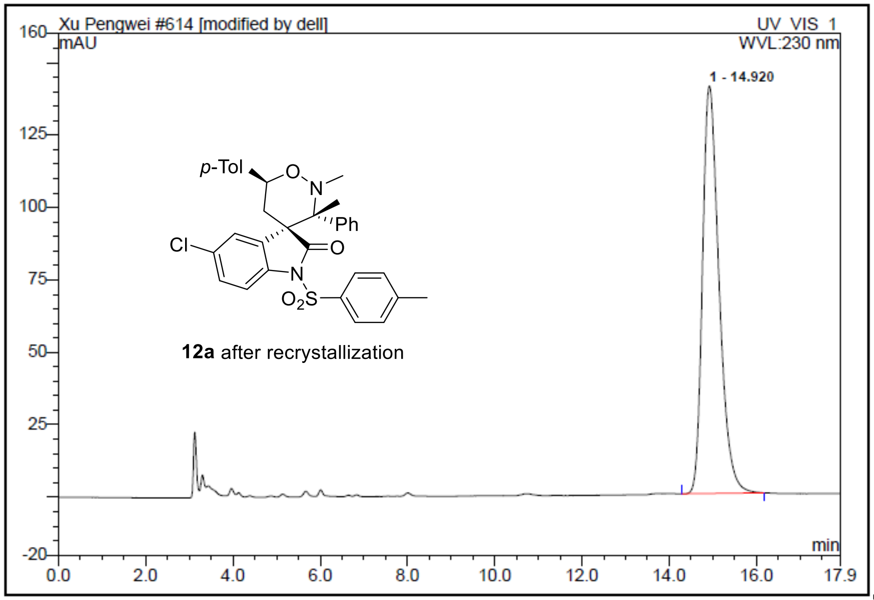

No.	Ret.Time min	Peak Name	Height mAU	Area mAU*min	Rel.Area %	Amount	Type
1	14.92	n.a.	140.730	61.838	100.00	n.a.	BMB*
Total:			140.730	61.838	100.00	0.000	

615 xpw-xf-09-1p-rac-ie-90/10-1.0-230

Sample Name:	**xpw-xf-09-1p-rac-ie-90/10-1.0-230**	*Injection Volume:*	**20.0**
Vial Number:	**733**	*Channel:*	**UV_VIS_1**
Sample Type:	**standard**	*Wavelength:*	**230**
Control Program:	**Xu Pengwei**	*Bandwidth:*	**n.a.**
Quantif. Method:	**Xu Pengwei**	*Dilution Factor:*	**1.0000**
Recording Time:	**2016-8-28 12:55**	*Sample Weight:*	**1.0000**
Run Time (min):	**17.83**	*Sample Amount:*	**1.0000**

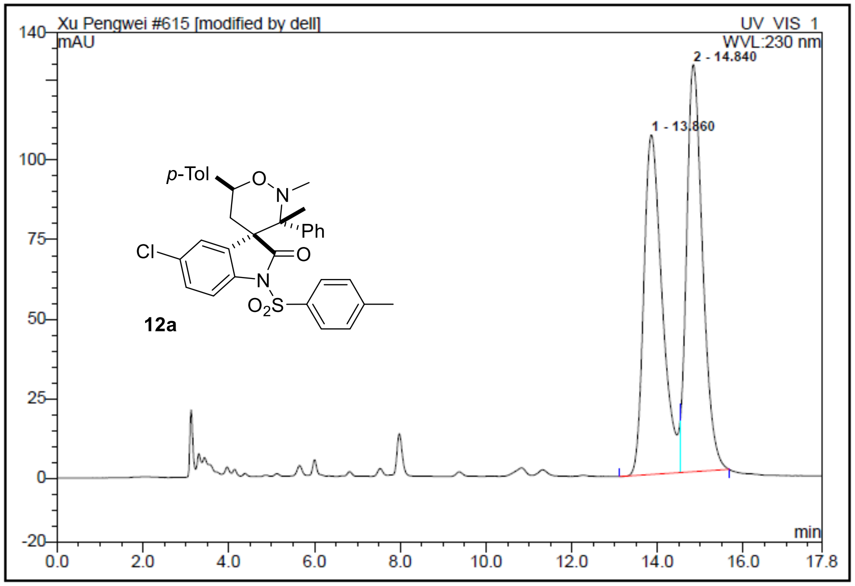

No.	Ret.Time min	Peak Name	Height mAU	Area mAU*min	Rel.Area %	Amount	Type
1	13.86	n.a.	106.735	53.375	49.16	n.a.	BM *
2	14.84	n.a.	127.992	55.203	50.84	n.a.	MB*
Total:			234.727	108.578	100.00	0.000	

Supplementary Figure 206. HPLC analysis for compound **12a** (after recrystallization)

Supplementary References

1 Tsuge, O., Sone, K., Urano, S. & Matsuda, K. Reactions of lithio trimethylsilyl compounds with nitrones. *J. Org. Chem.* **47**, 5171–5177 (1982).

2 Pfeiffer, J. Y. & Beauchemin, A. M. Simple reaction conditions for the formation of ketonitrones from ketones and hydroxylamines. *J. Org. Chem.* **74**, 8381–8383 (2009).

3 Cao, Z.-Y., Wang, X., Tan, C., Zhao, X.-L., Zhou, J. & Ding, K. Highly stereoselective olefin cyclopropanation of diazooxindoles catalyzed by a C2-symmetric spiroketal bisphosphine/Au(I) complex. *J. Am. Chem. Soc.* **135**, 8197-8200 (2013).

.
